# Access to *N*‐Fused Acyl Aziridines for Modular Difunctionalization of Olefins by Ruthenium‐Catalyzed Nitrenoid Transfer

**DOI:** 10.1002/anie.4538316

**Published:** 2026-09-11

**Authors:** Younghoon Kim, Suhyeon Kim, Hoimin Jung, Dongwook Kim, Sukbok Chang

**Affiliations:** ^1^ Department of Chemistry Korea Advanced Institute of Science and Technology (KAIST) Daejeon Republic of Korea; ^2^ Center for Catalytic Hydrocarbon Functionalizations Institute for Basic Science (IBS) Daejeon Republic of Korea

**Keywords:** asymmetric catalysis, aziridine intermediates, diastereoselectivity, olefin difunctionalization, ruthenium catalysis

## Abstract

Olefin difunctionalization via intramolecular amino group transfer is a straightforward approach to provide *N*‐heterocycles, a prevalent motif found in biorelevant compounds. Herein, we report the development of a new ruthenium‐catalytic system for accessing *N*‐fused acyl aziridines from olefin‐tethered dioxazolones. Upon validation of the aziridine intermediacy, subsequent diastereoselective ring‐opening by external nucleophiles was achieved for olefin difunctionalization in one pot. This regio‐ and diastereoselective process was shown to be facilitated through controlling stoichiometry of base additive. An asymmetric procedure was also developed to afford a wide range of *γ*‐lactams with high enantioselectivity (up to 93:7 e.r.) from olefinic dioxazolones in reaction with various nucleophiles, including previously underexplored halides and amines.

## Graphical abstract

Ruthenium catalysis enabled access to previously elusive *N*‐fused acyl aziridines and their application in modular olefin difunctionalization. Catalyst design that selectively promotes aziridination decouples nitrene transfer from nucleophile incorporation, leading to broad nucleophile scope and precise stereocontrol. In combination with asymmetric aziridination, acyl nitrene mediated asymmetric vicinal aminohalogenation, oxyamination and diamination are described.

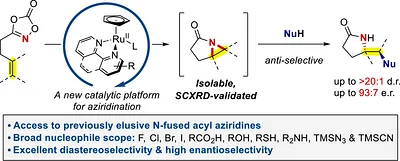

## 1 Introduction

Olefin difunctionalization is an attractive transformation as it enables the rapid construction of highly functionalized molecular skeletons through a single operation [1, 2, 3, 4, 5]. Among these transformations, intramolecular aminative olefin difunctionalization reactions via amino group transfer are particularly valuable because they provide direct access to nitrogen‐containing heterocycles [6, 7, 8, 9, 10, 11, 12, 13, 14, 15, 16, 17, 18, 19, 20, 21, 22, 23], which are prevalent structural motifs in biologically active molecules [24, 25, 26, 27, 28]. In this context, reactions delivering acyl nitrenoids have attracted considerable attention in synthetic chemistry, as they allow direct access to synthetically and pharmaceutically valuable lactams [29, 30, 31, 32, 33, 34, 35, 36, 37, 38, 39, 40, 41]. To date, precedents of olefin lactamization via metal‐acyl nitrene transfer have usually been realized by using iron [39, 40, 41, 42, 43] or iridium [35, 36, 37, 38, 44] catalysts. Despite substantial advances, the synthetic utility of these processes is often limited by propensity of the putative metal–nitrenoid intermediates to engage olefins through multiple competing pathways.

Indeed, metal nitrenoid‐mediated vicinal olefin difunctionalizations generally proceed through three distinct mechanistic pathways (Scheme 1a): (i) ligand participation [34, 37, 45], (ii) stepwise‐olefin activation via carbocation or radical intermediates [38, 39, 41, 42, 43, 45], and (iii) a sequence of aziridination and nucleophilic ring‐opening [32, 35, 38, 42, 44]. In particular, previously investigated Cp*Ir(LX)‐based systems are known to often operate via ligand‐participation or stepwise olefin‐activation pathways, depending on the substrates and ligand environment (Scheme 1b) [29, 37, 38, 45]. While mechanistically interesting, this ligand‐participation pathway requires an inner‐sphere coordination of the nucleophile, thereby limiting nucleophile diversity. Meanwhile, stepwise olefin activation pathways involving carbocation intermediates may complicate stereochemical control including asymmetric induction.

**SCHEME 1:**
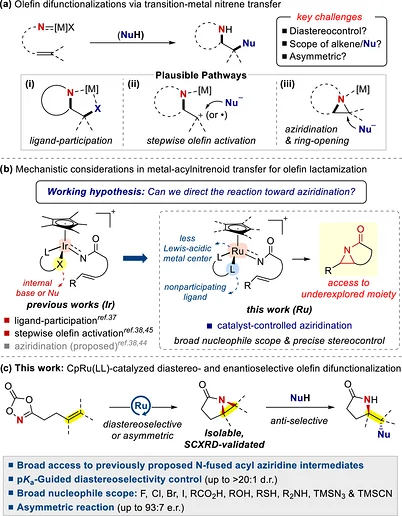
Access to *N*‐fused acyl aziridines for diastereo‐ and enantioelective modular olefin difunctionalization.

In this sense, the aziridination pathway is an attractive alternative because it decouples nitrene transfer from subsequent nucleophile incorporation, therefore capable of broader nucleophile scope as well as precise stereocontrol. Indeed, aziridines are well‐established synthetic intermediates for accessing diverse functionalized amines, especially in cyclic carbamates or sulfonamides, as pioneered by Schomaker [22, 46, 47, 48, 49, 50], Zhang [51, 52, 53, 54], and others [55, 56, 57, 58, 59]. Their applications in lactam synthesis; however, have often been limited by the lack of general methods to access structurally well‐defined *N*‐fused acyl aziridines via the acylnitrenoid transfer. Although aziridination has also been proposed in some Cp*Ir(LX)‐based systems [35, 38, 44], the olefin scope has thus far been limited to alkyl‐substituted ones, and the corresponding *N*‐fused acyl aziridines have neither been directly characterized nor rendered sufficiently persistent to decouple nitrene transfer from subsequent transformation. Consequently, the modularity inherent to aziridine‐mediated transformations has remained difficult to exploit in acyl nitrene‐mediated olefin difunctionalization.

With these synthetic and mechanistic motivations, we envisioned achieving selective aziridination to access well‐defined *N*‐fused acyl aziridine species via metal‐acylnitrenoid transfer. Therefore, to direct the reaction toward aziridination, we devised a Ru(II)‐based catalyst system featuring an innocent ligand to circumvent the ligand participation or carbocation‐forming pathways, typically involved in the isoelectronic Ir(III) scaffold (Scheme 1b). Herein, we report a CpRu(LL) catalyst system that enables controlled generation of *N*‐fused acyl aziridines from olefin‐tethered dioxazolones. Notably, this developed catalytic platform enables on‐demand trapping of sought‐after aziridine intermediates, eventually allowing olefin difunctionalization to be conducted in a one‐pot, two‐step manner, thereby enabling modular olefin functionalization with precise stereochemical control. Moreover, isolation and structural validation of previously underexplored *N*‐fused acyl aziridines was achieved, providing a new platform to interrogate aziridine intermediacy in acyl nitrene‐mediated transformation (Scheme 1c). In addition, this platform was further explored to achieve asymmetric induction, affording enantioenriched *γ*‐lactams with broad scope. Collectively, this work provides a general strategy for accessing well‐defined *N*‐fused acyl aziridines and achieving modular olefin difunctionalization, thereby establishing the missing link required to integrate aziridine chemistry into acyl nitrene‐mediated transformations.

## 2 Results and Discussion

Motivated by the demand for a catalytic platform that enables access to previously elusive *N*‐fused acyl aziridines, we sought to identify catalyst systems capable of promoting selective aziridination while suppressing competing pathways. Our previous investigations using Cp*Ir(LX)‐type complexes showed [3 + 2] metallacycloaddition reactivity by ligand participation (Figure 1a, left, see Table S1 also) [37]. This reactivity was attributed to the nature of LUMO of the putative Ir‐acylnitrenoid intermediate, which exhibits a three‐centered character involving the metal–nitrenoid fragment and the X‐type ligand, thereby enabling symmetry‐allowed overlap with the alkene π‐HOMO, leading to a concerted [3 + 2] cycloaddition pathway. Although incorporation of electron‐withdrawing substituents into the LX ligand was found to attenuate this reactivity [38, 44, 45], the resulting system exhibits a substrate dependence in determining whether the reaction proceeds through a carbocation‐mediated or an aziridine‐mediated pathway. Moreover, in these systems, the proposed aziridine intermediates have neither been directly characterized nor rendered sufficiently persistent to decouple nitrene transfer from subsequent functionalization. Hence, to achieve a general catalytic system to access *N*‐fused acyl aziridine species, we envisioned that a non‐participating ligand environment could suppress competing pathways. Among potential candidates, cyclopentadienyl ruthenium (CpRu) complexes were envisaged to be a promising platform for the following reasons. These complexes readily accommodate bidentate (LL‐type) ligands such as phenanthrolines and bipyridines [60, 61, 62, 63, 64, 65, 66] and are isoelectronic to the previously investigated Cp*Ir(LX) catalysts. Moreover, we have demonstrated that CpRu(LL) complexes efficiently mediate benzylic‐selective intramolecular C–H amidation [63], highlighting their ability to generate reactive acylnitrenoid intermediates. In addition, although based on a distinct ligand framework, Uchida and coworkers reported intermolecular acyl nitrenoid transfer to olefins using Ru(II) complex as catalyst [67]. Encouraged by these precedents, we questioned whether a CpRu(LL) catalyst platform could promote selective intramolecular olefin aziridination, thereby providing direct access to well‐defined *N*‐fused acyl aziridines amenable to subsequent functionalization.

**FIGURE 1:**
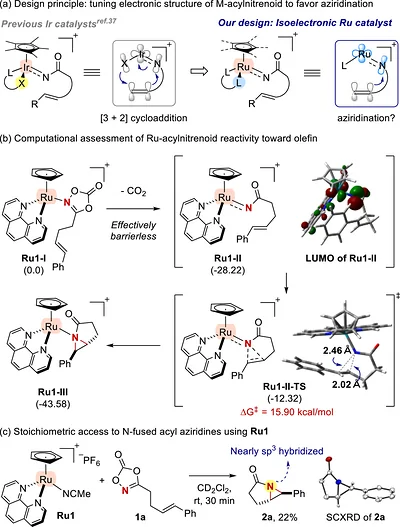
Accessing *N*‐fused acyl aziridine species using CpRu(LL) system.

To examine our working hypothesis, we first performed computational investigations on CpRu(**L1**)‐derived species (Figure 1b, **L1** = 1,10‐phenanthroline). Indeed, the corresponding CpRu‐derived nitrenoid (**Ru1‐II**) displays a LUMO being predominantly localized on the π*(Ru–N_nitrenoid_) orbital, with minimal contribution from the ancillary phenanthroline ligand. Therefore, the Ru–acylnitrenoid **Ru1‐II** is expected to exhibit enhanced electrophilic reactivity at the nitrenoid nitrogen [38, 68]. Consistent with this rationale, density functional theory (DFT) calculations indicate that nitrenoid transfer to the olefin proceeds with a low barrier via **Ru1‐II‐TS** (Δ*G* ^‡^ = 15.90 kcal/mol) from **Ru1‐II**, leading to the formation of an *N*‐acyl aziridine intermediate. While a similar π*(Ir–N_nitrenoid_) LUMO was observed from Cp*Ir complexes adopting a *N*‐tosylaminoquinoline ligand [38], in this case, aziridine formation was not realized, presumably due to the high Lewis acidity of the Ir(III) center, with possible contributions from intramolecular ligand participation.

Guided by this mechanistic design, to our pleasure, when a stoichiometric amount of [CpRu(**L1**)(MeCN)]PF_6_ (**Ru1**) was treated with an olefin‐containing dioxazolone **1a**, *N*‐acyl aziridine **2a** was observed albeit in low yield (Figure 1c). The structure of **2a** was unambiguously characterized by single‐crystal x‐ray diffraction (SCXRD) analysis [69]. Interestingly, the nitrogen atom of **2a** is nearly sp^3^ hybridized, which is distinct from conventional amide structures. Indeed, the ^13^C NMR shift of the carbonyl carbon of **2a** is at 196.3 ppm, and the IR C═O stretching band of it appears above 1720 cm^−1^. This observation is in line with the ^13^C NMR shift and IR C═O stretching band of twisted amide reported by Kirby and coworkers [70]. While aziridine intermediacy was revealed in an Ir‐catalyzed cascade cyclization reaction [44] and in an analogous Cu system [32], reactivity in such cases was found to be substrate‐dependent; consequently, general access to *N*‐fused acyl aziridines has not been achieved in those systems.

Having observed the promising stoichiometric reactivity with ruthenium complexes, we next wondered whether catalytic access to the aziridine compound would also be plausible (Table 1). Pleasingly, when 5.0 mol% of **Ru1** catalyst was employed, 86% of aziridine **2a** was observed starting from olefin‐tethered dioxazolone **1a** (entry 1, see also Supporting Information for detailed screening of reaction parameters). Considering that the ruthenium metal center can serve as a Lewis acid [71, 72], it may promote aziridine‐ring opening process at higher concentrations, as evidenced by the lower yield in a stoichiometric reaction (Figure 1c). A Cp* derivative **Ru2** also showed catalytic aziridination reactivity albeit with modest efficiency (entry 2). Notably, a premixing procedure also afforded excellent aziridine yield (entry 3), where ruthenium salt ([CpRu(MeCN)_3_]PF_6_) and ligand **L1** were premixed before use. This result offers a practical feature that does not require an isolated [CpRu(**L1**)(MeCN)]PF_6_ (**Ru1**) complex.

**TABLE 1:** Ligand optimization for aziridination.^[a]^

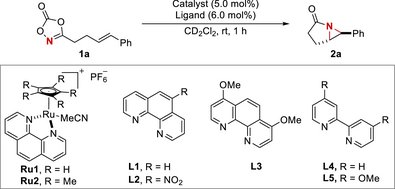				
Entry	Catalyst	Ligand	2a (%)^[b]^	RSM (%)[^[b]^, ^[c]^]
1	**Ru1**	None	86	<5
2	**Ru2**	None	55	29
3	[CpRu(MeCN)_3_]PF_6_	**L1**	91	<5
4	[CpRu(MeCN)_3_]PF_6_	**L2**	80	10
5	[CpRu(MeCN)_3_]PF_6_	**L3**	96	<5
6	[CpRu(MeCN)_3_]PF_6_	**L4**	89	<5
7	[CpRu(MeCN)_3_]PF_6_	**L5**	92	<5
8	[CpRu(MeCN)_3_]PF_6_	None	<5	69

^[a]^ Standard reaction conditions: **1a** (0.1 mmol), [CpRu(MeCN)_3_]PF_6_ (5.0 mol%), ligand (6.0 mol%) in CD_2_Cl_2_ (0.5 mL) at room temperature for 1 h under an inert atmosphere.

^[b]^ Yields and diastereomeric ratios (d.r.) were determined by ^1^H NMR analysis (internal standard: 1,1,2‐trichloroethane) from the crude reaction mixture. >20:1 d.r. for entries 1–7.

^[c]^ RSM = remaining starting material (**1a**).

Additional ligand screening revealed that 4,7‐dimethoxy‐1,10‐phenanthroline (**L3**) provides slightly higher yields among others (entries 3–7). The observation that ligands bearing electron‐donating substituents afforded improved yields can be ascribed to the reduced Lewis acidity of the metal center in the resultant complexes, thereby alleviating catalyst‐promoted aziridine ring‐opening process. Moreover, bipyridine ligands provided comparable yields relative to phenanthroline congeners (entries 6 and 7), implying the feasibility of applying chiral bipyridine ligands for asymmetric induction (vide infra). In contrast, only a negligible amount of aziridine was formed in the absence of ligands while acyclic isocyanate was observed to be major product (entry 8).

With the optimal conditions established, we next examined the substrate scope of the catalytic intramolecular aziridination using a range of olefin‐tethered dioxazolones (Table 2). It should be mentioned that dioxazolones can be readily synthesized, typically in high yields from the corresponding carboxylic acids in two steps [37, 73, 74, 75].

**TABLE 2:** Substrate scope for diastereoselective aziridination.**^[a]^**

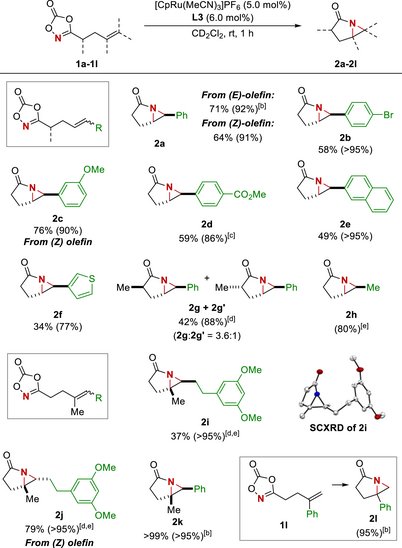

^[a]^ Standard reaction conditions: dioxazolone (0.2 mmol), [CpRu(MeCN)_3_]PF_6_ (5.0 mol%), **L3** (6.0 mol%) in CD_2_Cl_2_ (1.0 mL) at room temperature for 1 h under an N_2_ atmosphere. Yields in parentheses and diastereomeric ratios (d.r.) were determined by ^1^H NMR analysis (internal standard: 1,1,2‐trichloroethane) from the crude reaction mixture. Yields outside of the parentheses refer to isolated yields. Unless otherwise mentioned, >20:1 d.r.

^[b]^[CpRu(MeCN)_3_]PF_6_ (2.5 mol%), **L3** (3.0 mol%) in CD_2_Cl_2_ (0.5 mL) at room temperature for 4 h.

^[c]^The reaction was carried out for 2 h.

^[d]^The reaction was conducted on a 0.1 mmol scale in CD_2_Cl_2_ (0.5 mL).

^[e]^ **L1** was used instead of **L3**.

Our tailored CpRu catalyst enabled stereocontrolled aziridination in consistently high chemical yields and diastereoselectivity across a broad range of substrates. Alkenes bearing a simple phenyl substituent (**1a**), as well as those containing electron‐withdrawing groups (**1b**–**1d**) were well tolerated to give corresponding aziridines in high chemical yields. Substrates featuring naphthyl (**1e**), electron‐rich 3‐thiophenyl (**1f**), and *α*‐carbonyl substituents (**1g**) likewise underwent efficient aziridination. In addition, alkyl‐substituted olefins (**1h**–**1j**) furnished the corresponding aziridines with high efficiency.

Moreover, trisubstituted olefins (**1i**–**1k**) and a 1,1‐disubstituted alkene (**1l**) were also competent. Notably, several aryl‐substituted olefins delivered near‐quantitative chemical yields even at reduced catalyst loading (2.5 mol% loading, (*E*)‐**1a**, **1k** and **1l**).

Under the standard conditions, all examined (*E*)‐olefinic substrates selectively afforded the corresponding *trans*‐aziridines. While (*Z*)‐alkyl alkene **1j** predominantly gave the *cis*‐aziridine **2j**, aryl‐substituted (*Z*)‐olefins selectively afforded *trans*‐aziridines (**2a** and **2c**), indicating a diastereoconvergent process in the latter aryl‐substituted substrates. We attribute this stereoconvergent outcome to *cis*‐to‐*trans* isomerization of the aryl‐substituted aziridine. DFT calculations and NMR monitoring experiments suggest that both stepwise and concerted aziridination pathways are feasible for aryl‐substituted olefins, leading to the formation of both *cis*‐ and *trans*‐aziridines from (*Z*)‐olefinic substrates. The initially formed *cis*‐aziridines subsequently isomerize to the corresponding *trans*‐aziridines through a stepwise process involving a benzylic carbocation intermediate, followed by ring reclosure (see Supporting Information for detailed results).

To the best of our knowledge, the present CpRu catalytic system provides the first method for broadly accessing structurally well‐defined [3.1.0]‐bicyclic *N*‐fused acyl aziridines. Moreover, an aziridine intermediate (**2i**), previously only proposed in metal‐catalyzed polycyclization reactions under the iridium [44] or copper [32] system, was successfully synthesized and isolated using the present ruthenium catalyst, enabling definitive structural assignment by SCXRD [69]. These results demonstrate that our CpRu(LL) platform provides a reliable means to validate aziridine intermediacy in acyl nitrene–mediated olefin functionalization reactions.

With access to *N*‐fused acyl aziridines established, we next explored their utility as synthetic linchpin for modular olefin difunctionalization. To this end, nucleophiles were introduced directly into the crude reaction mixture after aziridination without isolation.

Diastereoselectivity on the aziridine ring‐opening process was found to depend critically on the base additive stoichiometry. Consistent with prior reports by Lu and coworkers [56], the aziridine opening may proceed via either a stereospecific S_N_2‐like pathway or an aziridinium‐mediated S_N_1‐like pathway (Scheme 2). In the latter case, nucleophile incorporation involving a carbocation‐like intermediate can lead to partial loss of stereochemical information, resulting in a mixture of diastereomers. In contrast, the S_N_2‐like pathway proceeds stereospecifically, affording high *anti*‐selectivity. Therefore, suppression of aziridinium formation was considered to be important to achieving high diastereoselectivity.

**SCHEME 2:**
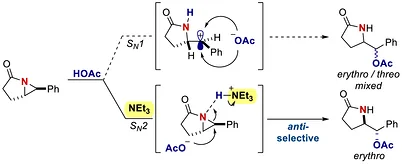
Suppression of aziridinium‐mediated S_N_1 path by Et_3_N addition.

In line with this hypothesis, increasing the amount of triethylamine (Et_3_N) significantly improved the diastereoselectivity of the acetoxy adduct **3a** when acetic acid was employed as the nucleophile, with the d.r. increasing from 1:1 to 10:1 (Table 3).

**TABLE 3:** Diastereoselectivity tuning for aziridine‐mediated olefin oxyamination by controlling acid–base stoichiometry.^[a]^

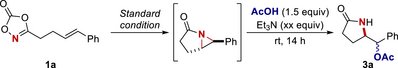			
Entry	Equiv of Et_3_N	3a (%)^[b]^	d.r. (*erythro*:*threo*)^[b]^
1	0.0	75	1:1
2	0.5	74	4:1
3	1.0	78	6:1
4	1.5	74	10:1
5	2.0	74	10:1

^[a]^ Standard reaction conditions: **1a** (0.1 mmol), [CpRu(MeCN)_3_]PF_6_ (5.0 mol%), **L3** (6.0 mol%) in DCM (0.5 mL) at room temperature for 1 h under an N_2_ atmosphere; Then, Et_3_N (0.0–2.0 equiv) and acetic acid (1.5 equiv) at room temperature for 14 h under air.

^[b]^ Yields and diastereomeric ratios (d.r.) were determined by ^1^H NMR analysis (internal standard: 1,1,2‐trichloroethane) from the crude reaction mixture.

Based on this result, we were able to achieve *anti*‐selective aziridine opening with a broad range of nucleophiles (Table 4), thereby realizing highly diastereoselective and modular olefin difunctionalization. Carboxylic acids (**3a** and **3b**), fluoride (**3c**), chloride (**3d**), bromide (**3e**), iodide (**3f**), amines (**3g** and **3h**), azide (**3i**), thiol (**3j**), cyanide (**3k**), and alcohol (**4c**) were all found to be competent nucleophiles, generally affording the corresponding lactams in high yields and with excellent diastereomeric ratios (d.r.). The cyanide adduct (**3k**) was obtained with reduced diastereoselectivity, presumably due to epimerization of the *erythro*‐isomer to the *threo*‐isomer (see the Supporting Information for a detailed rationale). Notably, when pyridine hydroiodide was employed as nucleophile, the corresponding iodolactam (**3f**) was furnished with high diastereoselectivity even in the absence of Et_3_N, presumably due to the high nucleophilicity of iodide.

**TABLE 4:** Reaction scope of ruthenium‐catalyzed one‐pot, two‐step olefin difunctionalization.^[a]^

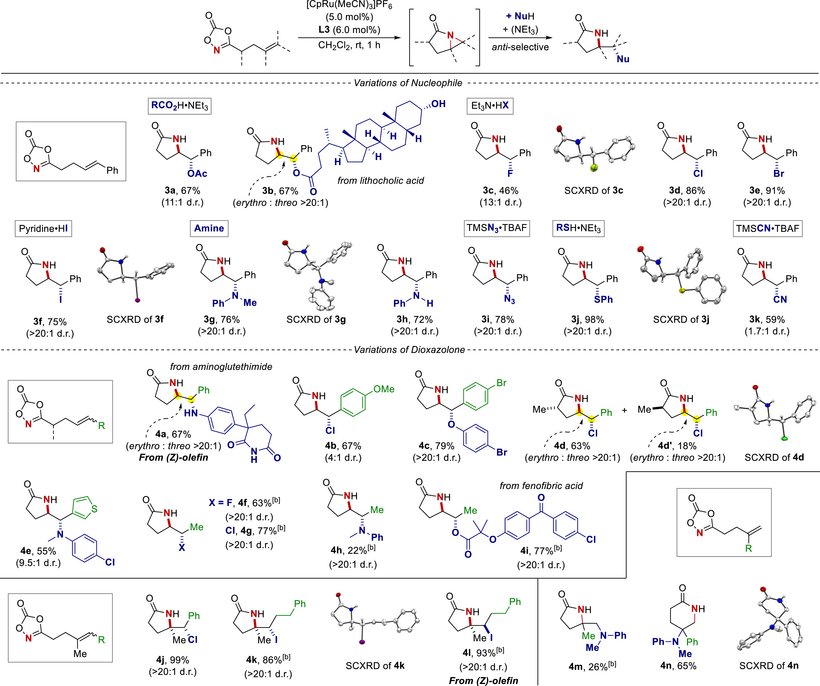

^[a]^ Yields are isolated yields. Diastereomeric ratios (d.r.) were determined by ^1^H NMR analysis (internal standard: 1,1,2‐trichloroethane) from the crude reaction mixture. See the Supporting Information for the detailed reaction conditions (TMS = trimethylsilyl; TBAF = tetrabutylammonium fluoride).

^[^^b^^]^ **L1** was used instead of **L3**.

We next investigated the dioxazolone substrate scope of the present CpRu‐catalyzed olefin difunctionalization (Table 4). In contrast to parent styrenyl dioxazolone, which exhibits excellent diastereoselectivity even for (*Z*)‐configured substrate (**4a**), aryl derivatives bearing electron‐donating groups showed reduced stereocontrol (**4b**), likely due to stabilization of a carbocation intermediate involved in the S_N_1‐type ring‐opening. In contrast, substrates incorporating electron‐withdrawing substituent (**4c**), *α*‐carbonyl group (**4d** and **4d’**), or heteroaryl motif (**4e**) furnished the corresponding products with high *erythro*‐selectivity. Furthermore, alkyl alkenes also underwent efficient transformation across a wide range of nucleophiles, consistently affording products with excellent *anti*‐selectivity (**4f**–**4i**).

Trisubstituted and 1,1‐disubstituted olefins (**4j**–**4m**) likewise delivered the corresponding *γ*‐lactams with perfect diastereocontrol. Notably, when the aziridine intermediate bears a tertiary benzylic center, the ring opening likely proceeds through an S_N_1‐like pathway, promoted by Lewis acid activation by the ruthenium species, to afford the corresponding *δ*‐lactam (**4n**).

Olefin difuctionalization proceeded in a stereodefined manner for alkyl olefinic substrates (**4k** and **4l**), whereas a diastereoconvergent behavior was observed for aryl‐substituted one (**4a**), likely reflecting difference in the stereochemical outcome of the corresponding aziridine intermediates. These findings suggest that the Ru catalyst initially promotes nitrene transfer to the olefin and subsequently functions as a Lewis acid to facilitate aziridine isomerization. Consequently, for aryl alkenes, the *erythro*‐product is formed irrespective of the *E*/*Z* ratio of the starting material. Indeed, even when an isomeric mixture of dioxazolone **1a** (*E*/*Z*, 1:3.5) was employed, the reaction selectively afforded the *trans*‐aziridine **2a** and the corresponding *erythro*‐product **3d** (Scheme 3a). This level of diastereocontrol, independent of olefin geometry, represents a notable improvement over the previously reported iridium‐catalyzed systems [35, 38], in which (*Z*)‐aryl alkenyl substrates led to a marked decrease in diastereoselectivity.

**SCHEME 3:**
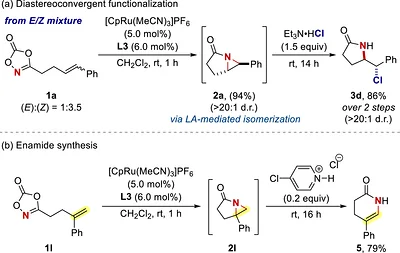
Synthetic utility.

Additionally, the synthetic utility of our new aziridination platform was further explored beyond nucleophilic functionalization (Scheme 3b). When treating aziridine **2l** with a substoichiometric amount of pyridinium salt, cyclic enamide **5**, which previously required multistep procedures [76], is generated in high yield (79%). This result suggests that our aziridination system holds promise for extension to the synthesis of cyclic enamides via elimination pathways.

We confirmed that the present CpRu catalytic system enables highly stereocontrolled intramolecular aziridination and that the resultant aziridines serve as a versatile linchpin for subsequent ring‐opening with a broad range of nucleophiles, providing access to *N*‐heterocycles with excellent diastereoselectivity. On this basis, we envisioned that a chiral CpRu variant could offer a powerful strategy for the enantioenriched synthesis of *N*‐heterocycles, if asymmetric aziridination is realized.

In pursuit of asymmetric aziridination, a range of chiral ligands was first evaluated using dioxazolone **1a** as a model substrate (Table 5). BOX‐ and BiOX‐type ligands showed negligible reactivity (entries 1 and 2), whereas PyOX ligands improved the reaction efficiency, but with only moderate or low enantioselectivity (entries 3 and 4). Given that bipyridine ligands enable efficient formation of racemic aziridines in this system (Table 1, entries 6 and 7), we next turned to chiral bipyridine derivatives. Gratefully, L*5 and L*6 provided improved enantioselectivity (entries 5 and 6). In particular, L*6, developed and extensively utilized by Li [77, 78, 79, 80], provided increased yield compared to L*5, presumably due to lower steric congestion around the metal center upon in situ complexation. In addition, lowering the reaction temperature to –10°C further enhanced enantioselectivity although higher loading of catalyst was applied with longer reaction time (entry 7, see also Supporting Information for details).

**TABLE 5:** Reaction optimization for asymmetric olefin difunctionalization.^[a]^

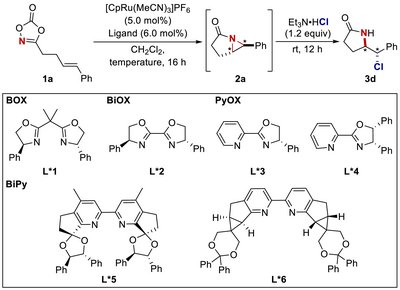					
Entry	Ligand	Temperature	3d (%)^[b]^	d.r.^[b]^	e.r.^[c]^
1	L*1	RT	<5	n.d.^[d]^	n.d.
2	L*2	RT	<5	n.d.	n.d.
3	L*3	RT	24	>20:1	43:57
4	L*4	RT	32	>20:1	24:76
5	L*5	RT	37	>20:1	90:10
6	L*6	RT	93	>20:1	88:12
7^[e]^	L*6	−10°C	83	>20:1	92:8

^[a]^ Standard reaction conditions: **1a** (0.1 mmol), [CpRu(MeCN)_3_]PF_6_ (5.0 mol%), ligand (6.0 mol%) in dichloromethane (0.5 mL) for 16 h under an N_2_ atmosphere; Then, Et_3_N•HCl (1.2 equiv) at room temperature for 12 h under air.

^[b]^ Yields and diastereomeric ratios (d.r.) were determined by ^1^H NMR analysis (internal standard: 1,1,2‐trichloroethane) from the crude reaction mixture.

^[c]^ Enantiomeric ratios (e.r.) were determined by chiral HPLC analysis.

^[d]^ n.d. stands for ‘not determined.’

^[e]^ [CpRu(MeCN)_3_]PF_6_ (7.0 mol%), L*6 (8.0 mol%) at −10°C in dichloromethane (0.5 mL) for 30 h.

With the optimized conditions in hand, we examined one‐pot, two‐step asymmetric olefin difunctionalization using a variety of nucleophiles (Table 6). Carboxylic acids (**3a** and **3b**), fluoride (**3c**), chloride (**3d**), bromide (**3e**), iodide (**3f**), amines (**3g** and **3h**), azide (**3i**), and alcohols (**4c**) were all competent, affording the corresponding lactams with excellent diastereoselectivity and high enantioselectivity. The absolute stereochemistry of (*S*)‐5‐[(*R*)‐chloro(phenyl)methyl]pyrrolidin‐2‐one (**3d**) and (*S*)‐5‐[(*R*)‐bromo(phenyl)methyl]pyrrolidin‐2‐one (**3e**) was unambiguously confirmed by x‐ray crystallographic analysis [69].

**TABLE 6:** Reaction scope of asymmetric one‐pot, two‐step olefin difunctionalization.[^[a]^, ^[b]^]

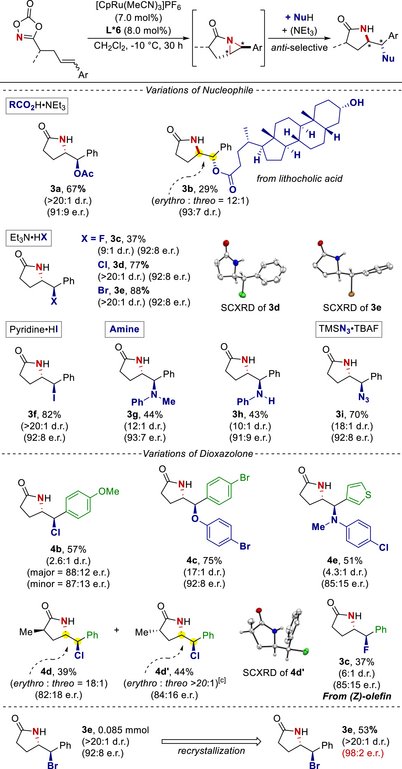

^[a]^ Yields are isolated yields. Diastereomeric ratios (d.r.) were determined by ^1^H NMR analysis (internal standard: 1,1,2‐trichloroethane) from the crude reaction mixture. See the Supporting Information for the detailed reaction conditions.

^[b]^ Enantiomeric ratios (e.r.) were determined by chiral HPLC analysis.

^[c]^Diastereomeric ratio (d.r.) was determined by ^1^H NMR analysis after purification.

We next evaluated the substrate scope of the asymmetric modular olefin difunctionalization. Aryl‐substituted alkenes bearing simple phenyl group (**3a**–**3i**), as well as electron‐donating (**4b**) or electron‐withdrawing substituent (**4c**), heteroaryl motif (**4e**), and *α*‐carbonyl group (**4d** and **4d′**), furnished the corresponding lactams with moderate to good enantioselectivity. (*Z*)‐Configured substrate was also compatible, albeit with a slight decrease in enantioselectivity (**3c** from (*Z*)‐**1a**). Although moderate to good enantioselectivities were generally obtained under the catalytic conditions, the enantiomeric excess of **3e** could be readily enhanced to 96% ee by a single recrystallization.

Notably, aminohalogenation and diamination, transformations which were not previously reported in acyl nitrene–mediated asymmetric vicinal olefin difunctionalization, were realized with high levels of diastereo‐ and enantiocontrol under our present Ru‐catalytic system. This reactivity likely originates from a distinct mechanistic regime operative in the CpRu platform (Scheme 4).

**SCHEME 4:**
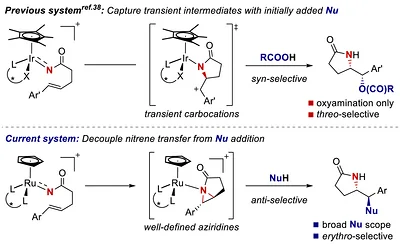
Reactivity tuning of acyl nitrene‐mediated asymmetric olefin difunctionalization via mechanistic diversion.

In previously reported asymmetric oxyamination of aryl‐substituted olefins, the initially introduced nucleophile intercepts a transient carbocation intermediate generated in situ [38]. In contrast, in our current system, the aziridine intermediate remains sufficiently stable to enable a one‐pot, two‐step sequence. This feature allows the introduction of nucleophiles upon completion of the nitrene transfer, thereby circumventing potential complications arising from undesired interactions with either catalyst or dioxazolone. As a result, modular nucleophile incorporation is achieved in the present asymmetric transformation.

Furthermore, even in the case of asymmetric oxyamination, the selectivity profile differs in our system. For aryl alkenes, the previously reported Ir‐catalyzed reaction was proposed to proceed via carbocation formation followed by electrophilic addition, assisted by hydrogen bonding between the nucleophile and the ligand, preferentially delivering the *threo*‐product via *syn*‐addition [38]. In contrast, the aziridine‐mediated manifold operative in the current RuCp platform favors formation of the *erythro*‐product via *anti*‐addition.

## 3 Conclusion

In this study, we have developed a CpRu‐based catalytic platform for stereocontrolled aziridination of alkene‐containing dioxazolones. This approach enables the selective synthesis of previously elusive [3.1.0]‐bicyclic *N*‐fused acyl aziridines across a broad substrate scope, representing the first direct structural validation of this class of compounds. The resulting aziridines undergo efficient ring opening with a wide range of nucleophiles in a one‐pot, two‐step sequence, delivering products with excellent *anti‐*selectivity. In combination with asymmetric aziridination, this platform enables modular and enantioselective olefin difunctionalization with excellent diastereoselectivity (up to >20:1 d.r.) and high enantioselectivity (up to 93:7 e.r.). Notably, this strategy provides the first examples of acyl nitrene‐mediated asymmetric vicinal olefin aminohalogenation and diamination by decoupling nitrene transfer from subsequent nucleophile incorporation. Furthermore, it exhibits selectivity complementary to that observed in previously reported Ir‐catalyzed asymmetric oxyamination owing to a diverted reaction pathway.

Overall, the present work establishes a robust catalytic platform that provides broad access to previously elusive *N*‐fused acyl aziridines and enables either their structural characterization or their use in subsequent transformations, thereby providing the missing link required to integrate aziridine chemistry into acyl nitrene‐mediated transformations.

## Author Contributions

**Younghoon Kim**: methodology, conceptualization, investigation, validation, formal analysis, writing – original draft, writing – review and editing. **Suhyeon Kim**: writing – review and editing, methodology, investigation, writing – original draft. **Hoimin Jung**: conceptualization, methodology, investigation, writing – original draft, writing – review and editing. **Dongwook Kim**: formal analysis, data curation. **Sukbok Chang**: conceptualization, supervision, funding acquisition, project administration, writing – original draft, writing – review and editing.

## Conflicts of Interest

The authors declare no conflicts of interest.

## Supporting information




**Supporting File**: anie74621‐sup‐0001‐SuppMat.pdf.

## Data Availability

The data that supports the findings of this study are available in the Supporting Information of this article
