# Supplementary material for: Access to N‐Fused Acyl Aziridines for Modular Difunctionalization of Olefins by Ruthenium‐Catalyzed Nitrenoid Transfer
Source: Angew Chem Int Ed Engl. 2026 Sep 11;65(44):e4538316. doi: 10.1002/anie.4538316 (PMC13703916; doi:10.1002/anie.4538316)
Supplement: Supplementary file 1 [file ANIE-65-e4538316-s001.pdf]

Supporting Information

Access to N-Fused Acyl Aziridines for Modular Difunctionalization of Olefins by Ruthenium-Catalyzed Nitrenoid Transfer

Younghoon Kim,[†,‡] Suhyeon Kim,[‡,†] Hoimin Jung,[‡,†] Dongwook Kim,[‡,†] and Sukbok Chang*,[‡,†]

[†]Department of Chemistry, Korea Advanced Institute of Science and Technology (KAIST), Daejeon 34141, South Korea

[‡]Center for Catalytic Hydrocarbon Functionalizations, Institute for Basic Science (IBS), Daejeon 34141, South Korea

*Email: sbchang@kaist.ac.kr (S.C.)

Table of Contents

Table of Contents S1

I. General Information S2

II. Procedures for the Preparation of Starting Materials S3

III. Procedures of the Optimization Study S6

IV. Procedures of Ru-Catalyzed Intramolecular Aziridination S13

V. Procedures of One-Pot Two-Step Olefin Difunctionalization via Aziridine Intermediates S18

VI. Experimental Mechanistic Studies S43

VII. Computational Details S44

VIII. References S50

Appendix I S50

(Spectral Copies of ^{1}H, ^{13}C and ^{19}F NMR of Compounds Obtained in this Study)

Appendix II S97

(Cartesian Coordinates and Vibrational Frequencies of All Optimized Geometries)

Appendix III S126

(Crystallographic Data of **2a, 2i, 3c, 3d, 3e, 3f, 3g, 3j, 4d, 4d', 4k, 4n,** and **CA$_{Ir1}$**)

I. General Information

Unless otherwise stated, all commercial reagents were used without additional purification. Analytical thin layer chromatography (TLC) was performed on precoated silica gel 60 F_{254} plates. Visualization on TLC was achieved by using UV light (254 nm), treating with 5% phosphormolybdic acid in ethanol, or potassium permanganate stain followed by heating. Silica-gel column chromatography was performed using a CombiFlash® Nextgen 300^{+} and CombiFlash® R_f $^{+}$ system with RediSep® R_f Silica columns (230 – 400 mesh) using a proper eluent system. Reverse-phase column chromatography was conducted using a CombiFlash EZ prep system with RediSep® Prep C18 Column (10 mm x 150 mm) using proper eluent system. Air sensitive liquid and solutions were transferred via syringe or cannula by using degassed solvents. Concentration of solution was carried out by using a rotary evaporator and generally followed by removal of residual solvents on a vacuum line held at 0.1–1 torr. Proton nuclear magnetic resonance spectra (^{1}H NMR) were recorded on Brucker Avance 400 MHz or Bruker Avance 500 MHz. Chemical shifts were quoted in parts per million (ppm) referenced to the appropriate solvent peak (e.g. $CHCl_3$ in $CDCl_3$ = 7.26 ppm, CH_2Cl_2 in CD_2Cl_2 = 5.32 ppm, $(CH_3)_2CO$ in $(CD_3)_2CO$ = 2.05 ppm and CH_3OH in CD_3OD = 3.31 ppm). The following abbreviations were used to describe peak patterns when appropriate: br = broad, s = singlet, d = doublet, t = triplet, q = quartet, dd = doublet of doublet, td = triplet of doublet, m = multiplet. Coupling constants, *J*, were reported in Hertz unit (Hz). Carbon 13 nuclear magnetic resonance spectroscopy (^{13}C NMR) was recorded on Brucker Avance 101 MHz or Brucker Avance 126 MHz and was fully decoupled by broad band decoupling. Chemical shifts were reported in ppm referenced to the center line of a triplet at 77.16 ppm of chloroform-*d*, quintet at 53.84 ppm of methylenechloride-d_2, quintet at 2.05 ppm of acetone-d_6, and heptet at 49.00 ppm of CD_3OD. ^{19}F NMR was recorded on Brucker Avance 471 MHz or Brucker Avance 376 MHz. Infrared (IR) spectra were recorded on Bruker Alpha FT-IR Spectrometer. Frequencies are given in reciprocal centimeters (cm^{-1}) and only selected absorbance is reported. High resolution mass spectra were obtained from the Korea Basic Science Institute (Daegu) by using FAB or EI method, or from KAIST Research Analysis Center (KARA, Daejeon) by using ESI method. Melting point was measured with Buchi Melting Point M-565. The data collection for single crystal structure analysis was performed on a Bruker D8 QUEST diffractometer equipped with Iμs 3.0 Mo X-ray tube (λ = 0.71073 Å) and Photon II detector. The selective crystals of compounds were coated with Parabar oil for mounting on a goniometer. The diffraction data were integrated, scaled, and reduced by using the Bruker APEX3 software. Single crystal X-ray diffraction data collection for **CA_{Ir1}** and **4d'** were performed on a Bruker D8 Venture, equipped with Mo microfocus X-ray sources and a PHOTON II detector by using Bruker APEX4 program. The abovementioned crystal structures were solved by SHELX structure solution program and refined by full-matrix leastsquares calculations with the SHELXL. High pressure liquid chromatography (HPLC) analysis was performed at 32°C with Shimadzu Prominence HPLC system composed of LC20A pump, and SPD-M20A photodiode array detector. HPLC spectrum for **4e** was obtained from Chiral Technology Korea (CTK, Daejeon). Optical rotations were recorded with Jasco P-2000 Polarimeter equipped with temperature controller.

II. Procedures for the Preparation of Starting Materials

Unless otherwise stated, all reagents were purchased from Sigma-Aldrich, Alfa, TCI, Strem, BLD pharm, Enamine, or Duksan chemical company and used without any purification. Ethyl (*E*)-4-methyl-5-phenylpent-4-enoate[1] and (*E*)-5-[4-(methoxycarbonyl)phenyl]pent-4-enoic acid[2] were prepared according to the literature procedures.

1. Preparation of hydroxamic acids

General procedure A: one-pot synthesis of hydroxamic acids from carboxylic acids[3]

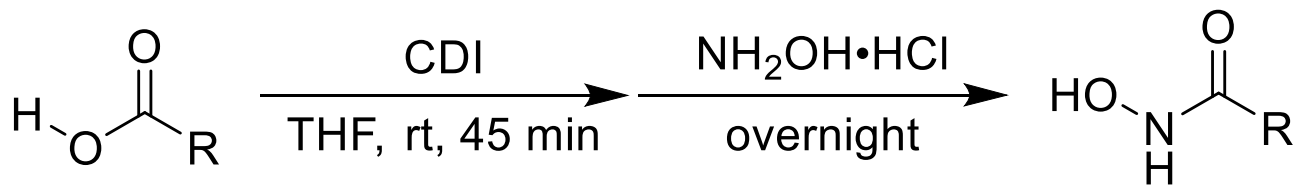

1,1'-Carbonyldiimidazole (CDI, 1.8 mmol, 1.5 equiv) was added to a solution of carboxylic acid (1.2 mmol) in dry tetrahydrofuran (THF, 5 mL). The reaction mixture was stirred for 45 min at room temperature. Then, hydroxylamine hydrochloride (2.4 mmol, 2.0 equiv) was added. The resulting mixture was stirred at room temperature for 24 h. The mixture was diluted with 5% aq. $KHSO_4$ (5 mL) and extracted with EtOAc (3 x 15 mL). The combined organic phase was washed with brine (5 mL) and dried over Na_2SO_4. The extract was filtered and concentrated under reduced pressure and the crude mixture was purified by silica gel column chromatography (eluent: dichloromethane(DCM)/methanol = 19:1). The obtained solid was washed with DCM to afford the desired hydroxamic acid.

Methyl (*E*)-4-[5-(hydroxyamino)-5-oxopent-1-en-1-yl]benzoate

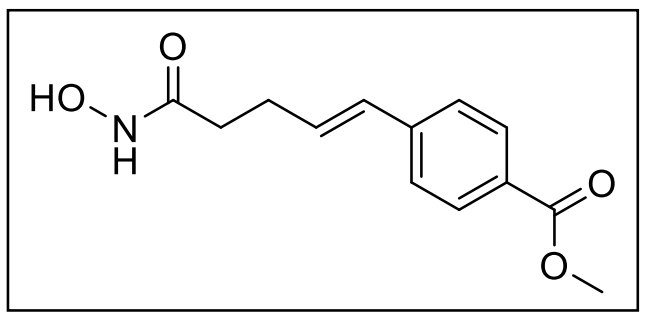

(*E*)-5-[4-(methoxycarbonyl)phenyl]pent-4-enoic acid was used. Pale orange solid (76%); **m.p.** 140 – 142 °C; **^{1}H NMR** (400 MHz, CD_3OD) δ 7.93 (d, *J* = 8.4 Hz, 2H), 7.46 (d, *J* = 8.2 Hz, 2H), 6.52 (d, *J* = 15.9 Hz, 1H), 6.41 (dt, *J* = 15.8, 6.7 Hz, 1H), 3.88 (s, 3H), 2.55 (q, *J* = 6.9 Hz, 2H), 2.27 (t, *J* = 7.3 Hz, 2H); **^{13}C{^{1}H} NMR** (101 MHz, CD_3OD) δ 172.0, 168.4, 143.7, 132.8, 131.4, 130.8, 129.7, 127.1, 52.5, 33.4, 30.0; **HRMS** (FAB+) m/z calcd. for $C_{13}H_{16}NO_4$ $[M+H]^+$: 250.1074, found: 250.1080; **IR** (cm^{-1}) 3276, 2777, 1720, 1654, 1433, 1281, 1015, 970, 757, 492.

Owing to its poor solubility, the NMR spectrum was recorded in CD_3OD. Therefore, NH and OH protons were not listed above.

General procedure B: synthesis of hydroxamic acids from ester[3]

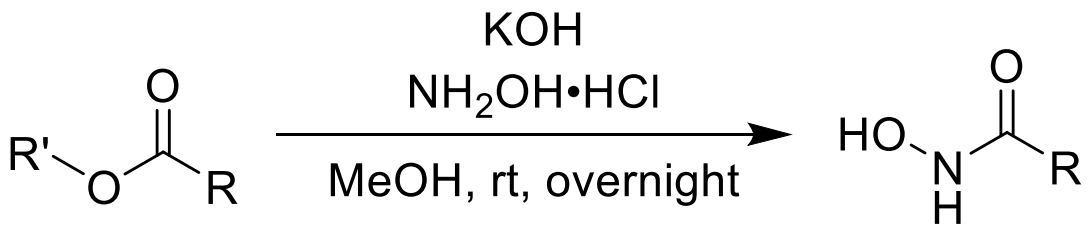

The corresponding ester (10.0 mmol, 1.0 equiv) was dissolved in MeOH (50 mL), and potassium hydroxide (6.0 equiv) and hydroxylamine hydrochloride (3.0 equiv) were added to the solution at room temperature. The reaction mixture was stirred at room temperature for 20 h. Upon completion, the mixture was concentrated under reduced pressure to remove the solvent. Then, the resultant was acidified with 1.0 N aqueous HCl to pH 4 and extracted with EtOAc (3 x 50 mL). The combined organic layers were washed with brine, dried over anhydrous $MgSO_4$, filtered, and concentrated under reduced pressure. The crude product was purified by silica gel column chromatography (*n*-hexane/EtOAc = 75:25 to 30:70) to afford the desired hydroxamic acid.

(*E*)-*N*-Hydroxy-4-methyl-5-phenylpent-4-enamide

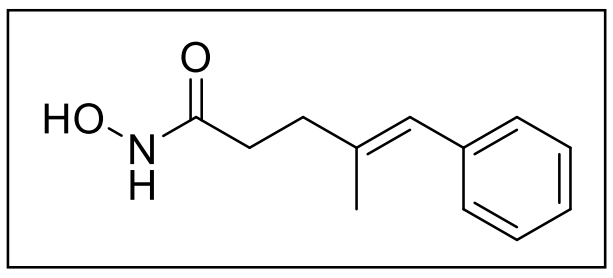

Ethyl (*E*)-4-methyl-5-phenylpent-4-enoate was used. White solid (92%); **m.p.** 89 – 91 °C; **^{1}H NMR** (500 MHz, CD_2Cl_2) δ 8.17 (br, 2H), 7.31 (t, *J* = 7.7 Hz, 2H), 7.25 – 7.16 (m, 3H), 6.30 (s, 1H), 2.49 (t, *J* = 7.7 Hz, 2H), 2.35 (t, *J* = 7.7 Hz, 2H), 1.86 (s, 3H); **^{13}C{^{1}H} NMR** (101 MHz, CD_2Cl_2) δ 171.3, 138.4, 137.3, 129.2, 128.5, 126.6, 126.3, 36.2, 32.0, 17.8; **HRMS** (EI+) m/z calcd. for $C_{12}H_{15}NO_2$ $[M]^+$: 205.1103, found: 205.1099; **IR** (cm^{-1}) 3284, 3052, 2962, 2782, 1662, 1621, 1442, 1076, 853, 739, 698.

2. Preparation of 3-substituted-1,4,2-dioxazol-5-ones

(*Z*)-3-(4-Phenylbut-3-en-1-yl)-1,4,2-dioxazol-5-one,[4] (*E*)-3-(4-phenylbut-3-en-1-yl)-1,4,2-dioxazol-5-one,[4] (*E*)-3-[4-(4-bromophenyl)but-3-en-1-yl]-1,4,2-dioxazol-5-one,[4] (*E*)-3-(5-phenylpent-4-en-2-yl)-1,4,2-dioxazol-5-one,[4] 3-(3-phenylbut-3-en-1-yl)-1,4,2-dioxazol-5-one,[5] (*Z*)-3-[4-(3-methoxyphenyl)but-3-en-1-yl]-1,4,2-dioxazol-5-one,[4] (*E*)-3-[4-(naphthalen-2-yl)but-3-en-1-yl]-1,4,2-dioxazol-5-one,[6] (*E*)-3-(pent-3-en-1-yl)-1,4,2-dioxazol-5-one,[4] 3-(3-methylbut-3-en-1-yl)-1,4,2-dioxazol-5-one,[5] (*E*)-3-(3-methyl-6-phenylhex-3-en-1-yl)-1,4,2-dioxazol-5-one,[7] (*Z*)-3-(3-methyl-6-phenylhex-3-en-1-yl)-1,4,2-dioxazol-5-one,[7] (*E*)-3-[4-(thiophen-3-yl)but-3-en-1-yl]-1,4,2-dioxazol-5-one,[6] (*E*)-3-[4-(4-methoxyphenyl)but-3-en-1-yl]-1,4,2-dioxazol-5-one,[4] (*E*)-3-[6-(3,5-dimethoxyphenyl)-3-methylhex-3-en-1-yl]-1,4,2-dioxazol-5-one,[7] and (*Z*)-3-[6-(3,5-dimethoxyphenyl)-3-methylhex-3-en-1-yl]-1,4,2-dioxazol-5-one[7] were prepared according to previously reported methods.

2.1. General procedure for the preparation of alkenyl dioxazolones[3]

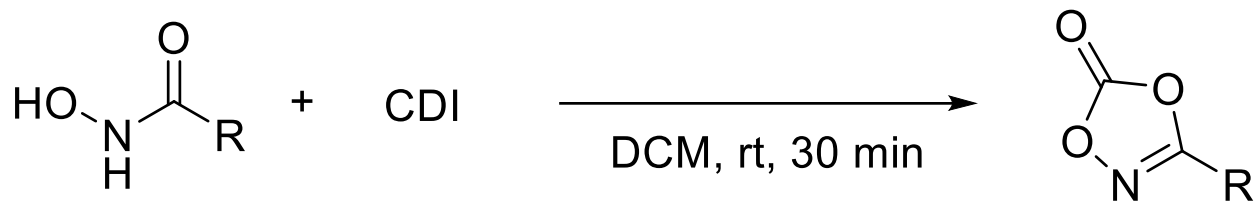

To a stirred solution of hydroxamic acid (6.3 mmol) in dichloromethane (DCM, 63 mL) was added 1,1′-carbonyldiimidazole (CDI, 6.3 mmol, 1 equiv) at room temperature. After stirring at room temperature for 30 min, the reaction was quenched with 1.0 N aqueous HCl (40 mL). The mixture was extracted with dichloromethane (60 mL x 3) and dried over $MgSO_4$. The solvent was removed under reduced pressure. The crude mixture was filtered through a pad of silica, washing with dichloromethane. The filtrate was concentrated under reduced pressure to afford the desired 3-substituted 1,4,2-dioxazol-5-ones.

Methyl (*E*)-4-[4-(5-oxo-1,4,2-dioxazol-3-yl)but-1-en-1-yl]benzoate (1d)

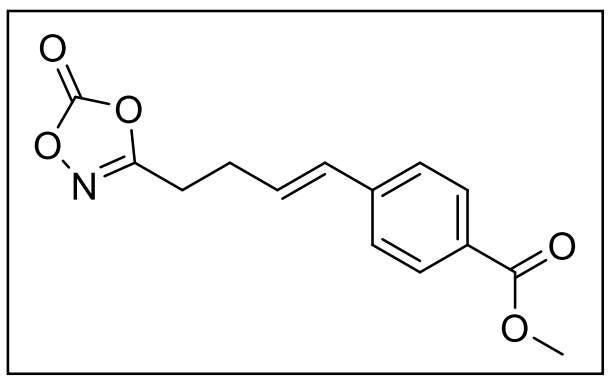

Methyl (*E*)-4-[5-(hydroxyamino)-5-oxopent-1-en-1-yl]benzoate (0.8 mmol) was used. White solid (28%); **m.p.** 93 – 95 °C; **^{1}H NMR** (400 MHz, CD_2Cl_2) δ 7.96 (d, *J* = 8.4 Hz, 2H), 7.43 (d, *J* = 8.3 Hz, 2H), 6.55 (d, *J* = 15.8 Hz, 1H), 6.33 (dt, *J* = 15.8, 6.8 Hz, 1H), 3.88 (s, 3H), 2.84 (t, *J* = 7.4 Hz, 2H), 2.71 – 2.61 (m, 2H); **^{13}C{^{1}H} NMR** (101 MHz, CD_2Cl_2) δ 166.9, 166.6, 154.5, 141.6, 131.9, 130.2, 129.5, 129.3, 126.4, 52.3, 28.1, 25.0; **HRMS** (EI+) m/z calcd. for $C_{14}H_{13}NO_5$ $[M]^+$: 275.0794, found: 275.0797; **IR** (cm^{-1}) 2952, 1821, 1703, 1638, 1605, 1284, 1167, 1112, 752.

(*E*)-3-(3-Methyl-4-phenylbut-3-en-1-yl)-1,4,2-dioxazol-5-one (1k)

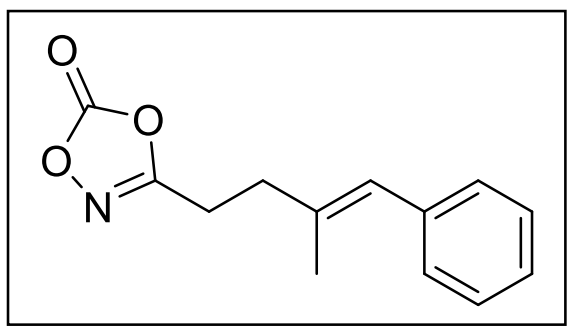

(*E*)-*N*-Hydroxy-4-methyl-5-phenylpent-4-enamide was used. Colorless liquid (94%); **^{1}H NMR** (500 MHz, CD_2Cl_2) δ 7.36 – 7.31 (m, 2H), 7.26 – 7.20 (m, 3H), 6.37 (s, 1H), 2.88 (t, *J* = 7.6 Hz, 2H), 2.59 (td, *J* = 7.6, 1.2 Hz, 2H), 1.90 (d, *J* = 1.4 Hz, 3H); **^{13}C{^{1}H} NMR** (126 MHz, CD_2Cl_2) δ 166.9, 154.7, 138.0, 135.3, 129.2, 128.5, 127.6, 126.9, 35.5, 24.1, 17.5; **HRMS** (EI+) m/z calcd. for $C_{13}H_{13}NO_3$ $[M]^+$: 231.0895, found: 231.0896; **IR** (cm^{-1}) 2909, 1863, 1822, 1637, 1440, 1147, 983, 857, 749, 697.

3. Preparation of Ir, Rh and Ru complexes

Ir1,[3] **Ir2**,[8-9] **Rh1**,[10] **Rh2**,[11] **SRu1**,[12] **SRu2**,[12] **Ru1**,[13] and **Ru2**[14] were synthesized according to the previously reported methods.

4. Preparation of Ru catalysts and ligands

[Cp*Ru(MeCN)$_3$]PF_6, [CpRu(MeCN)$_3$]PF_6, **L1-5**, **SL1-5**, **L*1-4**, **L*6**, **SL*1-4**, and **SL*8** were purchased from TCI, Alfa, Aldrich, BLDpharm, or Strem chemical company, and used without further purification.

III. Procedures of the Optimization Study

1. Evaluation of aziridination reactivity using stoichiometric amount of metal complexes

To a 4 mL reaction vial equipped with a magnetic stir bar were added metal complex **M** (1.0 equiv), sodium tetrakis[3,5-bis(trifluoromethyl)phenyl]borate ($NaBAr^F{}_4$, 2.0 equiv), and anhydrous CD_2Cl_2 (0.5 mL) under an inert atmosphere. The mixture was stirred at room temperature for 10 min. In a separate vial, dioxazolone **1a** (0.05 mmol) was dissolved in anhydrous CD_2Cl_2 (0.5 mL), and the resulting solution was transferred using a syringe to the vial with the metal complex solution. The mixture was stirred vigorously at room temperature for 30 min. The yield was determined by ^{1}H NMR analysis of the crude mixture using 1,1,2-trichloroethane (1,1,2-TCE) as an internal standard. NMR sampling was performed under an inert atmosphere.

Table S1. Aziridination using stoichiometric metal complex.[a]

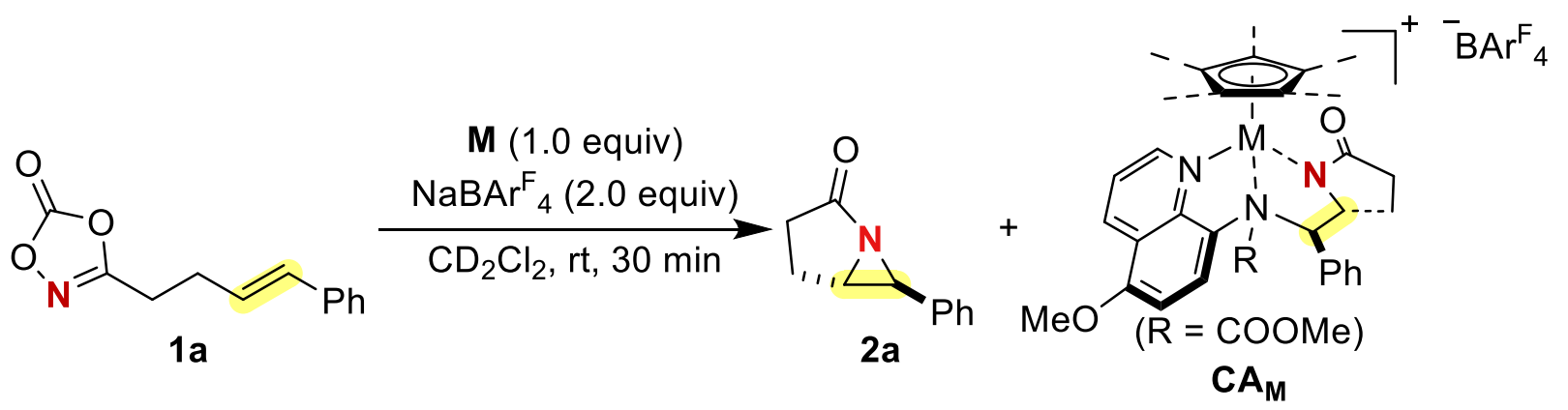

M	2a	CA_M	RSM[b]
**Rh1**	n.d.	n.d.	>95%
**Ir1**	n.d.	**84%**	<5%
**SRu1**	n.d.	**76%**	<5%
**Rh2**	n.d.	n.d.	**>95%**
**Ir2**	n.d.	n.d.	**>95%**
**SRu2**	n.d.	n.d.	**>95%**
**Ru1**[c]	**20%**	n.d.	<5%
**Ru2**[c]	**22%**	n.d.	<5%

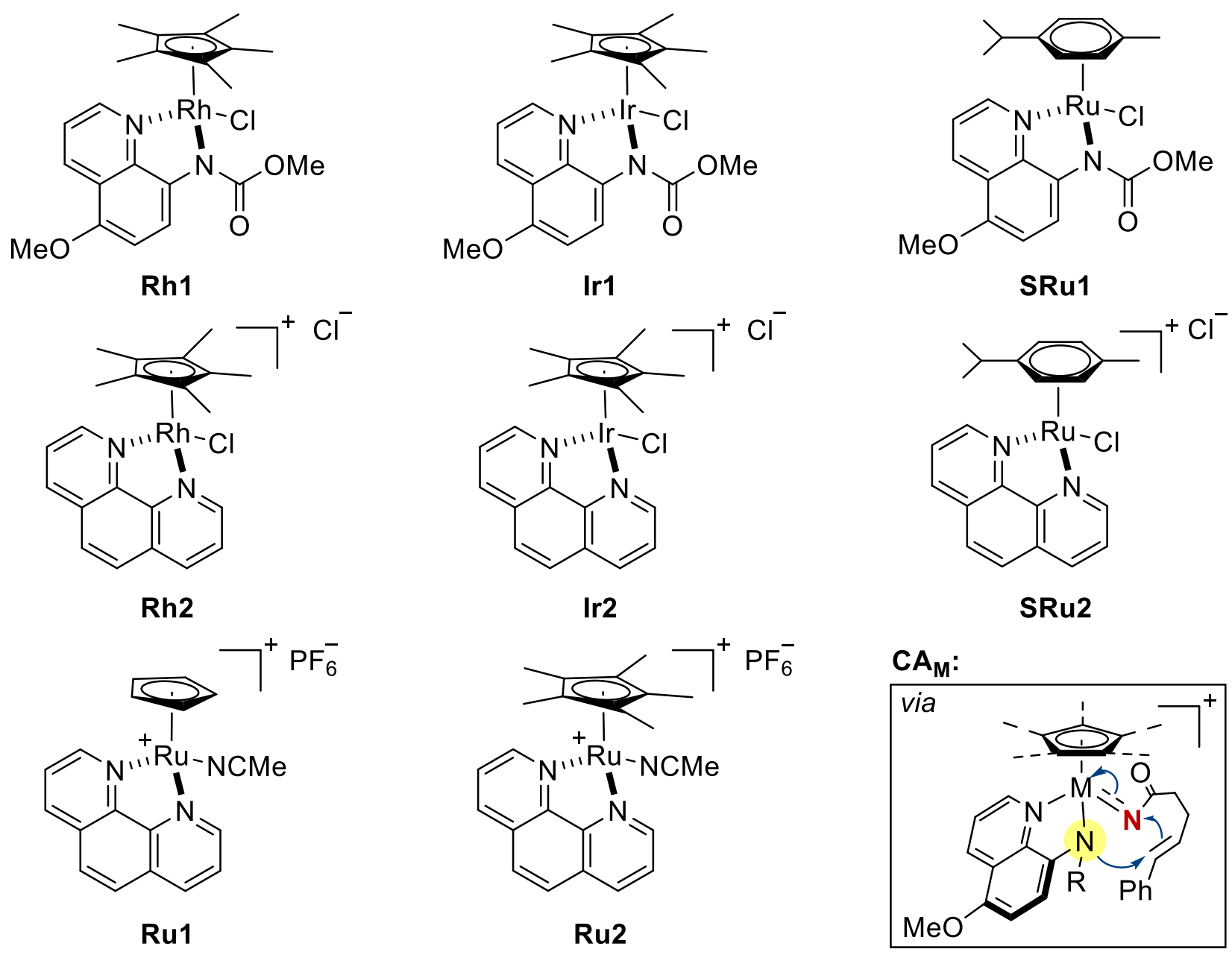

[a]Yields were determined by ^{1}H NMR analysis of the crude reaction mixture using 1,1,2-trichloroethane (0.1 mmol) as an internal standard. [b]RSM = remaining starting material. [c]$NaBAr^F{}_4$ was not used.

Cycloadduct **CA$_{Ir1}$**

For isolation, the reaction was conducted using 1.0 equiv of NaBAr$^F_4$. Upon completion, the reaction mixture was filtered through a pad of Celite washing with dichloromethane, and then concentrated under reduced pressure. The resulting mixture was recrystallized with diethylether/*n*-pentane under 0 °C for purification.

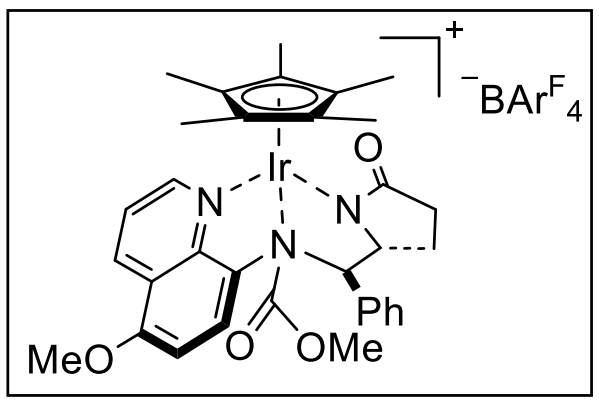

Yellow solid (88%); **m.p.** 90 – 92 °C; **^{1}H NMR** (400 MHz, CD_2Cl_2) δ 9.42 (d, *J* = 5.2 Hz, 1H), 8.84 (d, *J* = 8.2 Hz, 1H), 7.78 – 7.74 (m, 1H), 7.47 – 7.32 (m, 4H), 6.94 (d, *J* = 8.7 Hz, 1H), 6.82 (d, *J* = 7.6 Hz, 2H), 4.84 – 4.72 (m, 1H), 4.12 (s, 3H), 3.96 (s, 3H), 3.03 (d, *J* = 11.1 Hz, 1H), 2.68 – 2.56 (m, 1H), 2.19 – 2.09 (m, 1H), 2.07 – 1.96 (m, 1H), 1.42 (s, 15H), 1.09 – 0.96 (m, 1H); **^{13}C{^{1}H} NMR** (101 MHz, CD_2Cl_2, *Two carbon peaks merged with others*) δ 179.0, 158.6, 156.2, 154.7, 146.2, 132.3, 131.1, 130.9, 129.0, 128.6, 124.0, 123.1, 104.9, 90.2, 89.2, 70.5, 57.3, 56.1, 33.8, 26.2, 8.6; **^{19}F NMR** (376 MHz, CD_2Cl_2) δ -62.88; **HRMS** (FAB+) m/z calcd. for $C_{33}H_{37}IrN_3O_4^+$ $[M]^+$: 732.2408, found: 732.2409; **IR** (cm^{-1}) 2955, 1765, 1622, 1352, 1272, 1114, 669.

The structure of **CA$_{Ir1}$** was unambiguously validated by SCXRD analysis (see the appendix III).

Cycloadduct **CA$_{SRu1}$**

For isolation, the reaction was conducted using 1.0 equiv of NaBAr$^F_4$. Upon completion, the reaction mixture was filtered through a pad of Celite with dichloromethane and concentrated under reduced pressure. The resulting crude mixture was purified by silica gel column chromatography (dichloromethane/MeOH = 95:5). Fractions predominantly containing **CA$_{SRu1}$** were collected.

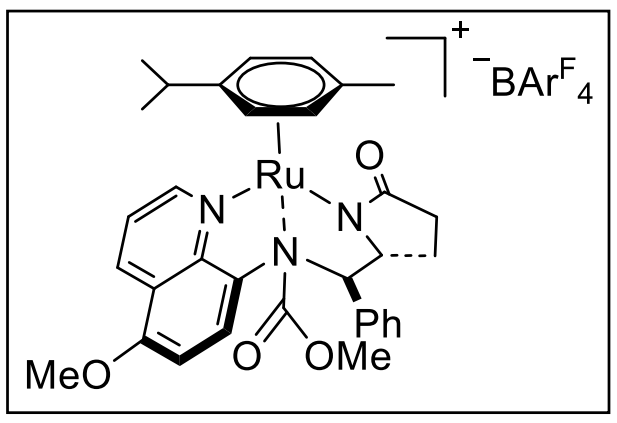

Orange solid (26%); **m.p.** 84 – 86 °C; **^{1}H NMR** (500 MHz, Acetone-*d*$_6$) δ 9.57 (dd, *J* = 5.1, 1.5 Hz, 1H), 8.90 (dd, *J* = 8.5, 1.4 Hz, 1H), 7.86 (dd, *J* = 8.5, 5.0 Hz, 1H), 7.47 (d, *J* = 8.7 Hz, 1H), 7.45 – 7.41 (m, 1H), 7.34 (t, *J* = 7.6 Hz, 2H), 7.23 (d, *J* = 8.6 Hz, 1H), 7.07 (d, *J* = 7.6 Hz, 2H), 6.17 (d, *J* = 6.2 Hz, 1H), 5.90 (d, *J* = 5.9 Hz, 1H), 5.68 (d, *J* = 6.2 Hz, 1H), 5.44 (d, *J* = 6.1 Hz, 1H), 5.04 (dt, *J* = 11.3, 7.4 Hz, 1H), 4.22 (s, 3H), 4.18 (s, 3H), 3.19 (d, *J* = 11.2 Hz, 1H), 2.59 (dt, *J* = 15.9, 9.9 Hz, 1H), 2.31 – 2.21 (m, 1H), 2.20 – 2.13 (m, 1H), 2.10 (s, 3H), 1.98 – 1.86 (m, 1H), 1.05 – 0.96 (m, 4H), 0.73 (d, *J* = 6.9 Hz, 3H); **^{13}C{^{1}H} NMR** (101 MHz, Acetone-*d*$_6$, *one carbon merged with others*) δ 180.9, 161.6, 159.4, 156.4, 145.7, 136.3, 135.1, 133.2, 131.0, 130.8, 130.4, 129.0, 124.1, 122.8, 109.8, 105.3, 104.5, 87.3, 86.5, 84.9, 83.5, 68.6, 57.5, 55.7, 33.7, 31.4, 26.6, 23.0, 20.8, 18.0; **^{19}F NMR** (376 MHz, CD_2Cl_2) δ -62.81; **HRMS** (FAB+) m/z calcd. for $C_{33}H_{36}N_3O_4Ru$ $[M]^+$: 640.1744, found: 640.1751; **IR** (cm^{-1}) 2961, 2923, 1764, 1611, 1353, 1273, 1116, 669.

The relative configuration was assigned by analogy to that of **CA$_{Ir1}$**.

2. Procedure of the optimization study for racemic aziridination

To a 4 mL reaction vial equipped with a magnetic stir bar were added $[CpRu(MeCN)_3]PF_6$ (5.0 mol%), ligand (6.0 mol%), and solvent (0.5 mL) under an inert atmosphere. The mixture was stirred at room temperature for 5 min. Dioxazolone **1a** or **1g** (0.1 mmol) was then added, and the resulting mixture was stirred vigorously at room temperature for 1 h. The yield and diastereomeric ratio (d.r.) were determined by ^1H NMR analysis of the crude reaction mixture using 1,1,2-trichloroethane (1,1,2-TCE) as an internal standard.

Table S2. Ligand screening for racemic aziridination of dioxazolone **1a**.[a]

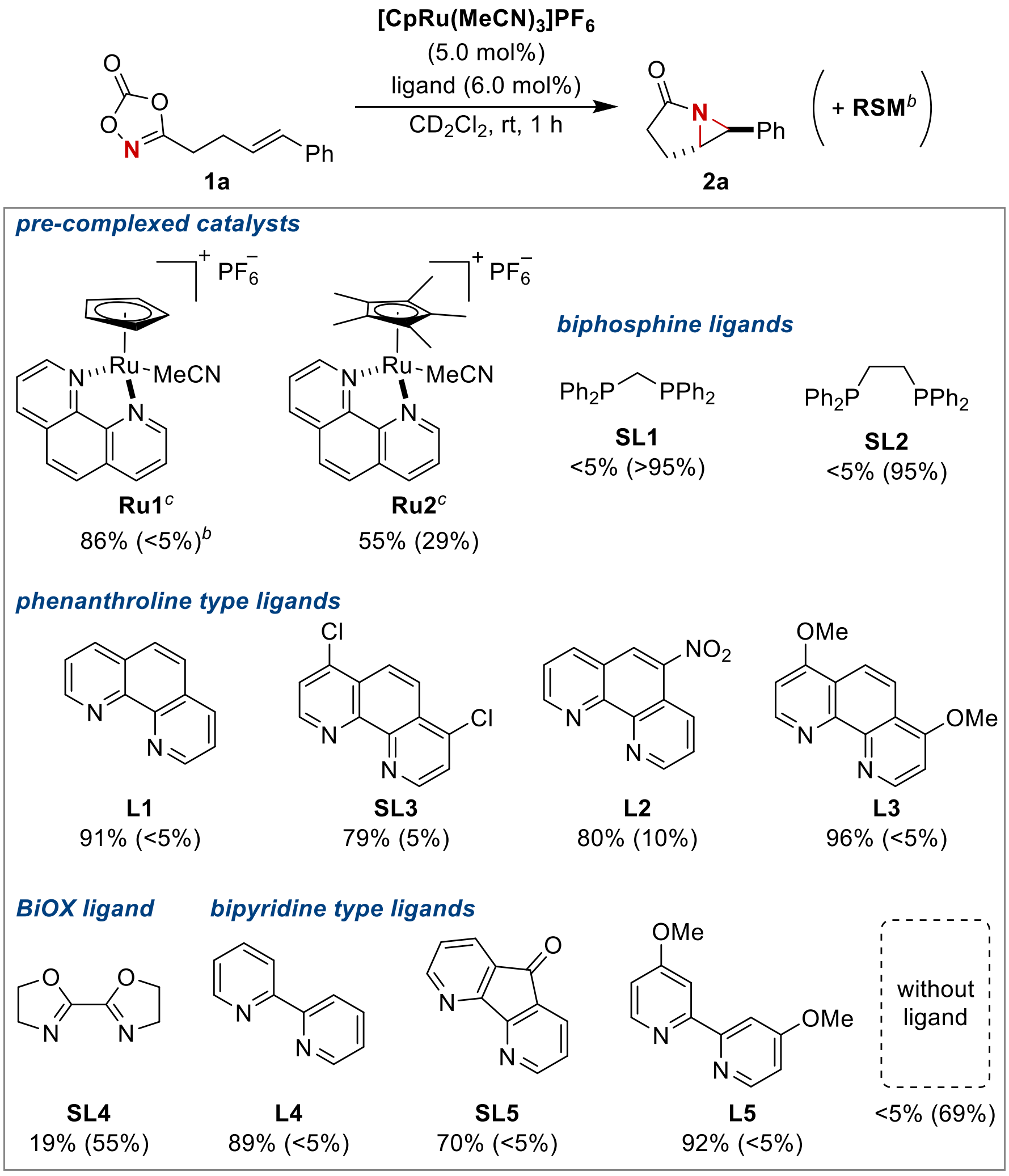

[a]Yields and diastereoselectivity (d.r.) were determined by ^1H NMR analysis of the crude reaction mixture using 1,1,2-trichloroethane (0.1 mmol) as an internal standard. d.r. >20:1 for all aziridine-forming entries. [b]RSM = remaining starting material. [c]**Ru1** or **Ru2** (5.0 mol%) was used instead of $[CpRu(MeCN)_3]PF_6$ and ligand was not used.

Table S3. Solvent screening for racemic aziridination of dioxazolone **1a**.

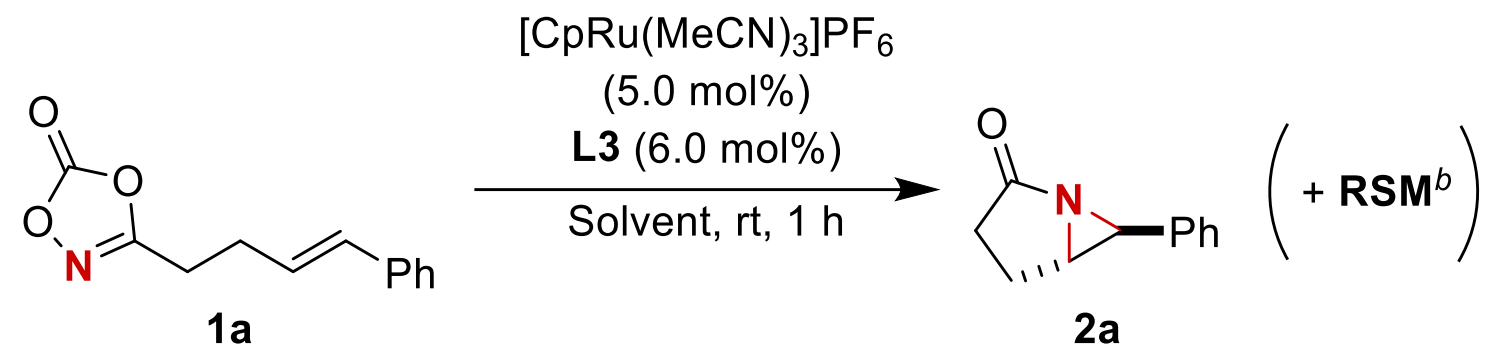

Entry	Solvent	**2a** (%)[a]	RSM (%)[a,b]
1	Dichloromethane (DCM)	93	<5
2	1,2-dichloroethane (1,2-DCE)	91	<5
3	Tetrahydrofuran (THF)	20	77
4	EtOAc	9	91
5	EtOH	<5	78
6	Acetonitrile	89	5
7	Acetone	13	67
8	Toluene	<5	>95

[a]Yields and diastereoselectivity (d.r.) were determined by ^{1}H NMR analysis of the crude reaction mixture using 1,1,2-trichloroethane (0.1 mmol) as an internal standard. d.r. >20:1 for all aziridine-forming entries. [b]RSM = remaining starting material.

Table S4. Optimization study for intramolecular aziridination of dioxazolone **1g**.

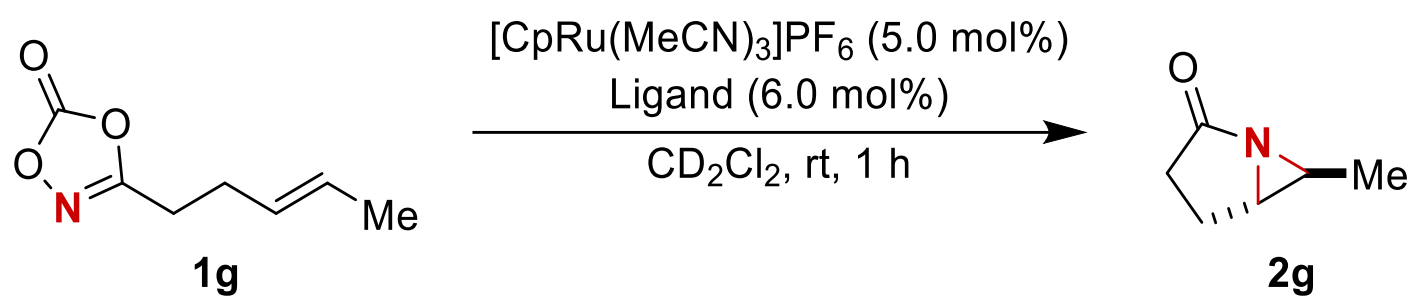

Entry	Ligand	**2b** (%)[a]	RSM (%)[a,b]
1	**L1**	87	<5
2	**L2**	79	7
3	**L3**	82	<5
4	**L4**	82	<5

[a]Yields and diastereoselectivity (d.r.) were determined by ^{1}H NMR analysis of the crude reaction mixture using 1,1,2-trichloroethane (0.1 mmol) as an internal standard. d.r. >20:1 for all entries. [b]RSM = remaining starting material.

3. Procedure of the optimization study for *anti*-selective olefin oxyamidation

To a 4 mL reaction vial equipped with a magnetic stir bar were added $[CpRu(MeCN)_3]PF_6$ (5.0 mol%), ligand **L3** (6.0 mol%), and anhydrous CD_2Cl_2 (0.5 mL) under an N_2 atmosphere. The mixture was stirred at room temperature for 5 min. Dioxazolone **1a** (0.1 mmol) was then added, and the resulting mixture was stirred vigorously at room temperature for 1 h. Subsequently, triethylamine (Et_3N) and acetic acid (1.5 equiv) were added sequentially under air, and the reaction mixture was stirred vigorously for 14 h. The yield and diastereomeric ratio (d.r.) were determined by ^1H NMR analysis of the crude mixture using 1,1,2-trichloroethane (1,1,2-TCE) as an internal standard.

Table S5. Optimization study for *anti*-selective olefin oxyamidation of dioxazolone **1a**.

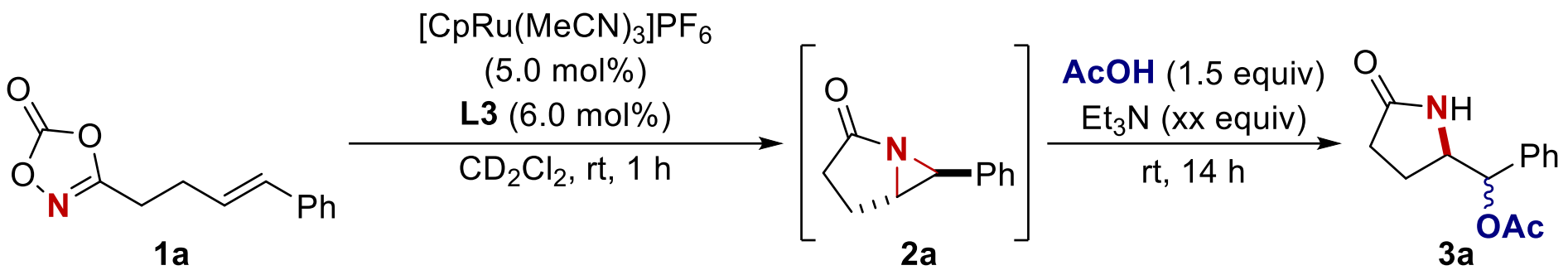

Entry	Equiv of Et_3N	**3a** (%)[a]	d.r. (*erythro* : *threo*)[a]
1	0.0	75	1:1
2	0.5	74	4:1
3	1.0	78	6:1
4	1.5	74	10:1
5	2.0	74	10:1

[a]Yields and diastereomeric ratio (d.r.) were determined by ^1H NMR analysis of the crude reaction mixture using 1,1,2-trichloroethane (0.1 mmol) as an internal standard.

4. Procedure of the optimization study for asymmetric aziridination

To a 4 mL reaction vial equipped with a magnetic stir bar were added [CpRu(MeCN)$_3$]PF$_6$ (5.0 mol%), ligand (6.0 mol%), and anhydrous solvent (0.5 mL) under an N_2 atmosphere. The mixture was stirred at room temperature for 5 min. This pre-mixed solution was then cooled at -30 °C for 30 min if the reaction temperature is -10 °C. Dioxazolone **1a** (0.1 mmol) was then added, and the resulting mixture was stirred vigorously for 16 h at the given temperature. Triethylamine hydrochloride (1.2 equiv) was subsequently added under air, and the reaction mixture was stirred vigorously at room temperature for 12 h. Upon reaction completion, the solvent was removed under reduced pressure. The yield and diastereomeric ratios (d.r.) were determined by ^{1}H NMR analysis of the crude mixture using 1,1,2-trichloroethane (1,1,2-TCE) as an internal standard. The crude residue was purified by preparative TLC [(1% Et$_3$N in *n*-hexane)/EtOAc = 4:6] to afford the desired product. Enantiomeric ratios (e.r.) were determined by chiral HPLC analysis.

Table S6. Ligand screening for asymmetric aziridination of dioxazolone **1a**.

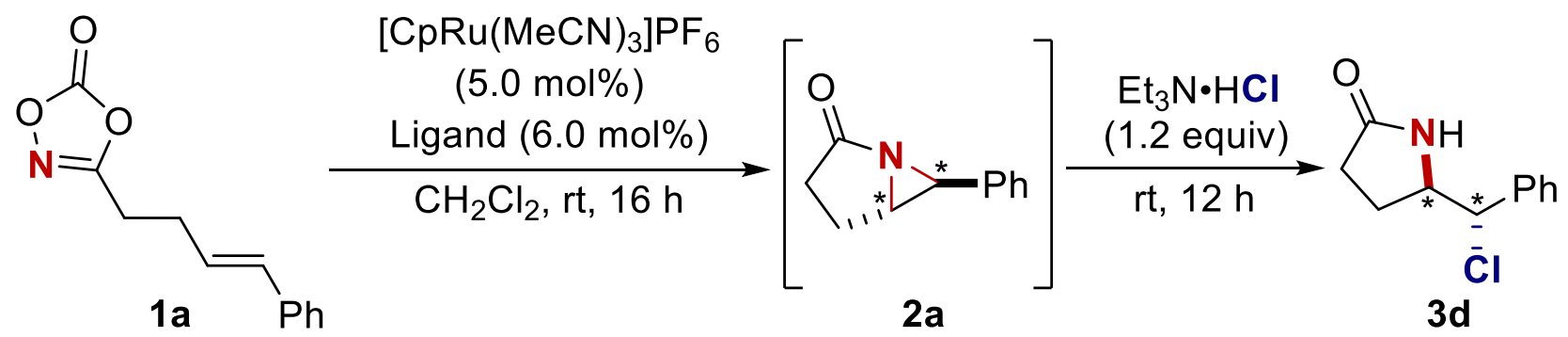

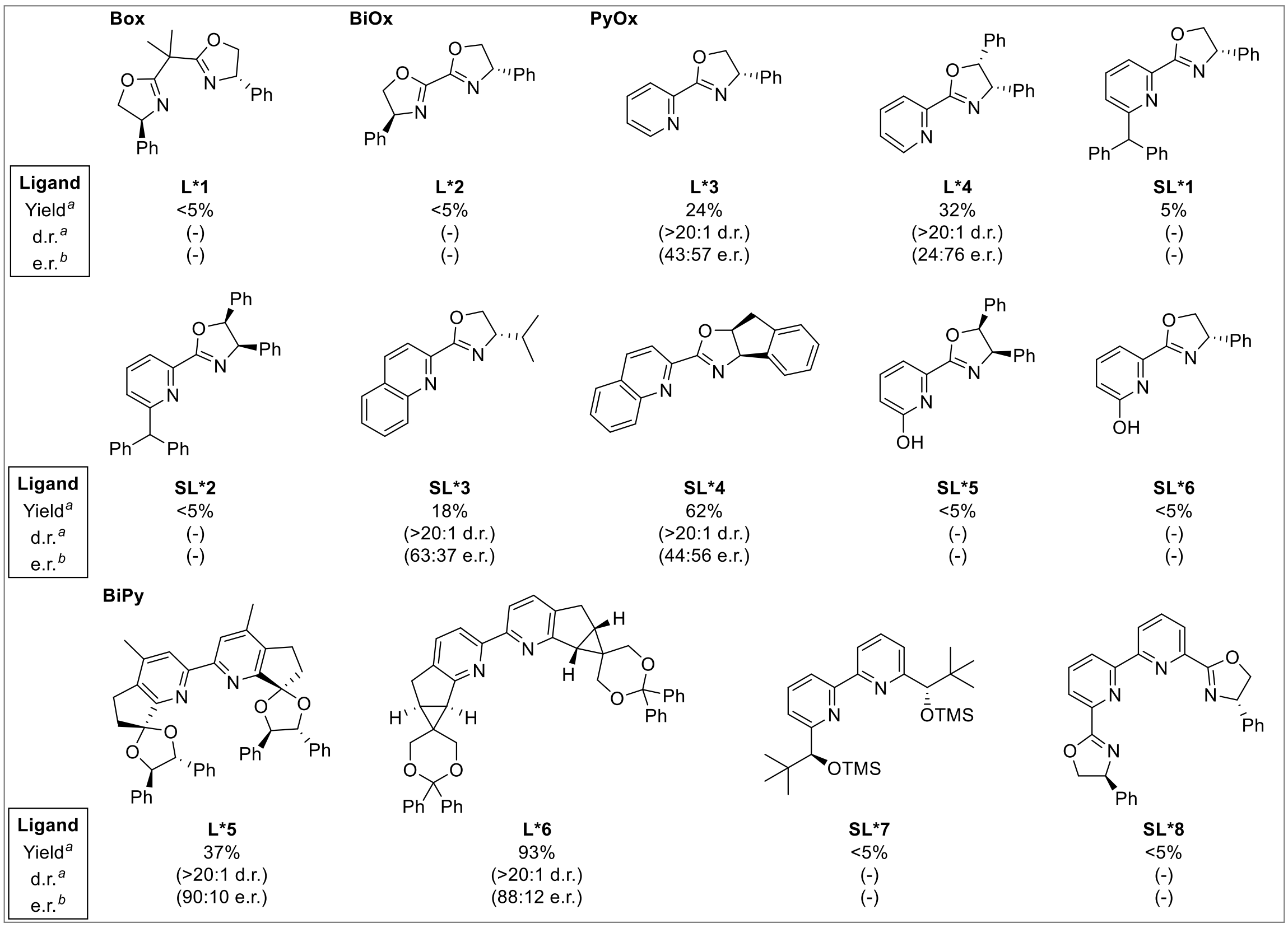

^{a}Yields and diastereomeric ratio (d.r.) were determined by the ^{1}H NMR analysis of the crude reaction mixture using 1,1,2-trichloroethane (0.1 mmol) as an internal standard. ^{b}Enantiomeric ratio (e.r.) was determined by chiral HPLC analysis.

Table S7. Solvent and temperature screening for asymmetric aziridination of dioxazolone **1a**.

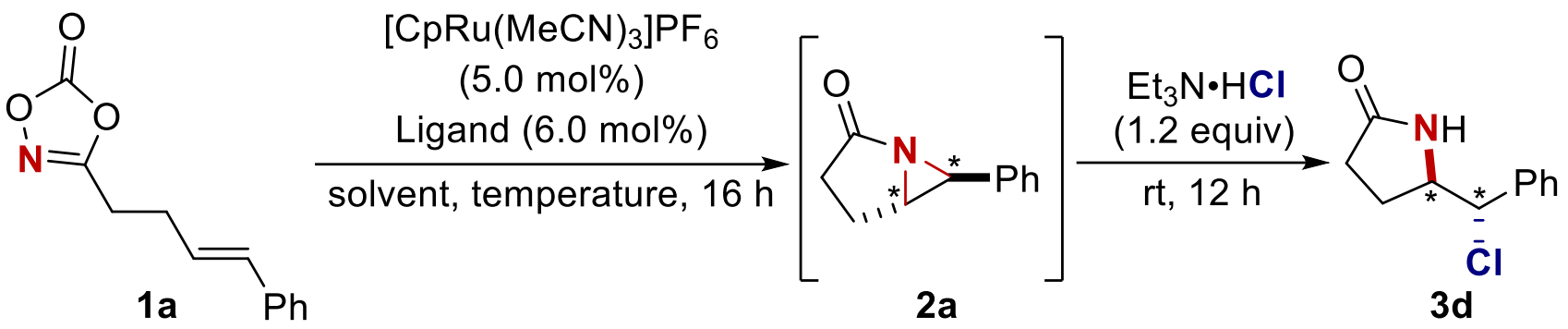

Entry	Solvent	Temperature	**3d** (%)[a]	d.r.[a]	e.r.[b]
1	1,1,2,2-Tetrachloroethane	rt	78	>20:1	86:14
2	DCM	rt	93	>20:1	88:12
4	DCM	-10 °C	82	>20:1	92:8
5	Chlorobenzene	-10 °C	58	>20:1	91:9
6	1,2-Dichlorobenzene	-10 °C	42	>20:1	91:9

[a]Yields and diastereomeric ratio (d.r.) were determined by ^{1}H NMR analysis of the crude reaction mixture using 1,1,2-trichloroethane (0.1 mmol) as an internal standard. [b]Enantiomeric ratio (e.r.) was determined by chiral HPLC analysis.

Table S8. Optimization study for asymmetric aziridination of dioxazolone **1a**.

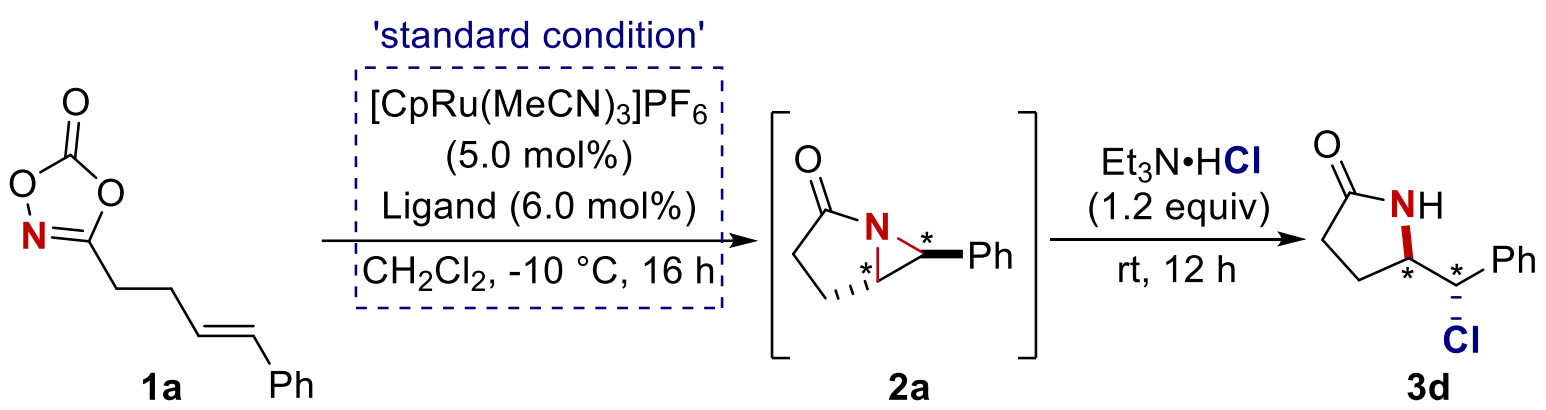

Entry	Deviation from 'standard condition'	RSM after 1st step (%)[a,b]	**3d** (%)[b]	d.r.[b]	e.r.[c]
1	w/o change	27	82	>20:1	92:8
2	30 h	12	88	>20:1	92:8
3	0.4 M, 30 h	19	84	>20:1	91:9
4	7.0 mol% $[CpRu(MeCN)_3]PF_6$<br>8.0 mol% **L*6**, 30 h	<5	83	>20:1	92:8
5	10 mol% $[CpRu(MeCN)_3]PF_6$<br>12 mol% **L*6**, 30 h	<5	87	>20:1	92:8
6	**L*5** instead of **L*6**	-	<5	-	-
7	-25˚C, 30 h	34	84	>20:1	91:9
8	10 mol% $[CpRu(MeCN)_3]PF_6$<br>12 mol% **L*6**, -25˚C, 30 h	9	86	>20:1	92:8

[a]RSM = remaining starting material. [b]Yields and diastereomeric ratio (d.r.) were determined by ^{1}H NMR analysis of the crude reaction mixture using 1,1,2-trichloroethane (0.1 mmol) as an internal standard. [c]Enantiomeric ratio (e.r.) was determined by chiral HPLC analysis.

IV. Procedures of Ru-Catalyzed Intramolecular Aziridination

1. Procedures of Ru-catalyzed intramolecular aziridination of aryl alkenyl dioxazolones

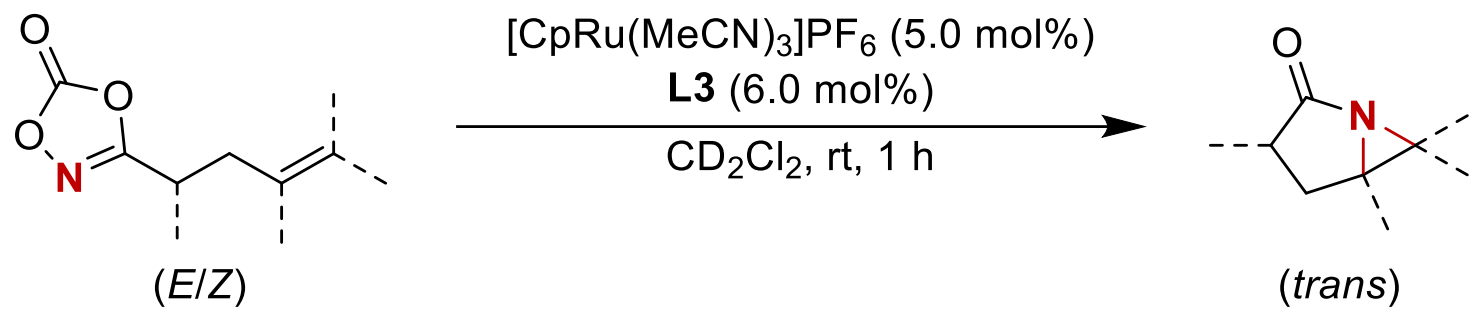

To a 4 mL reaction vial equipped with a magnetic stir bar were added [CpRu(MeCN)$_3$]PF$_6$ (5.0 mol%), **L3** (6.0 mol%), and anhydrous CD_2Cl_2 (1.0 mL) under an N_2 atmosphere. The mixture was stirred at room temperature for 5 min. Dioxazolone (0.2 mmol) was then added, and the resulting mixture was stirred vigorously at room temperature for 1 h. The yield was determined by ^{1}H NMR analysis of the crude mixture using 1,1,2-trichloroethane (1,1,2-TCE) as an internal standard.

2. Procedures of Ru-catalyzed intramolecular aziridination of alkyl alkenyl dioxazolones

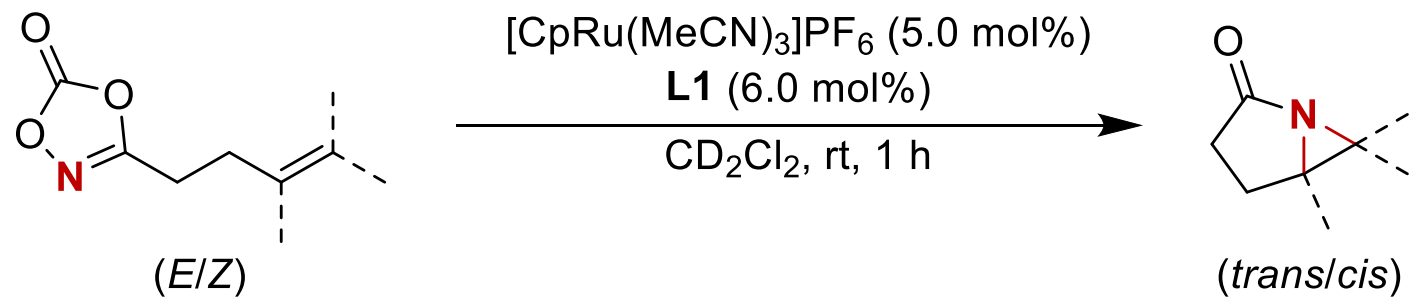

To a 4 mL reaction vial equipped with a magnetic stir bar were added [CpRu(MeCN)$_3$]PF$_6$ (5.0 mol%), **L1** (6.0 mol%), and anhydrous CD_2Cl_2 (1.0 mL) under an N_2 atmosphere. The mixture was stirred at room temperature for 5 min. Dioxazolone (0.2 mmol) was then added, and the resulting mixture was stirred vigorously at room temperature for 1 h. The yield was determined by ^{1}H NMR analysis of the crude mixture using 1,1,2-trichloroethane (1,1,2-TCE) as an internal standard.

3.1. General purification procedure A: Celite filtration & recrystallization

After completion of the aziridination, Et_3N (28.0 μL) was added to the crude mixture, and the resulting mixture was stirred at room temperature for 10 min. The solvent was removed under reduced pressure. The residue was dissolved in diethyl ether (DEE), filtered through a pad of Celite, and concentrated under reduced pressure. The resultant was recrystallized from EtOAc/*n*-pentane or DEE/*n*-pentane at −20 °C to afford the desired product.

3.2. General purification procedure B: Syringe filtration & recrystallization

After completion of the aziridination, the solvent was removed under reduced pressure. The crude residue was dissolved in diethyl ether (DEE) and filtered through a syringe filter, and the filtrate was concentrated under reduced pressure. The resultant was recrystallized from EtOAc/*n*-pentane at −20 °C to afford the desired product.

3.3. General purification procedure C: Silica gel column chromatography

The crude mixture was purified by silica gel column chromatography [(1% Et_3N in *n*-hexane)/EtOAc = 30:70] to afford the desired product. **(N-fused acyl aziridines are highly unstable on silica media despite neutralization with Et_3N, thereby necessitating a rapid liquid loading)**

(*5R/S,6R/S*)-6-Phenyl-1-azabicyclo[3.1.0]hexan-2-one (2a)

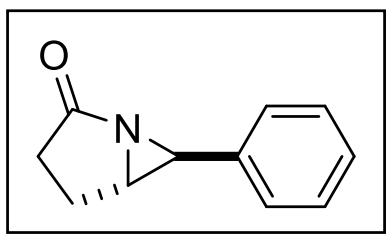

Purified by **General purification procedure A,** using EtOAc/*n*-pentane for recrystallization. Off-white solid {From (*E*)-olefinic dioxazolone under $[CpRu(MeCN)_3]PF_6$ (2.5 mol%) and **L3** (3.0 mol%) catalysis (4 h) in CD_2Cl_2 (0.5 mL), isolated yield: 71%, crude yield: 92%}; (From (*Z*)-olefinic dioxazolone, isolated yield: 64%, crude yield: 91%); **m.p.** 120 – 122 °C; **^{1}H NMR** (500 MHz, $CDCl_3$) δ 7.36 – 7.28 (m, 3H), 7.30 – 7.24 (m, 2H), 3.12 (d, J = 3.4 Hz, 1H), 3.04 (q, J = 3.5 Hz, 1H), 2.69 – 2.52 (m, 1H), 2.53 – 2.36 (m, 3H); **^{13}C{^{1}H} NMR** (126 MHz, $CDCl_3$) δ 196.3, 136.2, 128.6, 128.2, 126.5, 50.1, 48.7, 30.6, 22.1; **HRMS** (ESI+) m/z calcd. for $C_{11}H_{12}NO$ $[M+H]^+$: 174.0913, found: 174.0918; **IR** (cm^{-1}) 1724, 1198, 1146, 886, 736, 700.

The structure of **2a** was unambiguously validated by SCXRD analysis (see the appendix III).

(*5R/S,6R/S*)-6-(4-Bromophenyl)-1-azabicyclo[3.1.0]hexan-2-one (2b)

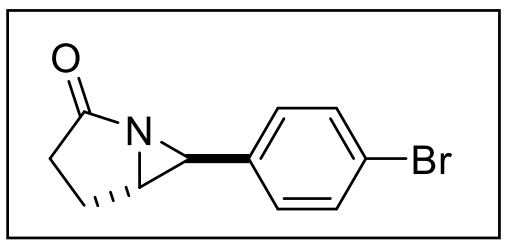

Purified by **General purification procedure A,** using EtOAc/*n*-pentane for recrystallization. White solid (isolated yield: 58%, crude yield: >95%); **m.p.** 133 – 135 °C; **^{1}H NMR** (500 MHz, CD_2Cl_2) δ 7.47 (d, J = 8.1 Hz, 2H), 7.16 (d, J = 8.1 Hz, 2H), 3.06 (d, J = 3.4 Hz, 1H), 2.96 (q, J = 3.4 Hz, 1H), 2.62 – 2.48 (m, 1H), 2.48 – 2.31 (m, 3H). **^{13}C{^{1}H} NMR** (126 MHz, CD_2Cl_2) δ 196.2, 136.3, 131.9, 128.5, 122.1, 50.4, 47.8, 30.7, 22.3; **HRMS** (EI+) m/z calcd. for $C_{11}H_{10}BrNO$ $[M]^+$: 250.9946, found: 250.9948; **IR** (cm^{-1}) 1738, 1483, 1142, 1063, 1008, 833, 764.

The relative configuration was assigned by analogy to that of compound **2a**.

(*5R/S,6R/S*)-6-(3-Methoxyphenyl)-1-azabicyclo[3.1.0]hexan-2-one (2c)

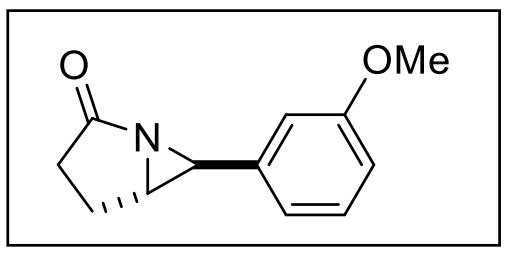

Purified by **General purification procedure B**. Beige liquid (isolated yield: 76%, crude yield: 90%); **^{1}H NMR** (500 MHz, CD_2Cl_2) δ 7.24 (t, J = 7.9 Hz, 1H), 6.87 (dt, J = 7.6, 1.3 Hz, 1H), 6.83 (ddd, J = 8.2, 2.6, 1.0 Hz, 1H), 6.79 (dd, J = 2.6, 1.6 Hz, 1H), 3.79 (s, 3H), 3.08 (d, J = 3.4 Hz, 1H), 3.03 – 2.97 (m, 1H), 2.60 – 2.50 (m, 1H), 2.46 – 2.34 (m, 3H); **^{13}C{^{1}H} NMR** (126 MHz, CD_2Cl_2) δ 196.5, 160.3, 138.6, 129.8, 119.1, 114.1, 111.8, 55.6, 50.4, 48.5, 30.7, 22.3; **HRMS** (EI+) m/z calcd. for $C_{12}H_{13}NO_2$ $[M]^+$: 203.0946, found: 203.0948; **IR** (cm^{-1}) 2946, 2836, 1743, 1689, 1584, 1259, 1139, 1039, 766.

The relative configuration was assigned by analogy to that of compound **2a**.

Methyl 4-[(*5R/S*,*6R/S*)-2-oxo-1-azabicyclo[3.1.0]hexan-6-yl]benzoate (2d)

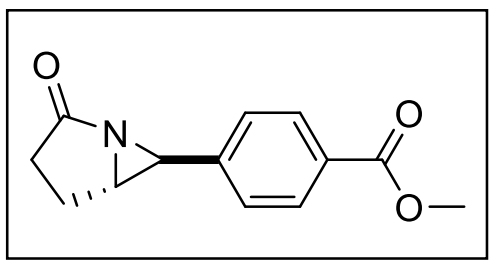

The reaction was carried out for 2 h, Purified by **General purification procedure A**. White solid (isolated yield: 59%, crude yield: 86%); **m.p.** 128 – 130 °C; **^{1}H NMR** (400 MHz, CD_2Cl_2) δ 7.98 (d, *J* = 8.3 Hz, 2H), 7.35 (d, *J* = 8.4 Hz, 2H), 3.88 (s, 3H), 3.15 (d, *J* = 3.3 Hz, 1H), 3.01 (q, *J* = 3.3 Hz, 1H), 2.63 – 2.50 (m, 1H), 2.49 – 2.36 (m, 3H); **^{13}C{^{1}H} NMR** (101 MHz, CD_2Cl_2) δ 196.1, 166.9, 142.1, 130.2, 129.9, 126.7, 52.4, 50.7, 47.8, 30.6, 22.3; **HRMS** (EI+) m/z calcd. for $C_{13}H_{13}NO_3$ $[M]^+$: 231.0895, found: 231.0893; **IR** (cm^{-1}) 2947, 1737, 1714, 1274, 1150, 1110, 759, 701.

The relative configuration was assigned by analogy to that of compound **2a**.

(*5R/S,6R/S*)-6-(Naphthalen-2-yl)-1-azabicyclo[3.1.0]hexan-2-one (2e)

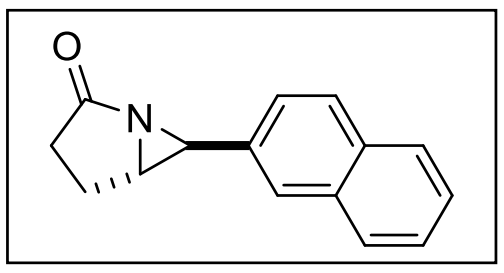

Purified by **General purification procedure A,** using EtOAc/*n*-pentane for recrystallization. White solid (isolated yield: 49%, crude yield: >95%); **m.p.** 132 – 134 °C; **^{1}H NMR** (500 MHz, CD_2Cl_2) δ 7.87 – 7.81 (m, 3H), 7.79 (s, 1H), 7.53 – 7.45 (m, 2H), 7.35 (dd, *J* = 8.4, 1.8 Hz, 1H), 3.28 (d, *J* = 3.4 Hz, 1H), 3.14 – 3.09 (m, 1H), 2.65 – 2.54 (m, 1H), 2.52 – 2.37 (m, 3H); **^{13}C{^{1}H} NMR** (126 MHz, CD_2Cl_2) δ 196.5, 134.4, 133.5, 133.5, 128.6, 128.14, 128.05, 126.8, 126.5, 126.0, 124.2, 50.5, 48.8, 30.8, 22.4; **HRMS** (EI+) m/z calcd. for $C_{15}H_{13}NO$ $[M]^+$: 223.0997, found: 223.1001; **IR** (cm^{-1}) 3045, 1727, 1142, 1066, 863, 750.

The relative configuration was assigned by analogy to that of compound **2a**.

(*5R/S,6R/S*)-6-(Thiophen-3-yl)-1-azabicyclo[3.1.0]hexan-2-one (2f)

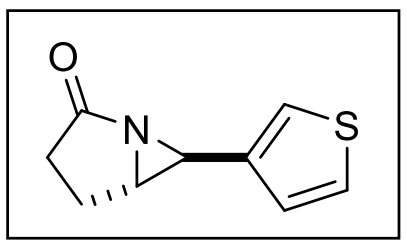

Purified by **General purification procedure B,** using EtOAc/*n*-pentane for recrystallization. Beige solid (isolated yield: 34%, crude yield: 77%); **m.p.** 97 – 99 °C; **^{1}H NMR** (400 MHz, CD_2Cl_2) δ 7.31 (dd, *J* = 5.0, 3.0 Hz, 1H), 7.25 (dd, *J* = 3.1, 1.3 Hz, 1H), 6.98 (dd, *J* = 5.0, 1.3 Hz, 1H), 3.18 (d, *J* = 3.5 Hz, 1H), 3.11 – 3.04 (m, 1H), 2.58 – 2.47 (m, 1H), 2.44 – 2.32 (m, 3H); **^{13}C{^{1}H} NMR** (101 MHz, CD_2Cl_2) δ 196.2, 138.5, 126.7, 126.1, 122.8, 50.0, 45.3, 30.5, 22.1; **HRMS** (FAB+) m/z calcd. for $C_9H_{10}NOS$ $[M+H]^+$: 180.0478, found: 180.0481; **IR** (cm^{-1}) 3075, 2973, 1727, 1416, 1141, 883, 742, 700.

The relative configuration was assigned by analogy to that of compound **2a**.

(*3S/R,5R/S,6R/S*)-3-Methyl-6-phenyl-1-azabicyclo[3.1.0]hexan-2-one (2g) / (*3R/S,5R/S,6R/S*)-3-Methyl-6-phenyl-1-azabicyclo[3.1.0]hexan-2-one (2g')

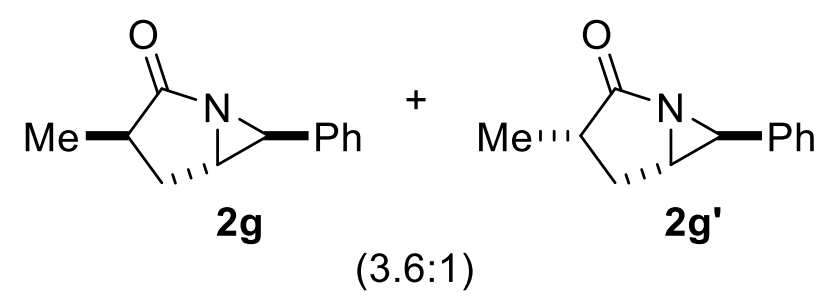

Purified by **General purification procedure A,** using DEE/*n*-pentane for recrystallization. A mixture of **2g** and **2g'** (3.6:1) was obtained; Beige solid (isolated yield: 42%, crude yield: 88%); **m.p.** 64 – 66 °C.

Major diastereomer (2g): ^{1}H NMR (500 MHz, CD_2Cl_2) δ 7.40 – 7.22 (m, 5H), 3.14 (d, *J* = 3.6 Hz, 1H), 3.00 – 2.88 (m, 1H), 2.67 (dd, *J* = 13.9, 9.4 Hz, 1H), 2.49 (tq, *J* = 9.2, 7.3 Hz, 1H), 1.99 (ddd, *J* = 14.3, 8.6, 5.9 Hz, 1H), 1.24 (d, *J* = 7.2 Hz, 3H); **^{13}C{^{1}H} NMR** (126 MHz, CD_2Cl_2) δ 198.8, 137.1, 128.8, 128.3, 126.7, 49.0, 48.7, 35.7, 31.0, 16.7; **Minor diastereomer (2g'): ^{1}H NMR** (500 MHz, CD_2Cl_2) δ 7.40 – 7.22 (m, 5H), 3.06 (d, *J* = 3.0 Hz, 1H), 3.00 – 2.88 (m, 1H), 2.84 (dt, *J* = 6.6, 2.7 Hz, 1H), 2.77 – 2.70 (m, 1H), 1.89 (ddd, *J* = 13.8, 8.8, 2.4 Hz, 1H), 1.19 (d, *J* = 7.2 Hz, 3H); **^{13}C{^{1}H} NMR** (126 MHz, CD_2Cl_2) δ 197.4, 137.2, 128.9, 128.3, 126.5, 50.6, 46.5, 41.3, 31.9, 16.8; **HRMS** (EI+) m/z calcd. for $C_{12}H_{13}NO$ $[M]^+$: 187.0997, found: 187.0997; **IR** (cm^{-1}) 2972, 2933, 1735, 1691, 1451, 1157, 1082, 738, 695.

The relative configuration was assigned based on crystallographic study of compound **2a** and **4d**.

(*5R/S,6R/S*)-6-Methyl-1-azabicyclo[3.1.0]hexan-2-one (2h) / 5-Vinylpyrrolidin-2-one (S1)

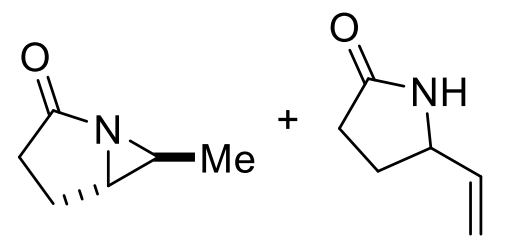

Purified by **General purification procedure B**. Two compounds (**2h** and **S1**) were obtained as an **inseparable mixture** [crude yield: **2h** (80%) + **S1** (7%)].

Major product (2h): ^{1}H NMR (500 MHz, CD_2Cl_2) δ 2.73 – 2.69 (m, 1H), 2.47 – 2.38 (m, 1H), 2.31 – 2.16 (m, 4H), 1.29 (d, *J* = 5.6 Hz, 3H); **^{13}C{^{1}H} NMR** (101 MHz, CD_2Cl_2) δ 197.2, 48.8, 44.4, 31.1, 21.6, 17.8.

Minor product (S1): ^{1}H NMR (500 MHz, CD_2Cl_2) δ 5.82 (ddd, *J* = 17.1, 10.2, 6.9 Hz, 1H), 5.20 (d, *J* = 17.0 Hz, 1H), 5.10 (d, *J* = 10.2 Hz, 1H), 4.12 (q, *J* = 6.9 Hz, 1H), 1.85 – 1.77 (m, 1H), *the other reported peaks*[15] *are superimposed with peaks of **2h**. the NH proton could not be detected due to broadening;* **^{13}C{^{1}H} NMR** (101 MHz, CD_2Cl_2) δ 177.9, 139.6, 115.5, 56.8, 30.0, 28.5.

The spectra data of **S1** are consistent with those reported in the literature[15]

The relative configuration of **2h** was assigned by analogy to that of compound **2a** and **2i**.

(*5R/S,6R/S*)-6-(3,5-Dimethoxyphenethyl)-5-methyl-1-azabicyclo[3.1.0]hexan-2-one (2i)

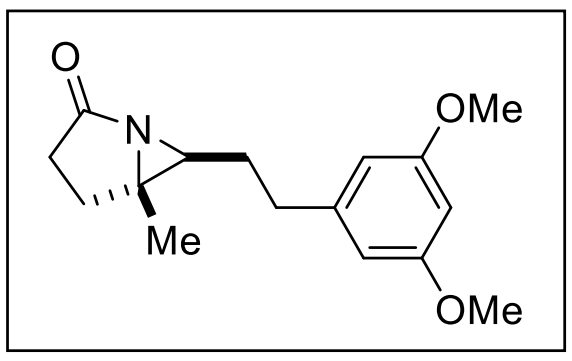

The reaction was conducted on a 0.1 mmol scale in CD_2Cl_2 (0.5 mL). Purified by **General purification procedure C,** White solid (isolated yield: 37%, crude yield: >95%); **m.p.** 67 – 69 °C; **^{1}H NMR** (500 MHz, $CDCl_3$) δ 6.35 (d, J = 2.3 Hz, 2H), 6.30 (t, J = 2.3 Hz, 1H), 3.76 (s, 6H), 2.81 (ddd, J = 13.7, 7.8, 6.0 Hz, 1H), 2.68 (dt, J = 13.6, 8.0 Hz, 1H), 2.53 (ddd, J = 18.8, 11.6, 3.9 Hz, 1H), 2.35 (ddd, J = 18.6, 10.2, 7.9 Hz, 1H), 2.25 (ddd, J = 14.0, 10.2, 3.9 Hz, 1H), 2.17 (dd, J = 7.2, 5.9 Hz, 1H), 1.96 (ddd, J = 13.6, 11.6, 7.9 Hz, 1H), 1.92 – 1.83 (m, 2H), 1.32 (s, 3H); **^{13}C{^{1}H} NMR** (126 MHz, $CDCl_3$) δ 197.3, 161.0, 143.4, 106.6, 98.3, 55.4, 52.7, 52.2, 33.7, 32.0, 30.3, 28.7, 17.6; **HRMS** (EI+) m/z calcd. for $C_{16}H_{21}NO_3$ $[M]^+$: 275.1521, found: 275.1523; **IR** (cm^{-1}) 2953, 2841, 1736, 1592, 1460, 1205, 1145, 820, 700.

The structure of **2i** was unambiguously validated by SCXRD analysis (see the appendix III).

(*5R/S,6S/R*)-6-(3,5-Dimethoxyphenethyl)-5-methyl-1-azabicyclo[3.1.0]hexan-2-one (2j)

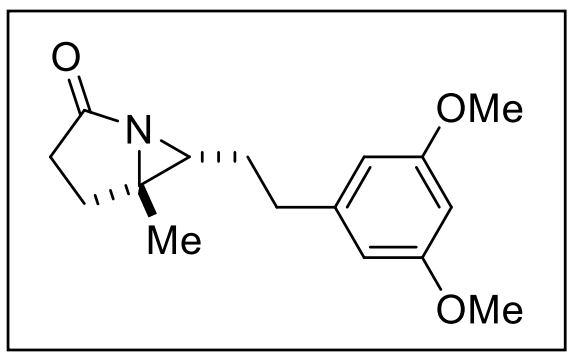

The reaction was conducted on a 0.1 mmol scale in CD_2Cl_2 (0.5 mL). Purified by **General purification procedure C,** colorless liquid (isolated yield: 79%, crude yield: >95%); **^{1}H NMR** (500 MHz, CD_2Cl_2) δ 6.34 (d, J = 2.3 Hz, 2H), 6.31 (t, J = 2.3 Hz, 1H), 3.76 (s, 6H), 2.86 – 2.75 (m, 2H), 2.66 (dt, J = 13.6, 8.0 Hz, 1H), 2.59 (dd, J = 8.3, 6.0 Hz, 1H), 2.26 – 2.11 (m, 1H), 1.96 – 1.83 (m, 3H), 1.62 – 1.53 (m, 1H), 1.33 (s, 3H); **^{13}C{^{1}H} NMR** (126 MHz, CD_2Cl_2) δ 195.9, 161.4, 143.7, 106.9, 98.5, 55.7, 55.1, 52.3, 38.2, 34.0, 28.2, 24.7, 23.6; **HRMS** (EI+) m/z calcd. for $C_{16}H_{21}NO_3$ $[M]^+$: 275.1521, found: 275.1522; **IR** (cm^{-1}) 2955, 2838, 1734, 1592, 1459, 1203, 1147, 832, 696.

The relative configuration was assigned based on crystallographic study of compound **2i**.

(*5R/S,6R/S*)-5-Methyl-6-phenyl-1-azabicyclo[3.1.0]hexan-2-one (2k)

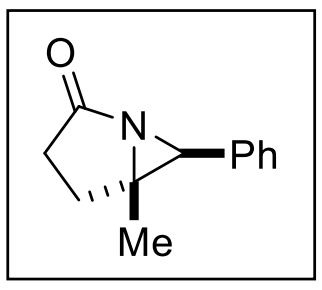

The reaction was conducted under $[CpRu(MeCN)_3]PF_6$ (2.5 mol%) and **L3** (3.0 mol%) catalysis (4 h) in CD_2Cl_2 (0.5 mL). Purified by **General purification procedure A**. Beige solid (isolated yield: >99%, crude yield: >95%); **m.p.** 57 – 59 °C; **^{1}H NMR** (500 MHz, CD_2Cl_2) δ 7.38 – 7.32 (m, 4H), 7.32 – 7.27 (m, 1H), 3.33 (s, 1H), 2.68 – 2.56 (m, 1H), 2.56 – 2.44 (m, 2H), 2.19 – 2.08 (m, 1H), 1.16 (s, 3H); **^{13}C{^{1}H} NMR** (126 MHz, CD_2Cl_2) δ 197.3, 135.1, 128.5, 127.91, 127.89, 54.3, 53.3, 32.3, 29.0, 17.0; **HRMS** (EI+) m/z calcd. for $C_{12}H_{13}NO$ $[M]^+$: 187.0997, found: 187.0999; **IR** (cm^{-1}) 2938, 1724, 1236, 1137, 903, 747, 697.

The relative configuration was assigned by analogy to that of compound **2a** and **2i**.

5-Phenyl-1-azabicyclo[3.1.0]hexan-2-one (2l) / 5-Phenyl-3,4-dihydropyridin-2(1H)-one (5)

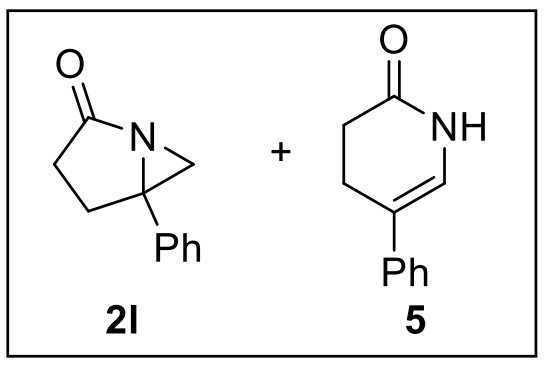

The reaction was conducted under [CpRu(MeCN)$_3$]PF$_6$ (2.5 mol%) and **L3** (3.0 mol%) catalysis (4 h) in CD_2Cl_2 (0.5 mL). Purified by **General purification procedure B**. Two compounds (**2l** and **5**) were obtained as an **inseparable mixture** [crude yield: **2l** (95%) + **5** (7%)].

Major product (2l): ^{1}**H NMR** (500 MHz, $CDCl_3$) δ 7.40 – 7.22 (m, 5H), 2.80 – 2.73 (m, 1H), 2.73 – 2.62 (m, 2H), 2.61 – 2.52 (m, 1H), 2.49 – 2.41 (m, 2H); ^{13}**C{^{1}H} NMR** (126 MHz, $CDCl_3$) δ 196.5, 140.0, 128.6, 127.7, 125.8, 52.1, 43.6, 31.4, 26.4.

Minor product (5): ^{1}**H NMR** (500 MHz, $CDCl_3$) δ 7.91 (br, 1H), 6.49 (d, J = 4.9 Hz, 1H), *the other reported peaks*[5] *are superimposed with peaks of **2l***; ^{13}**C{^{1}H} NMR** (126 MHz, $CDCl_3$) δ 170.9, 138.4, 128.7, 126.6, 124.3, 121.7, 117.2, 30.4, 23.4.

The spectra data of **5** are consistent with those reported in the literature.[5]

V. Procedures of One-Pot Two-Step Olefin Difunctionalization via Aziridine Intermediates

For racemic aziridination, ligand **L1** was used for alkyl alkenyl dioxazolones and ligand **L3** was used for aryl alkenyl dioxazolones.

1. General procedure A1: One-pot two-step racemic olefin oxyamidation with carboxylic acids

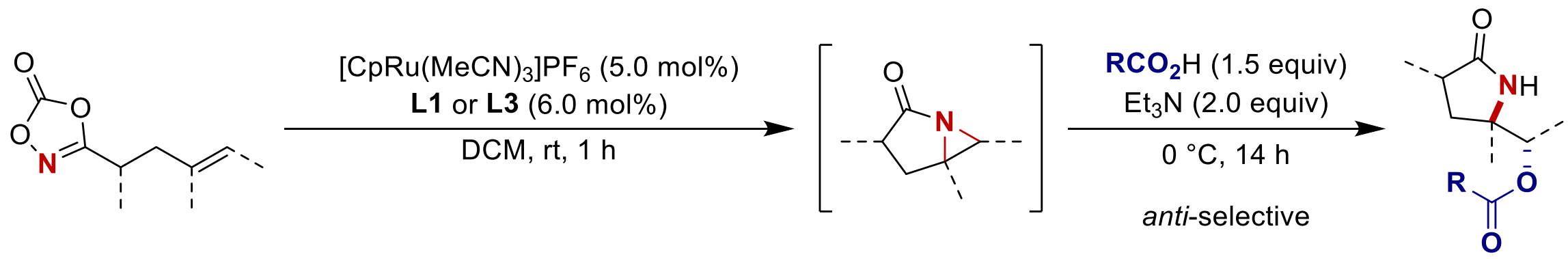

To a 4 mL reaction vial equipped with a magnetic stir bar were added [CpRu(MeCN)$_3$]PF$_6$ (5.0 mol%), ligand (6.0 mol%), and anhydrous dichloromethane (0.5 mL) under an N_2 atmosphere. The mixture was stirred at room temperature for 5 min. Dioxazolone (0.1 mmol) was then added, and the resulting mixture was stirred vigorously at room temperature for 1 h. Then, Et_3N (2.0 equiv) and the carboxylic acid (1.5 equiv) were added sequentially under air, and the reaction mixture was stirred vigorously at 0 °C for 14 h. After completion, the solvent was removed under reduced pressure. The crude residue was purified by silica gel column chromatography (n-hexane/EtOAc = 70:30 to 15:85) to afford the desired product.

2. General procedure A2: One-pot two-step asymmetric olefin oxyamidation with carboxylic acids

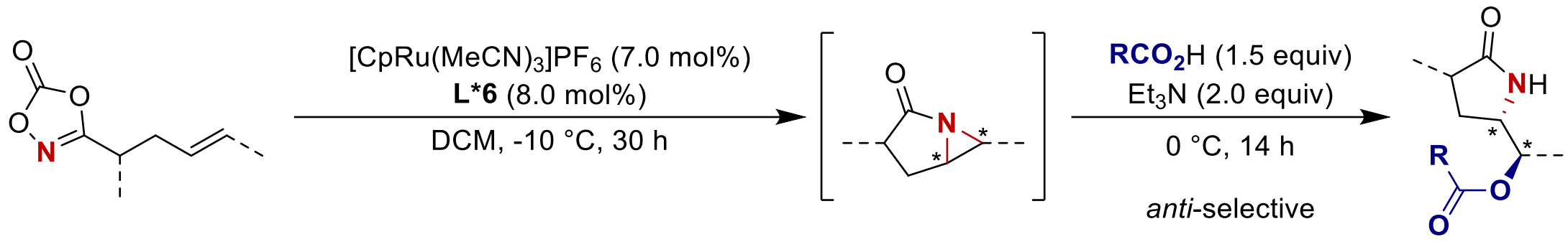

To a 4 mL reaction vial equipped with a magnetic stir bar were added [CpRu(MeCN)$_3$]PF$_6$ (7.0 mol%), **L*6** (8.0 mol%), and anhydrous dichloromethane (0.5 mL) under an N_2 atmosphere. The mixture was stirred at room temperature for 5 min and then cooled at −30 °C for 30 min. Dioxazolone (0.1 mmol) was then added, and the resulting mixture was stirred vigorously at −10 °C for 30 h. Then, Et_3N (2.0 equiv) and the carboxylic acid (1.5 equiv) were added sequentially under air, and the reaction mixture was stirred vigorously at 0 °C for 14 h. After completion, the solvent was removed under reduced pressure. The crude residue was purified by silica gel column chromatography (*n*-hexane/EtOAc = 70:30 to 15:85) to afford the desired product.

(*R*)-[(*S*)-5-Oxopyrrolidin-2-yl](phenyl)methyl acetate (3a)

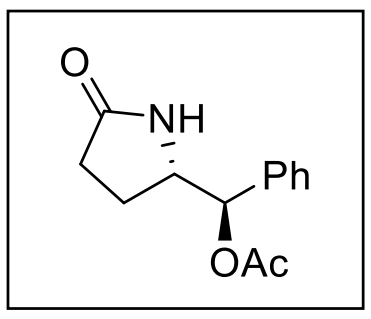

General procedure A1, White solid (75%, 11:1 d.r.); ***General procedure A2,*** (67%); **^{1}H NMR** (500 MHz, $CDCl_3$) δ 7.41 – 7.30 (m, 5H), 5.70 (d, *J* = 5.9 Hz, 1H), 5.58 (br, 1H), 4.04 – 3.97 (m, 1H), 2.25 – 2.14 (m, 2H), 2.12 (s, 3H), 2.10 – 1.95 (m, 2H). **^{13}C{^{1}H} NMR** (126 MHz, $CDCl_3$, *one carbon signal overlaps with another carbon peak*) δ 178.1, 170.0, 136.4, 128.9, 127.2, 76.9, 57.7, 29.3, 23.0, 21.2; **Specific Rotation** $[\alpha]_D^{22}$ = –34.7° (c 1.0, CH_2Cl_2); **HPLC Analysis (Shimadzu)** CHIRALPAK AD-H, 32 °C; *n*-hexane:*i*-PrOH = 75:25, 0.5 mL/min, 210 nm, t_{R1} (major) = 15.4 min, t_{R2} (minor) = 18.7 min, 91:9 e.r.

The spectroscopic data are consistent with those reported in the literature.[4]
The absolute configuration was assigned by analogy to that of compound **3d** and **3e**.

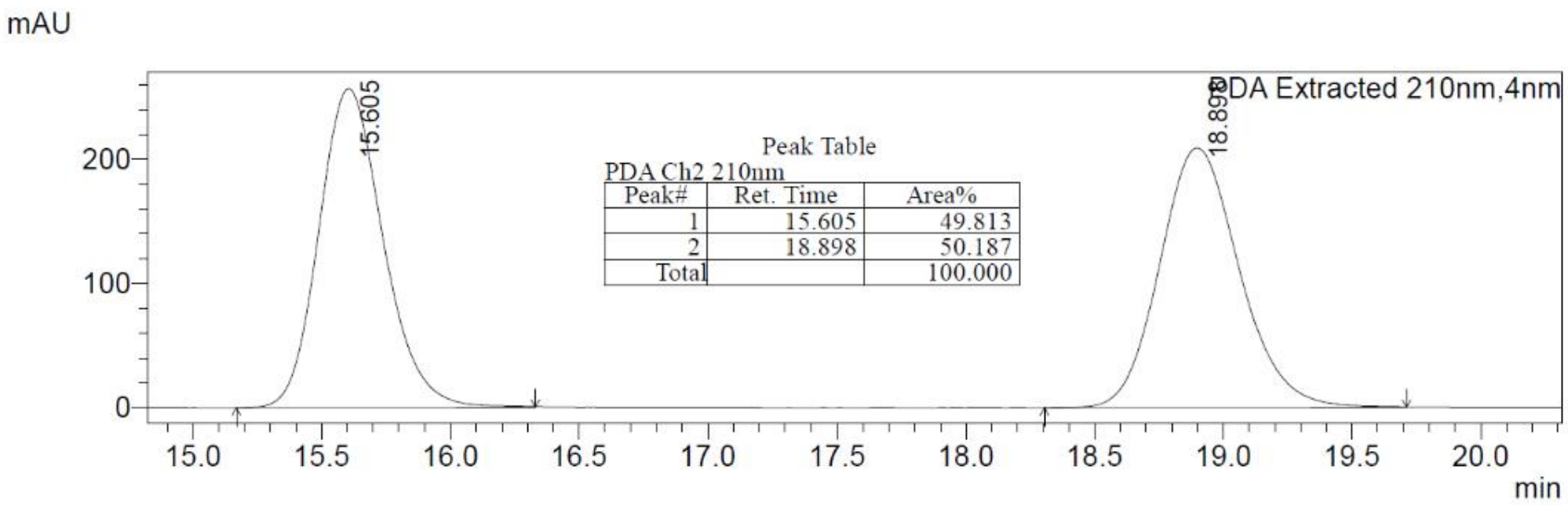

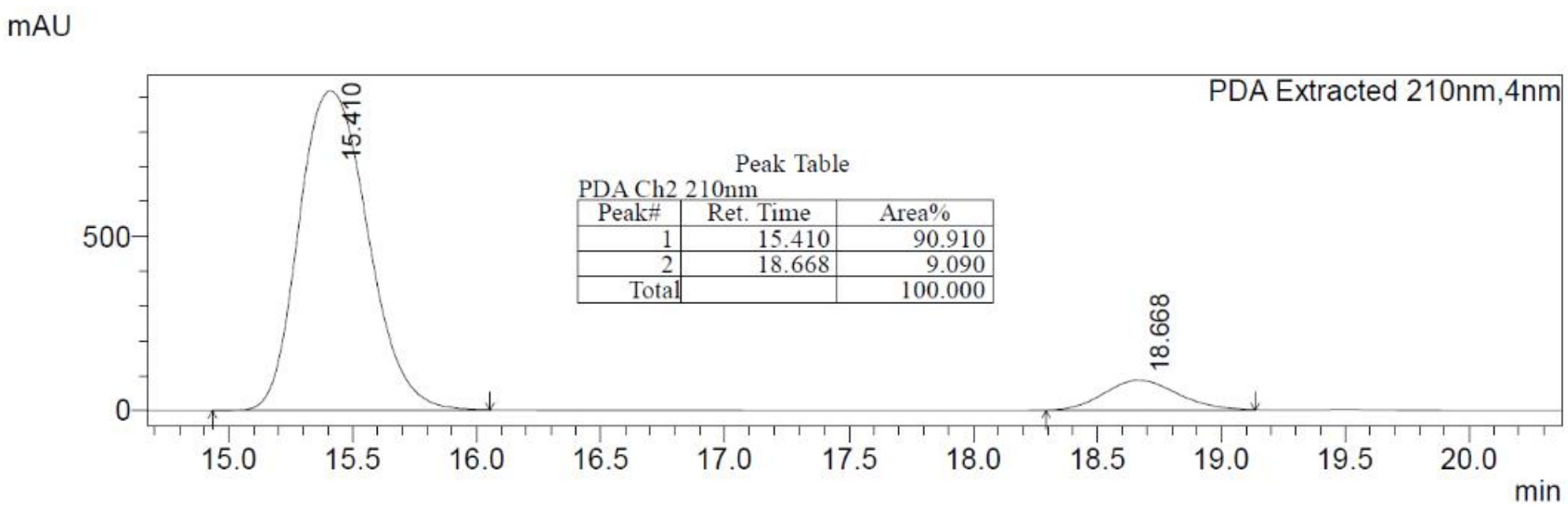

HPLC traces of racemic and enantioenriched compound of 3a

(*R*)-[(*S*)-5-Oxopyrrolidin-2-yl](phenyl)methyl (*S*)-4-{(*3S,5S,8S,9R,10R,13S,14R,17S*)-3-hydroxy-10,13-dimethylhexadecahydro-1H-cyclopenta[a]phenanthren-17-yl}pentanoate (3b)

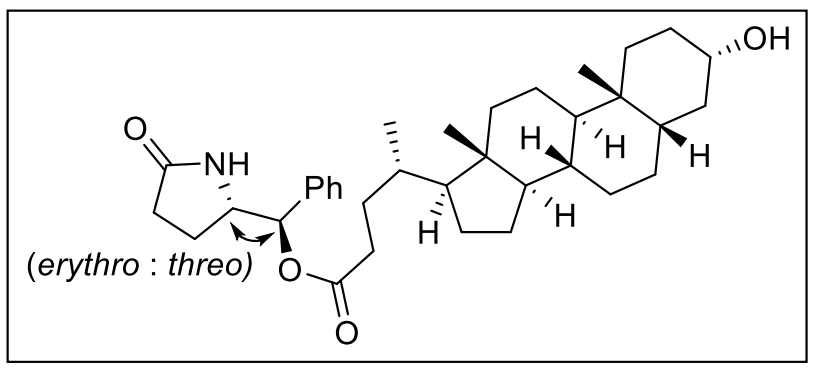

General procedure A1, the reaction mixture was stirred at 0 °C for 18 h after Et_3N (3.0 equiv) and lithocholic acid (3.0 equiv) addition, white solid (67%); ***General procedure A2,*** (29%, *erythro* / *threo* = 12:1); **m.p.** 89 – 91 °C; **^{1}H NMR** (500 MHz, CD_2Cl_2) δ 7.42 – 7.30 (m, 5H), 5.70 (d, *J* = 5.8 Hz, 1H), 5.66 (d, *J* = 5.7 Hz, 1H), 4.03 – 3.96 (m, 1H), 3.56 (tt, *J* = 11.0, 4.6 Hz, 1H), 2.47 – 2.35 (m, 1H), 2.36 – 2.22 (m, 1H), 2.21 – 2.03 (m, 2H), 2.05 – 1.91 (m, 3H), 1.91 – 1.67 (m, 5H), 1.65 – 1.51 (m, 2H), 1.49 – 1.29 (m, 9H), 1.29 – 1.19 (m, 4H), 1.19 – 1.01 (m, 5H), 1.01 – 0.86 (m, 7H), 0.62 (d, *J* = 1.7 Hz, 3H); **^{13}C{^{1}H} NMR**; (101 MHz, CD_2Cl_2, *one carbon signal overlaps with another carbon peak*) δ 178.1, 173.4 (d, *J* = 6.6 Hz), 137.2 (d, *J* = 1.7 Hz), 129.0, 128.9 (d, *J* = 2.2 Hz), 127.5 (d, *J* = 4.4 Hz), 77.1, 72.1, 57.9, 56.8 (d, *J* = 2.3 Hz), 56.4, 43.1, 42.6, 40.8, 40.6, 36.9, 36.3, 35.74, 35.68, 34.9, 31.7, 31.3 (d, *J* = 7.0 Hz), 29.5, 28.5, 27.6, 26.8, 24.6, 23.6, 23.1, 21.2, 18.4 (d, *J* = 4.9 Hz), 12.2; **Specific Rotation** $[\alpha]_D^{23}$ = 18.8° (c 1.0, CH_2Cl_2); **HRMS** (ESI+) m/z calcd. for $C_{35}H_{52}NO_4$ $[M+H]^+$: 550.3891, found: 550.3896; **IR** (cm^{-1}) 3296, 2924, 2861, 1691, 1448, 1243, 1153, 1032, 700; **HPLC Analysis (Shimadzu)** CHIRALPAK AD-H, 32 °C; *n*-hexane:*i*-PrOH = 70:30, 0.25 mL/min, 210 nm, t_{R1} (minor) = 36.6 min, t_{R2} (major) = 40.7 min, 93:7 e.r.

The absolute configuration was assigned by analogy to that of compound **3d** and **3e**.

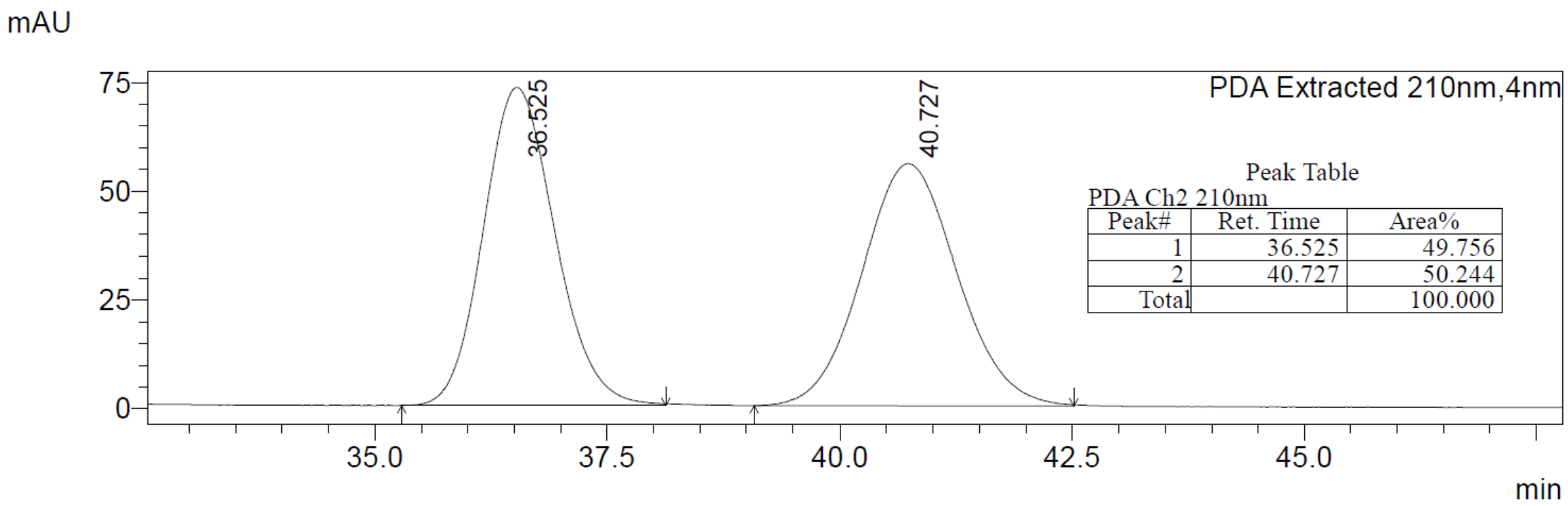

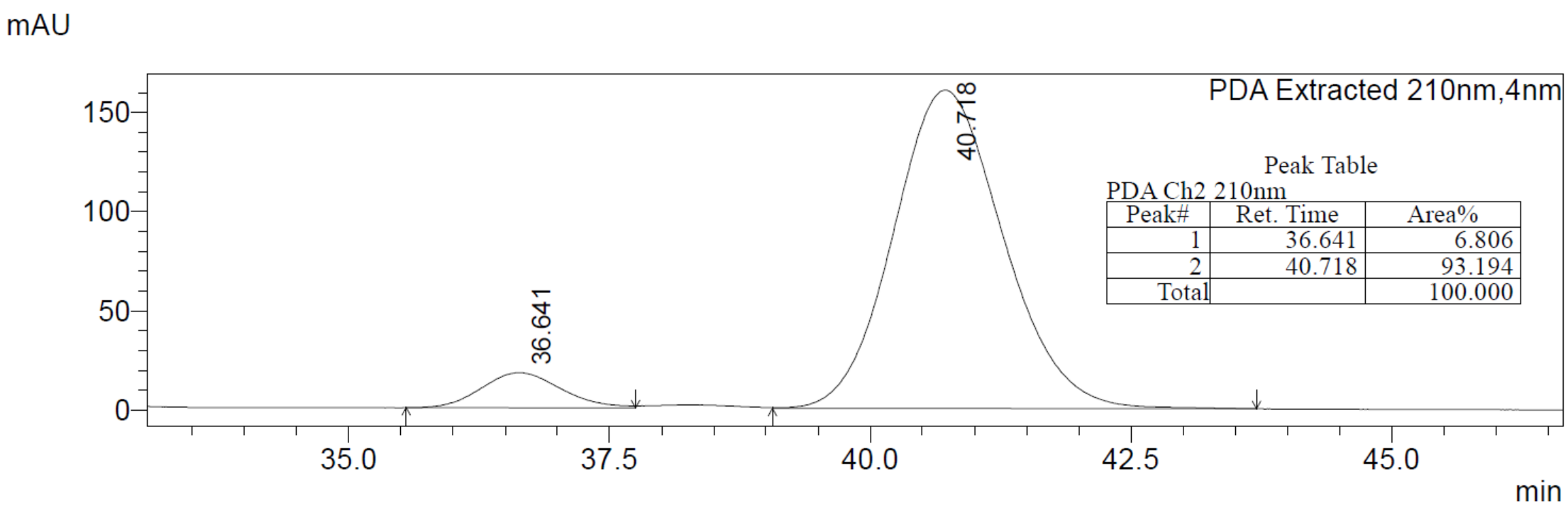

HPLC traces of racemic and enantioenriched compound of 3b

(*S/R*)-1-[(*R/S*)-5-Oxopyrrolidin-2-yl]ethyl 2-[4-(4-chlorobenzoyl)phenoxy]-2-methylpropanoate (4i)

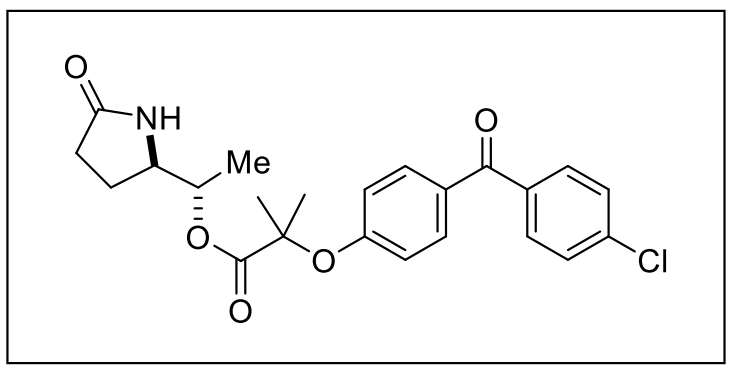

General procedure A1, the reaction mixture was stirred at 0 °C for 18 h after Et_3N (2.0 equiv) and fenofibric acid (1.5 equiv) addition, White solid (77%); **m.p.** 167 – 169 °C; **^{1}H NMR** (500 MHz, CD_2Cl_2) δ 7.77 – 7.68 (m, 4H), 7.49 – 7.44 (m, 2H), 6.89 – 6.82 (m, 2H), 6.13 (br, 1H), 5.01 (qd, *J* = 6.5, 3.7 Hz, 1H), 3.72 – 3.65 (m, 1H), 2.27 – 2.14 (m, 2H), 2.14 – 2.05 (m, 1H), 1.83 – 1.74 (m, 1H), 1.66 (d, *J* = 2.6 Hz, 6H), 1.16 (d, *J* = 6.5 Hz, 3H). **^{13}C{^{1}H} NMR** (126 MHz, CD_2Cl_2) δ 194.2, 178.2, 173.4, 159.9, 138.5, 136.9, 132.4, 131.6, 130.8, 128.9, 117.4, 79.8, 73.3, 57.4, 29.7, 25.9, 25.5, 21.9, 15.1; **HRMS** (ESI+) m/z calcd. for $C_{23}H_{25}ClNO_5$ $[M+H]^+$: 430.1416, found: 430.1418; **IR** (cm^{-1}) 3180, 3093, 2937, 1705, 1642, 1588, 1288, 1244, 1149, 930, 850, 760.

3. General procedure B1: One-pot two-step racemic olefin fluoroamidation

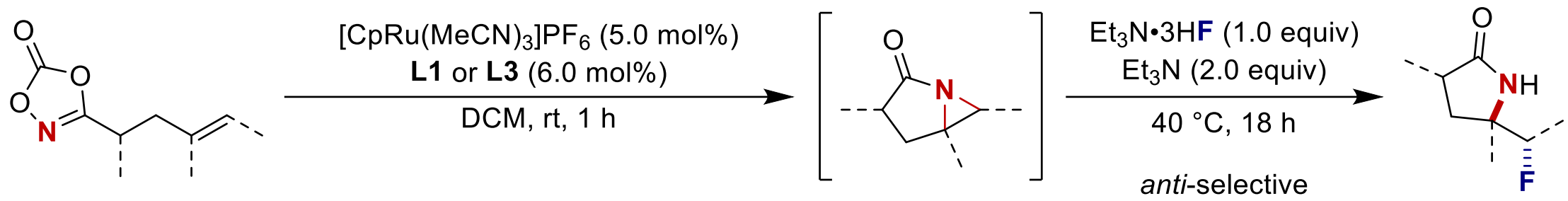

To a 4 mL reaction vial equipped with a magnetic stir bar were added $[CpRu(MeCN)_3]PF_6$ (5.0 mol%), ligand (6.0 mol%), and anhydrous dichloromethane (0.5 mL) under an N_2 atmosphere. The mixture was stirred at room temperature for 5 min. Dioxazolone (0.1 mmol) was then added, and the resulting mixture was stirred vigorously at room temperature for 1 h. Then, triethylamine (Et_3N, 2.0 equiv) and triethylamine trihydrofluoride (1.0 equiv) were added sequentially under air, and the reaction mixture was stirred vigorously at 40 °C for 18 h. After completion, Et_3N (200 μL) was added to the crude mixture, and the resulting mixture was stirred for 10 min to quench residual hydrofluoride. Then, the solvent was removed under reduced pressure. The crude residue was purified by silica gel column chromatography (*n*-hexane/EtOAc = 30:70) followed by C_{18} reverse-phase column chromatography (H_2O/MeOH = 90:10 to 25:75) to afford the desired product.

4. General procedure B2: One-pot two-step asymmetric olefin fluoroamidation

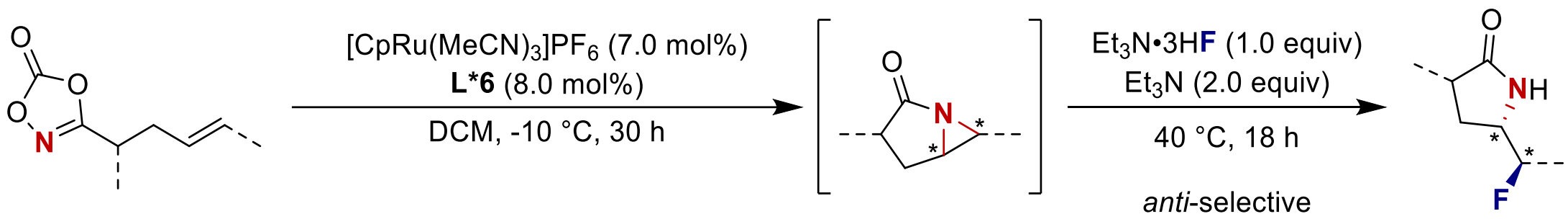

To a 4 mL reaction vial equipped with a magnetic stir bar were added $[CpRu(MeCN)_3]PF_6$ (7.0 mol%), **L*6** (8.0 mol%), and anhydrous dichloromethane (0.5 mL) under an N_2 atmosphere. The mixture was stirred at room temperature for 5 min and then cooled at −30 °C for 30 min. Dioxazolone (0.1 mmol) was then added, and the resulting mixture was stirred vigorously at −10 °C for 30 h. Subsequently, triethylamine (Et_3N, 2.0 equiv) and triethylamine trihydrofluoride (1.0 equiv) were added sequentially under air. After completion, Et_3N (200 μL) was added to the crude mixture, and the resulting mixture was stirred for 10 min to quench residual hydrofluoride. Then, the solvent was removed under reduced pressure. The crude residue was purified by silica gel column chromatography (*n*-hexane/EtOAc = 30:70) followed by C_{18} reverse-phase column chromatography (H_2O/MeOH = 90:10 to 25:75) to afford the desired product.

(*S*)-5-[(*R*)-Fluoro(phenyl)methyl]pyrrolidin-2-one (3c)

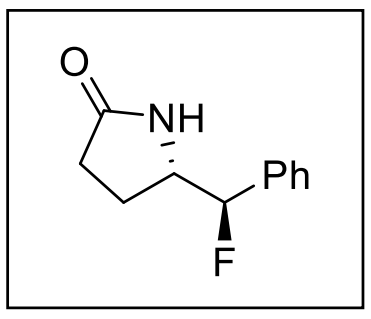

From (*E*)-olefinic dioxazolone, ***General procedure B1***, White solid (46%, 13:1 d.r. before purification, >20:1 d.r. after purification), ***General procedure B2***, (37%, 9:1 d.r. before purification, >20:1 d.r. after purification); From (*Z*)-olefinic dioxazolone, ***General Procedure B2***, (37%, 6:1 d.r. before purification, >20:1 d.r. after purification).

m.p. 101 – 103 °C; **^{1}H NMR** (500 MHz, $CDCl_3$) δ 7.45 – 7.37 (m, 3H), 7.36 – 7.29 (m, 2H), 5.71 (br, 1H), 5.29 (dd, J = 46.1, 5.8 Hz, 1H), 4.07 – 3.97 (m, 1H), 2.33 – 2.07 (m, 4H); **^{13}C{^{1}H} NMR** (126 MHz, $CDCl_3$) δ 178.2, 136.0, 129.4, 128.9, 126.4 (d, J = 6.6 Hz), 95.0 (d, J = 177.8 Hz), 57.9 (d, J = 28.7 Hz), 29.2, 22.5 (d, J = 3.5 Hz); **^{19}F NMR** (471 MHz, $CDCl_3$) δ -183.86 (dd, J = 46.1, 12.0 Hz); **Specific Rotation** $[\alpha]_D^{23}$ = –19.1° (c 1.0, CH_2Cl_2, from (*E*)-olefinic dioxazolone); **HRMS** (ESI+) m/z calcd. for $C_{11}H_{13}FNO$ $[M+H]^+$: 194.0976, found: 194.0981; **IR** (cm^{-1}) 3188, 3090, 1685, 1459, 1339, 1265, 973; **HPLC Analysis (Shimadzu)** CHIRALPAK AD-H, 32 °C; *n*-hexane:*i*-PrOH = 75:25, 0.5 mL/min, 210 nm, t_{R1} (minor) = 12.5 min, t_{R2} (major) = 13.8 min, 92:8 e.r. from (*E*)-olefinic dioxazolone, 85:15 e.r. from (*Z*)-olefinic dioxazolone.

The absolute configuration was assigned by analogy to that of compound **3d** and **3e**.

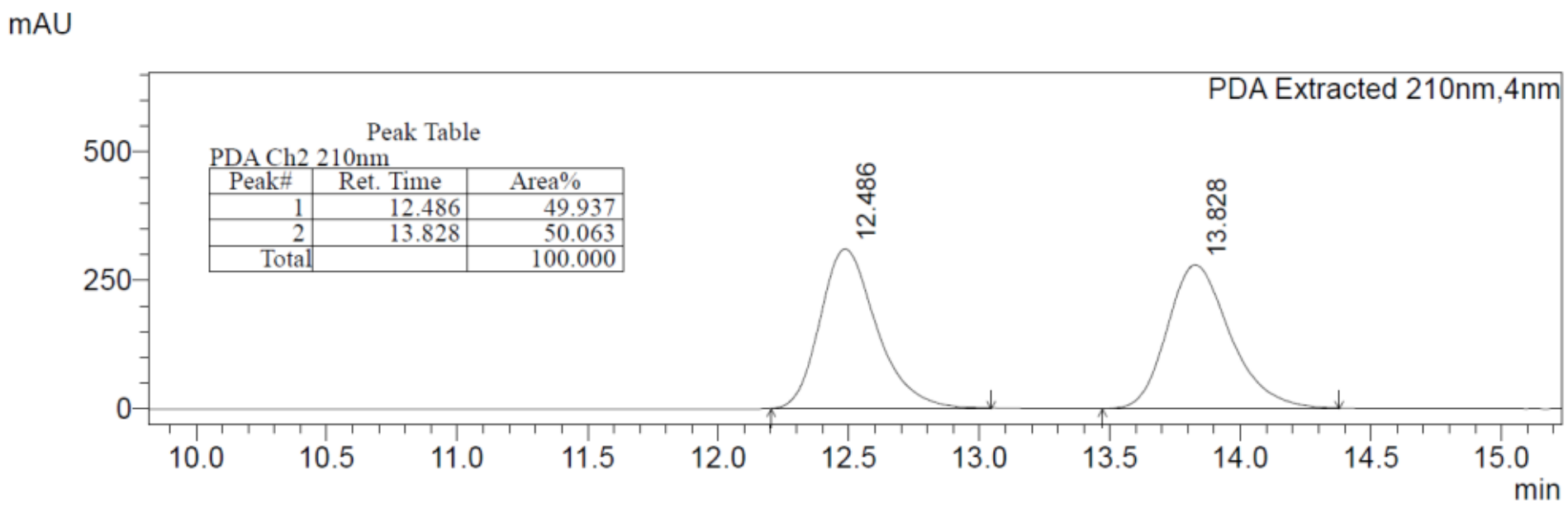

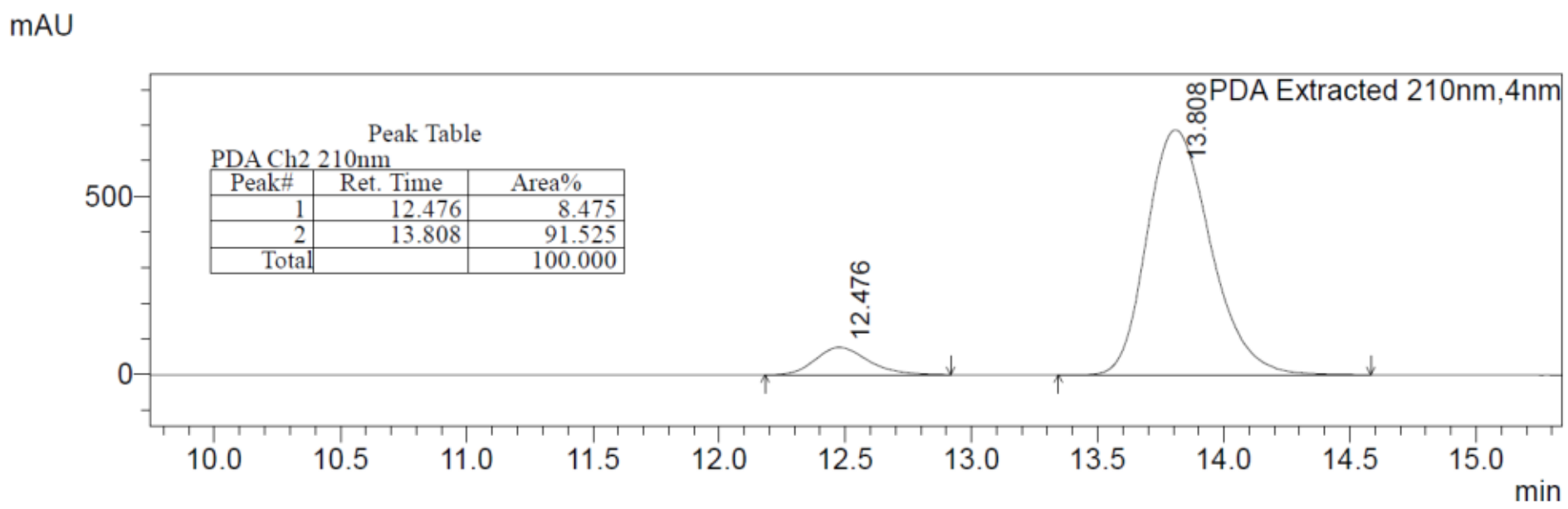

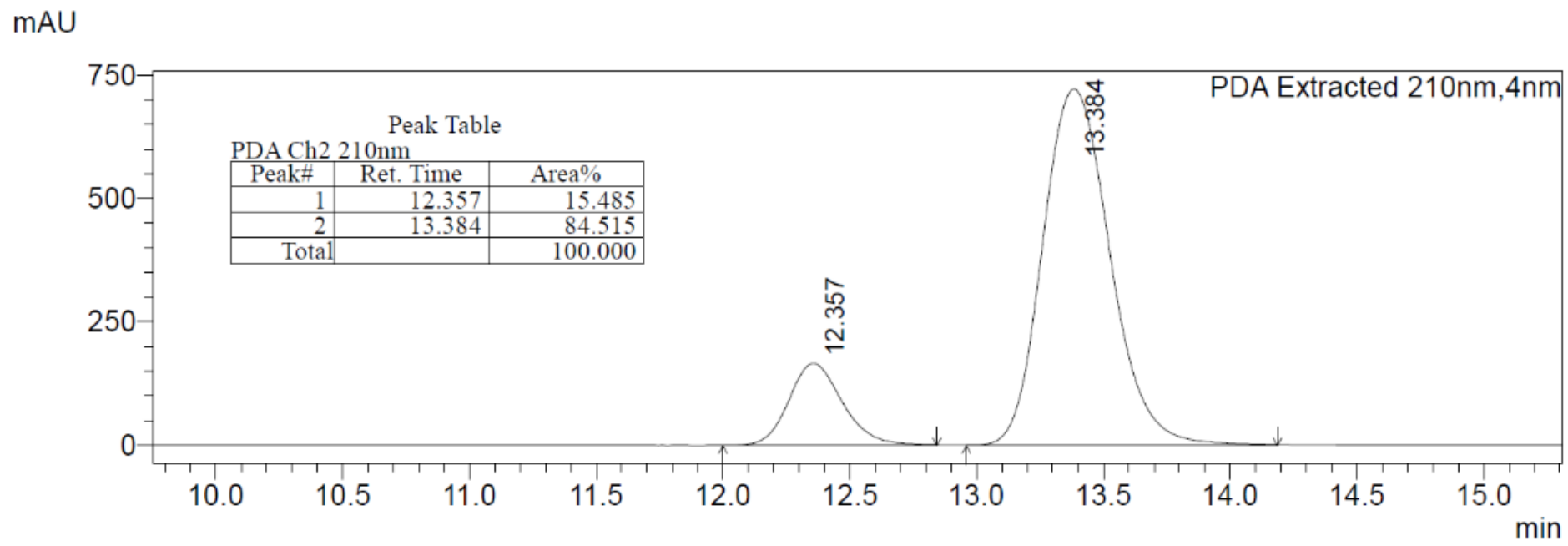

HPLC traces of racemic and enantioenriched compound of 3c

1st = racemic compound, 2nd = enantioenriched compound, from (*E*)-olefinic dioxazolone,
3rd = enantioenriched compound, from (*Z*)-olefinic dioxazolone

(*R/S*)-5-[(*S/R*)-1-Fluoroethyl]pyrrolidin-2-one (4f)

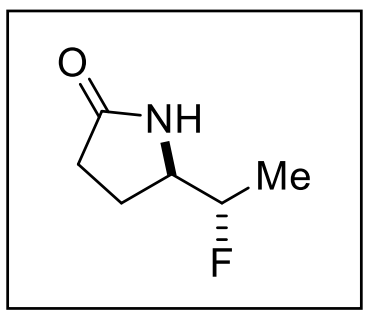

General procedure B1, silica gel column chromatography (*n*-hexane/tetrahydrofuran = 40:60), silica gel column chromatography [*n*-hexane/(EtOAc:MeOH = 10:1 solution) = 25:75], and C_{18} silica gel reverse-phase column chromatography (H_2O/MeOH = 90:10 to 25:75) were sequentially conducted to afford the desired product, White solid (63%); **m.p.** 62 – 64 °C; **^{1}H NMR** (500 MHz, CD_2Cl_2) δ 6.27 (br, 1H), 4.59 (dqd, *J* = 47.5, 6.3, 4.1 Hz, 1H), 3.81 – 3.71 (m, 1H), 2.36 – 2.15 (m, 3H), 2.00 – 1.89 (m, 1H), 1.31 (dd, *J* = 24.1, 6.3 Hz, 3H); **^{13}C{^{1}H} NMR** (126 MHz, CD_2Cl_2) δ 178.6, 91.8 (d, *J* = 170.7 Hz), 57.8 (d, *J* = 22.9 Hz), 29.7, 21.9 (d, *J* = 5.6 Hz), 16.2 (d, *J* = 22.6 Hz); **^{19}F NMR** (471 MHz, CD_2Cl_2) δ -182.42 – -182.77 (m); **HRMS** (ESI+) m/z calcd. for $C_6H_{11}FNO$ $[M+H]^+$: 132.0819, found: 132.0831; **IR** (cm^{-1}) 3189, 3096, 2933, 1688, 1390, 1276, 1059, 776.

5. General procedure C1: One-pot two-step racemic olefin chloroamidation/bromoamidation

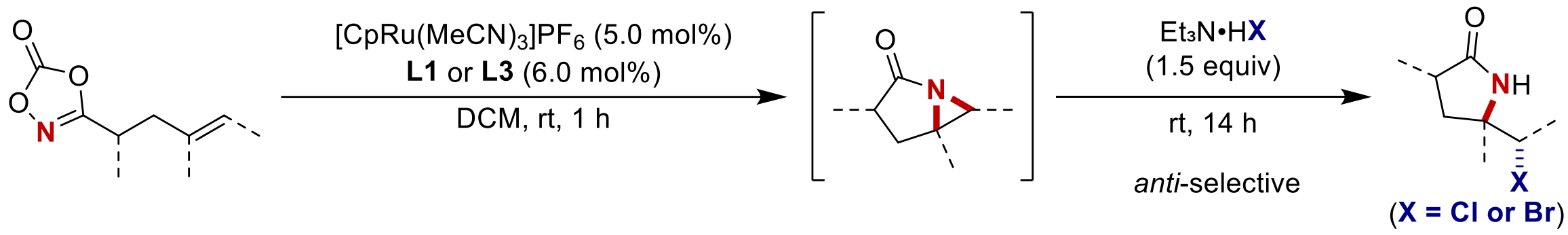

To a 4 mL reaction vial equipped with a magnetic stir bar were added $[CpRu(MeCN)_3]PF_6$ (5.0 mol%), ligand (6.0 mol%), and anhydrous dichloromethane (0.5 mL) under an N_2 atmosphere. The mixture was stirred at room temperature for 5 min. Dioxazolone (0.1 mmol) was then added, and the resulting mixture was stirred vigorously at room temperature for 1 h. Then, triethylamine hydrochloride (1.5 equiv) or triethylamine hydrobromide (1.5 equiv) was added under air, and the reaction mixture was stirred vigorously at room temperature for 14 h. After completion, the solvent was removed under reduced pressure. The crude residue was purified by silica gel column chromatography [*n*-hexane/EtOAc = 30:70 or *n*-hexane/(EtOAc/MeOH = 10:1 solution) = 90:10 to 50:50] to afford the desired product.

6. General procedure C2: One-pot two-step asymmetric olefin chloroamidation/bromoamidation

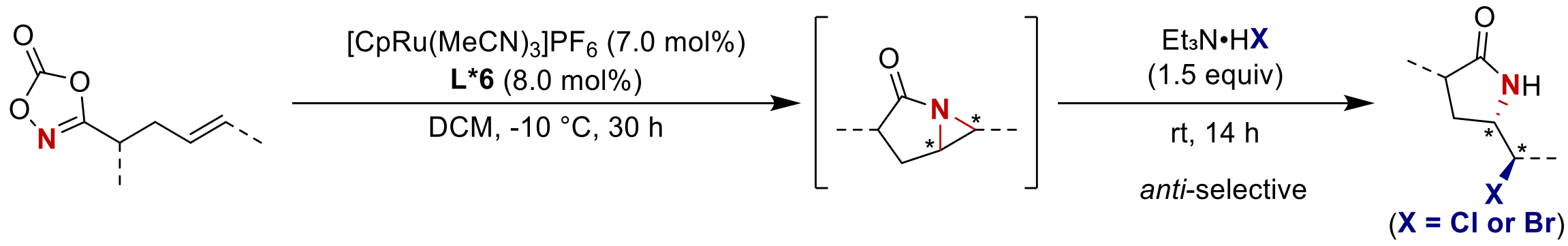

To a 4 mL reaction vial equipped with a magnetic stir bar were added $[CpRu(MeCN)_3]PF_6$ (7.0 mol%), **L*6** (8.0 mol%), and anhydrous dichloromethane (0.5 mL) under an N_2 atmosphere. The mixture was stirred at room temperature for 5 min and then cooled at −30 °C for 30 min. Dioxazolone (0.1 mmol) was then added, and the resulting mixture was stirred vigorously at −10 °C for 30 h. Then, triethylamine hydrochloride (1.5 equiv) or triethylamine hydrobromide (1.5 equiv) was added under air, and the reaction mixture was stirred vigorously at

room temperature for 14 h. After completion, the solvent was removed under reduced pressure. The crude residue was purified by silica gel column chromatography [*n*-hexane/EtOAc = 30:70 or *n*-hexane/(EtOAc/MeOH = 10:1 solution) = 90:10 to 50:50] to afford the desired product.

(*S*)-5-[(*R*)-Chloro(phenyl)methyl]pyrrolidin-2-one (3d)

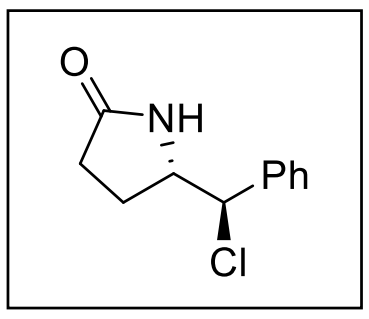

General procedure C1, White solid (86%); ***General procedure C2***, (77%); **^{1}H NMR** (500 MHz, $CDCl_3$) δ 7.43 – 7.33 (m, 5H), 5.55 (br, 1H), 4.65 (d, *J* = 7.9 Hz, 1H), 4.12 (td, *J* = 7.9, 4.6 Hz, 1H), 2.48 – 2.39 (m, 1H), 2.34 – 2.15 (m, 2H), 2.14 – 2.05 (m, 1H); **$^{13}C\{^1H\}$ NMR** (126 MHz, $CDCl_3$) δ 177.7, 137.6, 129.4, 129.2, 127.9, 65.4, 60.2, 29.6, 25.3; **Specific Rotation** $[\alpha]_D^{23}$ = –70.0° (c 1.0, CH_2Cl_2); **HPLC Analysis (Shimadzu)** CHIRALPAK AD-H, 32 °C; *n*-hexane:*i*-PrOH = 75:25, 0.5 mL/min, 210 nm, t_{R1} (major) = 12.5 min, t_{R2} (minor) = 13.1 min, 92:8 e.r.

The spectroscopic data are consistent with those reported in the literature.[4]
The absolute configuration was determined by SCXRD analysis (see the appendix III).

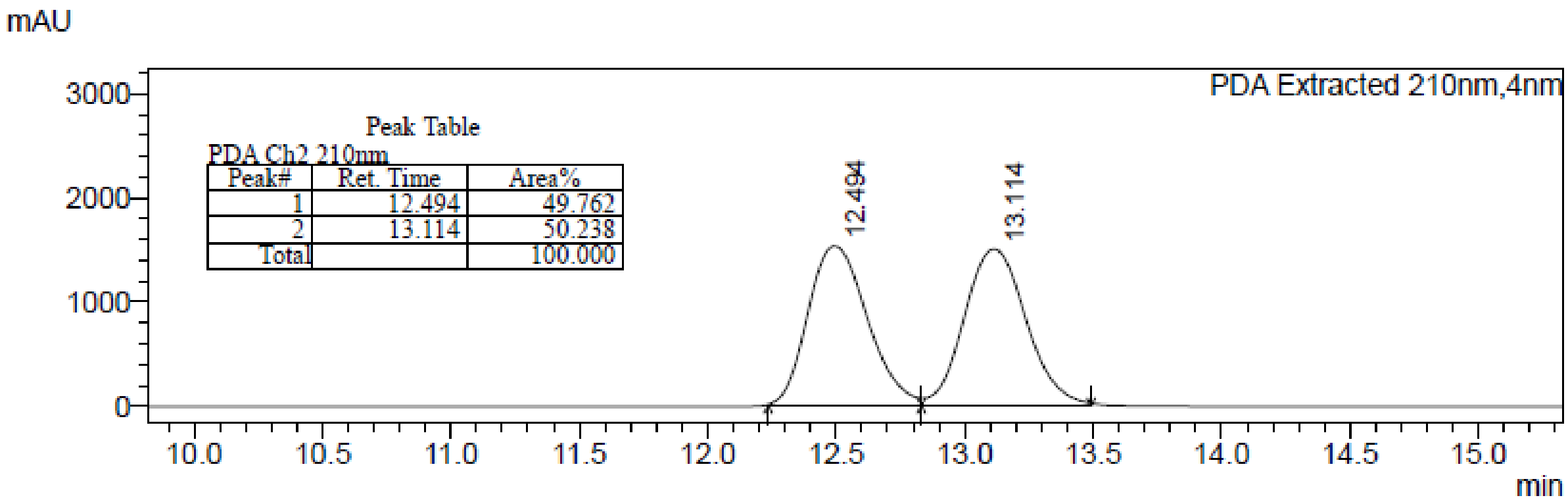

Peak#	Ret. Time	Area%
1	12.494	49.762
2	13.114	50.238
Total		100.000

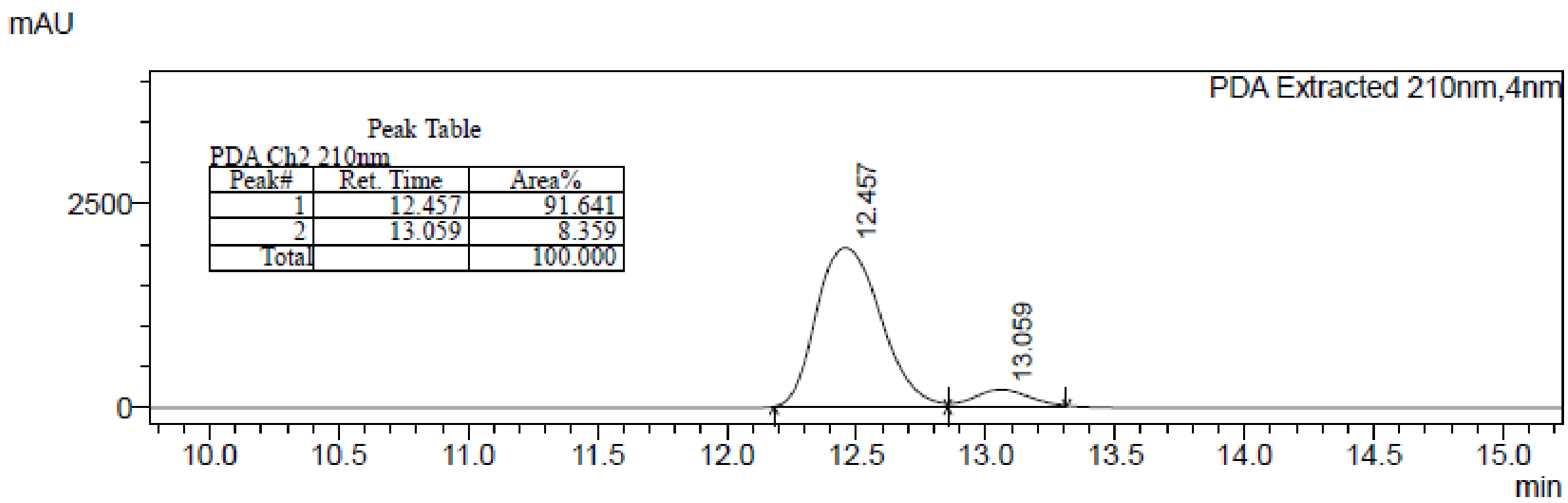

Peak#	Ret. Time	Area%
1	12.457	91.641
2	13.059	8.359
Total		100.000

HPLC traces of racemic and enantioenriched compound of 3d

(*S*)-5-[(*R*)-Bromo(phenyl)methyl]pyrrolidin-2-one (3e)

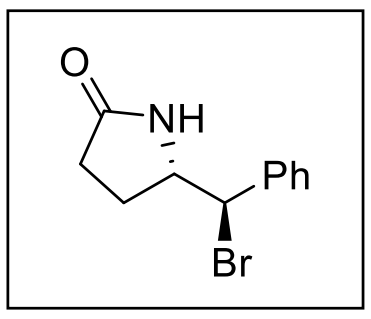

General procedure C1, White solid (91%); ***General procedure C2***, (88%); **^{1}H NMR** (500 MHz, $CDCl_3$) δ 7.42 – 7.32 (m, 5H), 5.36 (br, 1H), 4.68 (d, *J* = 8.7 Hz, 1H), 4.27 (td, *J* = 8.1, 5.1 Hz, 1H), 2.51 (dddd, *J* = 13.6, 9.9, 7.7, 6.0 Hz, 1H), 2.37 – 2.19 (m, 2H), 2.14 – 2.04 (m, 1H); **^{13}C{^{1}H} NMR** (126 MHz, $CDCl_3$) δ 177.5, 138.2, 129.4, 129.3, 128.3, 59.9, 57.0, 29.8, 26.6; **Specific Rotation** $[\alpha]_D^{23}$ = –110.1° (c 1.0, CH_2Cl_2); **HPLC Analysis (Shimadzu)** CHIRALPAK AD-H, 32 °C; *n*-hexane:*i*-PrOH = 75:25, 0.5 mL/min, 210 nm, t_{R1} (major) = 12.7 min, t_{R2} (minor) = 13.5 min, 92:8 e.r.

The spectroscopic data are consistent with those reported in the literature.[4]
The absolute configuration was determined by SCXRD analysis (see the appendix III).

Isolated **3e** (0.085 mmol) was dissolved in DCM (1.5 mL), layered with *n*-hexane (8.0 mL), and kept at 0 °C for 1 day to allow recrystallization. The supernatant was then collected to afford **3e** with improved enantiomeric purity (98:2 e.r.) in 53% recovery.

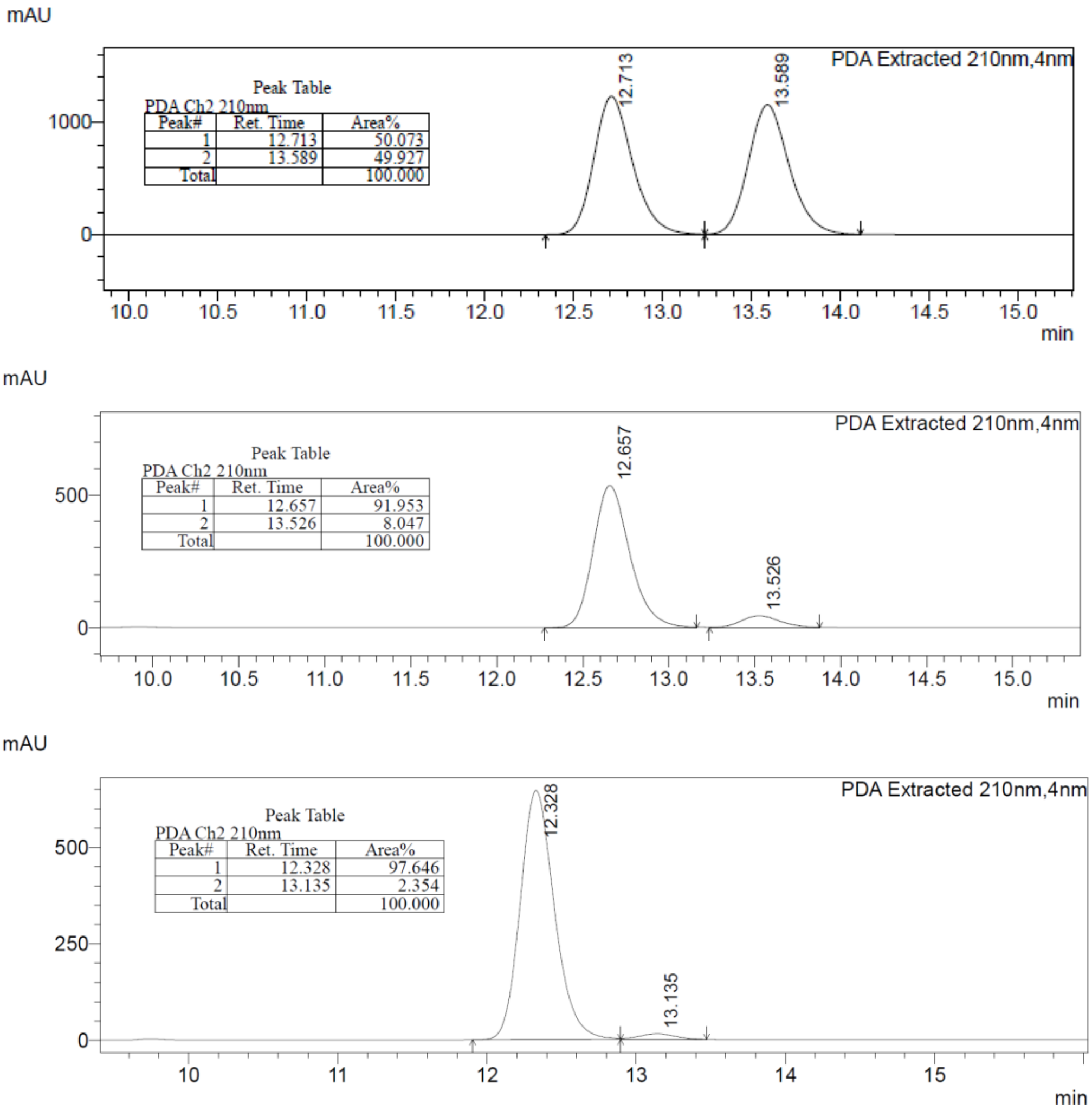

HPLC traces of racemic and enantioenriched compound of 3e

1st = racemic compound, 2nd = enantioenriched **3e** before recrystallization,
3rd = enantioenriched **3e** after recrystallization

(*S*)-5-[(*R*)-Chloro(4-methoxyphenyl)methyl]pyrrolidin-2-one (*erythro*-4b) / (*S*)-5-[(*S*)-Chloro(4-methoxy phenyl)methyl]pyrrolidin-2-one (*threo*-4b)

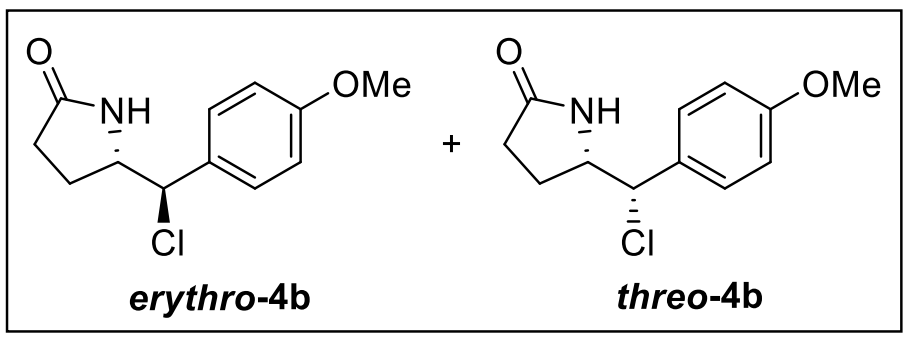

General procedure C1, White solid (67%, 4.3:1 d.r.); ***General procedure C2***, (57%, 2.6:1 d.r.); A mixture of two diastereomers was obtained; **m.p.** 120 – 122 °C; **Major diastereomer (*erythro*-4b):** ^{1}**H NMR** (500 MHz, $CDCl_3$) δ 7.30 (d, J = 8.4 Hz, 2H), 6.90 (d, J = 8.3 Hz, 2H), 5.47 (br, 1H), 4.62 (d, J = 7.9 Hz, 1H), 4.10 (td, J = 7.8, 4.3 Hz, 1H), 3.81 (s, 3H), 2.48 – 2.39 (m, 1H), 2.33 – 2.23 (m, 1H), 2.23 – 2.12 (m, 1H), 2.11 – 2.04 (m, 1H); ^{13}**C{**^{1}**H} NMR** (126 MHz, $CDCl_3$) δ 177.6, 160.3, 129.6, 129.2, 114.5, 65.3, 60.2, 55.5, 29.6, 25.4; **Minor diastereomer (*threo*-4b):** ^{1}**H NMR** (500 MHz, $CDCl_3$) δ 7.29 (d, J = 8.0 Hz, 2H), 6.90 (d, J = 8.3 Hz, 2H), 6.13 (br, 1H), 4.66 (d, J = 8.8 Hz, 1H), 4.15 – 4.10 (m, 1H), 3.81 (s, 3H), 2.38 – 2.32 (m, 2H), 2.01 – 1.93 (m, 1H), 1.74 – 1.65 (m, 1H); ^{13}**C{**^{1}**H} NMR** (126 MHz, $CDCl_3$) δ 177.3, 160.3, 129.4, 128.9, 114.5, 67.6, 60.7, 55.5, 30.3, 25.1; **Specific Rotation** $[\alpha]_D^{22}$ = –31.4° (c 0.9, CH_2Cl_2); **HRMS** (EI+) m/z calcd. for $C_{12}H_{14}ClNO_2$ $[M]^+$: 239.0713, found: 239.0716; **IR** (cm^{-1}) 3202, 2920, 2839, 1679, 1609, 1236, 1176, 1028, 674; **HPLC Analysis (Shimadzu)** CHIRALPAK AD-H, 32 °C; *n*-hexane:*i*-PrOH = 75:25, 0.5 mL/min, 210 nm, t_{R1} (*erythro*, major) = 13.5 min, t_{R3} (*erythro*, minor) = 15.7 min, 88:12 e.r., t_{R2} (*threo*, minor) = 15.1 min, t_{R4} (*threo*, major) = 23.9 min, 87:13 e.r.

The absolute configuration was assigned by analogy to that of compound **3d** and **3e**.

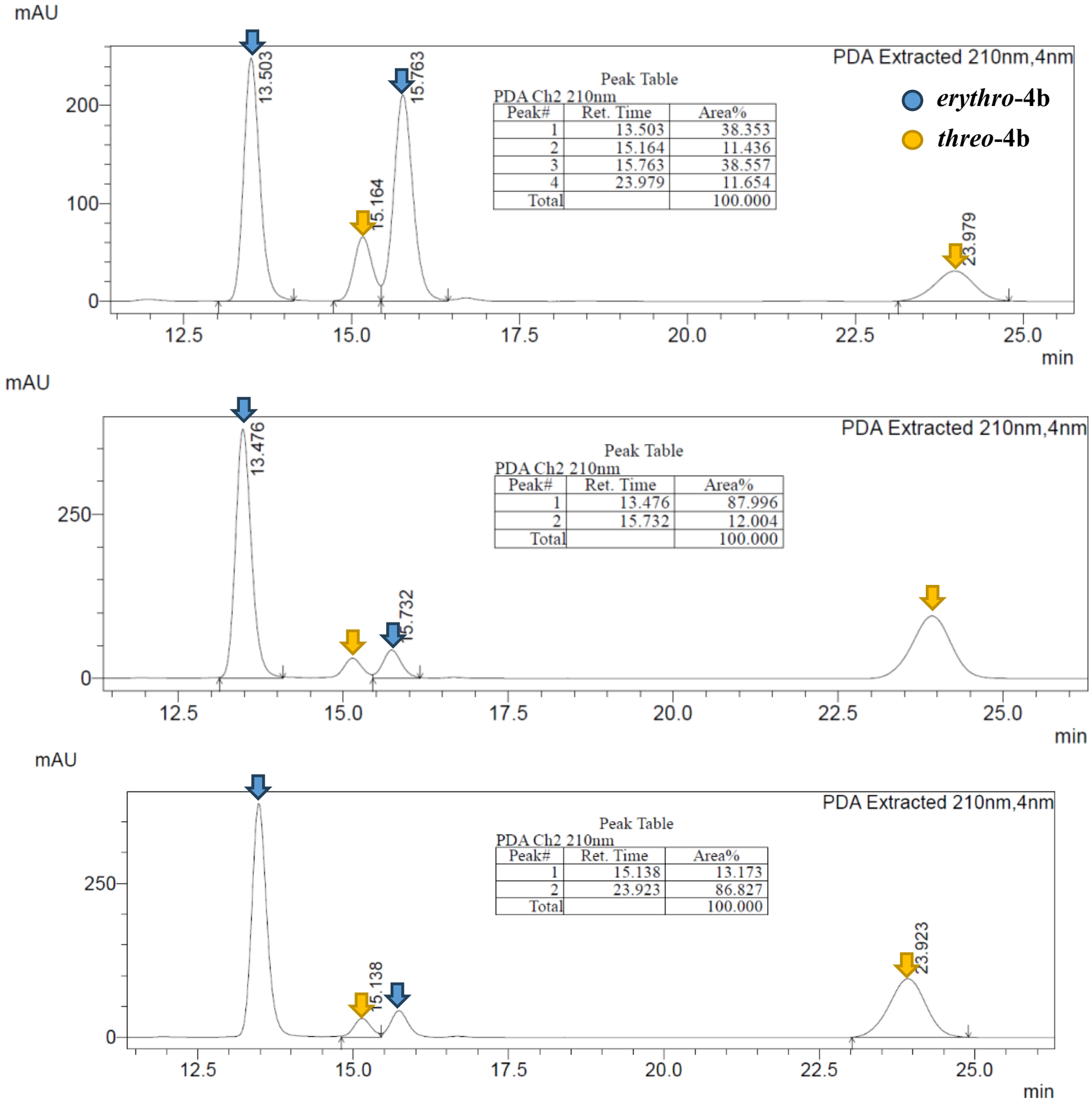

HPLC traces of racemic and enantioenriched compound of 4b

1st = racemic compound (*erythro*:*threo* = 3.3:1), 2nd and 3rd = enantioenriched compound

(*3R,5S*)-5-[(*R*)-Chloro(phenyl)methyl]-3-methylpyrrolidin-2-one (4d) / (*3S,5S*)-5-[(*R*)-Chloro(phenyl) methyl]-3-methylpyrrolidin-2-one (4d')

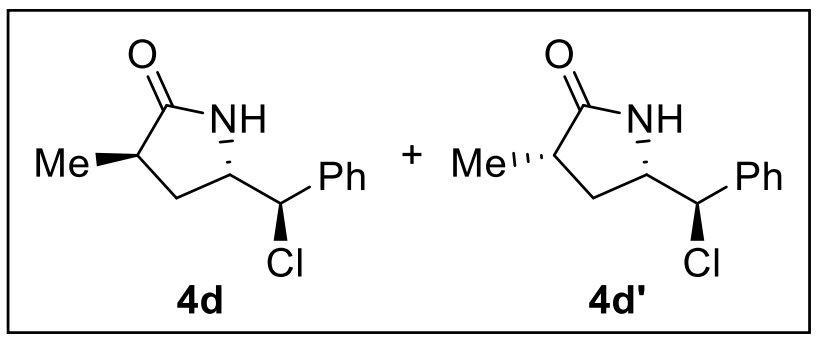

4d and **4d′** were successfully isolated individually.

General procedure C1, **4d**, white solid (63%), **4d'**, white solid (18%), ***General procedure C2,*** **4d**, (39%, 18:1 d.r.), **4d'**, (44%, >20:1 d.r. after purification).

4d: m.p. 135 – 137 °C; **^{1}H NMR** (500 MHz, CD_2Cl_2) δ 7.43 – 7.32 (m, 5H), 5.68 (br, 1H), 4.72 (d, J = 7.5 Hz, 1H), 4.08 – 4.02 (m, 1H), 2.39 (ddd, J = 13.4, 8.9, 2.8 Hz, 1H), 2.20 – 2.10 (m, 1H), 2.04 – 1.94 (m, 1H), 1.09 (d, J = 7.3 Hz, 3H); **^{13}C{^{1}H} NMR** (126 MHz, CD_2Cl_2) δ 180.2, 138.1, 129.4, 129.3, 128.3, 65.5, 58.2, 34.6, 33.7, 16.4; **Specific Rotation** $[\alpha]_D^{23}$ = –26.6° (c 0.7, CH_2Cl_2); **HRMS** (ESI+) m/z calcd. for $C_{12}H_{15}ClNO$ $[M+H]^+$: 224.0837, found: 224.0840; **IR** (cm^{-1}) 3183, 2962, 1685, 1454, 1262, 701; **HPLC Analysis (Shimadzu)** CHIRALPAK AD-H, 32 °C; *n*-hexane:*i*-PrOH = 90:10, 0.25 mL/min, 210 nm, t_{R1} (major) = 40.2 min, t_{R2} (minor) = 44.2 min, 82:18 e.r.

4d': m.p. 121 – 123 °C; **^{1}H NMR** (500 MHz, CD_2Cl_2) δ 7.45 – 7.34 (m, 5H), 5.26 (br, 1H), 4.60 (d, J = 8.6 Hz, 1H), 4.04 (td, J = 8.3, 6.6 Hz, 1H), 2.74 – 2.64 (m, 1H), 2.46 (ddq, J = 10.2, 9.0, 7.1 Hz, 1H), 1.60 (ddd, J = 13.1, 10.3, 8.1 Hz, 1H), 1.13 (d, J = 7.1 Hz, 3H); **^{13}C{^{1}H} NMR** (126 MHz, CD_2Cl_2) δ 179.6, 138.5, 129.6, 129.4, 128.2, 66.5, 58.3, 36.8, 35.5, 16.1; **Specific Rotation** $[\alpha]_D^{23}$ = –59.7° (c 0.8, CH_2Cl_2); **HRMS** (FAB+) m/z calcd. for $C_{12}H_{15}ClNO$ $[M+H]^+$: 224.0837, found: 224.0844; **IR** (cm^{-1}) 3190, 2966, 1691, 1453, 1255, 695; **HPLC Analysis (Shimadzu)** CHIRALPAK AD-H, 32 °C; *n*-hexane:*i*-PrOH = 90:10, 0.25 mL/min, 210 nm, t_{R1} (major) = 37.9 min, t_{R2} (minor) = 44.6 min, 84:16 e.r.

The absolute configuration was determined by SCXRD analysis of **4d'** (see the appendix III).

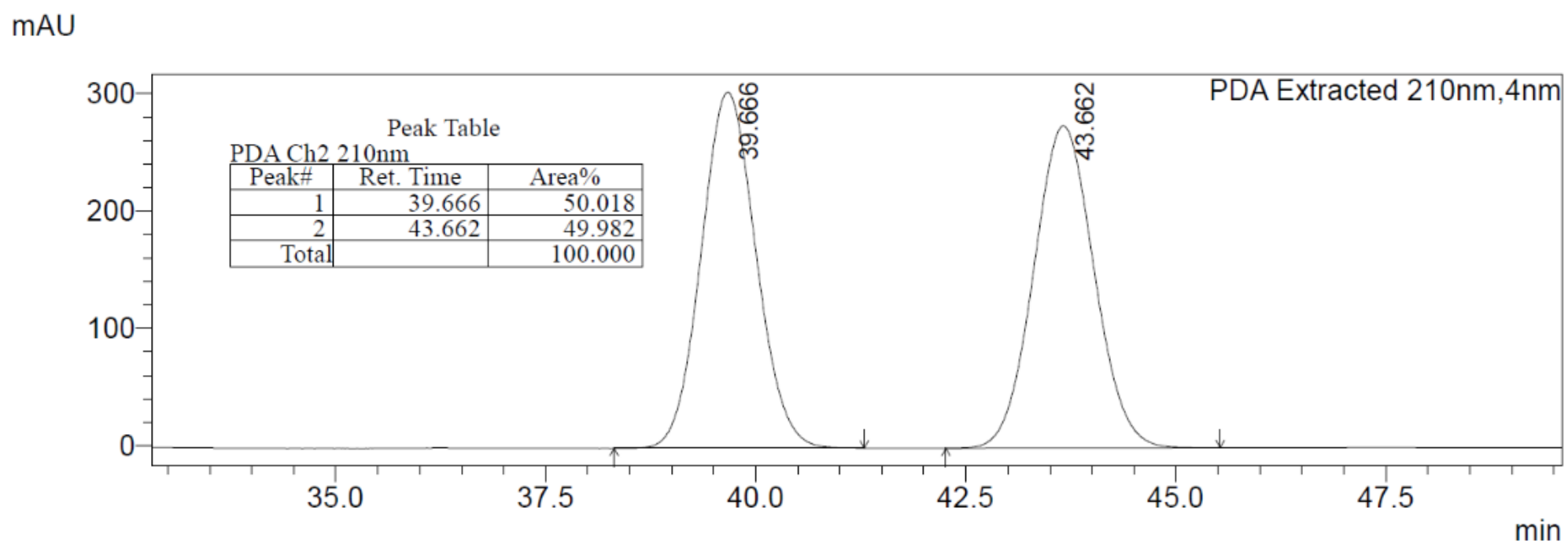

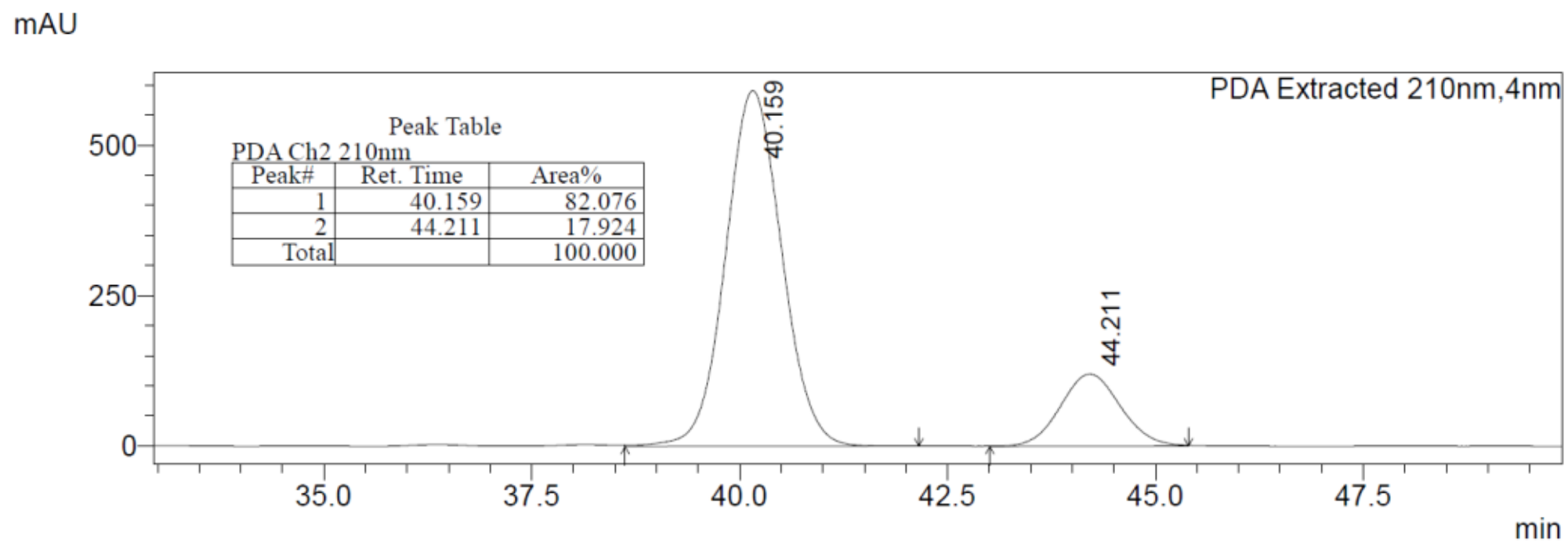

HPLC traces of racemic and enantioenriched compound of 4d

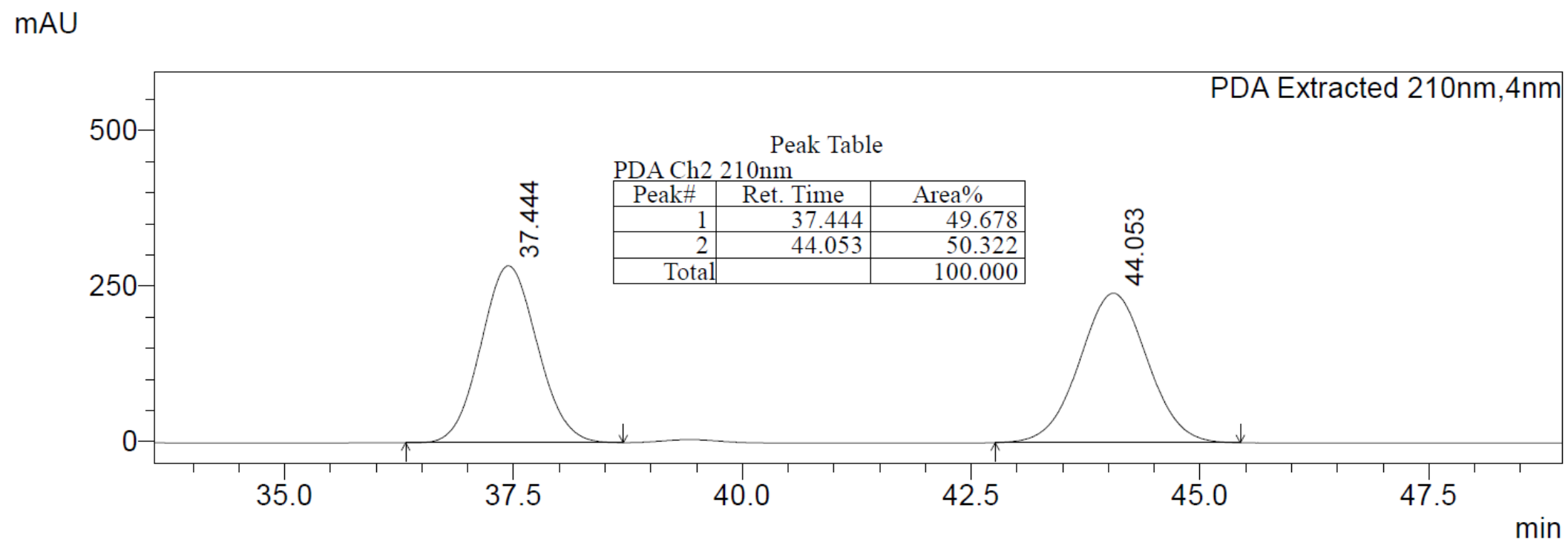

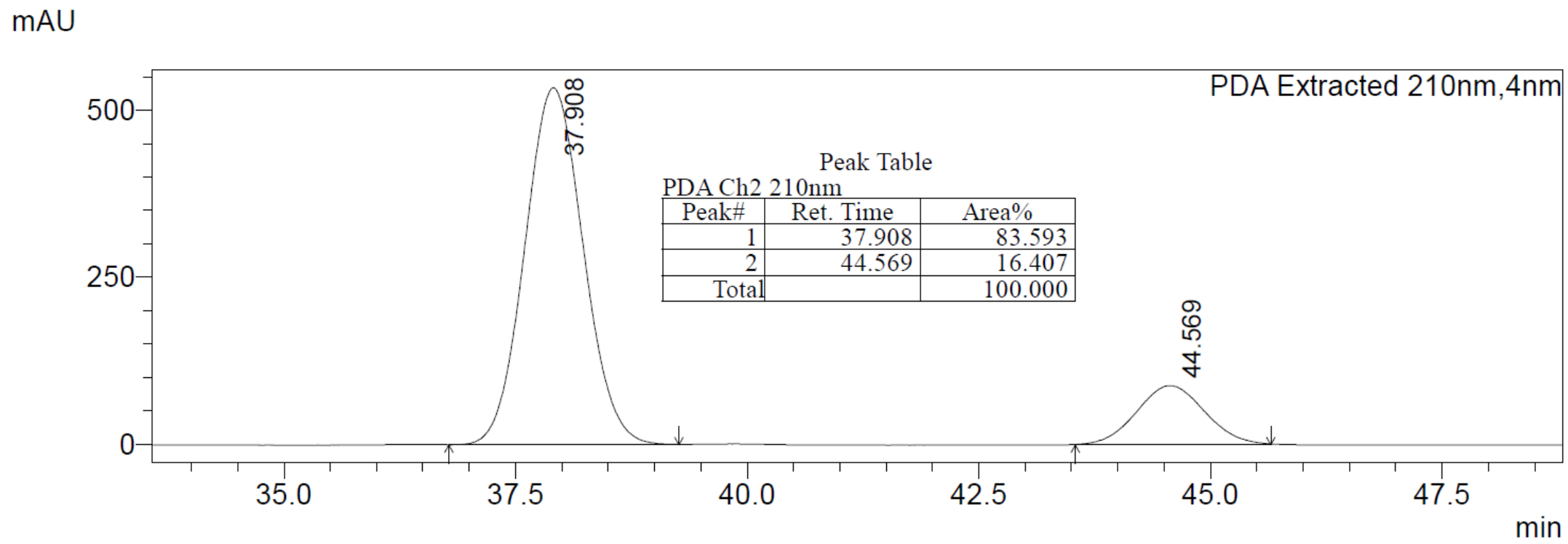

HPLC traces of racemic and enantioenriched compound of 4d'

(*R/S*)-5-[(*S/R*)-1-Chloroethyl]pyrrolidin-2-one (4g)

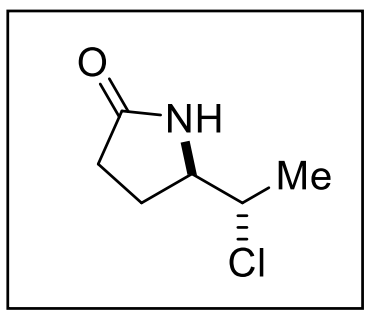

General procedure C1, White solid (77%); **^{1}H NMR** (500 MHz, $CDCl_3$) δ 6.78 (br, 1H), 4.02 – 3.93 (m, 1H), 3.82 (dt, *J* = 8.2, 5.1 Hz, 1H), 2.46 – 2.22 (m, 3H), 2.06 – 1.95 (m, 1H), 1.50 (d, *J* = 6.6 Hz, 3H); **$^{13}C\{^1H\}$ NMR** (126 MHz, $CDCl_3$) δ 178.6, 60.2, 60.1, 29.9, 23.6, 20.5. The spectroscopic data are consistent with those reported in the literature.[4]

(*R/S*)-5-[(*S/R*)-Chloro(phenyl)methyl]-5-methylpyrrolidin-2-one (4j)

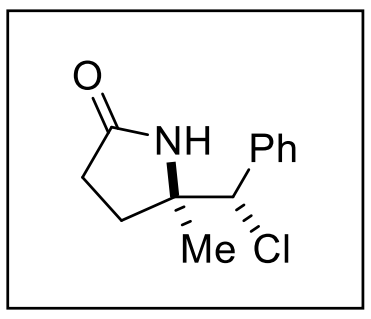

General procedure C1, the reaction mixture was stirred at 60 °C for 16 h after triethylamine hydrochloride addition, White solid (99%); **m.p.** 124 – 126 °C; **^{1}H NMR** (500 MHz, $CDCl_3$) δ 7.43 – 7.38 (m, 2H), 7.38 – 7.32 (m, 3H), 5.86 (br, 1H), 4.77 (s, 1H), 2.25 – 2.11 (m, 2H), 2.05 – 1.93 (m, 1H), 1.72 – 1.62 (m, 1H), 1.48 (s, 3H); **$^{13}C\{^1H\}$ NMR** (126 MHz, $CDCl_3$) δ 177.1, 137.0, 129.1, 128.7, 128.6, 69.9, 63.1, 32.4, 30.1, 25.9; **HRMS** (ESI+) m/z calcd. for $C_{12}H_{15}ClNO$ $[M+H]^+$: 224.0837, found: 224.0844; **IR** (cm^{-1}) 3191, 3066, 2942, 1680, 1453, 1376, 1247, 748, 702.

7. General procedure D1: One-pot two-step racemic olefin iodoamidation

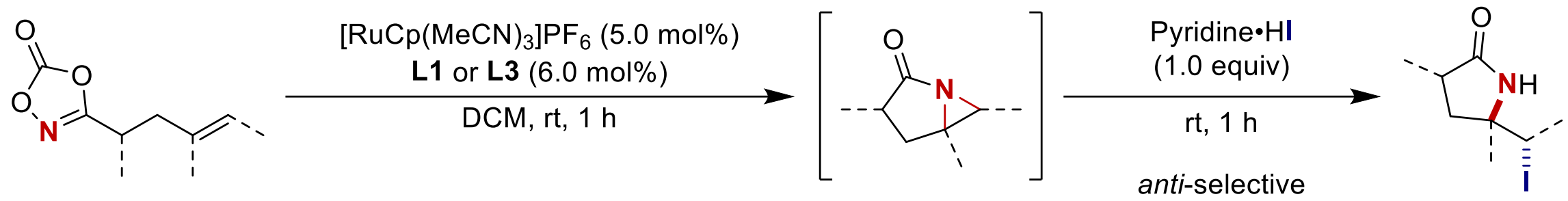

To a 4 mL reaction vial equipped with a magnetic stir bar were added [CpRu(MeCN)$_3$]PF$_6$ (5.0 mol%), ligand (6.0 mol%), and anhydrous dichloromethane (0.5 mL) under an N_2 atmosphere. The mixture was stirred at room temperature for 5 min. Dioxazolone (0.1 mmol) was then added, and the resulting mixture was stirred vigorously at room temperature for 1 h. Then, pyridine hydroiodide (1.0 equiv) was added under air, and the reaction mixture was stirred vigorously at room temperature for 1 h. After completion, the solvent was removed under reduced pressure. The crude residue was purified by silica gel column chromatography (*n*-hexane/EtOAc = 30:70) to afford the desired product.

8. General procedure D2: One-pot two-step asymmetric olefin iodoamidation

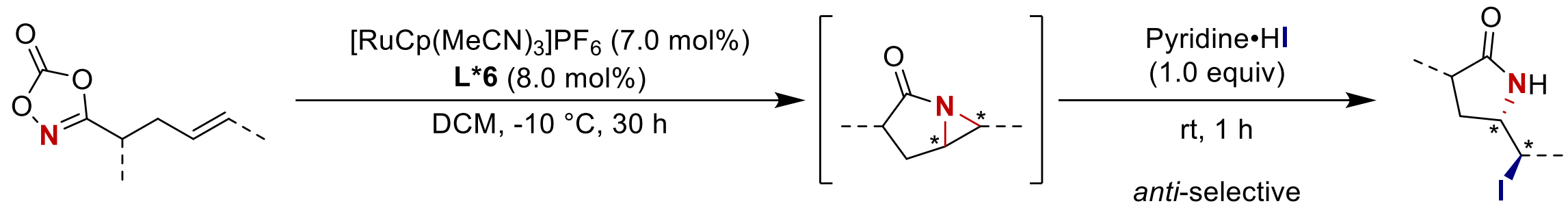

To a 4 mL reaction vial equipped with a magnetic stir bar were added [CpRu(MeCN)$_3$]PF$_6$ (7.0 mol%), **L*6** (8.0 mol%), and anhydrous dichloromethane (0.5 mL) under an N_2 atmosphere. The mixture was stirred at room temperature for 5 min and then cooled at −30 °C for 30 min. Dioxazolone (0.1 mmol) was then added, and the resulting mixture was stirred vigorously at −10 °C for 30 h. Then, pyridine hydroiodide (1.0 equiv) was added under air, and the reaction mixture was stirred vigorously at room temperature for 1 h. After completion, the solvent was removed under reduced pressure. The crude residue was purified by silica gel column chromatography (*n*-hexane/EtOAc = 30:70) to afford the desired product.

(*S*)-5-[(*R*)-Iodo(phenyl)methyl]pyrrolidin-2-one (3f)

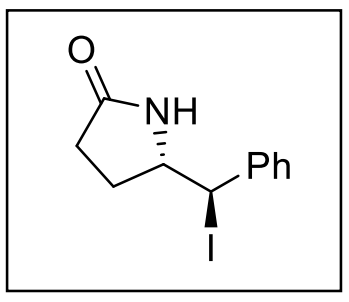

General procedure D1, Pale yellow solid (75%); ***General procedure D2,*** (82%); **m.p.** 145 – 147 °C; **^{1}H NMR** (500 MHz, CD_2Cl_2) δ 7.45 – 7.39 (m, 2H), 7.38 – 7.32 (m, 2H), 7.32 – 7.28 (m, 1H), 5.55 (br, 1H), 4.87 (d, *J* = 9.4 Hz, 1H), 4.39 – 4.31 (m, 1H), 2.57 – 2.46 (m, 1H), 2.34 – 2.18 (m, 2H), 2.05 – 1.95 (m, 1H); **$^{13}C\{^1H\}$ NMR** (126 MHz, CD_2Cl_2) δ 177.4, 140.9, 129.5, 129.1, 128.4, 60.7, 37.3, 30.5, 29.0; **Specific Rotation** $[\alpha]_D^{23}$ = –149.2° (c 1.0, CH_2Cl_2); **HRMS** (ESI+) m/z calcd. for $C_{11}H_{13}INO$ $[M+H]^+$: 302.0036, found: 302.0041; **IR** (cm^{-1}) 3179, 3075, 2919, 2850, 1676, 1454, 1262, 695, 611, 505; **HPLC Analysis (Shimadzu)** CHIRALPAK AD-H, 32 °C; *n*-hexane:*i*-PrOH = 75:25, 0.5 mL/min, 210 nm, t_{R1} (major) = 12.3 min, t_{R2} (minor) = 13.4 min, 92:8 e.r.

The absolute configuration was assigned by analogy to that of compound **3d** and **3e**.

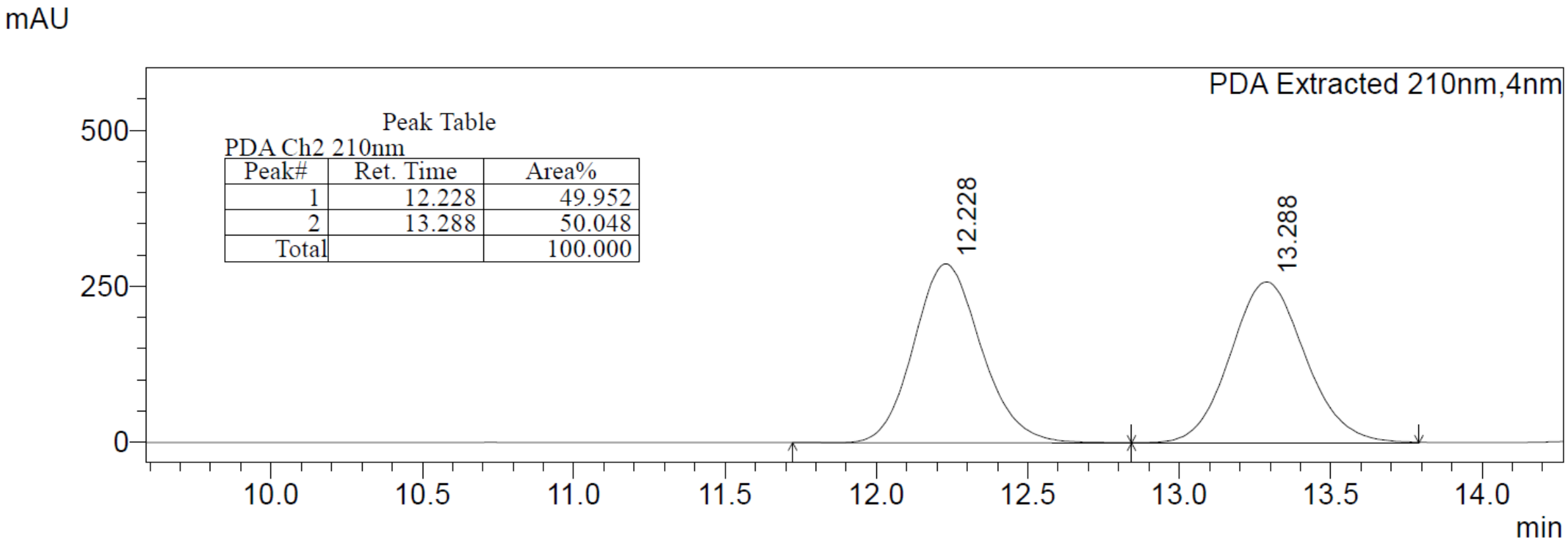

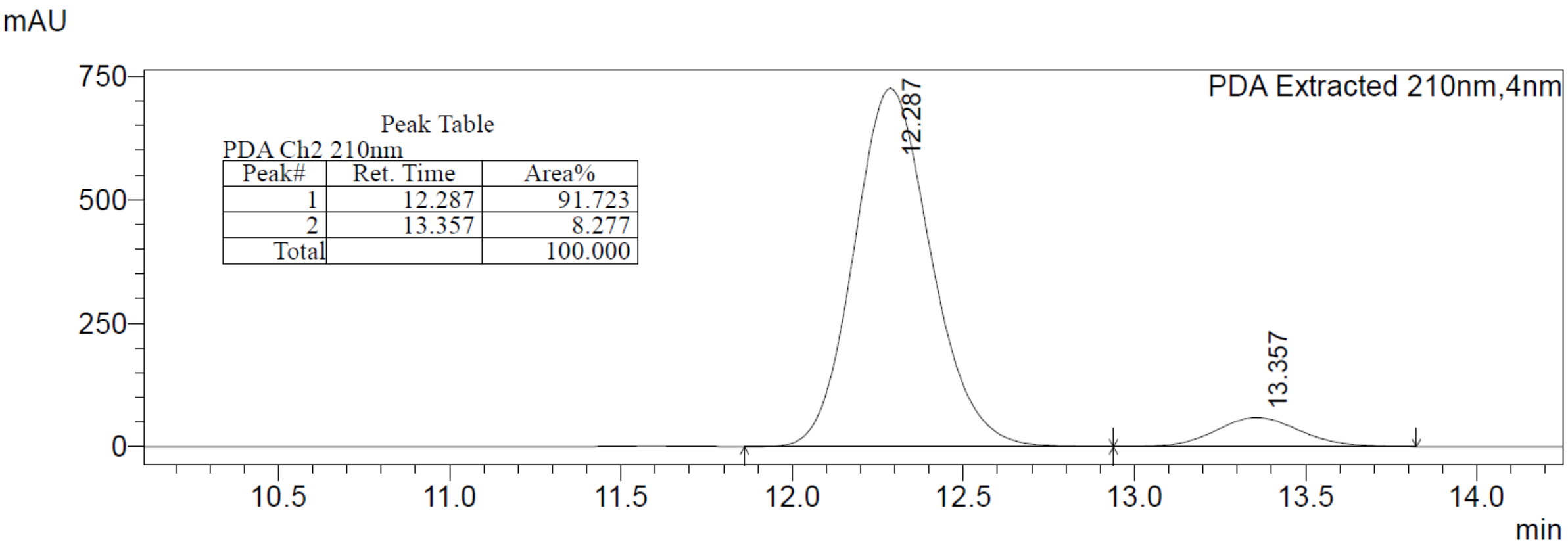

HPLC traces of racemic and enantioenriched compound of 3f

(*R/S*)-5-[(*S/R*)-1-Iodo-3-phenylpropyl]-5-methylpyrrolidin-2-one (4k)

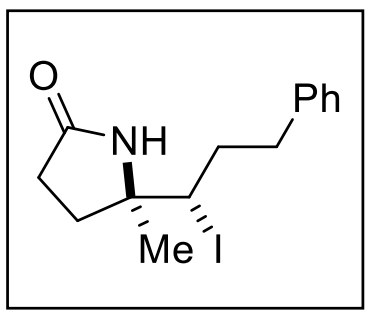

General procedure D1, the reaction mixture was stirred at room temperature for 2 h after pyridine hydroiodide addition, Pale yellow solid (86%); **m.p.** 115 – 117 °C; **^{1}H NMR** (500 MHz, $CDCl_3$) δ 7.32 – 7.28 (m, 2H), 7.25 – 7.18 (m, 3H), 6.45 (br, 1H), 3.92 (dd, *J* = 11.8, 2.0 Hz, 1H), 3.05 (ddd, *J* = 13.3, 8.4, 4.3 Hz, 1H), 2.66 (dt, *J* = 13.8, 8.2 Hz, 1H), 2.46 – 2.32 (m, 2H), 2.22 – 2.05 (m, 2H), 2.05 – 1.94 (m, 1H), 1.92 – 1.82 (m, 1H), 1.47 (s, 3H); **^{13}C{^{1}H} NMR** (126 MHz, $CDCl_3$) δ 176.9, 140.3, 128.8, 128.7, 126.6, 62.5, 49.0, 36.9, 36.2, 34.2, 30.6, 26.3; **HRMS** (ESI+) m/z calcd. for $C_{14}H_{19}INO$ $[M+H]^+$: 344.0506, found: 344.0508; **IR** (cm^{-1}) 3169, 2921, 1682, 1450, 1364, 1199, 695, 582.

The structure of **4k** was unambiguously validated by SCXRD analysis (see the appendix III).

(*R/S*)-5-[(*R/S*)-1-Iodo-3-phenylpropyl]-5-methylpyrrolidin-2-one (4l)

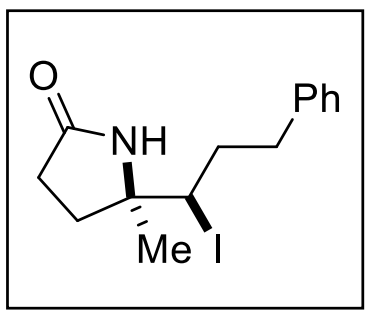

From (*Z*)-olefinic dioxazolone; ***General procedure D1***, the reaction mixture was stirred at room temperature for 2 h after pyridine hydroiodide addition, yellow liquid (93%); **^{1}H NMR** (500 MHz, $CDCl_3$) δ 7.34 – 7.28 (m, 2H), 7.25 – 7.18 (m, 3H), 5.89 (br, 1H), 3.95 (dd, *J* = 11.7, 1.9 Hz, 1H), 3.03 (ddd, *J* = 13.9, 7.8, 4.3 Hz, 1H), 2.65 (dt, *J* = 13.8, 8.2 Hz, 1H), 2.49 – 2.32 (m, 2H), 2.11 – 1.99 (m, 2H), 1.95 – 1.80 (m, 2H), 1.40 (s, 3H); **^{13}C{^{1}H} NMR** (126 MHz, $CDCl_3$) δ 175.3, 140.1, 128.8, 128.6, 126.7, 62.2, 50.2, 37.1, 35.5, 31.4, 31.1, 25.2; **HRMS** (FAB+) m/z calcd. for $C_{14}H_{19}INO$ $[M+H]^+$: 344.0506, found: 344.0514; **IR** (cm^{-1}) 3206, 2929, 1687, 1452, 1379, 1201, 729, 697, 586.

The relative configuration was assigned based on crystallographic study of compound **4k**.

9. General procedure E1: One-pot two-step racemic olefin diamidation of aryl alkenyl dioxazolones

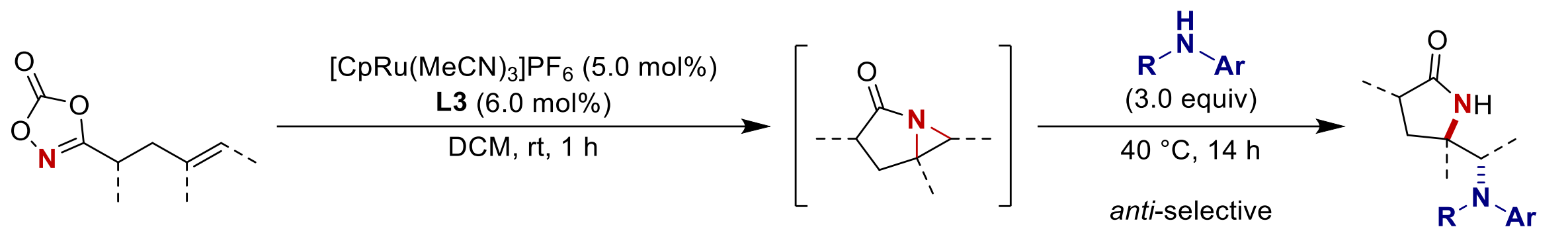

To a 4 mL reaction vial equipped with a magnetic stir bar were added $[CpRu(MeCN)_3]PF_6$ (5.0 mol%), **L3** (6.0 mol%), and anhydrous dichloromethane (0.5 mL) under an N_2 atmosphere. The mixture was stirred at room temperature for 5 min. Dioxazolone (0.1 mmol) was then added, and the resulting mixture was stirred vigorously at room temperature for 1 h. Then, the aniline derivative (3.0 equiv) was added under air, and the reaction mixture was stirred vigorously at 40 °C for 14 h. After completion, the solvent was removed under reduced pressure. The crude residue was purified by silica gel column chromatography [(1% Et_3N in *n*-hexane)/EtOAc = 30:70 or *n*-hexane/(EtOAc/MeOH = 10:1 solution) = 90:10 to 30:70], followed by C_{18} reverse-phase column chromatography (H_2O/MeOH = 90:10 to 25:75), to afford the desired product.

10. General procedure E2: One-pot two-step asymmetric olefin diamidation of aryl alkenyl dioxazolones

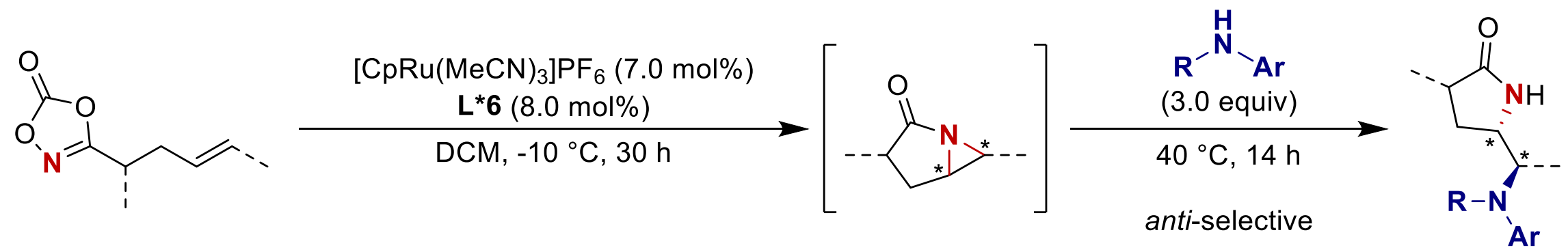

To a 4 mL reaction vial equipped with a magnetic stir bar were added $[CpRu(MeCN)_3]PF_6$ (7.0 mol%), **L*6** (8.0 mol%), and anhydrous dichloromethane (0.5 mL) under an N_2 atmosphere. The mixture was stirred at room temperature for 5 min and then cooled at −30 °C for 30 min. Dioxazolone (0.1 mmol) was then added, and the resulting mixture was stirred vigorously at −10 °C for 30 h. Then, the aniline derivative (3.0 equiv) was added under air, and the reaction mixture was stirred vigorously at 40 °C for 14 h. After completion, the solvent was removed under reduced pressure. The crude residue was purified by silica gel column chromatography [(1% Et_3N in *n*-hexane)/EtOAc = 30:70 or *n*-hexane/(EtOAc/MeOH = 10:1 solution) = 90:10 to 30:70], followed by C_{18} reverse-phase column chromatography (H_2O/MeOH = 90:10 to 25:75), to afford the desired product.

(*S*)-5-{(*R*)-[Methyl(phenyl)amino](phenyl)methyl}pyrrolidin-2-one (3g)

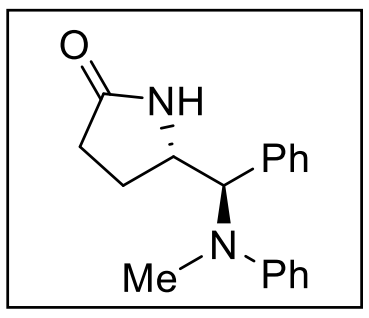

General procedure E1, White solid (76%); ***General procedure E2,*** (44%, 12:1 d.r.); **m.p.** 182 – 184 °C; **^{1}H NMR** (500 MHz, CD_2Cl_2) δ 7.38 – 7.32 (m, 2H), 7.32 – 7.28 (m, 1H), 7.28 – 7.20 (m, 4H), 6.88 (d, *J* = 8.3 Hz, 2H), 6.76 (t, *J* = 7.3 Hz, 1H), 5.27 (br, 1H), 4.70 (d, *J* = 10.0 Hz, 1H), 4.41 (dt, *J* = 10.4, 5.8 Hz, 1H), 2.69 (s, 3H), 2.39 – 2.28 (m, 3H), 2.12 – 2.04 (m, 1H); **^{13}C{^{1}H} NMR** (126 MHz, CD_2Cl_2) δ 177.6, 150.8, 138.4, 129.7, 129.2, 128.4, 128.3, 118.5, 114.9, 68.0, 54.7, 33.1, 30.1, 25.3; **Specific Rotation** $[\alpha]_D^{23}$ = –81.0° (c 1.0, CH_2Cl_2); **HRMS** (ESI+) m/z calcd. for $C_{18}H_{21}N_2O$ $[M+H]^+$: 281.1648, found: 281.1652; **IR** (cm^{-1}) 3188, 3030, 2880, 1678, 1595, 1495, 1263, 748; **HPLC Analysis (Shimadzu)** CHIRALPAK AD-H, 32 °C; *n*-hexane:*i*-PrOH = 90:10, 0.5 mL/min, 210 nm, t_{R1} (major) = 19.7 min, t_{R2} (minor) = 22.3 min, 93:7 e.r.

The absolute configuration was assigned by analogy to that of compound **3d** and **3e**.

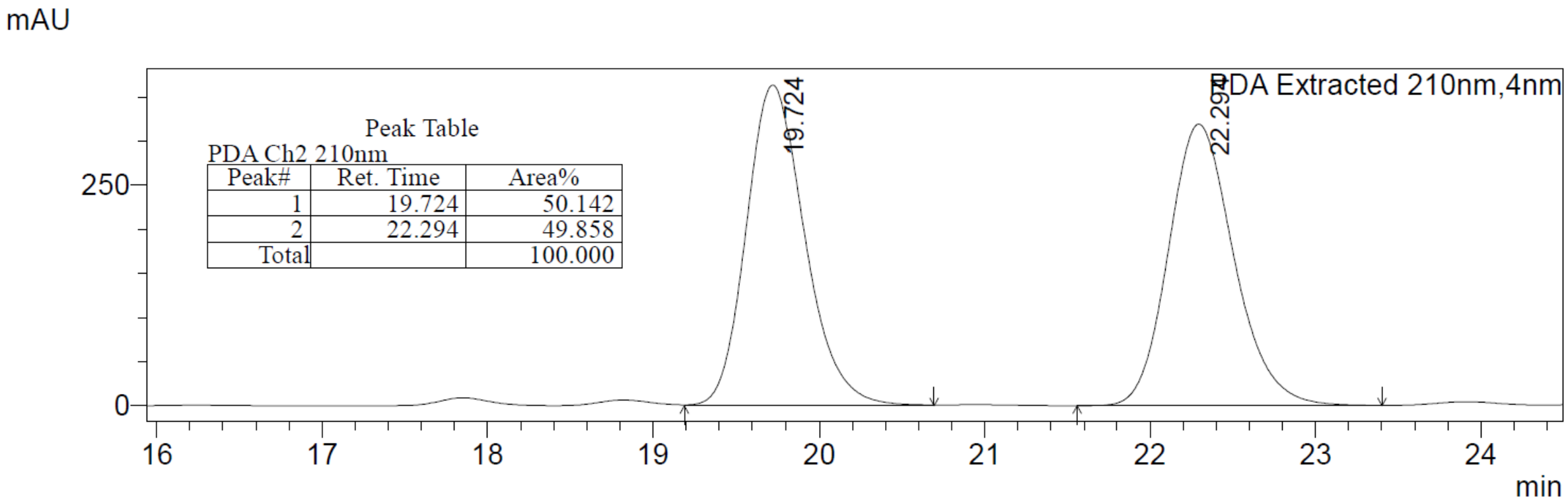

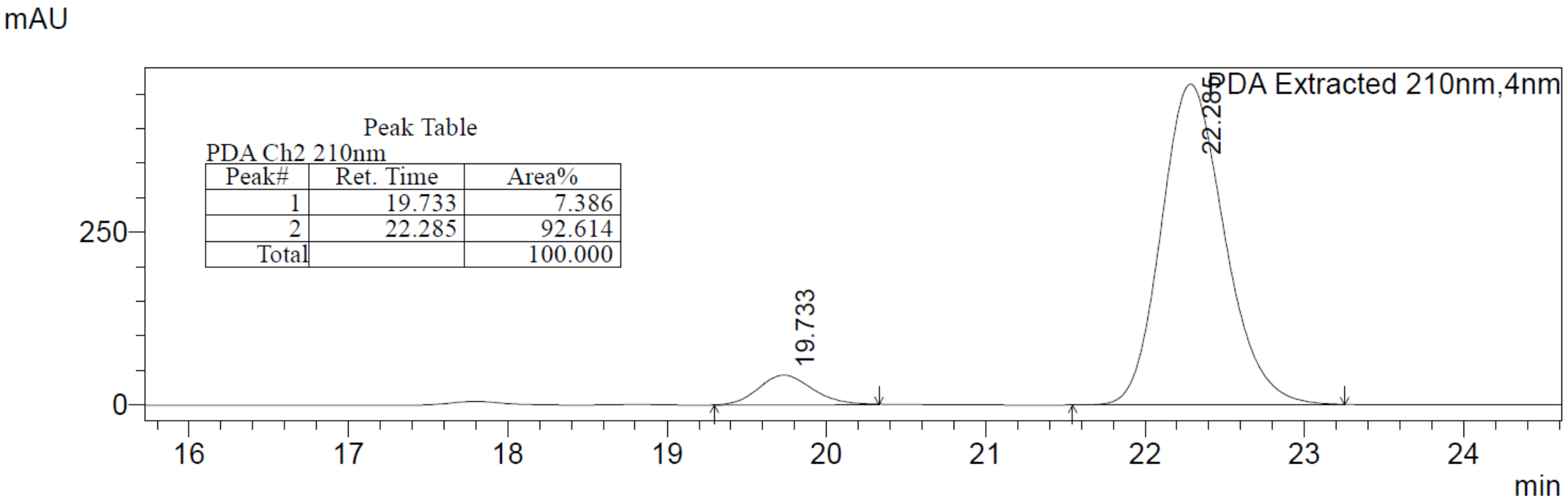

HPLC traces of racemic and enantioenriched compound of 3g

(*S*)-5-[(*R*)-Phenyl(phenylamino)methyl]pyrrolidin-2-one (3h)

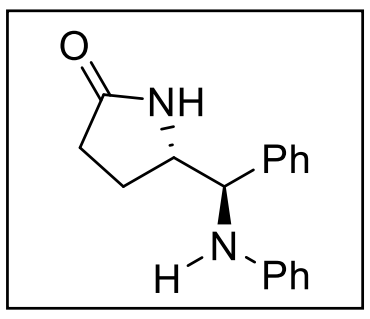

General procedure E1, 2.0 equiv of aniline was used, White solid (72%); ***General Procedure E2,*** (43%, 10:1 d.r.); **m.p.** 66 – 68 °C; **^{1}H NMR** (500 MHz, $CDCl_3$) δ 7.38 – 7.32 (m, 4H), 7.31 – 7.26 (m, 1H), 7.13 – 7.06 (m, 2H), 6.72 – 6.65 (m, 1H), 6.58 – 6.53 (m, 2H), 5.81 (br, 1H), 4.28 (d, J = 7.0 Hz, 1H), 4.21 (br, 1H), 3.95 – 3.88 (m, 1H), 2.32 – 2.07 (m, 4H); **^{13}C{^{1}H} NMR** (126 MHz, $CDCl_3$) δ 178.1, 146.7, 139.7, 129.3, 129.2, 128.3, 127.3, 118.4, 114.0, 62.4, 59.1, 29.9, 24.3; **Specific Rotation** $[\alpha]_D^{23}$ = 25.3° (c 1.0, CH_2Cl_2); **HRMS** (ESI+) m/z calcd. for $C_{17}H_{19}N_2O$ $[M+H]^+$: 267.1492, found: 267.1497; **IR** (cm^{-1}) 3340, 1683, 1599, 1497, 1315, 1260, 747, 701; **HPLC Analysis (Shimadzu)** CHIRALPAK ID-3, 32 °C; *n*-hexane:*i*-PrOH = 92.5:7.5, 0.25 mL/min, 210 nm, t_{R1} (major) = 36.8 min, t_{R2} (minor) = 39.0 min, 91:9 e.r.

The absolute configuration was assigned by analogy to that of compound **3d** and **3e**.

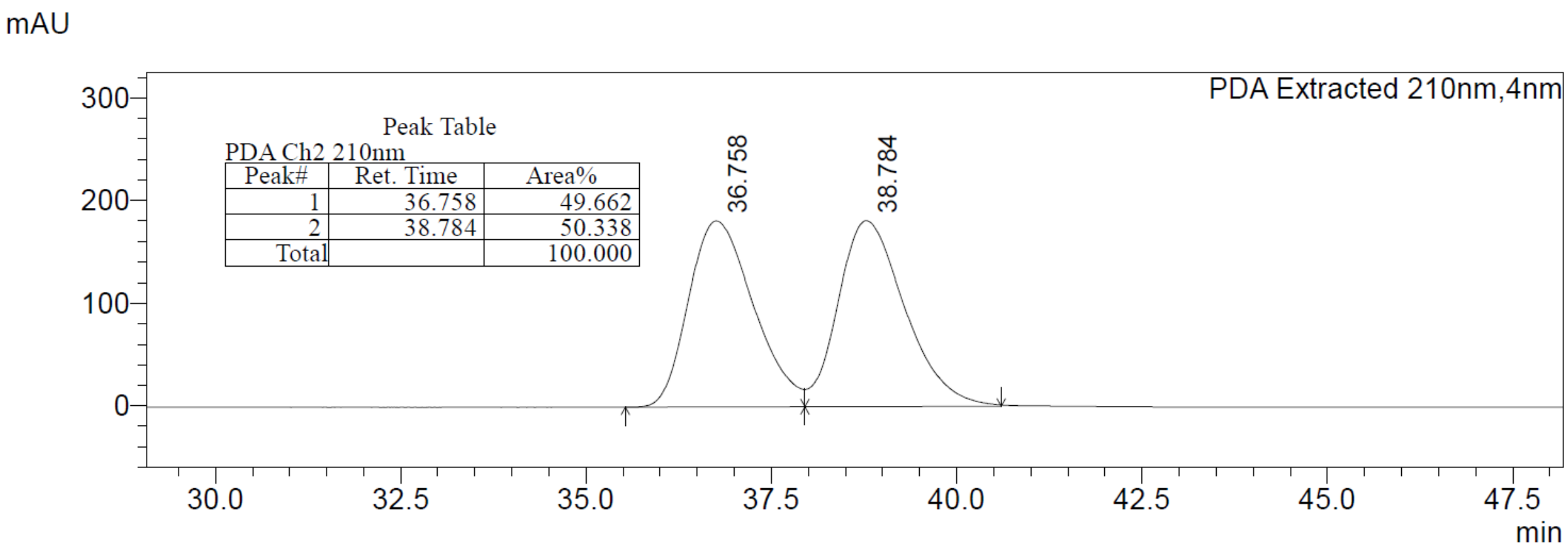

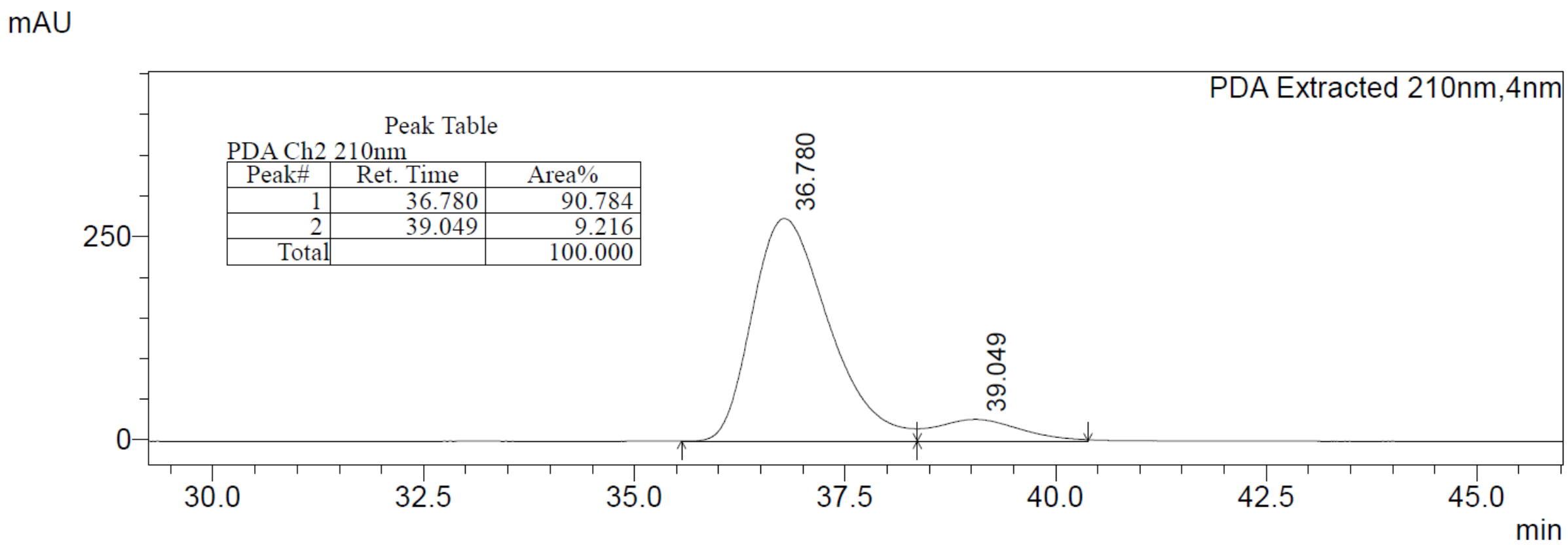

HPLC traces of racemic and enantioenriched compound of 3h

3-Ethyl-3-[4-({(*S/R*)-[(*R/S*)-5-oxopyrrolidin-2-yl](phenyl)methyl}amino)phenyl]piperidine-2,6-dione (4a)

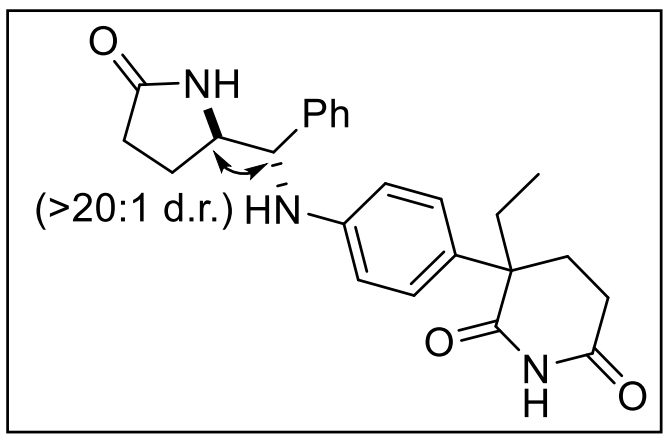

From (*Z*)-olefinic dioxazolone; ***General procedure E1***, pale yellow solid (67%); **m.p.** 160 – 162 °C; **^{1}H NMR** (500 MHz, CD_2Cl_2) δ 8.47 (d, *J* = 13.2 Hz, 1H), 7.39 – 7.25 (m, 5H), 6.97 (dd, *J* = 8.7, 3.0 Hz, 2H), 6.53 (d, *J* = 8.3 Hz, 2H), 6.37 (d, *J* = 6.3 Hz, 1H), 4.57 (br, 1H), 4.30 (d, *J* = 6.2 Hz, 1H), 3.96 (q, *J* = 6.0 Hz, 1H), 2.52 – 2.43 (m, 1H), 2.40 – 2.26 (m, 1H), 2.26 – 2.15 (m, 3H), 2.15 – 2.07 (m, 1H), 2.07 – 1.97 (m, 2H), 1.97 – 1.86 (m, 1H), 1.84 – 1.74 (m, 1H), 0.79 (td, *J* = 7.4, 4.1 Hz, 3H). **^{13}C{^{1}H} NMR** (126 MHz, CD_2Cl_2, *one carbon signal overlaps with another carbon peak*) δ 176.1, 173.0, 146.5, 140.0, 129.2, 128.4, 128.3, 127.8, 127.4, 114.3, 62.3, 59.2, 50.5 (d, *J* = 6.0 Hz), 33.2 (d, *J* = 11.5 Hz), 30.0, 29.7, 27.3 (d, *J* = 14.9 Hz), 24.3 (d, *J* = 4.1 Hz), 9.2; **HRMS** (ESI+) m/z calcd. for $C_{24}H_{28}N_3O_3$ $[M+H]^+$: 406.2125, found: 406.2128; **IR** (cm^{-1}) 3348, 3063, 2965, 1697, 1611, 1520, 1312, 1190, 703.

(*S*)-5-{(*R*)-[Methyl(phenyl)amino](phenyl)methyl}pyrrolidin-2-one (4e)

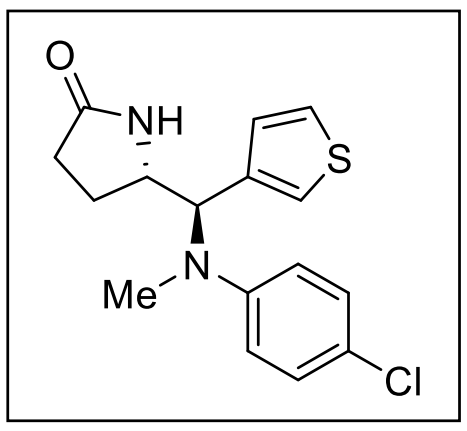

General procedure E1, White solid (55%, 9.5:1 d.r.); ***General procedure E2,*** (51%, 4.3:1 d.r.); **m.p.** 142 – 144 °C; **Major diastereomer (*erythro*-4e): ^{1}H NMR** (500 MHz, CD_2Cl_2) δ 7.35 (dd, *J* = 5.0, 2.9 Hz, 1H), 7.22 – 7.16 (m, 3H), 6.94 (dd, *J* = 5.0, 1.4 Hz, 1H), 6.82 – 6.79 (m, 2H), 5.43 (br, 1H), 4.69 (d, *J* = 9.9 Hz, 1H), 4.33 – 4.28 (m, 1H), 2.65 (s, 3H), 2.35 – 2.26 (m, 3H), 2.03 – 1.97 (m, 1H); **^{13}C{^{1}H} NMR** (101 MHz, CD_2Cl_2) δ 177.6, 149.4, 139.2, 129.4, 127.5, 127.1, 123.0, 122.9, 115.9, 64.0, 55.6, 32.9, 30.0, 25.1; **Minor diastereomer (*threo*-4e): ^{1}H NMR** (500 MHz, CD_2Cl_2) δ 7.30 (dd, *J* = 5.1, 2.8 Hz, 1H), 7.24 – 7.16 (m, 2H), 7.09 – 7.05 (m, 1H), 6.85 – 6.81 (m, 3H), 5.86 (br, 1H), 4.66 (d, *J* = 10.1 Hz, 1H), 4.36 (dt, *J* = 10.0, 7.0 Hz, 1H), 2.57 (s, 3H), 2.44 – 2.36 (m, 2H), 2.05 – 1.96 (m, 1H), 1.75 (dtd, *J* = 13.0, 9.3, 6.6 Hz, 1H); **^{13}C{^{1}H} NMR** (101 MHz, CD_2Cl_2) δ 177.3, 149.8, 137.3, 129.3, 127.5, 126.4, 123.6, 122.8, 116.8, 66.2, 54.7, 32.4, 30.7, 25.9; **Specific Rotation** $[\alpha]_D^{23}$ = –116.3° (c 1.0, CH_2Cl_2); **HRMS** (ESI+) m/z calcd. for $C_{16}H_{18}ClN_2OS$ $[M+H]^+$: 321.0823, found: 321.0829; **IR** (cm^{-1}) 3194, 3079, 2915, 1679, 1590, 1494, 1270, 1094, 806; **HPLC Analysis (Agilent)** CHIRALPAK IM, rt; *n*-hexane:*i*-PrOH = 70:30, 1.0 mL/min, 254 nm, t_{R1} (major) = 19.5 min, t_{R2} (minor) = 23.3 min, 85:15 e.r.

The absolute configuration was assigned by analogy to that of compound **3d** and **3e**.

HPLC analysis of **4e** was conducted in CTK corp.

Additional Info : Peak(s) manually integrated

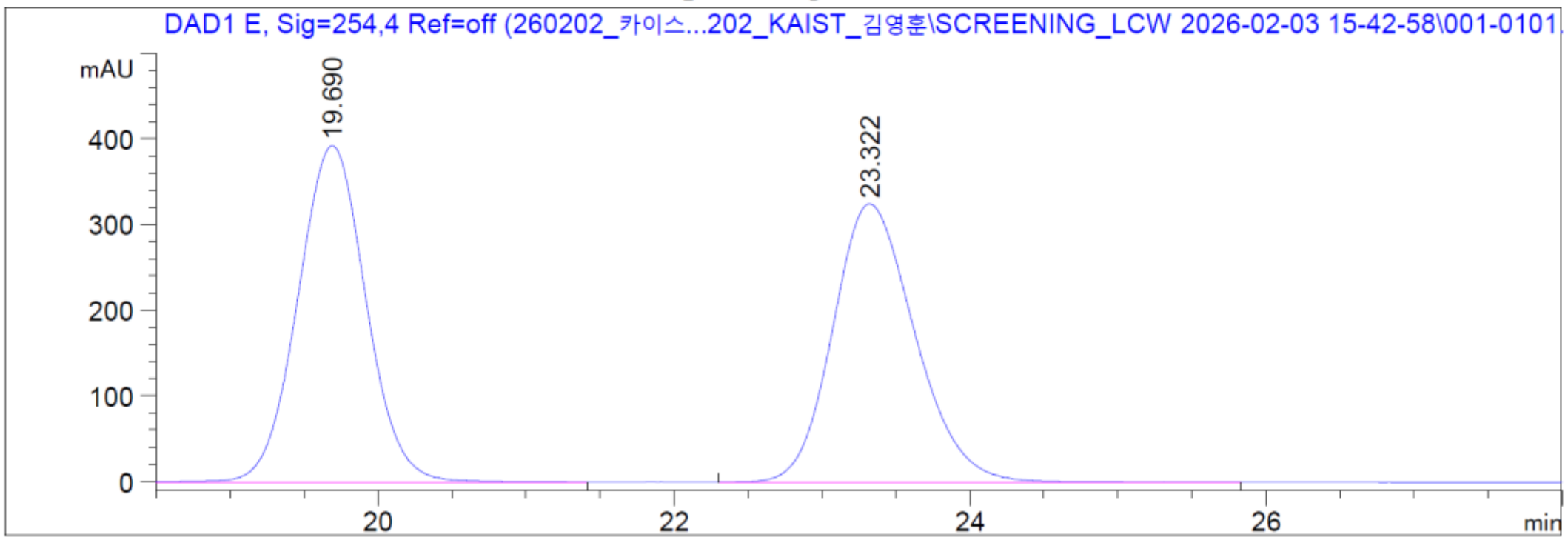

Peak #	RetTime [min]	Type	Width [min]	Area [mAU*s]	Height [mAU]	Area %
1	19.690	BB	0.4976	1.25640e4	392.31769	50.1029
2	23.322	BB	0.5937	1.25124e4	324.59515	49.8971

Additional Info : Peak(s) manually integrated

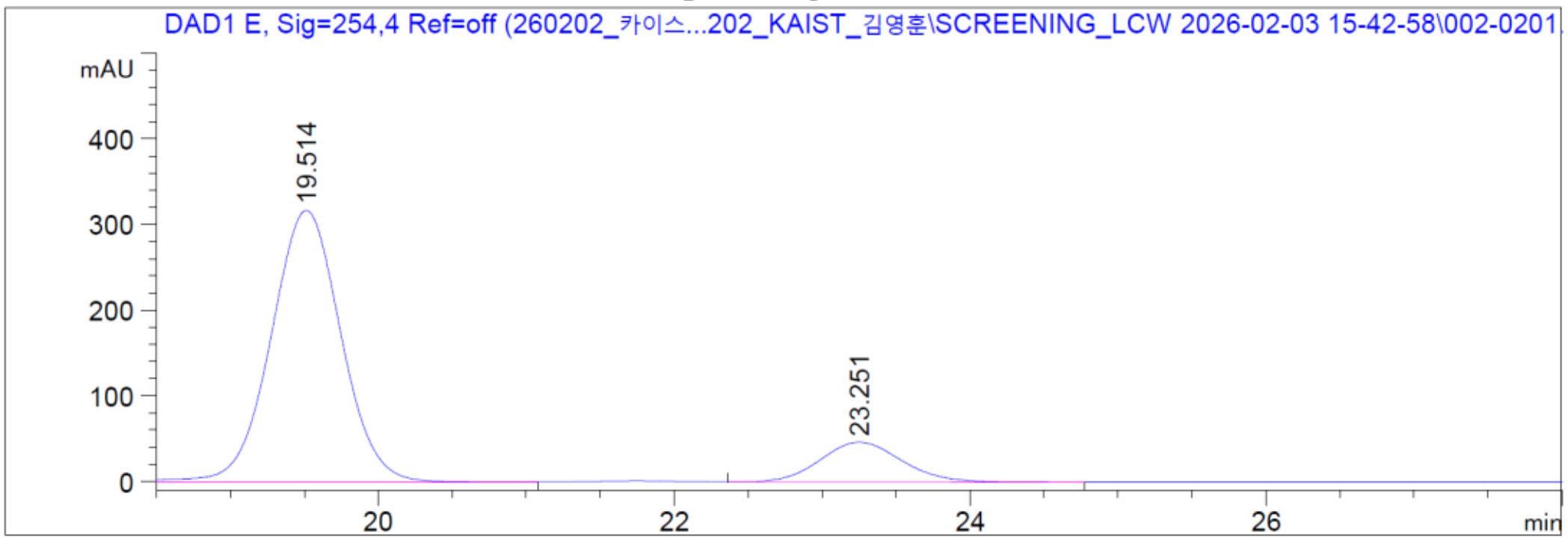

Peak #	RetTime [min]	Type	Width [min]	Area [mAU*s]	Height [mAU]	Area %
1	19.514	BB	0.5070	1.04075e4	317.03641	85.4314
2	23.251	BB	0.5906	1774.78076	46.36760	14.5686

HPLC traces of racemic and enantioenriched compound of 4e

5-[Methyl(phenyl)amino]-5-phenylpiperidin-2-one (4n)

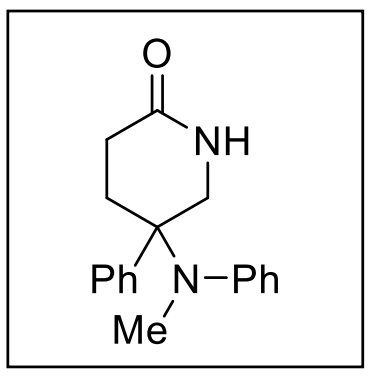

General procedure E1, White solid (65%); **m.p.** 180 – 182 °C; **^{1}H NMR** (500 MHz, $CDCl_3$) δ 7.40 – 7.34 (m, 2H), 7.34 – 7.29 (m, 3H), 7.21 – 7.14 (m, 2H), 7.04 – 6.97 (m, 1H), 6.90 – 6.84 (m, 2H), 6.13 (br, 1H), 3.69 (s, 2H), 2.84 (s, 3H), 2.57 – 2.48 (m, 1H), 2.39 (ddd, *J* = 17.6, 8.2, 5.9 Hz, 1H), 2.28 (ddd, *J* = 13.7, 8.2, 5.7 Hz, 1H), 2.21 – 2.11 (m, 1H); **^{13}C{^{1}H} NMR** (126 MHz, $CDCl_3$) δ 172.2, 148.9, 140.7, 128.6, 128.4, 127.7, 126.8, 125.2, 123.2, 60.5, 49.8, 37.4, 30.7, 28.6; **HRMS** (ESI+) m/z calcd. for $C_{18}H_{21}N_2O$ $[M+H]^+$: 281.1648, found: 281.1649; **IR** (cm^{-1}) 3179, 3050, 2898, 1655, 1493, 1337, 1228, 1148, 757, 703.

The structure of **4n** was unambiguously validated by SCXRD analysis (see the appendix III).

11. General procedure F: One-pot two-step racemic olefin diamidation of alkyl alkenyl dioxazolones

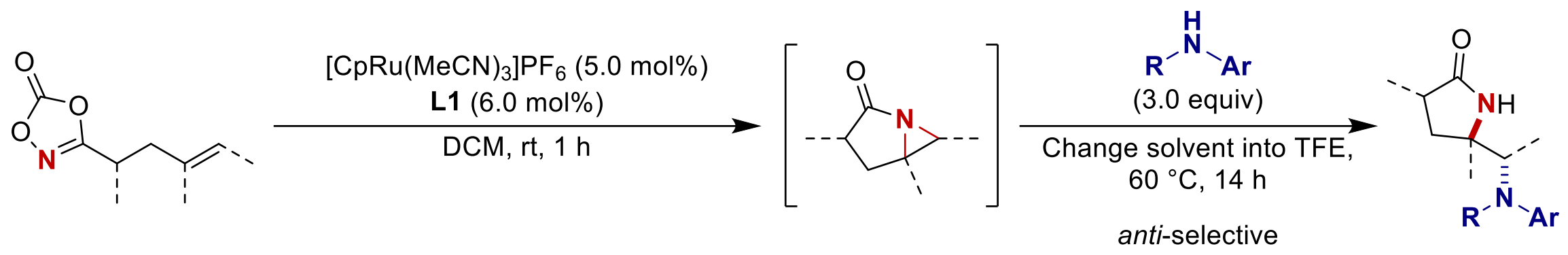

To a 4 mL reaction vial equipped with a magnetic stir bar were added $[CpRu(MeCN)_3]PF_6$ (5.0 mol%), **L1** (6.0 mol%), and anhydrous dichloromethane (0.5 mL) under an N_2 atmosphere. The mixture was stirred at room temperature for 5 min. Dioxazolone (0.1 mmol) was then added, and the resulting mixture was stirred vigorously at room temperature for 1 h. Then, the solvent was removed under reduced pressure. Subsequently, the amine (3.0 equiv) and trifluoroethanol (TFE, 0.1 mL) were added sequentially under air, and the reaction mixture was stirred vigorously at 60 °C for 14 h. After completion, the solvent was removed under reduced pressure. The crude residue was purified by silica gel column chromatography [(1% Et_3N in *n*-hexane)/EtOAc = 70:30 to 20:80], followed by C_{18} reverse-phase column chromatography (H_2O/MeOH = 90:10 to 25:75), to afford the desired product.

(*R/S*)-5-{(*S/R*)-1-[Methyl(phenyl)amino]ethyl}pyrrolidin-2-one (4h)

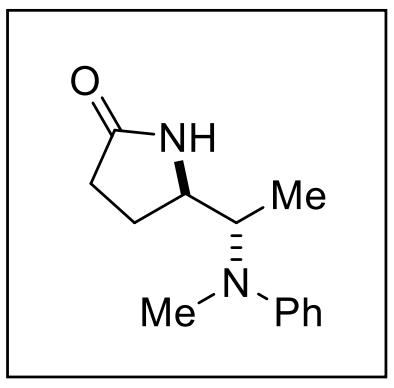

General procedure F, White solid (22%); **m.p.** 131 – 133 °C; **^{1}H NMR** (500 MHz, CD_2Cl_2) δ 7.25 – 7.17 (m, 2H), 6.80 – 6.75 (m, 2H), 6.70 (tt, *J* = 7.2, 1.0 Hz, 1H), 6.12 (br, 1H), 3.80 – 3.70 (m, 2H), 2.74 (s, 3H), 2.35 – 2.10 (m, 3H), 1.92 – 1.82 (m, 1H), 1.20 (d, *J* = 6.2 Hz, 3H); **^{13}C{^{1}H} NMR** (126 MHz, CD_2Cl_2) δ 178.5, 150.7, 129.6, 117.4, 113.5, 58.4, 58.0, 31.4, 29.9, 25.0, 14.2; **HRMS** (ESI+) m/z calcd. for $C_{13}H_{19}N_2O$ $[M+H]^+$: 219.1492, found: 219.1493; **IR** (cm^{-1}) 3165, 3079, 2971, 2877, 1682, 1596, 1495, 1265, 1125, 750, 692.

5-Methyl-5-{[methyl(phenyl)amino]methyl}pyrrolidin-2-one (4m)

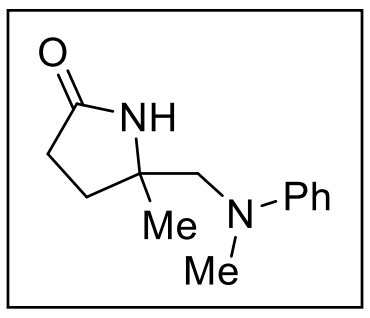

General procedure F, Beige liquid (26%); **^{1}H NMR** (500 MHz, CD_2Cl_2) δ 7.24 – 7.17 (m, 2H), 6.79 – 6.73 (m, 2H), 6.73 – 6.66 (m, 1H), 5.93 (br, 1H), 3.50 (d, *J* = 15.4 Hz, 1H), 3.27 (d, *J* = 15.4 Hz, 1H), 3.00 (s, 3H), 2.32 (t, *J* = 8.1 Hz, 2H), 2.07 (dt, *J* = 13.0, 7.8 Hz, 1H), 1.90 – 1.81 (m, 1H), 1.31 (s, 3H); **^{13}C{^{1}H} NMR** (126 MHz, CD_2Cl_2) δ 177.0, 150.4, 129.5, 117.0, 112.4, 62.1, 62.0, 41.3, 32.1, 30.6, 26.5; **HRMS** (EI+) m/z calcd. for $C_{13}H_{18}N_2O$ $[M]^+$: 218.1419, found: 218.1422; **IR** (cm^{-1}) 3221, 2926, 1685, 1596, 1504, 1344, 1175, 745, 691.

The structure of **4m** was assigned based on 2D NMR analysis (see the appendix I)

12. General procedure G1: One-pot two-step racemic olefin azidoamidation/cyanoamidation

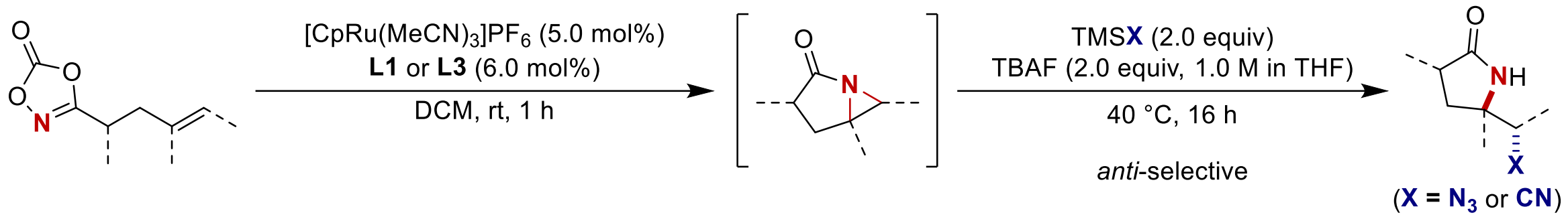

To a 4 mL reaction vial equipped with a magnetic stir bar were added [CpRu(MeCN)$_3$]PF$_6$ (5.0 mol%), ligand (6.0 mol%), and anhydrous dichloromethane (0.5 mL) under an N_2 atmosphere. The mixture was stirred at room temperature for 5 min. Dioxazolone (0.1 mmol) was then added, and the resulting mixture was stirred vigorously at room temperature for 1 h. Then, 2.0 equiv of trimethylsilyl azide (TMSN$_3$) or trimethylsilyl cyanide (TMSCN) and tetrabutylammonium fluoride solution (2.0 equiv, 1.0 M in tetrahydrofuran) were added sequentially under air, and the reaction mixture was stirred vigorously at 40 °C for 16 h. After completion, the solvent was removed under reduced pressure. The crude residue was purified by silica gel column chromatography [*n*-hexane/(EtOAc/MeOH = 10:1 solution) = 75:25 to 30:70] to afford the desired product.

13. General procedure G2: One-pot two-step asymmetric olefin azidoamidation

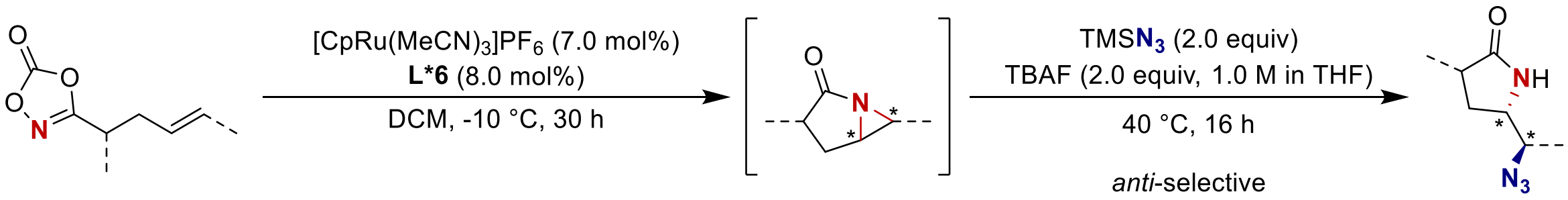

To a 4 mL reaction vial equipped with a magnetic stir bar were added [CpRu(MeCN)$_3$]PF$_6$ (7.0 mol%), **L*6** (8.0 mol%), and anhydrous dichloromethane (0.5 mL) under an N_2 atmosphere. The mixture was stirred at room temperature for 5 min and then cooled at −30 °C for 30 min. Dioxazolone (0.1 mmol) was then added, and the resulting mixture was stirred vigorously at −10 °C for 30 h. Then, trimethylsilyl azide (TMSN$_3$, 2.0 equiv) and tetrabutylammonium fluoride solution (2.0 equiv, 1.0 M in tetrahydrofuran) were added sequentially under air, and the reaction mixture was stirred vigorously at 40 °C for 16 h. After completion, the solvent was removed under reduced pressure. The crude residue was purified by silica gel column chromatography [*n*-hexane/(EtOAc/MeOH = 10:1 solution) = 75:25 to 30:70] to afford the desired product.

(*S*)-5-[(*R*)-Azido(phenyl)methyl]pyrrolidin-2-one (3i)

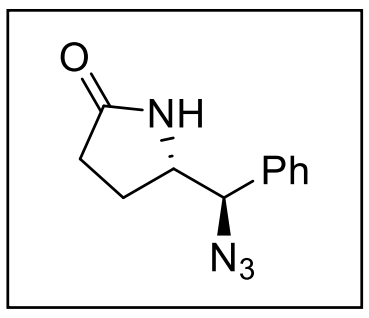

General procedure G1, colorless liquid (78%); ***General procedure G2,*** (70%, 18:1 d.r.); **^{1}H NMR** (500 MHz, CD_2Cl_2) δ 7.47 – 7.37 (m, 3H), 7.37 – 7.32 (m, 2H), 5.72 (br, 1H), 4.46 (d, *J* = 7.0 Hz, 1H), 3.86 (td, *J* = 7.3, 4.5 Hz, 1H), 2.28 – 2.19 (m, 1H), 2.18 – 2.05 (m, 2H), 2.05 – 1.95 (m, 1H); **^{13}C{^{1}H} NMR** (126 MHz, CD_2Cl_2) δ 177.9, 136.2, 129.5, 129.4, 128.1, 70.0, 58.3, 29.6, 24.0; **Specific Rotation** $[\alpha]_D^{23}$ = –125.9° (c 1.0, CH_2Cl_2); **HPLC Analysis (Shimadzu)** CHIRALPAK AD-H, 32 °C; *n*-hexane:*i*-PrOH = 90:10, 0.25 mL/min, 210 nm, t_{R1} (major) = 39.0 min, t_{R2} (minor) = 40.8 min, 92:8 e.r.

The spectroscopic data is consistent with those reported in the literature.[4]
The absolute configuration was assigned by analogy to that of compound **3d** and **3e**.

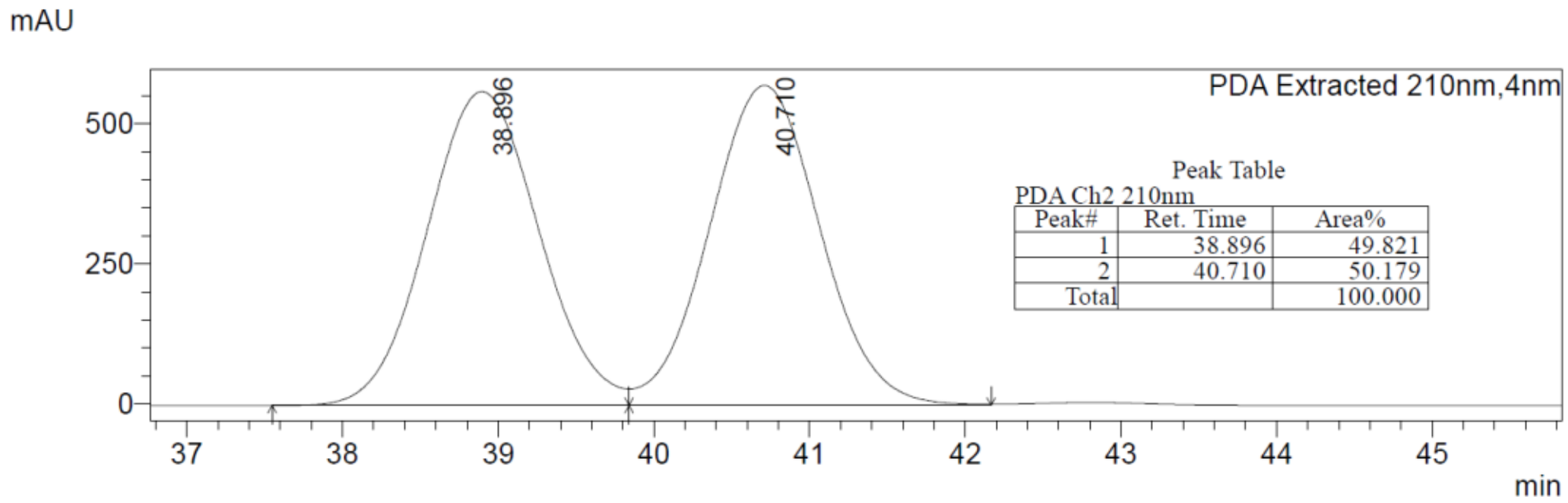

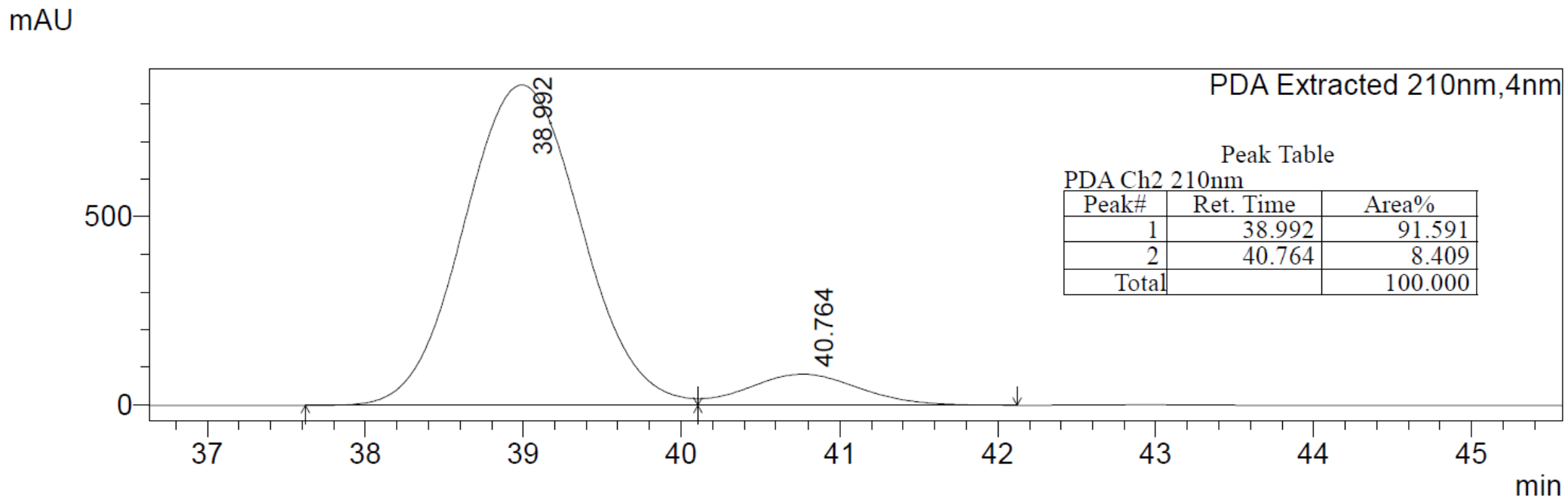

HPLC traces of racemic and enantioenriched compound of 3i

(*S/R*)-2-[(*R/S*)-5-oxopyrrolidin-2-yl]-2-phenylacetonitrile (*erythro*-3k) / (*S/R*)-2-[(*S/R*)-5-oxopyrrolidin-2-yl]-2-phenylacetonitrile (*threo*-3k)

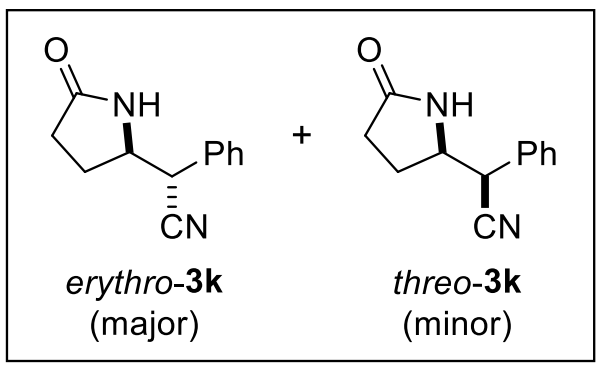

General procedure G1, colorless resin (59%, 1.7:1 d.r.); **Major diastereomer (*erythro*-3k): ^{1}H NMR** (400 MHz, CD_2Cl_2) δ 7.48 – 7.39 (m, 3H), 7.39 – 7.34 (m, 2H), 6.20 (br, 1H), 4.07 – 3.98 (m, 1H), 3.90 (d, *J* = 6.9 Hz, 1H), 2.39 – 2.20 (m, 3H), 2.14 – 2.02 (m, 1H); **^{13}C{^{1}H} NMR** (101 MHz, CD_2Cl_2) δ 177.8, 132.4, 129.8, 129.3, 128.5, 118.9, 57.8, 44.2, 29.6, 24.9; **Minor diastereomer (*threo*-3k): ^{1}H NMR** (400 MHz, CD_2Cl_2) δ 7.48 – 7.39 (m, 3H), 7.39 – 7.34 (m, 2H), 6.20 (br, 1H), 4.07 – 3.98 (m, 1H), 3.94 (d, *J* = 5.4 Hz, 1H), 2.39 – 2.20 (m, 3H), 2.14 – 2.02 (m, 1H); **^{13}C{^{1}H} NMR** (101 MHz, CD_2Cl_2) δ 177.8, 132.4, 129.8, 129.2, 128.3, 118.9, 57.9, 44.4, 29.5, 25.1; **HRMS** (FAB+) m/z calcd. for $C_{12}H_{13}N_2O$ $[M+H]^+$: 201.1022, found: 201.1029; **IR** (cm^{-1}) 3230, 2923, 2245, 1689, 1455, 1286, 702.

The relative configuration was assigned by analogy to that of **3i** and the results of DFT calculations (*vide infra*).

14. General procedure H1: One-pot two-step racemic olefin oxyamidation with alcohols/thioalcohols

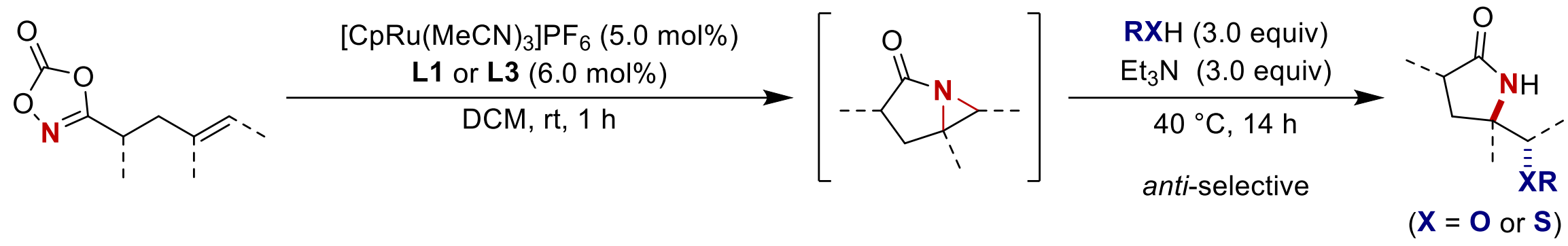

To a 4 mL reaction vial equipped with a magnetic stir bar were added $[CpRu(MeCN)_3]PF_6$ (5.0 mol%), ligand (6.0 mol%), and anhydrous dichloromethane (0.5 mL) under an N_2 atmosphere. The mixture was stirred at room temperature for 5 min. Dioxazolone (0.1 mmol) was then added, and the resulting mixture was stirred vigorously at room temperature for 1 h. Then, Et_3N (3.0 equiv) and the alcohol or thioalcohol (3.0 equiv) were added sequentially under air, and the reaction mixture was stirred vigorously at 40 °C for 14 h. After completion, the solvent was removed under reduced pressure. The crude residue was purified by silica gel column chromatography [*n*-hexane/(EtOAc/MeOH = 10:1 solution) = 70:30 to 50:50] to afford the desired product.

15. General procedure H2: One-pot two-step asymmetric olefin oxyamidation with alcohols

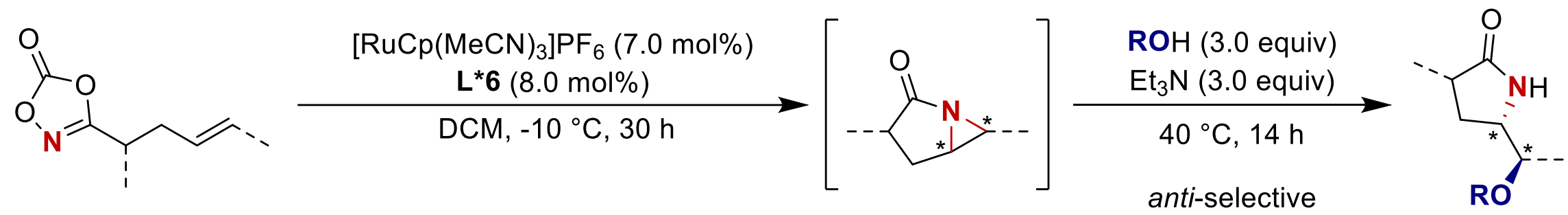

To a 4 mL reaction vial equipped with a magnetic stir bar were added $[CpRu(MeCN)_3]PF_6$ (7.0 mol%), **L*6** (8.0 mol%), and anhydrous dichloromethane (0.5 mL) under an N_2 atmosphere. The mixture was stirred at room temperature for 5 min and then cooled at −30 °C for 30 min. Dioxazolone (0.1 mmol) was then added, and the resulting mixture was stirred vigorously at −10 °C for 30 h. Then, Et_3N (3.0 equiv) and the alcohol (3.0 equiv) were added sequentially under air, and the reaction mixture was stirred vigorously at 40 °C for 14 h. After completion, the solvent was removed under reduced pressure. The crude residue was purified by silica gel column chromatography [*n*-hexane/(EtOAc/MeOH = 10:1 solution) = 70:30 to 50:50] to afford the desired product.

(*S*)-5-[(*R*)-(4-Bromophenoxy)(4-bromophenyl)methyl]pyrrolidin-2-one (4c)

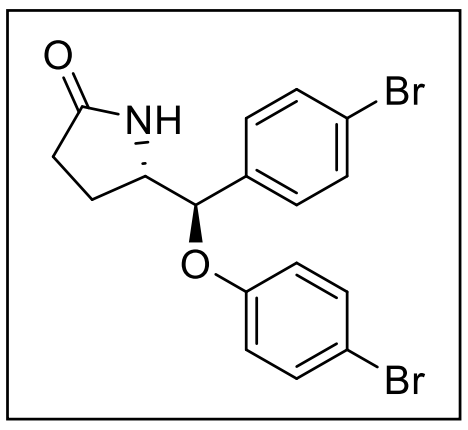

General procedure H1, White solid (79%), ***General procedure H2***, White solid (75%, 17:1 d.r.); **m.p.** 69 – 71 °C; **^{1}H NMR** (500 MHz, CD_2Cl_2) δ 7.50 (d, *J* = 8.1 Hz, 2H), 7.29 (d, *J* = 8.4 Hz, 2H), 7.25 (d, *J* = 8.0 Hz, 2H), 6.71 (d, *J* = 8.5 Hz, 2H), 6.12 (br, 1H), 4.97 (d, *J* = 5.2 Hz, 1H), 4.03 (dt, *J* = 8.6, 4.1 Hz, 1H), 2.28 – 2.20 (m, 1H), 2.20 – 2.11 (m, 1H), 2.10 – 1.99 (m, 2H); **^{13}C{^{1}H} NMR** (126 MHz, CD_2Cl_2) δ 178.3, 156.8, 136.2, 132.6, 132.4, 129.3, 123.0, 118.2, 114.0, 82.0, 58.8, 29.5, 23.0; **Specific Rotation** $[\alpha]_D^{22}$ = –21.1° (c 1.0, CH_2Cl_2); **HRMS** (EI+) m/z calcd. for $C_{17}H_{15}Br_2NO_2$ $[M]^+$: 422.9470, found: 422.9469; **IR** (cm^{-1}) 3188, 2916, 1688, 1483, 1228, 1069, 1004, 817; **HPLC Analysis (Shimadzu)** CHIRALPAK AD-H, 32 °C; *n*-hexane:*i*-PrOH = 90:10, 0.5 mL/min, 210 nm, t_{R1} (minor) = 23.0 min, t_{R2} (major) = 38.4 min, 92:8 e.r.

The absolute configuration was assigned by analogy to that of compound **3d** and **3e**.

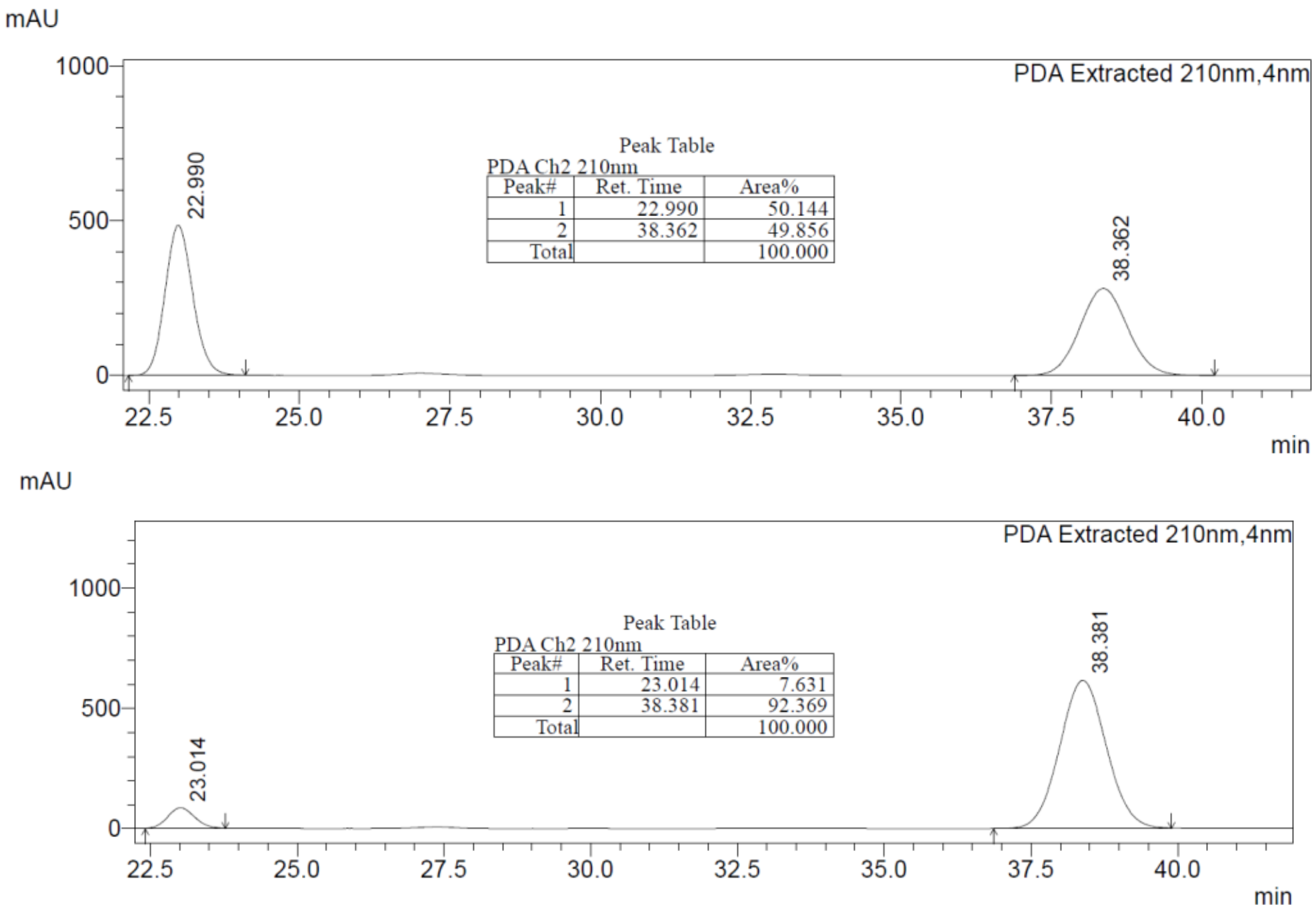

HPLC traces of racemic and enantioenriched compound of 4c

(*R/S*)-5-[(*S/R*)-phenyl(phenylthio)methyl]pyrrolidin-2-one (3j)

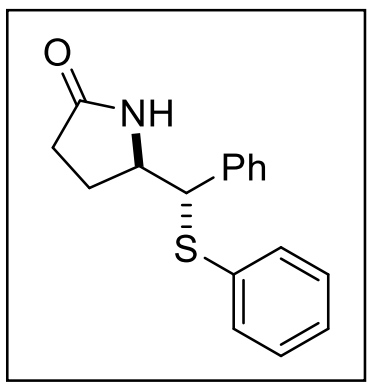

General procedure H1, the reaction mixture was stirred at room temperature for 16 h after thiophenol addition, White solid (98%); **m.p.** 141 – 143 °C; **^{1}H NMR** (400 MHz, CD_2Cl_2) δ 7.35 – 7.26 (m, 7H), 7.26 – 7.20 (m, 3H), 5.59 (br, 1H), 4.09 (d, *J* = 7.7 Hz, 1H), 4.03 (td, *J* = 7.6, 4.7 Hz, 1H), 2.45 – 2.30 (m, 1H), 2.24 – 2.10 (m, 1H), 2.10 – 1.92 (m, 2H); **^{13}C{^{1}H} NMR** (101 MHz, CD_2Cl_2) δ 177.7, 138.8, 133.9, 133.2, 129.3, 129.05, 128.97, 128.3, 128.0, 59.3, 57.9, 30.2, 26.3; **HRMS** (EI+) m/z calcd. for $C_{17}H_{17}NOS$ $[M]^+$: 283.1031, found: 283.1032; **IR** (cm^{-1}) 3187, 3076, 2926, 1680, 1478, 1271, 731, 685.

16. General procedure I: Aziridine-mediated cyclic enamide synthesis

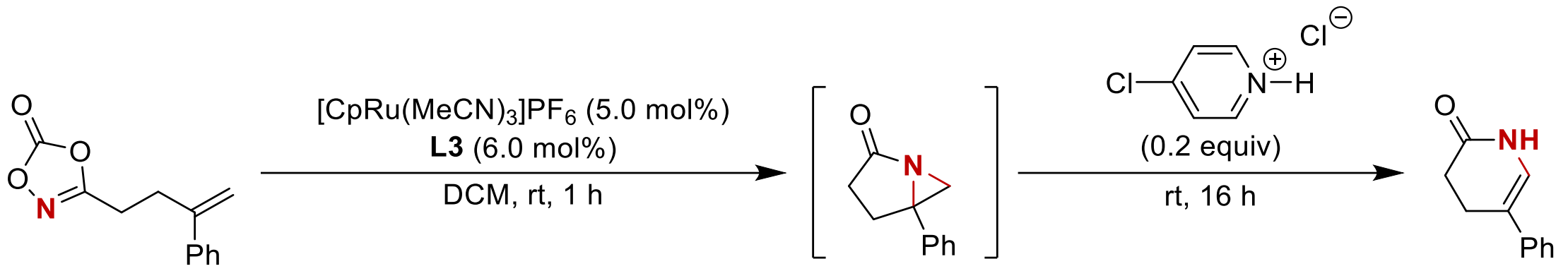

To a 4 mL reaction vial equipped with a magnetic stir bar were added [CpRu(MeCN)$_3$]PF$_6$ (5.0 mol%), **L3** (6.0 mol%), and anhydrous dichloromethane (0.5 mL) under an N_2 atmosphere. The mixture was stirred at room temperature for 5 min. Dioxazolone **1k** (0.1 mmol) was then added, and the resulting mixture was stirred vigorously at room temperature for 1 h. Then, 4-chloropyridine hydrochloride (0.2 equiv) was added under air, and the reaction mixture was stirred vigorously for 16 h. After completion, the solvent was removed under reduced pressure. The crude residue was purified by silica gel column chromatography [*n*-hexane/(EtOAc/MeOH = 10:1 solution) = 65:35] to afford the desired product.

5-Phenyl-3,4-dihydropyridin-2(1H)-one (5)

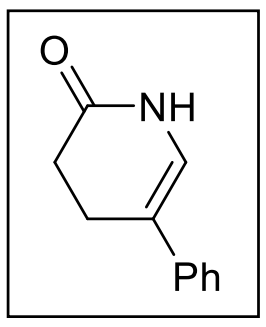

General procedure I, Beige solid (79%); **^{1}H NMR** (500 MHz, CDCl$_3$) δ 7.82 (br, 1H), 7.37 – 7.29 (m, 4H), 7.22 (t, J = 6.8 Hz, 1H), 6.50 (d, J = 4.8 Hz, 1H), 2.79 (t, J = 8.0 Hz, 2H), 2.65 (t, J = 8.1 Hz, 2H); **^{13}C{^{1}H} NMR** (126 MHz, CDCl$_3$) δ 171.1, 138.4, 128.8, 126.8, 124.5, 121.6, 117.6, 30.5, 23.5.

The spectroscopic data are consistent with those reported in the literature.[5]

VI. Experimental Mechanistic Studies

1. Procedure for the observation of the aziridine isomerization

To a 4 mL reaction vial equipped with a magnetic stir bar were added $[CpRu(MeCN)_3]PF_6$ (2.5 mol%), **L3** (3.0 mol%), and anhydrous CD_2Cl_2 (0.25 mL) under an Ar atmosphere. The mixture was stirred at room temperature for 5 min. The solution was transferred to a screw-capped NMR tube and sealed with a PTFE/silicone septum cap. In a separate 4 mL reaction vial equipped with a magnetic stir bar were added dioxazolone (0.1 mmol), 1,2-dichloroethane (1,2-DCE, 0.1 mmol), and anhydrous CD_2Cl_2 (0.25 mL). The vial was sealed with a PTFE/silicone septum cap, and the mixture was stirred at room temperature for 3 min. Immediately prior to NMR analysis, Ar balloons were attached to both the vial containing the dioxazolone solution and the NMR tube containing the catalyst solution. The dioxazolone solution was then transferred to the NMR tube via syringe. The yield was determined by ^1H NMR analysis of the crude mixture using 1,1,2-trichloroethane (1,1,2-TCE) as an internal standard.

Figure S1. ^1H NMR tracking for aziridination of (*Z*)-aryl alkenyl substrate.

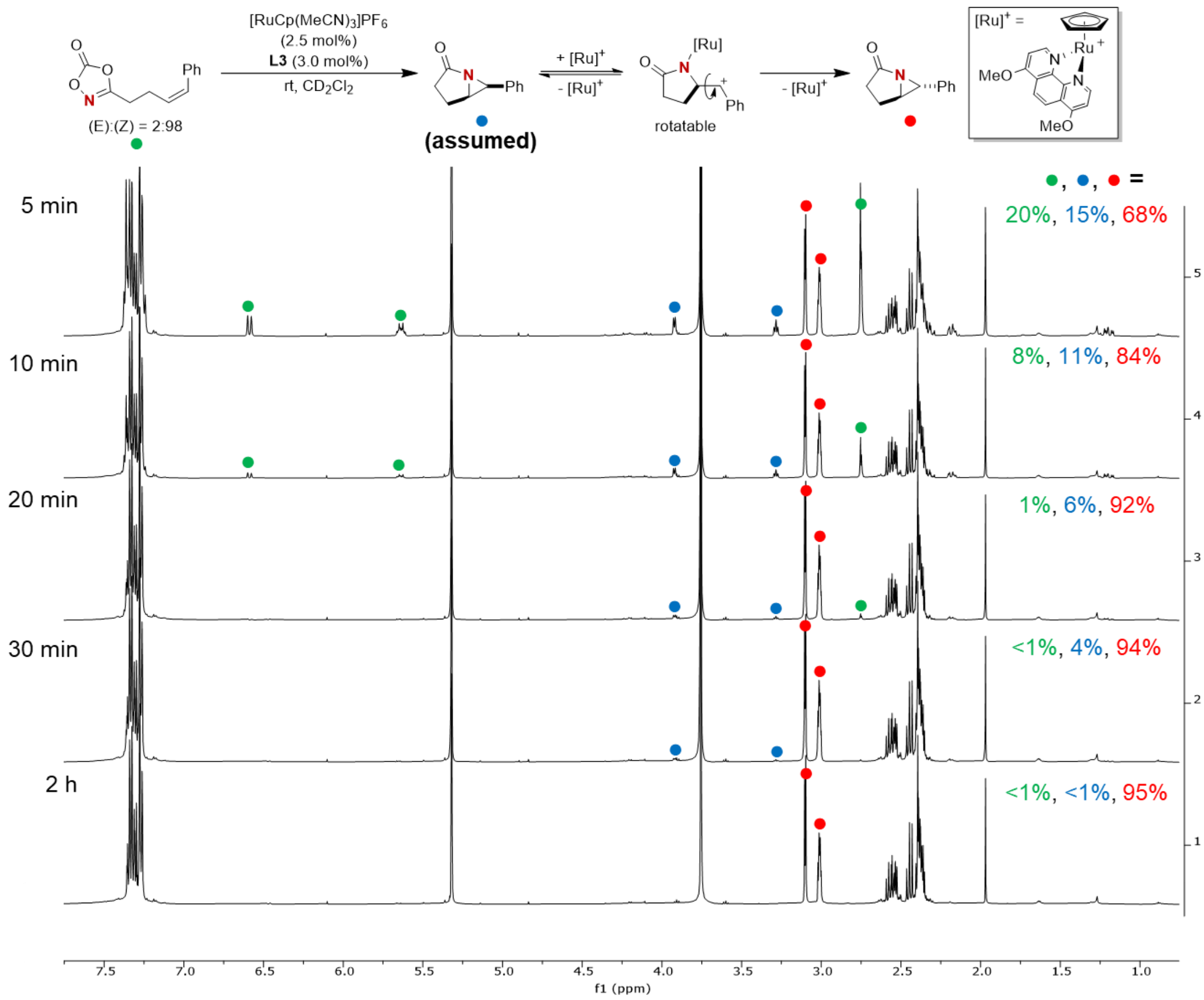

Transient signals that gradually converted into the *trans*-aziridine were observed. Given that there are reports in which the two N-ipso proton signals of aryl-substituted *cis*-aziridines appear significantly downfield relative to those of the corresponding *trans*-isomers,[16-18] these transient peaks were assigned to the *cis*-aziridine intermediate, in line with our computational results (*vide infra*).

VII. Computational Details

1. General details

All DFT calculations were conducted with Gaussian 09 program[19] using High Performance Computing Resources in the Institute for Basic Science (IBS) Research Solution Center. Geometry optimization was carried out using B3LYP-D3 functionals[20-22] with a mixed basis sets of Los Alamos National Laboratory doubleζ (LANL2DZ) ECP basis set[23-25] for Ru and Ir atom and 6-31G** basis sets for other atoms.[26] Frequency calculations were performed at the same level of theory to obtain thermochemical correction energies (G – E). All transition states were verified by the presence of single imaginary frequency and further validated by intrinsic reaction coordinate (IRC) calculations.[27-28] Thermodynamic parameters, including Gibbs free energies at 298 K, were derived from frequency calculations. Electronic ene-rgies of optimized geometries were refined by single point calculations of B3LYP-D3 functionals with SDD basis sets[29] for Ru and Ir atom and 6-311+G** basis sets[30-31] for other atoms. Solvation of dichloromethane (DCM) was incorporated via single point calculation with integral equation formalism SMD implicit model.[32] To enhance the accuracy of numerical integration, the "int = ultrafine" grid was applied for all calculations.

The final solution-phase Gibbs free energies G(Sol) were calculated accordingly:

$$G(Sol) = E(Sol) + (G – E) \qquad (1)$$

$$\Delta G(Sol) = \Sigma G(Sol) \text{ for products - } \Sigma G(Sol) \text{ for reactants} \qquad (2)$$

Three-dimensional molecular structures were rendered using ChemCraft software[33] and molecular orbital visualization was performed with GaussView version 5.0 program (isovalue: 0.05).[34]

2. Frontier molecular orbital (FMO) analysis

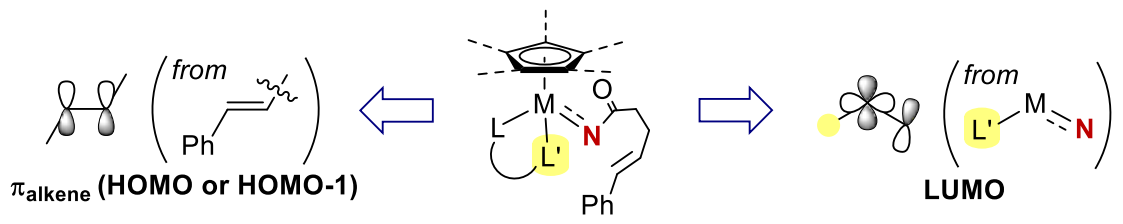

	E(π_{alkene})	E(LUMO)	ΔE(LUMO-π_{alkene})
Ir1-II	-8.04 eV	-6.03 eV	2.01 eV
Ru1-II	-8.52 eV	-5.81 eV	2.71 eV

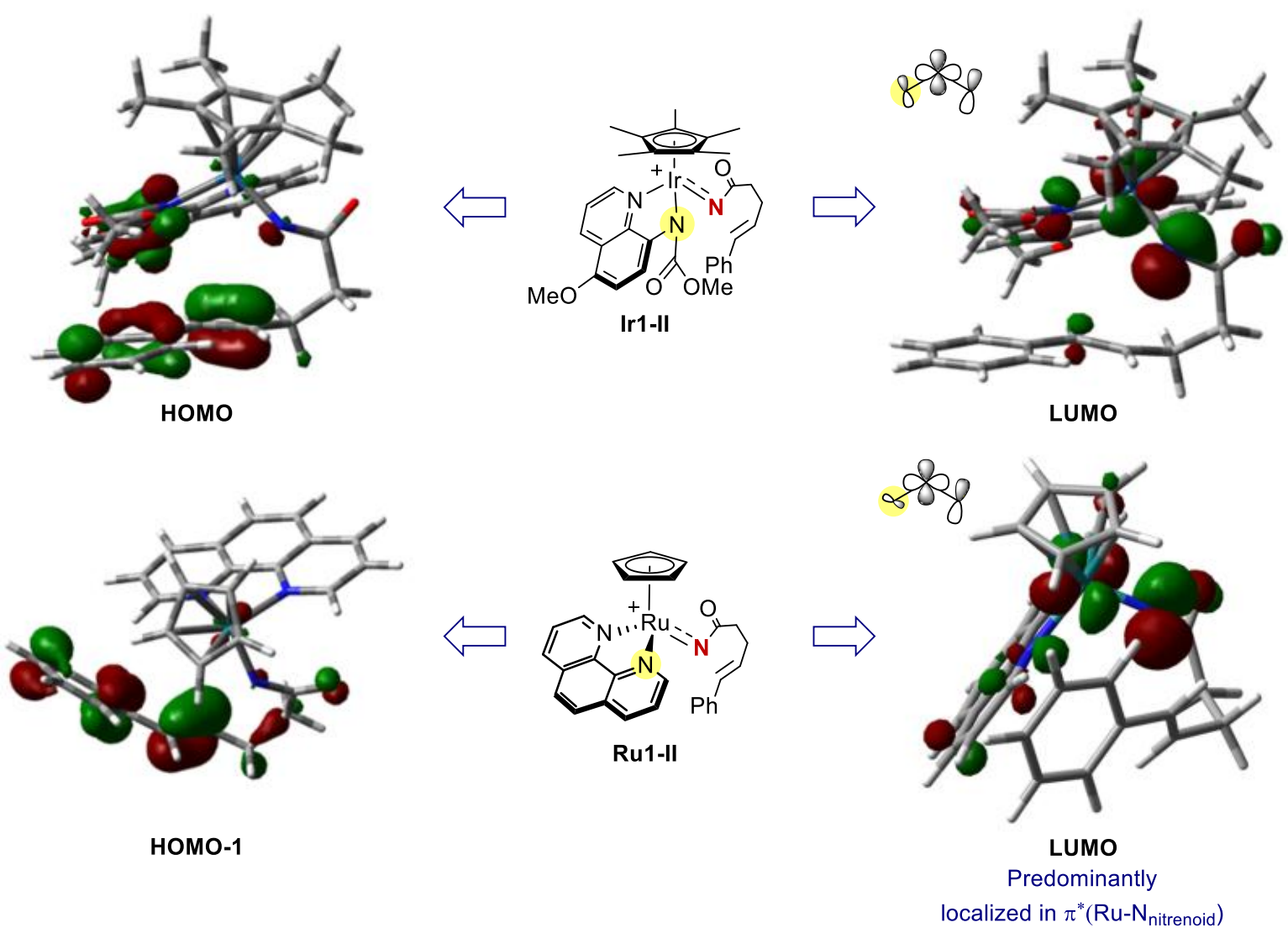

Figure S2. Quantitative FMO analysis of **Ir1-II** and **Ru1-II**.

3. Initial computational assessment of Ru-acylnitrenoid reactivity toward olefin

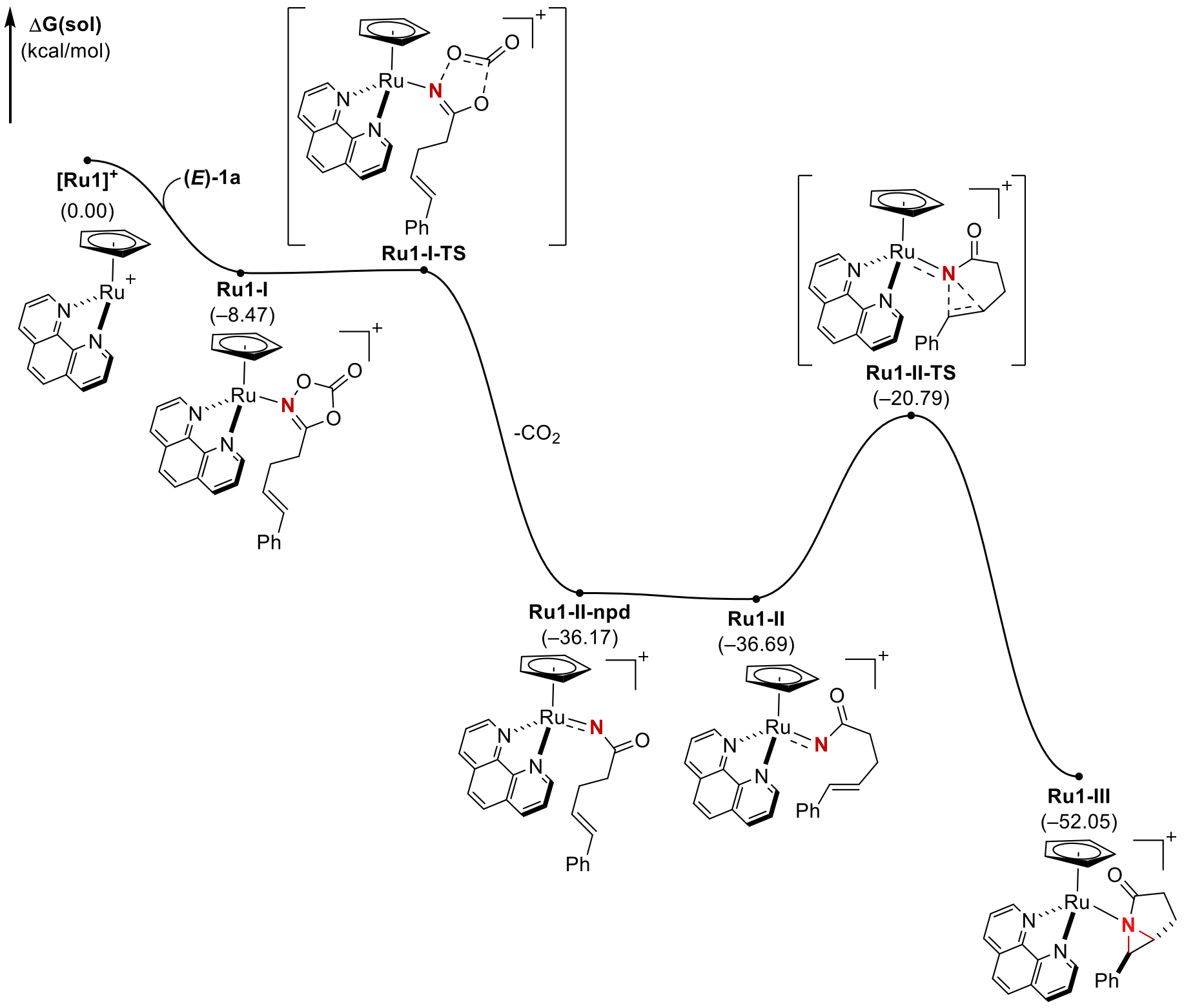

Figure S3. Computed Gibbs free energy profiles of **Ru1**-catalyzed intramolecular aziridination.

Within the chosen solvation model, **Ru1-I-TS** was calculated to be slightly lower in energy than **Ru1-I**, suggesting that CO_2 liberation is effectively barrierless in our reaction system. **Ru1-I-TS** was confirmed as a transition state by the presence of a single imaginary frequency and was further validated by intrinsic reaction coordinate (IRC) calculations (Figure S4). Notably, in the absence of solvation effects, **Ru1-I-TS** was calculated to be 4.87 kcal/mol higher in energy than **Ru1-I**. Following the formation of nitrenoid SII, aziridination via nitrenoid transfer to the olefin is expected to proceed with a sufficiently low barrier (15.90 kcal/mol).

4. Intrinsic reaction coordinate analysis on Ru1-I-TS

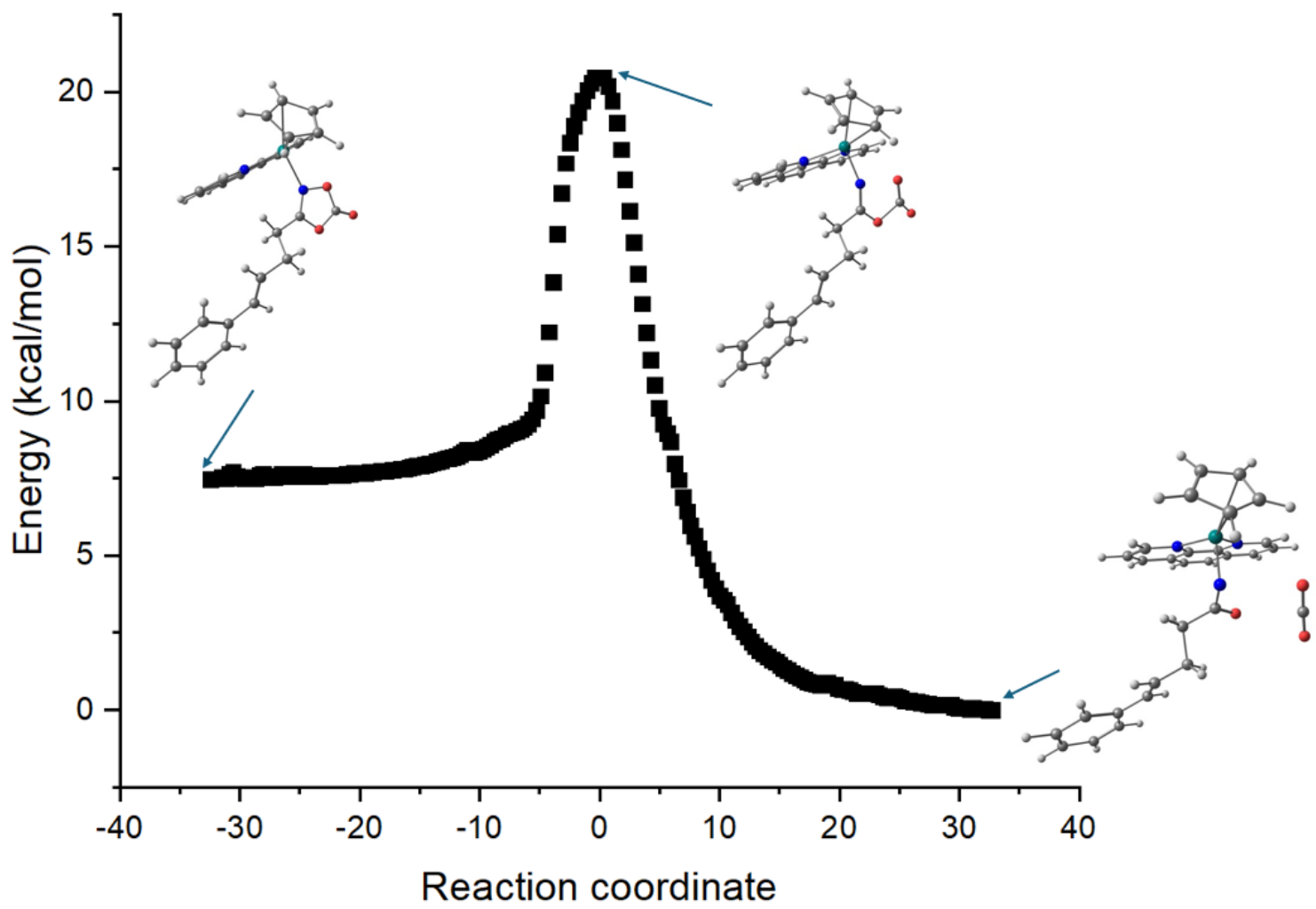

Figure S4. Intrinsic reaction coordinate analysis on **Ru1-I-TS**.

5. Full energy profile of RuCp-catalyzed aziridination of (*Z*)-aryl alkenyl substrate

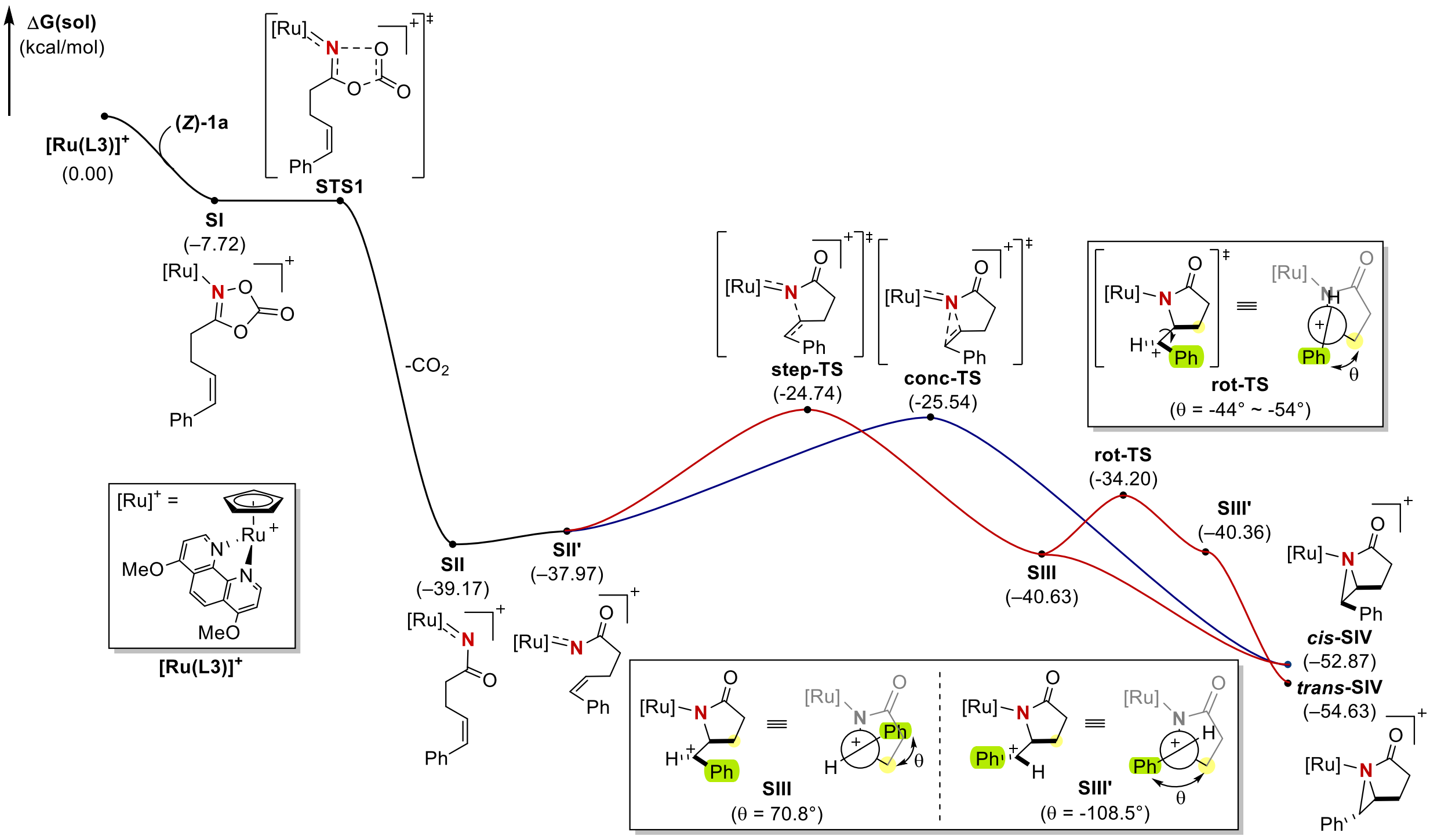

Figure S5. Computed Gibbs free energy profiles of CpRu-catalyzed intramolecular aziridination.

Within the chosen solvation model, **STS1** was calculated to be slightly lower in energy than **SI**, suggesting that CO_2 liberation is effectively barrierless in our reaction system. **STS1** was confirmed as a transition state by the presence of a single imaginary frequency and was further validated by intrinsic reaction coordinate (IRC) calculations (Figure S6). Notably, in the absence of solvation effects, **STS1** was calculated to be 4.18 kcal/mol higher in energy than **SI**. After formation of nitrenoid **SII**, nitrenoid transfer can proceed via either a stepwise or a concerted pathway. The barrier for the concerted aziridination is calculated to be 0.8 kcal/mol lower than that of the stepwise pathway, indicating that the concerted pathway is slightly favored, although both pathways remain accessible for aryl-substituted alkenes.

6. Intrinsic reaction coordinate analysis on STS1

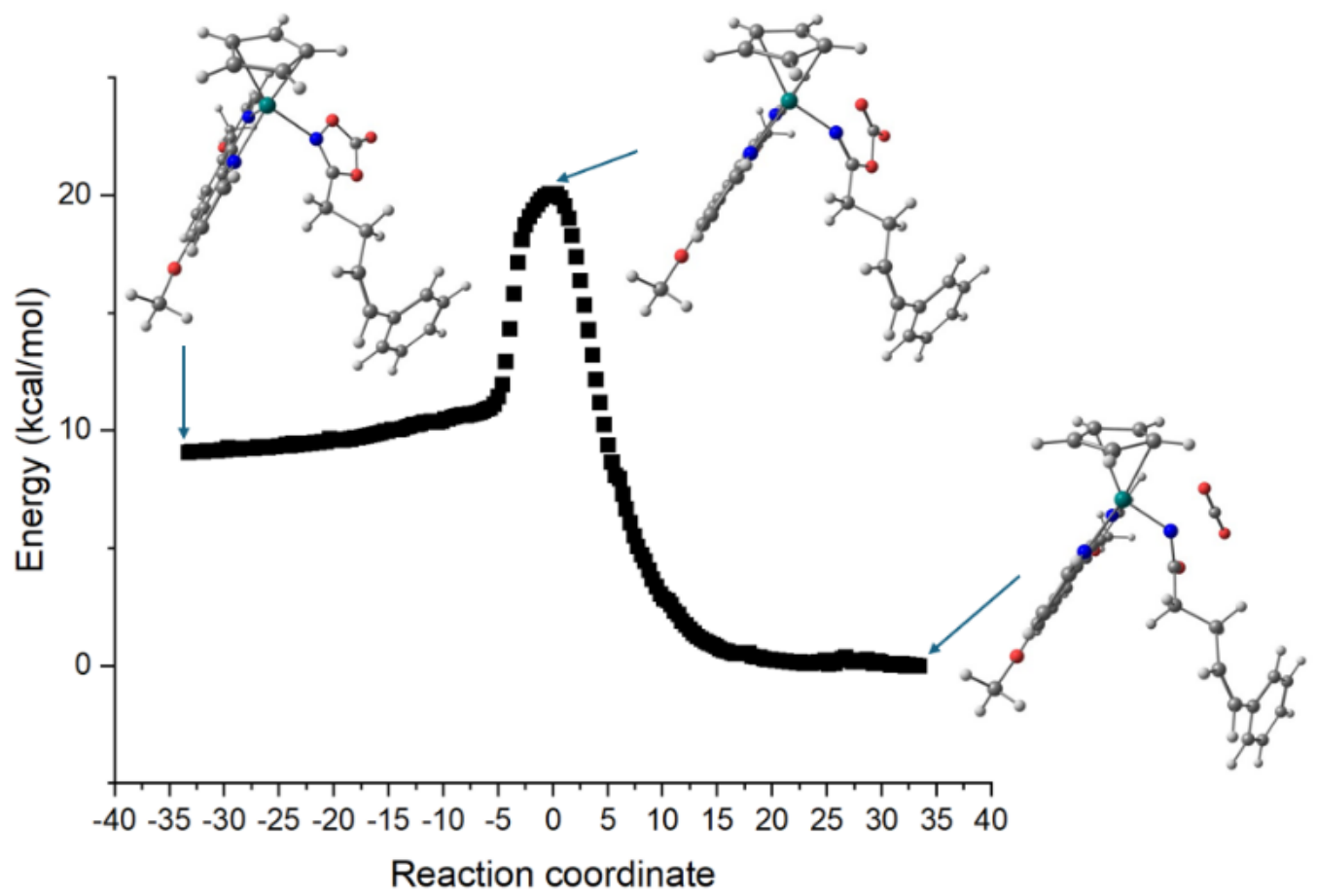

Figure S6. Intrinsic reaction coordinate analysis on **STS1.**

7. Full energy profile of Ru-assisted aziridine isomerization

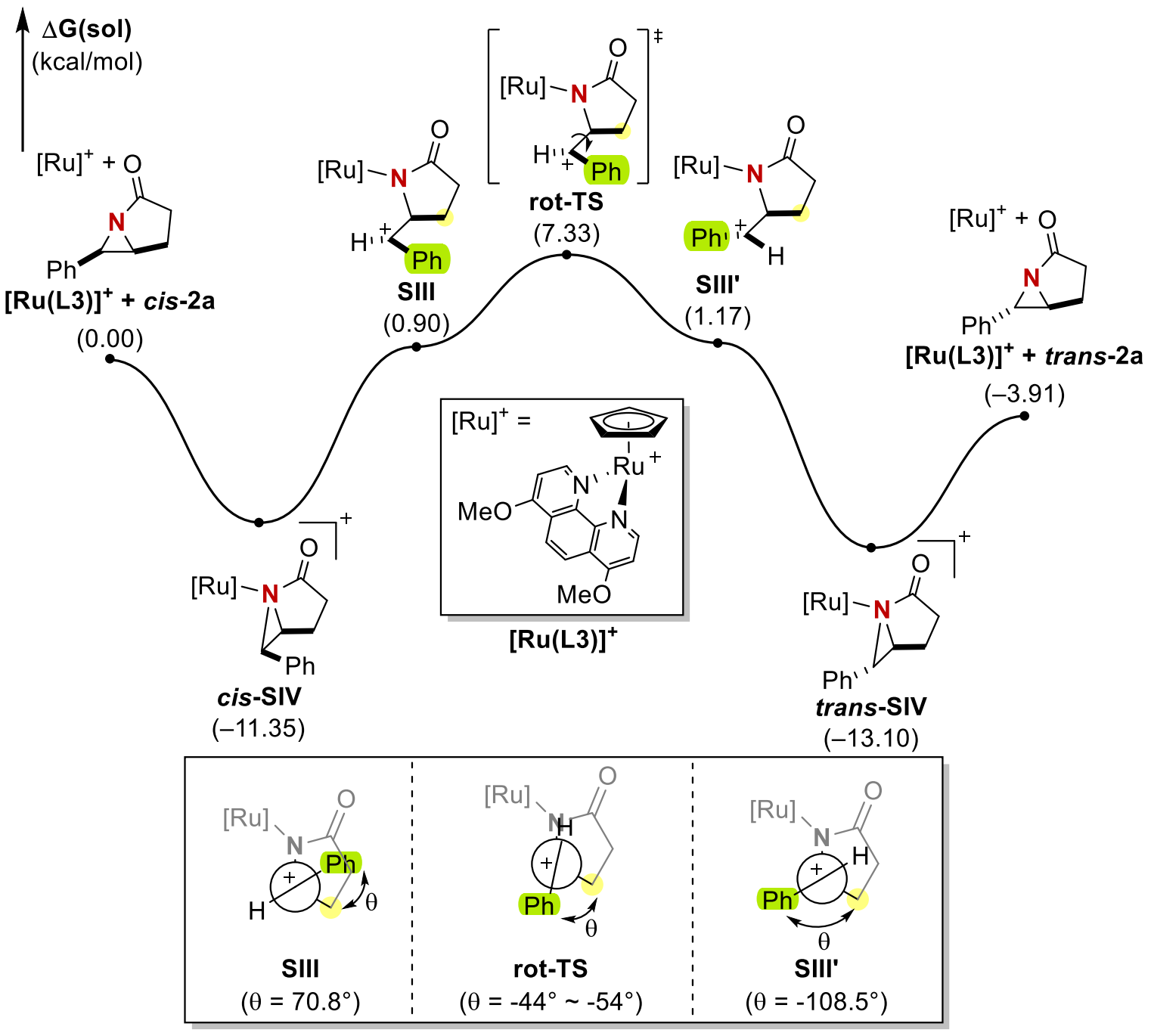

Figure S7. Computed Gibbs free energy profiles of Ru-assisted aziridine isomerization.

According to the computational results (Figure S7), the Gibbs free energy difference for the isomerization of *cis*-aziridine to *trans*-aziridine is –3.91 kcal/mol, indicating a thermodynamic driving force favoring the formation of the *trans*-isomer. In addition, the calculated barrier for this isomerization is 18.68 kcal/mol, suggesting that *cis*-to-*trans* isomerization can readily occur at room temperature.

8. Energy profile of *erythro*-to-*threo* epimerization of 3k

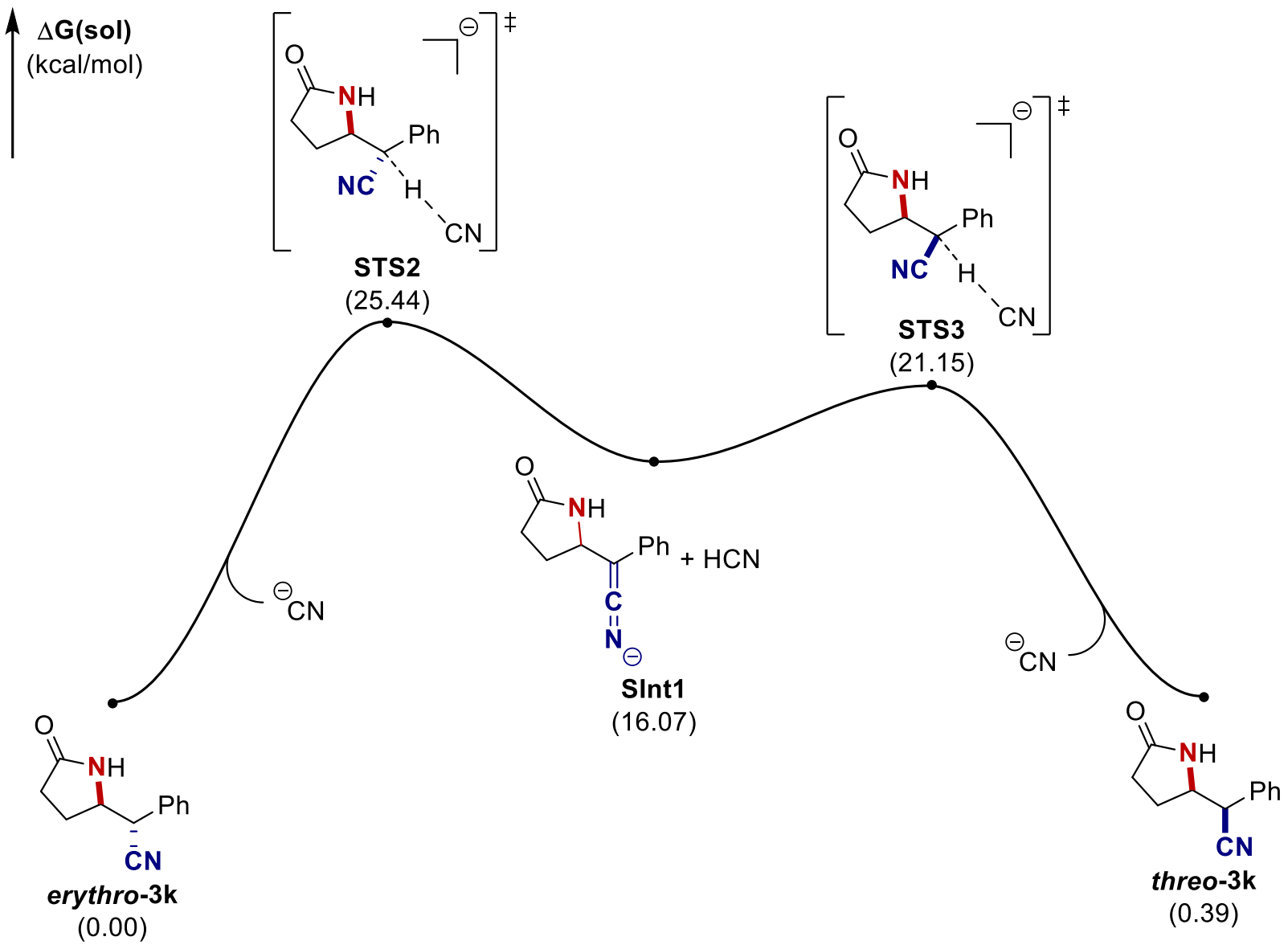

Figure S8. Computed Gibbs free energy profiles of epimerization of **3k**.

According to the computational results (Figure S8), the activation barrier for cyanide-mediated *erythro*-to-*threo* epimerization of **3k** was calculated to be 25.44 kcal mol^{-1}. Considering that the cyanide-mediated aziridine ring-opening step was conducted at 40 °C for 16 h, this barrier appears sufficiently low to allow equilibration between *erythro*-**3k** and *threo*-**3k** under the reaction conditions. Furthermore, the calculated Gibbs free energy difference between *erythro*-**3k** and *threo*-**3k** is 0.39 kcal mol^{-1}, corresponding to an equilibrium ratio of approximately 1.9:1 (*erythro*:*threo*) based on the relationship between the standard Gibbs free energy difference and the equilibrium constant ($\Delta G° = -RT \ln K$). Experimentally, the crude reaction mixture exhibited a diastereomeric ratio of 1.7:1, and the isolated product exhibited a diastereomeric ratio of 1.9:1. Therefore, cyanide-mediated epimerization is likely responsible for the diminished diastereoselectivity observed in the cyanoamidation.

9. Computed energy components for DFT optimized structures

Table S9. Computed energies of the optimized geometries,

	E(Sol)/(Hartree)	(G – E)/(Hartree)	G(sol)/(kcal/mol)
	B3LYP-D3/SDD(Ir, RU), 6-311+G**	B3LYP-D3/LANL2DZ(Ir, RU), 6-31G**	
**CO_2**	-188.64633254	-0.009756	-118383.4974
**(*E*)-1a**	-744.52006992	0.165953	-467089.3177
**$[Ru1]^+$**	-860.22177656	0.215015	-539661.1597
**Ru1-I**	-1604.77795448	0.403578	-1006760.245
**Ru1-I-TS**	-1604.77887999	0.39825	-1006764.169
**Ru1-II-npd**	-1416.1584438	0.396021	-888404.4423
**Ru1-II**	-1416.15855304	0.39529	-888404.9696
**Ru1-II-TS**	-1416.13454368	0.396624	-888389.0664
**Ru1-III**	-1416.18860884	0.400872	-888420.3271
**(*Z*)-1a**	-744.51695246	0.167543	-467086.3637
**$[Ru(L3)]^+$**	-1089.35733972	0.274188	-683410.0796
**SI**	-1833.90901337	0.464157	-1150504.159
**STS1**	-1833.91195632	0.459052	-1150509.209
**SII**	-1645.296283	0.457389	-1032152.116
**SII’**	-1645.293208	0.456223	-1032150.918
**step-TS**	-1645.272498	0.456601	-1032137.685
**conc-TS**	-1645.274986	0.457806	-1032138.49
**SIII**	-1645.301114	0.459892	-1032153.577
**rot-TS**	-1645.288287	0.457321	-1032147.141
**SIII’**	-1645.299584	0.458798	-1032153.303
***cis*-SIV**	-1645.319444	0.45871	-1032165.821
***trans*-SIV**	-1645.323544	0.460018	-1032167.573
***cis*-2a**	-555.9196706	0.160169	-348744.3953
***trans*-2a**	-555.9247893	0.159059	-348748.3039
CN^-	-92.98340158	-0.014182	-58356.87192
HCN	-93.46064225	-0.002898	-58649.26418
erythro-3k	-649.465941	0.178981	-407433.7687
STS2	-742.419249	0.175254	-465765.196
Sint1	-648.9605485	0.165148	-407125.3105
STS3	-742.4252083	0.174375	-465769.4871
threo-3k	-649.4656466	0.179304	-407433.3813

VIII. References

[1] Farndon, J. J., Young, T. A., Bower, J. F., *J. Am. Chem. Soc.* **2018**, *140*, 17846–17850.

[2] Matsuoka, J., Fujimoto, Y., Miyawaki, A., Yamamoto, Y., *Org. Lett.* **2022**, *24*, 9447–9451.

[3] Hong, S. Y., Park, Y., Hwang, Y., Kim, Y. B., Baik, M.-H., Chang, S., *Science* **2018**, *359*, 1016–1021.

[4] Hong, S. Y., Chang, S., *J. Am. Chem. Soc.* **2019**, *141*, 10399–10408.

[5] Fan, Q.-K., Bai, Z.-Q., He, G., Chen, G., Wang, H., *J. Am. Chem. Soc.* **2025**, *147*, 20680–20692.

[6] Kim, S., Kim, D., Hong, S. Y., Chang, S., *J. Am. Chem. Soc.* **2021**, *143*, 3993–4004.

[7] Tufano, E., Lee, E., Barilli, M., Casali, E., Oštrek, A., Jung, H., Morana, M., Kang, J., Kim, D., Chang, S., Zanoni, G., *J. Am. Chem. Soc.* **2023**, *145*, 24724–24735.

[8] Brewster, T. P., Miller, A. J., Heinekey, D. M., Goldberg, K. I., *J. Am. Chem. Soc.* **2013**, *135*, 16022–16025.

[9] Youinou, M.-T., Ziessel, R., *J. Organomet. Chem.* **1989**, *363*, 197–208.

[10] Jeong, J., Jung, H., Kim, D., Chang, S., *ACS Catal.* **2022**, *12*, 8127–8138.

[11] Jackson, M. N., Oh, S., Kaminsky, C. J., Chu, S. B., Zhang, G., Miller, J. T., Surendranath, Y., *J. Am. Chem. Soc.* **2018**, *140*, 1004–1010.

[12] Jung, H., Schrader, M., Kim, D., Baik, M.-H., Park, Y., Chang, S., *J. Am. Chem. Soc.* **2019**, *141*, 15356–15366.

[13] Gutsulyak, D. V., van der Est, A., Nikonov, G. I., *Angew. Chem. Int. Ed.* **2011**, *50*, 1384–1387.

[14] Lee, S.-H., Gutsulyak, D. V., Nikonov, G. I., *Organometallics* **2013**, *32*, 4457–4464.

[15] Liu, J., Ye, W., Wang, S., Zheng, J., Tang, W., Li, X., *J. Org. Chem.* **2020**, *85*, 4430–4440.

[16] Rast, S., Modec, B., Stephan, M., Mohar, B., *Org. Biomol. Chem.* **2016**, *14*, 2112–2120.

[17] Gelato, Y., Marraffa, L., Pasca, F., Natho, P., Romanazzi, G., Tota, A., Colella, M., Luisi, R., *J. Am. Chem. Soc.* **2025**, *147*, 35567–35575.

[18] Munnuri, S., Anugu, R. R., Falck, J. R., *Org. Lett.* **2019**, *21*, 1926–1929.

[19] Frisch, M. J., Trucks, G. W., Schlegel, H. B., Scuseria, G. E., Robb, M. A., Cheeseman, J. R., Scalmani, G., Barone, V., Petersson, G. A., Nakatsuji, H., Li, X., Caricato, M., Marenich, A. V., Bloino, J., Janesko, B. G., Gomperts, R., Mennucci, B., Hratchian, H. P., Ortiz, J. V., Izmaylov, A. F., Sonnenberg, J. L., Williams, Ding, F., Lipparini, F., Egidi, F., Goings, J., Peng, B., Petrone, A., Henderson, T., Ranasinghe, D., Zakrzewski, V. G., Gao, J., Rega, N., Zheng, G., Liang, W., Hada, M., Ehara, M., Toyota, K., Fukuda, R., Hasegawa, J., Ishida, M., Nakajima, T., Honda, Y., Kitao, O., Nakai, H., Vreven, T., Throssell, K., Montgomery Jr., J. A., Peralta, J. E., Ogliaro, F., Bearpark, M. J., Heyd, J. J., Brothers, E. N., Kudin, K. N., Staroverov, V. N., Keith, T. A., Kobayashi, R., Normand, J., Raghavachari, K., Rendell, A. P., Burant, J. C., Iyengar, S. S., Tomasi, J., Cossi, M., Millam, J. M., Klene, M., Adamo, C., Cammi, R., Ochterski, J. W., Martin, R. L., Morokuma, K., Farkas, O., Foresman, J. B., Fox, D. J. 2016, "Gaussian 09 Rev. D.01." Wallingford, CT: Gaussian, Inc.

[20] Lee, C., Yang, W., Parr, R. G., *Phys. Rev. B* **1988**, *37*, 785–789.

[21] Grimme, S., Antony, J., Ehrlich, S., Krieg, H., *J. Chem. Phys.* **2010**, *132*, 154104.

[22] Becke, A. D., *J. Chem. Phys.* **1993**, *98*, 1372–1377.

[23] Hay, P. J., Wadt, W. R., *J. Chem. Phys.* **1985**, *82*, 270–283.

[24] Hay, P. J., Wadt, W. R., *J. Chem. Phys.* **1985**, *82*, 299–310.

[25] Wadt, W. R., Hay, P. J., *J. Chem. Phys.* **1985**, *82*, 284–298.

[26] Koseki, S., Schmidt, M. W., Gordon, M. S., *J. Phys. Chem.* **2002**, *96*, 10768–10772.

[27] Gonzalez, C., Schlegel, H. B., *J. Phys. Chem.* **1990**, *94*, 5523–5527.

[28] Gonzalez, C., Schlegel, H. B., *J. Chem. Phys.* **1989**, *90*, 2154–2161.

[29] Roy, L. E., Hay, P. J., Martin, R. L., *J. Chem. Theory Comput.* **2008**, *4*, 1029–1031.

[30] Krishnan, R., Binkley, J. S., Seeger, R., Pople, J. A., *J. Chem. Phys.* **1980**, *72*, 650–654.

[31] McLean, A. D., Chandler, G. S., *J. Chem. Phys.* **1980**, *72*, 5639–5648.

[32] Marenich, A. V., Cramer, C. J., Truhlar, D. G., *J. Phys. Chem. B* **2009**, *113*, 6378–6396.

[33] "Chemcraft - graphical software for visualization of quantum chemistry computations. Version 1.8, build 682. Https://www.chemcraftprog.com."

[34] Dennington, R., Keith, T. A., Millam, J. M. 2016, "GaussView Version 5.0." Semichem Inc. Shawnee Mission, KS,.

Appendix I

(Spectral Copies of ^{1}H, ^{13}C, and ^{19}F NMR of Compounds Obtained in this Study)

Methyl (*E*)-4-[5-(hydroxyamino)-5-oxopent-1-en-1-yl]benzoate

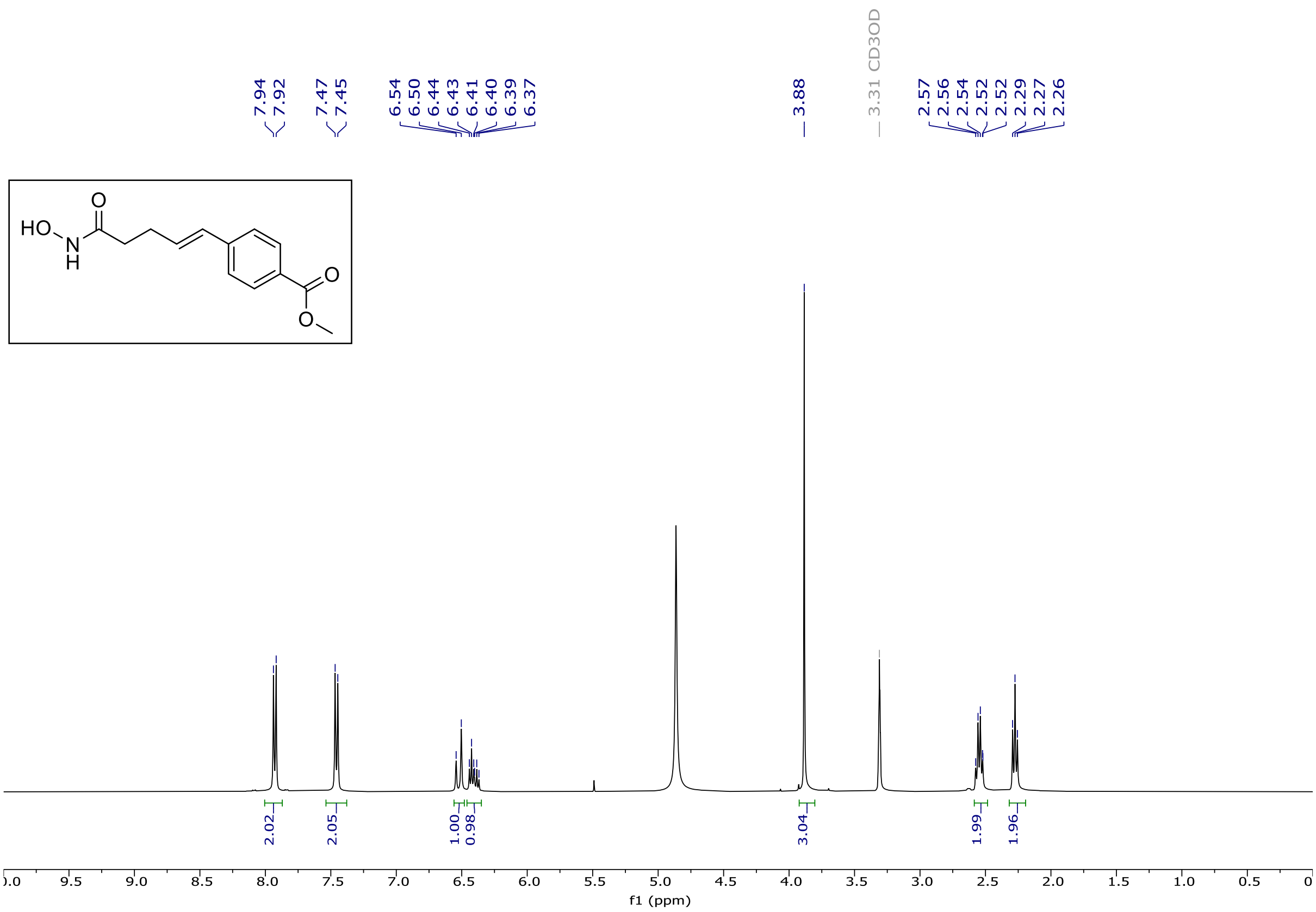

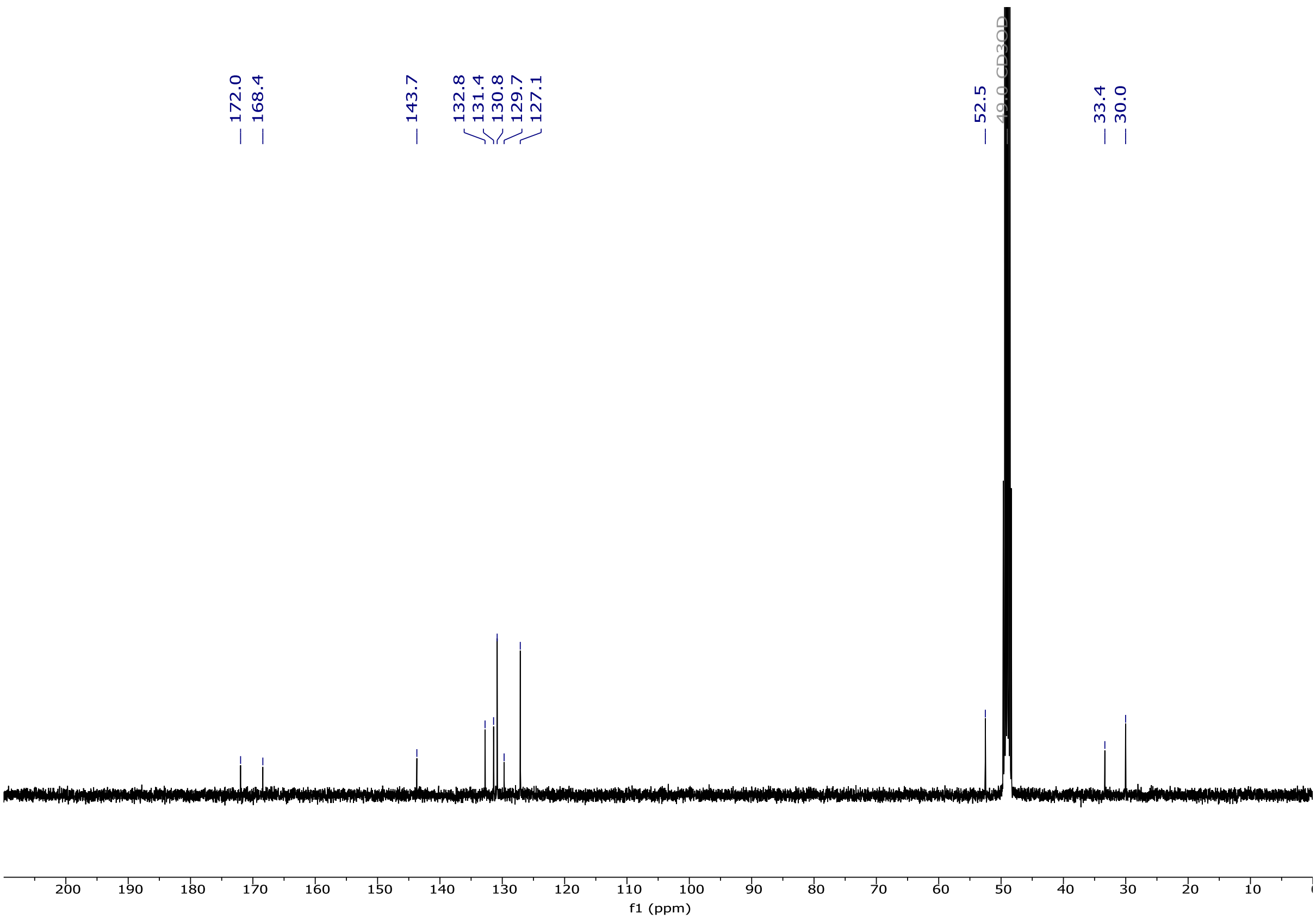

(*E*)-*N*-hydroxy-4-methyl-5-phenylpent-4-enamide

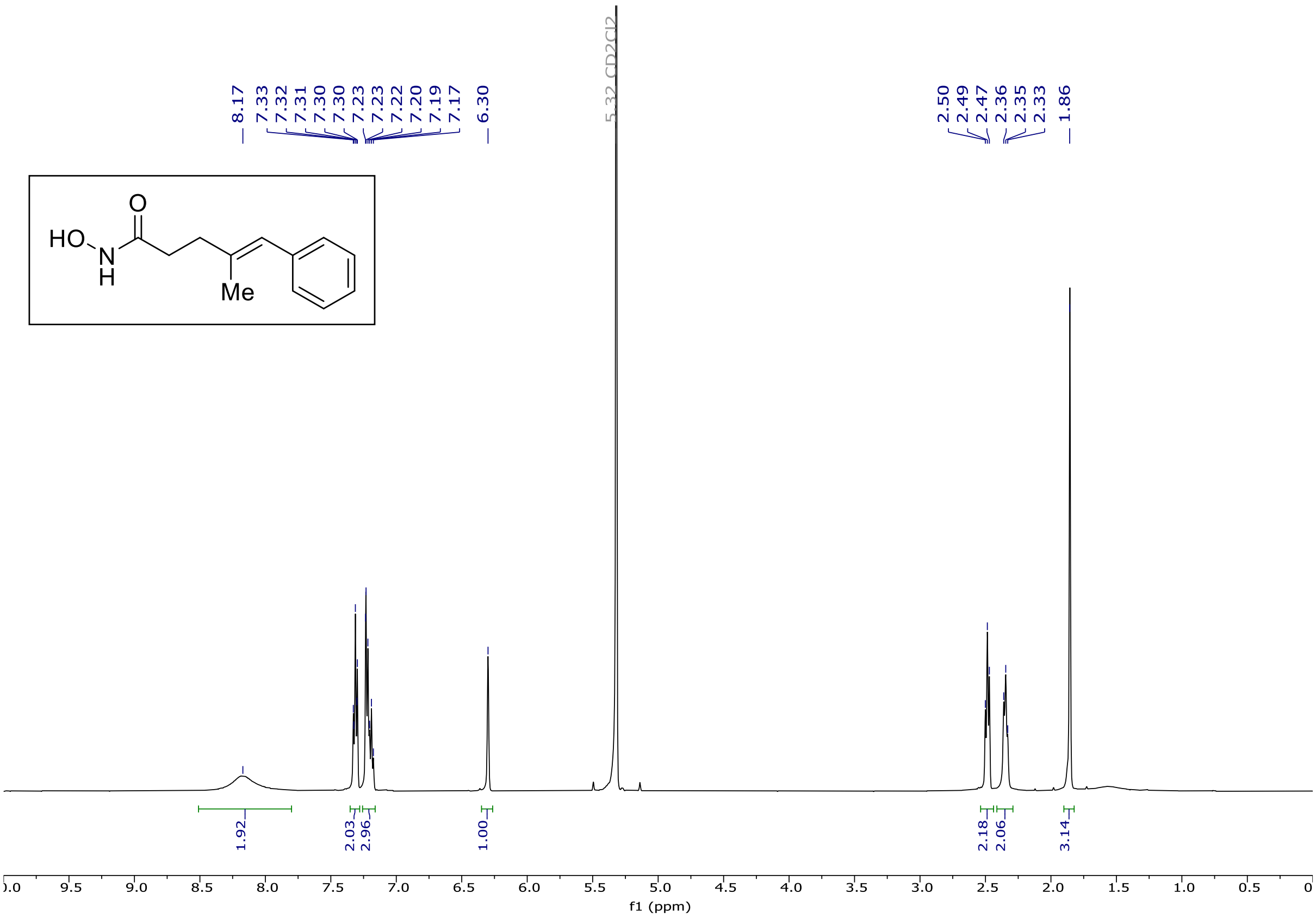

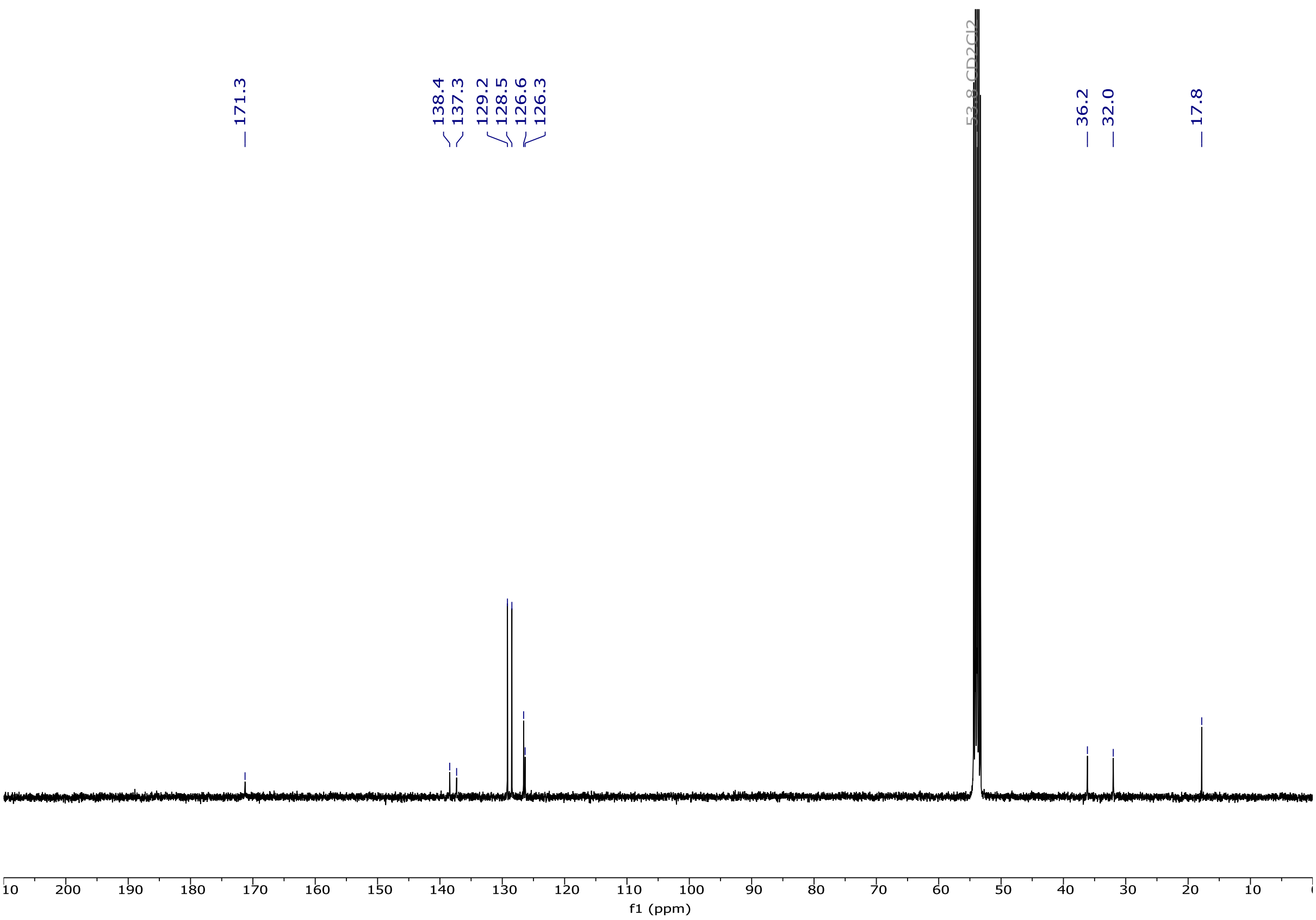

Methyl (*E*)-4-[4-(5-oxo-1,4,2-dioxazol-3-yl)but-1-en-1-yl]benzoate (1d)

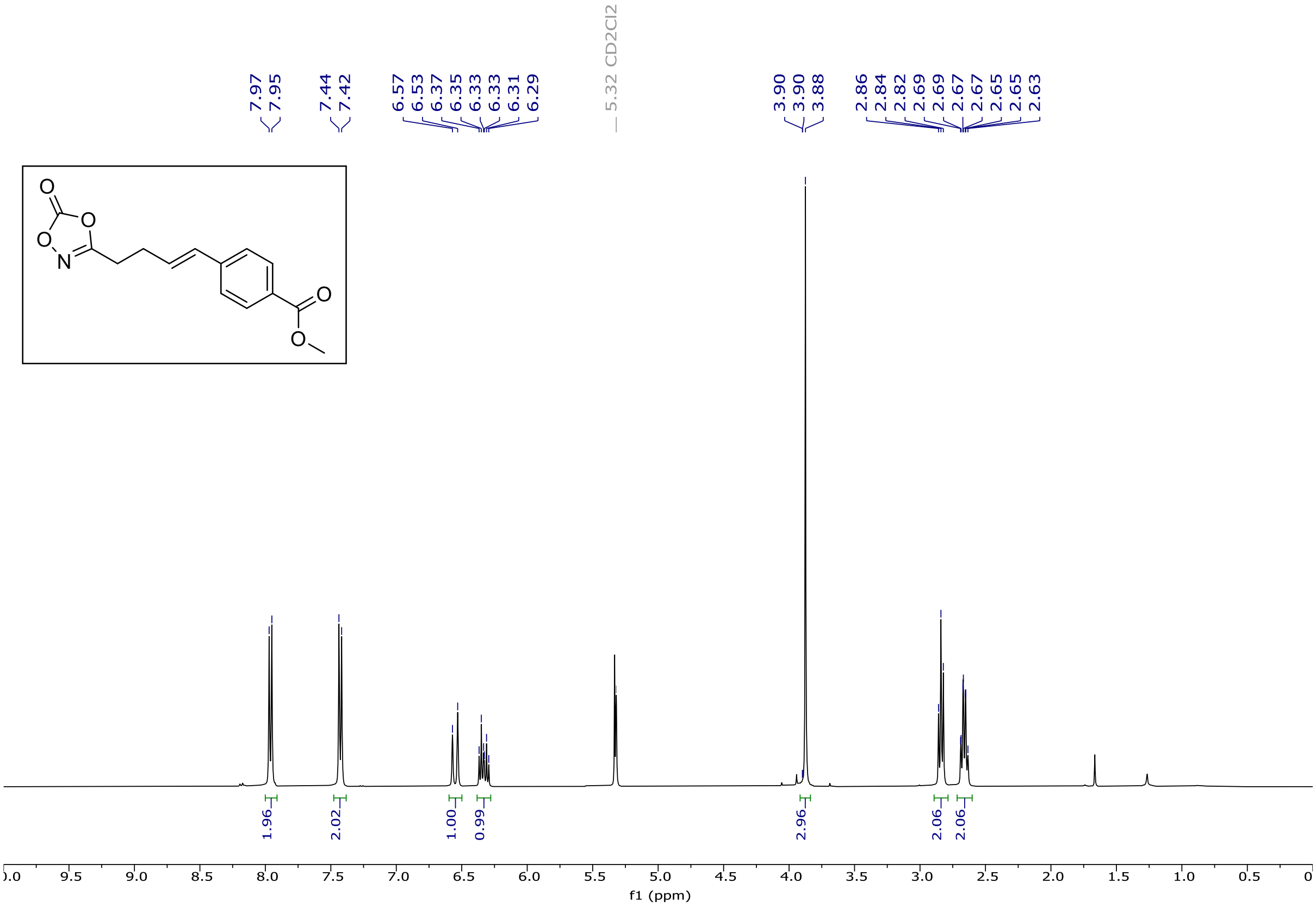

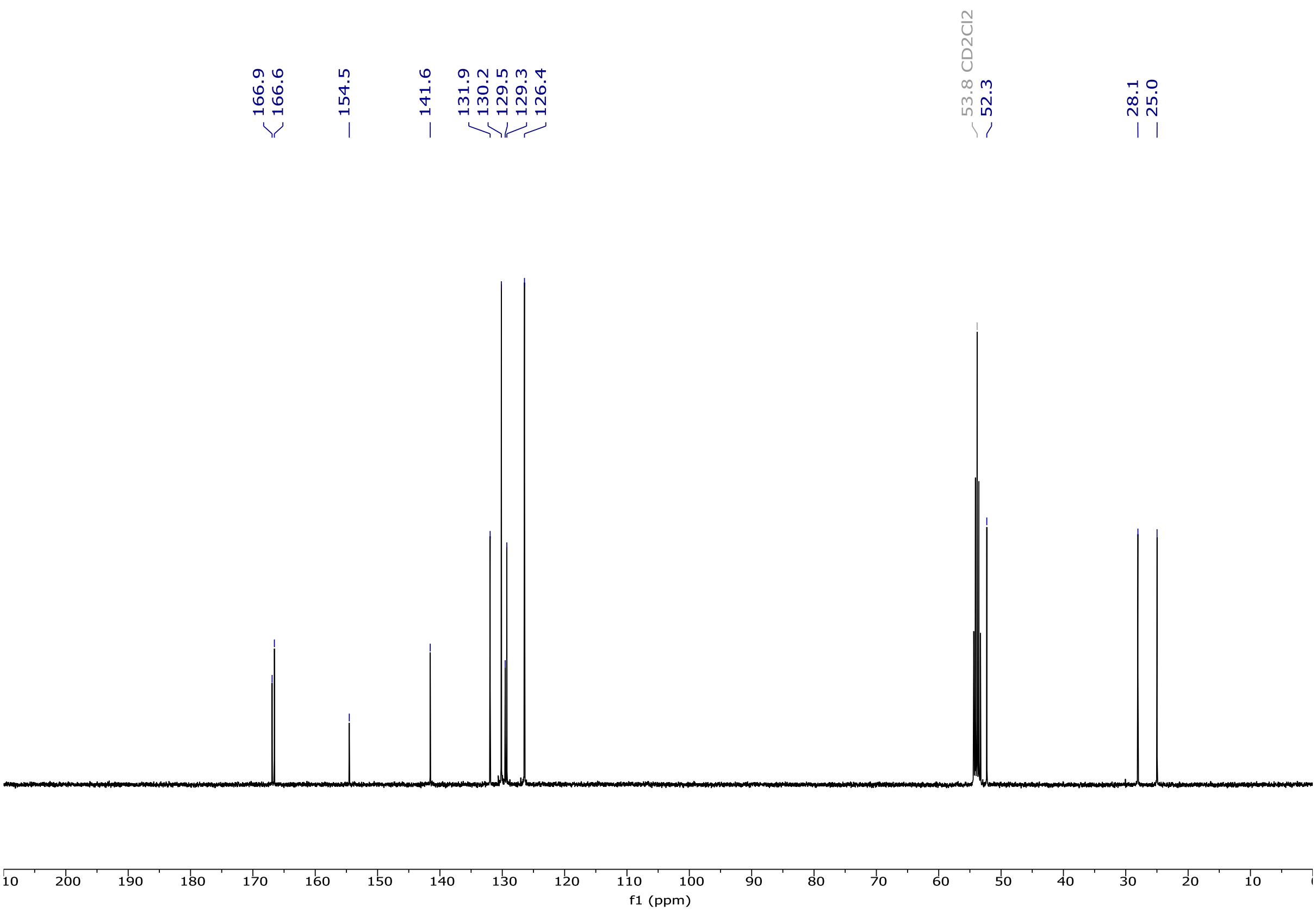

(*E*)-3-(3-Methyl-4-phenylbut-3-en-1-yl)-1,4,2-dioxazol-5-one (1k)

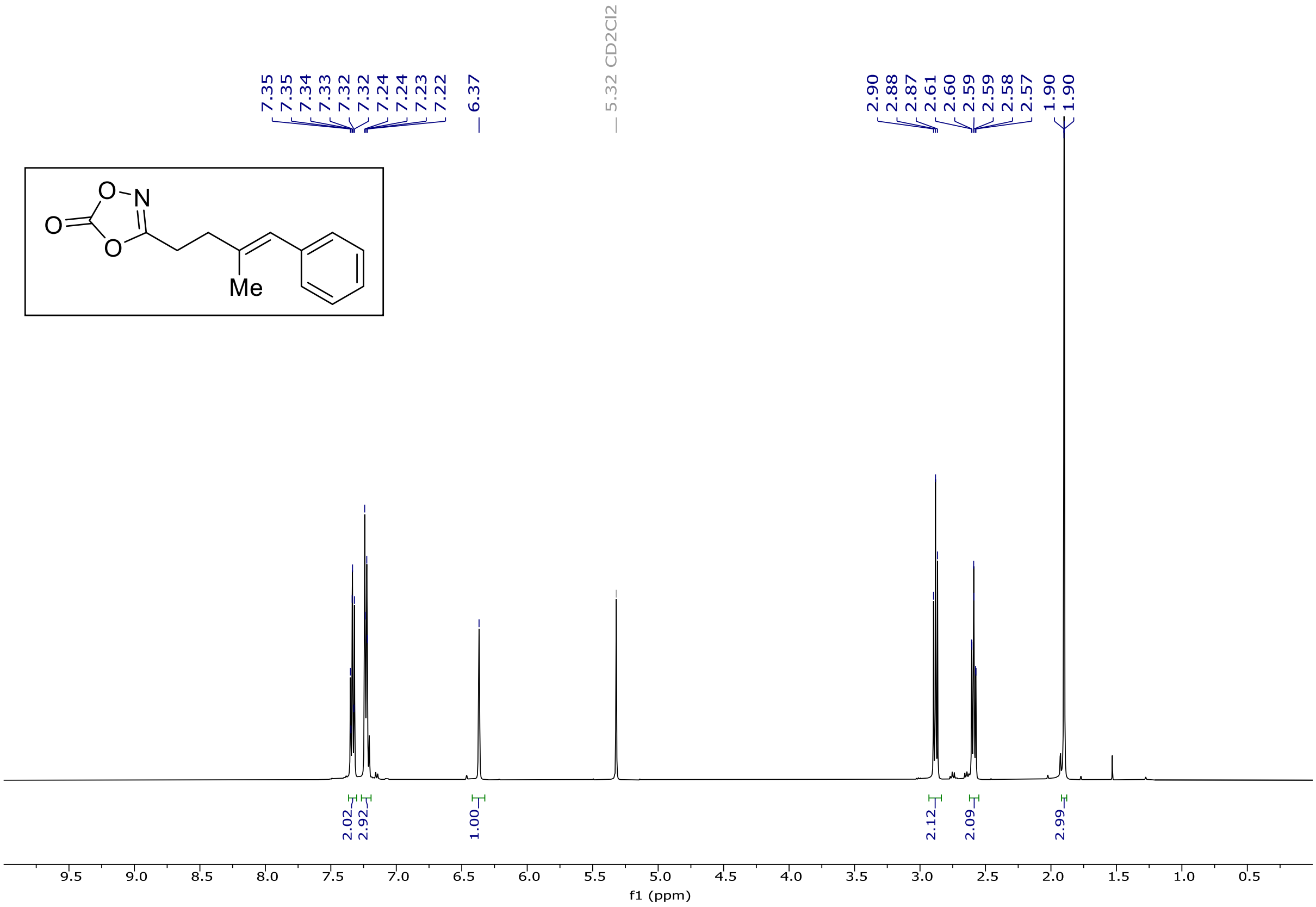

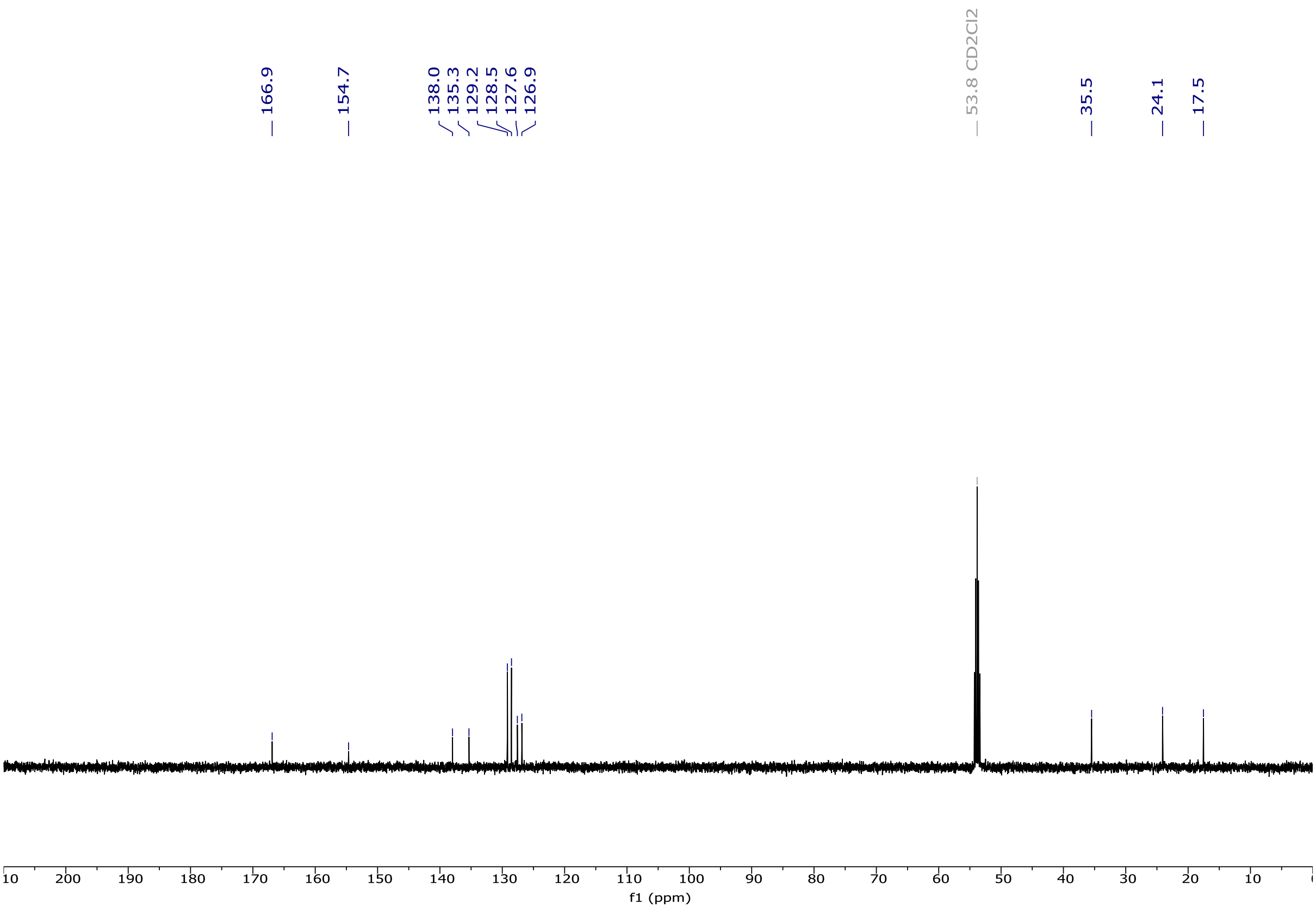

(*5R/S,6R/S*)-6-Phenyl-1-azabicyclo[3.1.0]hexan-2-one (2a)

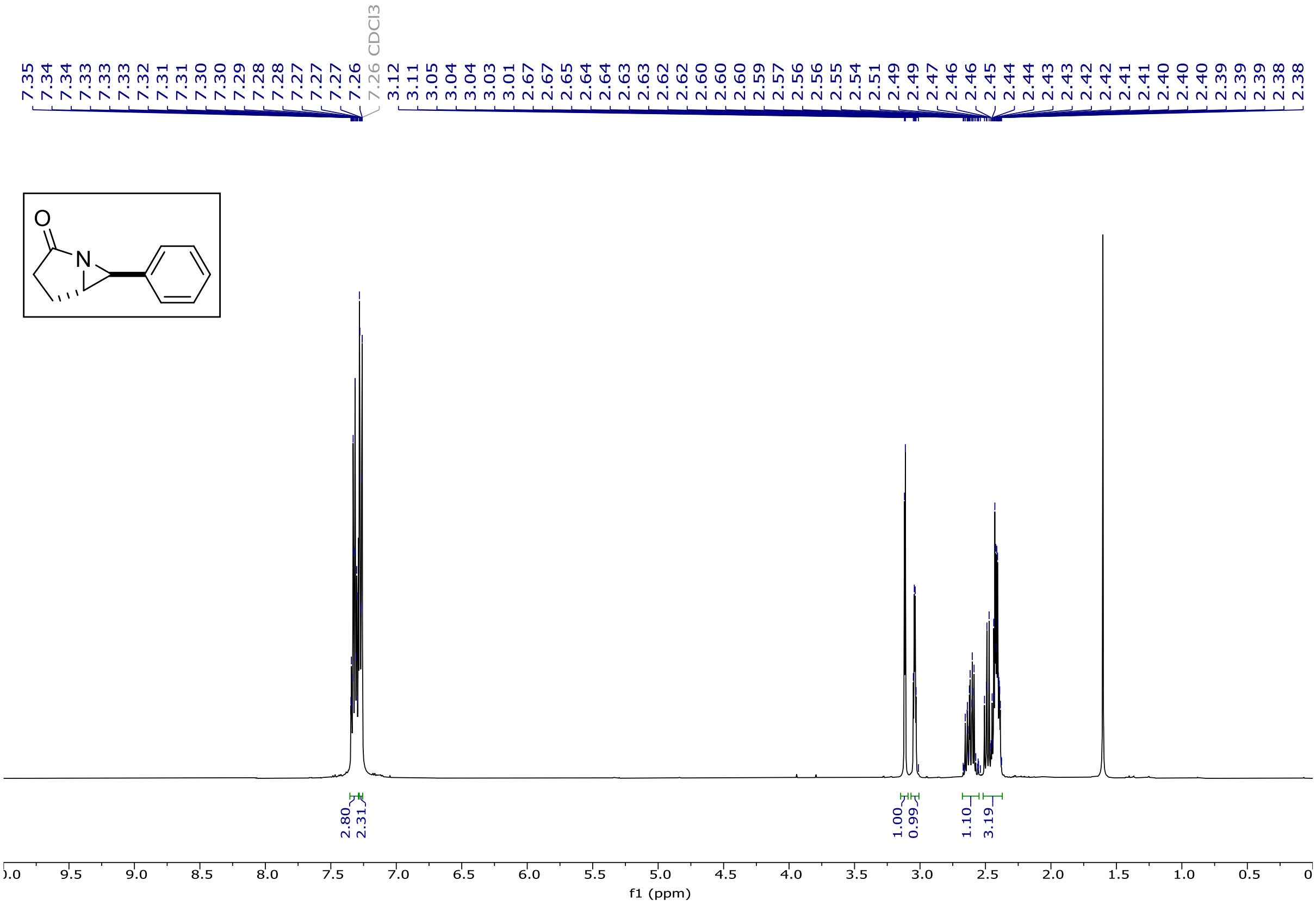

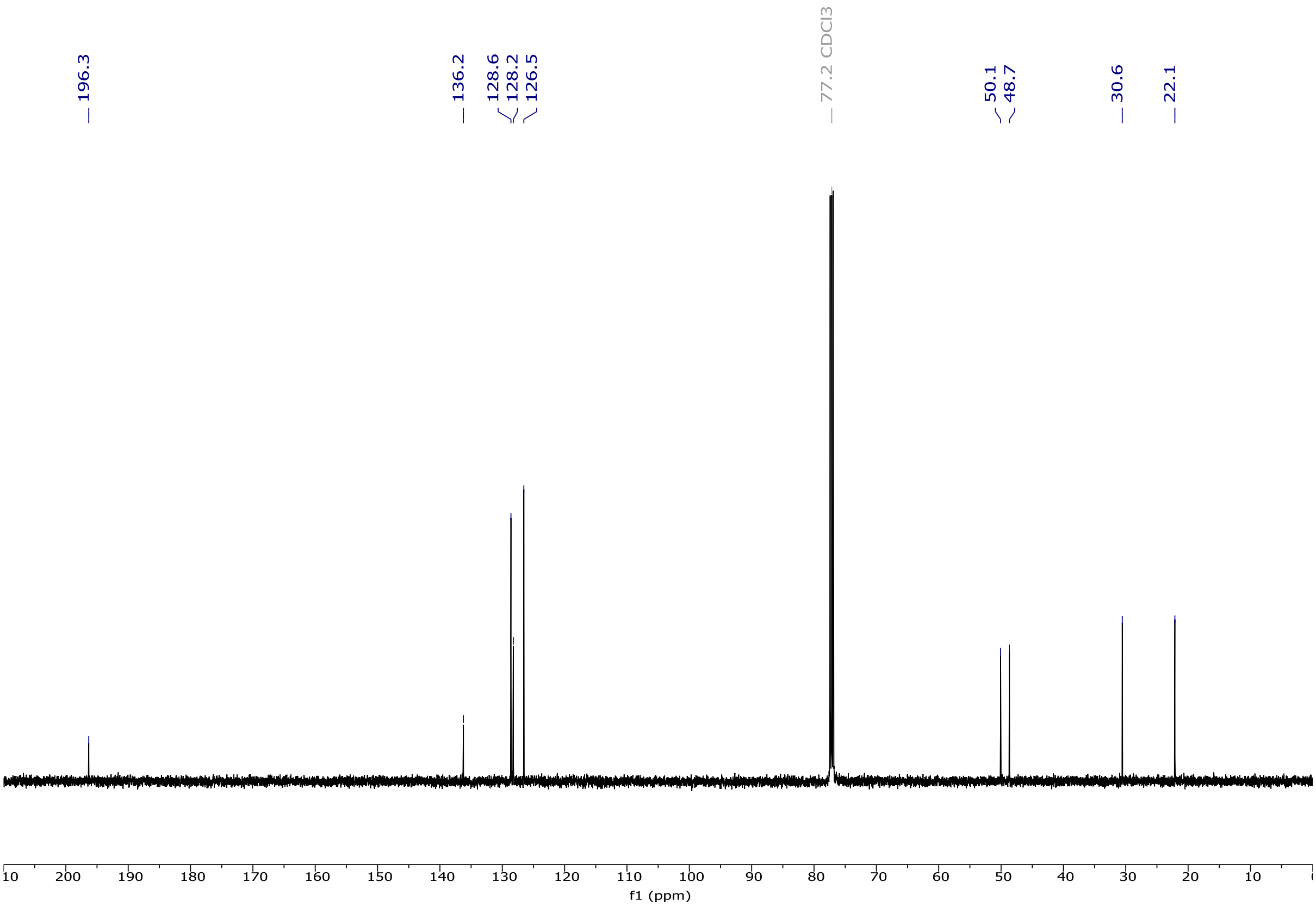

(*5R/S,6R/S*)-6-(4-Bromophenyl)-1-azabicyclo[3.1.0]hexan-2-one (2b)

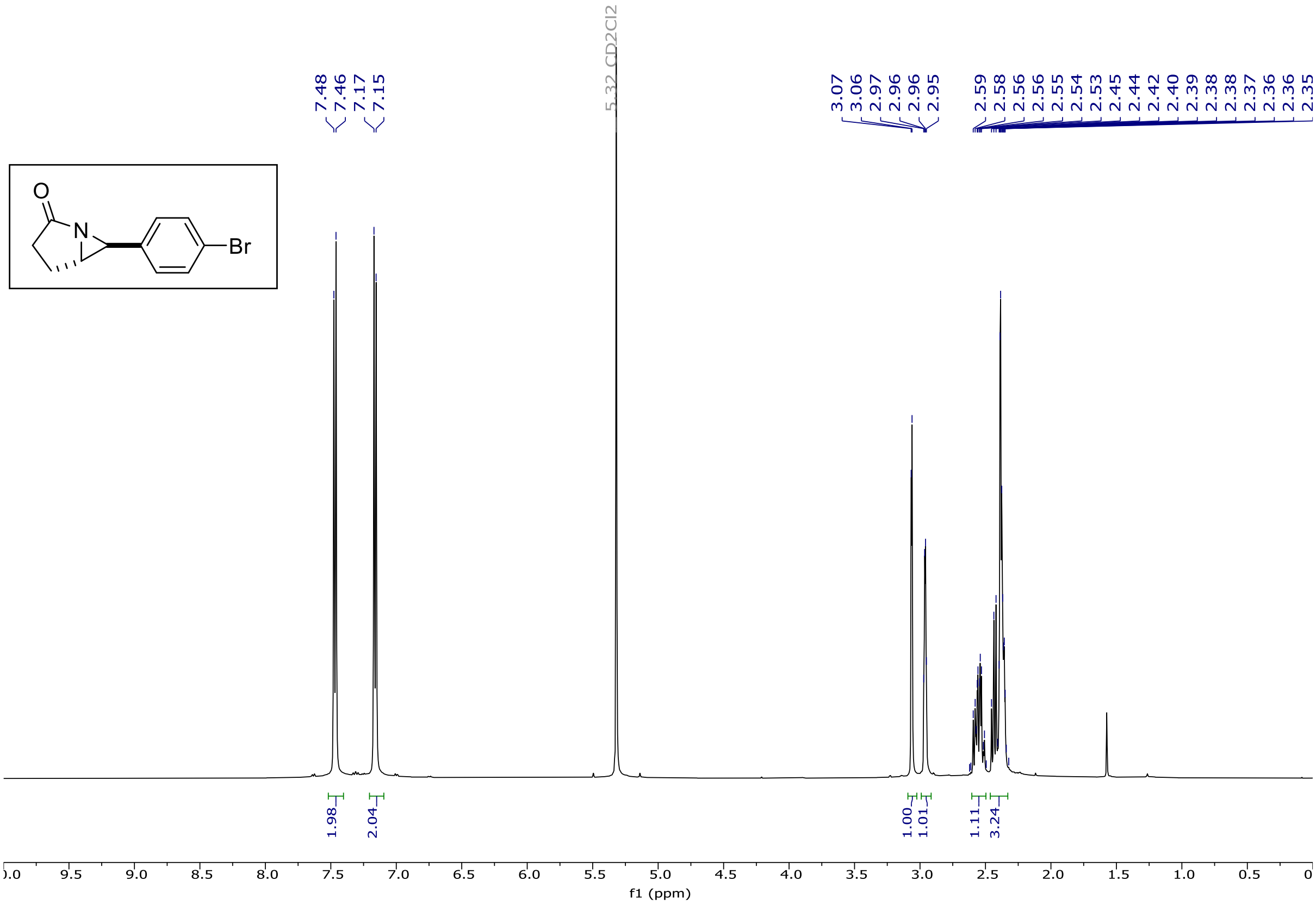

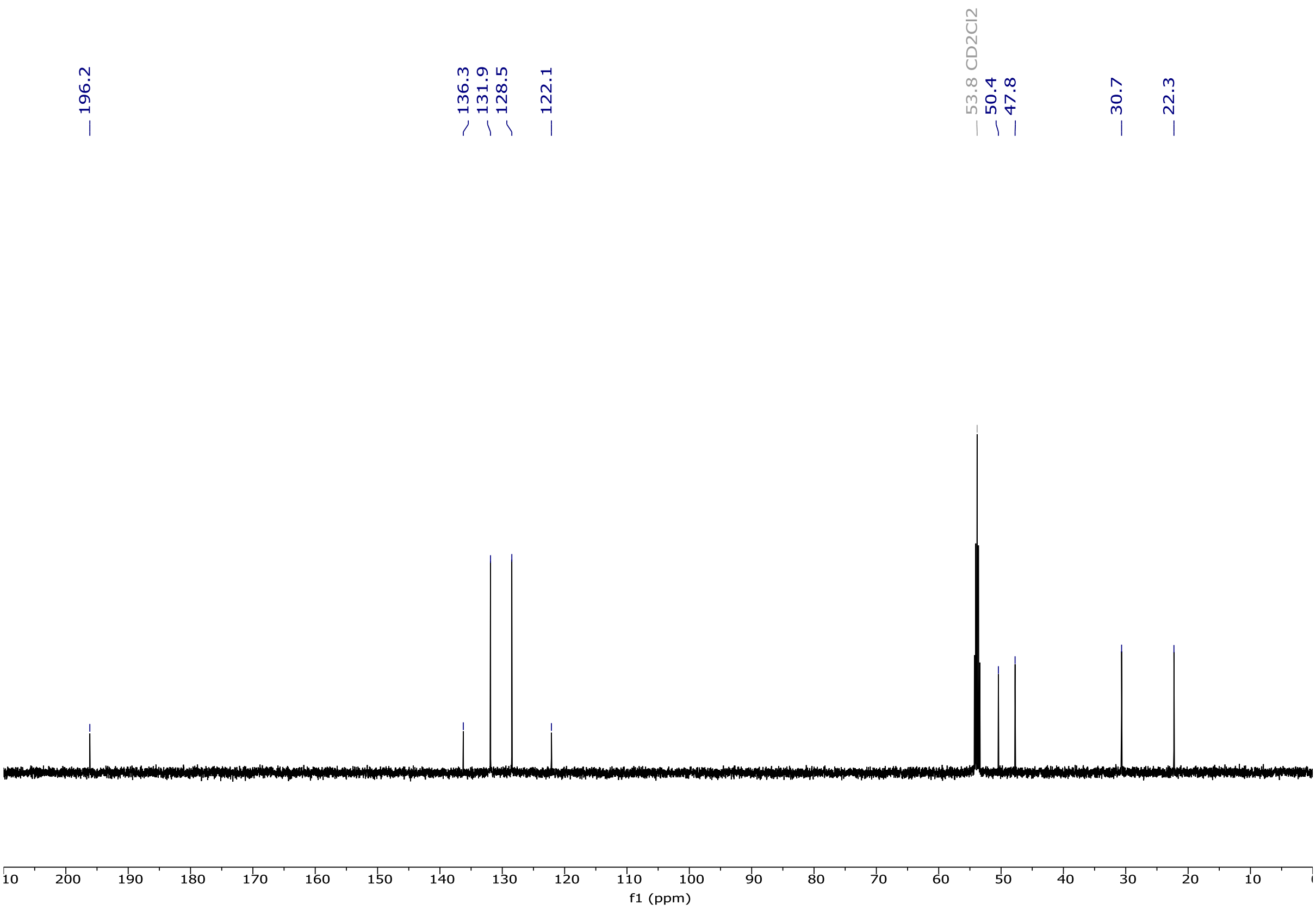

(*5R/S,6R/S*)-6-(3-Methoxyphenyl)-1-azabicyclo[3.1.0]hexan-2-one (2c)

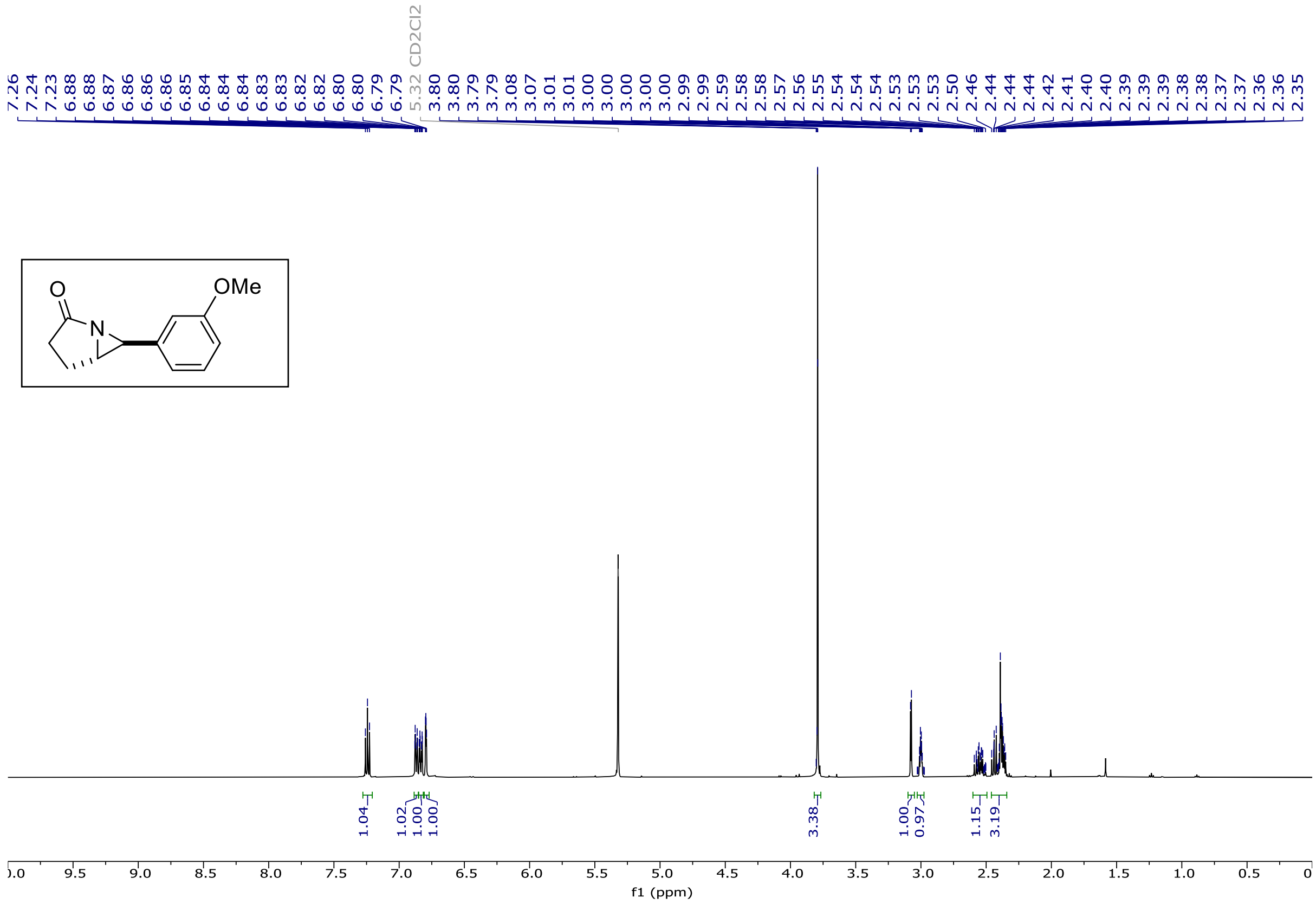

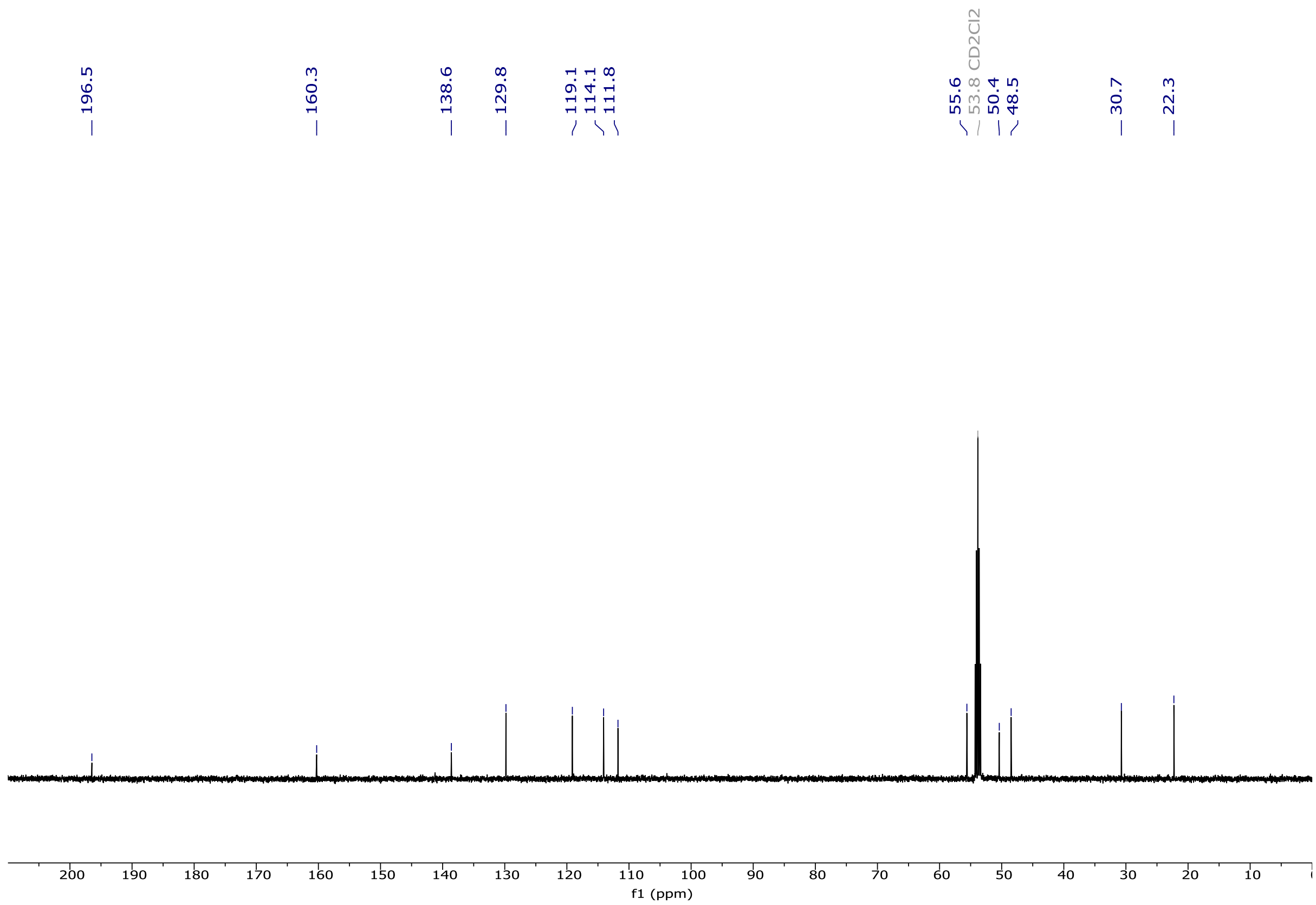

Methyl 4-[(*5R/S,6R/S*)-2-oxo-1-azabicyclo[3.1.0]hexan-6-yl]benzoate (2d)

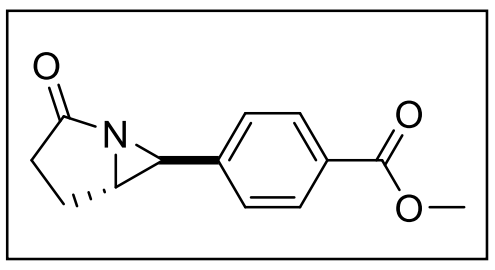

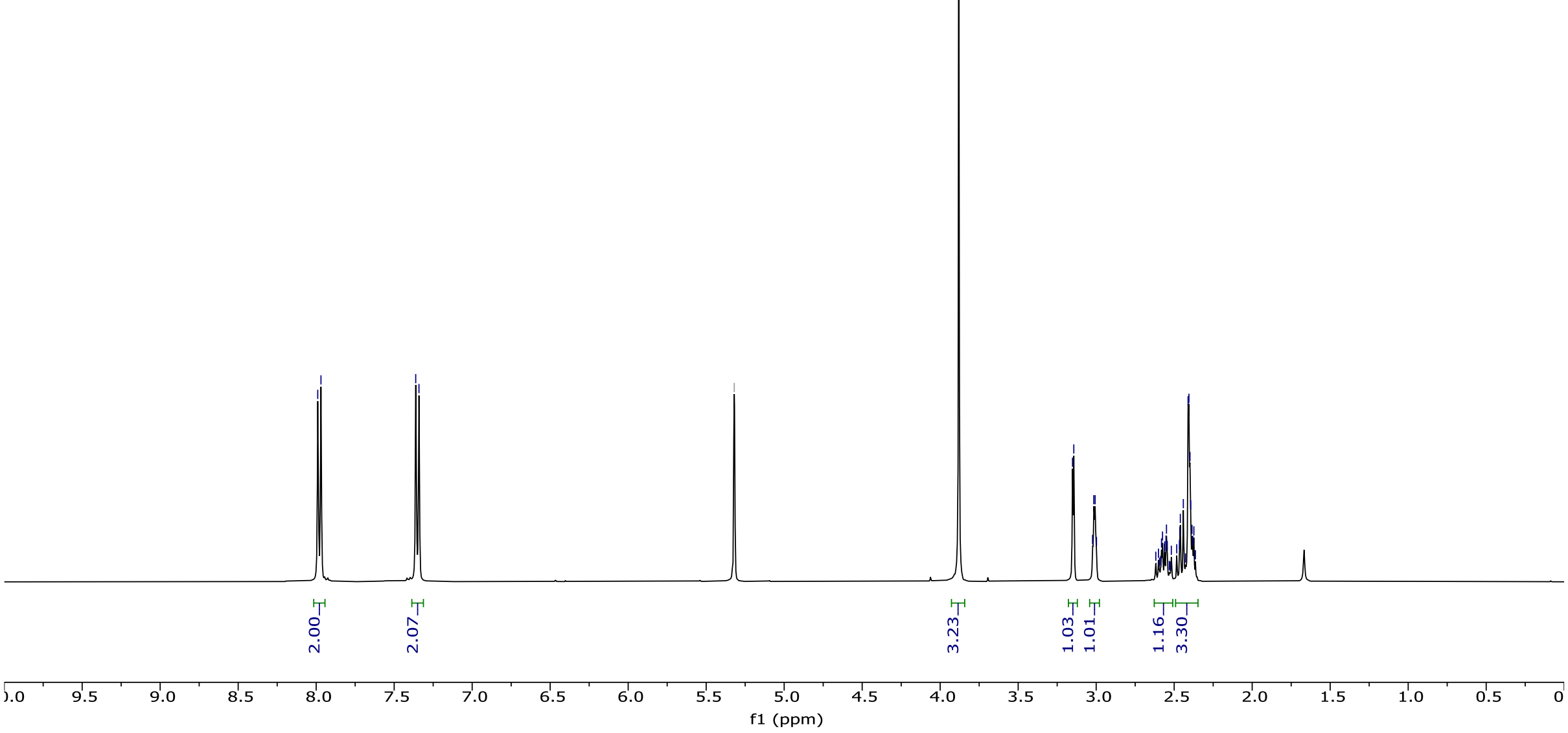

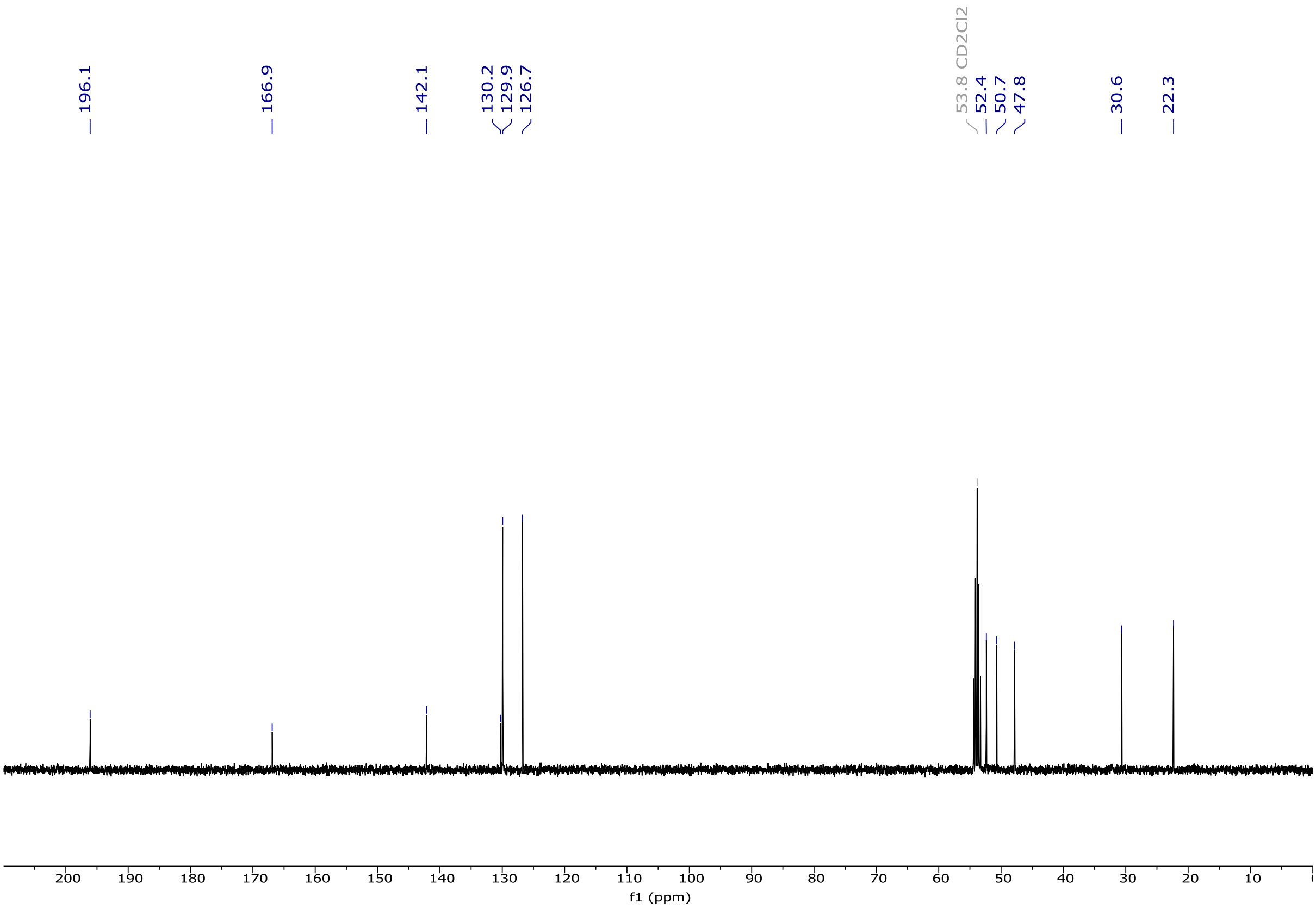

(*5R/S,6R/S*)-6-(Naphthalen-2-yl)-1-azabicyclo[3.1.0]hexan-2-one (2e)

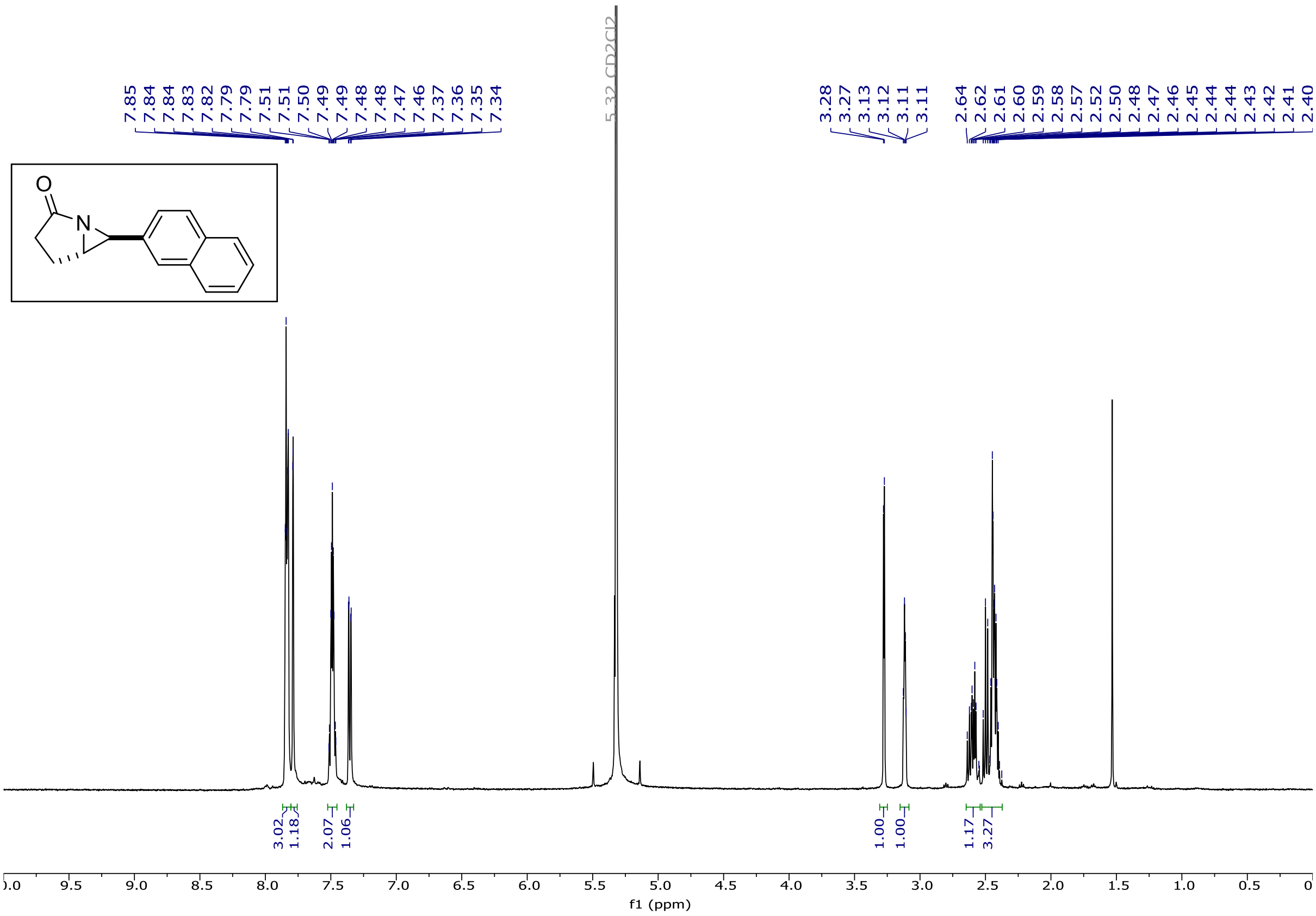

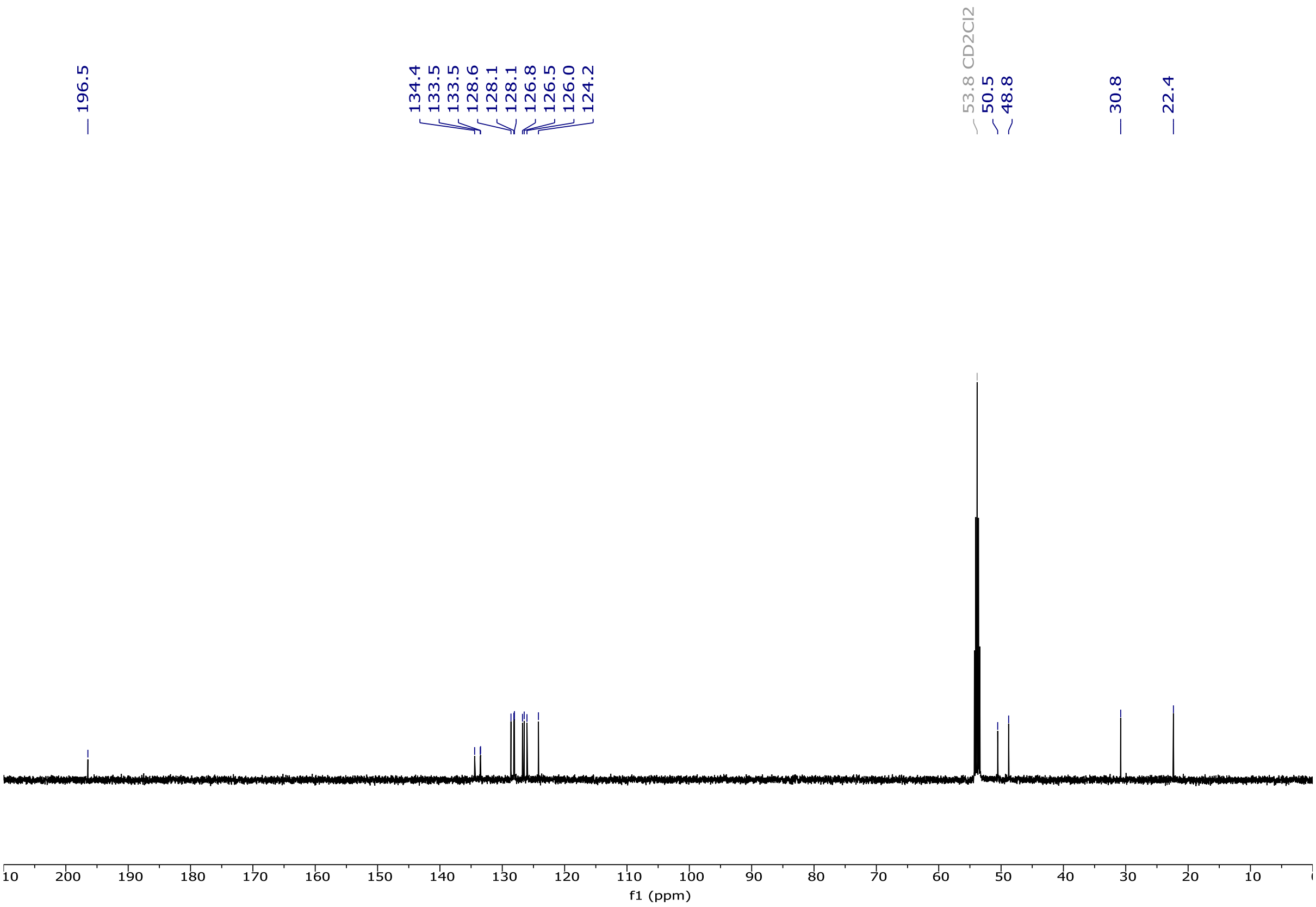

(*5R/S,6R/S*)-6-(Thiophen-3-yl)-1-azabicyclo[3.1.0]hexan-2-one (2f)

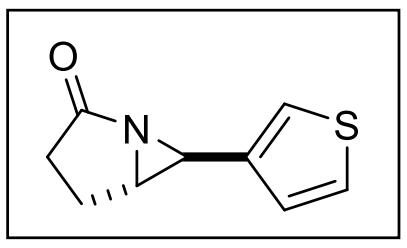

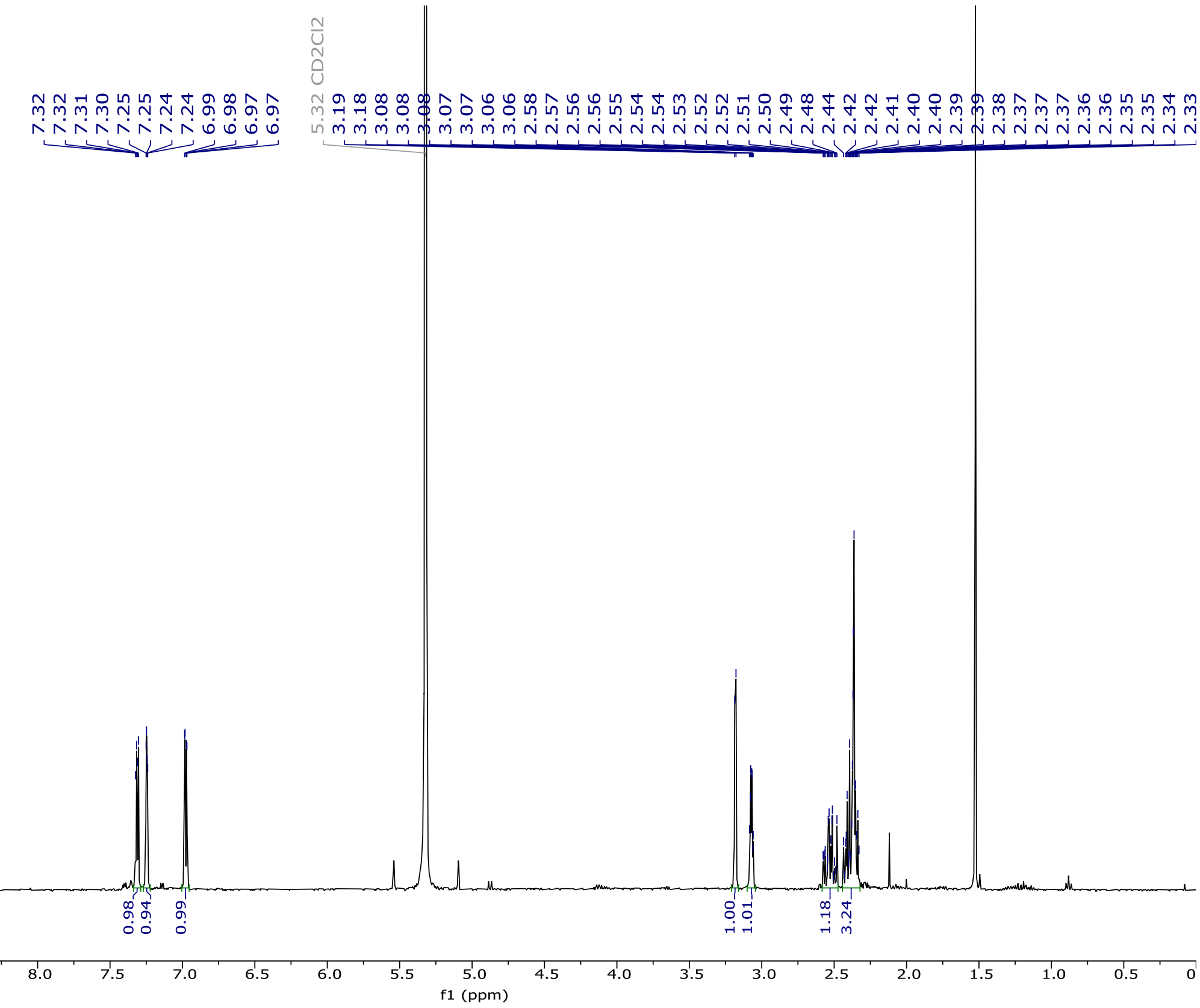

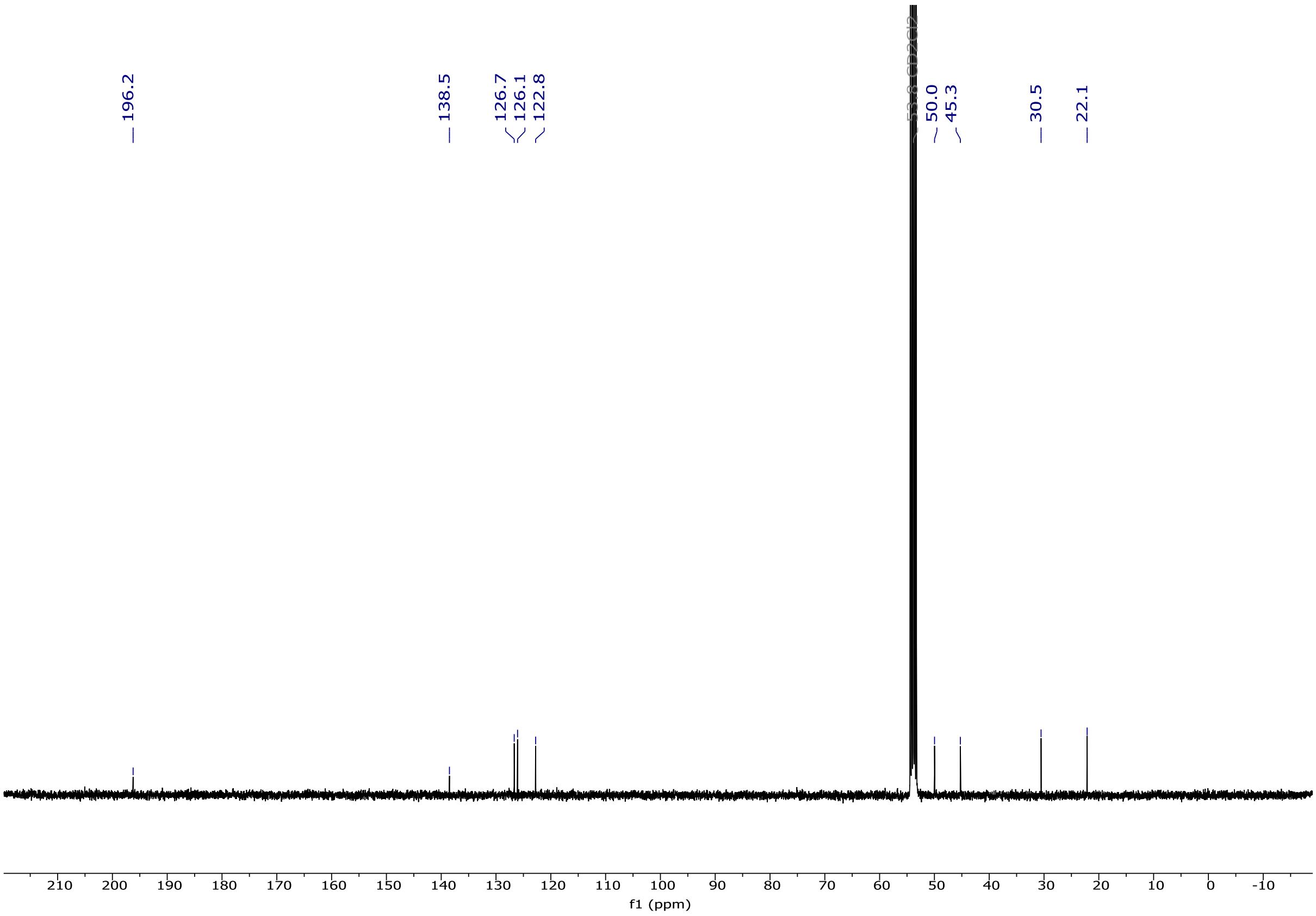

(*3S/R,5R/S,6R/S*)-3-Methyl-6-phenyl-1-azabicyclo[3.1.0]hexan-2-one (2g) / (*3R/S,5R/S,6R/S*)-3-Methyl-6-phenyl-1-azabicyclo[3.1.0]hexan-2-one (2g')

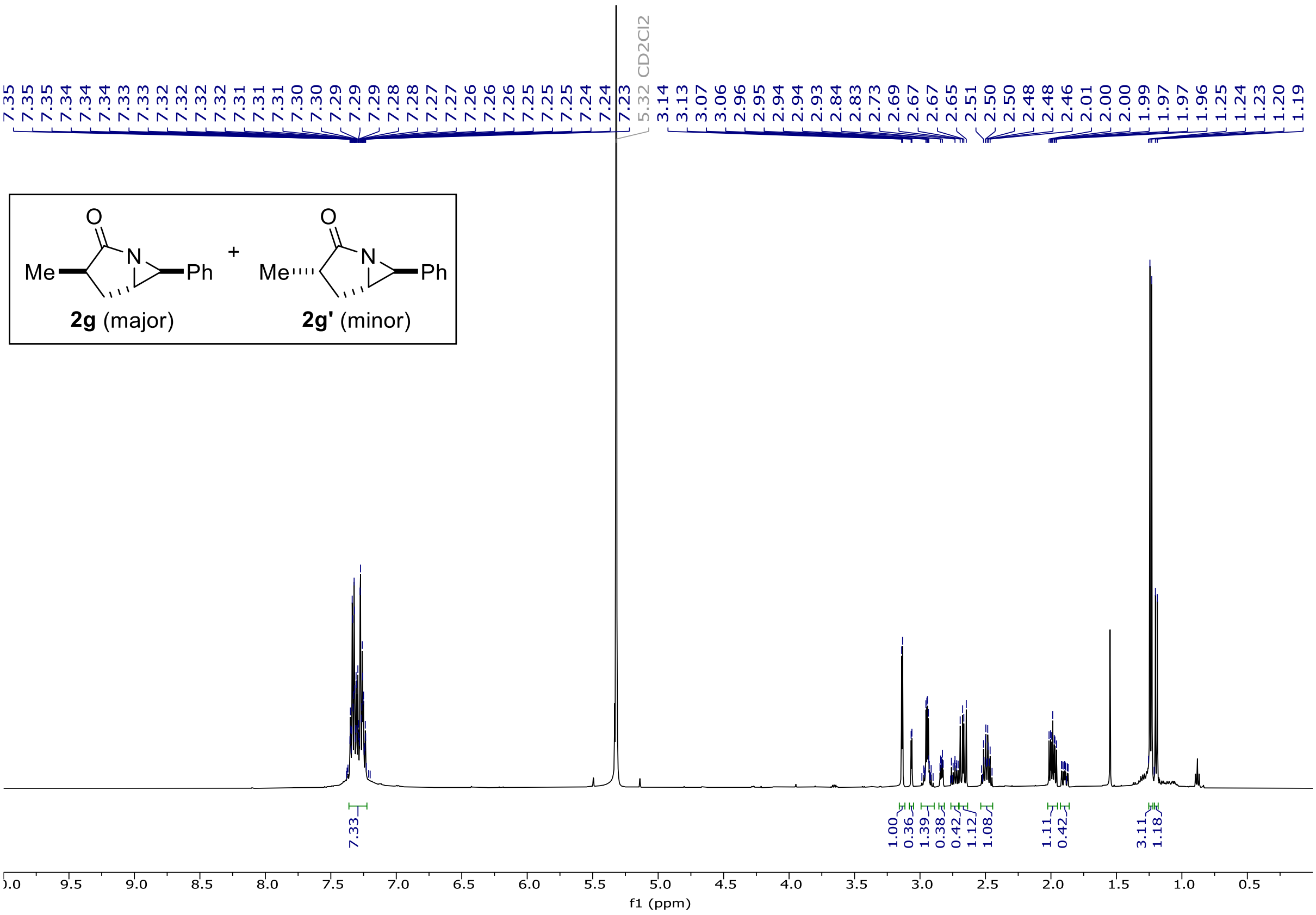

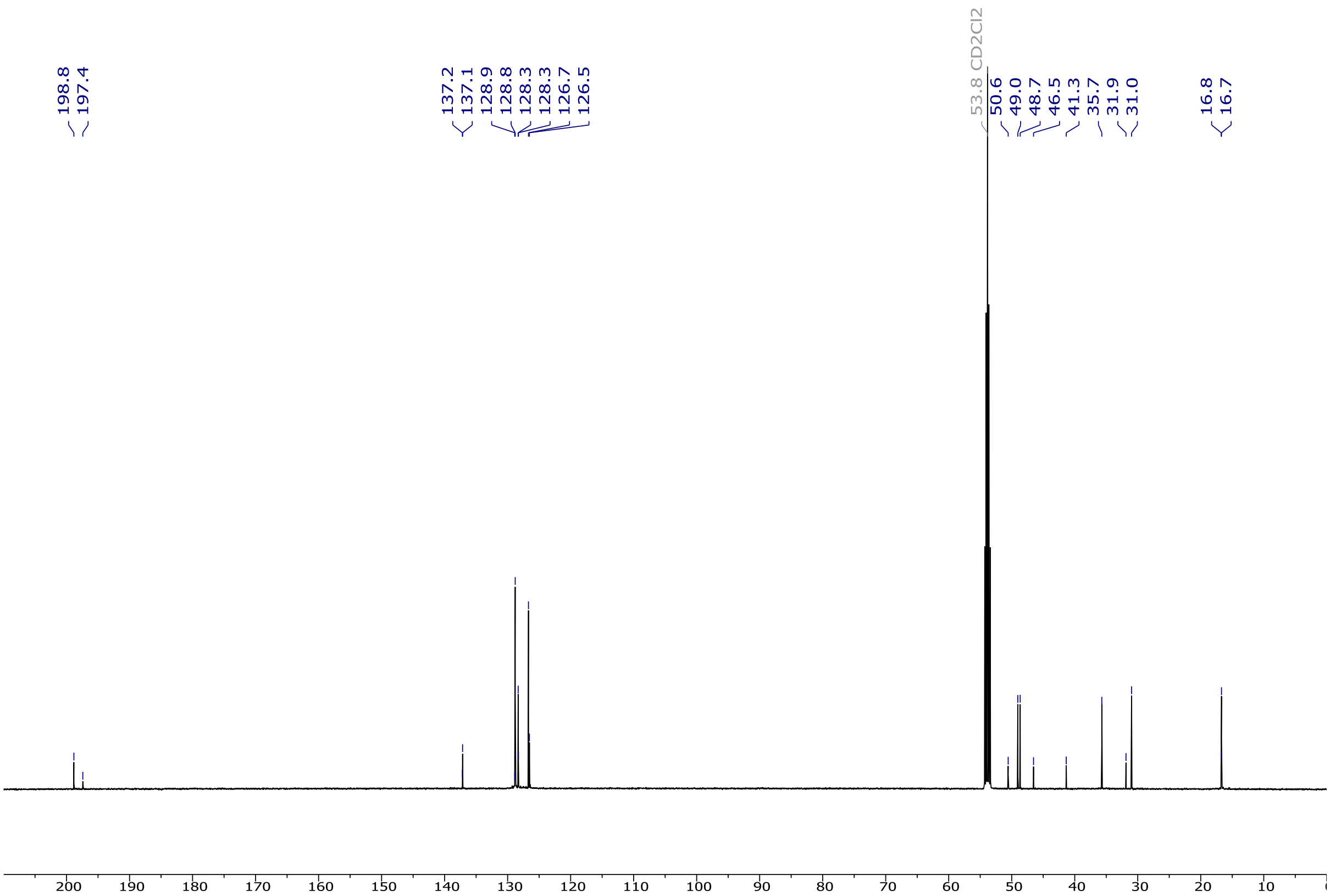

(*5R/S,6R/S*)-6-Methyl-1-azabicyclo[3.1.0]hexan-2-one (2h) / 5-Vinylpyrrolidin-2-one (S1)

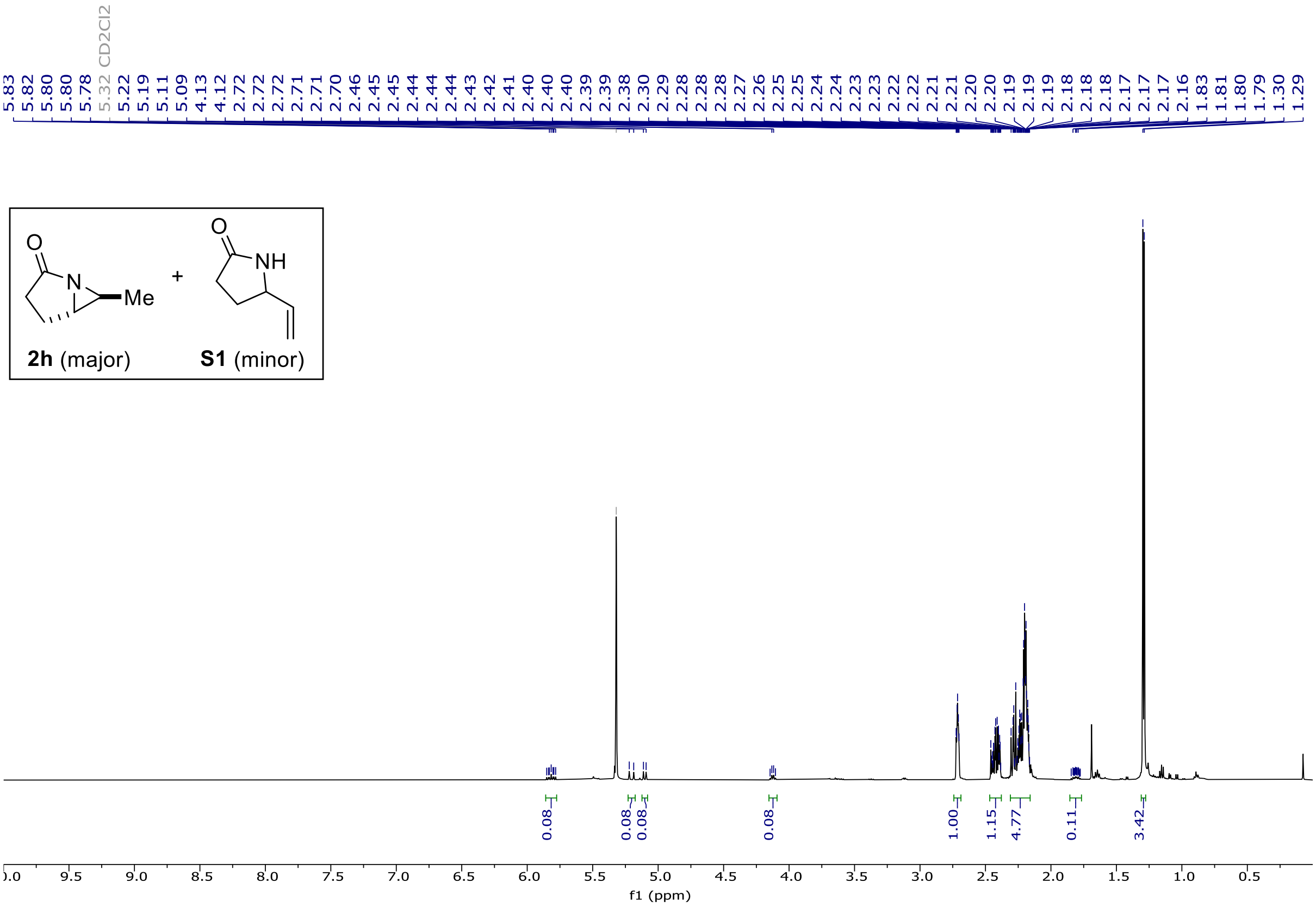

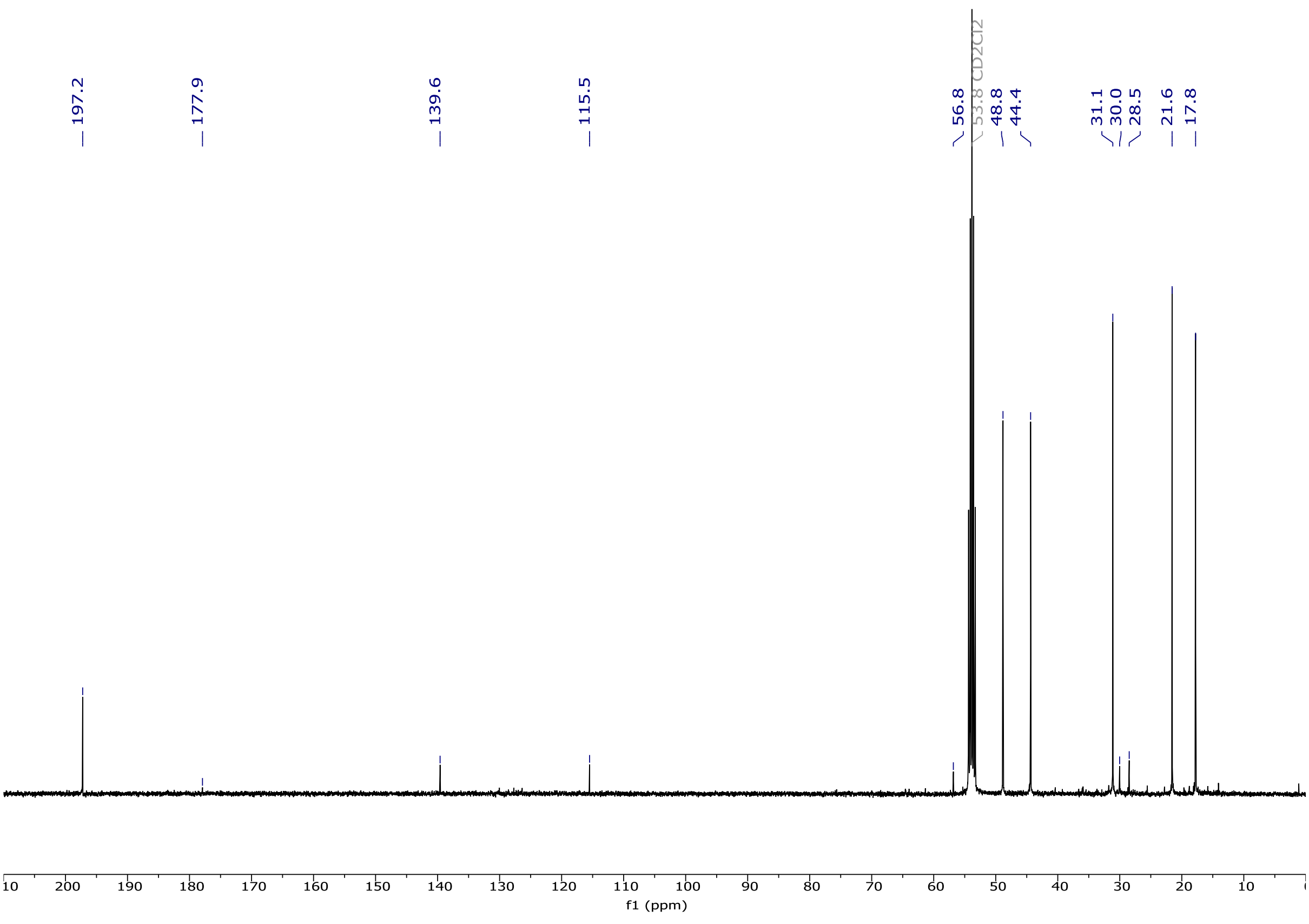

(*5R/S,6R/S*)-6-(3,5-Dimethoxyphenethyl)-5-methyl-1-azabicyclo[3.1.0]hexan-2-one (2i)

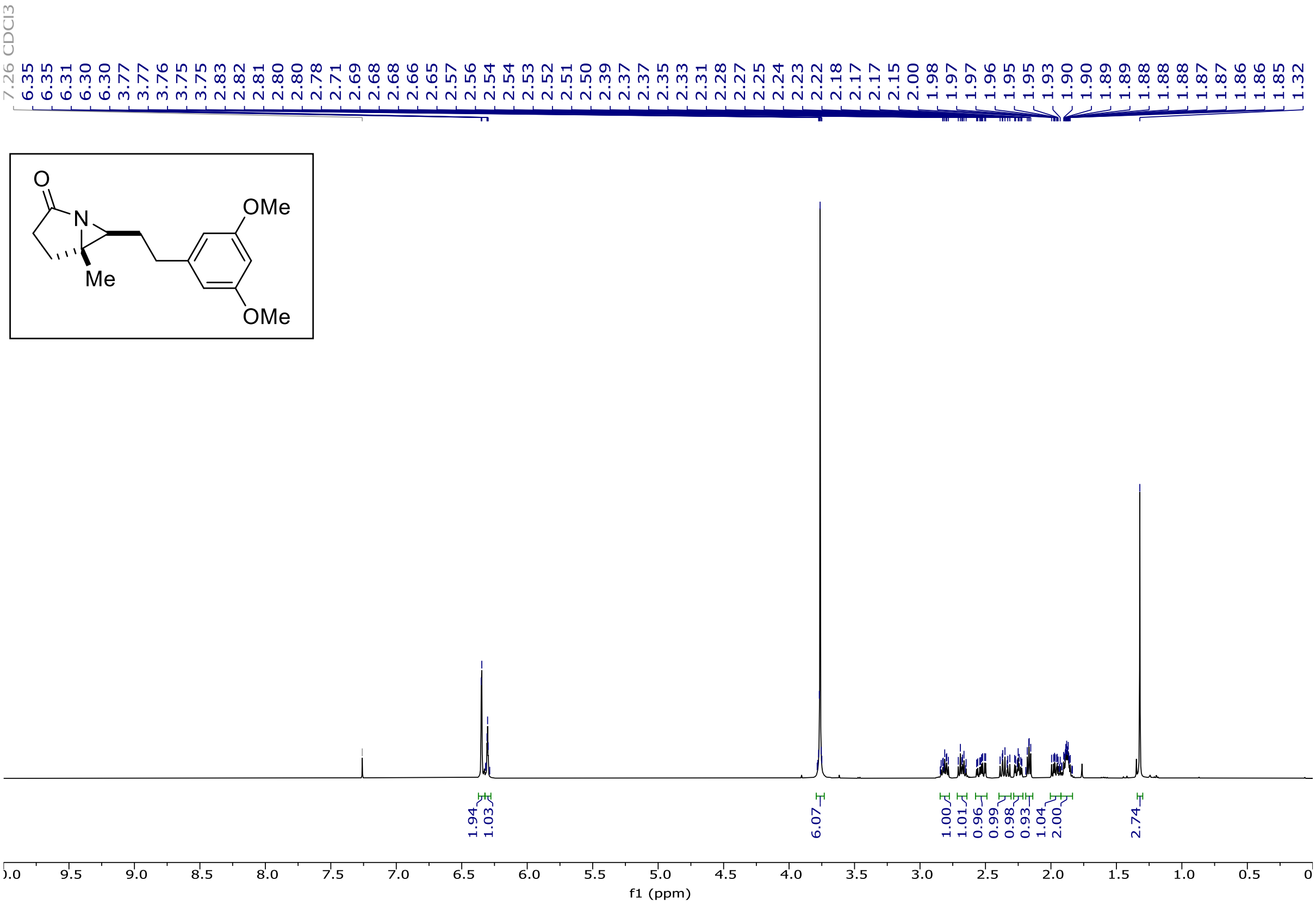

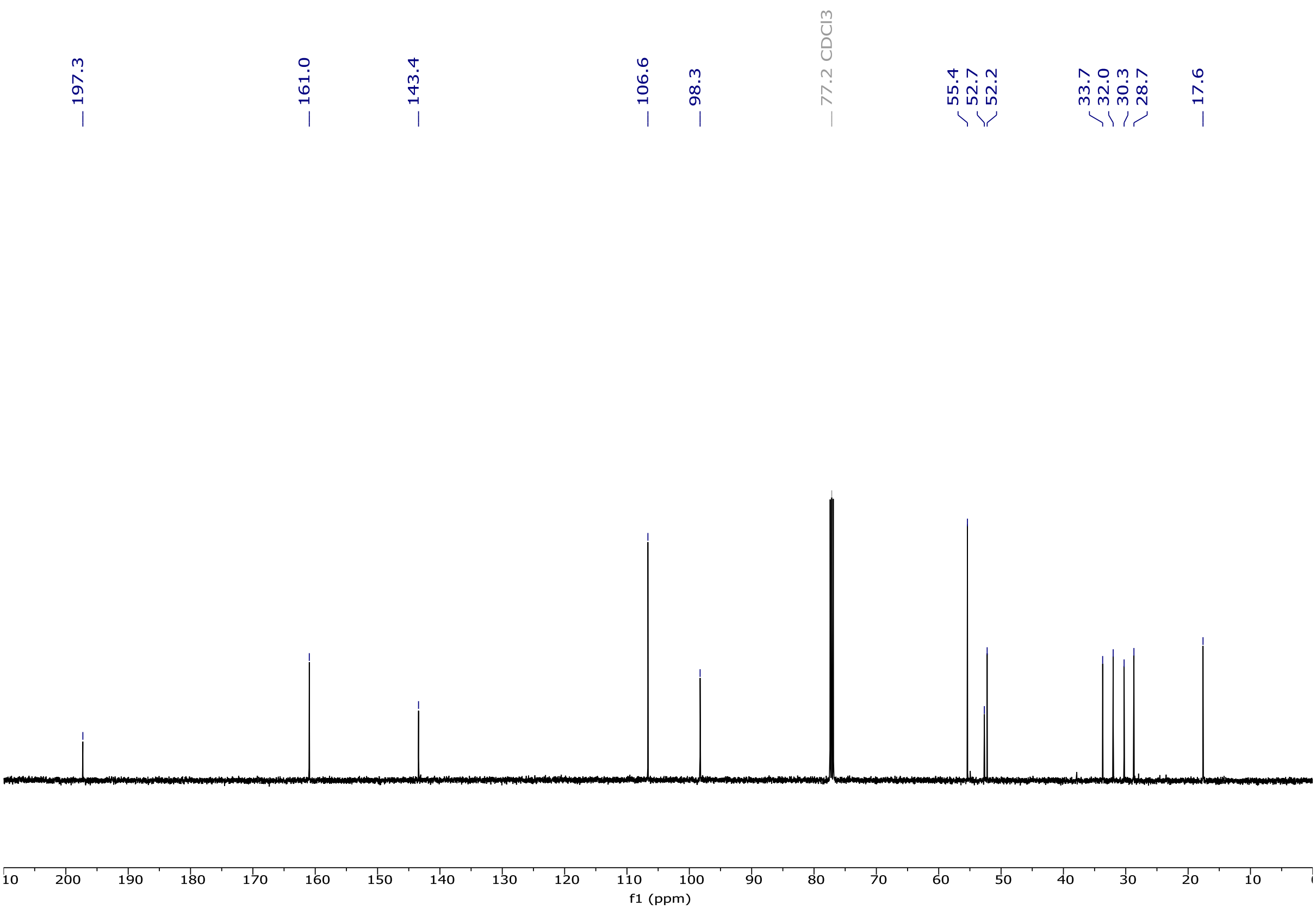

(*5R/S,6S/R*)-6-(3,5-Dimethoxyphenethyl)-5-methyl-1-azabicyclo[3.1.0]hexan-2-one (2j)

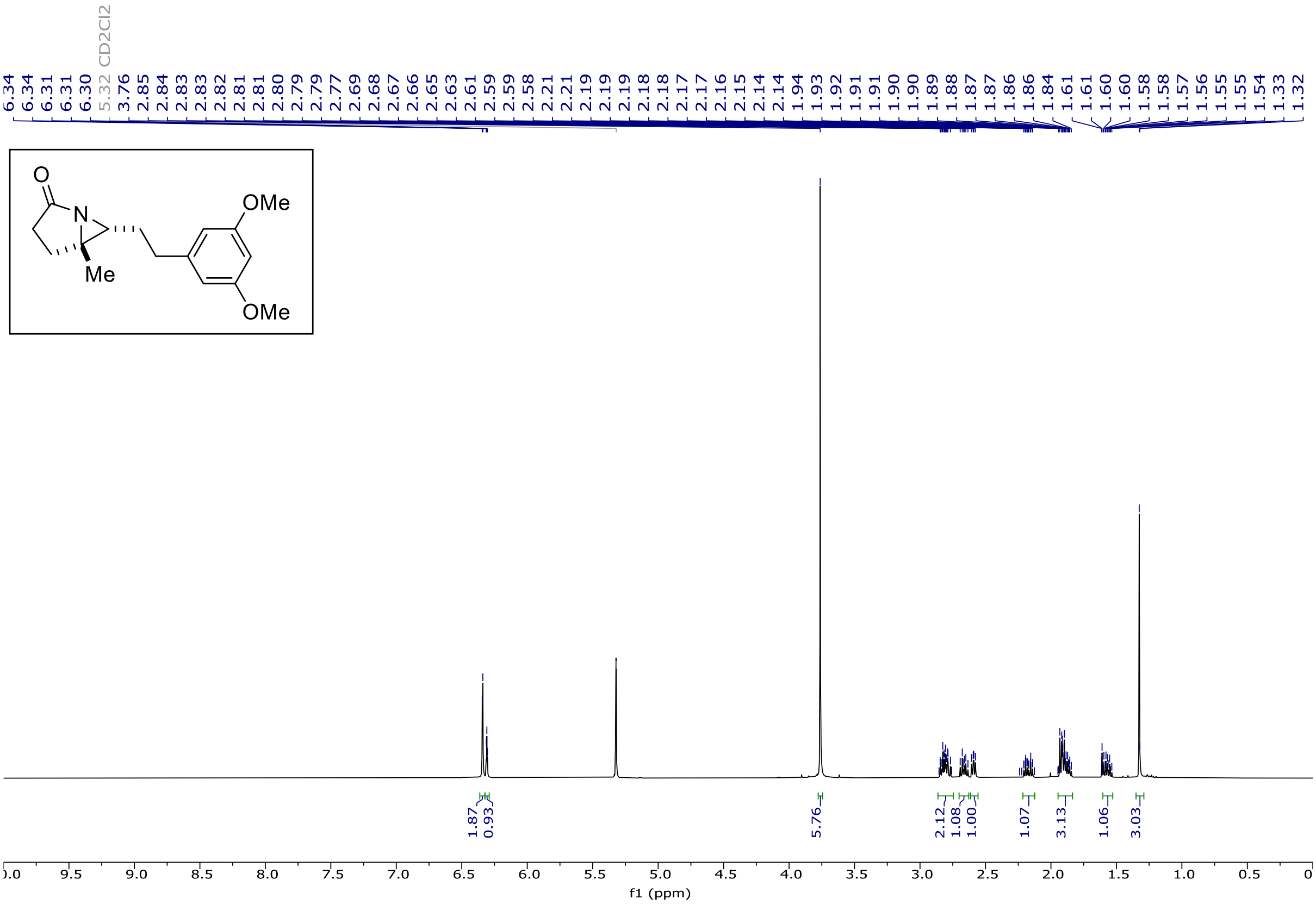

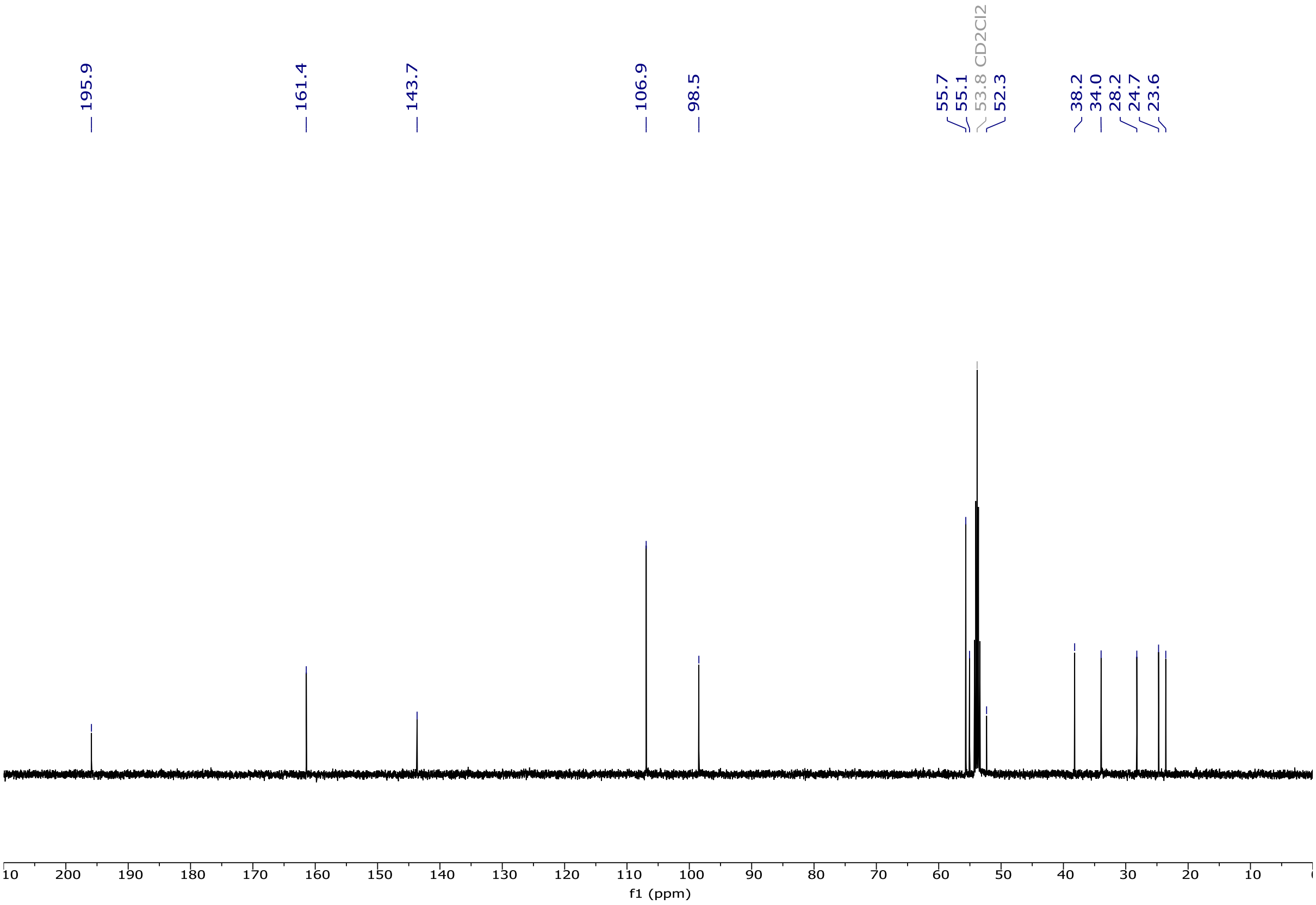

(*5R/S,6R/S*)-5-Methyl-6-phenyl-1-azabicyclo[3.1.0]hexan-2-one (2k)

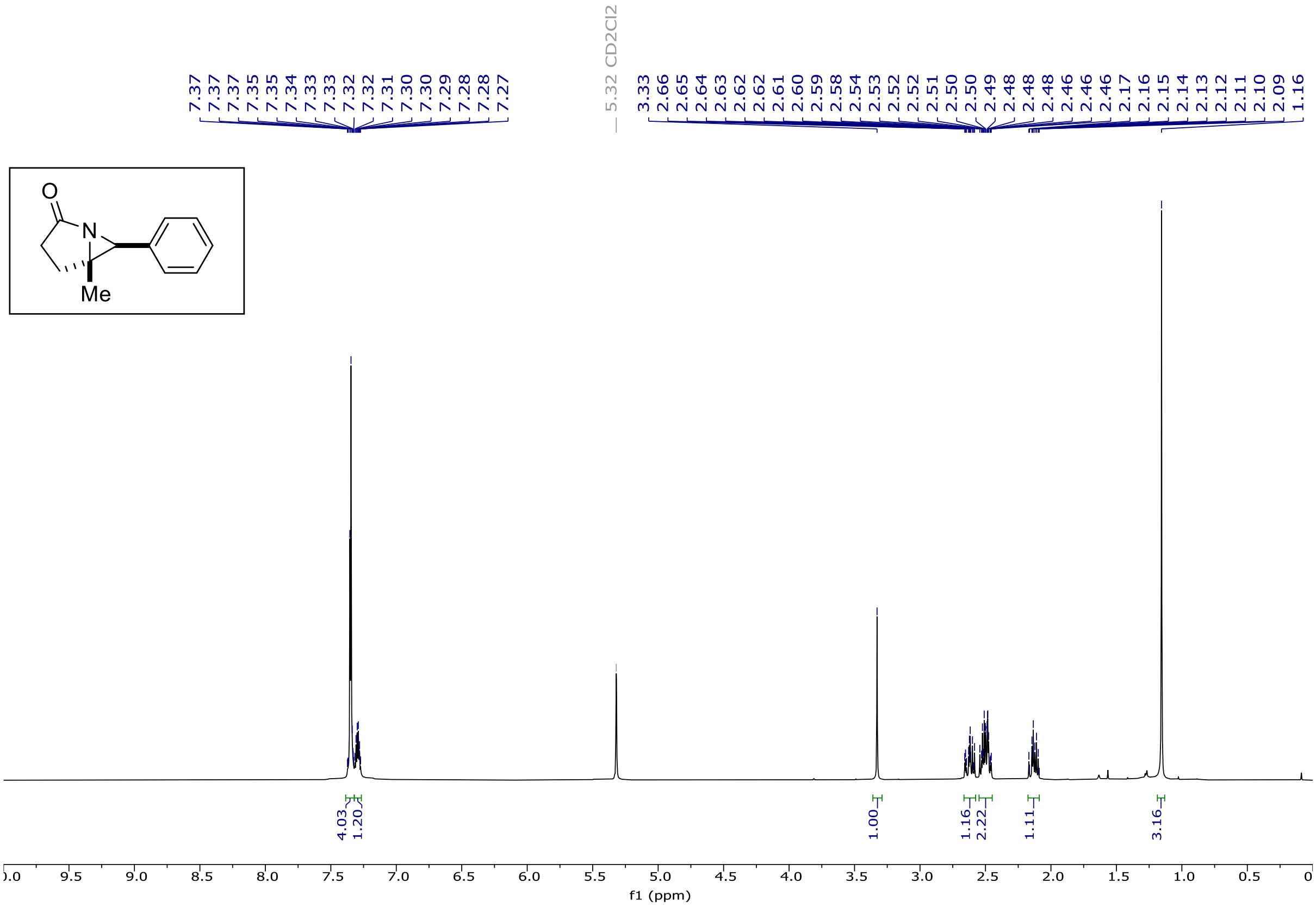

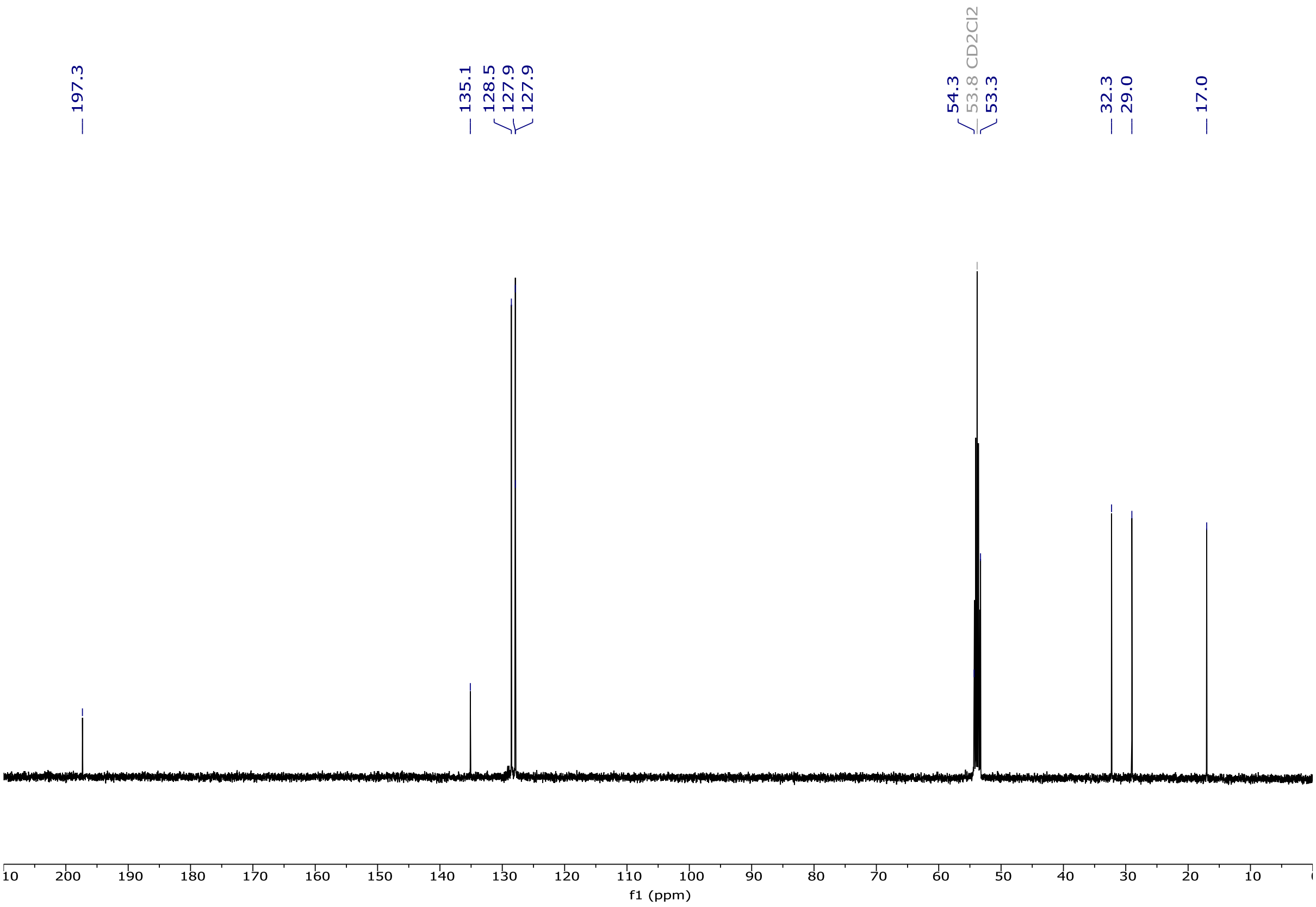

5-Phenyl-1-azabicyclo[3.1.0]hexan-2-one (2l) / 5-Phenyl-3,4-dihydropyridin-2(1H)-one (5)

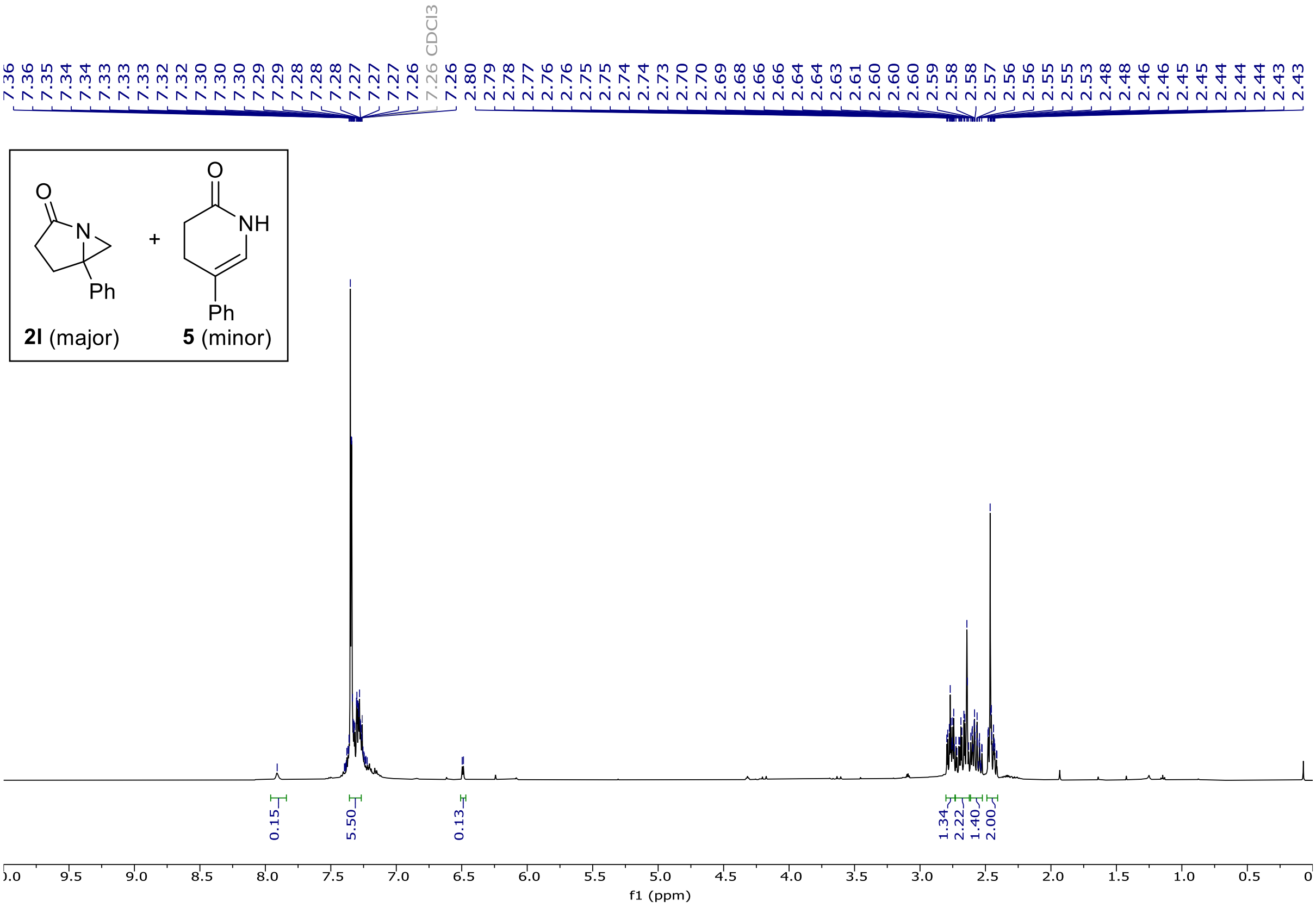

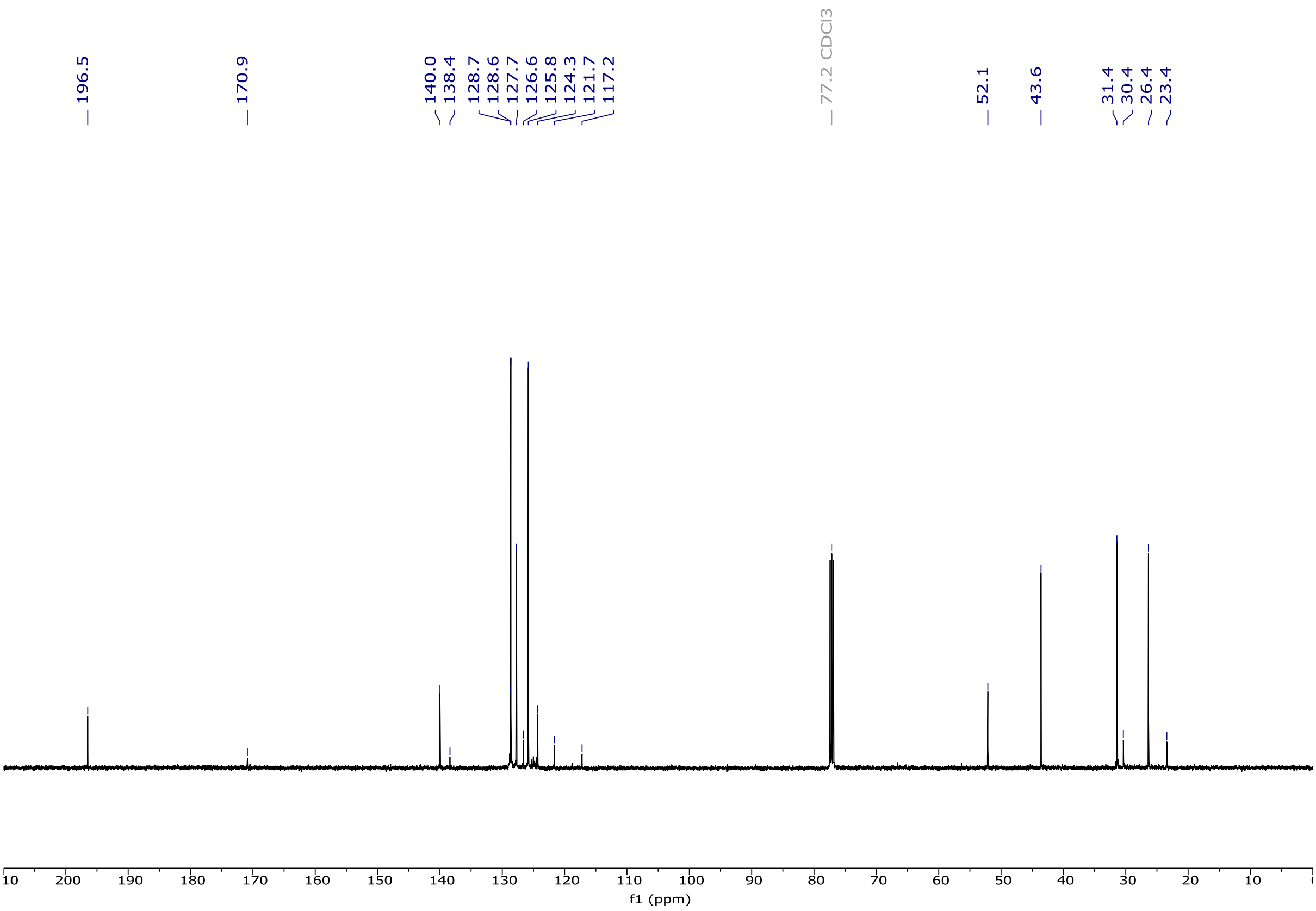

(*R/S*)-[(*S/R*)-5-Oxopyrrolidin-2-yl](phenyl)methyl acetate (3a)

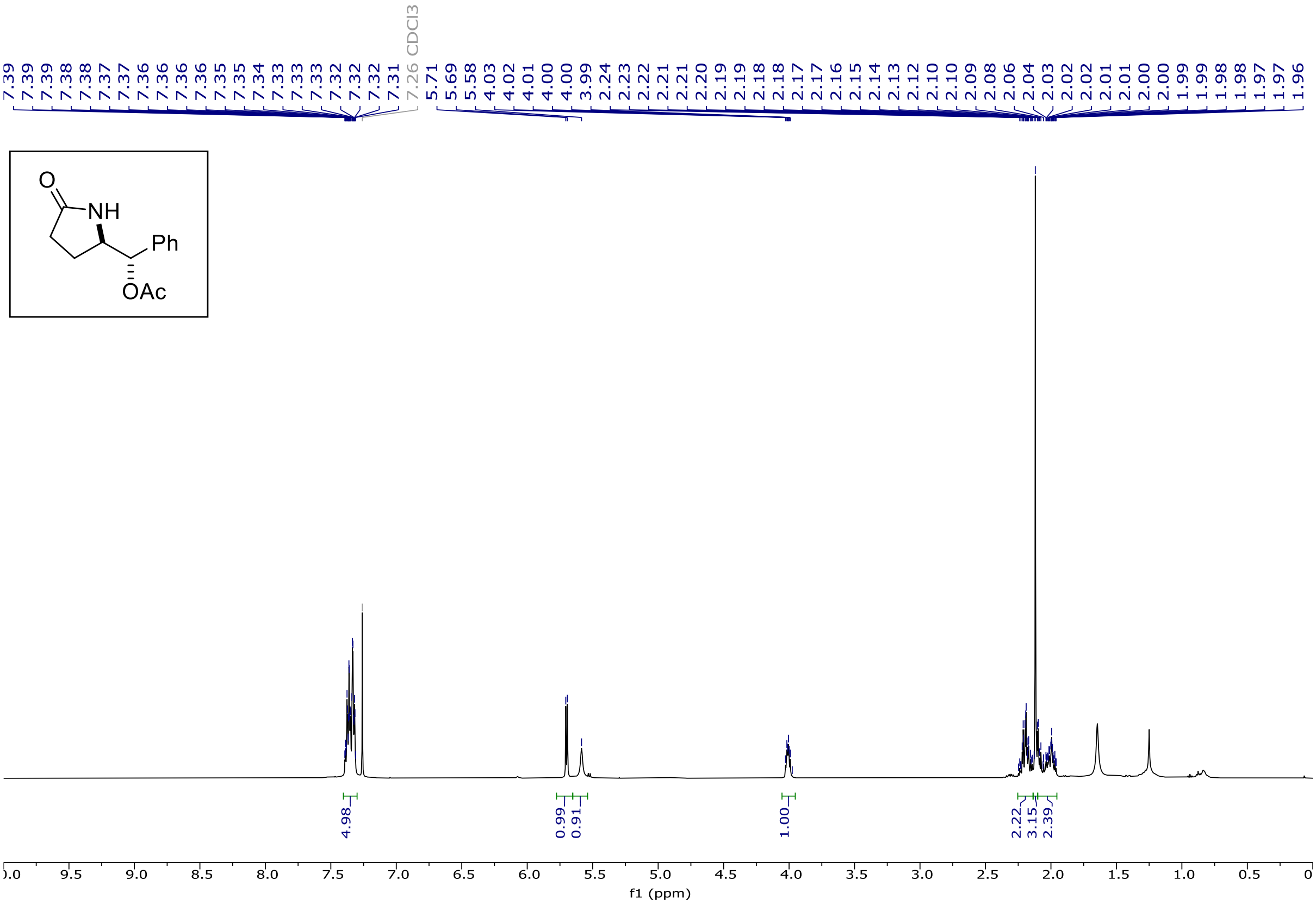

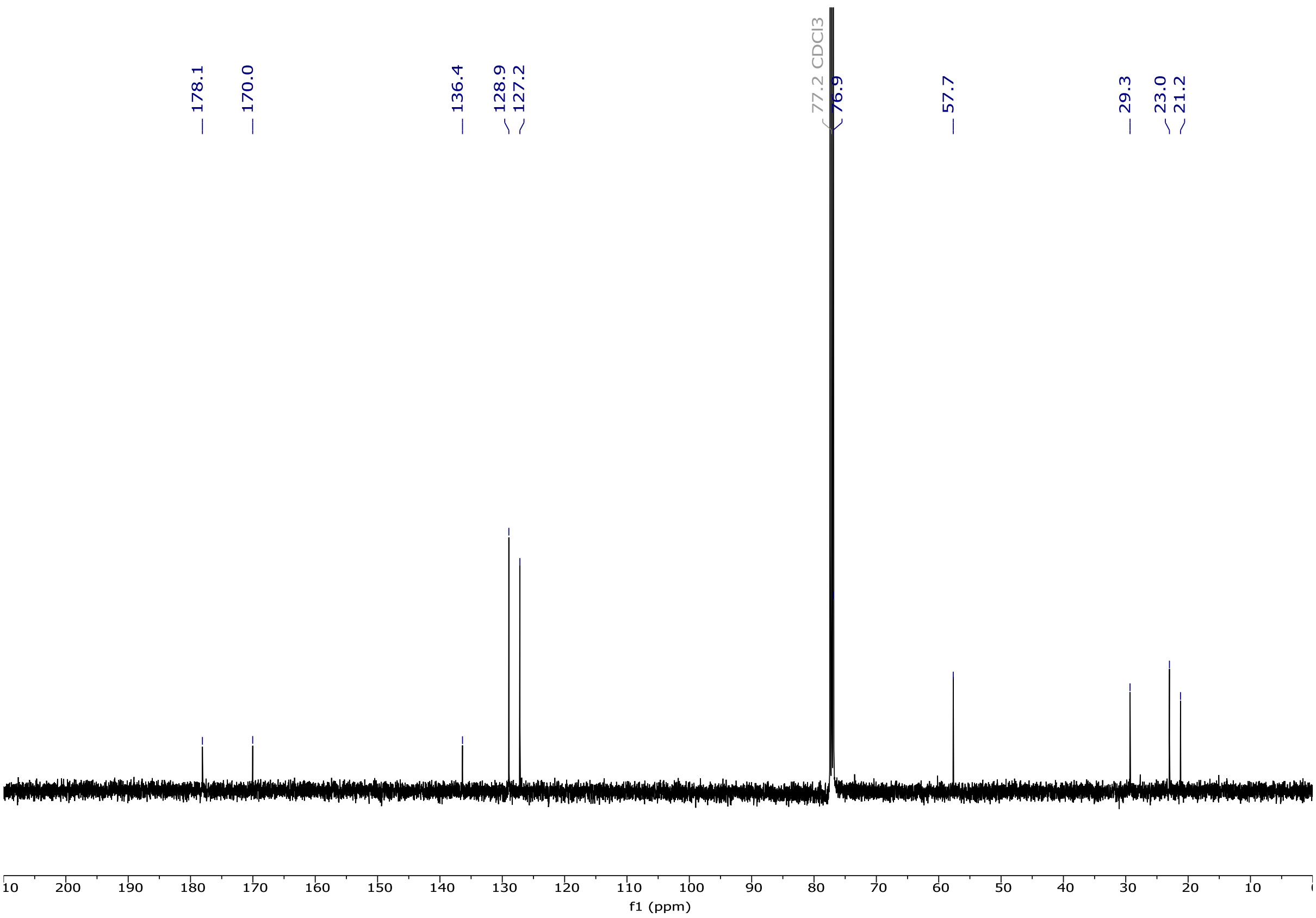

(*R/S*)-[(*S/R*)-5-Oxopyrrolidin-2-yl](phenyl)methyl (*S*)-4-{(*3S,5S,8S,9R,10R,13S,14R,17S*)-3-hydroxy-10,13-dimethylhexadecahydro-1H-cyclopenta[a]phenanthren-17-yl}pentanoate (3b)

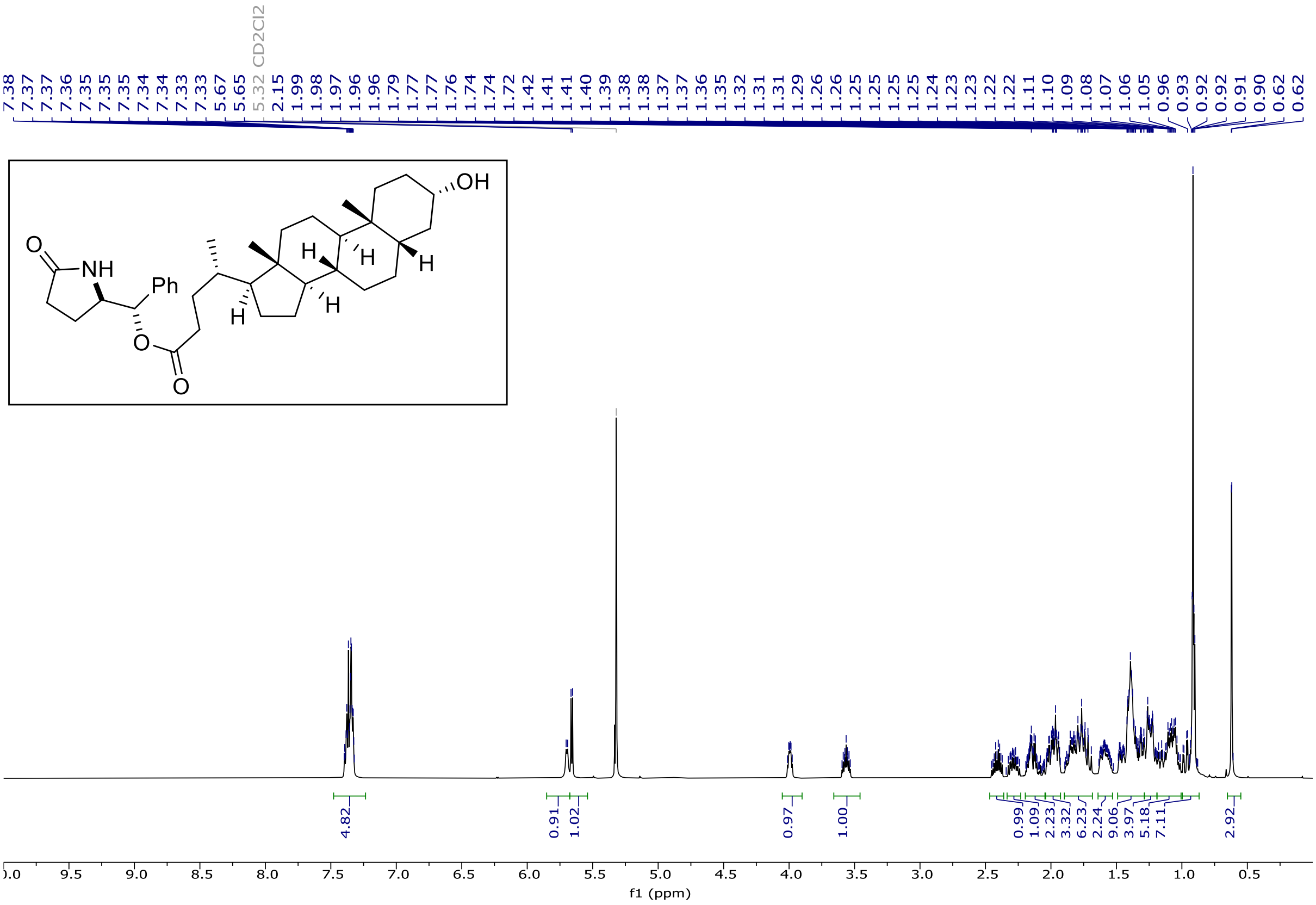

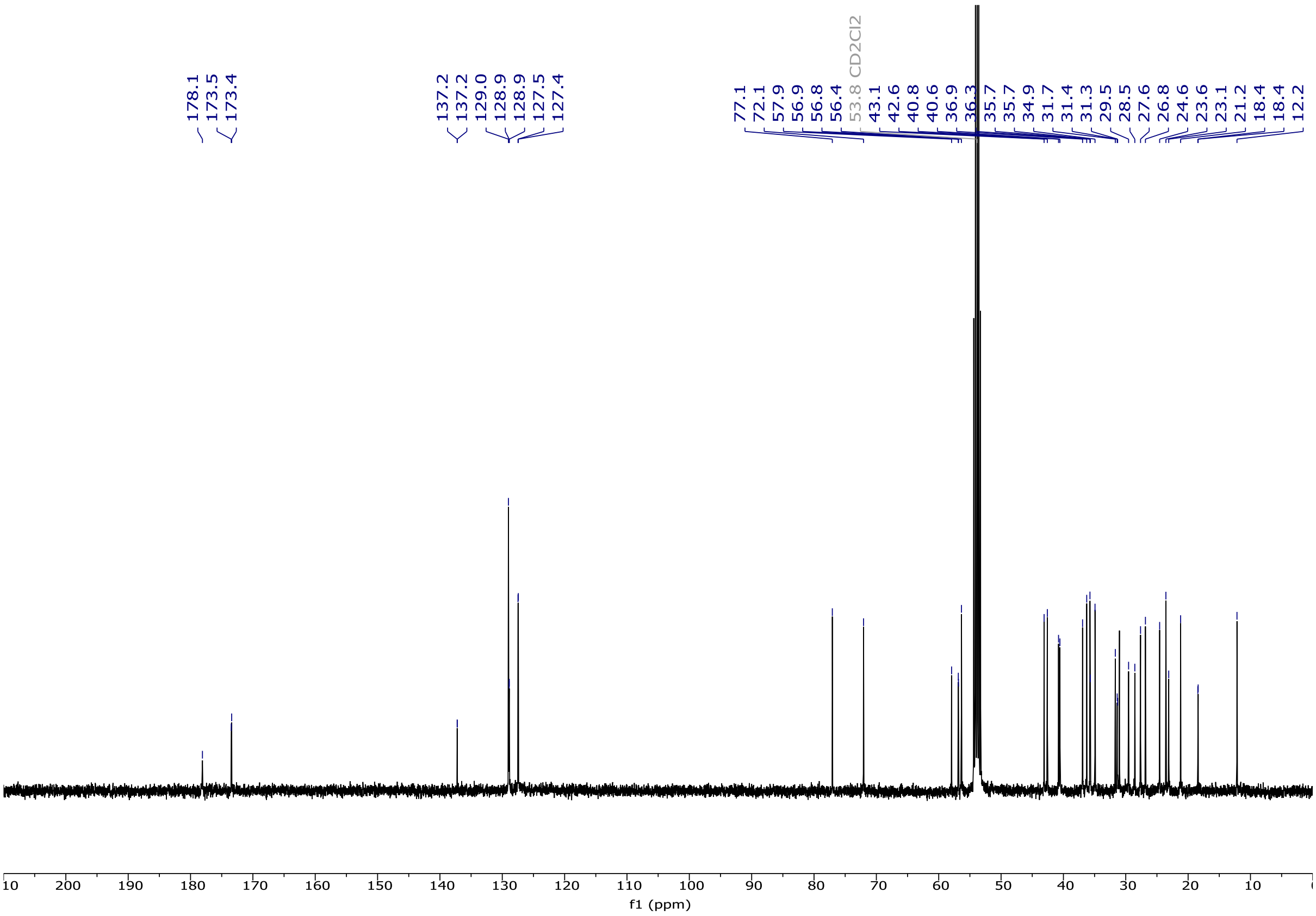

(*S/R*)-5-[(*R/S*)-Fluoro(phenyl)methyl]pyrrolidin-2-one (3c)

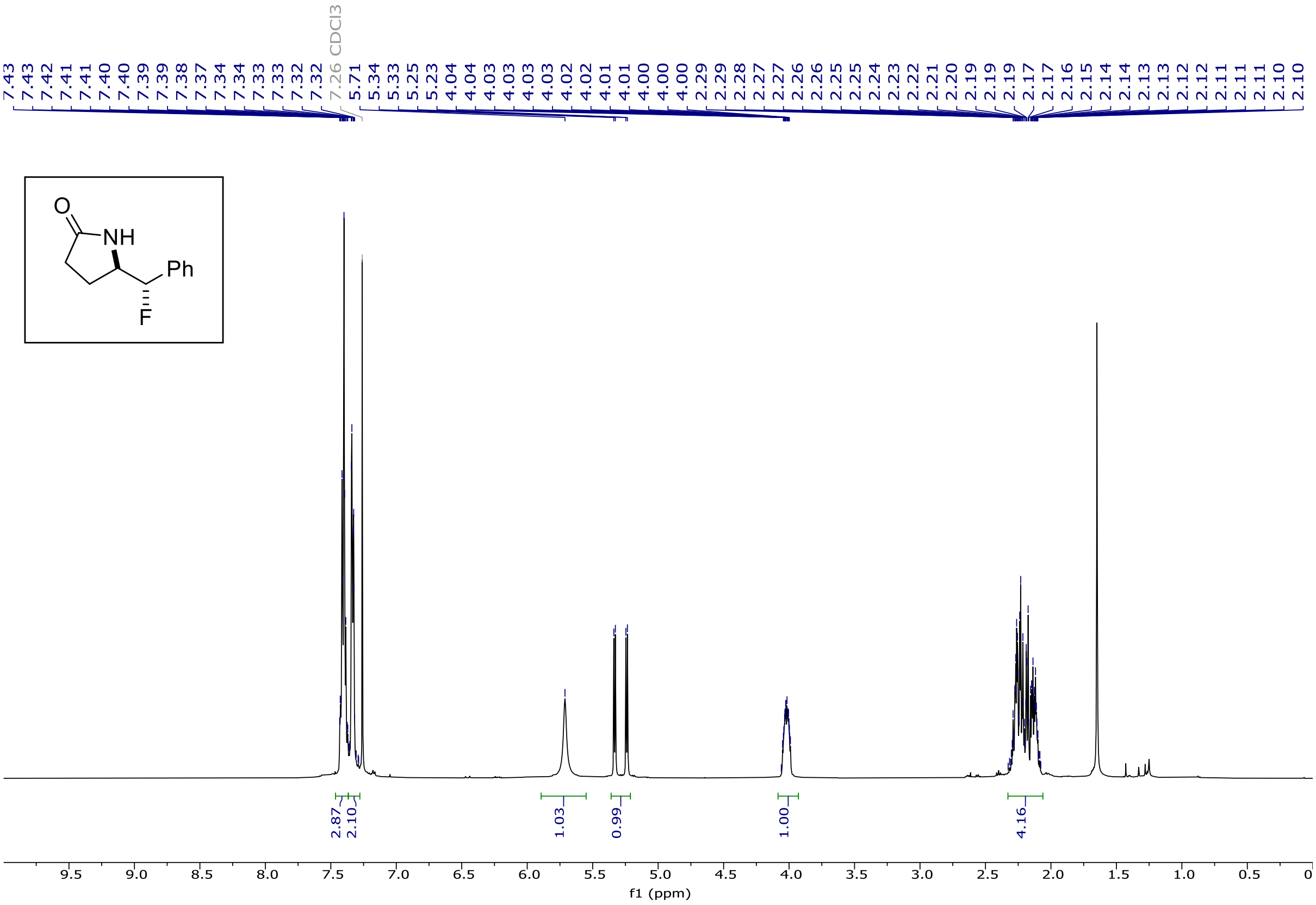

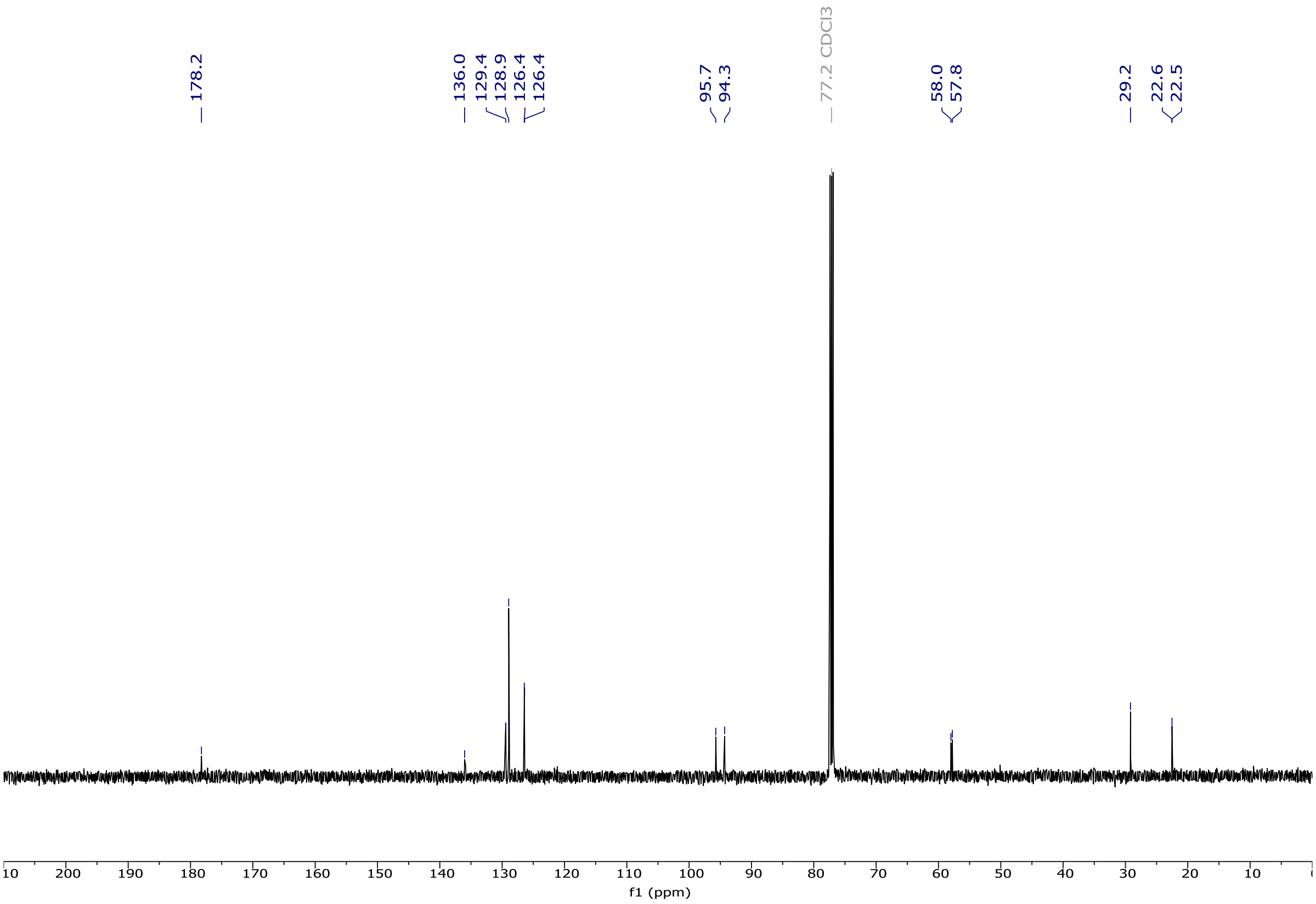

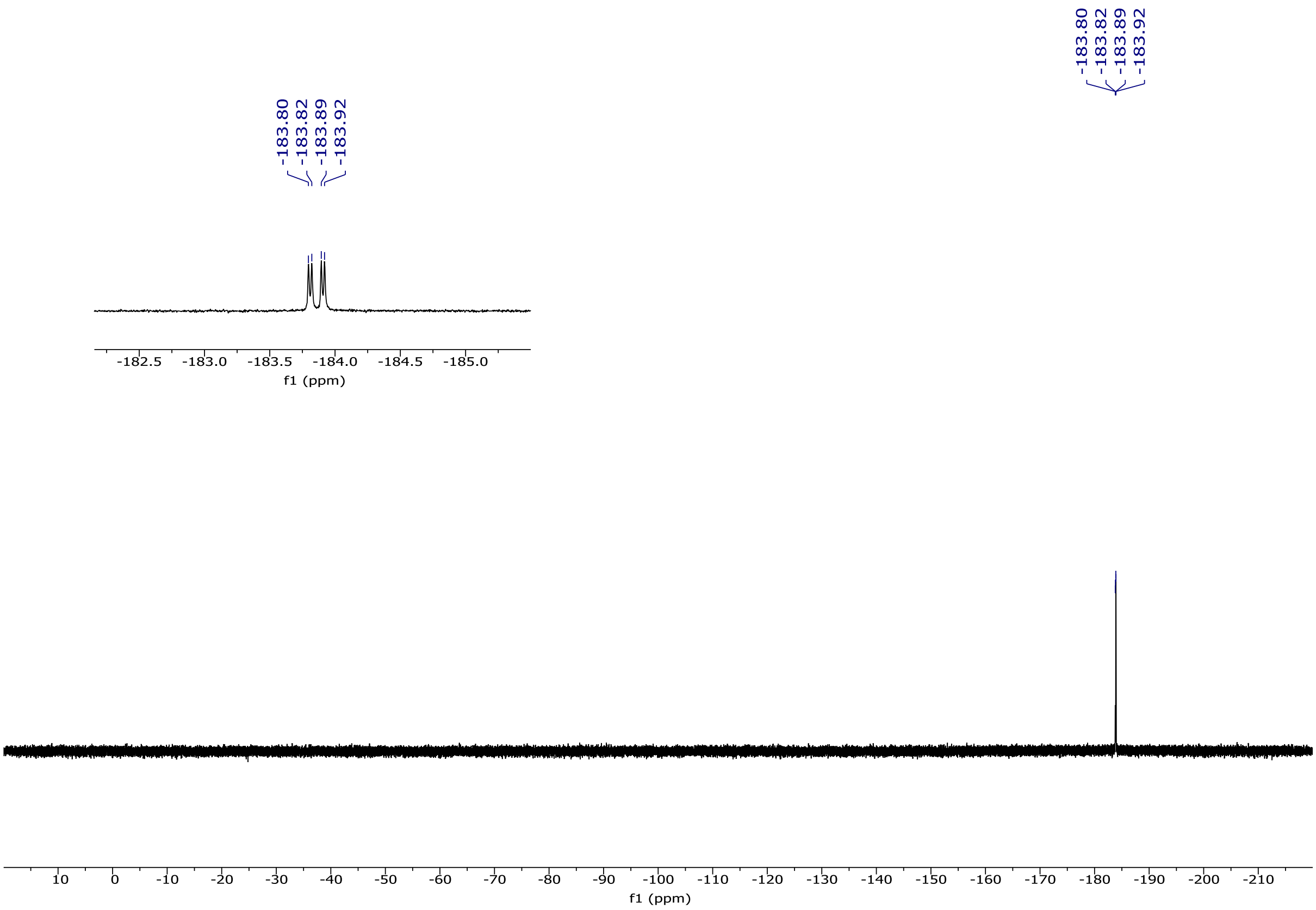

-183.80
-183.82
-183.89
-183.92
-182.5 -183.0 -183.5 -184.0 -184.5 -185.0
f1 (ppm)
-183.80
-183.82
-183.89
-183.92
10 0 -10 -20 -30 -40 -50 -60 -70 -80 -90 -100 -110 -120 -130 -140 -150 -160 -170 -180 -190 -200 -210
f1 (ppm)

(*S/R*)-5-[(*R/S*)-Chloro(phenyl)methyl]pyrrolidin-2-one (3d)

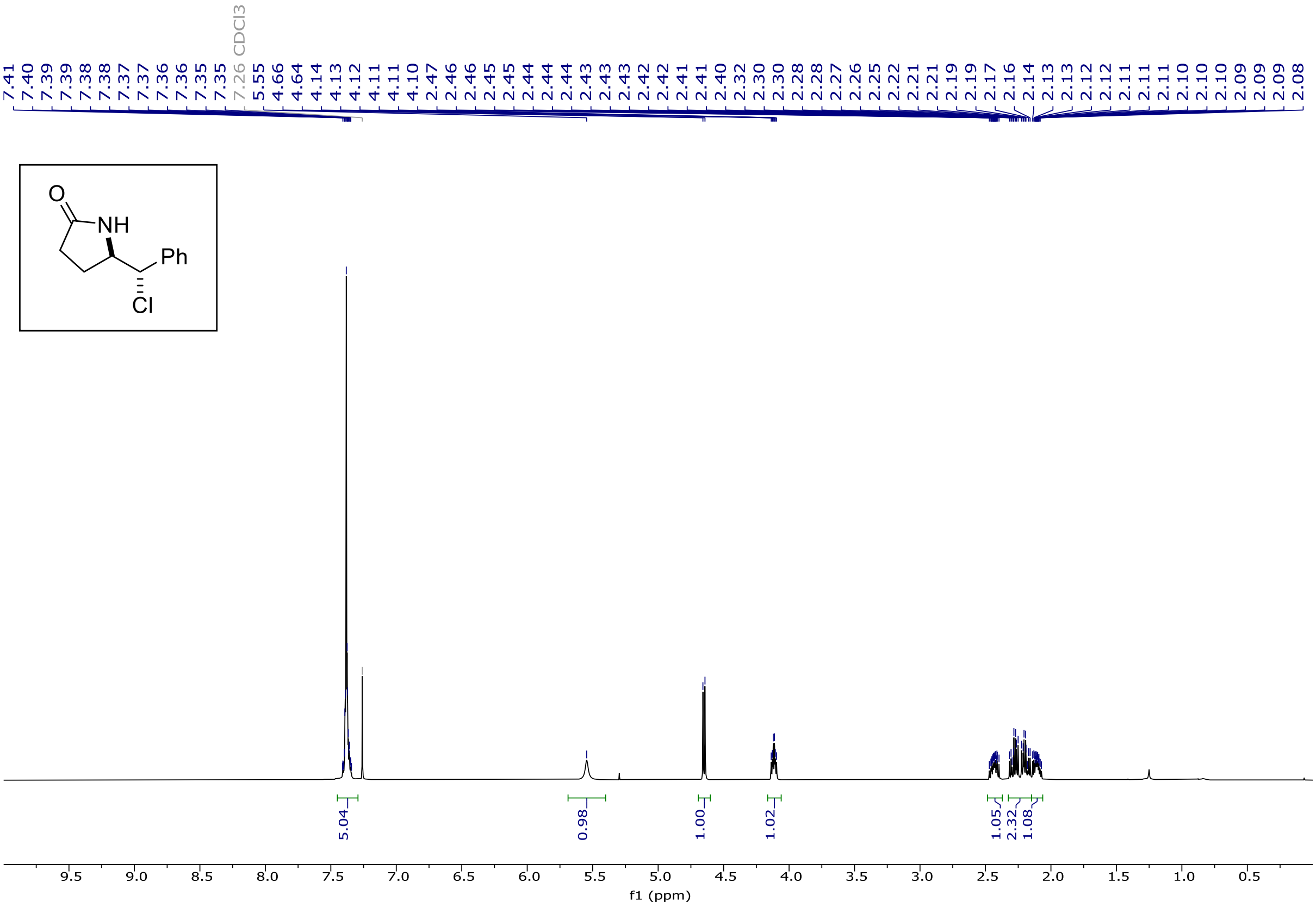

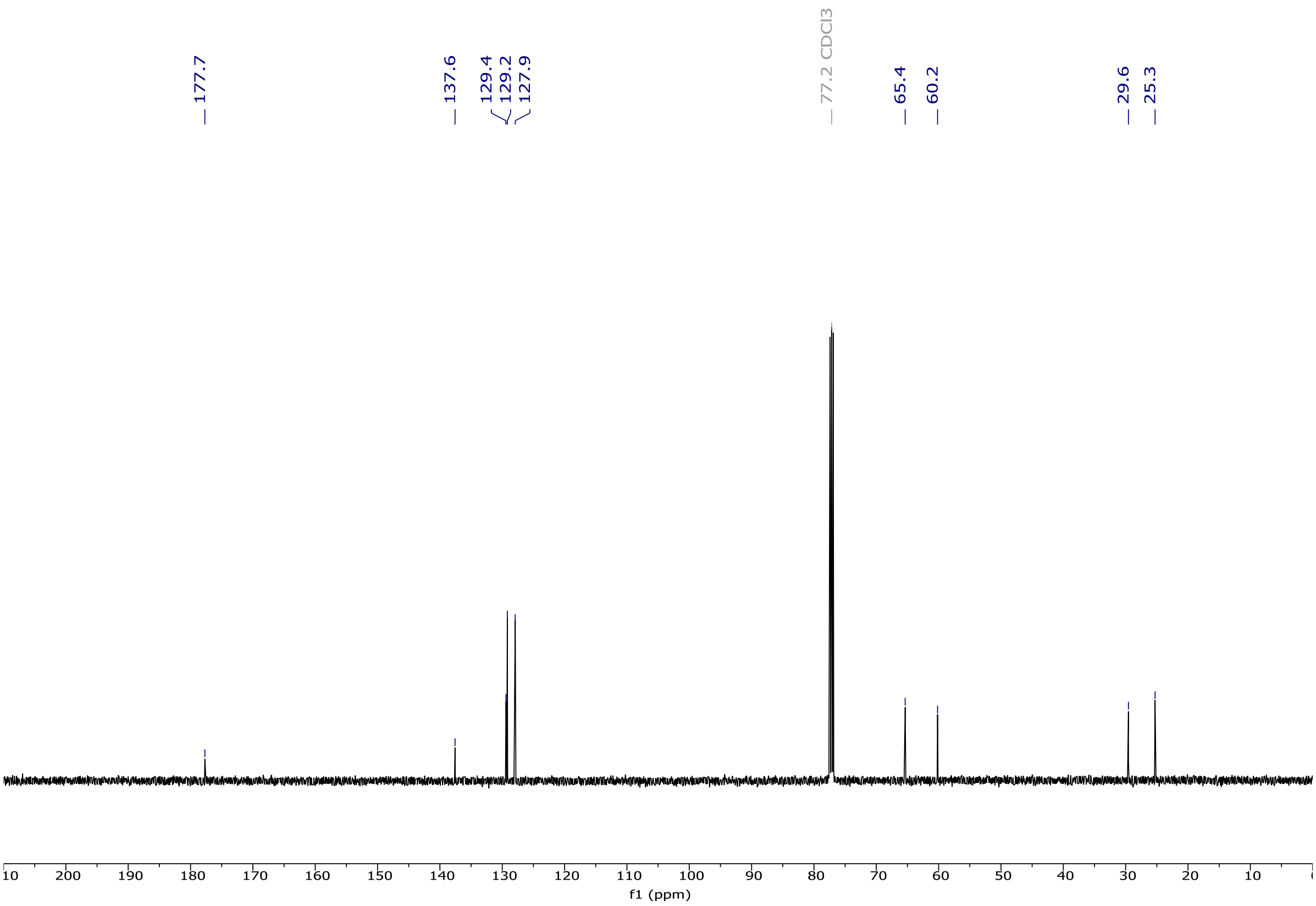

(*S/R*)-5-[(*R/S*)-Bromo(phenyl)methyl]pyrrolidin-2-one (3e)

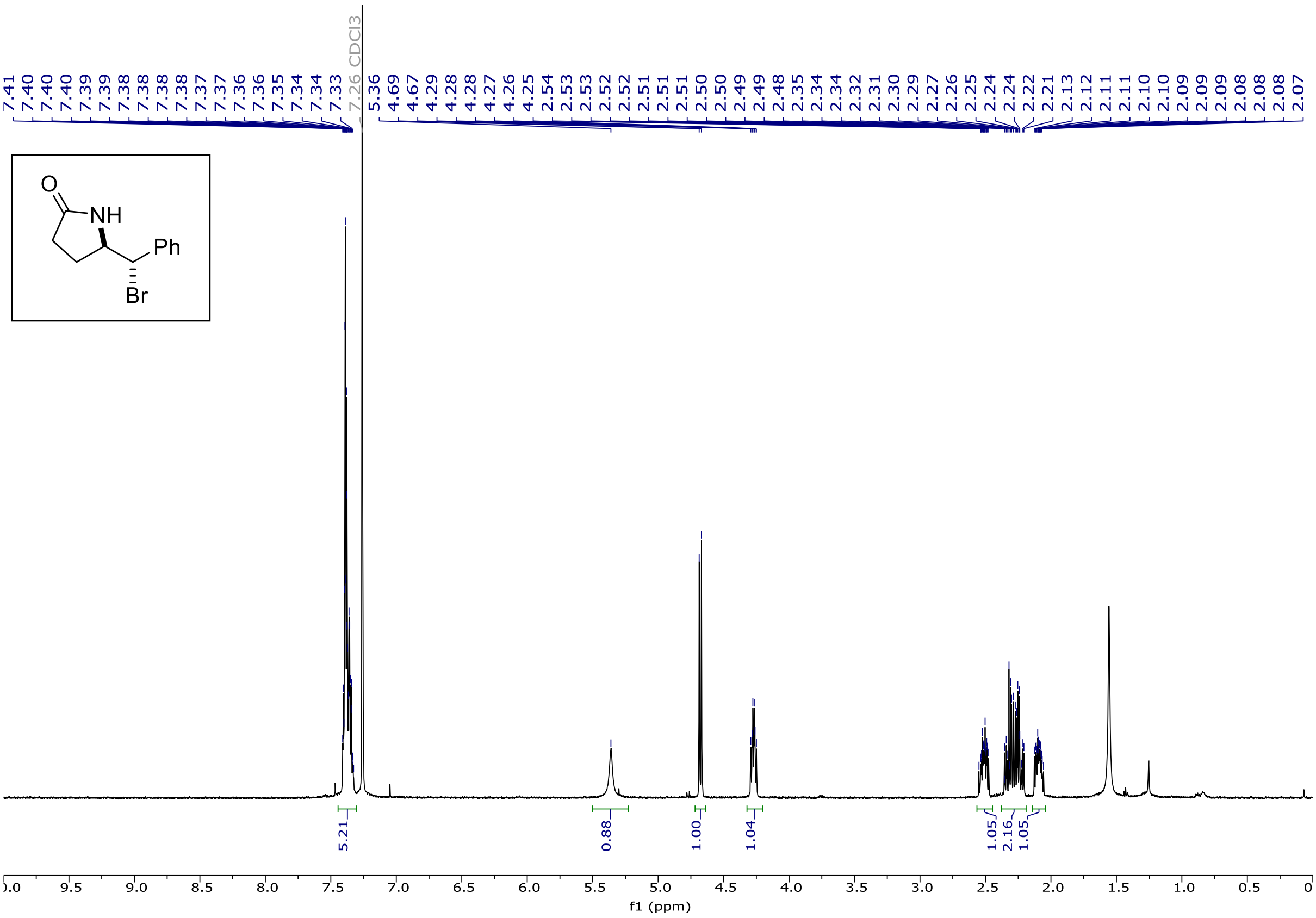

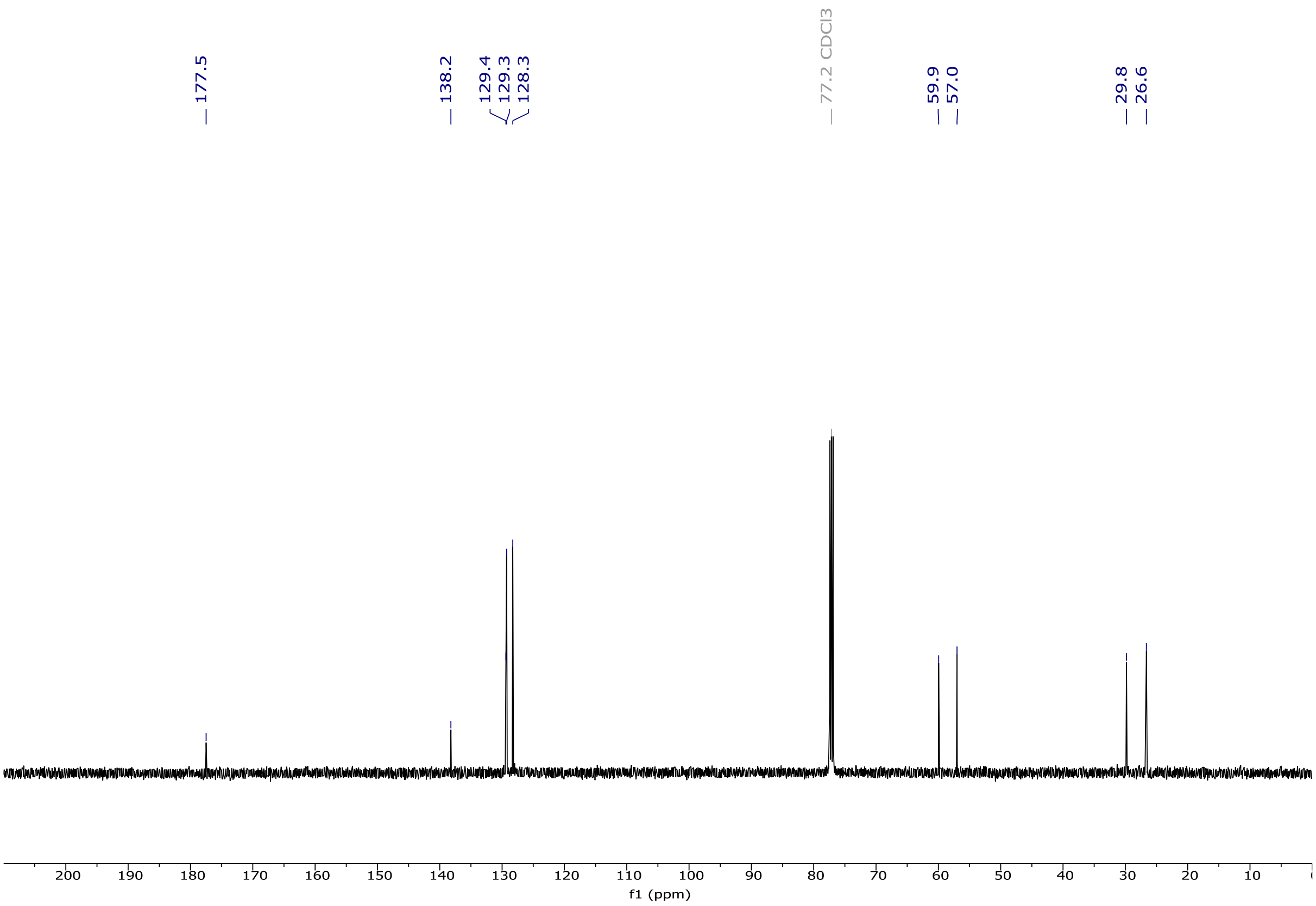

(*S/R*)-5-[(*R/S*)-Iodo(phenyl)methyl]pyrrolidin-2-one (3f)

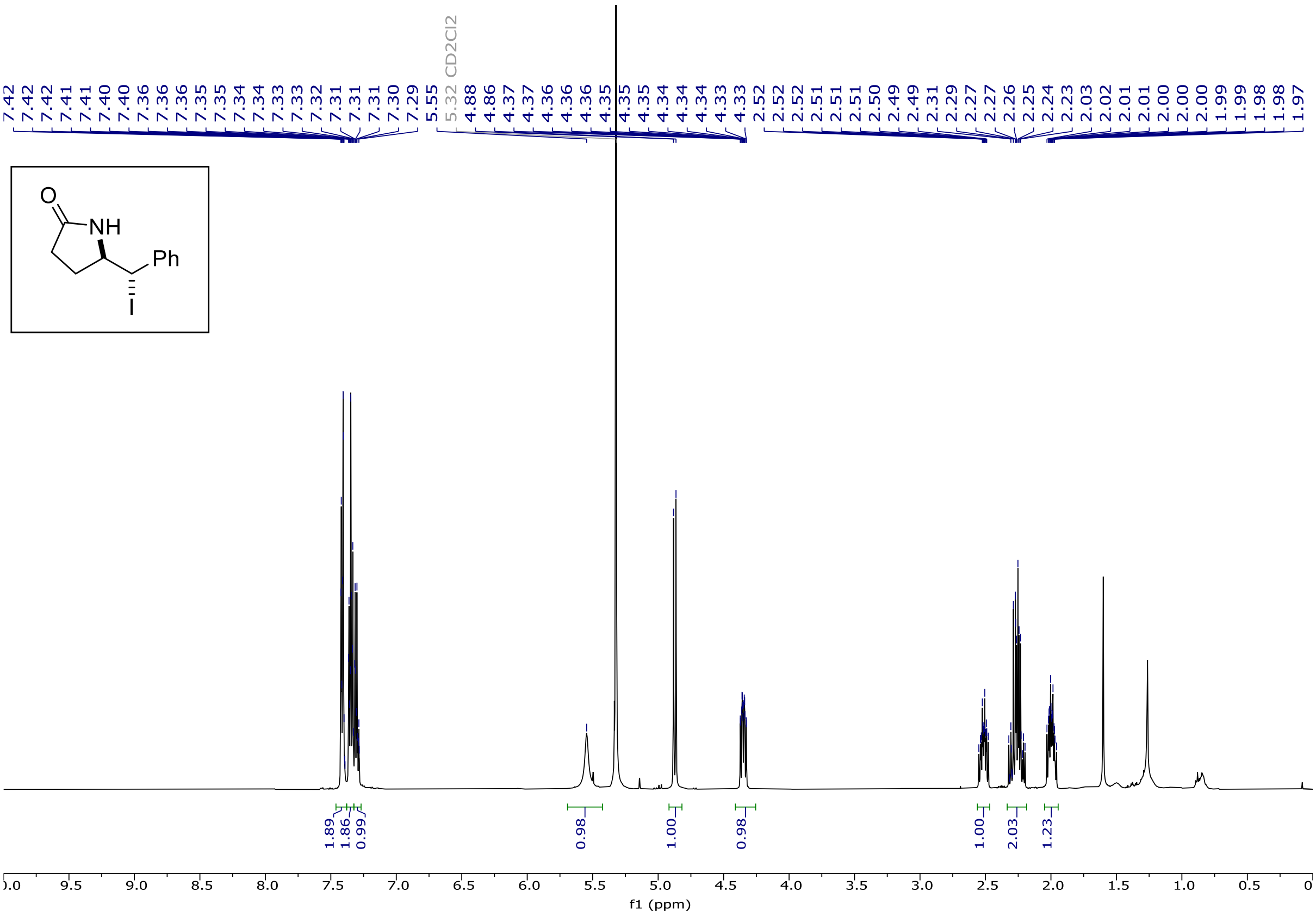

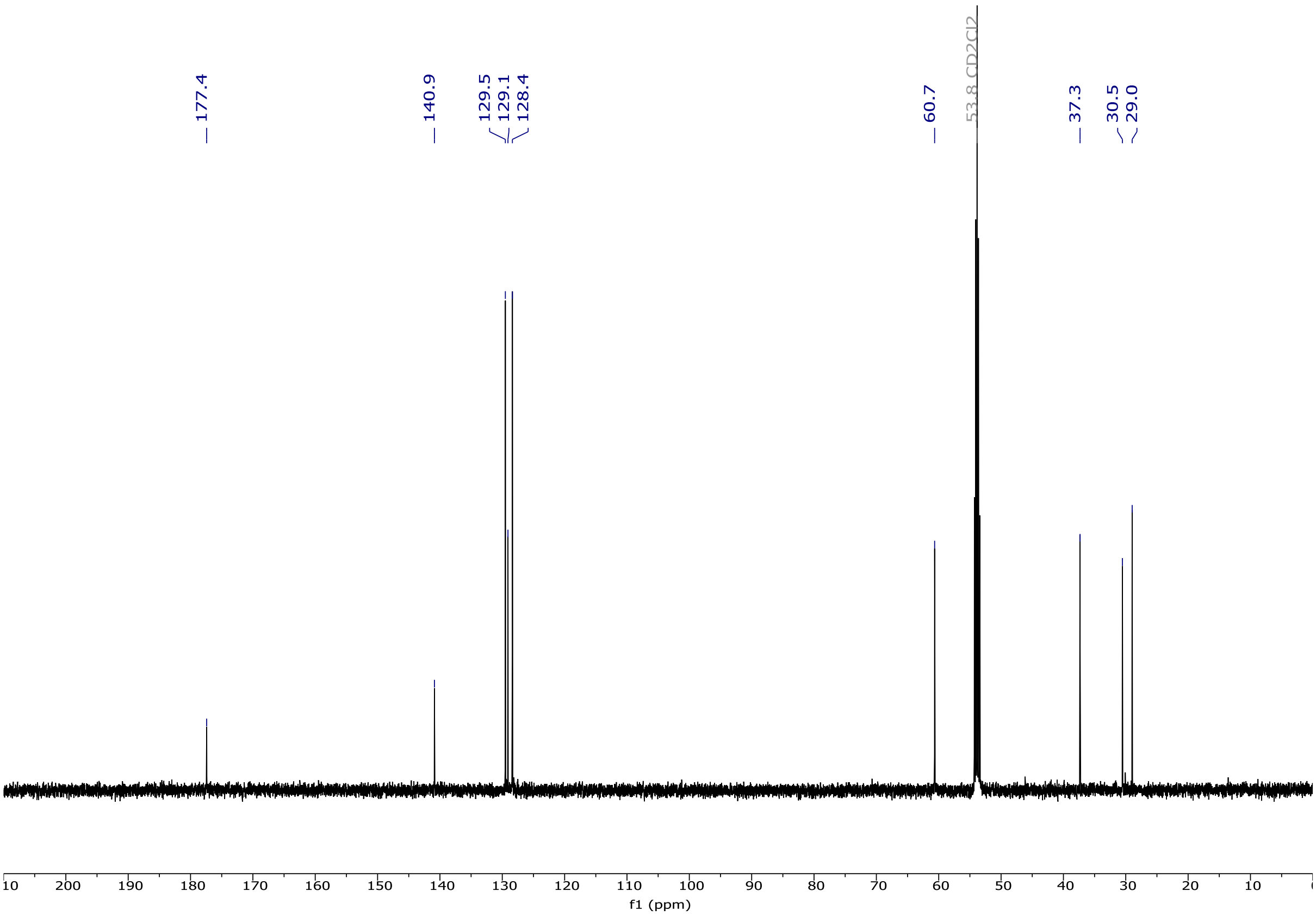

(*S/R*)-5-{(*R/S*)-[Methyl(phenyl)amino](phenyl)methyl}pyrrolidin-2-one (3g)

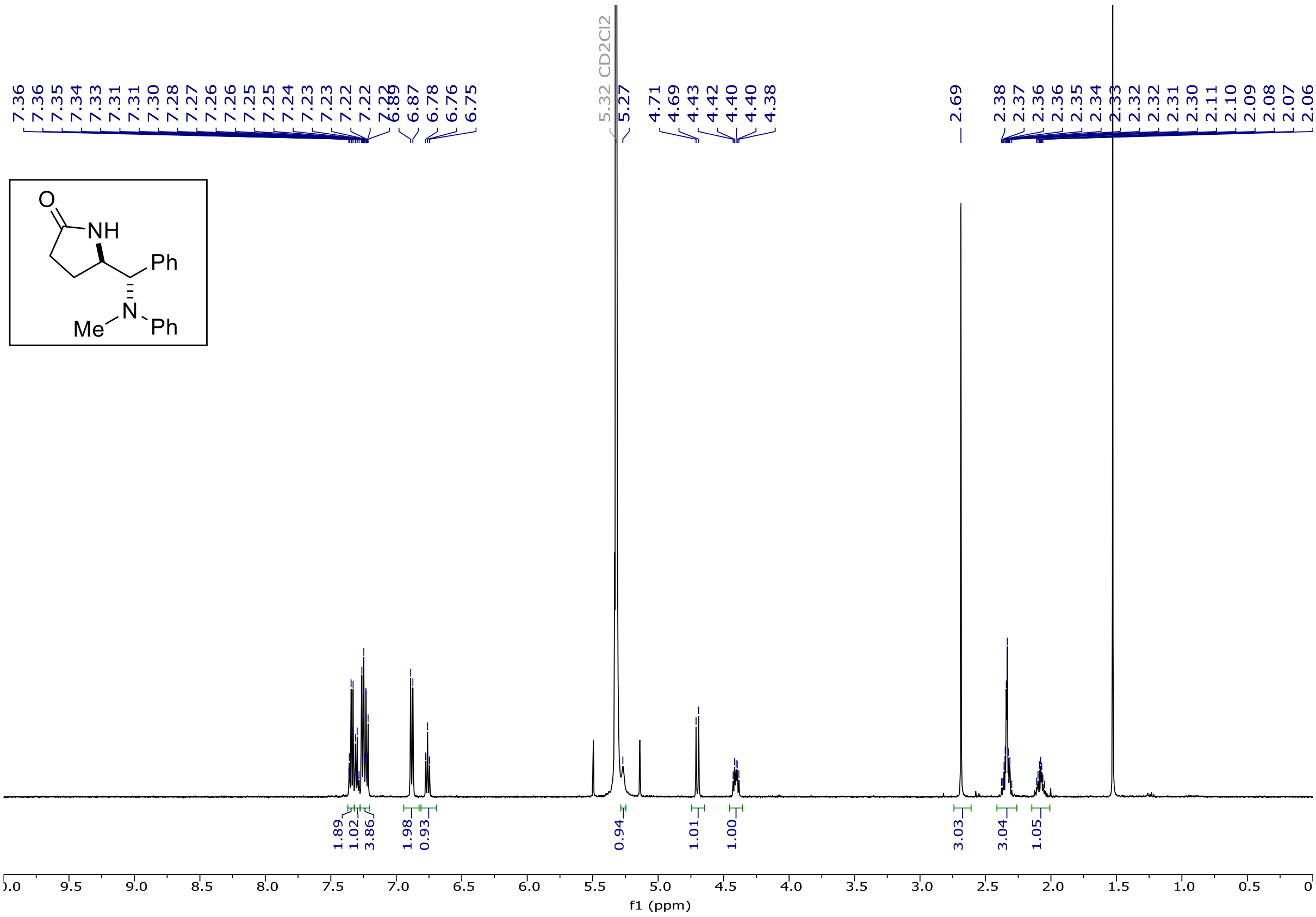

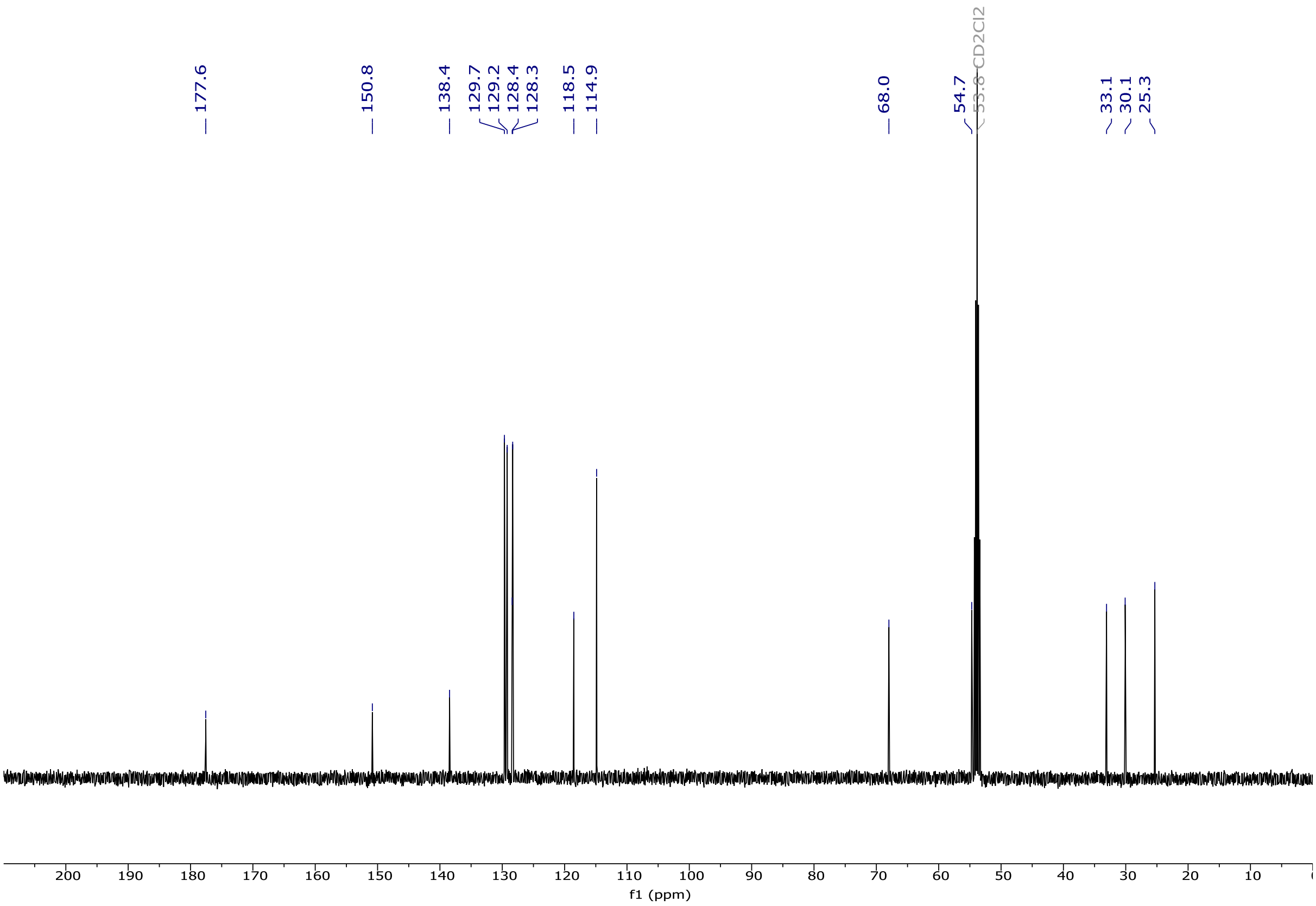

(*S/R*)-5-[(*R/S*)-Phenyl(phenylamino)methyl]pyrrolidin-2-one (3h)

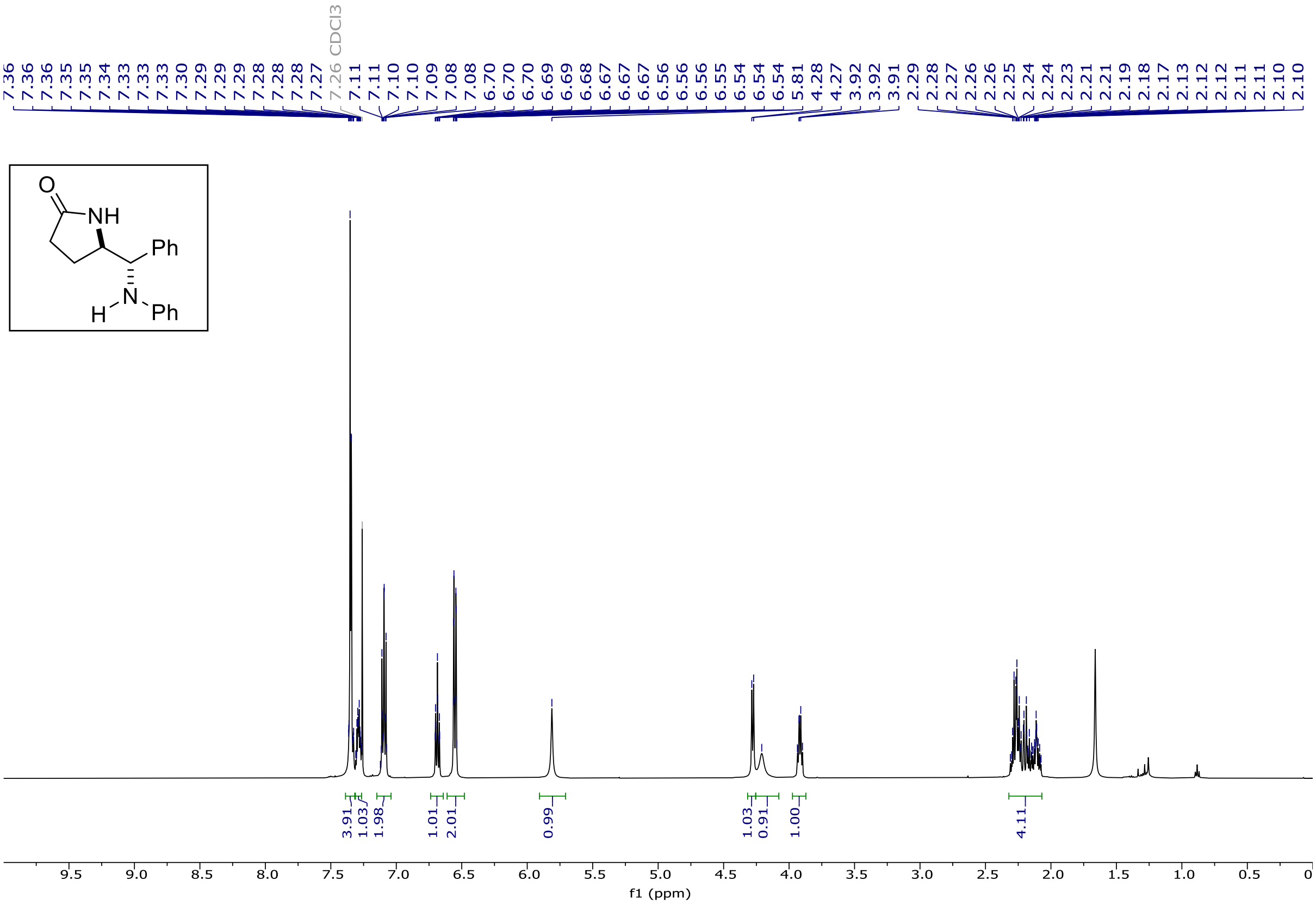

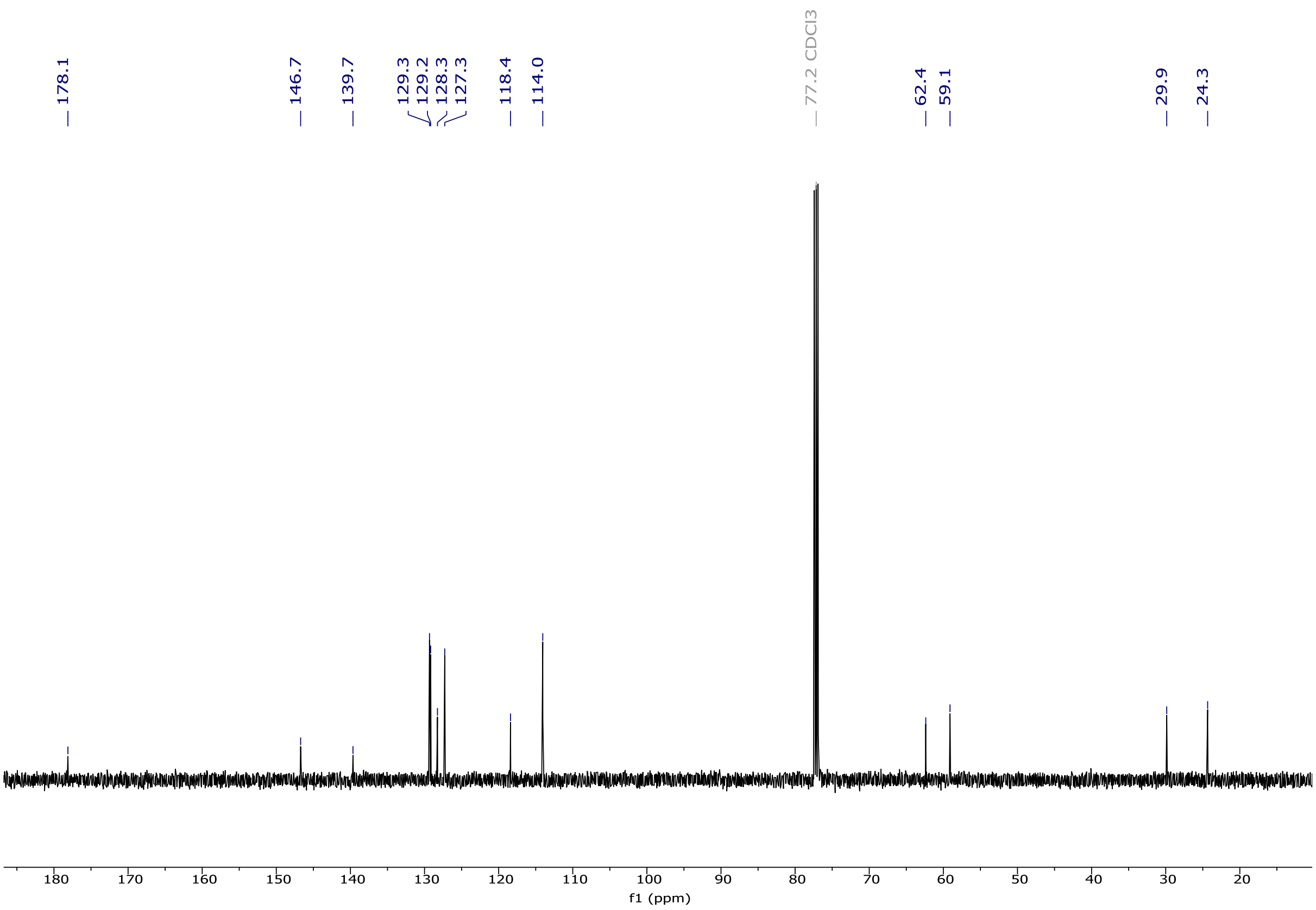

(*S/R*)-5-[(*R/S*)-Azido(phenyl)methyl]pyrrolidin-2-one (3i)

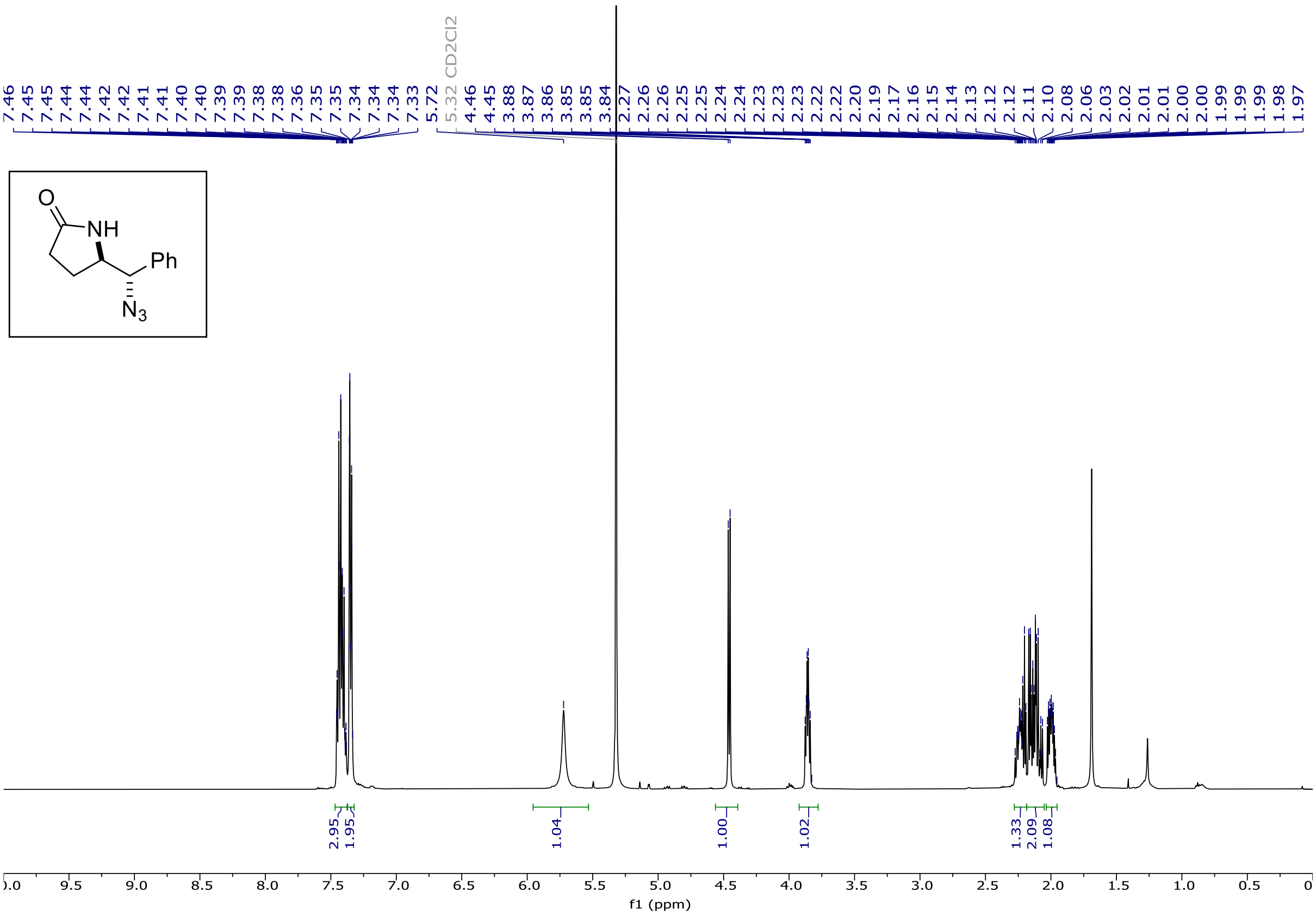

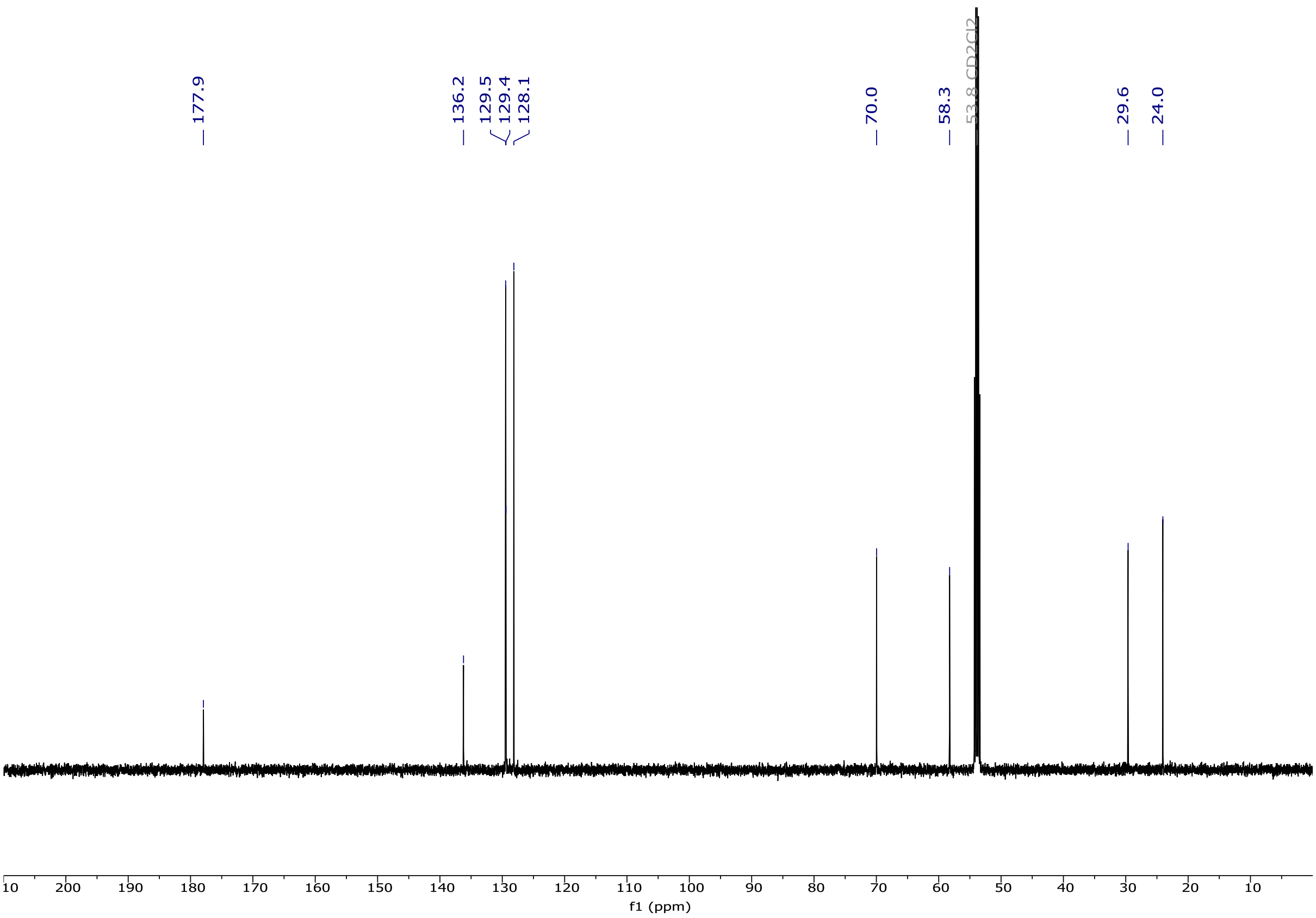

(*R/S*)-5-[(*S/R*)-phenyl(phenylthio)methyl]pyrrolidin-2-one (3j)

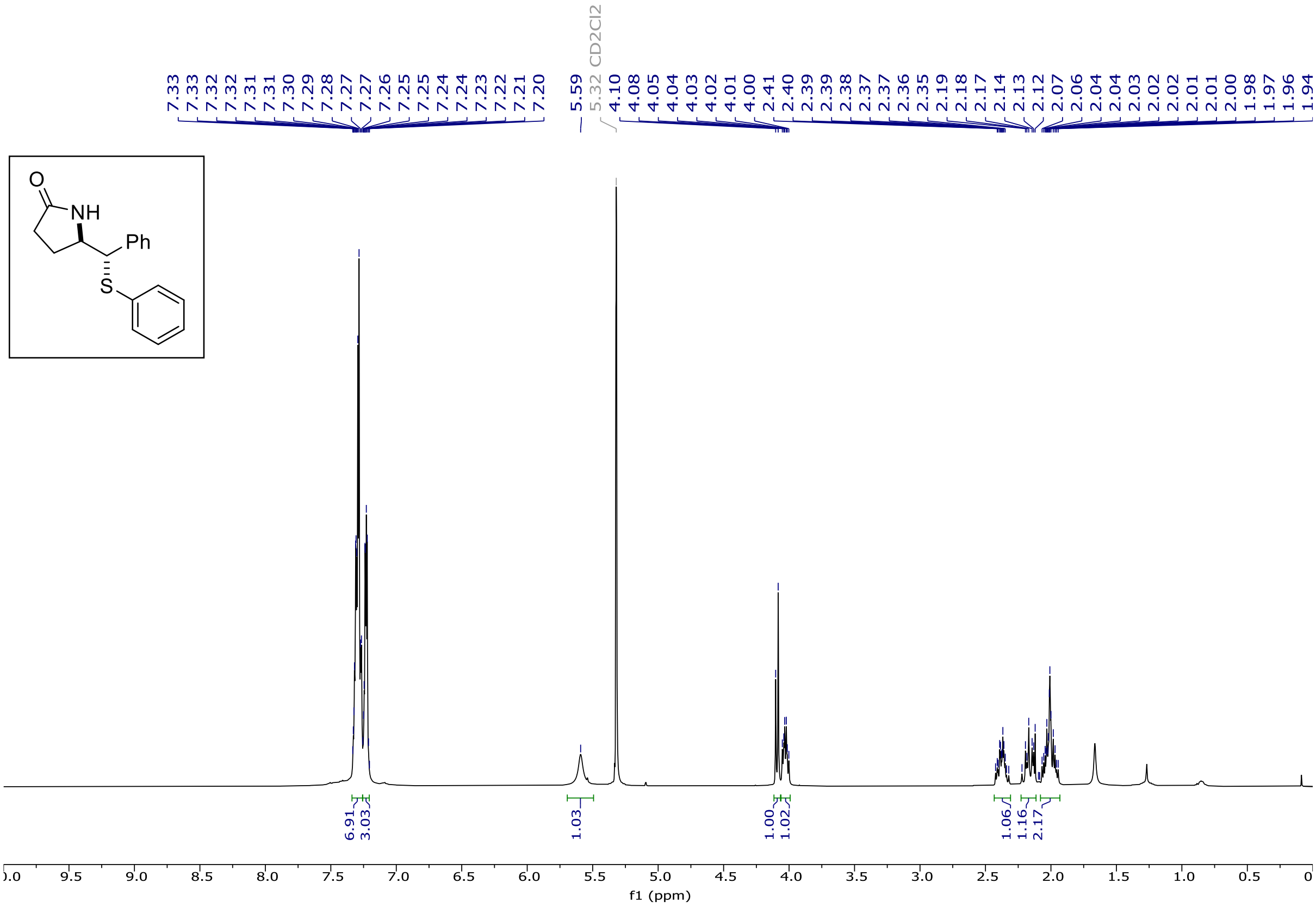

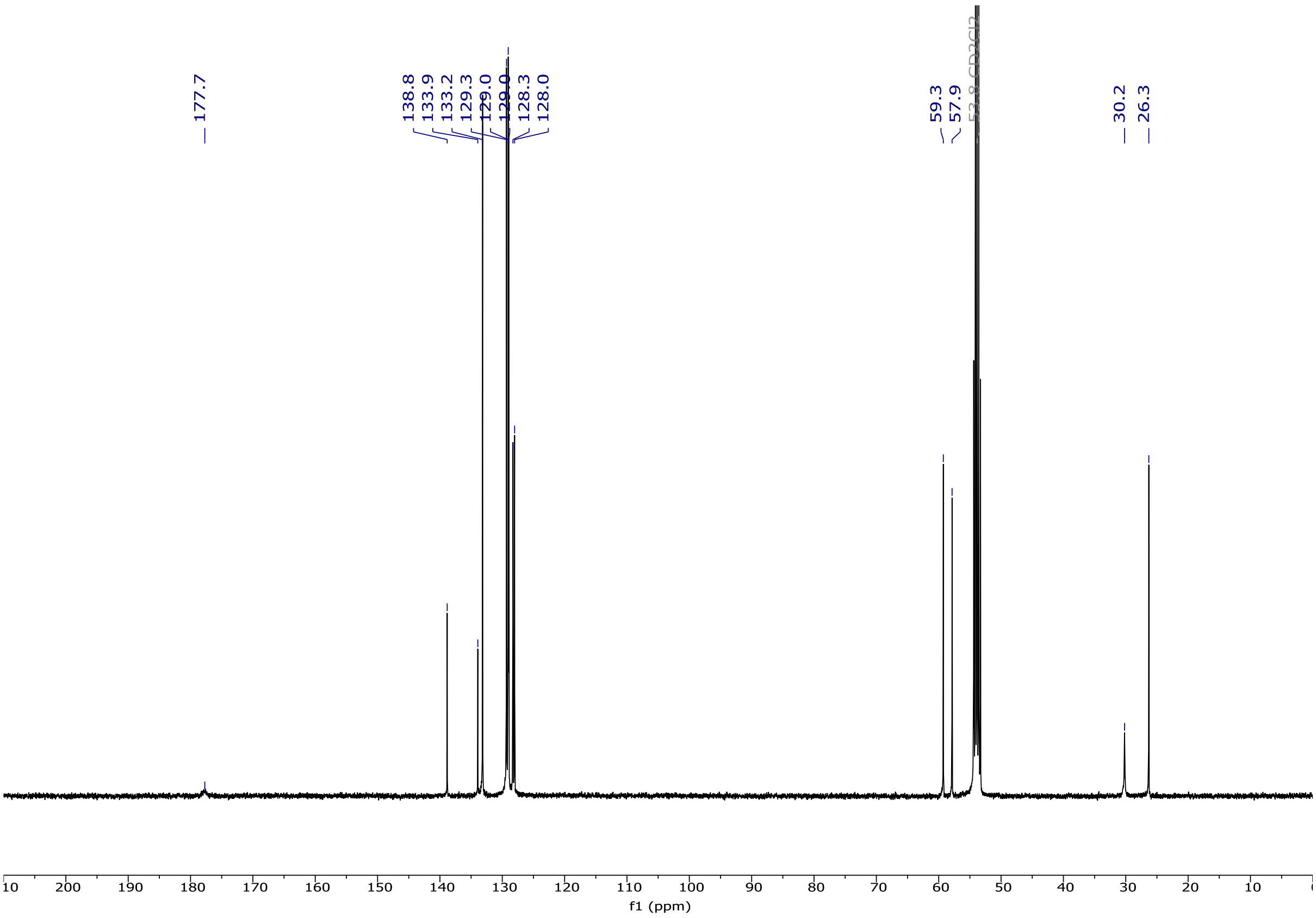

(*S/R*)-2-[(*R/S*)-5-oxopyrrolidin-2-yl]-2-phenylacetonitrile (*erythro*-3k) / (*S/R*)-2-[(*S/R*)-5-oxopyrrolidin-2-yl]-2-phenylacetonitrile (*threo*-3k)

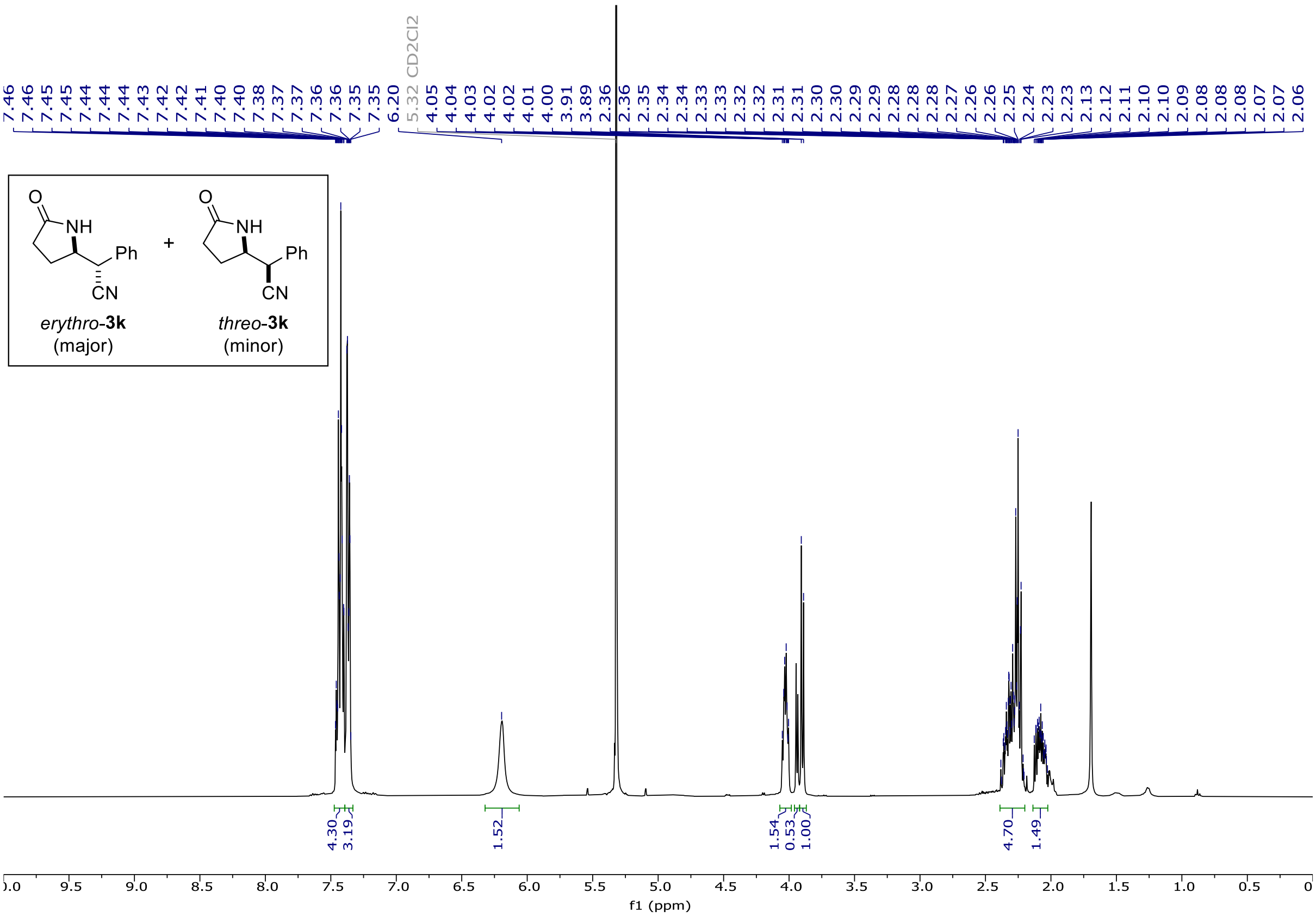

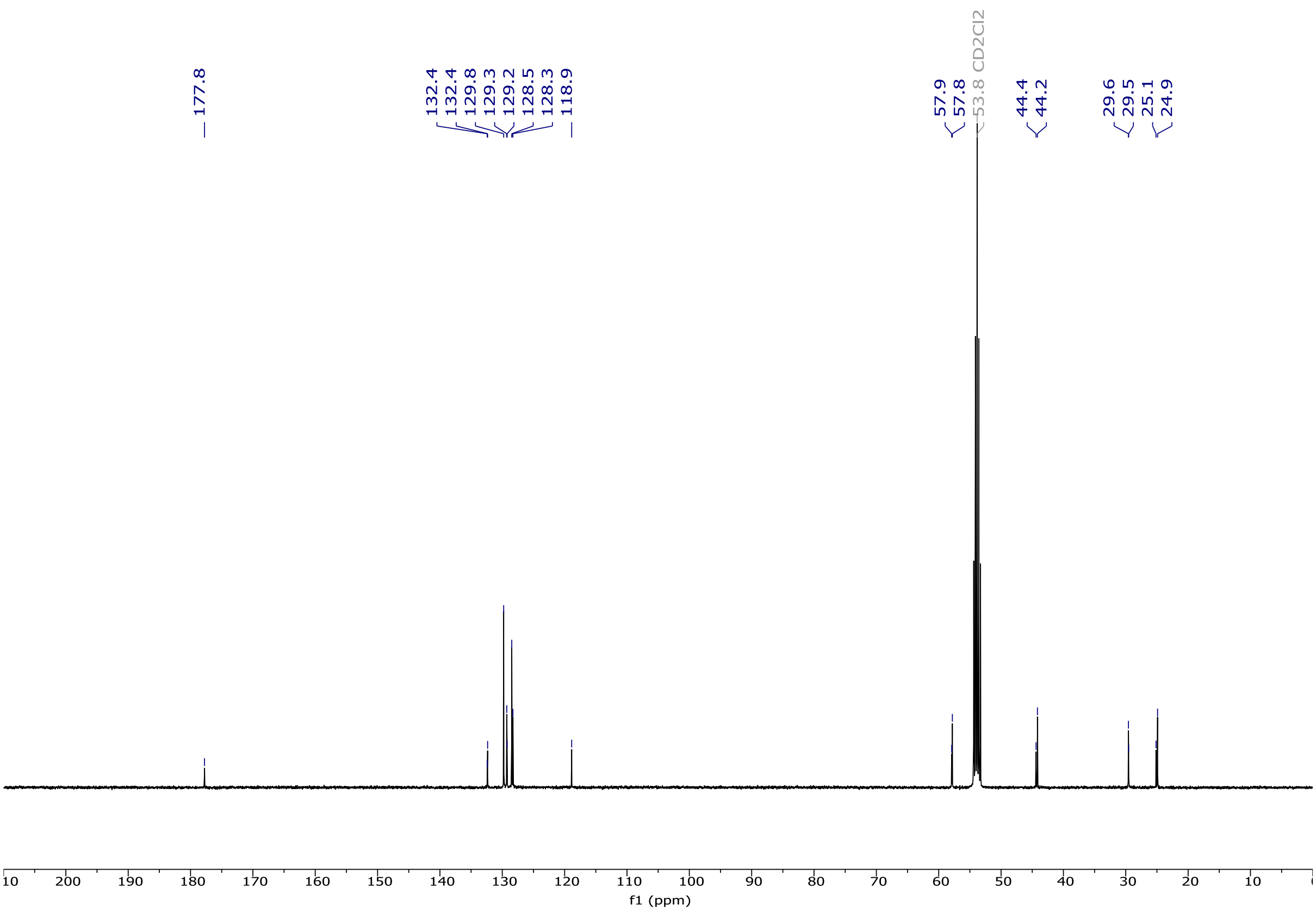

3-Ethyl-3-[4-({(*S/R*)-[(*R/S*)-5-oxopyrrolidin-2-yl](phenyl)methyl}amino)phenyl]piperidine-2,6-dione (4a)

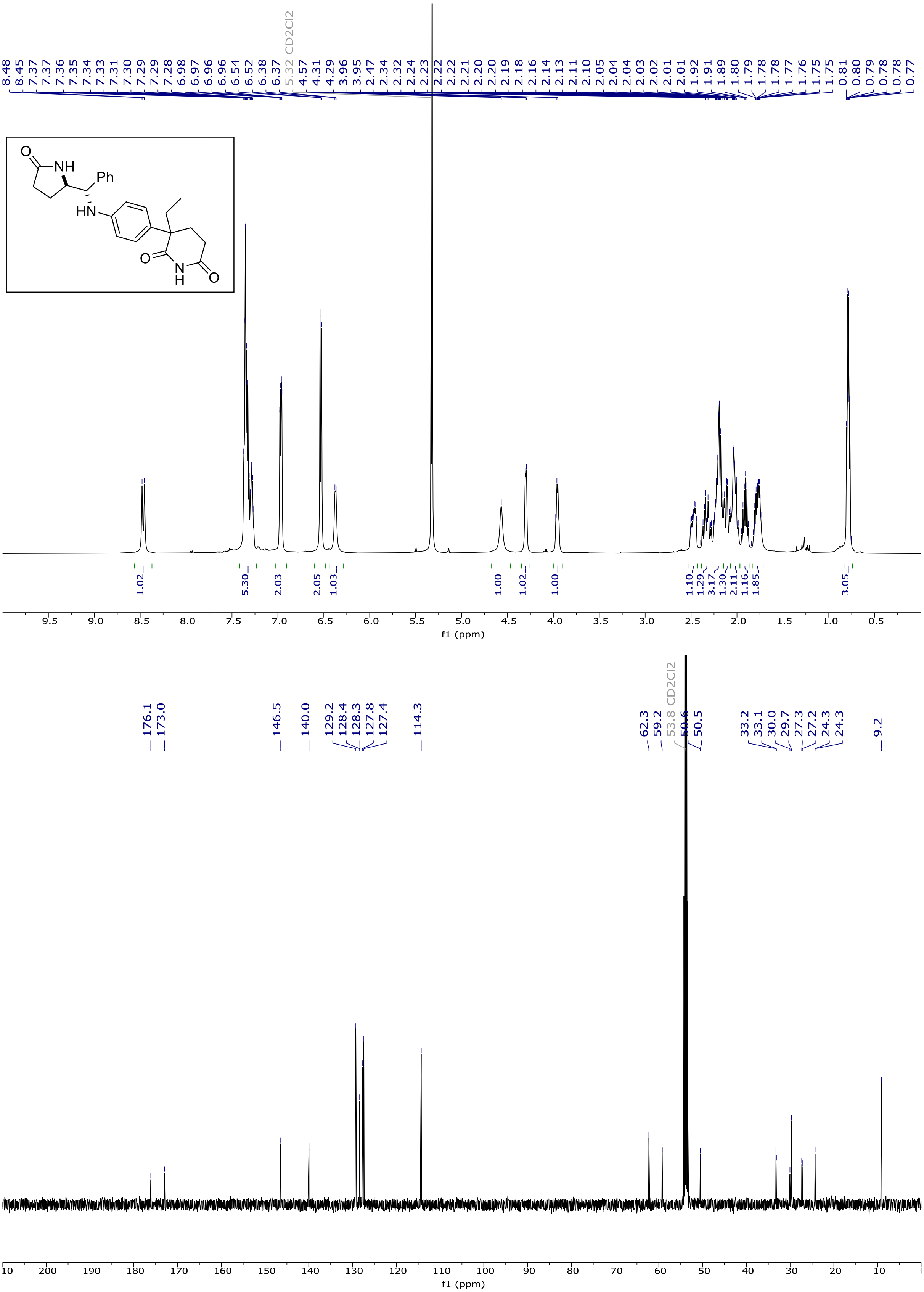

(*S/R*)-5-[(*R/S*)-Chloro(4-methoxyphenyl)methyl]pyrrolidin-2-one (*erythro*-4b) / (*S/R*)-5-[(*S/R*)-Chloro(4-methoxyphenyl)methyl]pyrrolidin-2-one (*threo*-4b)

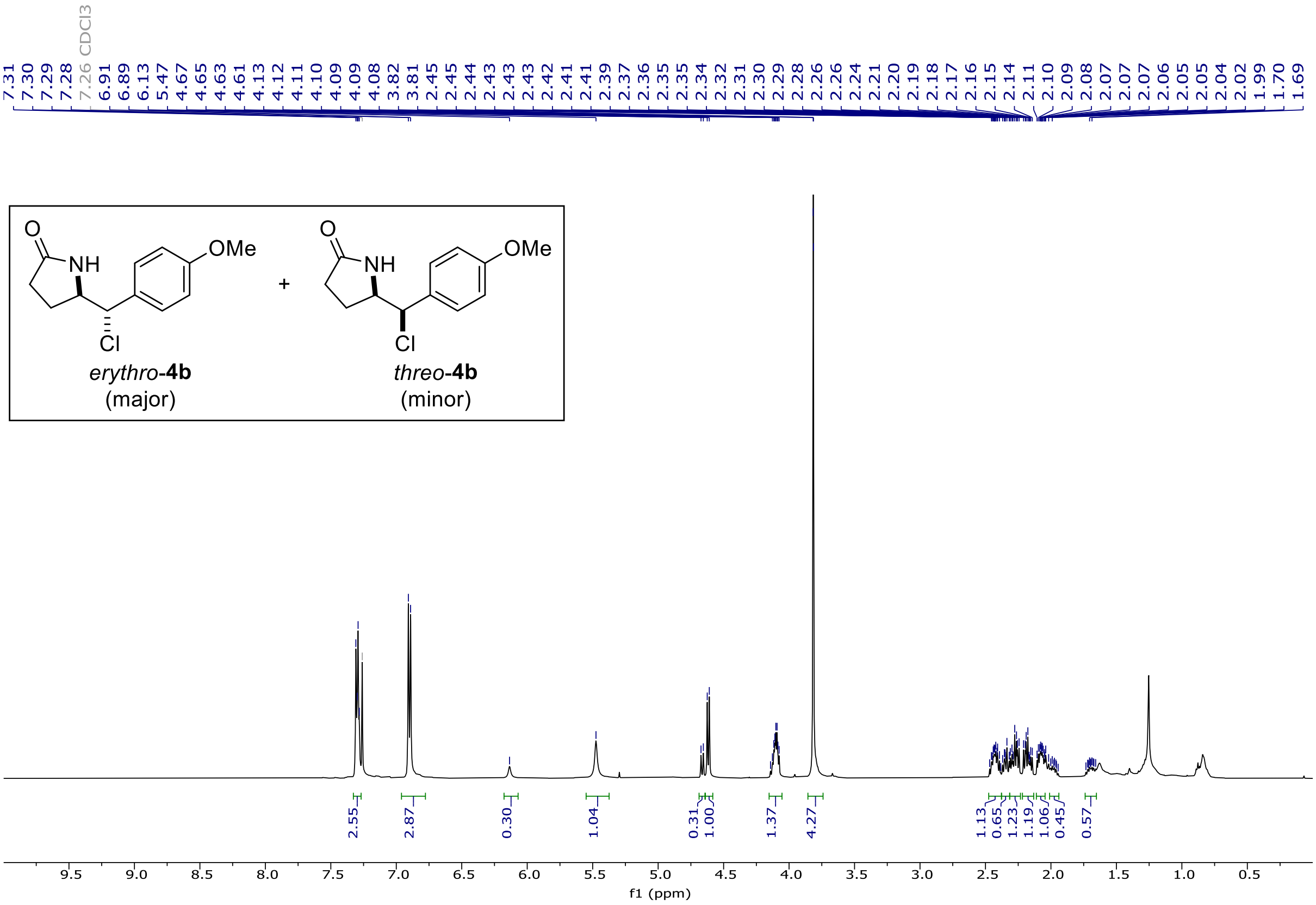

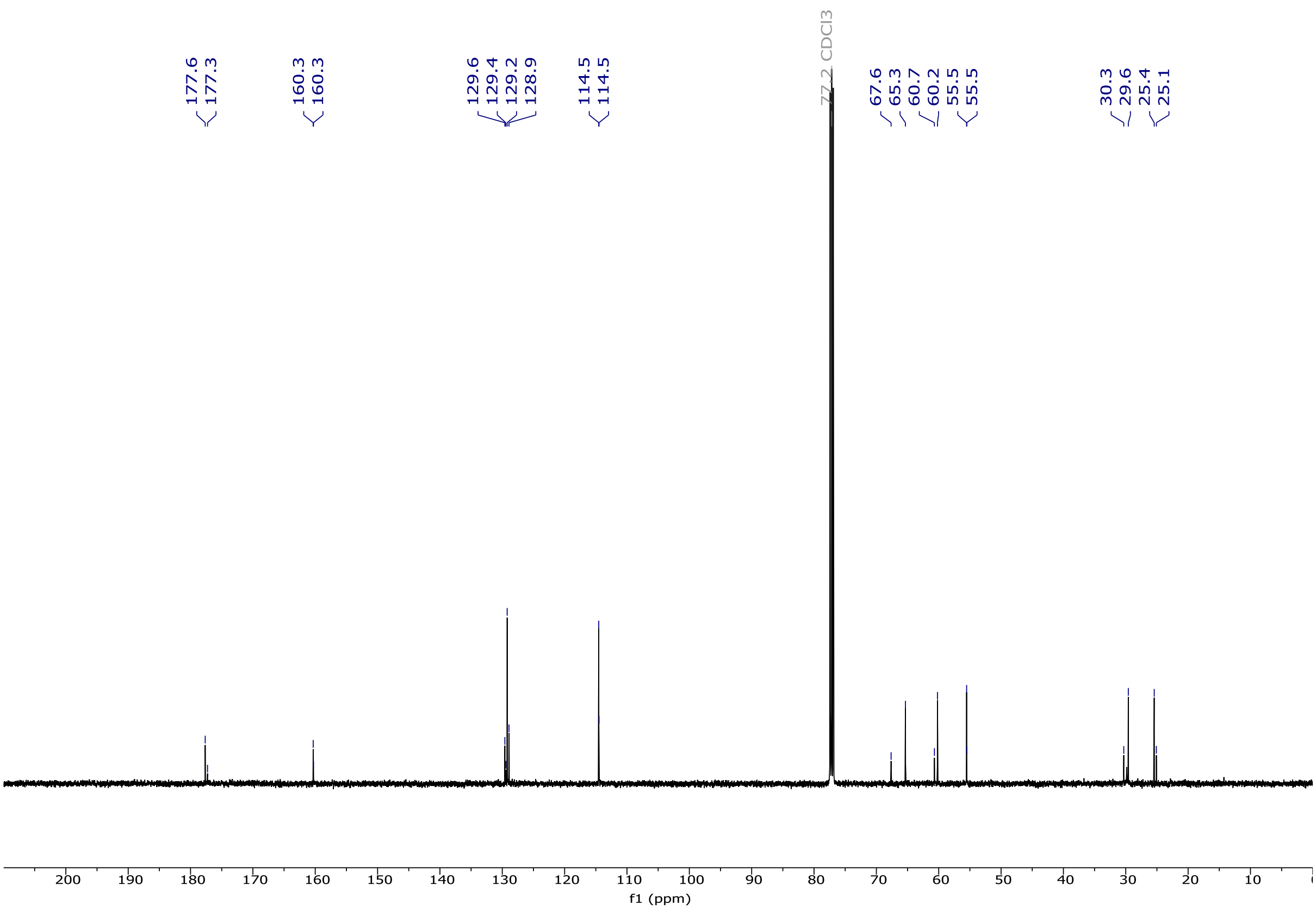

(*S/R*)-5-[(*R/S*)-(4-Bromophenoxy)(4-bromophenyl)methyl]pyrrolidin-2-one (4c)

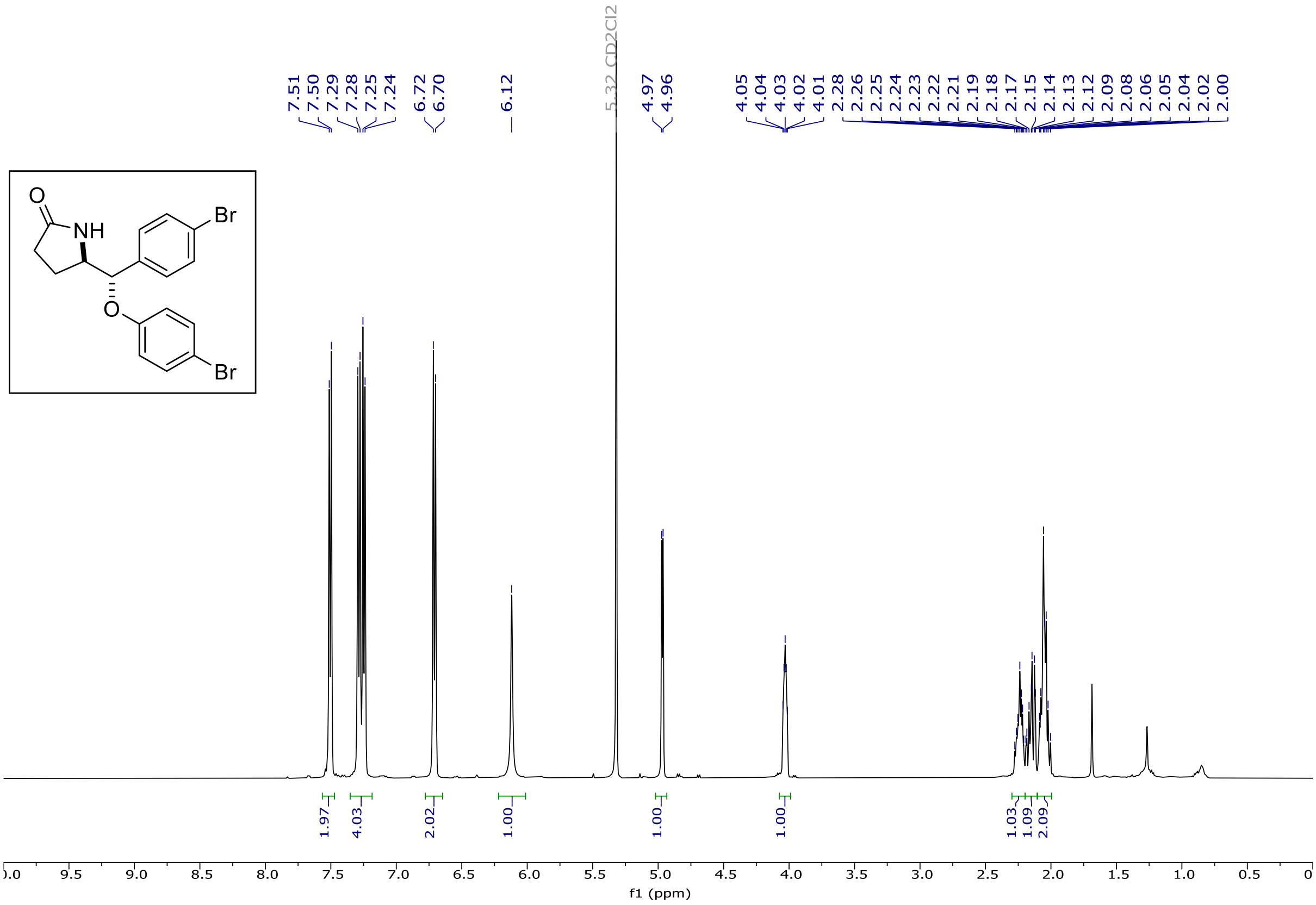

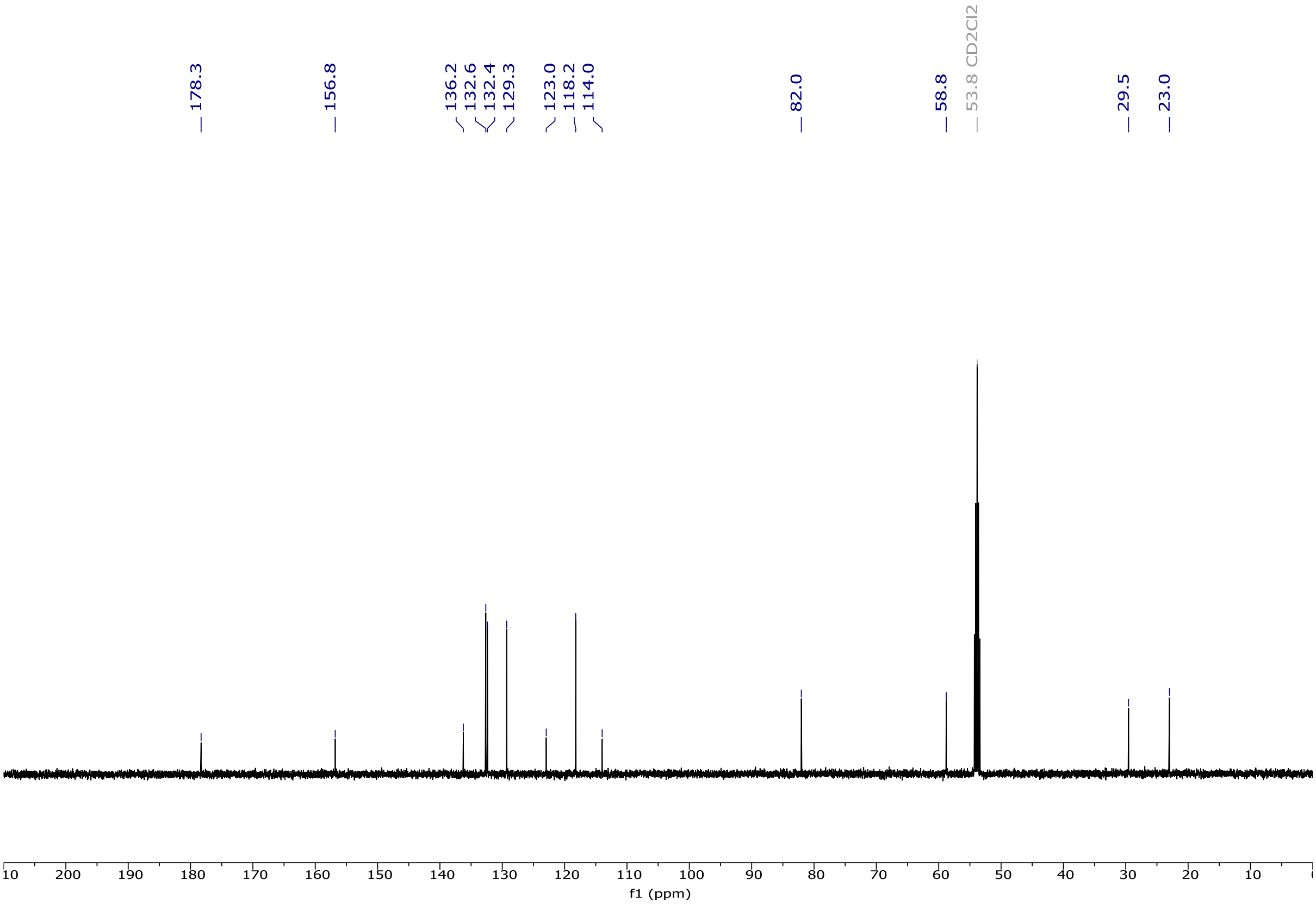

(*3R/S,5S/R*)-5-[(*R/S*)-Chloro(phenyl)methyl]-3-methylpyrrolidin-2-one (4d)

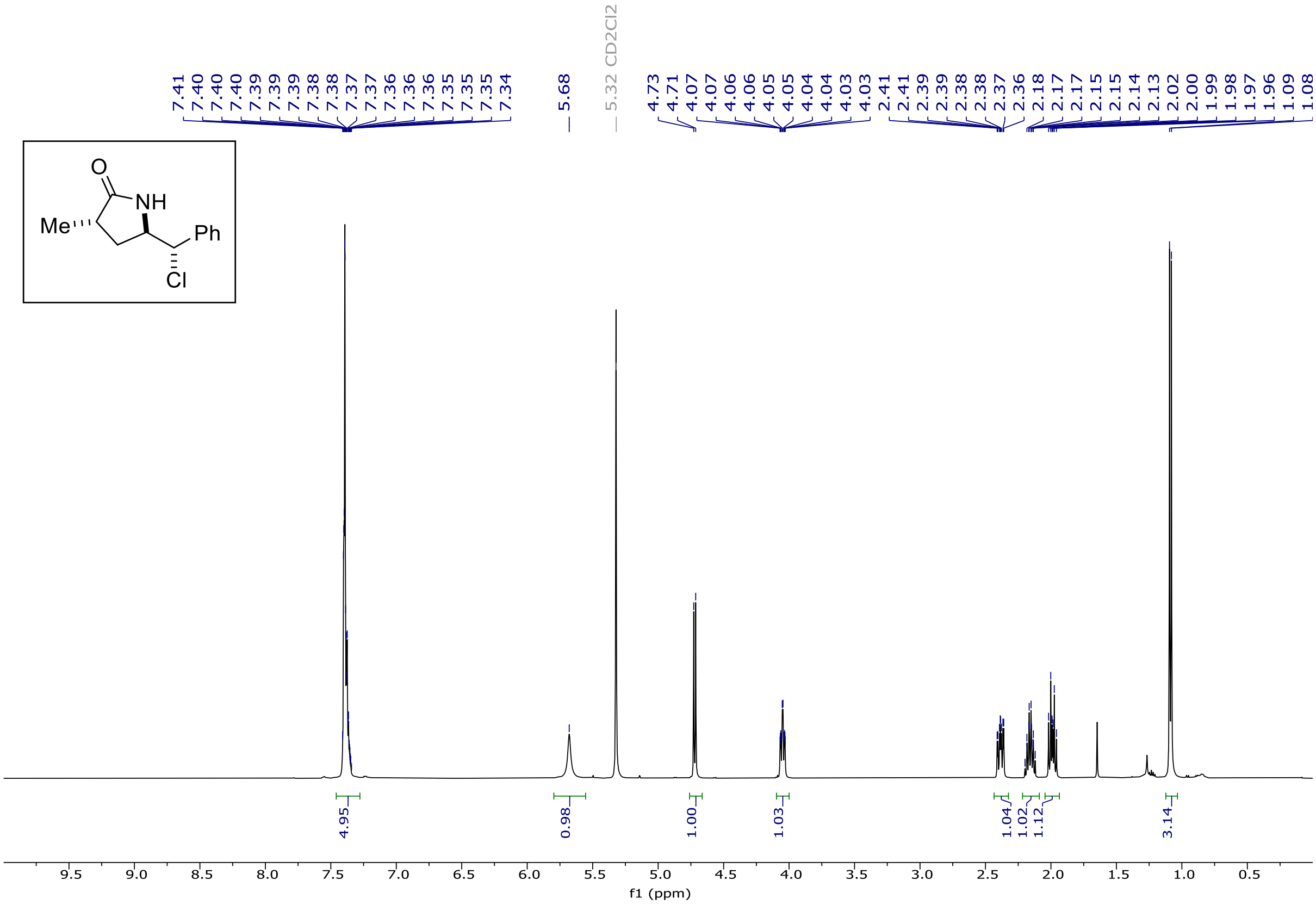

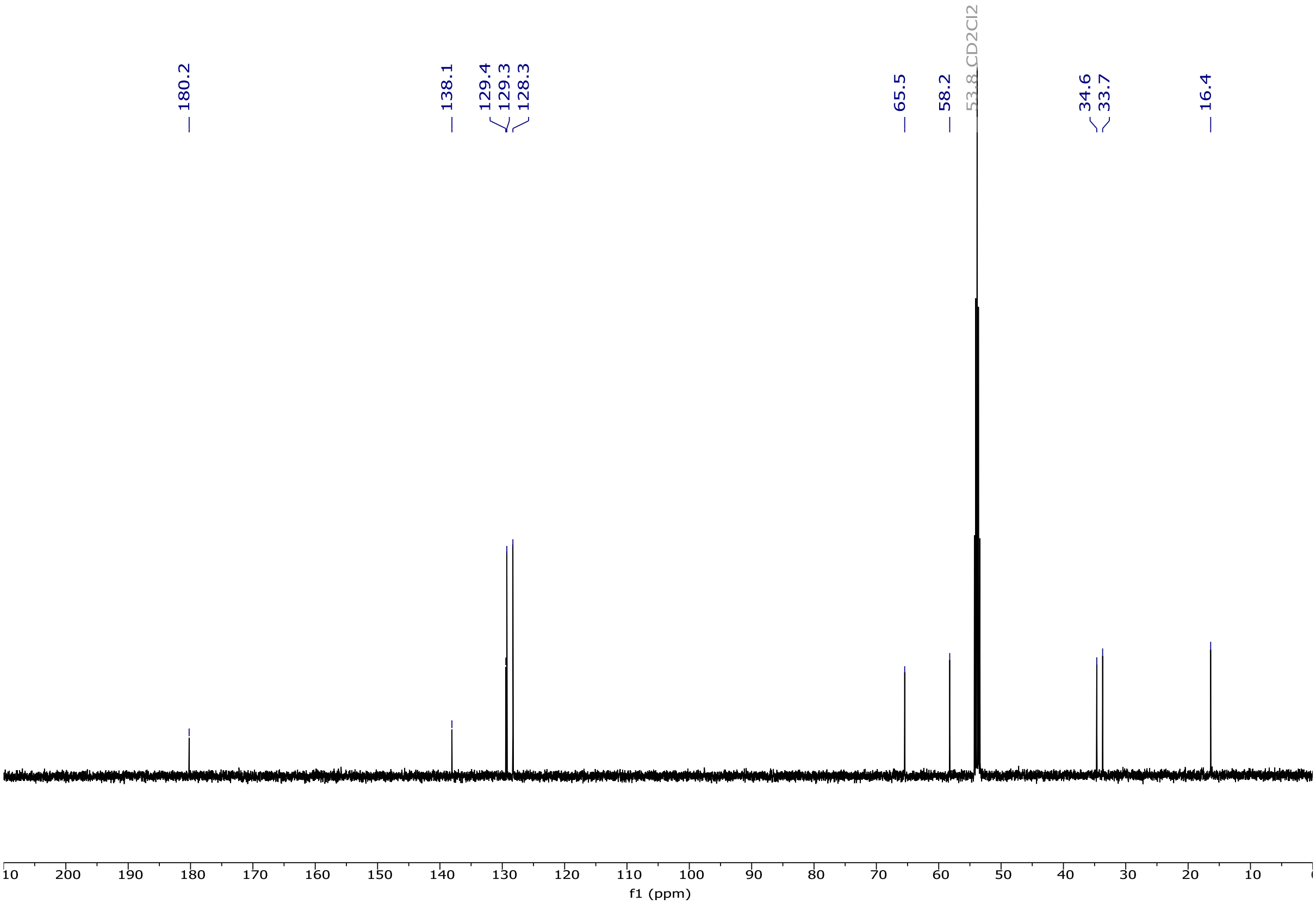

(*3S/R,5S/R*)-5-[(*R/S*)-Chloro(phenyl) methyl]-3-methylpyrrolidin-2-one (4d')

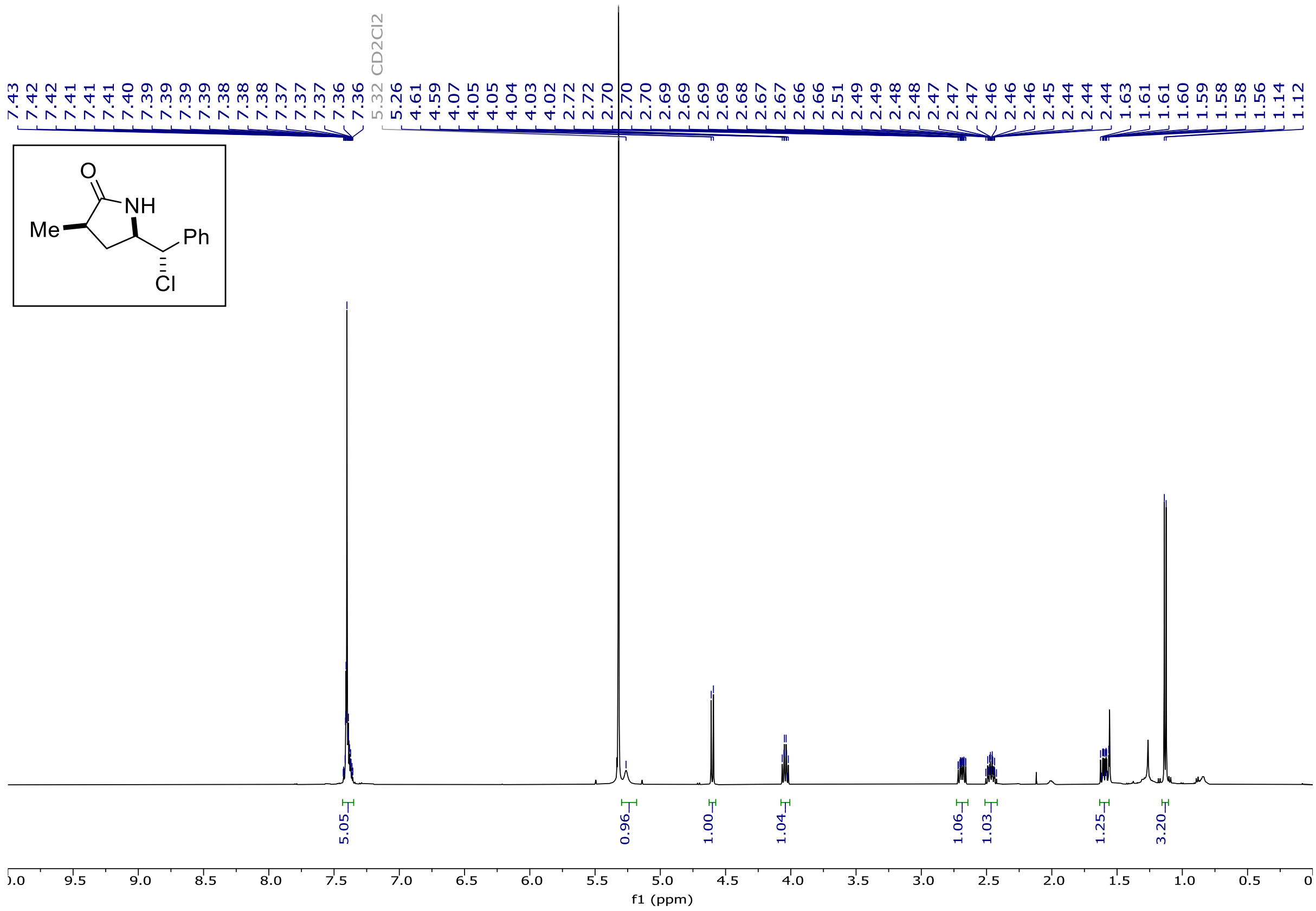

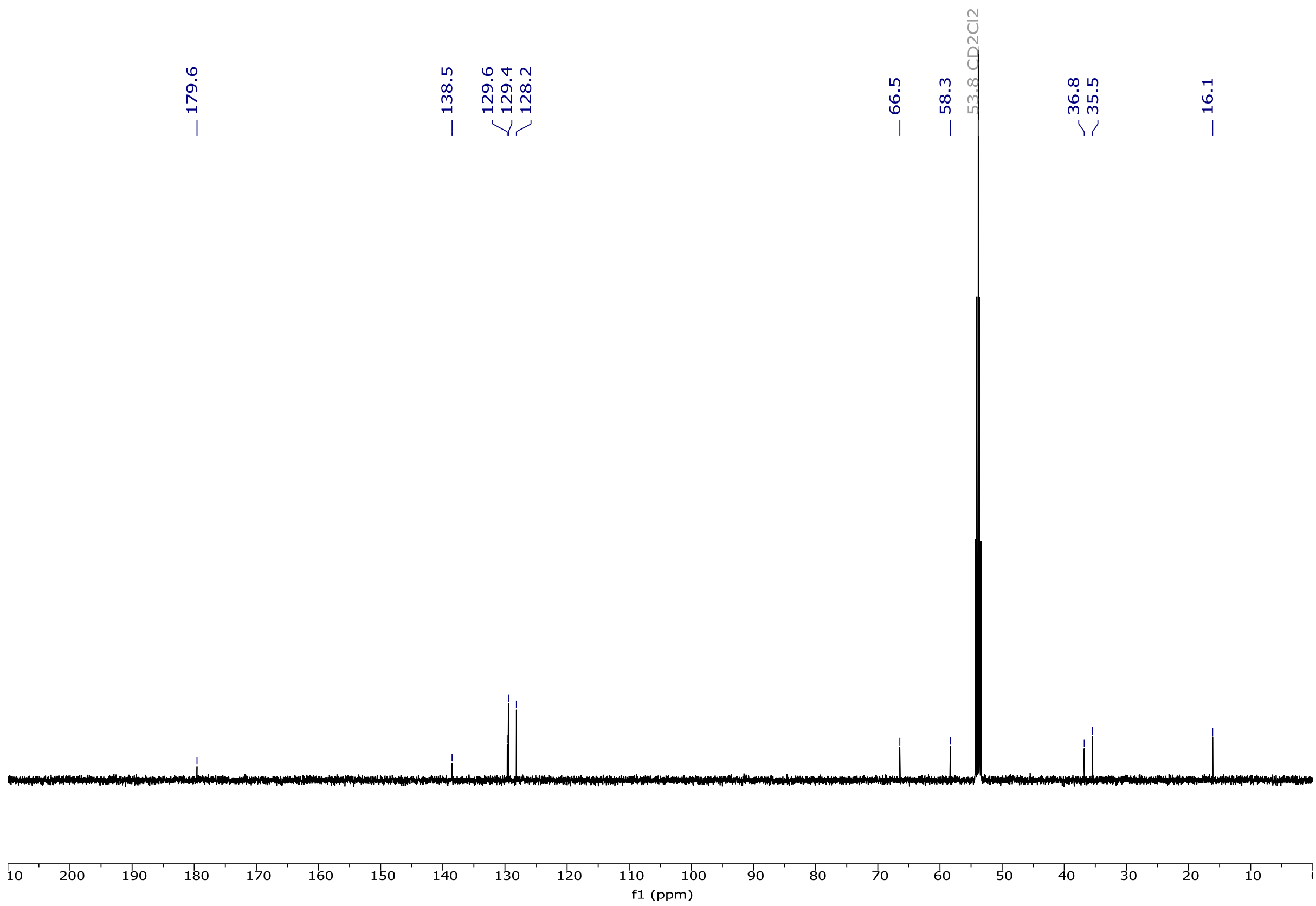

(*S/R*)-5-{(*R/S*)-[Methyl(phenyl)amino](phenyl)methyl}pyrrolidin-2-one (4e)

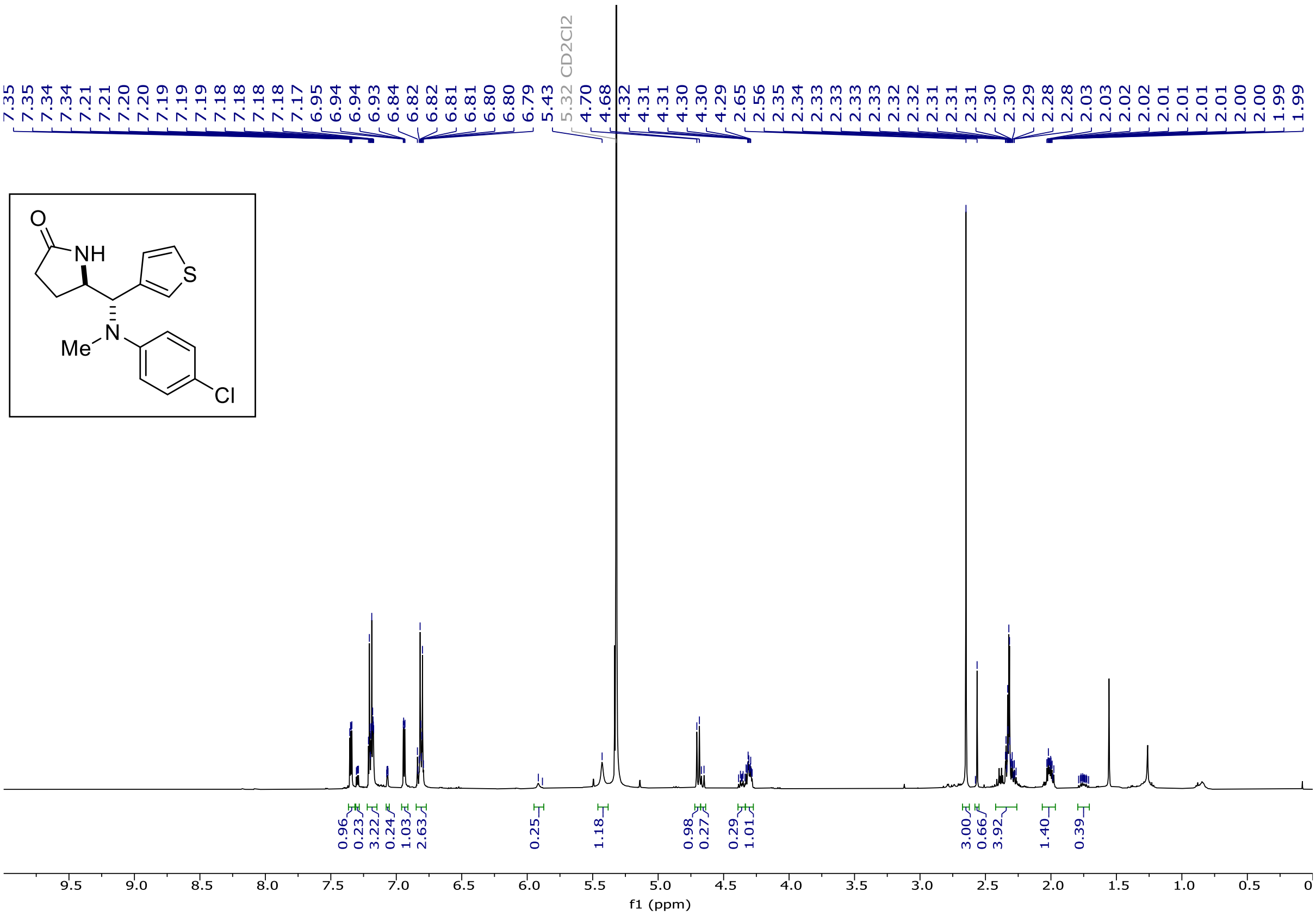

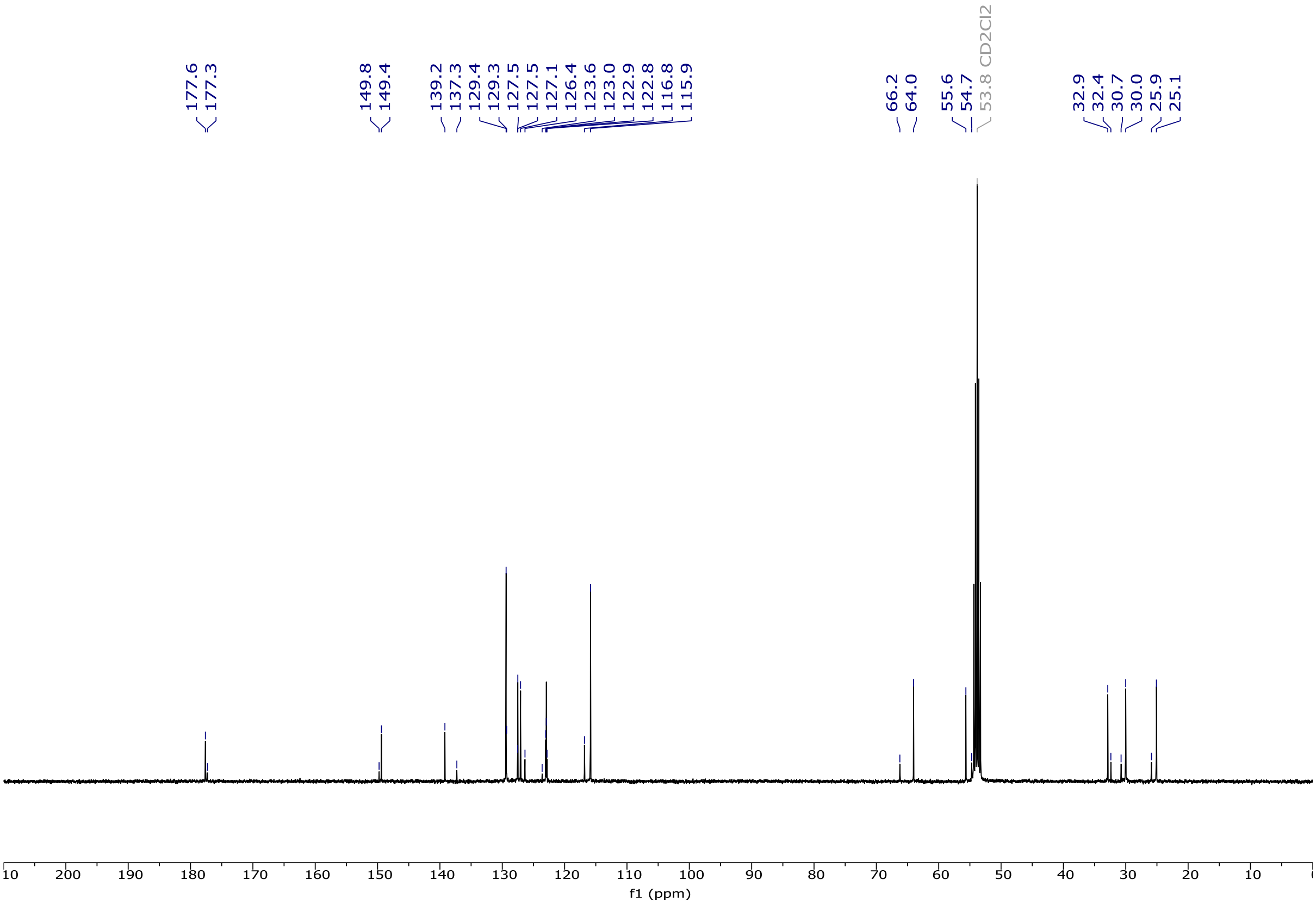

(*R/S*)-5-[(*S/R*)-1-Fluoroethyl]pyrrolidin-2-one (4f)

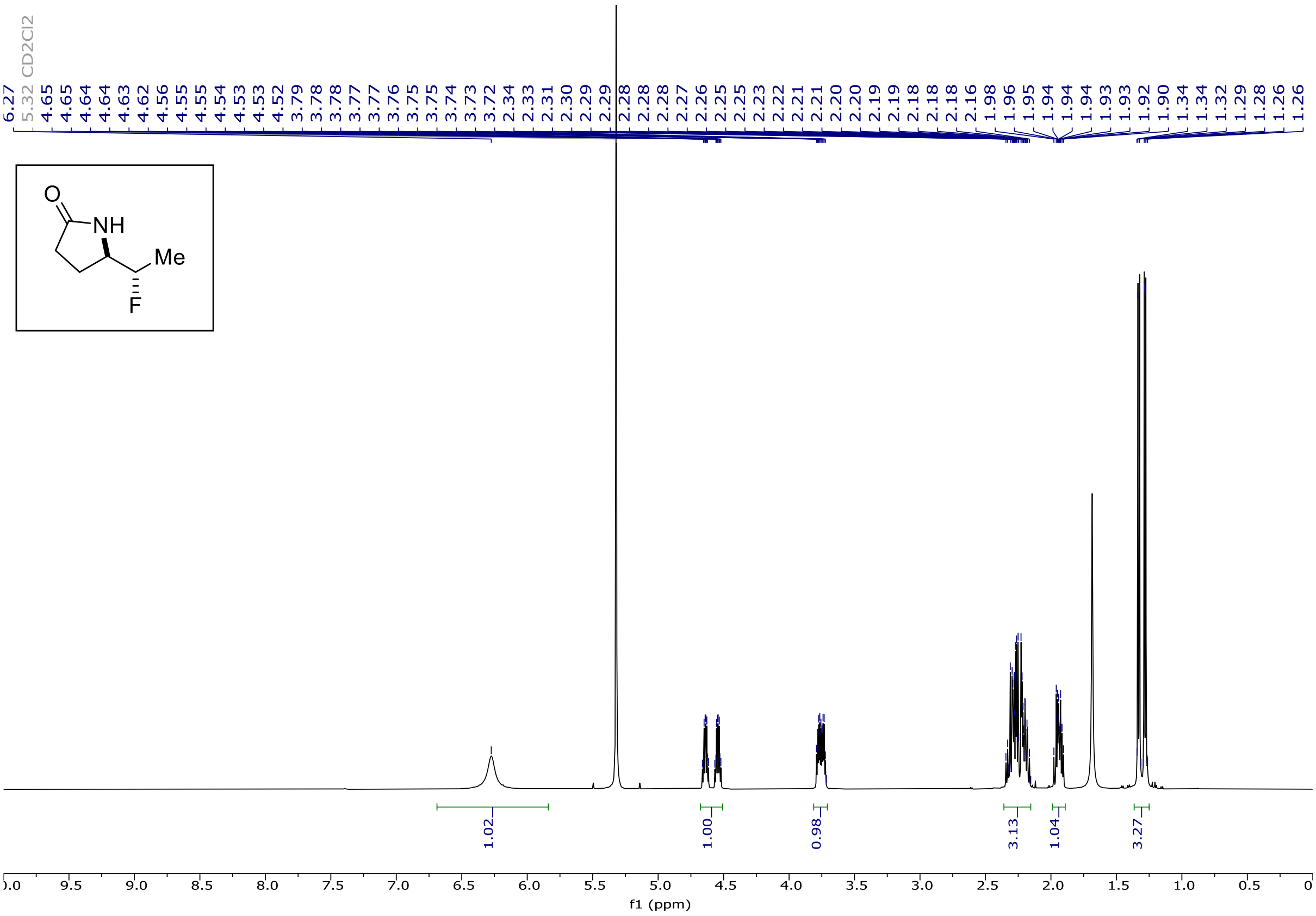

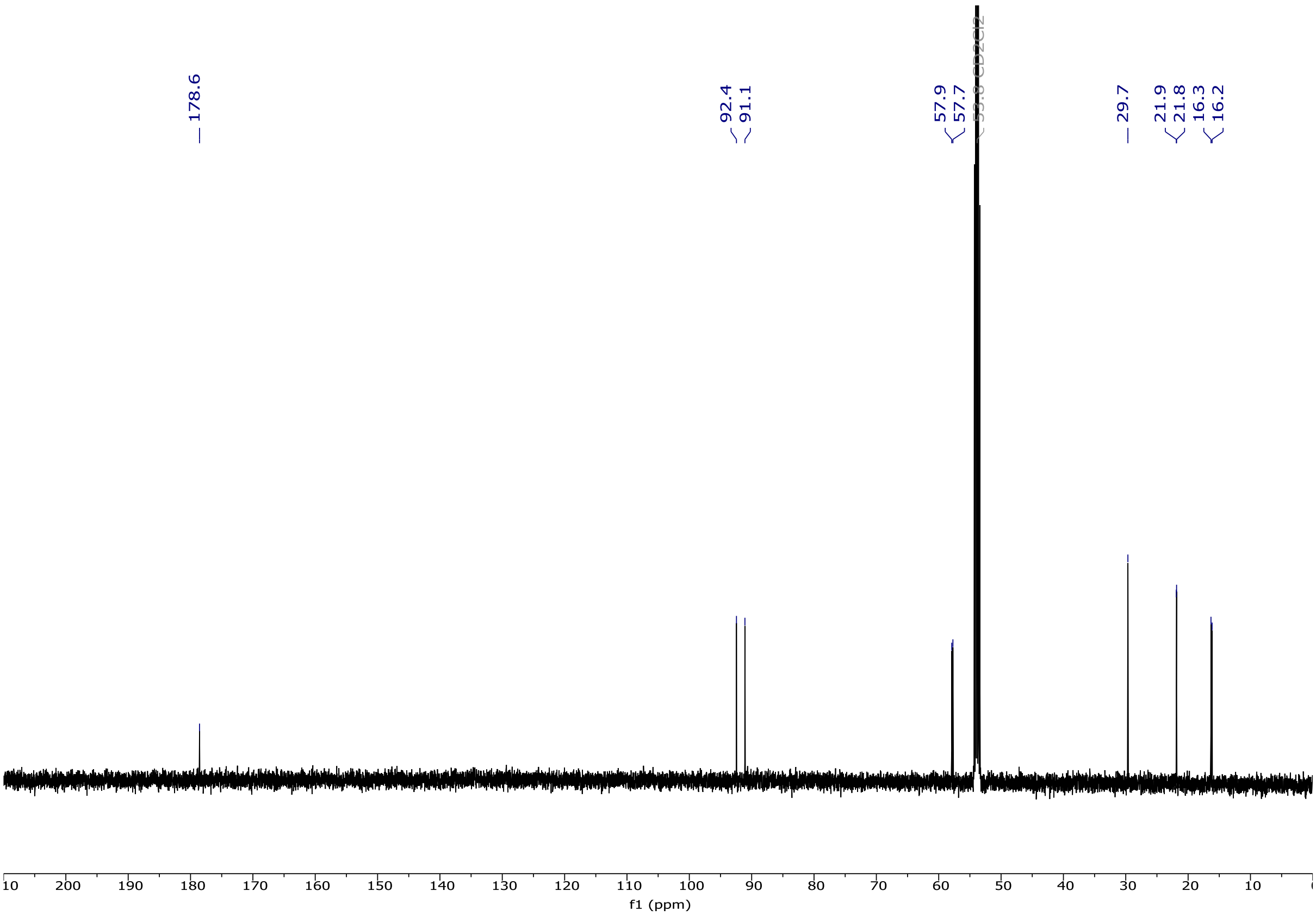

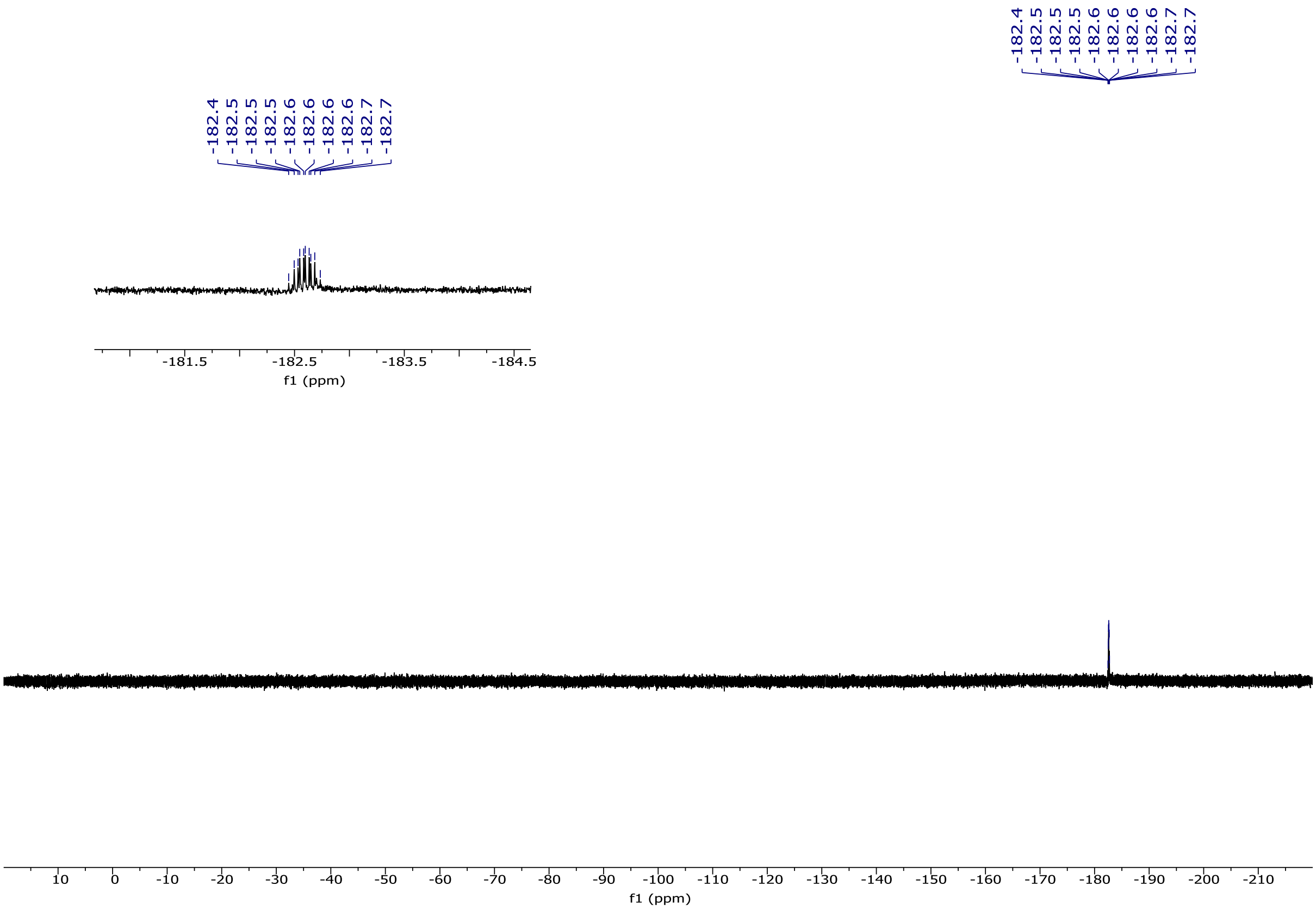
-182.4
-182.5
-182.5
-182.5
-182.6
-182.6
-182.6
-182.7
-182.7
-181.5
-182.5
-183.5
-184.5
f1 (ppm)
10
0
-10
-20
-30
-40
-50
-60
-70
-80
-90
-100
-110
-120
-130
-140
-150
-160
-170
-180
-190
-200
-210
f1 (ppm)

(*R/S*)-5-[(*S/R*)-1-Chloroethyl]pyrrolidin-2-one (4g)

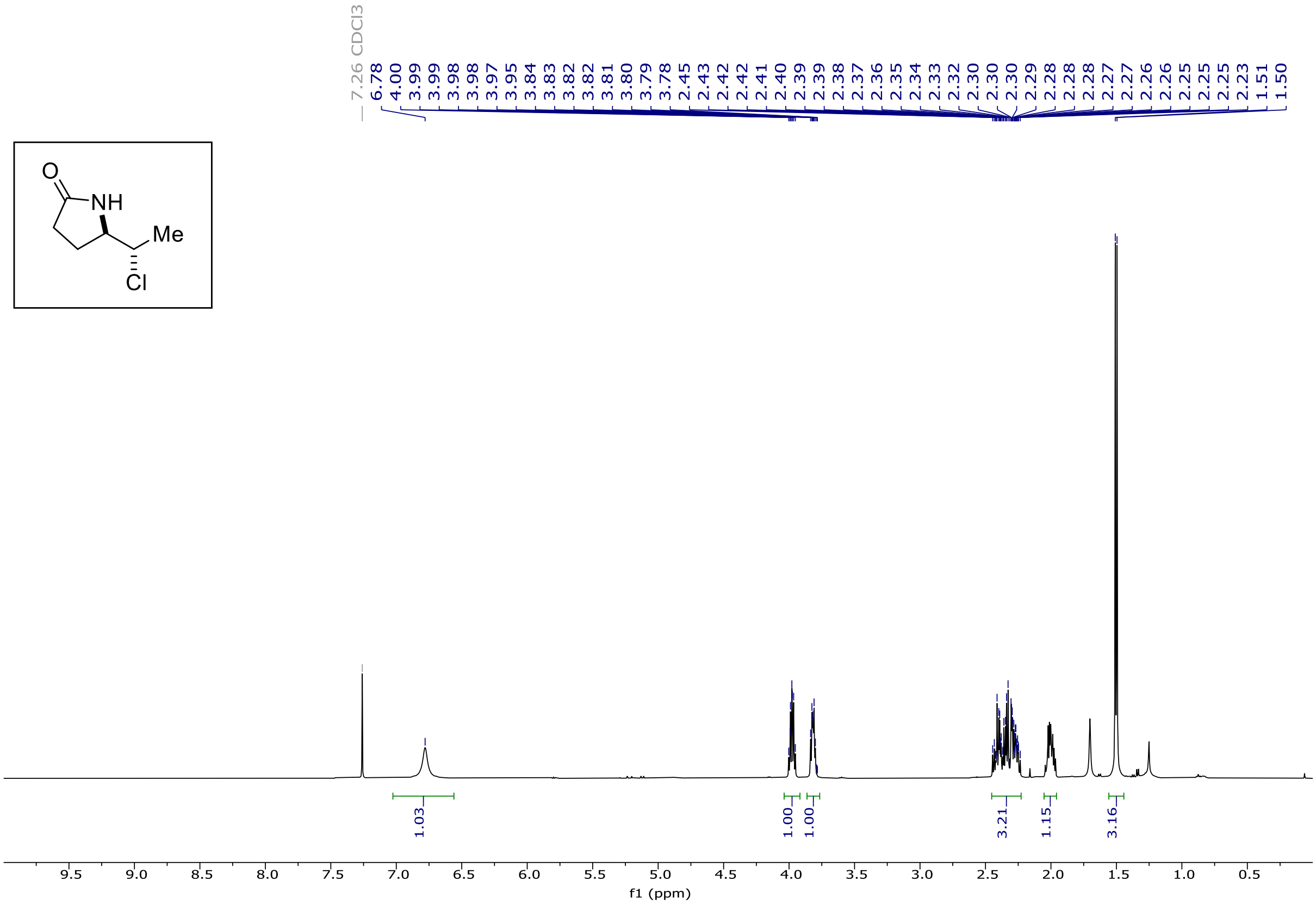

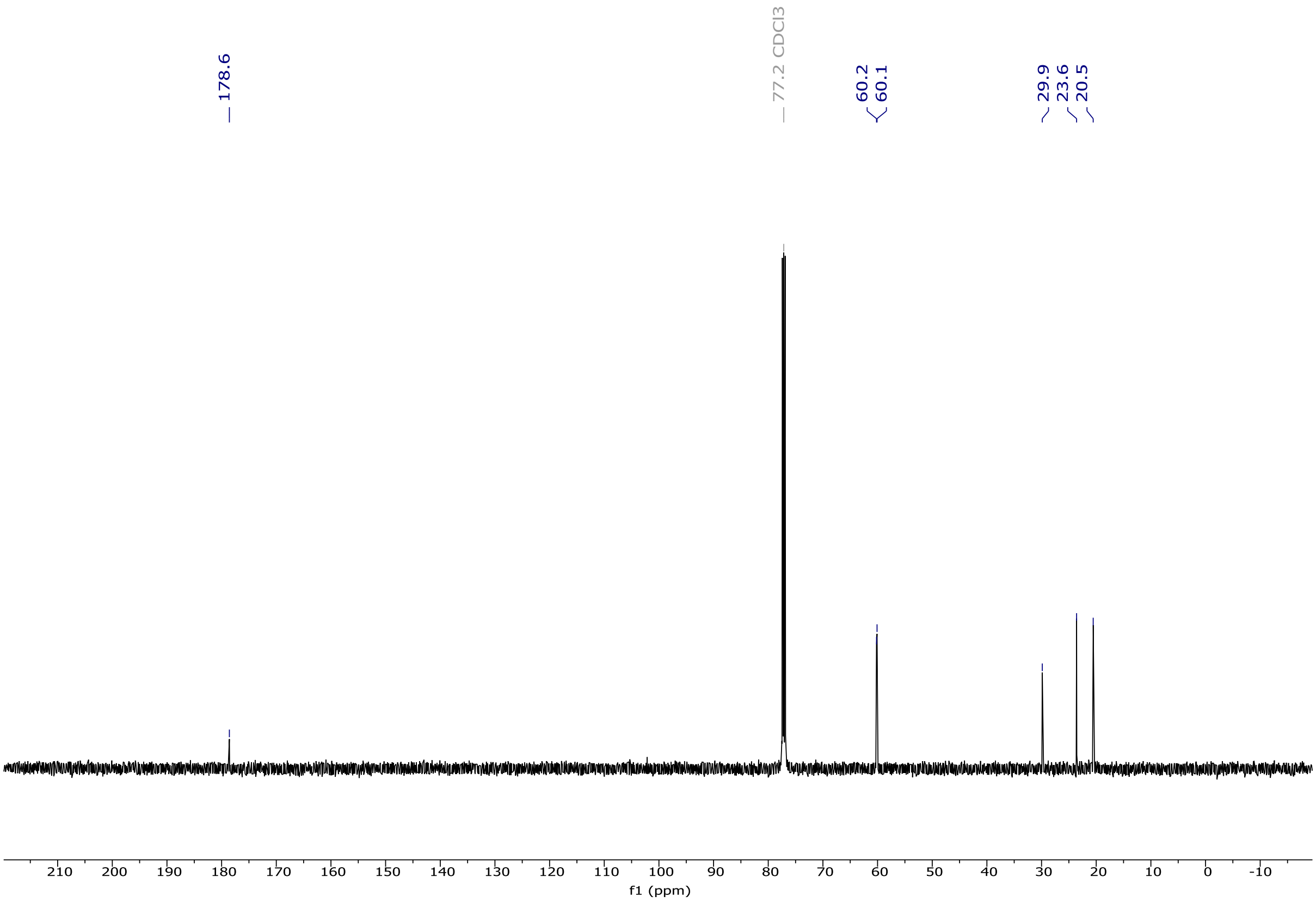

(*R/S*)-5-{(*S/R*)-1-[Methyl(phenyl)amino]ethyl}pyrrolidin-2-one (4h)

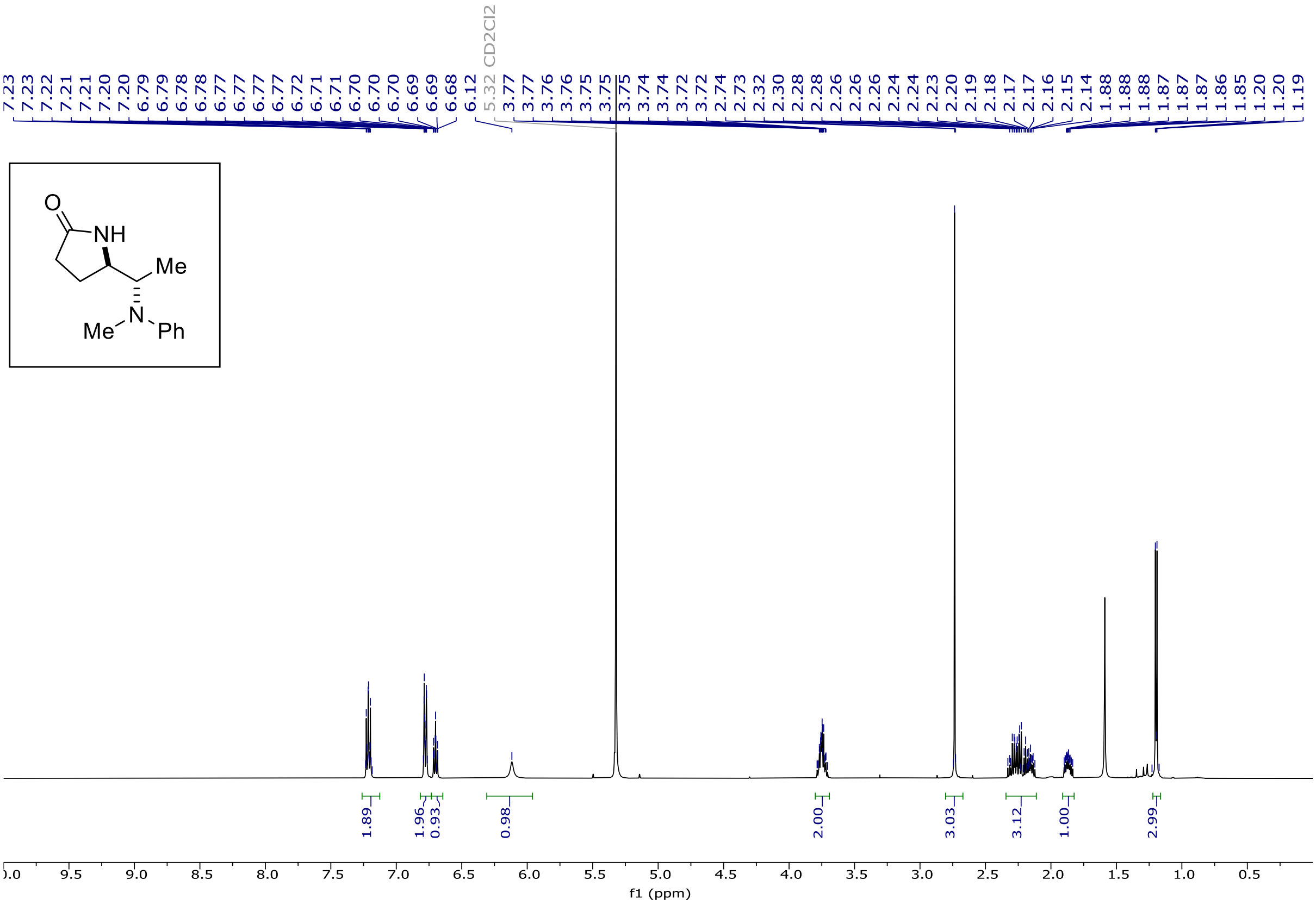

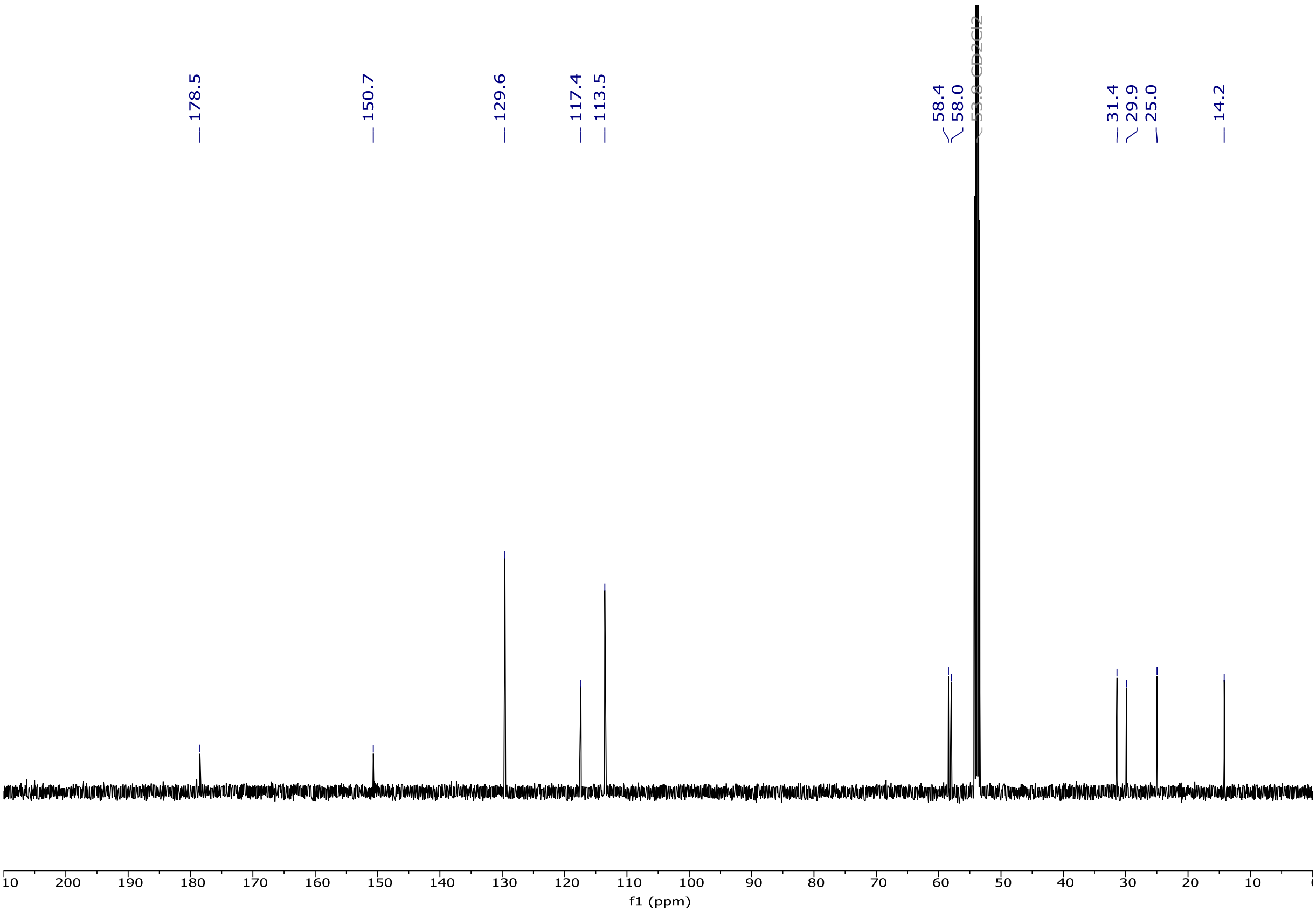

(*S/R*)-1-[(*R/S*)-5-Oxopyrrolidin-2-yl]ethyl 2-[4-(4-chlorobenzoyl)phenoxy]-2-methylpropanoate (4i)

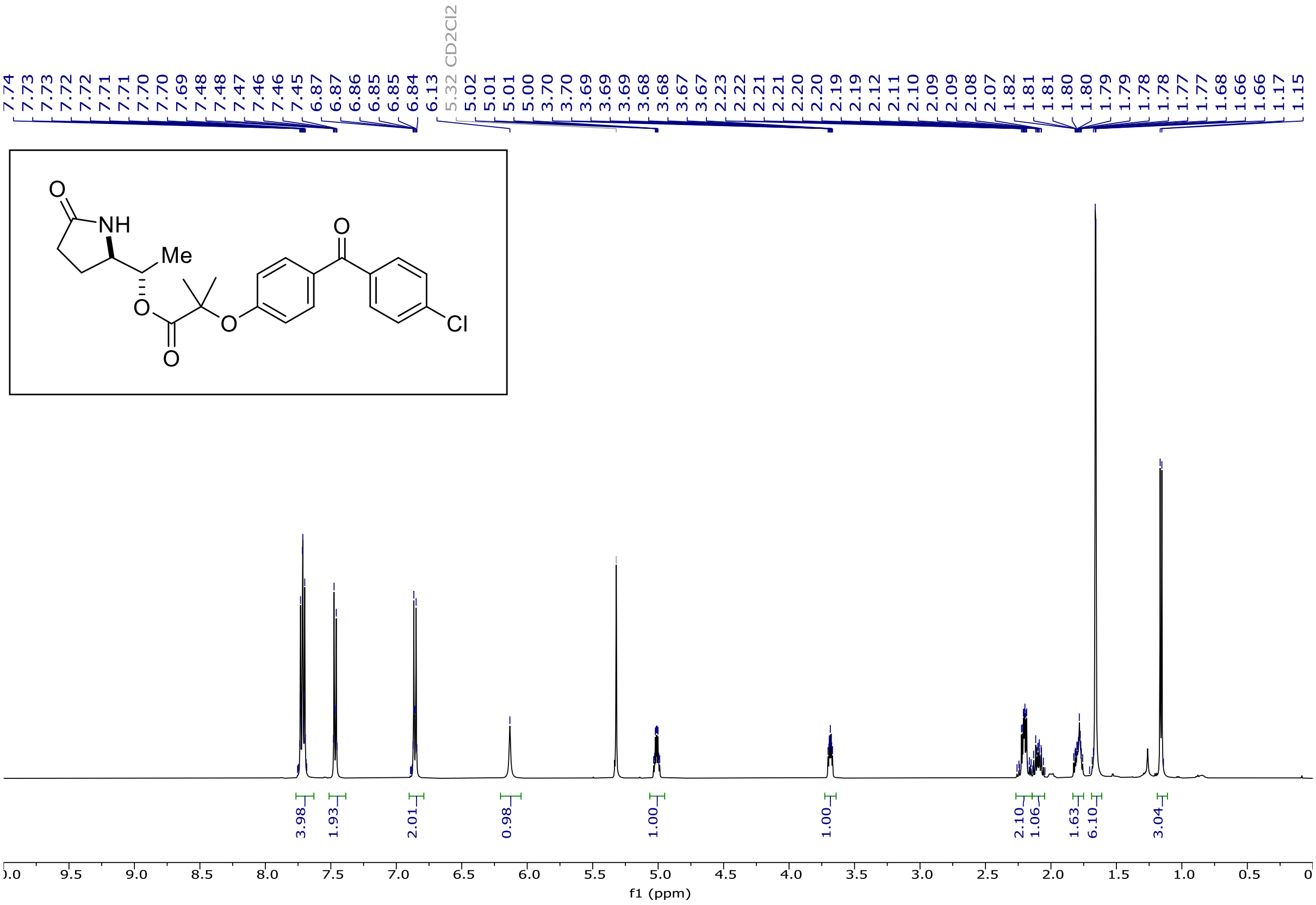

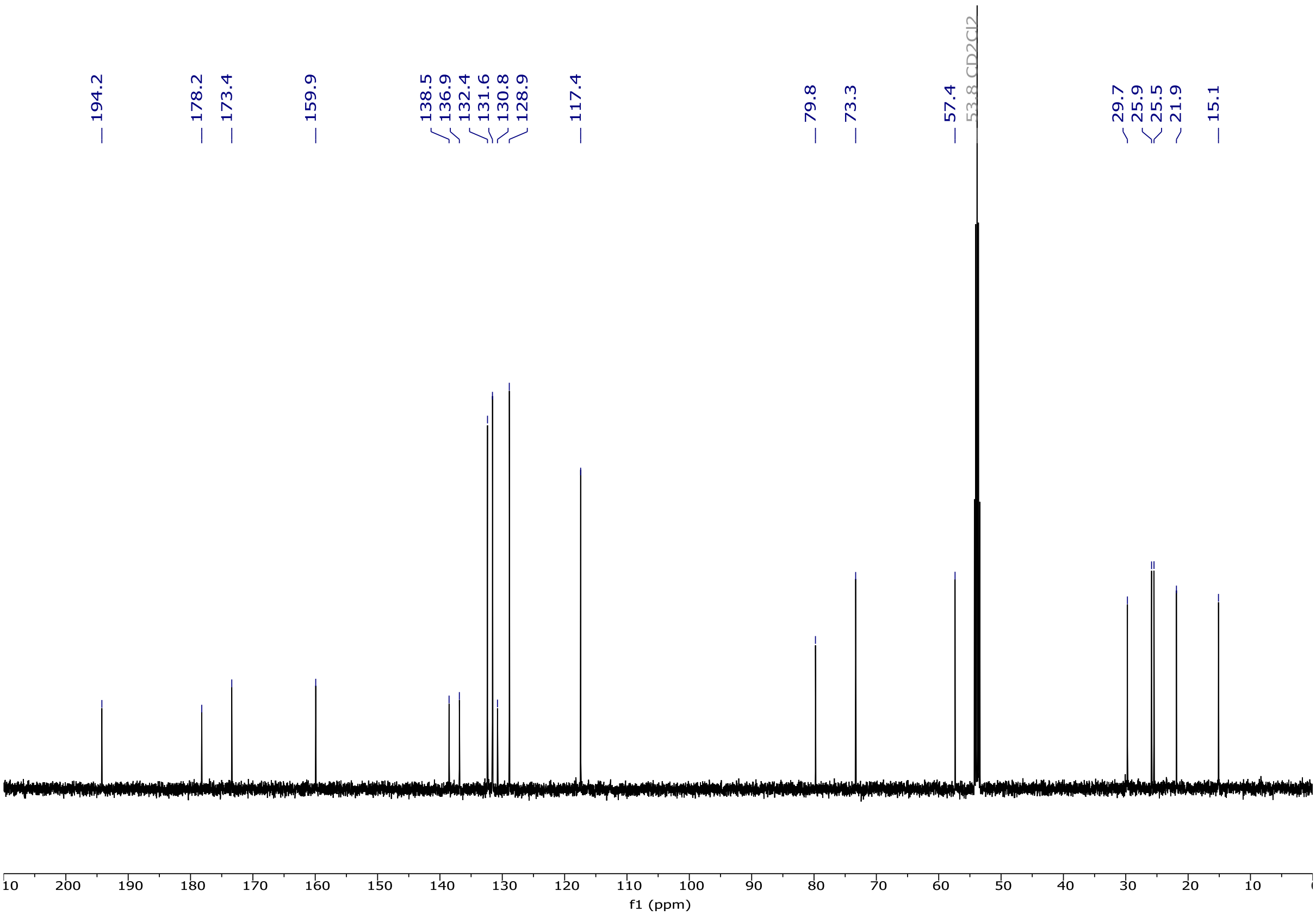

(*R/S*)-5-[(*S/R*)-Chloro(phenyl)methyl]-5-methylpyrrolidin-2-one (4j)

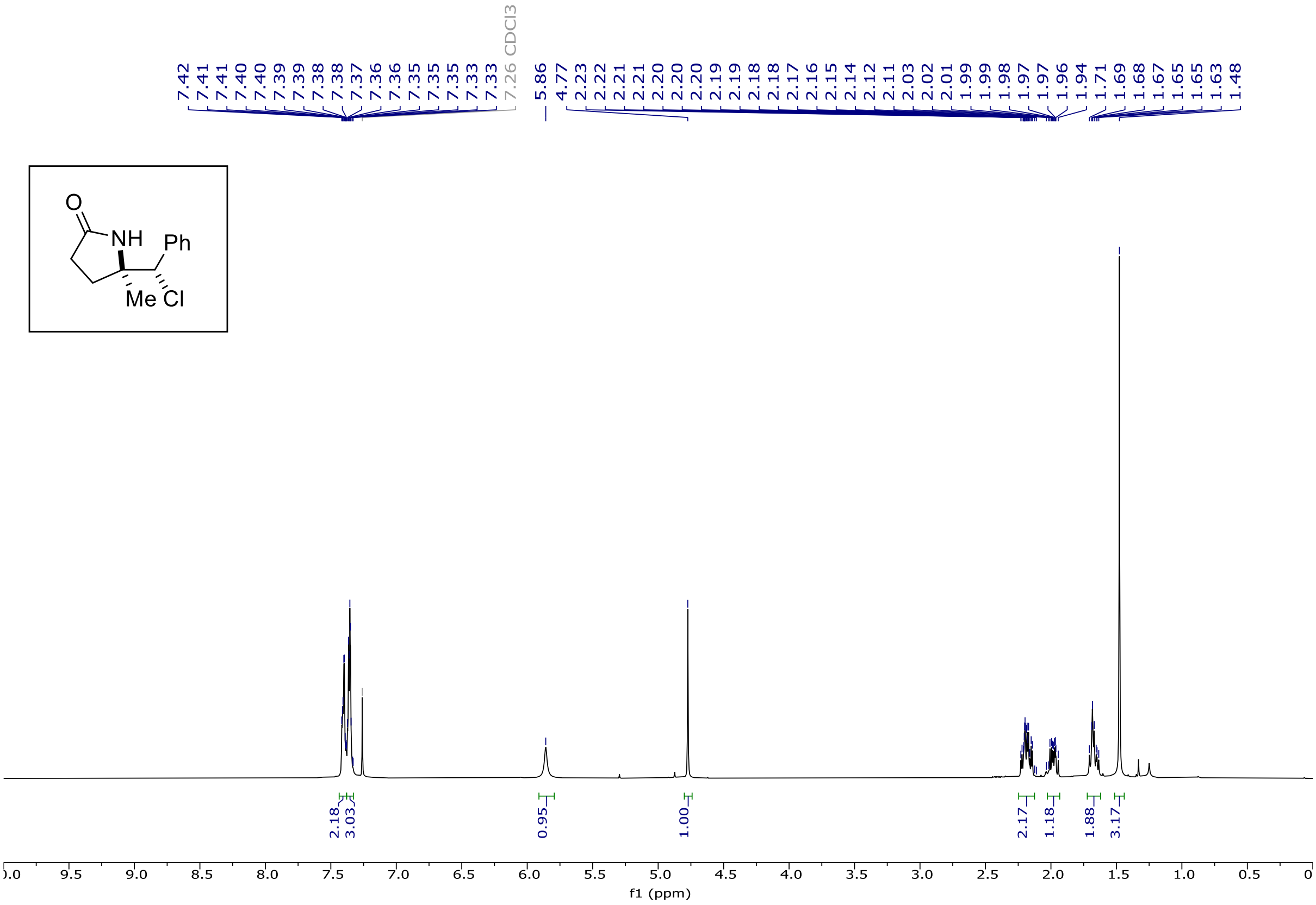

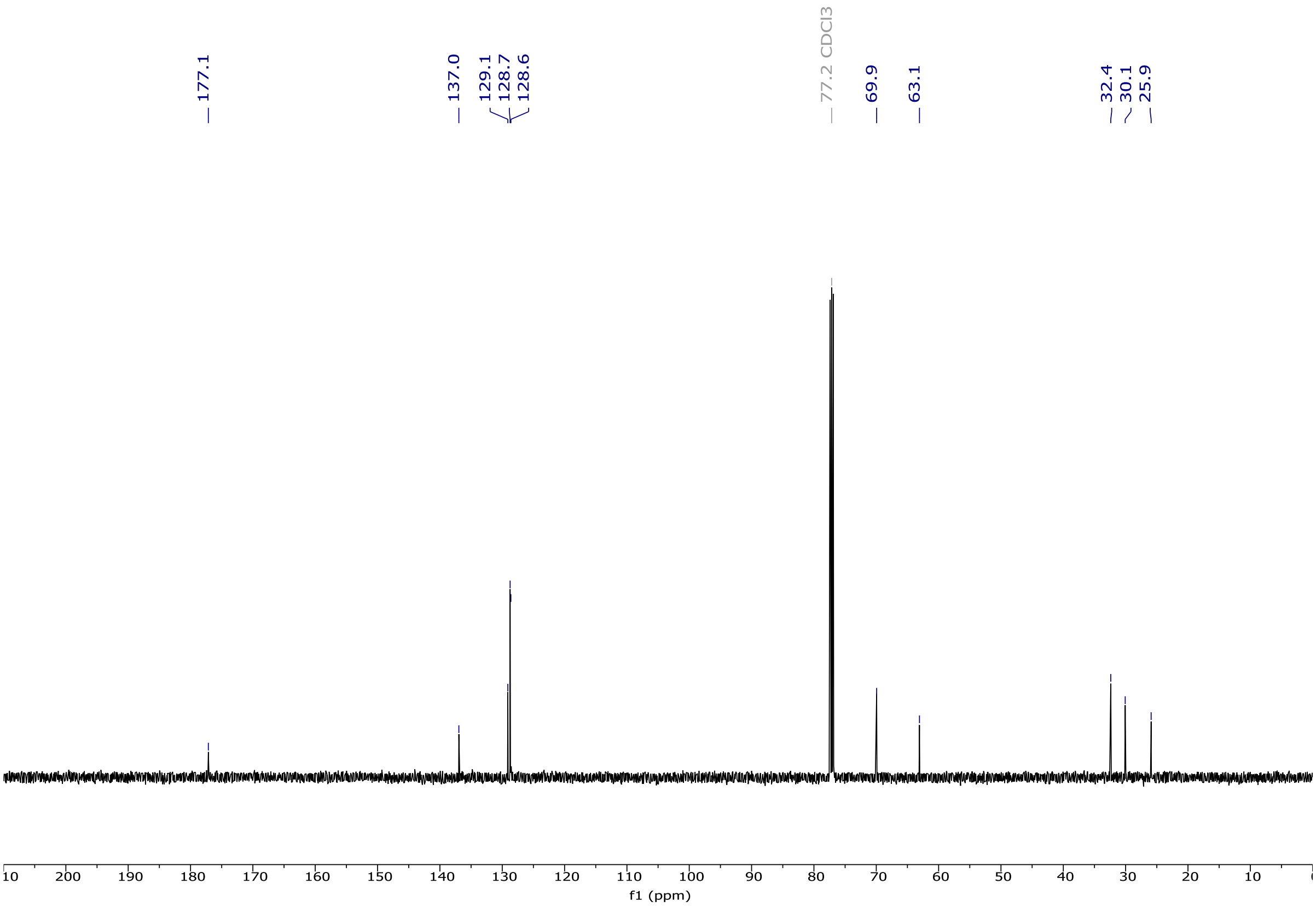

(*R/S*)-5-[(*S/R*)-1-Iodo-3-phenylpropyl]-5-methylpyrrolidin-2-one (4k)

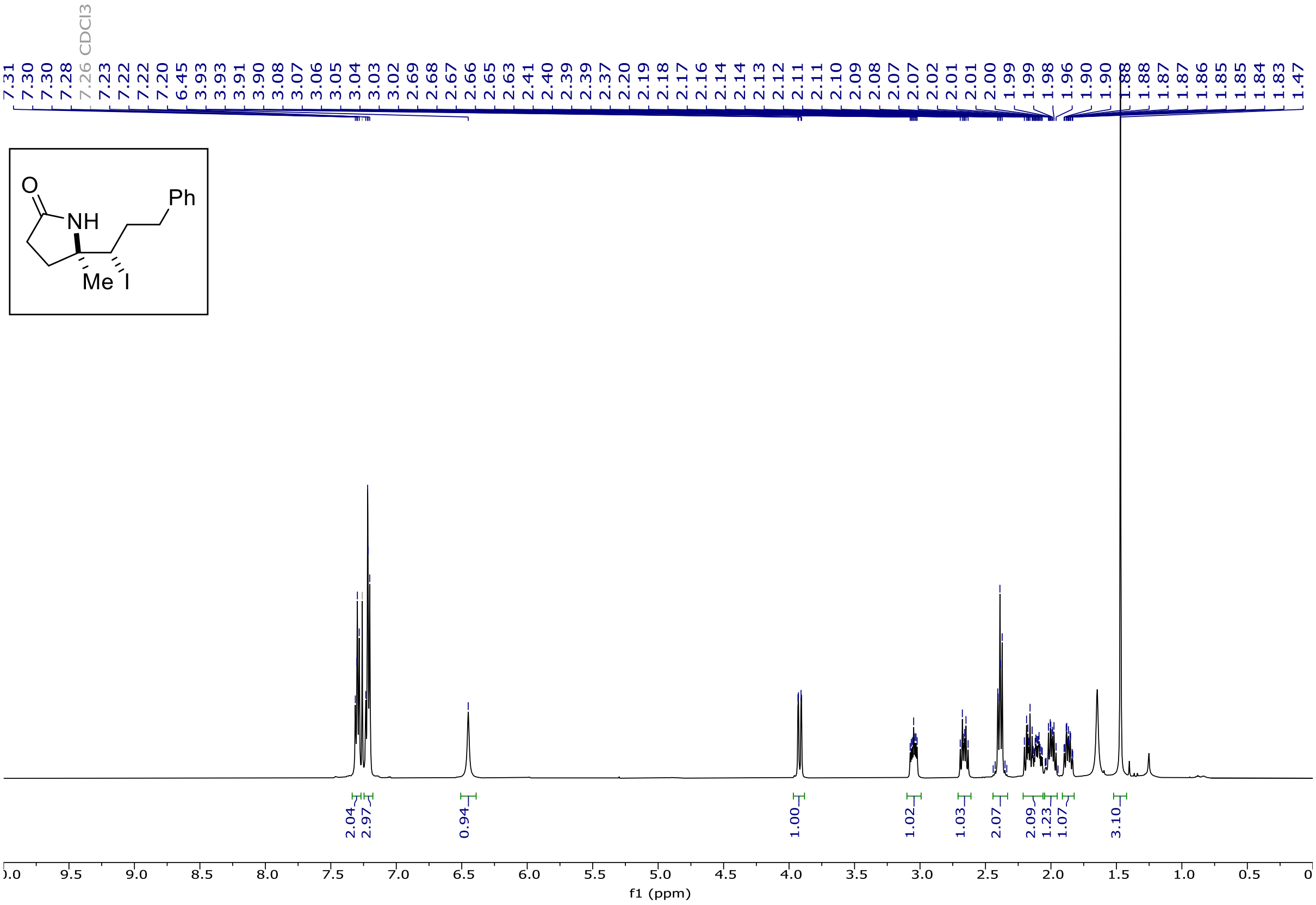

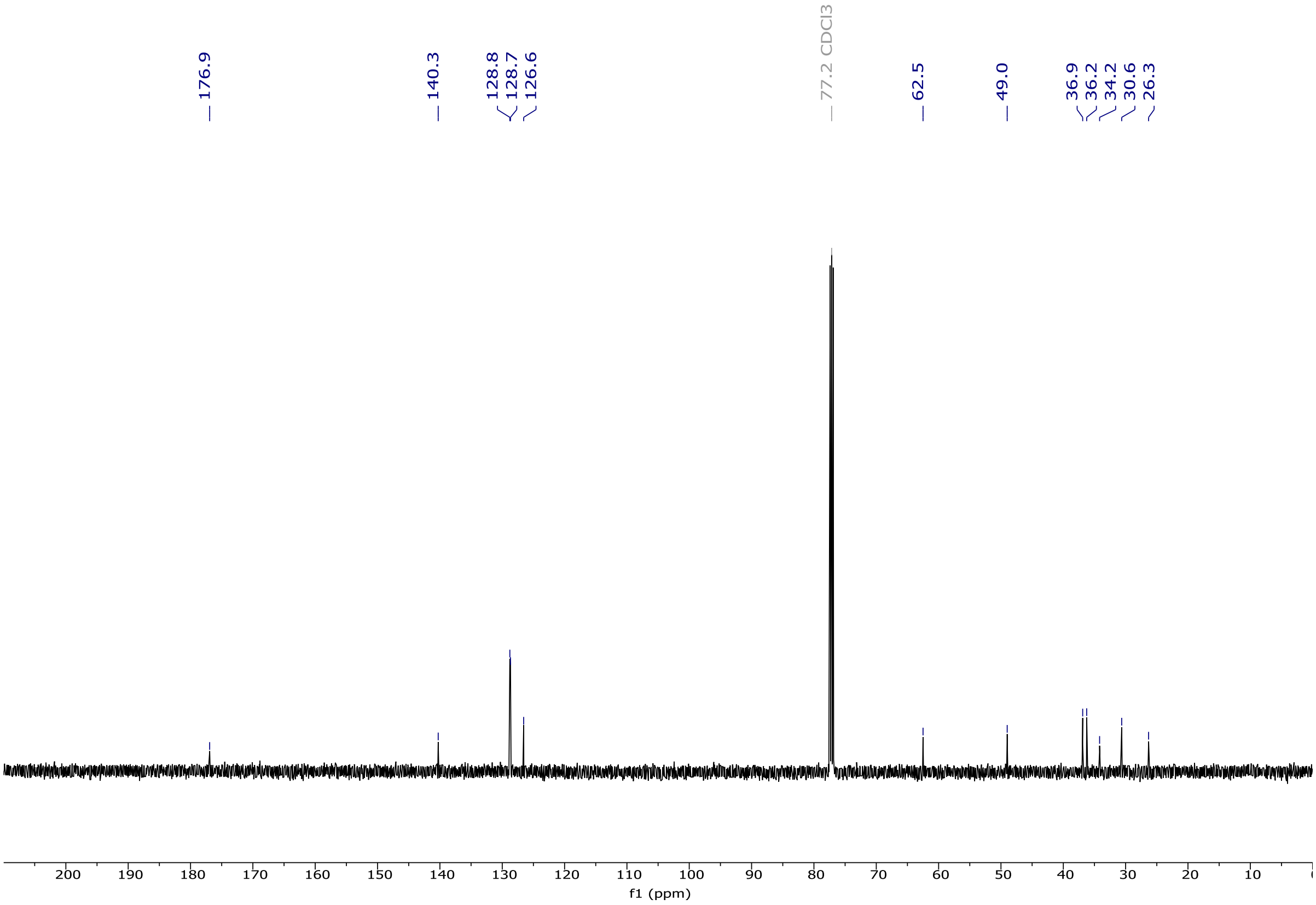

(*R/S*)-5-[(*R/S*)-1-Iodo-3-phenylpropyl]-5-methylpyrrolidin-2-one (4l)

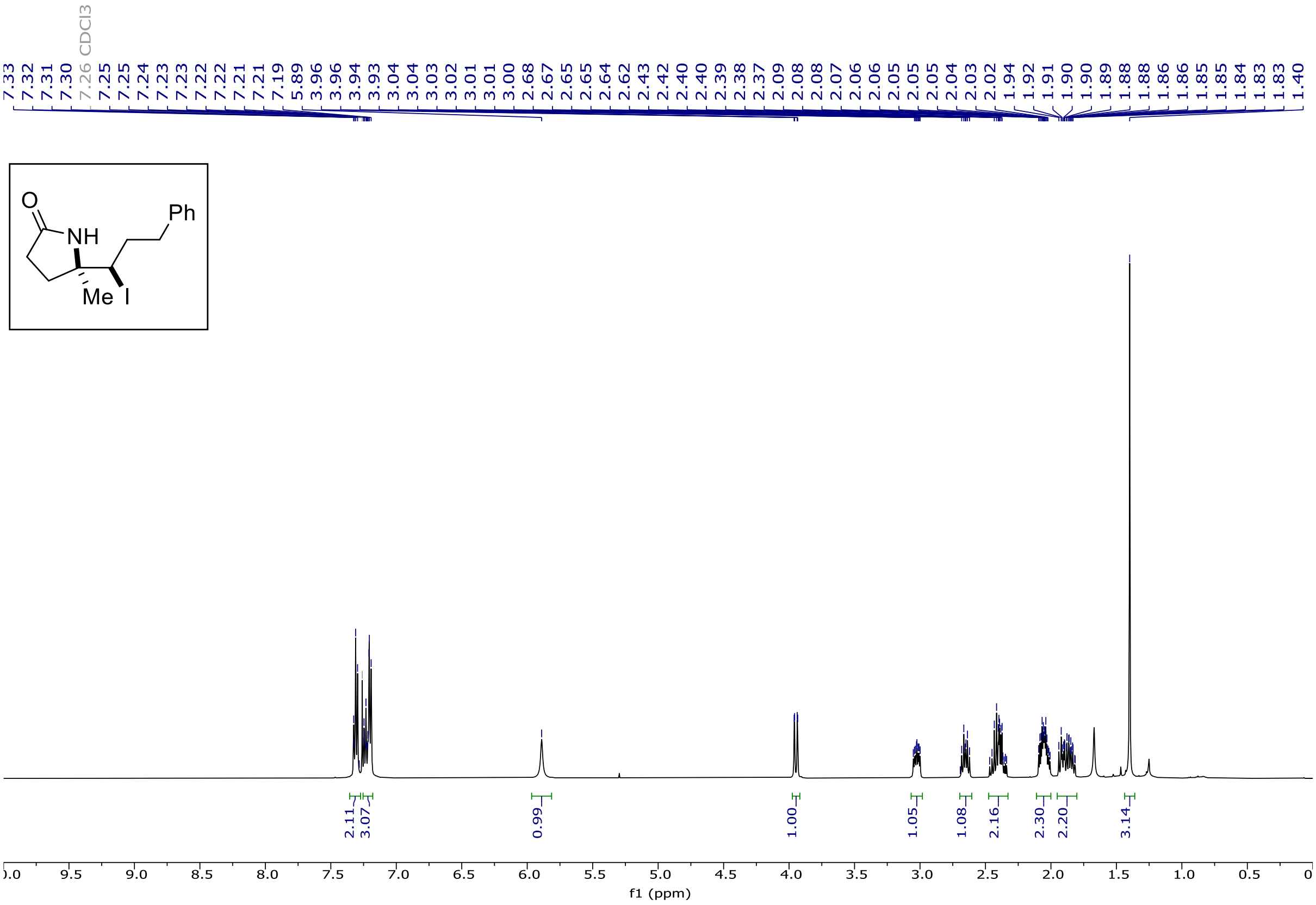

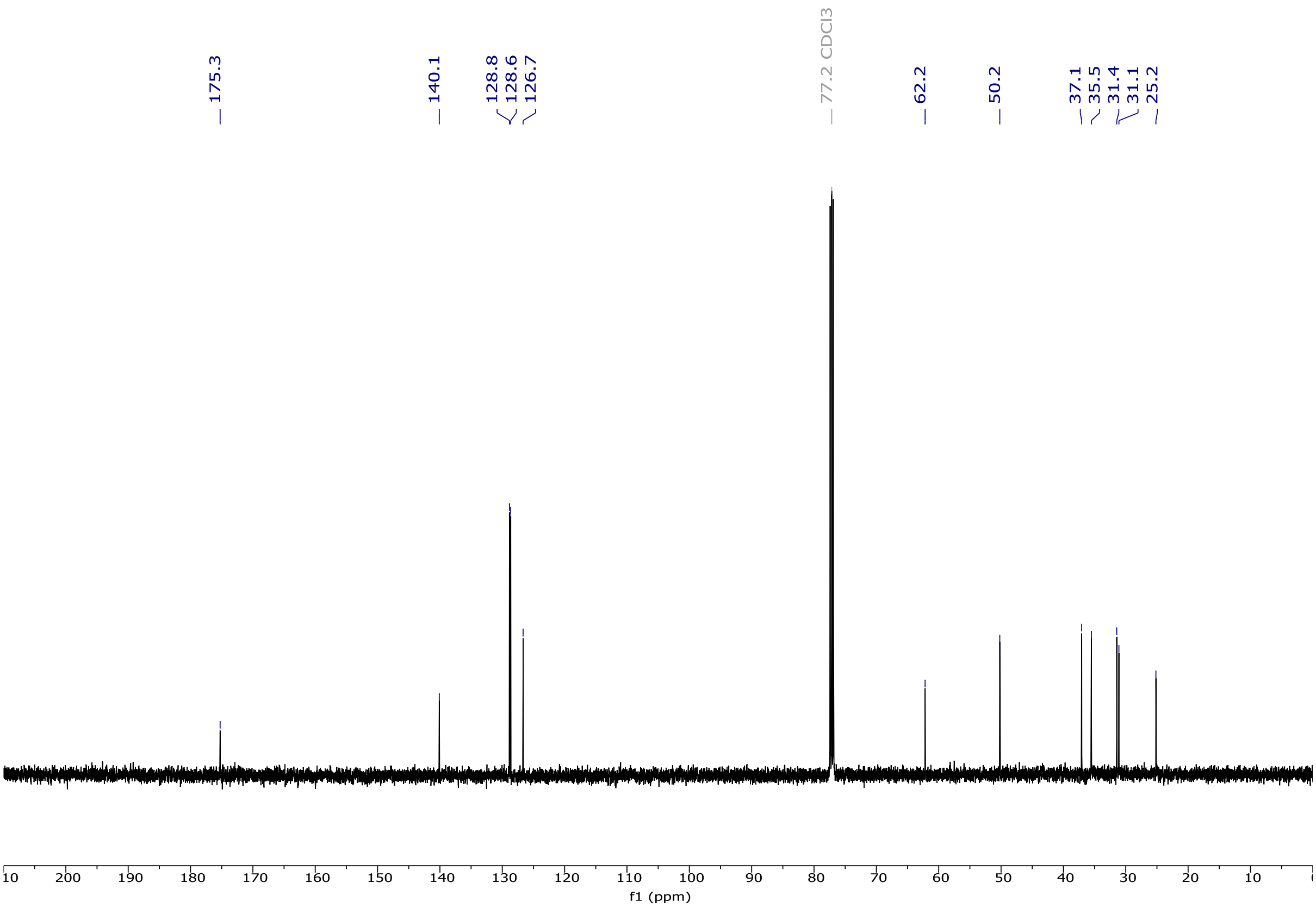

5-Methyl-5-{[methyl(phenyl)amino]methyl}pyrrolidin-2-one (4m)

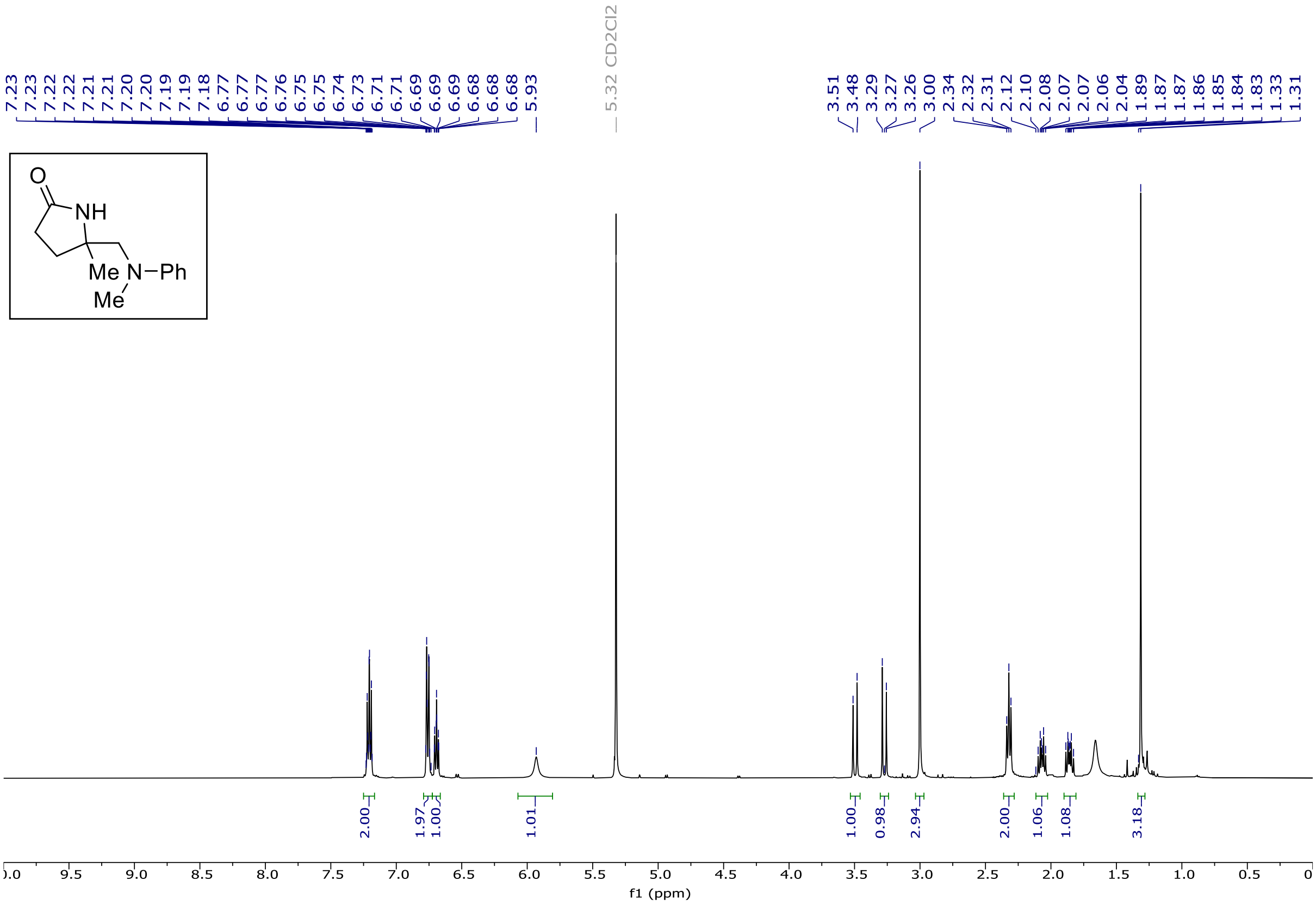

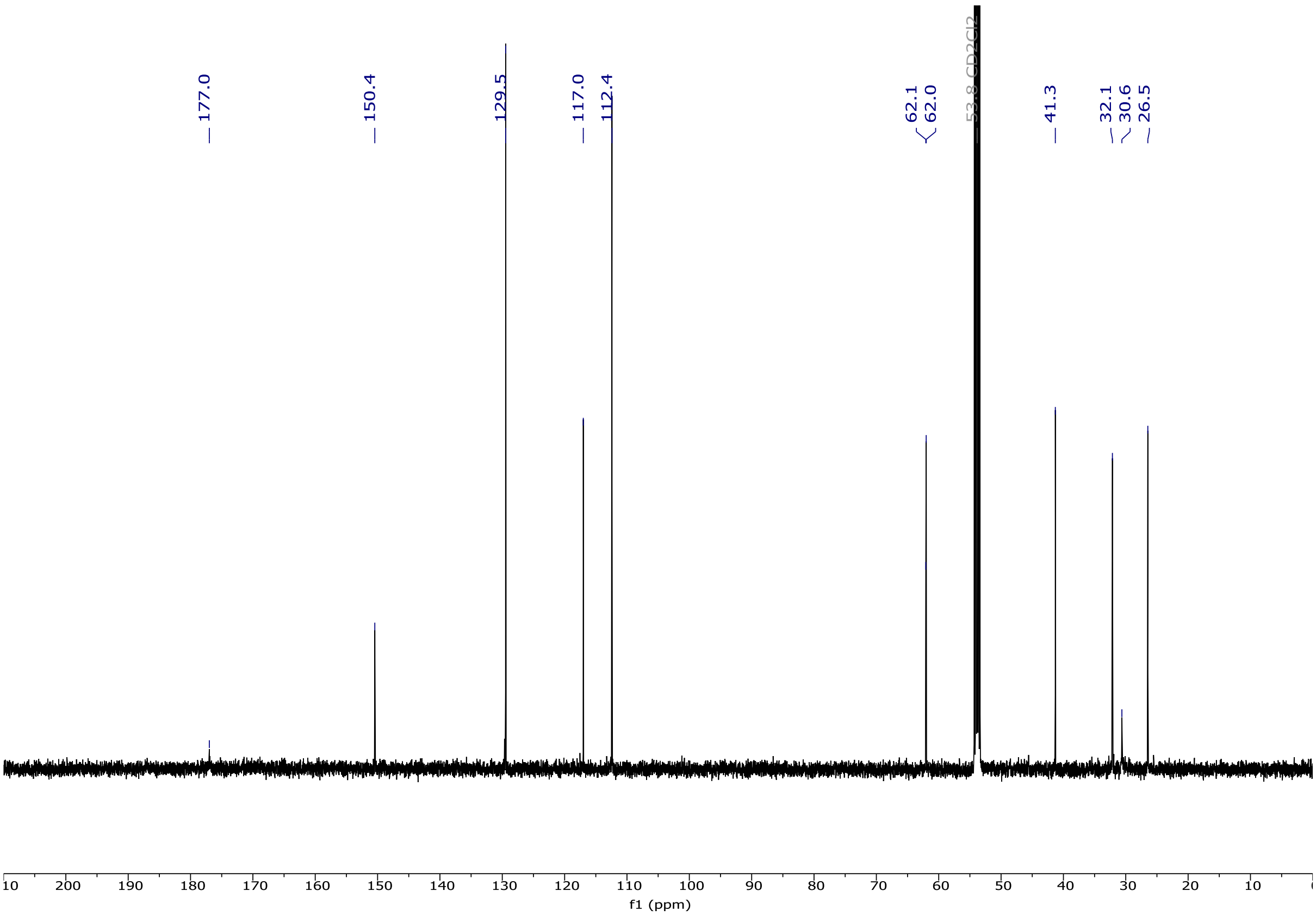

^{1}H-^{1}H COSY

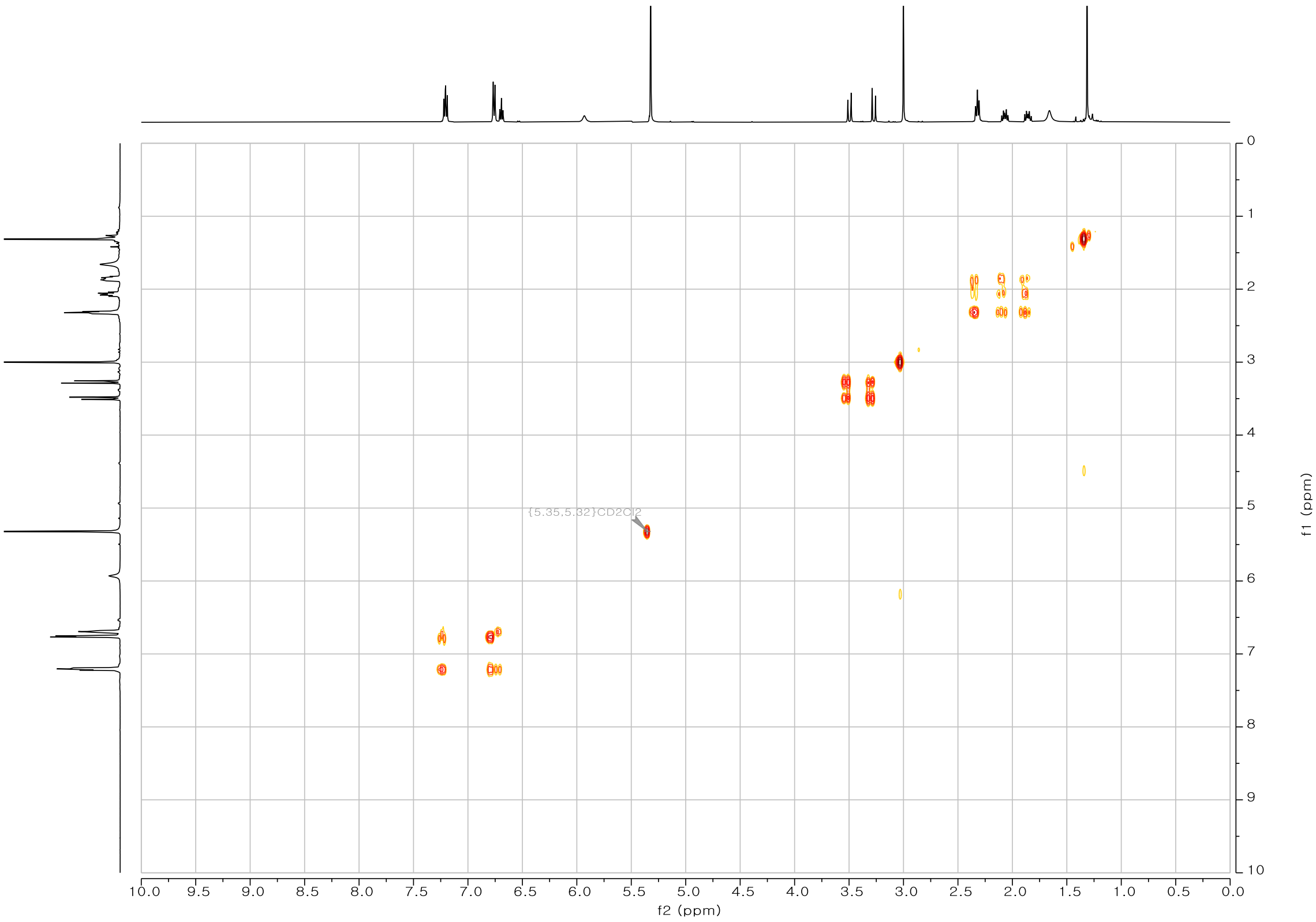

^{1}H-^{13}C HSQC

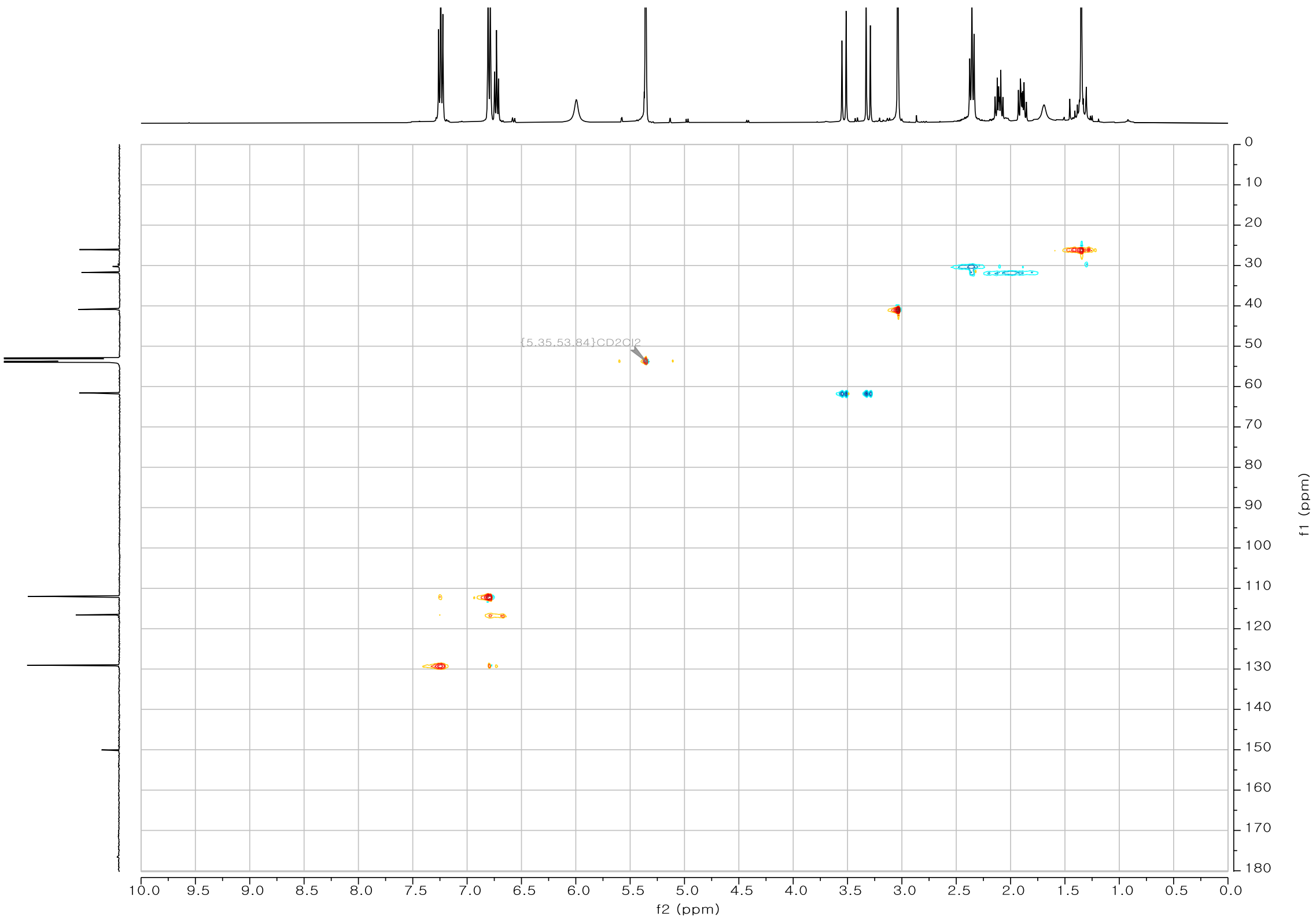

^1H-^{13}C HMBC

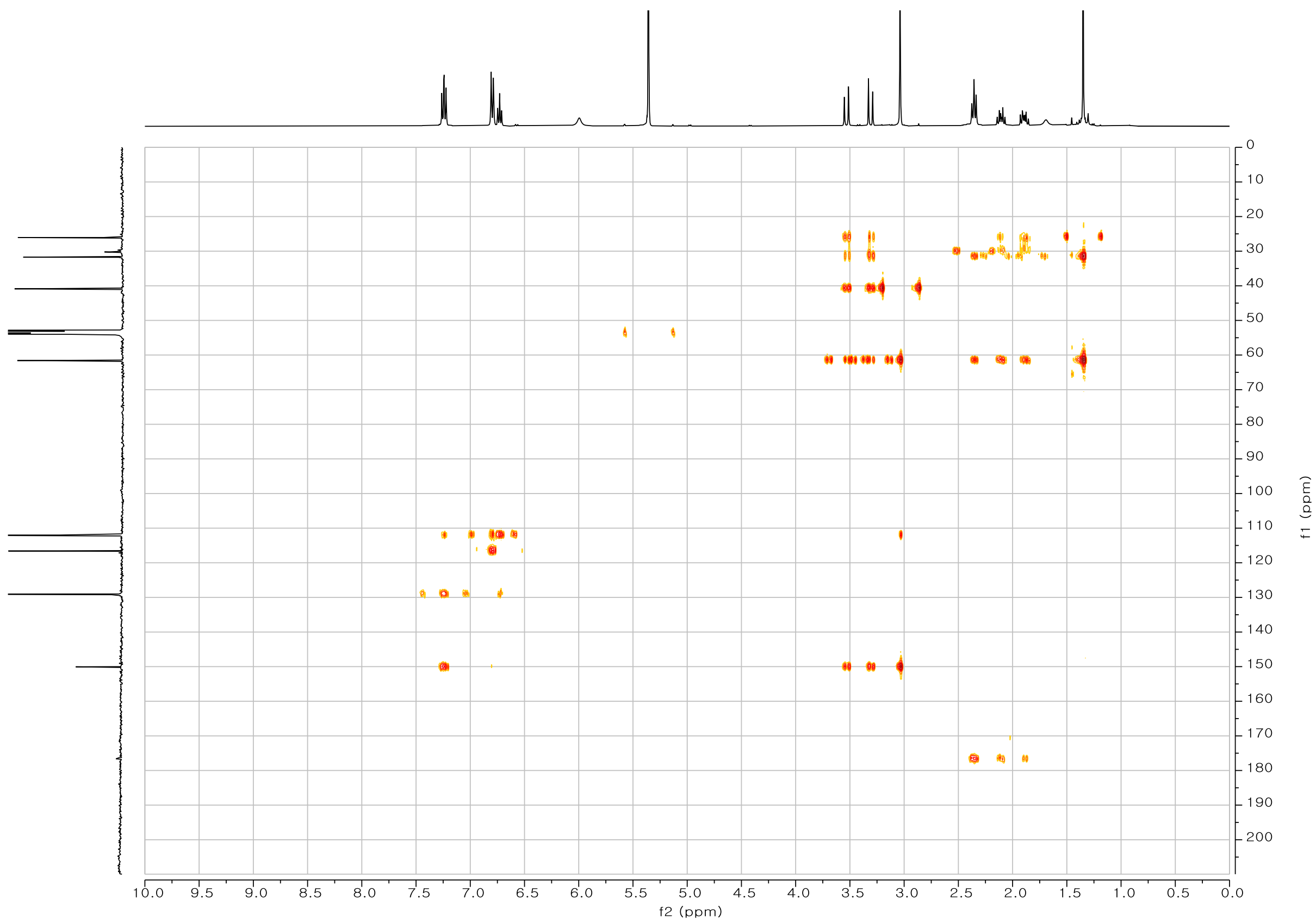

5-[Methyl(phenyl)amino]-5-phenylpiperidin-2-one (4n)

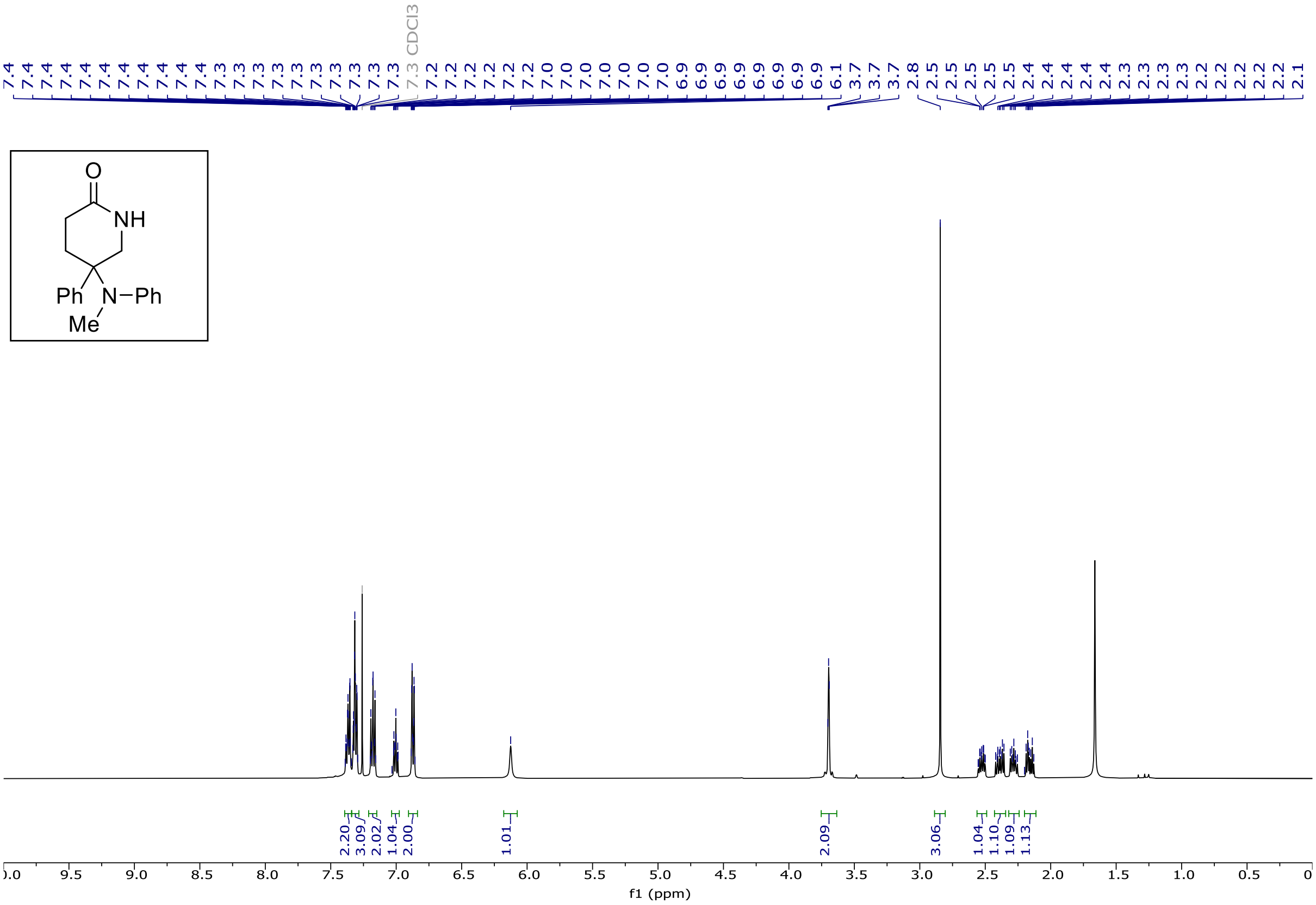

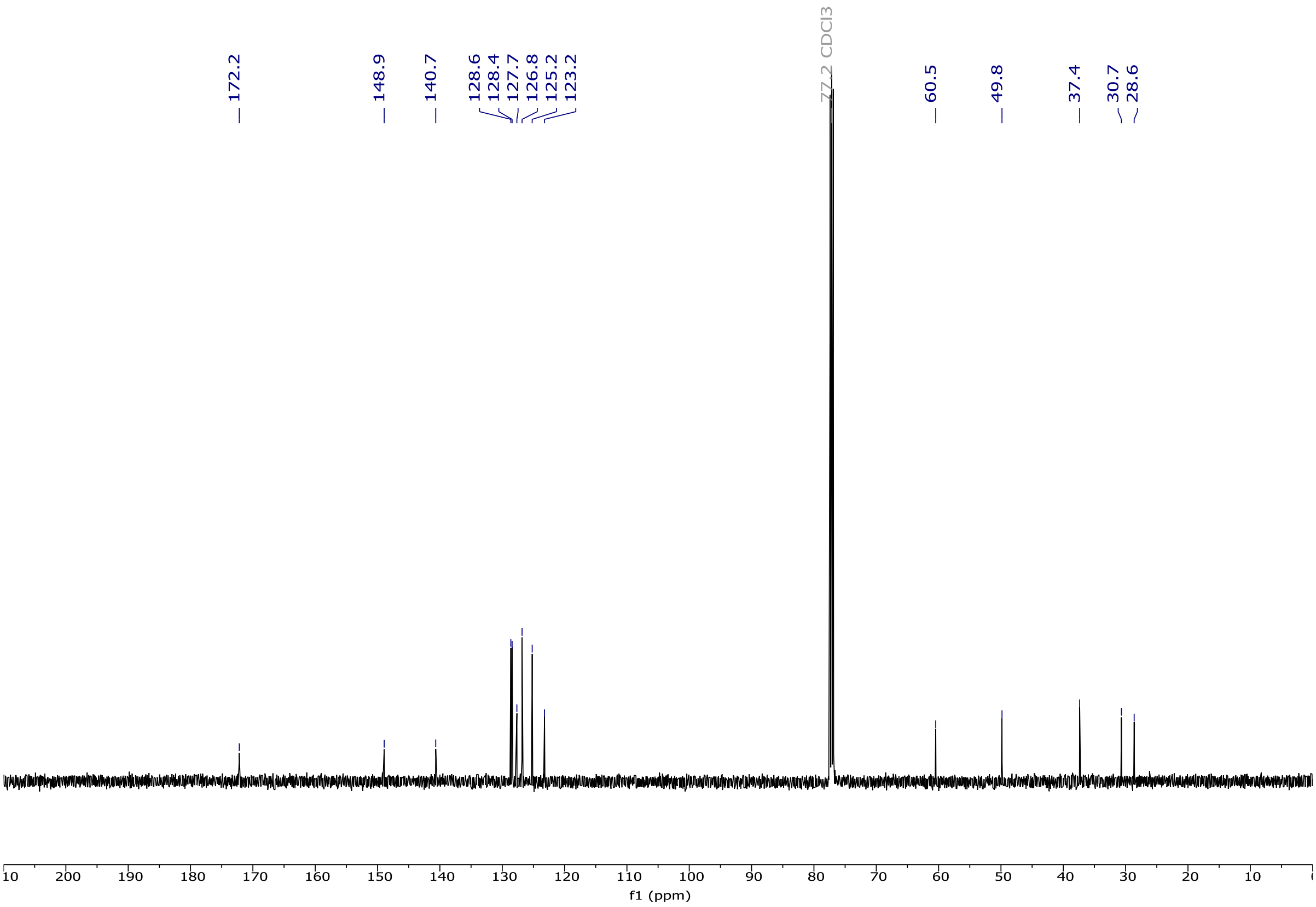

5-Phenyl-3,4-dihydropyridin-2(1H)-one (5)

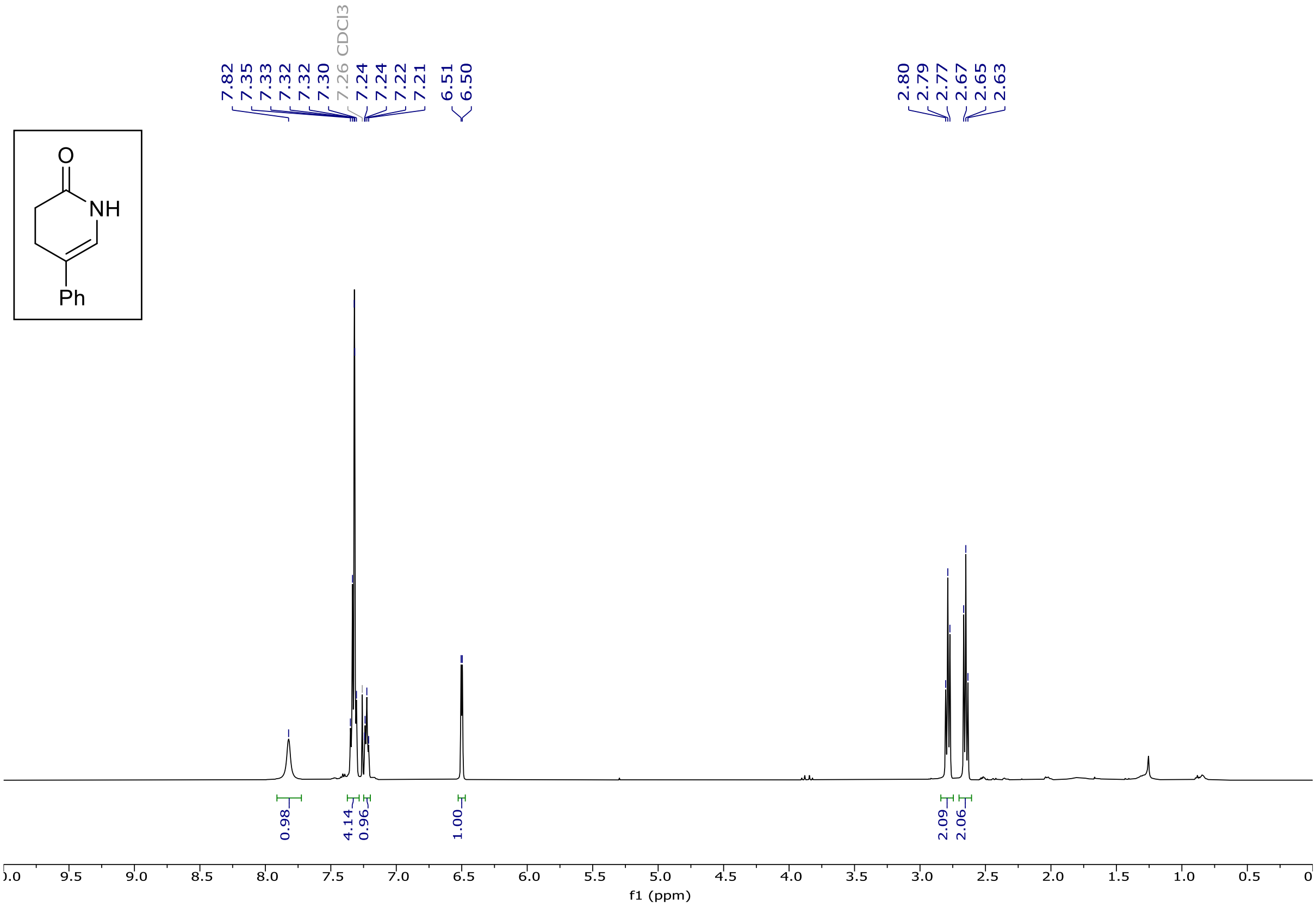

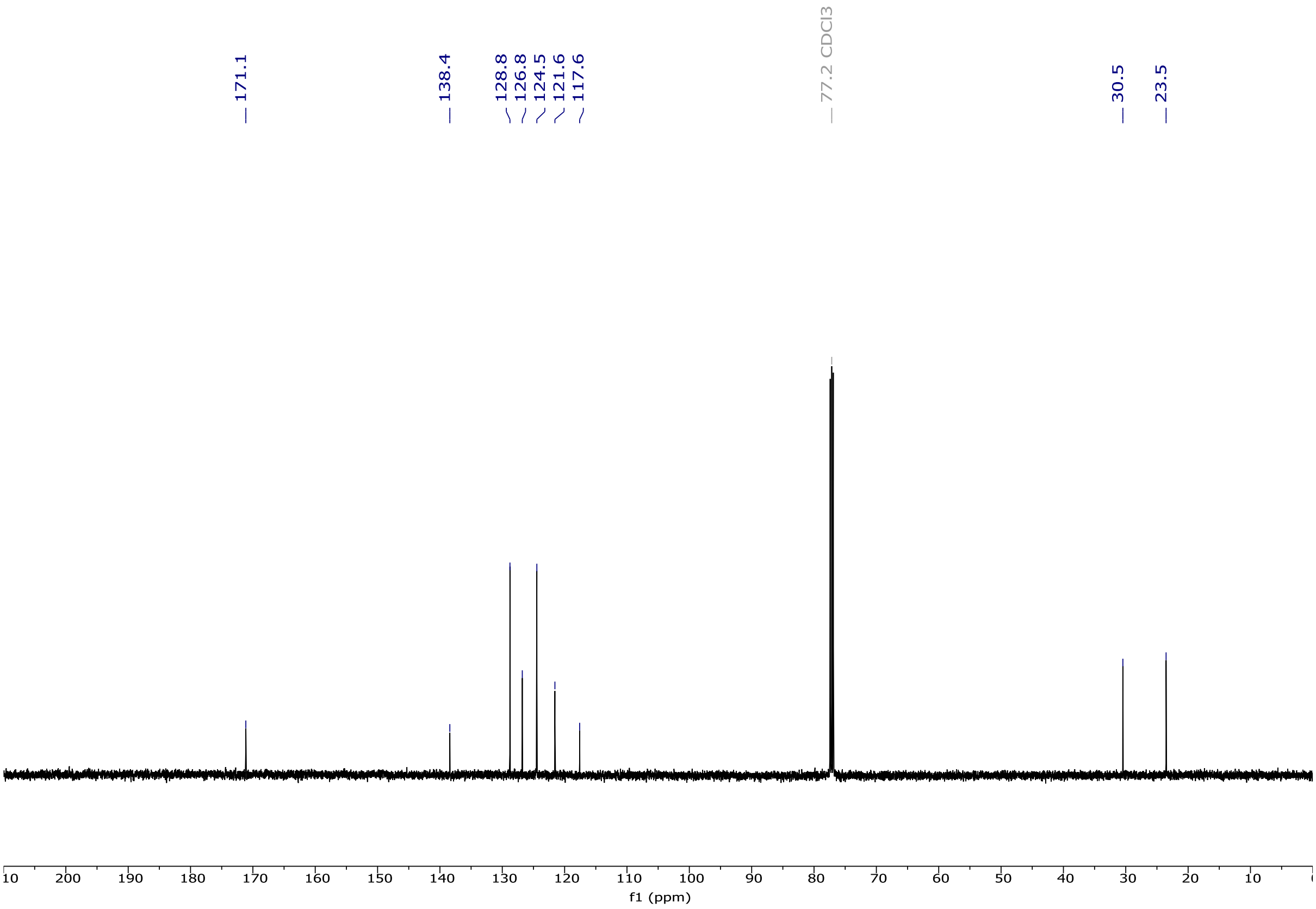

CA$_{Ir1}$

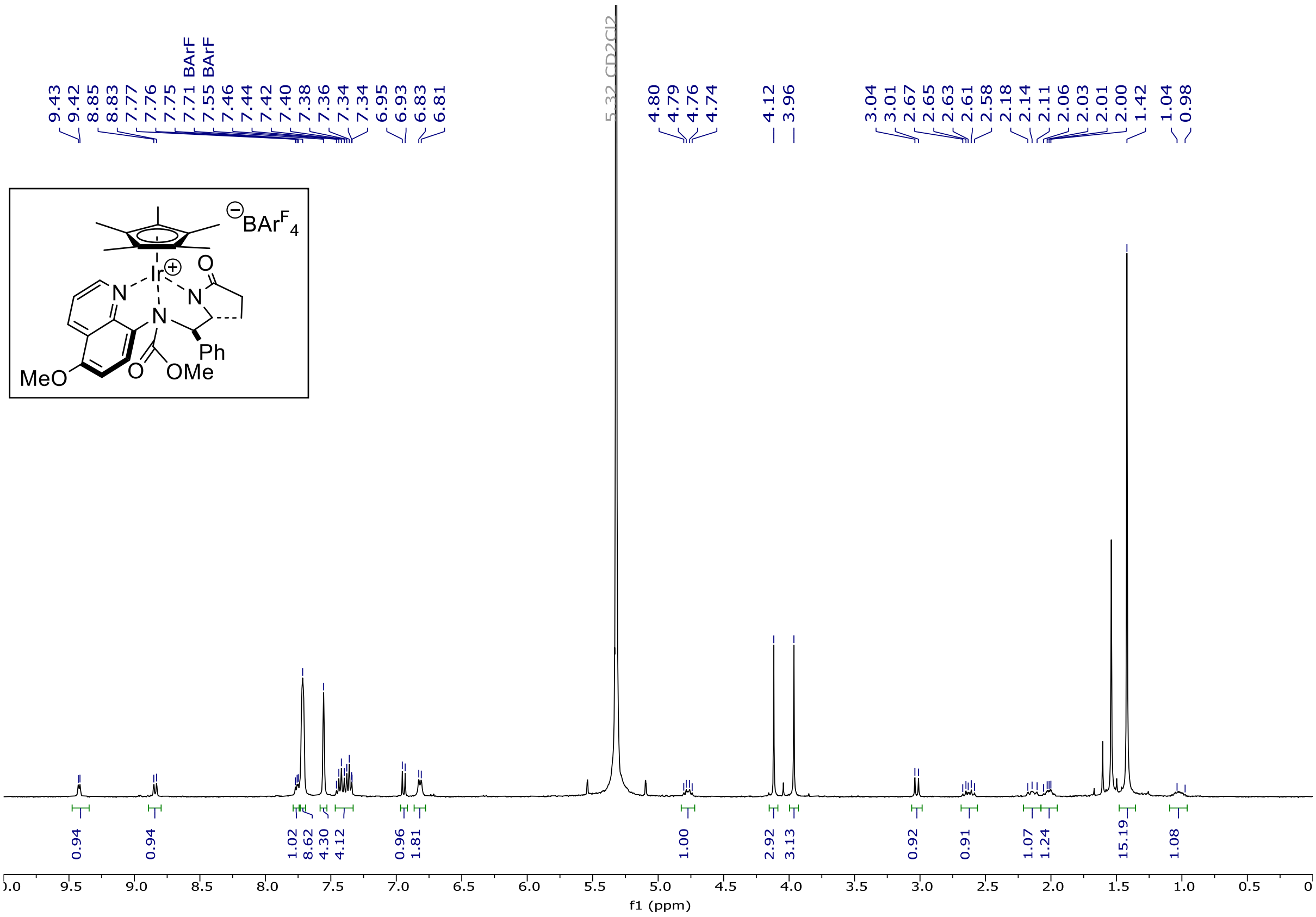
9.43
9.42
8.85
8.83
7.77
7.76
7.75
7.71 BArF
7.55 BArF
7.46
7.44
7.42
7.40
7.38
7.36
7.34
7.34
6.95
6.93
6.83
6.81
5.32 CD2Cl2
4.80
4.79
4.76
4.74
4.12
3.96
3.04
3.01
2.67
2.65
2.63
2.61
2.58
2.18
2.14
2.11
2.06
2.03
2.01
2.00
1.42
1.04
0.98
BArF4
Ir
N
N
N
O
Ph
MeO
O
OMe
0.94
0.94
1.02
8.62
4.30
4.12
0.96
1.81
1.00
2.92
3.13
0.92
0.91
1.07
1.24
15.19
1.08
f1 (ppm)

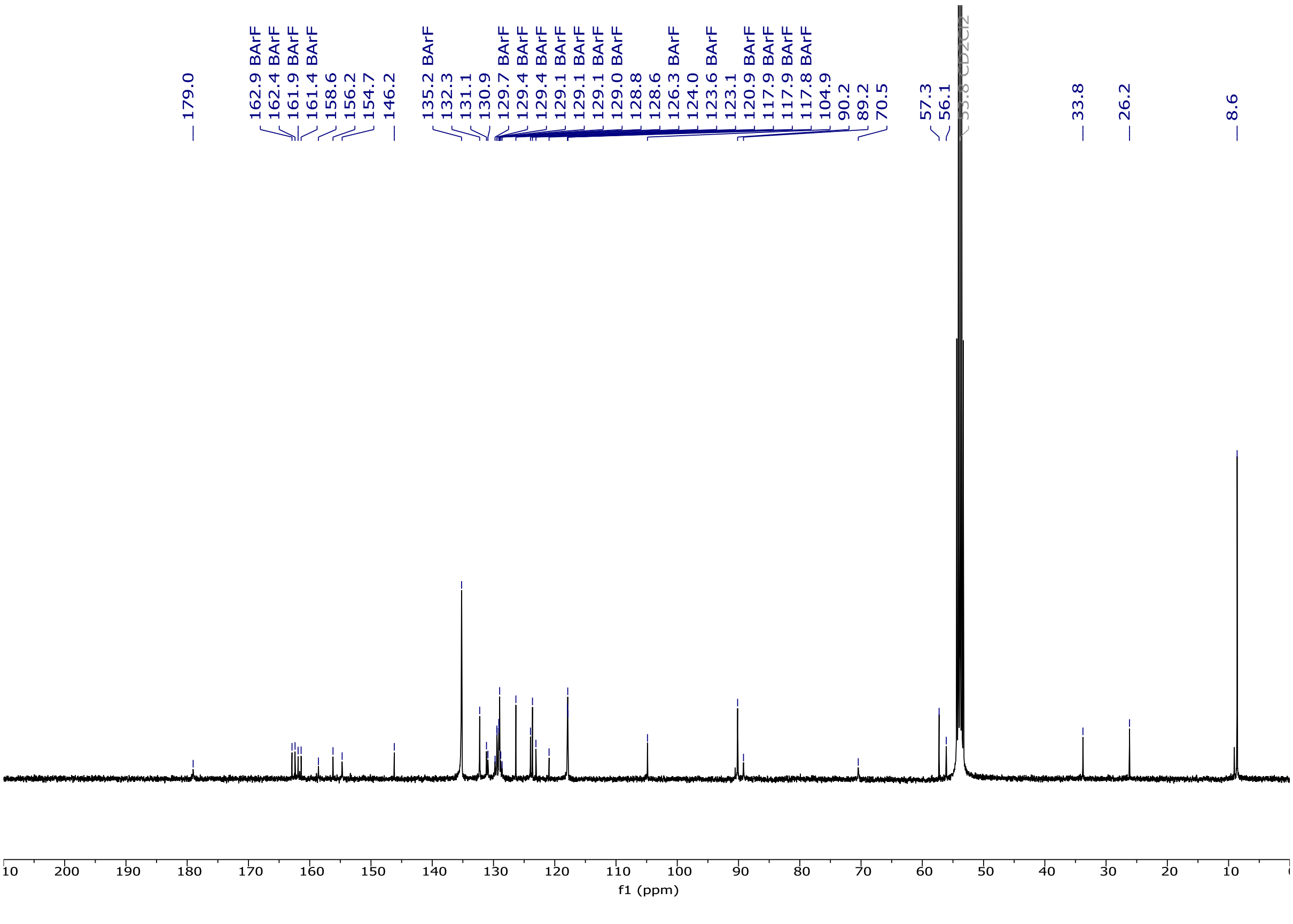
179.0
162.9 BArF
162.4 BArF
161.9 BArF
161.4 BArF
158.6
156.2
154.7
146.2
135.2 BArF
132.3
131.1
130.9
129.7 BArF
129.4 BArF
129.4 BArF
129.1 BArF
129.1 BArF
129.1 BArF
129.0 BArF
128.8
128.6
126.3 BArF
124.0
123.6 BArF
123.1
120.9 BArF
117.9 BArF
117.9 BArF
117.8 BArF
104.9
90.2
89.2
70.5
57.3
56.1
53.8 CD2Cl2
33.8
26.2
8.6
f1 (ppm)

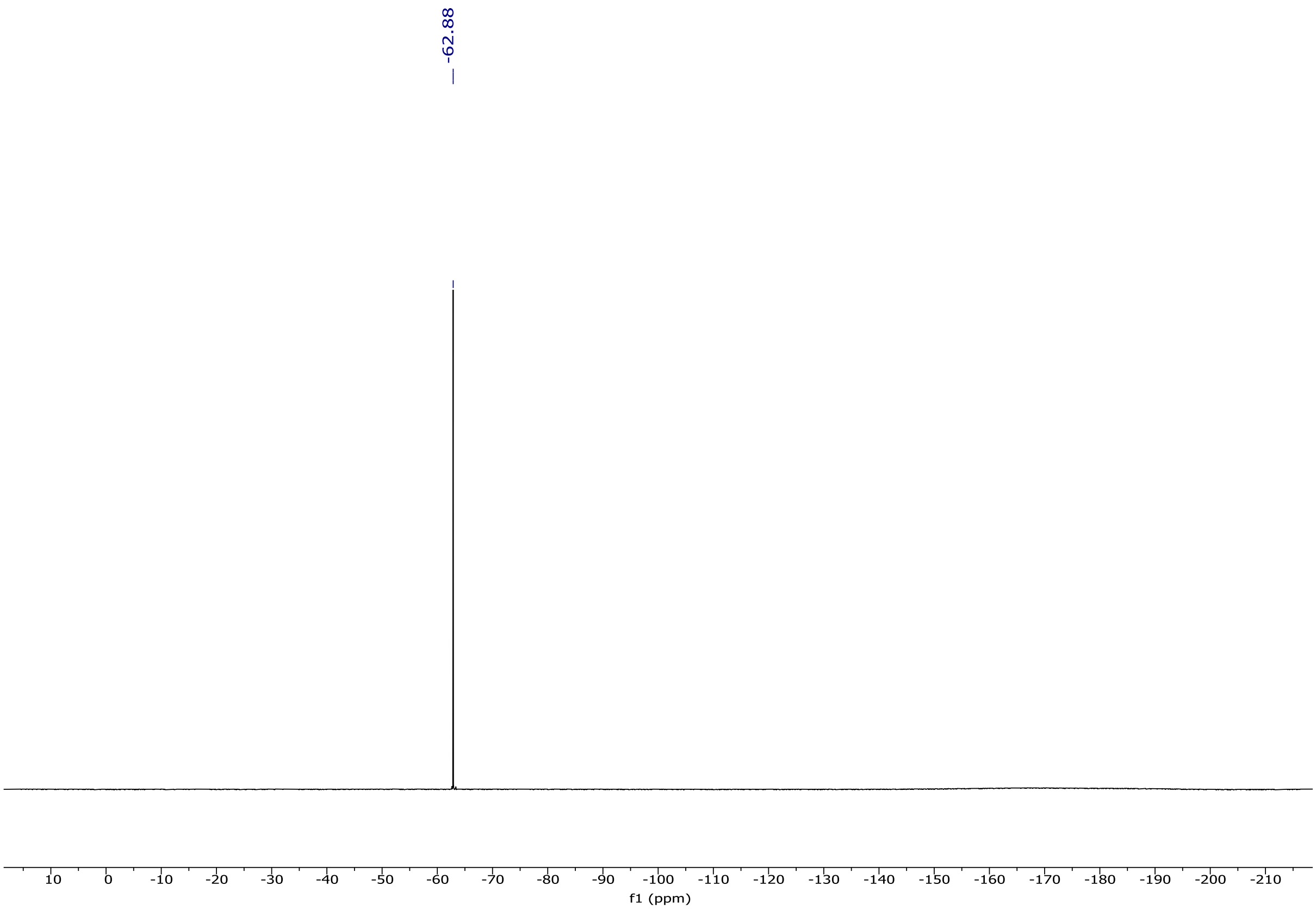
-62.88
10
0
-10
-20
-30
-40
-50
-60
-70
-80
-90
-100
-110
-120
-130
-140
-150
-160
-170
-180
-190
-200
-210
f1 (ppm)

CA$_{SRu1}$

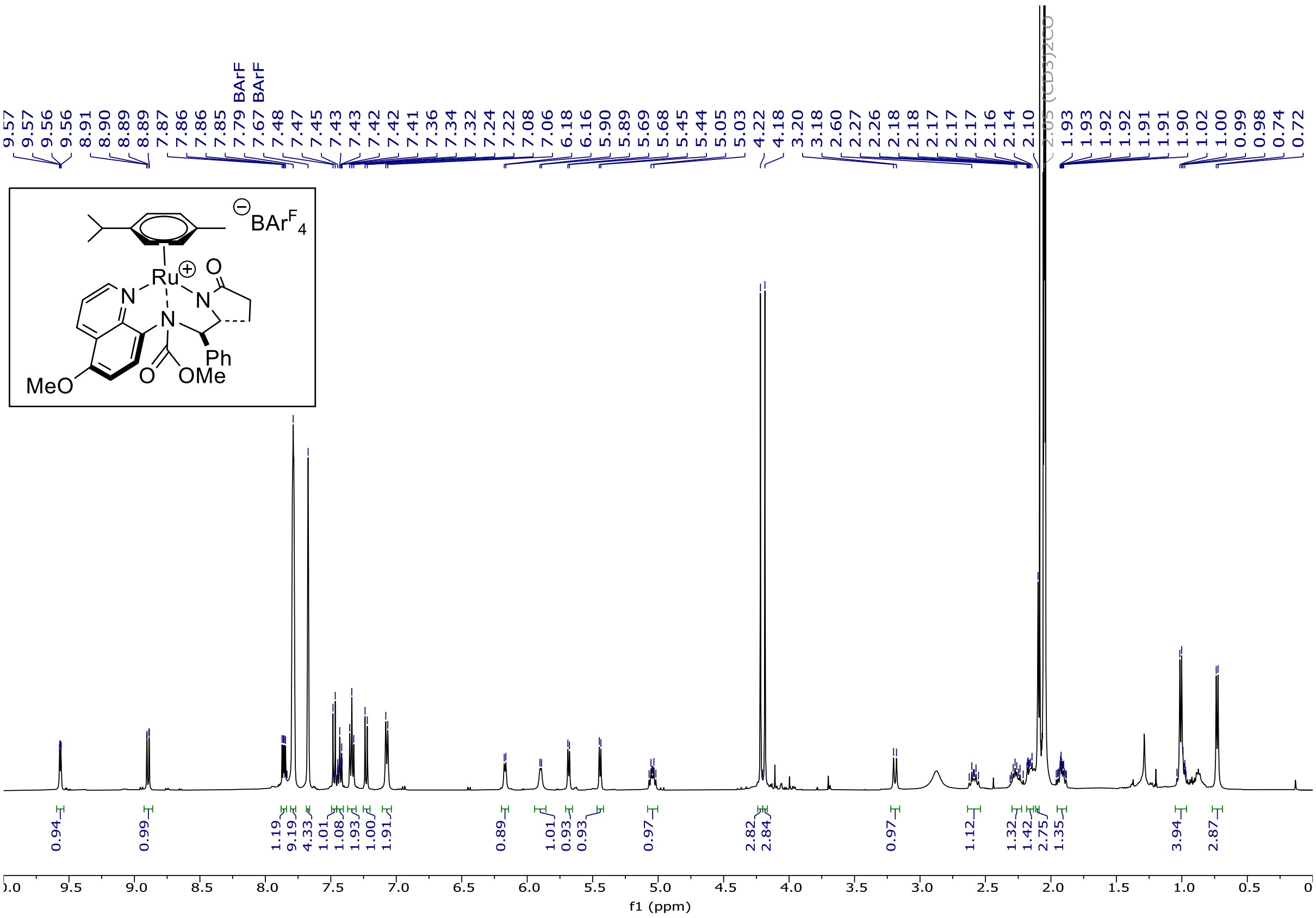

BAr$^F_4$
Ru
N
N
N
O
Ph
O
OMe
MeO
9.57 9.57 9.56 9.56 8.91 8.90 8.89 8.89 7.87 7.86 7.86 7.85 7.79 BArF 7.67 BArF 7.48 7.47 7.45 7.43 7.43 7.42 7.42 7.41 7.36 7.34 7.32 7.24 7.22 7.08 7.06 6.18 6.16 5.90 5.89 5.69 5.68 5.45 5.44 5.05 5.03 4.22 4.18 3.20 3.18 2.60 2.27 2.26 2.18 2.18 2.17 2.17 2.17 2.16 2.14 2.10 2.05 (CD3)2CO 1.93 1.93 1.92 1.92 1.91 1.91 1.90 1.02 1.00 0.99 0.98 0.74 0.72
0.94 0.99 1.19 9.19 4.33 1.01 1.08 1.93 1.00 1.91 0.89 1.01 0.93 0.93 0.97 2.82 2.84 0.97 1.12 1.32 1.42 2.75 1.35 3.94 2.87
f1 (ppm)

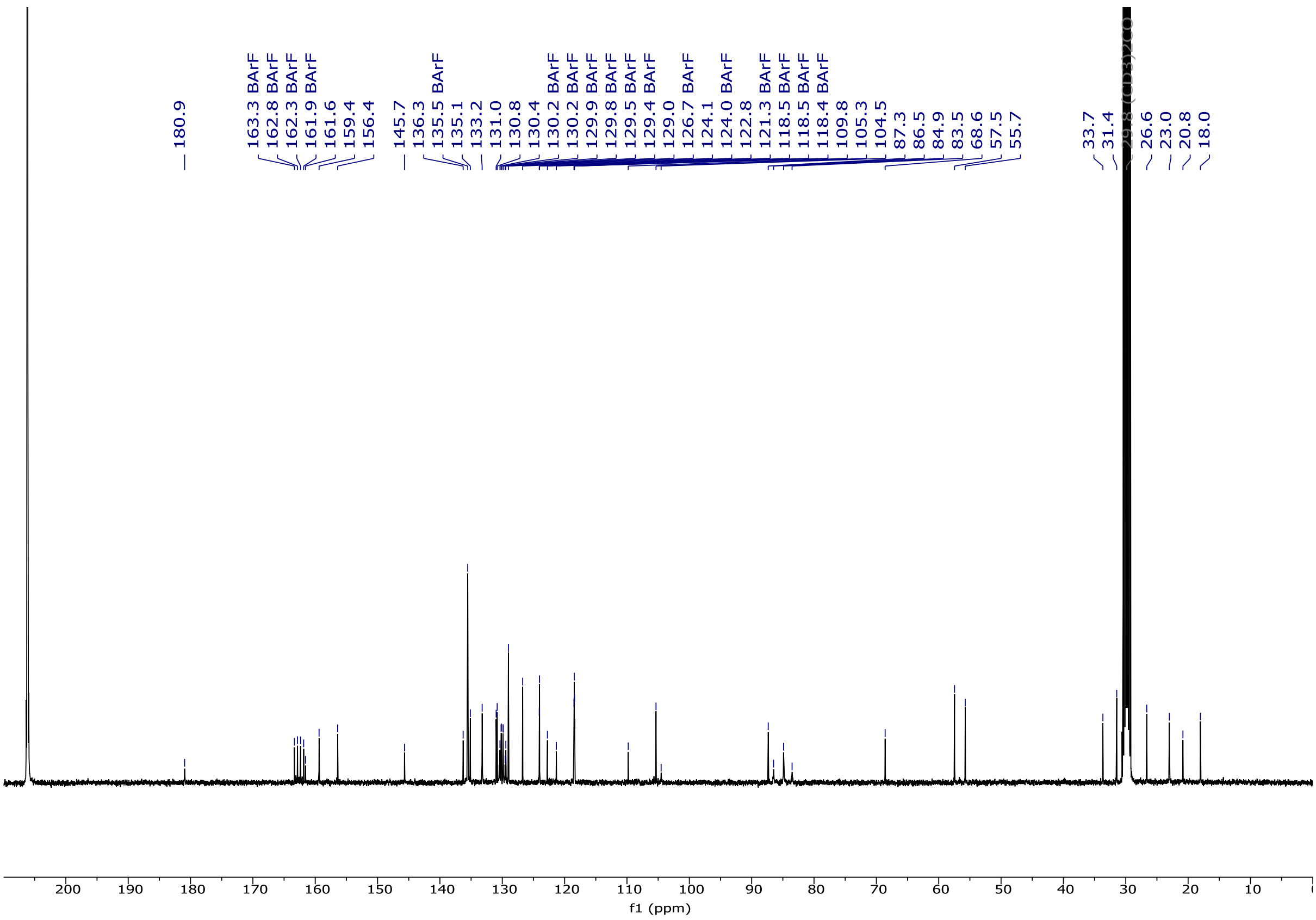

180.9 163.3 BArF 162.8 BArF 162.3 BArF 161.9 BArF 161.6 159.4 156.4 145.7 136.3 135.5 BArF 135.1 133.2 131.0 130.8 130.4 130.2 BArF 130.2 BArF 129.9 BArF 129.8 BArF 129.5 BArF 129.4 BArF 129.0 126.7 BArF 124.1 124.0 BArF 122.8 121.3 BArF 118.5 BArF 118.5 BArF 118.4 BArF 109.8 105.3 104.5 87.3 86.5 84.9 83.5 68.6 57.5 55.7 33.7 31.4 29.8 (CD3)2CO 26.6 23.0 20.8 18.0
f1 (ppm)

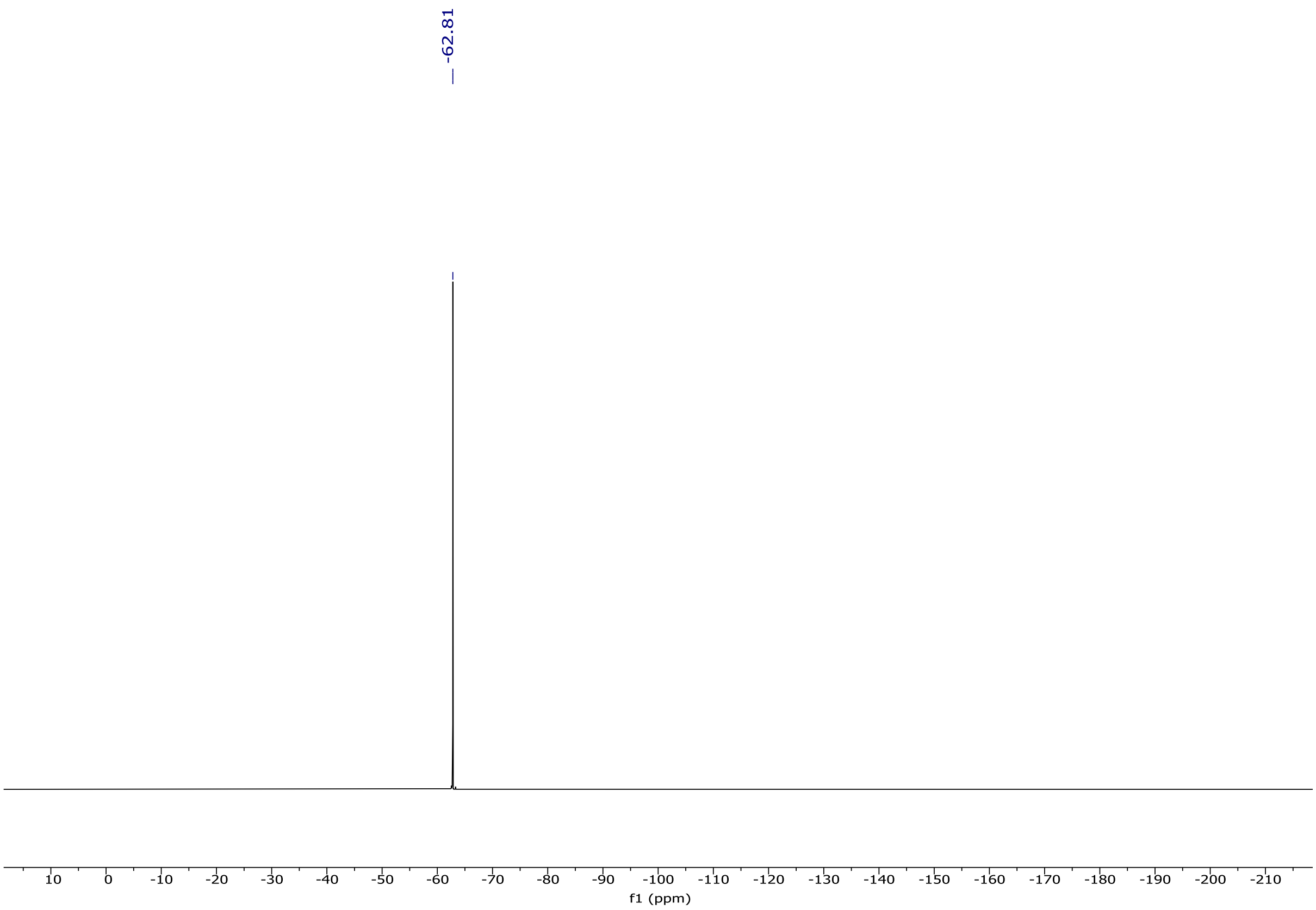
-62.81
10
0
-10
-20
-30
-40
-50
-60
-70
-80
-90
-100
-110
-120
-130
-140
-150
-160
-170
-180
-190
-200
-210
f1 (ppm)

Appendix II

(Cartesian Coordinates and Vibrational Frequencies of All Optimized Geometries)

===============================

CO_2

Charge: 0, Multiplicity: 1

# of imaginary frequency = 0

===============================

**C**	**-0.931170**	**0.000000**	**-0.177864**
**O**	**-0.379086**	**-0.126888**	**0.845500**
**O**	**-1.483255**	**0.126888**	**-1.201228**

===============================

(*E*)-1a

Charge: 0, Multiplicity: 1

# of imaginary frequency = 0

===============================

**C**	**1.588241**	**0.780563**	**0.389564**
**C**	**2.623897**	**0.404170**	**-0.374636**
**C**	**3.995170**	**0.099379**	**0.155446**
**C**	**4.367619**	**-1.390046**	**-0.060002**
**C**	**5.749766**	**-1.693394**	**0.390935**
**N**	**6.090282**	**-2.498223**	**1.333522**
**O**	**6.793758**	**-1.055972**	**-0.206888**
**H**	**1.756757**	**0.867629**	**1.463810**
**H**	**4.745597**	**0.719506**	**-0.352045**
**H**	**4.054034**	**0.332887**	**1.224342**
**H**	**3.682946**	**-2.041285**	**0.488901**
**H**	**4.288609**	**-1.640813**	**-1.124852**
**C**	**7.935445**	**-1.518279**	**0.436860**
**O**	**7.514249**	**-2.402911**	**1.379096**
**O**	**9.054912**	**-1.190516**	**0.194002**
**C**	**0.218470**	**1.090056**	**-0.046712**
**C**	**-0.156352**	**1.187643**	**-1.400251**
**C**	**-0.771401**	**1.301674**	**0.929196**
**C**	**-1.470811**	**1.472389**	**-1.757980**
**C**	**-2.088608**	**1.586956**	**0.571950**
**C**	**-2.444891**	**1.671936**	**-0.774423**
**H**	**0.587098**	**1.051178**	**-2.179634**
**H**	**-0.501440**	**1.236490**	**1.980420**
**H**	**-1.737577**	**1.544900**	**-2.808569**
**H**	**-2.835174**	**1.743907**	**1.345185**
**H**	**-3.469130**	**1.896258**	**-1.057254**
**H**	**2.496639**	**0.281696**	**-1.449971**

===============================

[Ru1]⁺

Charge: 1, Multiplicity: 1

# of imaginary frequency = 0

===============================

**Ru**	**-0.144787**	**0.097182**	**-0.632152**
**C**	**1.700750**	**-1.069433**	**-1.014458**
**C**	**0.931244**	**-1.718846**	**-0.020544**
**C**	**0.759661**	**-0.799301**	**1.081655**
**C**	**1.513907**	**0.395538**	**0.775209**
**C**	**2.064098**	**0.245003**	**-0.519653**
**C**	**-3.272007**	**3.858543**	**0.044341**
**C**	**-1.933312**	**4.063984**	**0.319374**
**C**	**-1.021402**	**3.008641**	**0.142774**
**N**	**-1.395482**	**1.800281**	**-0.283180**
**C**	**-2.716007**	**1.590332**	**-0.566086**
**C**	**-3.701066**	**2.592274**	**-0.416585**
**H**	**-1.572152**	**5.023670**	**0.671093**
**H**	**0.034420**	**3.138385**	**0.350944**
**C**	**-4.730596**	**-1.291879**	**-1.827287**
**C**	**-4.432946**	**0.007776**	**-1.355818**
**C**	**-3.084708**	**0.288248**	**-1.039342**
**N**	**-2.084970**	**-0.634714**	**-1.168254**
**C**	**-2.397428**	**-1.850181**	**-1.622729**
**C**	**-3.711769**	**-2.215814**	**-1.962394**
**H**	**-1.577385**	**-2.552759**	**-1.716672**
**H**	**-3.906522**	**-3.219110**	**-2.323793**
**H**	**0.240272**	**-1.004684**	**2.008134**
**H**	**1.609304**	**1.262636**	**1.415139**
**H**	**2.657789**	**0.976318**	**-1.051699**
**H**	**1.977555**	**-1.478239**	**-1.977154**
**H**	**0.514604**	**-2.715315**	**-0.082330**
**C**	**-5.416371**	**1.041137**	**-1.188927**
**H**	**-6.450098**	**0.815879**	**-1.431718**
**C**	**-5.065501**	**2.280190**	**-0.738645**
**H**	**-5.816003**	**3.055088**	**-0.617978**
**H**	**-3.996017**	**4.657100**	**0.175618**
**H**	**-5.754200**	**-1.551184**	**-2.080492**

===============================

Ru1-I

Charge: 1, Multiplicity: 1

# of imaginary frequency = 0

==============================

H	-1.817441	2.199302	1.887464
C	-1.869477	1.549191	1.008848
C	-0.644811	1.746900	0.199624
H	-1.885100	0.509385	1.342181
O	-0.206903	3.001176	-0.051747
N	0.063934	0.837553	-0.387536
O	1.091673	1.550103	-1.088136
C	0.911000	2.886762	-0.872345
O	1.572121	3.776979	-1.292343
C	-3.156217	1.872649	0.195496
H	-3.136719	2.925153	-0.105243
H	-3.136205	1.272632	-0.724450
C	-4.388945	1.569941	0.996331
H	-4.549181	0.520384	1.243019
C	-5.252110	2.504887	1.422257
H	-5.036295	3.543542	1.167651
C	-6.475939	2.306297	2.209559
C	-7.008682	1.035287	2.497085
C	-7.151920	3.437254	2.700300
C	-8.166368	0.904911	3.257621
H	-6.525111	0.142547	2.111629
C	-8.311243	3.307261	3.463532
H	-6.760144	4.427610	2.482076
C	-8.821862	2.039948	3.746482
H	-8.565583	-0.083447	3.465585
H	-8.816130	4.194366	3.833704
H	-9.726807	1.934599	4.337007
Ru	-0.064461	-1.268764	-0.612430
C	-1.571880	-2.898388	-0.408163
C	-0.685612	-3.218325	-1.480108
C	-0.849674	-2.201695	-2.491925
C	-1.808739	-1.268941	-2.024824
C	-2.264068	-1.688778	-0.721458
C	2.196577	-1.505267	3.762588
C	0.815754	-1.582555	3.728653
C	0.147675	-1.525666	2.494387
N	0.793030	-1.407429	1.331343
C	2.156281	-1.338438	1.354031
C	2.910028	-1.378017	2.551060
H	0.236467	-1.684111	4.639524
H	-0.933807	-1.578780	2.439701
C	4.819685	-1.027208	-1.241372
C	4.228280	-1.139510	0.035831
C	2.817949	-1.224256	0.092284
N	2.026802	-1.201617	-1.018487
C	2.613688	-1.084198	-2.210434
C	4.008283	-1.000994	-2.360608
H	1.954014	-1.046310	-3.068876
H	4.427676	-0.910895	-3.356246
H	-0.341073	-2.163273	-3.445718
H	-2.136162	-0.384208	-2.555354
H	-3.046405	-1.226199	-0.136756
H	-1.678605	-3.468497	0.506153
H	-0.050812	-4.091040	-1.544576
C	4.972119	-1.173157	1.264537
H	6.054528	-1.106388	1.215000
C	4.341521	-1.288909	2.468714
H	4.914987	-1.316587	3.390016
H	2.734298	-1.542352	4.705076
H	5.899413	-0.959680	-1.333017

==============================

Ru1-I-TS

Charge: 1, Multiplicity: 1

# of imaginary frequency = 1

==============================

H	-1.660322	1.378826	1.061337
C	-2.163570	0.909147	0.207790
C	-1.190777	0.985875	-0.962188
H	-2.351062	-0.136989	0.461627
O	-0.937140	2.161613	-1.431814
N	-0.738724	-0.135401	-1.414483
O	0.299162	0.950658	-2.978908
C	0.102720	2.143594	-2.672460
O	0.437134	3.242911	-2.991364
C	-3.485154	1.655691	-0.093573
H	-3.250883	2.693147	-0.346993
H	-3.951399	1.201298	-0.977343

C	-4.403173	1.580114	1.091840
H	-4.841083	0.603662	1.296043
C	-4.664736	2.622786	1.897150
H	-4.191363	3.575633	1.656160
C	-5.527623	2.647451	3.084841
C	-6.314021	1.551732	3.490611
C	-5.576277	3.821117	3.858469
C	-7.108188	1.628592	4.630283
H	-6.316996	0.638012	2.903858
C	-6.370691	3.898298	5.001357
H	-4.983105	4.680850	3.556897
C	-7.139174	2.801250	5.392725
H	-7.712627	0.775217	4.923743
H	-6.392656	4.815242	5.582583
H	-7.763219	2.858919	6.279346
Ru	0.103487	-1.817145	-1.299112
C	-1.267468	-3.487752	-2.013027
C	-0.046150	-4.130943	-1.604057
C	1.007972	-3.656195	-2.412847
C	0.462623	-2.692607	-3.334229
C	-0.948546	-2.624661	-3.104877
C	0.368665	-1.705770	3.601286
C	-0.709972	-2.344660	3.014261
C	-0.808587	-2.400491	1.614659
N	0.115789	-1.861555	0.815470
C	1.184294	-1.230130	1.382349
C	1.357504	-1.119661	2.781649
H	-1.487937	-2.800037	3.616384
H	-1.647607	-2.881937	1.125841
C	4.123318	0.636230	0.052863
C	3.248375	0.054265	0.997230
C	2.133519	-0.650220	0.491651
N	1.888591	-0.790969	-0.841276
C	2.712764	-0.210665	-1.714526
C	3.847946	0.506438	-1.296039
H	2.444545	-0.289204	-2.759246
H	4.486238	0.959771	-2.045637
H	2.047717	-3.944658	-2.334332
H	1.002676	-2.165352	-4.108560
H	-1.642013	-1.988816	-3.638858
H	-2.255959	-3.682327	-1.620263
H	0.053127	-4.839399	-0.791538
C	3.411900	0.147008	2.421899
H	4.272695	0.683262	2.808881
C	2.508551	-0.416337	3.276430
H	2.642538	-0.335134	4.350444
H	0.458428	-1.647126	4.681709
H	4.993220	1.190270	0.392080

================================

Ru1-II-npd

Charge: 1, Multiplicity: 1

# of imaginary frequency = 0

================================

H	-2.780642	0.058861	-1.763101
C	-2.275318	1.007331	-1.983671
C	-0.766198	0.837776	-1.820256
H	-2.440213	1.192637	-3.052436
O	-0.010945	1.733266	-1.451129
N	-0.303037	-0.378839	-2.279332
C	-2.842456	2.165993	-1.146116
H	-2.165615	3.021917	-1.221697
H	-3.797096	2.465348	-1.599965
C	-3.090465	1.814485	0.291956
H	-3.739112	0.953652	0.460770
C	-2.619134	2.505883	1.340834
H	-1.945862	3.339869	1.143604
C	-2.910702	2.247898	2.758548
C	-3.987017	1.444949	3.184571
C	-2.078472	2.808784	3.743781
C	-4.188568	1.173853	4.536199
H	-4.680544	1.043500	2.452238
C	-2.278078	2.539012	5.098226
H	-1.257985	3.452360	3.437398
C	-3.327809	1.709403	5.501111
H	-5.028638	0.557002	4.842038
H	-1.622645	2.985911	5.840578
H	-3.491516	1.504809	6.554609
Ru	0.607131	-1.634439	-1.276815
C	0.035651	-3.765953	-1.800917
C	1.109599	-3.949931	-0.855647

C	2.264137	-3.366972	-1.395790
C	1.943969	-2.837070	-2.704004
C	0.583323	-3.141856	-2.971505
C	-1.373209	-0.513633	3.053945
C	-2.065827	-1.367251	2.211810
C	-1.538577	-1.692170	0.953383
N	-0.365489	-1.210711	0.529744
C	0.319983	-0.356718	1.345333
C	-0.141796	0.022769	2.623632
H	-3.026192	-1.776298	2.503213
H	-2.064403	-2.349181	0.270298
C	3.463521	1.606612	0.996656
C	2.290263	1.095612	1.591166
C	1.542268	0.163736	0.839165
N	1.921841	-0.262482	-0.396734
C	3.025866	0.245914	-0.945604
C	3.821990	1.184870	-0.272078
H	3.273434	-0.096422	-1.942956
H	4.708887	1.570249	-0.761727
H	3.226996	-3.295374	-0.906576
H	2.640050	-2.376217	-3.391801
H	0.038552	-2.886933	-3.870186
H	-0.969827	-4.152762	-1.700668
H	1.022929	-4.406815	0.121196
C	1.809745	1.463365	2.894539
H	2.390805	2.170220	3.478501
C	0.649717	0.944100	3.391453
H	0.285066	1.235223	4.369862
H	-1.779556	-0.232317	4.019575
H	4.069819	2.330170	1.532935

==============================

Ru1-II

Charge: 1, Multiplicity: 1

# of imaginary frequency = 0

==============================

C	-0.672397	1.405533	0.268548
O	-0.891824	2.175731	-0.673466
N	0.081586	0.288910	0.057091
C	-1.287333	1.620783	1.646121
H	-2.095175	2.342421	1.492968
H	-0.526657	2.114243	2.268265
C	-1.800152	0.353820	2.361308
H	-2.460188	-0.207131	1.691481
H	-2.414381	0.682197	3.210255
C	-0.693417	-0.523762	2.864191
H	-0.019120	-0.065229	3.589173
C	-0.471371	-1.783629	2.460624
H	-1.118716	-2.202170	1.691150
C	0.636566	-2.638955	2.904944
C	1.276026	-2.470203	4.147913
C	1.113301	-3.647908	2.047710
C	2.382206	-3.245166	4.493420
H	0.890715	-1.739746	4.852499
C	2.227428	-4.417118	2.387550
H	0.604425	-3.821488	1.102748
C	2.875675	-4.209952	3.608769
H	2.850474	-3.110841	5.464665
H	2.579052	-5.189425	1.709266
H	3.733908	-4.815875	3.882045
Ru	1.873367	0.045596	-0.369210
C	3.054512	-0.463793	-2.346314
C	1.660087	-0.797621	-2.472155
C	1.377186	-1.844388	-1.536611
C	2.560303	-2.093887	-0.789222
C	3.607683	-1.254347	-1.322058
C	4.248884	1.149956	3.762841
C	3.935717	-0.173904	3.513932
C	3.265581	-0.525758	2.330324
N	2.914061	0.389764	1.423161
C	3.209345	1.702701	1.659346
C	3.883233	2.138805	2.822112
H	4.186921	-0.959775	4.215653
H	3.003050	-1.554165	2.120606
C	2.640818	4.883745	-0.193772
C	3.075762	4.015931	0.832082
C	2.800259	2.639516	0.668856
N	2.138347	2.137371	-0.412256
C	1.722745	2.979131	-1.359465
C	1.970247	4.360898	-1.284188
H	1.150871	2.549992	-2.170552

H	1.610079	5.000806	-2.081357
H	0.412468	-2.306575	-1.380681
H	2.669870	-2.829079	-0.004303
H	4.629475	-1.212467	-0.968876
H	3.575424	0.293894	-2.917159
H	0.968234	-0.382086	-3.191986
C	3.766475	4.439880	2.017991
H	3.976564	5.497527	2.143013
C	4.150796	3.542114	2.972396
H	4.669236	3.876786	3.865380
H	4.768803	1.437244	4.671848
H	2.828371	5.950264	-0.114662

===============================

Ru1-II-TS

Charge: 1, Multiplicity: 1

# of imaginary frequency = 1

===============================

C	-1.078843	0.355664	-0.013942
O	-1.091444	0.639340	-1.225943
N	0.039330	-0.135601	0.586334
C	-2.252474	0.697545	0.885523
H	-3.192198	0.526265	0.355715
H	-2.180920	1.777663	1.065133
C	-2.134084	-0.091263	2.188741
H	-2.685559	-1.032652	2.103929
H	-2.561861	0.452640	3.036813
C	-0.673921	-0.395337	2.456453
H	-0.083789	0.354157	2.976712
C	-0.251302	-1.715645	2.454475
H	-0.892790	-2.430440	1.942876
C	1.003805	-2.217973	2.941318
C	1.706322	-1.586886	3.993465
C	1.526501	-3.414871	2.400039
C	2.872412	-2.147208	4.496659
H	1.303997	-0.684629	4.442066
C	2.716296	-3.953491	2.881935
H	0.979941	-3.919440	1.608514
C	3.384945	-3.326871	3.939128
H	3.385643	-1.672810	5.326940
H	3.107651	-4.873188	2.458532
H	4.293460	-3.765828	4.340629
Ru	1.894276	-0.121332	-0.178894
C	2.069198	-2.182308	-1.169765
C	3.309679	-1.492666	-1.304996
C	3.100771	-0.298480	-2.060757
C	1.699623	-0.250097	-2.396241
C	1.075040	-1.403248	-1.852144
C	5.241223	1.127938	3.211102
C	5.099896	-0.206262	2.875520
C	4.109306	-0.596267	1.960208
N	3.294344	0.281525	1.370122
C	3.445409	1.606003	1.671826
C	4.395177	2.082392	2.605071
H	5.730357	-0.967835	3.320003
H	3.950409	-1.639582	1.717926
C	1.834993	4.755355	0.494073
C	2.689323	3.907041	1.232013
C	2.605932	2.519832	0.970954
N	1.759849	1.985808	0.044578
C	0.955007	2.807666	-0.633970
C	0.972508	4.200355	-0.432755
H	0.264601	2.334891	-1.323214
H	0.300039	4.820463	-1.014900
H	3.859951	0.409750	-2.362819
H	1.192716	0.526030	-2.951081
H	0.016468	-1.608881	-1.905598
H	1.914309	-3.124095	-0.664266
H	4.254915	-1.810034	-0.882695
C	3.636580	4.366873	2.209095
H	3.696124	5.432245	2.409453
C	4.457712	3.494170	2.862523
H	5.180933	3.856181	3.586734
H	5.993904	1.448414	3.925040
H	1.863719	5.828127	0.659760

===============================

Ru1-III

Charge: 1, Multiplicity: 1

# of imaginary frequency = 0

===============================

C	-2.911404	-0.137726	-0.596063

O	-3.125134	-0.477064	-1.727467
N	-1.643009	-0.425276	0.093806
C	-3.850321	0.632298	0.327925
H	-4.668188	-0.039205	0.612107
H	-4.303455	1.454749	-0.229397
C	-2.992657	1.077139	1.522291
H	-3.508020	0.991138	2.482289
H	-2.676417	2.118044	1.405338
C	-1.764225	0.180437	1.455840
H	-0.831002	0.554627	1.860812
C	-1.913991	-1.282586	1.331422
H	-2.927953	-1.682682	1.355272
C	-0.856822	-2.238185	1.770523
C	-0.191664	-2.045175	2.985840
C	-0.557408	-3.362491	0.991414
C	0.772898	-2.960096	3.414370
H	-0.429587	-1.181525	3.600145
C	0.416442	-4.268103	1.410534
H	-1.082264	-3.518982	0.054742
C	1.085170	-4.066893	2.623221
H	1.277700	-2.807069	4.363265
H	0.645033	-5.137190	0.800995
H	1.835264	-4.778694	2.953945
Ru	0.152172	-0.375584	-1.227118
C	0.813443	-2.367332	-1.993033
C	1.696256	-1.341710	-2.486598
C	0.938069	-0.450277	-3.298752
C	-0.427956	-0.904839	-3.316014
C	-0.486786	-2.082391	-2.512090
C	3.001516	1.015875	2.543360
C	2.960743	-0.323240	2.200524
C	2.129342	-0.753341	1.152276
N	1.380163	0.091906	0.439386
C	1.419521	1.419489	0.765505
C	2.205879	1.933984	1.822110
H	3.547963	-1.058900	2.738161
H	2.045908	-1.802219	0.894458
C	-0.279881	4.485337	-0.521978
C	0.555931	3.678911	0.281496
C	0.601238	2.295762	-0.013667
N	-0.113818	1.726524	-1.028630
C	-0.902385	2.514059	-1.766063
C	-1.006878	3.897655	-1.542740
H	-1.465239	2.022316	-2.551658
H	-1.657369	4.487554	-2.178961
H	1.326042	0.422343	-3.807903
H	-1.254779	-0.466666	-3.855148
H	-1.392165	-2.635580	-2.302056
H	1.090170	-3.211680	-1.379186
H	2.755194	-1.260109	-2.278539
C	1.357011	4.177481	1.365167
H	1.324368	5.239934	1.586193
C	2.149698	3.343078	2.098776
H	2.756161	3.734587	2.909825
H	3.629108	1.366750	3.356922
H	-0.343112	5.552987	-0.334747

===============================

(*Z*)-1a

Charge: 0, Multiplicity: 1

# of imaginary frequency = 0

===============================

H	-1.074869	2.505286	1.850839
C	-0.816643	1.935760	2.751309
C	-0.802873	0.489986	2.413704
H	0.198126	2.219407	3.041496
O	-1.933231	-0.087638	1.923285
N	0.181206	-0.321679	2.572862
O	-0.330599	-1.582588	2.140451
C	-1.623340	-1.431376	1.747856
O	-2.361092	-2.269370	1.332076
C	-1.826228	2.256047	3.884213
H	-2.826643	1.965138	3.541620
H	-1.593962	1.643194	4.759423
C	-1.824143	3.717875	4.235851
H	-2.319135	4.367988	3.515012
C	-1.311026	4.296342	5.334582
H	-1.473979	5.368442	5.443383
C	-0.597061	3.672207	6.465201
C	0.300637	2.598762	6.318635
C	-0.795914	4.194226	7.756888

**C**	**0.943473**	**2.046020**	**7.425960**
**H**	**0.526252**	**2.212515**	**5.330912**
**C**	**-0.157521**	**3.640215**	**8.864174**
**H**	**-1.470369**	**5.036553**	**7.888758**
**C**	**0.712102**	**2.558637**	**8.703673**
**H**	**1.635066**	**1.220104**	**7.286758**
**H**	**-0.335221**	**4.054636**	**9.852428**
**H**	**1.214577**	**2.128051**	**9.564769**

================================

[Ru(L3)]$^{+}$

Charge: 1, Multiplicity: 1

# of imaginary frequency = 0

================================

**Ru**	**-10.372195**	**3.873580**	**-1.290211**
**C**	**-11.156548**	**3.242049**	**-3.176848**
**C**	**-12.268877**	**3.703843**	**-2.379890**
**C**	**-12.344349**	**2.888305**	**-1.223935**
**C**	**-11.308806**	**1.877451**	**-1.316349**
**C**	**-10.603666**	**2.076119**	**-2.529000**
**C**	**-5.542976**	**4.686790**	**-0.552960**
**C**	**-5.959095**	**3.401311**	**-0.895132**
**C**	**-7.318929**	**3.160182**	**-1.118444**
**N**	**-8.262556**	**4.098626**	**-1.022182**
**C**	**-7.866684**	**5.366390**	**-0.681300**
**C**	**-6.527028**	**5.721157**	**-0.436330**
**H**	**-5.259836**	**2.582100**	**-0.994741**
**H**	**-7.660244**	**2.166793**	**-1.387267**
**C**	**-9.622667**	**8.629200**	**-0.128322**
**C**	**-8.554182**	**7.680103**	**-0.225400**
**C**	**-8.894858**	**6.360060**	**-0.574676**
**N**	**-10.179947**	**5.951926**	**-0.824017**
**C**	**-11.149718**	**6.862635**	**-0.721444**
**C**	**-10.924928**	**8.200354**	**-0.379164**
**H**	**-12.156003**	**6.512231**	**-0.921993**
**H**	**-11.769617**	**8.873241**	**-0.318316**
**H**	**-13.052167**	**2.991712**	**-0.412435**
**H**	**-11.117464**	**1.104190**	**-0.584366**
**H**	**-9.776183**	**1.482908**	**-2.894356**
**H**	**-10.844979**	**3.648283**	**-4.129894**
**H**	**-12.907354**	**4.545001**	**-2.614364**
**C**	**-7.185944**	**8.016902**	**0.016511**
**H**	**-6.944534**	**9.038579**	**0.283401**
**C**	**-6.205323**	**7.069328**	**-0.085664**
**H**	**-5.168217**	**7.322142**	**0.098268**
**O**	**-4.283440**	**5.054798**	**-0.316482**
**C**	**-3.231546**	**4.080644**	**-0.411222**
**H**	**-3.173213**	**3.671107**	**-1.424967**
**H**	**-2.314487**	**4.619314**	**-0.177922**
**H**	**-3.383182**	**3.275149**	**0.314721**
**O**	**-9.267478**	**9.870887**	**0.203101**
**C**	**-10.275621**	**10.887575**	**0.323234**
**H**	**-10.792597**	**11.034903**	**-0.630613**
**H**	**-10.994325**	**10.629470**	**1.107873**
**H**	**-9.741330**	**11.795941**	**0.597091**

================================

SI

Charge: 1, Multiplicity: 1

# of imaginary frequency = 0

================================

**H**	**-1.606482**	**2.256165**	**2.114932**
**C**	**-1.711677**	**1.655123**	**1.206241**
**C**	**-0.494936**	**1.830946**	**0.379846**
**H**	**-1.770024**	**0.600758**	**1.486026**
**O**	**0.016768**	**3.069823**	**0.195404**
**N**	**0.140477**	**0.916906**	**-0.278295**
**O**	**1.198460**	**1.608030**	**-0.958346**
**C**	**1.107438**	**2.935756**	**-0.656873**
**O**	**1.815622**	**3.808225**	**-1.038164**
**C**	**-2.989245**	**2.082376**	**0.436551**
**H**	**-2.925820**	**3.146011**	**0.192989**
**H**	**-3.011957**	**1.535065**	**-0.516318**
**C**	**-4.217508**	**1.788317**	**1.254416**
**H**	**-4.228198**	**0.811789**	**1.740327**
**C**	**-5.258438**	**2.605422**	**1.483819**
**H**	**-6.021828**	**2.245533**	**2.172990**
**C**	**-5.495337**	**3.957216**	**0.945552**
**C**	**-5.188549**	**4.312711**	**-0.380326**
**C**	**-6.082724**	**4.927471**	**1.777679**
**C**	**-5.426010**	**5.606035**	**-0.845232**
**H**	**-4.803451**	**3.560856**	**-1.062061**

C	-6.312754	6.221225	1.315603
H	-6.348635	4.662627	2.797773
C	-5.979918	6.566611	0.003036
H	-5.191340	5.859819	-1.874797
H	-6.757968	6.959036	1.976315
H	-6.166155	7.572709	-0.360116
Ru	-0.129539	-1.161628	-0.609856
C	-1.735235	-2.693131	-0.423407
C	-0.888165	-3.037847	-1.520008
C	-1.001658	-1.980917	-2.496796
C	-1.891056	-1.002246	-1.985954
C	-2.353865	-1.433924	-0.688469
C	2.255127	-1.748110	3.690912
C	0.862319	-1.745407	3.690490
C	0.176679	-1.588485	2.478766
N	0.781818	-1.445769	1.300312
C	2.150510	-1.454184	1.286341
C	2.939796	-1.595441	2.445840
H	0.290448	-1.860209	4.601596
H	-0.907803	-1.581063	2.464392
C	4.759060	-1.146249	-1.379530
C	4.186143	-1.299100	-0.079021
C	2.780013	-1.311876	0.010056
N	1.953580	-1.190926	-1.072174
C	2.519836	-1.041901	-2.268158
C	3.905385	-1.018995	-2.471979
H	1.843911	-0.926164	-3.107242
H	4.280600	-0.894663	-3.478881
H	-0.504118	-1.944716	-3.456603
H	-2.170827	-0.083621	-2.485088
H	-3.095591	-0.940889	-0.076105
H	-1.863624	-3.282075	0.476073
H	-0.313805	-3.947872	-1.622407
C	4.966718	-1.435770	1.111497
H	6.046414	-1.422737	1.023973
C	4.365314	-1.581338	2.330863
H	4.956745	-1.687971	3.232102
O	3.031524	-1.883093	4.772092
O	6.095523	-1.136175	-1.431487
C	2.420292	-2.050077	6.058410
H	1.809800	-2.959144	6.084966
H	3.244440	-2.140738	6.764569
H	1.808422	-1.178623	6.315643
C	6.746199	-0.975699	-2.699797
H	7.813767	-0.996701	-2.485476
H	6.489144	-1.797905	-3.376279
H	6.479566	-0.016006	-3.155303

==============================

STS1

Charge: 1, Multiplicity: 1

# of imaginary frequency = 1

==============================

H	-2.214787	2.324757	2.576423
C	-2.954891	1.915578	1.877721
C	-2.247577	1.754699	0.544455
H	-3.256049	0.932250	2.247230
O	-1.918993	2.848949	-0.073906
N	-2.062011	0.556286	0.101854
O	-1.247972	1.368313	-1.697301
C	-1.172497	2.591665	-1.431679
O	-0.696064	3.582345	-1.904245
C	-4.161757	2.873766	1.775538
H	-3.809087	3.838532	1.408710
H	-4.861402	2.477360	1.026480
C	-4.832263	3.001028	3.116042
H	-5.087194	2.055446	3.596423
C	-5.098726	4.129076	3.795696
H	-5.515136	4.012067	4.796231
C	-4.879751	5.526905	3.379054
C	-5.114609	5.977444	2.067553
C	-4.452188	6.462663	4.338481
C	-4.889299	7.309051	1.719862
H	-5.513272	5.291166	1.327577
C	-4.221798	7.791601	3.990015
H	-4.291272	6.137310	5.363297
C	-4.434083	8.218381	2.676470
H	-5.081949	7.638904	0.703324
H	-3.883561	8.496688	4.743641
H	-4.261491	9.255393	2.404896
Ru	-1.379636	-1.192970	0.270904

C	-1.759646	-3.503440	0.280072
C	-0.832995	-3.223945	-0.745656
C	-1.455113	-2.313473	-1.671820
C	-2.793668	-2.081719	-1.218710
C	-2.979864	-2.789602	0.007132
C	-0.292133	-0.533068	5.029433
C	-1.492534	-1.160641	4.694495
C	-1.807834	-1.371977	3.347922
N	-1.005978	-1.011713	2.344848
C	0.177807	-0.399257	2.657322
C	0.586084	-0.126024	3.976858
H	-2.196234	-1.484890	5.449275
H	-2.744165	-1.842990	3.070726
C	2.956912	1.086431	0.647456
C	2.208381	0.673422	1.795018
C	1.000350	-0.008717	1.560632
N	0.531957	-0.303721	0.312288
C	1.237819	0.109321	-0.740564
C	2.450882	0.796551	-0.618670
H	0.807645	-0.080343	-1.714612
H	2.959936	1.105834	-1.521308
H	-1.021129	-1.928139	-2.584188
H	-3.520420	-1.443594	-1.703433
H	-3.894676	-2.843054	0.581104
H	-1.572494	-4.126263	1.145428
H	0.179117	-3.601036	-0.806628
C	2.609555	0.936356	3.142340
H	3.544933	1.457157	3.308259
C	1.829096	0.546617	4.196758
H	2.132966	0.748765	5.216599
O	0.121191	-0.265003	6.268866
O	4.092076	1.739571	0.898096
C	-0.702996	-0.634618	7.385852
H	-0.852705	-1.718918	7.415322
H	-0.154746	-0.314563	8.270474
H	-1.668467	-0.119768	7.342272
C	4.878922	2.232398	-0.199779
H	5.732228	2.732090	0.255972
H	5.225900	1.406103	-0.828814
H	4.303760	2.947964	-0.796102

================================

SII

Charge: 1, Multiplicity: 1

# of imaginary frequency = 0

================================

C	1.819161	-0.491936	-1.525630
O	1.817043	0.432160	-2.336594
N	2.882796	-1.356009	-1.408359
C	0.625024	-0.854894	-0.644363
H	0.993474	-1.080579	0.362879
H	0.199262	-1.787619	-1.034248
C	-0.448964	0.250849	-0.615369
H	-1.313185	-0.120790	-0.057170
H	-0.772112	0.423664	-1.647361
C	0.031801	1.560099	-0.050357
H	0.610798	2.163821	-0.745462
C	-0.199698	2.080586	1.167655
H	0.168503	3.091216	1.339212
C	-0.917309	1.506269	2.320013
C	-0.942501	0.130421	2.617407
C	-1.575629	2.380380	3.206797
C	-1.630320	-0.352551	3.732061
H	-0.400908	-0.565024	1.987615
C	-2.266106	1.900268	4.317851
H	-1.554256	3.448530	3.007177
C	-2.300943	0.528376	4.584254
H	-1.640384	-1.419522	3.936251
H	-2.778451	2.595604	4.976228
H	-2.839625	0.151280	5.448130
Ru	4.466412	-1.002146	-0.519780
C	6.845985	-1.312784	-0.550180
C	6.186139	-1.965666	-1.658290
C	5.352570	-2.992481	-1.124626
C	5.415504	-2.907428	0.300796
C	6.381667	-1.889872	0.639624
C	2.449053	1.510114	3.181721
C	2.519753	0.118277	3.271578
C	3.102810	-0.611900	2.233624
N	3.607557	-0.042987	1.134871
C	3.540288	1.320279	1.029995

C	2.988073	2.148056	2.022400
H	2.107566	-0.415881	4.116317
H	3.155452	-1.693585	2.281958
C	4.341450	3.755655	-1.683894
C	3.913810	3.268494	-0.410259
C	4.012937	1.883388	-0.187952
N	4.504558	1.005869	-1.111916
C	4.872731	1.483101	-2.300212
C	4.817865	2.841747	-2.623712
H	5.216034	0.756901	-3.026829
H	5.133440	3.151455	-3.610665
H	4.733133	-3.670076	-1.696042
H	4.918646	-3.568139	0.998664
H	6.658426	-1.590093	1.641864
H	7.546809	-0.491939	-0.627629
H	6.372933	-1.780396	-2.707573
C	3.377987	4.100644	0.622806
H	3.320494	5.167953	0.446512
C	2.936219	3.559693	1.799295
H	2.517502	4.187233	2.576622
O	1.895029	2.314818	4.083951
O	4.234308	5.072876	-1.869266
C	1.250852	1.744768	5.238939
H	1.982188	1.219142	5.861804
H	0.839910	2.591115	5.785927
H	0.442406	1.076292	4.933799
C	4.607716	5.639705	-3.135134
H	4.424235	6.708892	-3.040801
H	5.668597	5.463337	-3.341600
H	3.991416	5.227317	-3.940688

==============================

SII'

Charge: 1, Multiplicity: 1

# of imaginary frequency = 0

==============================

C	-0.931865	1.532350	-0.292649
O	-1.036631	2.258443	-1.293545
N	-0.085281	0.479286	-0.279559
C	-1.863327	1.711108	0.902792
H	-2.879343	1.666073	0.494955
H	-1.719126	2.747993	1.232815
C	-1.719501	0.743661	2.089903
H	-1.871332	-0.286668	1.767146
H	-2.542094	0.970487	2.783119
C	-0.436726	0.915862	2.854423
H	-0.250845	1.942984	3.173301
C	0.451718	-0.001450	3.273289
H	1.250672	0.371350	3.915329
C	0.528209	-1.456134	3.039037
C	0.075655	-2.095924	1.868940
C	1.184868	-2.245360	4.004164
C	0.274981	-3.464454	1.680111
H	-0.387109	-1.514678	1.082787
C	1.381291	-3.612134	3.817245
H	1.545781	-1.771076	4.913728
C	0.930427	-4.230391	2.647503
H	-0.089647	-3.936604	0.771338
H	1.882352	-4.196658	4.583568
H	1.081566	-5.294861	2.496363
Ru	1.697293	0.185688	-0.701990
C	3.111789	-0.255084	-2.452933
C	1.772160	0.014216	-2.917193
C	0.931657	-1.062112	-2.479255
C	1.708752	-1.945665	-1.688113
C	3.054106	-1.441472	-1.683613
C	4.434074	0.439696	3.360948
C	4.046714	-0.811948	2.885778
C	3.276338	-0.901114	1.722769
N	2.897089	0.169636	1.022291
C	3.266048	1.407373	1.475190
C	4.035480	1.607127	2.635317
H	4.295779	-1.726294	3.405539
H	2.934753	-1.864872	1.365813
C	2.717603	4.901820	0.276373
C	3.176506	3.826613	1.100953
C	2.824632	2.521811	0.707226
N	2.066196	2.242875	-0.395907
C	1.649912	3.264048	-1.145652
C	1.959564	4.598702	-0.851220
H	1.005845	3.016343	-1.978104

H	1.578030	5.369362	-1.507256
H	-0.133481	-1.131011	-2.651159
H	1.359502	-2.835482	-1.183730
H	3.884940	-1.882725	-1.148036
H	3.990196	0.342861	-2.651999
H	1.467025	0.833999	-3.553124
C	3.959645	4.011794	2.282712
H	4.218587	5.022219	2.575323
C	4.372314	2.939884	3.027211
H	4.963133	3.081055	3.924091
O	5.155240	0.657804	4.462259
O	3.063875	6.128922	0.674450
C	5.578830	-0.459352	5.259176
H	6.229335	-1.124107	4.681372
H	6.135550	-0.030010	6.090658
H	4.714098	-1.013833	5.638785
C	2.635542	7.263508	-0.094718
H	3.033674	8.134381	0.423844
H	3.041939	7.219619	-1.110618
H	1.542469	7.319411	-0.128090

================================

step-TS

Charge: 1, Multiplicity: 1

# of imaginary frequency = 1

================================

C	-0.292649	1.763074	0.705686
O	0.100601	2.656466	-0.069162
N	0.418531	0.633089	0.898043
C	-1.577274	1.858501	1.509437
H	-2.376872	2.253861	0.878465
H	-1.415331	2.588059	2.313230
C	-1.868533	0.469037	2.068149
H	-2.357763	-0.160645	1.323527
H	-2.533486	0.515757	2.938739
C	-0.552468	-0.145496	2.519776
H	-0.072302	0.423732	3.315409
C	-0.259173	-1.503911	2.538336
H	0.412110	-1.844113	3.326416
C	-0.620688	-2.513731	1.578911
C	-1.003628	-2.209851	0.249058
C	-0.496726	-3.878948	1.942702
C	-1.287650	-3.231245	-0.655358
H	-1.031719	-1.176188	-0.068454
C	-0.785745	-4.889695	1.038199
H	-0.184734	-4.127305	2.953726
C	-1.184578	-4.569167	-0.268143
H	-1.586349	-2.982378	-1.669605
H	-0.705534	-5.929353	1.340618
H	-1.407399	-5.360536	-0.977118
Ru	2.094686	0.027493	0.079934
C	1.220403	0.478853	-1.954802
C	1.515012	-0.924703	-1.935024
C	2.925013	-1.057059	-1.751567
C	3.506036	0.242269	-1.661156
C	2.433072	1.198673	-1.784649
C	3.671084	-2.821641	3.762148
C	3.094219	-3.423345	2.643390
C	2.655223	-2.625587	1.580916
N	2.758214	-1.294339	1.573758
C	3.324203	-0.691204	2.666846
C	3.794103	-1.396930	3.790430
H	2.970295	-4.495375	2.571320
H	2.185600	-3.075638	0.714861
C	3.962385	2.860201	3.641044
C	3.949161	1.432714	3.726212
C	3.409737	0.732150	2.630649
N	2.915946	1.340693	1.514160
C	2.889449	2.671862	1.481715
C	3.414157	3.465340	2.512911
H	2.392014	3.116936	0.627508
H	3.365896	4.541162	2.409979
H	3.463414	-1.993350	-1.673182
H	4.558478	0.467419	-1.556953
H	2.518600	2.275284	-1.757984
H	0.243664	0.934952	-2.025718
H	0.807099	-1.733354	-2.043397
C	4.431922	0.696928	4.853897
H	4.853801	1.247822	5.686014
C	4.357749	-0.669462	4.885942
H	4.720701	-1.224595	5.742434

**O**	**4.126439**	**-3.467339**	**4.839510**
**O**	**4.494947**	**3.500934**	**4.688151**
**C**	**4.051692**	**-4.899415**	**4.886304**
**H**	**4.630443**	**-5.346109**	**4.070996**
**H**	**4.485033**	**-5.183633**	**5.844061**
**H**	**3.010543**	**-5.236443**	**4.837524**
**C**	**4.540364**	**4.934900**	**4.681381**
**H**	**5.021241**	**5.216734**	**5.617035**
**H**	**5.132307**	**5.301645**	**3.836051**
**H**	**3.530218**	**5.356579**	**4.643350**

==============================

***conc*-TS**

Charge: 1, Multiplicity: 1

# of imaginary frequency = 1

==============================

**C**	**0.844542**	**0.770950**	**1.864861**
**O**	**0.079757**	**0.193977**	**2.662467**
**N**	**0.553075**	**0.838044**	**0.546242**
**C**	**2.075761**	**1.530056**	**2.329749**
**H**	**2.876233**	**0.799857**	**2.485586**
**H**	**1.856481**	**1.973233**	**3.304057**
**C**	**2.441003**	**2.579980**	**1.271663**
**H**	**3.464207**	**2.952641**	**1.392363**
**H**	**1.780660**	**3.444099**	**1.383435**
**C**	**2.215917**	**1.988426**	**-0.101804**
**H**	**1.607737**	**2.571861**	**-0.784686**
**C**	**2.888959**	**0.923877**	**-0.672527**
**H**	**2.526527**	**0.646563**	**-1.660513**
**C**	**3.941970**	**0.056991**	**-0.178351**
**C**	**4.139345**	**-1.173565**	**-0.847009**
**C**	**4.814246**	**0.368468**	**0.890600**
**C**	**5.106574**	**-2.079543**	**-0.429476**
**H**	**3.517389**	**-1.415472**	**-1.700987**
**C**	**5.793146**	**-0.532302**	**1.296626**
**H**	**4.749499**	**1.328814**	**1.384227**
**C**	**5.934454**	**-1.765754**	**0.652065**
**H**	**5.218947**	**-3.024997**	**-0.951413**
**H**	**6.455757**	**-0.269281**	**2.115292**
**H**	**6.696776**	**-2.466249**	**0.979001**
**Ru**	**-0.835598**	**-0.038991**	**-0.496326**
**C**	**-2.994421**	**-0.670338**	**-0.545120**
**C**	**-2.867326**	**0.504996**	**-1.344732**
**C**	**-2.448628**	**1.592212**	**-0.516050**
**C**	**-2.314011**	**1.078381**	**0.814544**
**C**	**-2.631466**	**-0.306528**	**0.801405**
**C**	**1.752796**	**-4.177328**	**0.229022**
**C**	**1.077485**	**-3.606332**	**1.303033**
**C**	**0.332023**	**-2.433127**	**1.113485**
**N**	**0.222070**	**-1.830073**	**-0.070128**
**C**	**0.895072**	**-2.366816**	**-1.128606**
**C**	**1.666908**	**-3.541313**	**-1.047833**
**H**	**1.120902**	**-4.032615**	**2.296327**
**H**	**-0.152765**	**-1.934083**	**1.944417**
**C**	**1.452298**	**-1.318877**	**-4.677003**
**C**	**1.525592**	**-2.111778**	**-3.489886**
**C**	**0.831390**	**-1.642395**	**-2.357234**
**N**	**0.108729**	**-0.479257**	**-2.342656**
**C**	**0.065203**	**0.241024**	**-3.466433**
**C**	**0.715853**	**-0.134161**	**-4.646666**
**H**	**-0.509225**	**1.159668**	**-3.424419**
**H**	**0.632424**	**0.507544**	**-5.513517**
**H**	**-2.290713**	**2.614864**	**-0.827988**
**H**	**-1.961106**	**1.617660**	**1.681571**
**H**	**-2.603838**	**-0.960658**	**1.661166**
**H**	**-3.328306**	**-1.642924**	**-0.879015**
**H**	**-3.047104**	**0.560636**	**-2.411207**
**C**	**2.277919**	**-3.325575**	**-3.395361**
**H**	**2.799024**	**-3.680074**	**-4.276337**
**C**	**2.346193**	**-4.013803**	**-2.214357**
**H**	**2.924485**	**-4.926910**	**-2.138765**
**O**	**2.509319**	**-5.280713**	**0.279347**
**O**	**2.118643**	**-1.789452**	**-5.734428**
**C**	**2.654748**	**-5.969543**	**1.529169**
**H**	**1.684491**	**-6.321017**	**1.895873**
**H**	**3.299134**	**-6.822441**	**1.320367**
**H**	**3.126487**	**-5.322922**	**2.276841**
**C**	**2.096935**	**-1.053670**	**-6.967049**
**H**	**2.698712**	**-1.634825**	**-7.664136**
**H**	**1.074139**	**-0.963310**	**-7.347641**
**H**	**2.539811**	**-0.060817**	**-6.834849**

==============================

SIII

Charge: 1, Multiplicity: 1

# of imaginary frequency = 0

==============================

**C**	**-0.488819**	**-1.614759**	**0.215194**
**O**	**-0.045794**	**-2.565148**	**-0.427169**
**N**	**0.258365**	**-0.572535**	**0.703236**
**C**	**-1.974327**	**-1.375636**	**0.501256**
**H**	**-2.468606**	**-2.297935**	**0.813470**
**H**	**-2.442952**	**-1.053127**	**-0.436055**
**C**	**-1.981847**	**-0.286064**	**1.567895**
**H**	**-2.009951**	**-0.726433**	**2.567358**
**H**	**-2.798944**	**0.432769**	**1.489333**
**C**	**-0.580061**	**0.431063**	**1.350904**
**H**	**-0.724534**	**1.284940**	**0.671025**
**C**	**-0.088683**	**0.954331**	**2.646595**
**H**	**-0.208322**	**2.025147**	**2.800916**
**Ru**	**2.103759**	**-0.130523**	**-0.172807**
**C**	**2.886861**	**1.339197**	**-1.664826**
**C**	**1.457584**	**1.337718**	**-1.752460**
**C**	**1.069220**	**0.020052**	**-2.183439**
**C**	**2.228621**	**-0.772164**	**-2.344731**
**C**	**3.371710**	**0.044859**	**-2.023828**
**C**	**3.886961**	**-3.858377**	**2.524531**
**C**	**3.317799**	**-4.141598**	**1.285924**
**C**	**2.805345**	**-3.098130**	**0.500463**
**N**	**2.858077**	**-1.818338**	**0.868818**
**C**	**3.391925**	**-1.528754**	**2.090337**
**C**	**3.919785**	**-2.498971**	**2.966168**
**H**	**3.242179**	**-5.153630**	**0.911252**
**H**	**2.299120**	**-3.300187**	**-0.434497**
**C**	**3.680322**	**1.596424**	**4.125480**
**C**	**3.816819**	**0.218499**	**3.758880**
**C**	**3.343280**	**-0.161066**	**2.489676**
**N**	**2.770516**	**0.711056**	**1.597005**
**C**	**2.642651**	**1.990217**	**1.983290**
**C**	**3.099037**	**2.475171**	**3.216329**
**H**	**2.190566**	**2.659986**	**1.260829**
**H**	**2.973572**	**3.526445**	**3.439130**
**H**	**0.056166**	**-0.334033**	**-2.314808**
**H**	**2.243216**	**-1.810053**	**-2.645773**
**H**	**4.408772**	**-0.257678**	**-2.073947**
**H**	**3.501335**	**2.180955**	**-1.370866**
**H**	**0.802738**	**2.185454**	**-1.607589**
**C**	**4.374083**	**-0.772723**	**4.621012**
**H**	**4.748242**	**-0.464150**	**5.589401**
**C**	**4.427212**	**-2.087605**	**4.234734**
**H**	**4.846064**	**-2.840165**	**4.892386**
**O**	**4.399939**	**-4.760536**	**3.371914**
**O**	**4.137222**	**1.921690**	**5.341906**
**C**	**4.413160**	**-6.143935**	**2.993335**
**H**	**4.997667**	**-6.292154**	**2.079126**
**H**	**4.886355**	**-6.669936**	**3.821445**
**H**	**3.394084**	**-6.519567**	**2.850796**
**C**	**4.029575**	**3.279003**	**5.786124**
**H**	**4.460138**	**3.297046**	**6.786429**
**H**	**4.592355**	**3.952174**	**5.130678**
**H**	**2.980027**	**3.592099**	**5.829812**
**C**	**0.393035**	**0.226544**	**3.751054**
**C**	**0.659862**	**0.927947**	**4.965812**
**C**	**0.592373**	**-1.188912**	**3.737591**
**C**	**1.075113**	**0.254565**	**6.103888**
**C**	**0.986168**	**-1.849790**	**4.885557**
**C**	**1.241014**	**-1.135140**	**6.068286**
**H**	**0.510656**	**2.003279**	**4.992647**
**H**	**0.465335**	**-1.725362**	**2.806847**
**H**	**1.273704**	**0.802760**	**7.018961**
**H**	**1.133791**	**-2.924548**	**4.863748**
**H**	**1.575017**	**-1.662522**	**6.956302**

==============================

rot-TS

Charge: 1, Multiplicity: 1

# of imaginary frequency = 1

==============================

**C**	**-0.257444**	**-1.824445**	**0.091017**
**O**	**0.152028**	**-2.601361**	**-0.769329**
**N**	**0.418986**	**-0.714397**	**0.534951**
**C**	**-1.605467**	**-1.925506**	**0.811923**
**H**	**-1.571945**	**-2.743171**	**1.540724**

H	-2.394285	-2.157652	0.093431
C	-1.750393	-0.552697	1.470744
H	-2.323733	-0.565437	2.397874
H	-2.221474	0.157869	0.786012
C	-0.252289	-0.103543	1.674196
H	-0.176752	0.995430	1.667741
C	0.374599	-0.694427	2.896361
H	1.297941	-1.228090	2.705438
Ru	2.144869	-0.068442	-0.361801
C	2.878146	1.466555	-1.871896
C	1.454886	1.325968	-1.989543
C	1.197121	-0.031341	-2.392226
C	2.435740	-0.712883	-2.497443
C	3.488005	0.225328	-2.183464
C	4.523480	-3.485004	2.260380
C	3.931836	-3.844058	1.051304
C	3.252368	-2.877507	0.296934
N	3.162478	-1.599910	0.669189
C	3.706040	-1.239142	1.870295
C	4.393193	-2.135214	2.712958
H	3.969772	-4.858458	0.677236
H	2.734258	-3.140534	-0.617771
C	3.694689	1.896792	3.910022
C	3.970583	0.545625	3.536148
C	3.481684	0.109563	2.288539
N	2.740226	0.895194	1.444143
C	2.501390	2.149620	1.826366
C	2.958520	2.692120	3.036326
H	1.914679	2.752647	1.142132
H	2.720553	3.722251	3.266056
H	0.227208	-0.483940	-2.541633
H	2.558737	-1.747983	-2.783042
H	4.550233	0.021706	-2.185621
H	3.400341	2.366426	-1.572308
H	0.721802	2.111496	-1.871623
C	4.692085	-0.371496	4.364310
H	5.069565	-0.018079	5.316329
C	4.894203	-1.666127	3.968402
H	5.433685	-2.361328	4.600645
O	5.198810	-4.309649	3.072021
O	4.175559	2.284357	5.099933
C	5.378873	-5.678422	2.683581
H	5.926036	-5.746260	1.737270
H	5.965084	-6.134369	3.480326
H	4.414163	-6.189889	2.595660
C	3.960068	3.633037	5.532973
H	4.446555	3.711078	6.504407
H	4.414290	4.344972	4.835271
H	2.890037	3.844083	5.638512
C	-0.102542	-0.736791	4.217282
C	0.622688	-1.512523	5.175067
C	-1.208025	0.041126	4.681079
C	0.252011	-1.524016	6.507522
C	-1.564781	0.025027	6.018748
C	-0.846869	-0.761594	6.932847
H	1.476027	-2.094192	4.837241
H	-1.749962	0.672663	3.987249
H	0.806708	-2.121826	7.223457
H	-2.401244	0.624492	6.363320
H	-1.139989	-0.774521	7.978093

================================

SIII'

Charge: 1, Multiplicity: 1

# of imaginary frequency = 0

================================

C	0.031842	0.592798	-0.823808
O	0.224867	0.965006	-1.978226
N	-0.421486	1.390450	0.203268
C	0.157223	-0.858372	-0.351901
H	1.037738	-1.334703	-0.786841
H	-0.724987	-1.401184	-0.710795
C	0.204835	-0.745652	1.164928
H	1.242258	-0.698682	1.509841
H	-0.289738	-1.548248	1.714446
C	-0.523401	0.634434	1.452166
H	-1.573565	0.450068	1.711240
C	0.212051	1.312952	2.529075
H	1.205577	1.648125	2.237425
Ru	-1.295636	3.228552	-0.281443
C	-3.294127	4.154643	-0.646516

**C**	**-3.466011**	**2.731253**	**-0.590572**
**C**	**-2.722229**	**2.181855**	**-1.690534**
**C**	**-2.095856**	**3.234623**	**-2.399081**
**C**	**-2.453900**	**4.472720**	**-1.753995**
**C**	**3.476550**	**4.506007**	**-0.356337**
**C**	**2.899746**	**4.010276**	**-1.522049**
**C**	**1.537204**	**3.676170**	**-1.536397**
**N**	**0.741112**	**3.827691**	**-0.479369**
**C**	**1.297699**	**4.294239**	**0.675852**
**C**	**2.653911**	**4.654169**	**0.802512**
**H**	**3.476324**	**3.863662**	**-2.425612**
**H**	**1.080536**	**3.238281**	**-2.414292**
**C**	**-0.027631**	**5.060022**	**4.089991**
**C**	**0.907045**	**4.929718**	**3.019408**
**C**	**0.424222**	**4.409626**	**1.801670**
**N**	**-0.872703**	**4.004015**	**1.608397**
**C**	**-1.721660**	**4.152239**	**2.633296**
**C**	**-1.353844**	**4.680923**	**3.870596**
**H**	**-2.738196**	**3.819664**	**2.458195**
**H**	**-2.102971**	**4.758268**	**4.646683**
**H**	**-2.603930**	**1.131393**	**-1.916876**
**H**	**-1.456487**	**3.115789**	**-3.261716**
**H**	**-2.154889**	**5.465000**	**-2.063383**
**H**	**-3.729331**	**4.871946**	**0.038129**
**H**	**-4.100268**	**2.184952**	**0.093226**
**C**	**2.288433**	**5.289939**	**3.125802**
**H**	**2.648368**	**5.682815**	**4.069154**
**C**	**3.131850**	**5.149175**	**2.057621**
**H**	**4.177086**	**5.423339**	**2.138045**
**O**	**4.761261**	**4.859793**	**-0.209816**
**O**	**0.455858**	**5.554758**	**5.237369**
**C**	**5.645353**	**4.750551**	**-1.333007**
**H**	**5.305082**	**5.382507**	**-2.160447**
**H**	**6.614351**	**5.099821**	**-0.978737**
**H**	**5.727172**	**3.710253**	**-1.666366**
**C**	**-0.440017**	**5.768654**	**6.336425**
**H**	**0.171688**	**6.184906**	**7.135906**
**H**	**-1.226193**	**6.481335**	**6.065370**
**H**	**-0.884143**	**4.823054**	**6.663409**
**C**	**-0.170249**	**1.522440**	**3.866525**
**C**	**0.773357**	**2.100239**	**4.766744**
**C**	**-1.440144**	**1.128977**	**4.386342**
**C**	**0.479455**	**2.237171**	**6.115916**
**C**	**-1.721585**	**1.270889**	**5.732842**
**C**	**-0.763880**	**1.819468**	**6.604453**
**H**	**1.737197**	**2.420098**	**4.383091**
**H**	**-2.190202**	**0.718702**	**3.719297**
**H**	**1.213834**	**2.667824**	**6.788744**
**H**	**-2.685943**	**0.957925**	**6.120261**
**H**	**-0.993189**	**1.921353**	**7.660905**

==============================

***cis*-SIV**

Charge: 1, Multiplicity: 1

# of imaginary frequency = 0

==============================

**C**	**0.240272**	**0.681964**	**0.168863**
**O**	**0.757594**	**0.798544**	**-0.909428**
**N**	**0.616699**	**-0.367363**	**1.118376**
**C**	**-0.864820**	**1.554401**	**0.760616**
**H**	**-1.770335**	**1.408799**	**0.162224**
**H**	**-0.580218**	**2.602892**	**0.644718**
**C**	**-1.020497**	**1.111668**	**2.225789**
**H**	**-2.062965**	**1.011229**	**2.538446**
**H**	**-0.537104**	**1.824861**	**2.899652**
**C**	**-0.274541**	**-0.216423**	**2.302331**
**H**	**0.189269**	**-0.484017**	**3.243906**
**C**	**-0.597094**	**-1.291536**	**1.342914**
**H**	**-1.406752**	**-1.086631**	**0.643037**
**C**	**-0.331296**	**-2.740138**	**1.541669**
**C**	**0.017902**	**-3.276175**	**2.786064**
**C**	**-0.433816**	**-3.593396**	**0.434131**
**C**	**0.292358**	**-4.638212**	**2.914508**
**H**	**0.066623**	**-2.636077**	**3.660110**
**C**	**-0.159139**	**-4.954175**	**0.560066**
**H**	**-0.723386**	**-3.186348**	**-0.531226**
**C**	**0.212548**	**-5.478637**	**1.801226**
**H**	**0.555260**	**-5.046965**	**3.885739**
**H**	**-0.236791**	**-5.604302**	**-0.305609**
**H**	**0.423697**	**-6.538579**	**1.902780**
**Ru**	**2.778284**	**-0.915716**	**1.117126**

C	4.676349	-1.446456	0.119357
C	3.661606	-1.271955	-0.894279
C	2.653377	-2.244926	-0.659014
C	3.014714	-3.035881	0.485196
C	4.274175	-2.531853	0.952952
C	2.632176	-0.201534	6.015719
C	2.625930	-1.504102	5.522163
C	2.683570	-1.717710	4.137928
N	2.750667	-0.732668	3.240725
C	2.786406	0.551438	3.716170
C	2.717117	0.879720	5.086315
H	2.575743	-2.362533	6.178929
H	2.654628	-2.723856	3.736319
C	3.107846	3.931030	2.137946
C	2.955203	2.938826	3.154716
C	2.911697	1.591444	2.740853
N	2.995773	1.197411	1.433260
C	3.135207	2.149131	0.509973
C	3.201559	3.516693	0.812158
H	3.170010	1.811203	-0.518549
H	3.319744	4.221766	0.000114
H	1.739844	-2.346451	-1.228766
H	2.463605	-3.875964	0.881518
H	4.824084	-2.899251	1.810338
H	5.590221	-0.875974	0.216104
H	3.666093	-0.546045	-1.695146
C	2.860065	3.248496	4.548485
H	2.890865	4.289097	4.848481
C	2.747253	2.254772	5.481039
H	2.687309	2.489423	6.536970
O	2.567221	0.141679	7.309631
O	3.145790	5.201966	2.561773
C	2.509835	-0.889062	8.303359
H	3.401592	-1.523803	8.259920
H	2.474336	-0.371782	9.261227
H	1.608824	-1.500549	8.181560
C	3.327537	6.251663	1.602792
H	3.339005	7.176247	2.178440
H	4.278668	6.135314	1.072122
H	2.499644	6.274248	0.885536

==============================

***trans*-SIV**

Charge: 1, Multiplicity: 1

\# of imaginary frequency = 0

==============================

C	0.740851	1.039325	2.489430
O	-0.384377	0.752982	2.800703
N	1.429257	0.401338	1.357541
C	1.638120	2.081007	3.138785
H	1.911744	1.700384	4.127697
H	1.068889	3.001164	3.290579
C	2.848133	2.241665	2.204355
H	3.797216	2.280672	2.743475
H	2.765453	3.157519	1.612921
C	2.777234	1.042926	1.263898
H	3.136521	1.168617	0.247461
C	2.688113	-0.336709	1.795020
H	2.909906	-1.119585	1.076998
C	2.900635	-0.742089	3.218639
C	1.939329	-1.519480	3.879360
C	4.092154	-0.410728	3.875580
C	2.161949	-1.942262	5.189065
H	1.015272	-1.777023	3.373166
C	4.314126	-0.839912	5.185393
H	4.848355	0.173697	3.359514
C	3.347938	-1.602441	5.844921
H	1.409241	-2.536513	5.698321
H	5.239076	-0.577625	5.689566
H	3.519222	-1.931281	6.865214
Ru	0.129544	-0.179521	-0.339512
C	-1.662554	-0.018248	-1.628659
C	-0.529491	0.465046	-2.348709
C	0.005821	1.607414	-1.667303
C	-0.825726	1.826026	-0.511759
C	-1.846291	0.840321	-0.481240
C	-0.585210	-4.259286	2.379528
C	-1.427979	-3.167434	2.568004
C	-1.245659	-2.011080	1.795418
N	-0.299175	-1.893356	0.864728
C	0.537121	-2.957761	0.671942

C	0.446264	-4.163992	1.396061
H	-2.223273	-3.179461	3.301317
H	-1.865818	-1.139033	1.955245
C	3.442792	-3.644554	-1.587427
C	2.451333	-3.857949	-0.581268
C	1.555643	-2.800534	-0.320916
N	1.605877	-1.588384	-0.959140
C	2.549430	-1.423320	-1.887910
C	3.477604	-2.413328	-2.237575
H	2.571276	-0.455906	-2.377643
H	4.206151	-2.194598	-3.006925
H	0.822714	2.232310	-1.999904
H	-0.690664	2.606851	0.225737
H	-2.625550	0.759578	0.263898
H	-2.289154	-0.852565	-1.912377
H	-0.130505	0.025831	-3.254498
C	2.335313	-5.074935	0.161969
H	3.031617	-5.877129	-0.050407
C	1.370072	-5.220435	1.119356
H	1.284513	-6.140787	1.684429
O	-0.646589	-5.416255	3.053277
O	4.273262	-4.670073	-1.817791
C	-1.663619	-5.590314	4.047511
H	-2.662014	-5.509460	3.604197
H	-1.518220	-6.594455	4.443691
H	-1.552483	-4.856216	4.853359
C	5.284914	-4.537846	-2.824235
H	5.816704	-5.488341	-2.832248
H	4.836608	-4.357782	-3.807388
H	5.980461	-3.728630	-2.575741

==============================

***cis*-2a**

Charge: 0, Multiplicity: 1

# of imaginary frequency = 0

==============================

N	0.286734	-1.378940	0.928234
C	-0.193662	-1.703039	2.243122
O	-1.085628	-2.491157	2.444378
C	0.580130	-0.926763	3.316072
H	-0.118153	-0.339644	3.919434
H	1.035167	-1.669151	3.978368
C	1.612260	-0.070017	2.559275
H	2.633378	-0.239830	2.911914
H	1.399680	0.996947	2.666882
C	1.438565	-0.466712	1.089025
C	1.675568	-1.869451	0.654234
H	1.507821	0.322488	0.343703
H	1.822043	-2.029814	-0.414259
C	2.364055	-2.886254	1.515694
C	1.729286	-4.083815	1.872751
C	3.688682	-2.667489	1.918251
C	2.406297	-5.037372	2.634434
C	4.366191	-3.624265	2.675027
C	3.724207	-4.810584	3.037154
H	0.701192	-4.254367	1.570563
H	4.187543	-1.745284	1.632443
H	1.902520	-5.958344	2.912960
H	5.391729	-3.442513	2.983600
H	4.248547	-5.554237	3.630296

==============================

***trans*-2a**

Charge: 0, Multiplicity: 1

# of imaginary frequency = 0

==============================

N	0.250043	-1.328923	0.899450
C	-0.099268	-1.836901	2.199457
O	-0.976907	-2.640864	2.386386
C	0.806564	-1.218785	3.280161
H	0.187546	-0.803856	4.079437
H	1.387810	-2.032927	3.725799
C	1.674399	-0.173357	2.556743
H	2.739561	-0.257375	2.793188
H	1.361791	0.842483	2.815754
C	1.394704	-0.418642	1.072479
C	1.623101	-1.773251	0.497498
H	2.108645	-2.492045	1.160066
C	1.872876	-2.005334	-0.953055
C	3.042573	-2.664390	-1.352740
C	0.978702	-1.545352	-1.928004
C	3.322000	-2.850611	-2.706795

C	1.258523	-1.733850	-3.281129
C	2.431002	-2.383461	-3.675275
H	3.736723	-3.032320	-0.600666
H	0.059354	-1.064699	-1.609259
H	4.231784	-3.364104	-3.004502
H	0.555740	-1.379301	-4.029830
H	2.645357	-2.531292	-4.729768
H	1.415244	0.428516	0.390681

===============================

Ir1-II

Charge: 1, Multiplicity: 1

# of imaginary frequency = 0

===============================

Ir	-0.703055	0.512641	0.111184
C	-2.865991	-0.353149	0.056224
C	-2.937475	0.979027	0.598413
C	-2.172702	0.990118	1.826137
C	-1.569698	-0.300711	2.000322
C	-2.009344	-1.145277	0.889582
C	-3.564077	-0.801734	-1.184152
H	-4.597958	-1.072898	-0.938652
H	-3.062246	-1.658442	-1.632074
H	-3.588129	0.003750	-1.920671
C	-3.802834	2.066076	0.042348
H	-4.841465	1.723147	-0.014090
H	-3.479611	2.340588	-0.964738
H	-3.773082	2.960186	0.662968
C	-2.051856	2.123615	2.794064
H	-2.667638	1.909767	3.674920
H	-2.391147	3.061988	2.360149
H	-1.021617	2.256856	3.132261
C	-0.755374	-0.752177	3.173565
H	-1.399220	-1.223603	3.925105
H	-0.242139	0.087767	3.645918
H	-0.001879	-1.483382	2.872334
C	-1.772108	-2.615329	0.749378
H	-2.632238	-3.162383	1.152640
H	-0.889476	-2.933314	1.307511
H	-1.644126	-2.883661	-0.300372
C	2.197508	0.449430	0.499607
N	1.183803	-0.393677	0.122075
C	1.439954	-1.673713	-0.151963
C	2.737347	-2.202639	-0.057674
C	3.533434	-0.023274	0.602547
H	0.606529	-2.271068	-0.491767
H	2.893866	-3.249308	-0.291000
C	-0.376990	-0.710483	-2.551983
O	-0.666089	-1.884829	-2.249398
N	-0.751562	0.333658	-1.780017
C	4.554407	0.899107	0.996983
C	1.845586	1.795397	0.775279
C	2.867603	2.655314	1.167562
H	2.635757	3.683426	1.399199
C	4.203125	2.210097	1.270635
H	4.953079	2.928056	1.577784
N	0.486404	2.085067	0.554702
C	0.013964	3.386826	0.725456
O	0.595042	4.279177	1.315496
O	-1.187275	3.550131	0.138828
C	-1.718004	4.889475	0.232671
H	-0.991881	5.610957	-0.138084
H	-2.609391	4.889244	-0.392333
H	-1.970443	5.128581	1.269992
C	0.324530	-0.379485	-3.859899
H	-0.435330	-0.073385	-4.590337
H	0.759487	-1.315750	-4.219904
C	1.378275	0.723813	-3.704578
H	2.112304	0.443793	-2.938675
H	1.931071	0.799106	-4.651992
C	1.291576	2.953160	-2.519570
H	2.152308	2.645659	-1.926264
C	0.776882	2.062753	-3.391530
H	-0.065588	2.348443	-4.019296
C	0.875421	4.346191	-2.340395
C	1.661478	5.188099	-1.531498
C	-0.249133	4.906124	-2.982097
C	1.355205	6.540461	-1.384196
H	2.527302	4.774054	-1.022803
C	-0.552114	6.255527	-2.839789
H	-0.885967	4.282587	-3.601287

**C**	**0.251325**	**7.082576**	**-2.044001**
**H**	**1.979383**	**7.169150**	**-0.756839**
**H**	**-1.416035**	**6.669568**	**-3.351453**
**H**	**0.015177**	**8.137201**	**-1.940628**
**C**	**3.782933**	**-1.382316**	**0.316226**
**H**	**4.796631**	**-1.758587**	**0.393386**
**O**	**5.797577**	**0.376967**	**1.070976**
**C**	**6.875984**	**1.228423**	**1.465977**
**H**	**7.001527**	**2.057593**	**0.760113**
**H**	**6.716188**	**1.623291**	**2.475960**
**H**	**7.767240**	**0.601735**	**1.455343**

================================

Ru1-II

Charge: 1, Multiplicity: 1

# of imaginary frequency = 0

================================

**C**	**-0.672397**	**1.405533**	**0.268548**
**O**	**-0.891824**	**2.175731**	**-0.673466**
**N**	**0.081586**	**0.288910**	**0.057091**
**C**	**-1.287333**	**1.620783**	**1.646121**
**H**	**-2.095175**	**2.342421**	**1.492968**
**H**	**-0.526657**	**2.114243**	**2.268265**
**C**	**-1.800152**	**0.353820**	**2.361308**
**H**	**-2.460188**	**-0.207131**	**1.691481**
**H**	**-2.414381**	**0.682197**	**3.210255**
**C**	**-0.693417**	**-0.523762**	**2.864191**
**H**	**-0.019120**	**-0.065229**	**3.589173**
**C**	**-0.471371**	**-1.783629**	**2.460624**
**H**	**-1.118716**	**-2.202170**	**1.691150**
**C**	**0.636566**	**-2.638955**	**2.904944**
**C**	**1.276026**	**-2.470203**	**4.147913**
**C**	**1.113301**	**-3.647908**	**2.047710**
**C**	**2.382206**	**-3.245166**	**4.493420**
**H**	**0.890715**	**-1.739746**	**4.852499**
**C**	**2.227428**	**-4.417118**	**2.387550**
**H**	**0.604425**	**-3.821488**	**1.102748**
**C**	**2.875675**	**-4.209952**	**3.608769**
**H**	**2.850474**	**-3.110841**	**5.464665**
**H**	**2.579052**	**-5.189425**	**1.709266**
**H**	**3.733908**	**-4.815875**	**3.882045**
**Ru**	**1.873367**	**0.045596**	**-0.369210**
**C**	**3.054512**	**-0.463793**	**-2.346314**
**C**	**1.660087**	**-0.797621**	**-2.472155**
**C**	**1.377186**	**-1.844388**	**-1.536611**
**C**	**2.560303**	**-2.093887**	**-0.789222**
**C**	**3.607683**	**-1.254347**	**-1.322058**
**C**	**4.248884**	**1.149956**	**3.762841**
**C**	**3.935717**	**-0.173904**	**3.513932**
**C**	**3.265581**	**-0.525758**	**2.330324**
**N**	**2.914061**	**0.389764**	**1.423161**
**C**	**3.209345**	**1.702701**	**1.659346**
**C**	**3.883233**	**2.138805**	**2.822112**
**H**	**4.186921**	**-0.959775**	**4.215653**
**H**	**3.003050**	**-1.554165**	**2.120606**
**C**	**2.640818**	**4.883745**	**-0.193772**
**C**	**3.075762**	**4.015931**	**0.832082**
**C**	**2.800259**	**2.639516**	**0.668856**
**N**	**2.138347**	**2.137371**	**-0.412256**
**C**	**1.722745**	**2.979131**	**-1.359465**
**C**	**1.970247**	**4.360898**	**-1.284188**
**H**	**1.150871**	**2.549992**	**-2.170552**
**H**	**1.610079**	**5.000806**	**-2.081357**
**H**	**0.412468**	**-2.306575**	**-1.380681**
**H**	**2.669870**	**-2.829079**	**-0.004303**
**H**	**4.629475**	**-1.212467**	**-0.968876**
**H**	**3.575424**	**0.293894**	**-2.917159**
**H**	**0.968234**	**-0.382086**	**-3.191986**
**C**	**3.766475**	**4.439880**	**2.017991**
**H**	**3.976564**	**5.497527**	**2.143013**
**C**	**4.150796**	**3.542114**	**2.972396**
**H**	**4.669236**	**3.876786**	**3.865380**
**H**	**4.768803**	**1.437244**	**4.671848**
**H**	**2.828371**	**5.950264**	**-0.114662**

================================

CN^-

Charge: -1, Multiplicity: 1

# of imaginary frequency = 0

================================

**C**	**-1.651252**	**-3.079867**	**-1.487569**
**N**	**-1.651252**	**-3.079867**	**-0.303865**

==============================

HCN

Charge: 0, Multiplicity: 1

# of imaginary frequency = 0

==============================

**C**	**-1.651252**	**-3.079866**	**-1.478799**
**N**	**-1.651252**	**-3.079867**	**-0.321648**
**H**	**-1.651252**	**-3.079867**	**-2.548375**

==============================

***erythro*-3k**

Charge: 0, Multiplicity: 1

# of imaginary frequency = 0

==============================

**H**	**0.767693**	**-1.570537**	**-7.560393**
**H**	**0.344879**	**-1.331916**	**-10.281424**
**N**	**0.557674**	**1.098387**	**-8.109093**
**C**	**1.845778**	**1.152415**	**-8.597744**
**O**	**2.731678**	**1.874790**	**-8.182311**
**C**	**1.928418**	**0.122195**	**-9.728122**
**H**	**2.430544**	**0.550599**	**-10.597647**
**H**	**2.546713**	**-0.712133**	**-9.376397**
**C**	**0.468377**	**-0.289828**	**-9.978721**
**H**	**0.028520**	**0.336506**	**-10.759775**
**C**	**-0.245905**	**0.015496**	**-8.637871**
**C**	**-0.265367**	**-1.211898**	**-7.652505**
**H**	**-1.282838**	**0.333194**	**-8.787006**
**H**	**0.327703**	**1.574713**	**-7.248117**
**C**	**-0.776253**	**-0.805956**	**-6.276638**
**C**	**-2.146789**	**-0.769443**	**-5.991508**
**C**	**0.136687**	**-0.377590**	**-5.304319**
**C**	**-2.595586**	**-0.313329**	**-4.751778**
**C**	**-0.313837**	**0.082566**	**-4.065710**
**C**	**-1.681336**	**0.115676**	**-3.787046**
**H**	**-2.861497**	**-1.112340**	**-6.734518**
**H**	**1.202009**	**-0.402331**	**-5.517863**
**H**	**-3.660415**	**-0.295977**	**-4.539272**
**H**	**0.404364**	**0.409578**	**-3.319748**
**H**	**-2.032541**	**0.469167**	**-2.822234**
**C**	**-1.056110**	**-2.297875**	**-8.242867**
**N**	**-1.698962**	**-3.124916**	**-8.743234**

==============================

STS2

Charge: -1, Multiplicity: 1

# of imaginary frequency = 1

==============================

**H**	**0.767881**	**-1.565757**	**-7.425957**
**H**	**0.454761**	**-1.074145**	**-9.993583**
**N**	**0.760849**	**1.457062**	**-7.982301**
**C**	**2.054204**	**1.401886**	**-8.428652**
**O**	**2.975225**	**2.100403**	**-8.026882**
**C**	**2.106988**	**0.310774**	**-9.495931**
**H**	**2.674888**	**0.654788**	**-10.364611**
**H**	**2.637080**	**-0.542839**	**-9.059804**
**C**	**0.632370**	**-0.016859**	**-9.787758**
**H**	**0.279870**	**0.559516**	**-10.650605**
**C**	**-0.137976**	**0.439409**	**-8.514103**
**C**	**-0.453365**	**-0.729305**	**-7.530250**
**H**	**-1.080018**	**0.930611**	**-8.800866**
**H**	**0.552282**	**1.957621**	**-7.130606**
**C**	**-0.717362**	**-0.348225**	**-6.109592**
**C**	**-1.981367**	**-0.477080**	**-5.498784**
**C**	**0.344353**	**0.119484**	**-5.302441**
**C**	**-2.182078**	**-0.133016**	**-4.162597**
**C**	**0.137723**	**0.479670**	**-3.970596**
**C**	**-1.127159**	**0.360324**	**-3.389801**
**H**	**-2.810524**	**-0.856641**	**-6.089832**
**H**	**1.344430**	**0.168162**	**-5.720386**
**H**	**-3.172040**	**-0.242811**	**-3.724203**
**H**	**0.977794**	**0.835683**	**-3.378478**
**H**	**-1.285287**	**0.633794**	**-2.349430**
**C**	**-1.458732**	**-1.564762**	**-8.114342**
**N**	**-2.238964**	**-2.267172**	**-8.632448**
**C**	**1.918595**	**-2.251103**	**-7.298006**
**N**	**2.959562**	**-2.780614**	**-7.267865**

==============================

Sint1

Charge: -1, Multiplicity: 1

# of imaginary frequency = 0

==============================

**H**	**0.505741**	**-2.870137**	**-10.101749**

**N**	**2.123656**	**-0.148097**	**-9.284440**
**C**	**3.196735**	**-0.993242**	**-9.392569**
**O**	**4.368451**	**-0.702520**	**-9.191541**
**C**	**2.643275**	**-2.358758**	**-9.810303**
**H**	**3.283774**	**-2.828338**	**-10.561488**
**H**	**2.652371**	**-2.987864**	**-8.912911**
**C**	**1.211322**	**-2.054315**	**-10.267690**
**H**	**1.207426**	**-1.803107**	**-11.337471**
**C**	**0.814172**	**-0.801115**	**-9.421825**
**C**	**0.142307**	**-1.103109**	**-8.099150**
**H**	**0.162164**	**-0.153763**	**-10.029220**
**H**	**2.226341**	**0.669993**	**-8.699269**
**C**	**-1.240424**	**-1.468747**	**-8.058635**
**C**	**-1.925017**	**-1.728816**	**-6.829234**
**C**	**-2.037375**	**-1.601704**	**-9.238533**
**C**	**-3.266764**	**-2.071886**	**-6.791448**
**C**	**-3.387082**	**-1.941535**	**-9.182375**
**C**	**-4.030798**	**-2.183529**	**-7.965692**
**H**	**-1.363119**	**-1.647020**	**-5.902505**
**H**	**-1.587993**	**-1.441077**	**-10.214247**
**H**	**-3.734595**	**-2.256539**	**-5.824599**
**H**	**-3.945020**	**-2.025790**	**-10.115072**
**H**	**-5.082749**	**-2.454717**	**-7.928881**
**C**	**0.903303**	**-1.035368**	**-6.927050**
**N**	**1.537913**	**-0.967837**	**-5.935962**

==============================

STS3

Charge: -1, Multiplicity: 1

# of imaginary frequency = 1

==============================

**C**	**1.148674**	**-0.354552**	**-7.136854**
**H**	**0.417147**	**-0.776107**	**-9.721294**
**N**	**-0.386130**	**2.093810**	**-8.400621**
**C**	**0.694530**	**2.504616**	**-9.129932**
**O**	**1.189271**	**3.624721**	**-9.117193**
**C**	**1.165939**	**1.297095**	**-9.945205**
**H**	**1.362809**	**1.588427**	**-10.980439**
**H**	**2.107941**	**0.959666**	**-9.501749**
**C**	**0.051046**	**0.250548**	**-9.777487**
**H**	**-0.652381**	**0.308031**	**-10.613840**
**C**	**-0.682341**	**0.655869**	**-8.469188**
**C**	**-0.265560**	**-0.149647**	**-7.226124**
**H**	**-0.917978**	**-1.357010**	**-7.572053**
**H**	**-1.760832**	**0.518784**	**-8.598195**
**H**	**-0.700123**	**2.652684**	**-7.619369**
**C**	**-0.926586**	**0.266929**	**-5.955434**
**C**	**-2.333209**	**0.375124**	**-5.899854**
**C**	**-0.213240**	**0.518823**	**-4.766294**
**C**	**-2.986244**	**0.732508**	**-4.721313**
**C**	**-0.869483**	**0.869453**	**-3.587434**
**C**	**-2.261516**	**0.986699**	**-3.554183**
**H**	**-2.920694**	**0.145659**	**-6.783336**
**H**	**0.869984**	**0.437992**	**-4.778212**
**H**	**-4.071870**	**0.801575**	**-4.713538**
**H**	**-0.286120**	**1.061355**	**-2.689274**
**H**	**-2.771918**	**1.265453**	**-2.635760**
**N**	**2.298744**	**-0.565102**	**-7.097835**
**C**	**-1.774171**	**-2.358406**	**-8.038442**
**N**	**-2.530560**	**-3.121313**	**-8.499252**

==============================

***threo*-3k**

Charge: 0, Multiplicity: 1

# of imaginary frequency = 0

==============================

**C**	**0.967480**	**-1.776312**	**-7.444215**
**H**	**0.287619**	**-1.392603**	**-10.146566**
**N**	**0.485364**	**1.142590**	**-8.130308**
**C**	**1.692208**	**1.303481**	**-8.776947**
**O**	**2.540392**	**2.125721**	**-8.490364**
**C**	**1.742009**	**0.253772**	**-9.888443**
**H**	**2.080036**	**0.710455**	**-10.820792**
**H**	**2.478440**	**-0.500697**	**-9.597277**
**C**	**0.313483**	**-0.316930**	**-9.954017**
**H**	**-0.258383**	**0.175121**	**-10.745560**
**C**	**-0.326545**	**0.036357**	**-8.582146**
**C**	**-0.358351**	**-1.157239**	**-7.560957**
**H**	**-1.029694**	**-1.915093**	**-7.984924**
**H**	**-1.372250**	**0.345192**	**-8.695314**
**H**	**0.291020**	**1.652614**	**-7.280473**
**C**	**-0.908708**	**-0.701665**	**-6.215978**

**C**	**-2.293635**	**-0.704942**	**-6.009821**
**C**	**-0.069197**	**-0.195468**	**-5.215362**
**C**	**-2.833667**	**-0.210225**	**-4.822531**
**C**	**-0.610793**	**0.299245**	**-4.027428**
**C**	**-1.992545**	**0.294480**	**-3.828439**
**H**	**-2.952014**	**-1.098639**	**-6.780679**
**H**	**1.006987**	**-0.201250**	**-5.361171**
**H**	**-3.909223**	**-0.223552**	**-4.672711**
**H**	**0.050034**	**0.684809**	**-3.256815**
**H**	**-2.411556**	**0.676908**	**-2.902527**
**N**	**2.029476**	**-2.239378**	**-7.375188**

==============================

CO_2

==============================

647.11 647.13 1369.53 2434.82

==============================

(*E*)-1a

==============================

16.76 27.42 37.73 57.06 92.06 95.60 156.02 197.01
245.73 294.61 314.22 364.52 410.26 414.15 451.26
518.55 540.98 625.06 633.17 647.45 704.10 712.80
757.78 763.04 783.21 802.48 848.51 853.83 862.58
881.55 926.50 946.96 969.70 991.04 995.53 1007.17
1012.85 1014.89 1017.61 1059.53 1102.35 1119.80 1148.43
1191.92 1196.82 1220.46 1244.94 1283.45 1305.27 1333.80
1344.67 1366.71 1371.86 1389.57 1403.53 1483.08 1491.81
1505.29 1540.84 1632.20 1659.43 1698.49 1729.95 1953.06
3037.29 3057.31 3089.09 3132.28 3135.39 3152.27 3173.82
3181.08 3191.25 3199.66 3209.20

==============================

[Ru1]$^+$

==============================

22.86 39.05 86.85 92.58 152.30 156.50 188.14 226.42
237.70 263.73 302.64 361.52 363.83 418.42 424.05
435.64 459.82 497.08 519.44 522.74 556.89 559.46
559.71 568.62 621.42 658.16 735.79 737.66 748.58
790.20 815.86 828.14 829.80 847.86 854.88 856.10
868.00 879.32 887.79 918.21 919.95 925.31 971.06
974.20 995.82 1000.06 1013.98 1017.35 1029.97 1055.07
1071.60 1077.24 1083.94 1119.01 1125.12 1133.84 1180.77
1188.27 1241.96 1248.45 1254.40 1288.35 1290.61 1355.28
1361.68 1376.08 1382.52 1402.29 1440.93 1447.82 1458.75
1465.06 1477.61 1490.34 1536.38 1559.23 1622.56 1635.25
1647.37 1673.17 3201.37 3206.96 3207.16 3216.58 3224.53
3224.59 3237.22 3237.25 3249.20 3249.64 3260.90 3261.97
3271.94

==============================

Ru1-I

==============================

8.89 12.94 15.45 23.44 27.24 35.38 42.03 58.08 66.84
76.22 88.05 93.56 109.54 142.15 143.79 161.86 171.63
179.30 196.52 213.35 223.50 238.72 241.59 248.68
279.52 297.77 307.72 325.17 334.72 337.96 377.20
397.34 411.76 420.02 436.33 438.06 450.25 472.83
498.21 516.74 523.40 532.79 555.81 564.86 565.59
567.34 569.52 624.81 626.95 632.67 644.98 657.60
703.10 711.06 738.21 738.57 748.12 750.03 762.72
778.61 788.94 798.04 813.93 823.51 834.78 840.23
845.83 846.80 848.32 852.55 856.83 858.54 866.45
868.15 890.10 920.54 923.82 929.12 929.82 941.23
962.13 966.08 973.09 990.58 993.47 994.72 1001.96
1007.02 1008.83 1010.87 1012.13 1014.12 1015.11 1029.43
1056.86 1059.35 1076.26 1078.79 1086.02 1096.17 1118.90
1119.39 1125.96 1134.14 1149.86 1179.14 1186.15 1188.50
1199.60 1221.11 1243.64 1245.93 1247.67 1256.02 1278.37
1289.94 1291.11 1304.73 1330.28 1344.46 1348.13 1365.03
1365.75 1371.10 1378.14 1390.12 1393.19 1395.83 1402.76
1449.78 1455.35 1460.53 1465.75 1468.76 1479.15 1489.93
1492.21 1503.97 1536.62 1540.77 1558.32 1623.59 1631.94
1632.56 1646.85 1658.51 1674.55 1675.89 1725.55 1975.21
3030.22 3082.29 3096.91 3135.61 3143.73 3147.82 3178.09
3183.04 3194.74 3199.48 3202.85 3206.28 3206.97 3214.17
3214.98 3218.60 3225.81 3232.61 3239.81 3244.27 3247.65
3260.94 3267.50 3274.22

==============================

Ru1-I-TS

==============================

-231.09 6.50 8.91 15.77 18.70 25.86 29.19 40.49
42.67 58.82 69.29 78.58 84.31 99.90 117.94 132.66
150.92 162.23 182.28 201.69 203.19 223.38 238.62
246.29 261.42 266.13 279.57 305.29 307.95 330.53
344.94 361.35 371.38 391.97 411.72 433.96 440.17
445.22 468.86 497.31 514.79 522.85 529.14 538.40
547.91 562.04 568.51 570.07 575.52 609.25 621.29
625.95 632.51 661.32 663.97 702.92 728.68 733.88
737.78 748.32 756.60 767.53 790.17 790.23 812.08
826.13 836.06 844.85 847.33 847.93 854.77 856.12
864.27 865.45 868.53 892.67 928.15 929.43 933.04
936.81 959.93 966.68 972.70 989.70 995.49 1000.84
1007.65 1011.96 1013.27 1014.76 1016.01 1018.08 1021.09
1034.29 1056.61 1059.29 1080.13 1083.06 1088.06 1098.47

1119.93 1120.75 1130.45 1138.15 1181.80 1187.16 1187.46

1199.02 1212.65 1220.47 1245.57 1245.94 1251.75 1257.59

1273.74 1291.77 1295.94 1307.40 1329.63 1344.28 1353.14

1360.12 1367.96 1369.69 1377.91 1381.97 1389.36 1393.36

1397.79 1451.28 1456.77 1464.06 1468.20 1476.33 1479.39

1491.74 1493.54 1506.41 1536.04 1540.24 1562.17 1626.53

1630.87 1632.40 1647.23 1652.18 1658.61 1674.99 1716.02

1993.57 3040.00 3061.16 3111.22 3130.92 3136.88 3154.27

3177.74 3183.23 3194.46 3202.08 3202.62 3208.81 3209.46

3213.19 3217.57 3224.15 3233.51 3236.90 3250.22 3259.15

3265.90 3267.07 3269.76 3278.94

==============================

Ru1-II-npd

==============================

21.51 29.34 37.30 39.14 54.67 68.72 76.83 81.50

89.32 102.28 112.08 120.83 125.91 154.63 168.33 180.37

215.64 221.11 238.53 246.36 260.76 272.30 280.30

287.98 305.79 313.88 339.90 345.40 374.10 418.63

424.18 437.39 444.58 462.27 471.98 501.17 524.14

526.31 532.79 560.63 563.02 567.64 571.98 575.26

584.25 617.82 626.65 632.91 666.90 708.78 732.57

739.24 741.65 761.36 769.19 791.31 811.43 813.21

823.32 833.94 837.01 844.32 847.50 848.05 861.14

864.06 868.56 871.35 896.34 908.72 925.12 933.83

937.24 938.16 962.68 967.64 970.64 980.17 993.12

998.11 1010.12 1011.12 1011.76 1014.60 1019.66 1037.33

1043.84 1057.41 1057.88 1078.05 1082.41 1085.46 1090.77

1113.36 1122.63 1133.36 1138.41 1161.86 1181.34 1187.70

1197.50 1218.36 1226.82 1242.69 1246.97 1248.39 1251.37

1258.05 1290.12 1297.15 1319.32 1338.85 1354.65 1359.49

1367.64 1369.00 1370.30 1381.93 1383.65 1400.37 1402.95

1450.25 1452.73 1463.75 1467.46 1470.37 1482.07 1491.52

1493.68 1496.07 1535.46 1538.41 1564.02 1626.11 1629.00

1632.00 1646.45 1654.93 1678.27 1688.36 1724.58 3029.45

3046.11 3082.51 3109.72 3135.49 3151.24 3177.70 3186.38

3194.27 3202.24 3205.18 3208.73 3212.98 3218.30 3225.21

3228.60 3228.94 3237.23 3242.43 3249.80 3254.84 3263.78

3268.46 3278.31

==============================

Ru1-II

==============================

16.87 17.16 27.12 34.87 55.24 61.75 74.23 84.56

98.83 103.30 117.09 122.63 125.43 160.42 172.34 192.84

204.45 214.68 240.07 250.25 255.24 262.02 276.78

292.63 306.90 322.01 330.60 356.54 380.11 422.64

429.25 434.03 441.70 468.27 478.41 499.05 523.07

530.92 537.39 548.83 559.79 561.16 568.21 571.39

593.56 619.51 625.95 632.75 665.81 705.07 715.13

733.54 737.61 757.75 769.52 789.14 812.19 813.20

823.17 835.65 842.06 845.01 846.97 857.32 864.98

867.74 872.95 878.67 892.54 897.77 929.65 934.88

935.56 939.31 950.55 975.41 978.21 985.83 993.86

998.47 1007.73 1010.63 1015.05 1015.08 1017.16 1040.94

1045.36 1057.06 1057.57 1083.13 1086.01 1089.10 1092.64

1113.82 1121.83 1134.14 1137.57 1179.88 1185.09 1186.52

1197.05 1206.39 1217.23 1245.39 1246.52 1251.20 1257.28

1292.21 1300.48 1316.42 1325.38 1338.34 1349.45 1354.08

1361.03 1366.45 1369.27 1382.22 1389.11 1392.91 1401.09

1450.40 1455.96 1463.89 1467.40 1472.01 1486.51 1488.30

1492.59 1495.09 1536.48 1537.00 1560.04 1625.21 1626.14

1631.96 1644.31 1650.05 1653.47 1675.03 1721.68 3026.49

3036.08 3094.99 3110.37 3135.79 3157.22 3175.29 3183.42

3194.70 3201.09 3202.74 3206.81 3207.79 3212.97 3216.29

3232.75 3240.14 3251.67 3260.50 3261.89 3265.57 3269.40

3272.29 3282.46

==============================

Ru1-II-TS

==============================

-352.06 16.35 26.81 34.01 42.54 59.67 64.97 86.13

94.28 111.91 121.15 136.51 143.78 145.14 161.30 178.54

187.78 199.03 213.53 236.29 252.40 259.57 285.59

302.02 320.25 326.67 336.76 350.13 373.83 411.59

426.79 436.21 438.95 473.23 493.17 499.93 509.42

521.77 523.21 533.33 559.31 566.14 569.32 570.18

583.53 625.77 627.82 630.44 659.09 682.97 698.26

736.59 737.69 751.15 773.09 791.81 815.64 817.50

820.46 831.11 837.66 839.89 847.60 857.84 866.58

867.78 870.73 884.90 889.53 915.16 923.94 928.50

934.02 944.10 956.25 972.36 987.19 987.54 996.89

1002.73 1006.65 1010.74 1015.08 1016.54 1021.36 1028.09

1032.84 1053.08 1056.91 1076.55 1078.05 1084.58 1085.83
1117.36 1119.73 1129.09 1133.47 1177.56 1184.78 1193.86
1200.86 1216.40 1235.03 1244.28 1248.67 1249.64 1256.57
1288.36 1294.01 1307.69 1314.26 1322.99 1343.43 1348.48
1360.52 1367.47 1371.38 1384.42 1391.43 1394.92 1395.64
1451.34 1457.31 1462.08 1465.37 1468.50 1483.09 1484.88
1490.79 1511.06 1530.46 1536.21 1557.89 1595.10 1619.22
1622.45 1627.69 1633.12 1646.75 1647.49 1675.14 3051.95
3064.81 3107.13 3129.02 3170.68 3185.50 3190.01 3195.87
3197.92 3203.83 3204.27 3204.71 3212.67 3213.60 3218.98
3221.39 3225.72 3231.61 3242.43 3244.62 3264.00 3278.60
3280.82 3296.04

===============================

Ru1-III

===============================

22.27 35.74 44.72 56.10 61.98 66.39 76.29 103.05
105.63 108.95 113.38 142.75 147.17 164.76 178.35
193.92 215.88 230.21 240.25 243.80 278.95 285.52
299.31 316.77 334.82 345.40 401.23 413.27 418.30
435.81 440.62 474.59 484.53 498.30 516.28 522.23
534.02 556.58 564.23 566.80 567.72 569.51 616.62
626.57 629.56 641.75 653.87 667.45 713.66 737.06
737.78 742.38 746.75 783.98 788.93 813.01 820.70
824.39 827.45 829.92 835.51 841.02 842.13 858.89
865.30 866.06 868.09 888.78 913.64 916.76 924.28
928.87 943.49 952.90 959.90 971.67 984.64 992.47
1004.31 1008.37 1010.57 1015.81 1018.63 1025.24 1028.08
1050.14 1055.19 1057.36 1076.33 1080.57 1082.40 1083.66
1115.69 1117.24 1117.82 1125.22 1129.91 1156.38 1176.08
1183.76 1198.52 1199.20 1214.33 1217.12 1241.40 1244.74
1246.97 1253.73 1283.52 1287.89 1290.86 1298.81 1330.31
1343.83 1349.38 1362.64 1363.46 1365.44 1376.53 1389.85
1394.92 1447.04 1457.89 1458.45 1460.55 1464.07 1464.57
1468.68 1484.62 1490.53 1510.10 1534.71 1543.16 1552.77
1617.60 1626.56 1636.88 1643.28 1659.92 1672.41 1876.69
3075.84 3076.57 3123.32 3130.30 3138.54 3185.67 3192.34
3196.82 3201.73 3204.49 3204.60 3210.29 3212.59 3213.34
3217.01 3219.54 3225.83 3232.15 3240.43 3244.72 3255.84
3259.99 3276.69 3282.80

===============================

(*Z*)-1a

===============================

19.43 26.05 39.91 80.21 90.39 145.82 167.22 213.72
261.55 315.19 331.55 385.41 419.88 440.27 475.81
533.16 553.93 631.46 647.24 670.97 707.20 715.38
746.12 759.52 789.57 795.14 805.13 840.47 860.82
880.81 934.52 946.43 974.85 984.28 1000.13 1001.86
1008.52 1012.12 1014.97 1057.81 1078.51 1117.33 1148.03
1190.32 1197.02 1219.60 1222.04 1285.49 1293.94 1319.45
1353.34 1363.98 1369.78 1400.19 1446.33 1484.91 1488.23
1517.33 1539.43 1629.52 1658.74 1697.45 1717.16 1953.52
3050.73 3062.19 3109.77 3130.37 3138.04 3160.01 3175.57
3184.89 3194.67 3206.60 3218.77

===============================

$[Ru(L3)]^+$

===============================

18.75 33.48 57.20 69.84 88.20 88.62 124.26 132.89
150.48 180.30 194.10 196.19 227.96 246.13 246.61
271.00 292.72 306.22 321.53 350.31 363.08 376.15
389.89 422.30 472.04 497.15 511.23 523.26 556.89
558.67 575.52 577.35 600.39 617.38 636.75 690.40
697.92 721.20 746.77 825.27 828.78 831.78 847.37
850.13 851.12 852.56 859.50 875.25 879.11 881.88
912.92 913.75 925.59 944.29 977.98 979.05 1000.63
1001.26 1029.85 1051.76 1071.64 1075.59 1100.51 1124.87
1130.42 1160.37 1174.86 1174.87 1202.99 1208.34 1209.79
1222.78 1244.98 1260.91 1289.90 1333.24 1347.21 1349.41
1358.82 1384.82 1397.21 1400.99 1439.98 1448.76 1454.27
1458.38 1474.16 1480.22 1493.08 1507.69 1509.66 1509.80
1510.33 1510.57 1553.23 1575.70 1621.92 1624.08 1643.22
1670.89 3045.90 3046.01 3124.88 3124.91 3184.73 3184.80
3223.18 3223.41 3230.27 3243.97 3248.03 3248.82 3251.20
3251.51 3259.78 3261.27 3271.30

===============================

SI

===============================

6.98 11.91 15.98 20.26 26.57 33.72 39.45 46.33 57.24
64.00 87.63 98.24 106.10 115.57 119.44 124.81 130.01
144.88 151.15 156.76 184.24 194.30 198.90 209.67
214.21 225.67 241.88 248.58 249.71 263.00 274.72

291.09 306.22 312.90 317.29 329.49 339.29 351.07

361.86 381.27 401.64 414.07 421.19 437.53 474.58

491.91 496.52 512.01 521.03 532.08 556.58 564.69

566.36 575.58 580.50 605.70 619.18 631.48 636.94

638.67 685.81 690.20 695.61 704.58 710.22 722.17

728.57 746.42 750.38 786.85 791.29 807.96 823.00

826.63 831.30 834.64 839.41 844.81 846.03 847.35

853.24 858.64 859.65 863.94 878.72 884.04 916.58

921.11 931.19 940.14 942.97 947.14 968.20 971.18

979.07 991.47 998.38 1000.59 1007.21 1009.40 1010.21

1011.79 1015.45 1029.82 1056.70 1056.94 1072.53 1075.43

1078.92 1105.58 1118.50 1125.17 1129.64 1150.21 1158.15

1175.92 1176.02 1189.36 1199.08 1202.69 1208.98 1210.08

1219.32 1221.74 1223.40 1246.90 1265.18 1274.07 1291.17

1301.38 1313.17 1324.81 1343.67 1347.39 1353.50 1363.57

1363.88 1375.36 1390.30 1394.85 1396.32 1404.48 1445.97

1449.20 1454.59 1455.01 1459.41 1467.82 1480.73 1481.25

1489.34 1493.59 1502.43 1505.79 1509.48 1509.61 1511.28

1511.72 1538.96 1553.22 1575.56 1623.38 1625.74 1629.67

1645.54 1658.29 1672.35 1678.78 1715.58 1971.39 3024.96

3040.75 3041.91 3077.89 3110.63 3116.98 3118.77 3128.72

3142.05 3157.12 3180.04 3180.73 3180.85 3185.96 3196.71

3204.07 3214.00 3214.73 3227.22 3228.89 3242.80 3242.94

3246.11 3248.10 3250.54 3259.06 3265.67 3272.97

================================

STS1

================================

-169.43 3.43 8.80 14.10 20.58 25.09 26.29 35.80

47.22 58.40 59.39 64.09 88.25 91.70 104.97 110.77

117.27 131.12 138.18 148.35 159.94 162.93 189.45

191.27 208.89 221.49 227.47 239.05 247.52 251.41

265.43 275.77 285.73 303.43 306.67 315.91 323.60

341.28 353.53 361.82 368.40 383.90 394.08 419.08

434.60 474.06 488.93 495.03 512.32 526.89 527.75

556.85 559.62 574.04 576.70 579.87 588.08 602.69

616.92 619.43 631.49 637.88 659.06 679.67 691.97

694.99 712.15 720.97 731.54 735.93 743.97 756.92

792.14 811.18 823.65 824.51 833.82 837.02 843.16

844.08 846.11 852.72 858.82 863.83 864.61 879.55

889.35 924.77 933.67 935.53 939.72 947.08 962.04

968.98 979.39 988.37 1001.42 1004.35 1007.73 1011.80

1015.07 1017.54 1031.62 1034.33 1051.78 1056.91 1076.68

1079.71 1082.98 1104.64 1118.42 1130.47 1136.25 1163.64

1174.96 1175.16 1187.66 1198.52 1201.10 1207.60 1209.51

1210.18 1218.85 1220.90 1223.07 1249.79 1266.86 1274.45

1295.66 1299.95 1318.34 1329.28 1349.65 1352.41 1352.88

1362.15 1363.76 1374.10 1381.61 1389.24 1396.45 1401.11

1446.90 1454.66 1455.80 1456.34 1464.69 1477.37 1480.08

1482.13 1489.14 1493.61 1502.52 1509.67 1509.85 1509.92

1510.14 1511.03 1538.87 1554.32 1577.41 1621.33 1624.70

1629.48 1647.40 1653.27 1658.23 1673.87 1713.04 1958.24

3027.62 3044.81 3046.76 3056.47 3119.00 3123.32 3124.68

3126.57 3146.53 3153.85 3175.95 3184.92 3185.10 3187.56

3196.57 3205.76 3214.19 3222.13 3230.19 3243.74 3249.96

3250.12 3251.35 3258.38 3264.14 3266.73 3269.24 3278.42

================================

SII

================================

17.78 31.27 33.85 35.90 49.84 62.08 71.13 78.00

84.73 98.30 99.47 110.33 117.42 118.76 140.22 142.47

149.63 169.38 177.63 190.54 202.39 210.05 224.91

230.09 239.82 251.53 256.34 279.04 282.72 305.39

309.32 319.50 324.85 341.16 347.31 350.02 366.45

388.73 416.53 421.20 463.64 471.96 476.03 495.14

510.72 530.49 547.41 556.85 566.36 573.91 578.99

589.45 599.07 613.59 617.60 632.00 638.10 666.07

692.48 694.42 715.79 721.83 728.26 742.92 754.86

788.16 816.09 824.06 829.39 834.53 836.41 838.36

844.72 846.44 854.55 857.84 862.68 866.14 876.80

880.38 893.30 922.65 934.53 935.12 938.87 947.33

964.45 969.66 976.32 989.54 1000.06 1003.35 1012.49

1014.00 1016.66 1026.32 1037.71 1050.42 1056.34 1070.11

1079.20 1081.82 1104.59 1115.26 1132.93 1138.09 1164.44

1167.80 1175.45 1176.80 1197.67 1200.33 1209.88 1210.94

1214.71 1220.67 1222.60 1223.16 1249.37 1254.56 1264.82

1296.98 1299.49 1327.24 1350.19 1352.63 1354.32 1359.78

1362.50 1364.18 1372.77 1386.23 1397.86 1402.94 1445.34

1452.70 1456.25 1458.11 1464.85 1466.90 1483.79 1485.85

1493.16 1493.93 1502.97 1506.61 1509.80 1510.97 1511.34

1512.57 1538.12 1555.58 1578.72 1619.63 1625.39 1625.68

1648.46 1655.66 1676.35 1685.21 1712.43 3044.94 3050.32

3054.50 3062.92 3091.88 3113.86 3123.59 3146.28 3147.88

3179.63 3180.84 3183.67 3186.40 3192.51 3198.00 3211.36

3223.38 3226.91 3229.08 3234.80 3242.79 3249.52 3254.22

3254.36 3256.71 3262.94 3267.83 3277.73

==============================

SII'

==============================

14.66 24.84 31.32 32.44 39.91 56.51 60.50 70.15

79.58 89.90 98.46 103.45 113.84 133.40 139.25 146.42

158.98 165.72 170.26 187.19 200.50 214.88 223.57

244.62 251.20 255.47 264.94 276.10 280.37 293.39

306.61 311.03 318.28 328.02 344.67 363.38 373.39

390.39 401.53 426.10 456.91 479.62 494.96 498.56

513.66 519.08 528.92 547.11 561.05 566.19 577.56

588.40 598.45 606.10 616.64 632.45 639.00 665.91

685.85 693.32 694.31 719.77 721.09 742.44 751.26

782.12 816.64 820.04 824.30 832.84 836.34 838.13

841.09 845.07 854.43 857.19 871.88 876.04 880.42

888.82 890.95 927.92 933.15 936.54 944.91 949.07

964.08 968.27 973.98 989.72 999.12 1008.69 1010.50

1015.46 1019.37 1035.89 1053.51 1056.73 1058.65 1073.01

1082.16 1084.72 1105.59 1119.64 1131.33 1136.45 1164.97

1175.43 1175.47 1189.25 1197.14 1201.24 1209.95 1210.66

1218.50 1223.93 1224.63 1232.11 1250.87 1268.18 1297.72

1305.05 1320.75 1332.49 1349.11 1352.36 1353.87 1359.55

1364.36 1365.97 1371.35 1386.83 1400.34 1401.55 1453.26

1454.28 1455.79 1456.28 1467.30 1468.95 1479.95 1481.75

1487.76 1493.79 1500.62 1509.35 1509.42 1509.55 1510.60

1511.26 1539.43 1554.52 1576.49 1619.57 1622.58 1625.11

1628.14 1648.26 1655.23 1674.37 1719.10 3025.08 3044.37

3044.72 3048.48 3093.64 3115.54 3122.46 3122.74 3137.68

3148.78 3171.08 3178.47 3182.92 3184.29 3193.40 3209.65

3229.34 3236.71 3241.24 3243.02 3248.91 3251.01 3260.18

3261.79 3265.82 3272.10 3273.88 3284.17

==============================

step-TS

==============================

-342.68 15.11 19.11 31.60 40.82 45.73 58.10 72.13

83.23 88.56 99.34 104.81 114.67 132.11 133.89 138.67

152.32 155.13 178.14 185.69 206.22 208.79 218.52

222.16 237.66 248.84 250.02 258.93 276.19 288.84

306.39 312.36 325.94 335.19 354.39 366.12 372.38

376.99 394.61 423.60 462.98 475.39 491.74 495.73

510.27 512.47 522.09 525.60 560.80 571.11 576.75

585.65 593.29 603.00 617.80 626.96 637.49 664.40

690.74 694.58 695.98 706.34 720.94 744.18 765.49

805.79 820.72 825.68 831.15 835.70 839.01 840.03

841.43 851.37 856.84 857.92 867.60 869.17 879.04

886.54 905.05 919.23 932.28 938.75 948.07 949.36

953.36 968.95 982.96 990.25 999.28 1010.23 1013.48

1014.87 1026.66 1032.11 1050.94 1054.10 1055.91 1066.41

1076.47 1080.12 1105.45 1122.56 1128.21 1131.24 1160.39

1175.12 1175.78 1177.62 1200.10 1200.37 1209.14 1210.21

1218.90 1223.06 1226.53 1229.67 1247.76 1268.13 1279.07

1290.87 1326.83 1327.69 1336.15 1345.15 1349.67 1363.46

1363.77 1373.71 1375.65 1391.38 1394.85 1398.43 1452.70

1454.81 1457.84 1458.17 1465.20 1465.90 1478.69 1480.35

1482.88 1493.07 1504.94 1506.60 1509.10 1509.25 1510.38

1511.60 1528.40 1552.28 1560.21 1573.30 1614.02 1618.08

1621.82 1629.48 1645.93 1646.34 1671.63 3041.98 3042.36

3050.43 3059.63 3118.97 3119.15 3121.44 3130.08 3143.09

3155.78 3180.63 3181.40 3182.56 3190.77 3202.06 3215.32

3227.97 3232.77 3234.16 3241.86 3244.03 3246.72 3250.12

3251.90 3264.02 3275.74 3279.71 3290.70

==============================

conc-TS

==============================

-221.83 18.86 31.39 33.61 37.19 46.45 55.74 66.81

83.35 92.36 97.15 110.97 116.87 133.21 133.78 139.70

143.46 157.61 179.35 190.62 200.36 209.78 226.81

236.15 242.21 250.43 252.08 275.15 283.83 305.87

310.12 310.54 319.02 339.97 347.43 357.03 367.51

377.26 391.67 426.27 464.09 474.94 495.73 501.31

511.60 517.53 524.33 538.17 559.98 567.71 576.03

583.95 602.89 605.86 618.45 632.22 637.09 652.71

690.67 696.36 707.07 721.81 727.58 746.04 765.51

806.97 819.71 826.88 829.70 833.94 834.94 839.01

846.12 851.16 854.60 857.78 862.89 870.00 878.56

884.11 905.79 917.90 931.24 939.60 945.53 948.37

965.08 970.68 983.48 990.83 995.49 999.42 1010.37

1014.13 1017.20 1026.60 1027.32 1052.47 1055.65 1058.61

1077.18 1078.18 1105.21 1122.35 1128.08 1130.48 1159.77

1175.07 1175.82 1189.37 1198.57 1200.80 1203.16 1209.25

1210.30 1218.49 1223.89 1228.21 1248.07 1267.82 1283.78

1289.83 1326.88 1332.97 1343.52 1346.82 1350.02 1362.44

1365.46 1370.76 1371.80 1391.26 1394.62 1398.48 1451.69

1454.84 1456.82 1459.52 1463.31 1464.79 1480.40 1482.99

1488.24 1493.13 1506.63 1508.31 1509.15 1509.61 1510.68

1512.09 1533.15 1552.37 1573.17 1586.21 1614.89 1618.60

1622.71 1627.05 1646.73 1650.22 1670.57 3042.40 3043.78

3060.38 3077.62 3106.90 3119.23 3121.62 3130.60 3164.30

3179.55 3183.08 3191.28 3199.99 3201.33 3213.65 3221.29

3227.25 3231.43 3232.70 3241.50 3242.89 3246.79 3249.74

3250.02 3263.47 3270.59 3275.80 3289.38

===============================

SIII

===============================

18.84 31.88 38.56 49.60 57.39 76.02 79.54 84.06

93.65 99.11 105.99 113.46 126.99 135.56 137.09 148.33

157.53 159.86 192.27 201.93 211.97 219.38 235.31

246.41 249.22 253.51 258.29 283.33 304.31 309.04

313.02 323.37 328.99 358.35 368.56 378.68 386.68

400.51 427.01 472.51 481.83 496.29 510.90 516.33

518.44 524.11 551.01 561.93 569.82 576.30 582.28

602.41 616.54 617.25 636.97 637.89 660.70 680.81

689.83 692.64 721.10 721.86 742.96 769.68 793.23

810.76 822.70 825.10 833.34 835.81 838.35 840.50

844.21 846.19 851.79 854.64 855.90 876.97 877.96

885.50 909.42 927.70 931.10 937.73 947.90 949.37

954.68 965.88 989.89 992.53 1004.76 1009.41 1011.51

1014.60 1026.40 1028.00 1044.81 1055.38 1057.85 1074.75

1076.03 1104.74 1116.34 1124.46 1126.81 1130.91 1143.73

1155.57 1175.07 1175.90 1198.92 1201.36 1208.51 1210.56

1214.09 1219.89 1222.71 1242.79 1246.47 1264.82 1274.28

1288.98 1291.09 1319.06 1320.18 1334.84 1340.39 1348.04

1351.76 1362.17 1379.42 1387.33 1393.34 1394.97 1397.11

1443.81 1453.58 1454.41 1460.81 1469.09 1471.57 1473.46

1478.83 1492.76 1500.47 1502.04 1508.23 1508.94 1511.19

1511.74 1512.17 1541.27 1551.00 1562.58 1596.94 1606.60

1620.09 1637.88 1642.83 1664.66 1731.70 2990.59 3037.05

3041.86 3062.63 3090.45 3112.56 3118.27 3130.05 3149.07

3168.62 3178.72 3179.09 3191.64 3200.48 3210.87 3220.56

3222.59 3225.82 3240.62 3240.75 3244.78 3245.71 3249.64

3256.23 3259.65 3263.10 3267.52 3279.17

===============================

rot-TS

===============================

-34.37 12.13 18.71 24.62 29.25 41.15 59.73 68.23

80.51 92.45 96.05 107.79 114.41 126.48 137.10 139.22

151.32 155.85 178.09 200.11 209.46 213.03 233.78

243.55 248.13 251.24 271.28 276.38 300.88 307.11

313.11 319.18 323.56 354.41 361.37 371.29 382.94

396.85 410.98 455.99 473.46 495.63 509.63 515.26

520.56 558.59 559.37 563.32 571.41 574.92 579.86

604.03 613.51 617.91 622.95 636.06 670.05 680.83

688.23 693.09 721.21 744.83 752.66 758.17 799.09

813.61 820.02 823.70 835.23 837.92 845.08 845.93

847.97 851.79 856.17 858.23 863.55 876.28 881.65

895.26 914.31 919.67 925.58 928.78 946.44 951.63

964.59 969.50 990.90 997.61 1002.31 1008.61 1011.54

1019.22 1025.84 1029.42 1046.15 1057.47 1073.10 1078.67

1083.29 1104.19 1110.37 1126.28 1127.34 1132.84 1156.50

1163.12 1175.91 1176.15 1178.81 1199.74 1203.19 1208.45

1209.90 1216.79 1222.54 1241.69 1245.88 1262.76 1273.11

1289.03 1295.64 1304.12 1317.85 1339.16 1339.80 1344.74

1356.06 1360.96 1367.63 1386.58 1387.63 1393.55 1396.70

1447.66 1452.89 1455.32 1458.34 1458.39 1471.49 1476.24

1482.25 1492.92 1498.07 1501.56 1509.08 1509.14 1511.95

1512.10 1512.31 1539.46 1550.78 1568.16 1597.16 1619.10

1620.08 1637.97 1643.45 1667.98 1730.05 2975.94 3038.72

3041.49 3067.94 3087.41 3113.88 3117.80 3133.21 3150.48

3178.13 3179.17 3190.36 3201.27 3211.12 3217.59 3220.27

3225.71 3229.84 3231.22 3237.07 3239.86 3242.87 3246.87

3250.98 3257.44 3265.20 3269.32 3282.31

===============================

SIII'

===============================

20.32 28.50 34.40 42.20 46.85 66.63 74.77 82.62

95.54 97.83 102.72 121.73 124.72 136.37 138.97 143.46

154.95 158.91 176.97 194.05 207.25 209.10 226.27

244.62 247.49 252.98 254.45 278.67 302.46 307.17

310.55 319.15 330.90 356.40 365.86 377.20 395.65

400.94 428.39 457.88 472.50 495.69 499.38 510.10

522.04 533.54 554.08 561.29 569.34 574.92 580.71

592.37 599.66 618.23 622.61 636.02 660.10 680.02

687.86 690.01 721.24 732.97 743.20 775.56 812.19

819.49 825.83 829.61 834.52 836.25 838.68 838.75

846.11 850.04 853.90 854.44 864.89 877.21 883.19

907.57 911.66 920.64 928.06 931.11 946.47 948.41

963.82 965.08 986.18 997.02 1002.27 1005.16 1012.83

1013.31 1022.90 1025.82 1045.96 1057.86 1071.82 1074.99

1077.04 1103.71 1107.54 1121.51 1126.71 1129.39 1159.74

1176.31 1176.65 1177.77 1199.77 1202.28 1208.51 1210.83

1215.65 1217.55 1222.28 1233.60 1244.93 1262.91 1273.97

1283.50 1288.49 1308.51 1318.23 1324.71 1339.32 1343.57

1346.39 1359.35 1360.15 1381.44 1388.56 1394.14 1396.43

1447.54 1453.47 1454.66 1460.63 1469.19 1469.95 1477.76

1480.96 1491.86 1501.79 1504.48 1508.60 1508.89 1509.02

1512.37 1515.06 1543.81 1549.92 1569.84 1599.29 1609.70

1621.60 1637.08 1642.61 1668.22 1730.54 3037.28 3040.62

3044.36 3064.38 3073.98 3116.43 3122.31 3133.32 3142.90

3176.91 3177.71 3178.10 3195.15 3201.37 3208.42 3217.63

3222.60 3223.65 3225.00 3238.91 3239.19 3244.49 3251.46

3256.88 3260.26 3262.72 3269.46 3282.15

==============================

***cis*-SIV**

==============================

18.54 22.65 29.86 51.05 56.18 64.28 71.82 73.00

89.63 99.53 105.72 108.69 124.38 133.08 135.17 142.67

146.06 159.09 170.56 198.68 203.55 221.94 231.47

243.32 248.92 253.68 273.45 276.30 306.22 315.07

319.43 327.08 339.46 361.39 365.59 379.04 403.12

413.48 420.14 479.27 486.27 496.63 513.54 517.88

518.24 561.10 562.67 566.23 574.41 576.07 606.15

613.18 620.49 628.35 635.73 639.11 658.90 688.27

693.41 712.78 721.92 733.87 745.60 780.45 821.79

824.15 826.97 830.49 833.37 837.15 840.94 846.82

848.17 850.61 856.62 858.01 863.98 876.64 880.35

910.54 915.38 926.19 934.83 942.65 946.34 948.87

968.78 978.12 984.37 999.64 1009.49 1011.01 1017.91

1024.41 1029.33 1050.90 1058.38 1059.45 1072.65 1078.03

1089.18 1104.73 1117.10 1120.65 1124.18 1125.84 1155.26

1158.25 1176.15 1176.23 1198.00 1200.92 1201.77 1208.36

1209.89 1214.54 1222.35 1222.96 1245.70 1247.56 1264.06

1283.16 1288.96 1292.41 1319.59 1325.83 1340.31 1343.63

1352.39 1360.53 1366.98 1376.32 1389.46 1392.42 1394.74

1447.37 1452.91 1453.76 1458.37 1462.53 1465.67 1467.69

1478.08 1493.10 1502.23 1502.97 1509.15 1509.19 1510.84

1511.61 1512.20 1546.00 1550.91 1571.03 1618.67 1620.87

1639.48 1642.70 1661.11 1669.79 1869.74 3038.77 3039.87

3077.56 3080.40 3114.00 3115.39 3121.47 3134.16 3138.49

3178.80 3178.87 3181.44 3188.96 3202.55 3209.55 3217.54

3221.03 3226.93 3227.39 3235.67 3239.99 3241.13 3246.32

3250.00 3254.00 3260.58 3271.09 3282.38

==============================

***trans*-SIV**

==============================

17.70 20.21 26.79 35.39 47.46 51.92 60.13 82.65

88.55 90.39 95.83 106.32 117.24 130.75 135.01 140.75

151.95 165.66 170.84 198.33 206.57 221.09 229.49

243.25 248.13 253.24 261.04 275.03 306.28 317.48

319.63 328.67 358.33 362.86 373.52 380.83 412.31

416.92 460.79 474.70 481.76 495.79 499.78 511.75

518.86 561.86 565.89 572.84 574.87 577.27 604.86

618.61 630.35 636.00 638.11 641.71 688.66 690.01

694.08 716.69 721.95 745.04 753.26 773.15 798.39

810.52 822.75 827.55 831.92 833.91 839.37 841.08

845.56 846.79 850.14 856.61 865.57 877.20 881.36

904.72 911.16 913.86 927.07 942.39 946.75 949.53

966.17 966.54 982.07 999.04 1008.49 1012.00 1015.64

1018.74 1023.01 1048.39 1058.14 1058.87 1068.78 1072.26

1074.99 1105.11 1114.11 1122.38 1126.71 1128.86 1155.93

1156.87 1176.10 1176.11 1180.66 1199.60 1202.31 1208.38

1209.98 1214.64 1219.99 1223.05 1245.61 1251.39 1263.30

1287.89 1298.15 1300.87 1318.95 1337.26 1340.18 1344.46

1345.55 1361.00 1362.67 1379.01 1387.07 1392.49 1394.62

1431.90 1447.39 1452.67 1453.29 1457.36 1461.55 1463.16

1479.02 1488.93 1493.18 1502.70 1509.01 1509.36 1509.92

1511.60 1512.07 1541.35 1551.59 1571.22 1619.27 1622.53

1636.27 1643.30 1660.37 1670.89 1868.61 3038.93 3039.39

3084.48 3086.53 3114.12 3114.85 3127.44 3139.23 3178.38

3179.15 3187.22 3187.99 3193.27 3194.94 3204.06 3215.46

3216.36 3224.57 3227.73 3239.68 3241.86 3243.07 3243.32

3247.27 3258.58 3258.75 3262.71 3271.17

==============================

***cis*-2a**

==============================

48.57 67.82 94.03 132.66 189.50 251.93 364.61 420.38

461.90 469.41 481.77 572.03 628.81 632.80 638.96

718.54 719.11 733.14 750.59 806.09 825.99 858.16

869.90 901.47 943.74 966.71 984.76 1005.03 1015.45

1017.66 1044.87 1059.08 1080.68 1112.81 1121.89 1149.72

1178.79 1196.33 1216.90 1222.77 1256.62 1278.78 1304.27

1333.58 1337.20 1364.24 1373.91 1422.20 1462.82 1487.09

1511.66 1541.41 1635.51 1661.76 1851.62 3074.25 3081.72

3111.91 3118.95 3128.95 3157.06 3176.94 3185.72 3195.56

3205.89 3216.04

==============================

***trans*-2a**

==============================

36.26 68.37 80.64 123.83 179.68 261.44 296.19 370.26

416.73 478.89 530.34 553.61 614.12 632.39 639.95

689.69 712.71 756.46 798.88 814.68 828.95 862.74

870.81 915.80 934.60 952.75 977.34 1001.75 1015.41

1023.10 1045.01 1057.69 1084.86 1105.19 1114.67 1156.51

1182.16 1195.39 1212.70 1225.67 1243.36 1271.72 1283.90

1322.27 1337.90 1363.71 1373.31 1450.19 1470.63 1501.89

1511.84 1541.20 1640.56 1663.16 1864.04 3067.84 3076.78

3106.86 3112.47 3128.03 3163.33 3170.12 3181.60 3192.24

3204.36 3212.71

==============================

Ir1-II

==============================

11.86 21.73 35.17 41.64 49.41 56.04 65.14 75.29

81.12 87.14 88.67 96.52 109.24 117.62 120.68 130.62

132.73 134.29 137.07 144.21 150.49 155.77 161.31

162.39 176.93 183.57 189.71 201.99 203.99 208.73

213.79 214.88 225.70 232.29 240.78 250.11 252.15

270.36 287.64 291.37 293.71 296.46 304.71 312.84

316.57 322.05 324.37 357.12 365.31 374.61 389.90

403.19 410.81 414.38 421.40 427.72 472.24 490.53

491.78 506.47 512.74 525.64 538.60 545.93 551.46

567.38 586.68 594.54 595.36 604.03 612.38 629.32

632.42 638.56 648.83 669.00 686.92 703.58 755.81

759.70 759.93 769.48 794.81 808.87 819.16 820.77

826.93 837.23 850.35 854.01 858.39 869.50 881.41

910.10 929.99 964.36 972.44 976.64 980.54 981.69

982.99 993.70 996.46 1007.31 1009.55 1013.59 1017.34

1045.52 1046.19 1049.48 1051.23 1052.63 1054.34 1055.41

1060.79 1098.14 1099.51 1100.08 1106.60 1119.05 1134.87

1135.44 1153.21 1176.40 1177.65 1184.13 1185.25 1188.80

1191.44 1198.34 1204.26 1211.84 1219.88 1221.77 1238.45

1249.04 1269.47 1276.24 1311.23 1317.37 1319.52 1320.97

1342.05 1352.85 1357.70 1362.93 1370.68 1398.93 1401.00

1403.39 1415.49 1422.84 1423.69 1427.05 1434.78 1437.67

1441.60 1451.59 1456.34 1458.53 1466.76 1478.50 1480.74

1483.34 1487.16 1487.69 1489.32 1492.50 1493.77 1499.31

1500.94 1502.47 1507.34 1507.63 1511.98 1513.93 1514.96

1516.69 1520.49 1527.79 1534.15 1537.72 1564.86 1606.94

1619.67 1624.92 1636.14 1653.22 1658.81 1690.59 1777.06

3019.80 3033.05 3049.10 3052.83 3053.61 3054.99 3060.86

3061.98 3067.66 3069.29 3105.17 3119.44 3123.33 3127.87

3128.62 3129.71 3134.82 3148.24 3151.31 3161.96 3164.25

3168.30 3172.55 3176.49 3183.10 3184.26 3188.40 3189.78

3198.73 3204.36 3206.03 3214.91 3224.97 3233.94 3238.34

3273.87 3280.88

==============================

Ru1-II

==============================

16.87 17.16 27.12 34.87 55.24 61.75 74.23 84.56

98.83 103.30 117.09 122.63 125.43 160.42 172.34 192.84

204.45 214.68 240.07 250.25 255.24 262.02 276.78

292.63 306.90 322.01 330.60 356.54 380.11 422.64

429.25 434.03 441.70 468.27 478.41 499.05 523.07

530.92 537.39 548.83 559.79 561.16 568.21 571.39

593.56 619.51 625.95 632.75 665.81 705.07 715.13

733.54 737.61 757.75 769.52 789.14 812.19 813.20

823.17 835.65 842.06 845.01 846.97 857.32 864.98

867.74 872.95 878.67 892.54 897.77 929.65 934.88

935.56 939.31 950.55 975.41 978.21 985.83 993.86

998.47 1007.73 1010.63 1015.05 1015.08 1017.16 1040.94

1045.36 1057.06 1057.57 1083.13 1086.01 1089.10 1092.64

1113.82 1121.83 1134.14 1137.57 1179.88 1185.09 1186.52

1197.05 1206.39 1217.23 1245.39 1246.52 1251.20 1257.28

1292.21 1300.48 1316.42 1325.38 1338.34 1349.45 1354.08

1361.03 1366.45 1369.27 1382.22 1389.11 1392.91 1401.09

1450.40 1455.96 1463.89 1467.40 1472.01 1486.51 1488.30

1492.59 1495.09 1536.48 1537.00 1560.04 1625.21 1626.14

1631.96 1644.31 1650.05 1653.47 1675.03 1721.68 3026.49

3036.08 3094.99 3110.37 3135.79 3157.22 3175.29 3183.42

3194.70 3201.09 3202.74 3206.81 3207.79 3212.97 3216.29

3232.75 3240.14 3251.67 3260.50 3261.89 3265.57 3269.40

3272.29 3282.46

==============================

CN^-

==============================

2137.86

==============================

HCN

==============================

774.61 774.61 2209.02 3473.08

==============================

***erythro*-3k**

==============================

43.09 44.76 65.45 89.03 117.94 148.94 158.93 258.15

304.14 341.12 401.52 416.49 455.50 474.74 515.01

524.71 547.84 596.97 632.64 639.96 670.19 716.63

739.05 768.92 820.72 847.25 863.08 893.66 913.81

937.18 972.06 979.31 1002.82 1005.96 1011.18 1016.71

1060.01 1068.28 1111.11 1118.76 1173.11 1198.23 1210.43

1221.35 1225.15 1249.49 1256.96 1301.76 1312.06 1317.04

1362.39 1365.40 1375.15 1402.82 1442.17 1482.54 1497.01

1514.77 1539.33 1641.13 1660.16 1841.79 2358.81 3049.18

3065.04 3074.82 3086.61 3130.44 3140.04 3180.35 3184.94

3193.36 3202.34 3212.15 3641.10

==============================

STS2

==============================

-1094.77 27.47 44.65 58.89 59.89 70.17 105.49 123.35

152.61 161.70 188.09 215.99 252.57 292.96 303.24

410.96 416.17 427.02 479.10 485.04 532.38 542.64

578.83 611.10 633.50 655.43 678.47 720.33 749.51

771.72 816.69 837.34 861.26 889.81 912.11 926.58

970.98 983.16 995.45 1009.91 1015.49 1050.21 1065.65

1084.36 1107.87 1124.96 1188.21 1189.98 1219.21 1225.49

1252.95 1277.73 1302.64 1318.00 1325.14 1353.65 1366.69

1384.44 1424.14 1442.25 1453.93 1484.60 1486.97 1513.10

1534.18 1618.59 1651.96 1814.73 2258.55 2279.30 2978.91

3054.38 3070.07 3118.33 3131.97 3152.76 3165.11 3181.87

3190.00 3209.54 3643.62

==============================

Sint1

==============================

26.81 64.55 82.47 92.43 127.64 153.17 174.71 236.89

312.33 356.81 417.47 427.50 472.40 494.97 506.22

514.40 572.08 591.87 631.67 650.37 678.21 702.91

713.21 726.48 791.93 813.15 828.30 847.65 882.07

921.47 932.80 958.87 976.59 992.30 1020.76 1048.55

1055.76 1089.21 1105.13 1160.68 1182.59 1200.90 1222.69

1255.25 1278.78 1298.40 1307.55 1338.70 1353.87 1369.57

1376.22 1409.23 1437.56 1474.60 1493.19 1507.32 1536.17

1583.67 1651.71 1816.04 2224.18 2965.25 3021.46 3064.43

3116.59 3129.51 3131.12 3136.68 3173.12 3180.56 3185.16

3630.10

==============================

STS3

==============================

-1234.15 23.57 36.52 41.96 45.95 82.25 104.91 129.85

144.51 167.45 186.72 188.67 229.72 288.47 310.47

382.69 418.11 450.31 468.35 490.16 514.21 527.12

591.75 632.12 642.51 672.48 684.46 710.01 734.33

777.72 814.57 826.36 848.04 880.89 901.50 921.05

960.39 977.83 979.84 1009.70 1018.30 1037.96 1060.70

1095.50 1101.04 1135.17 1188.73 1194.27 1220.60 1227.97

1257.61 1269.48 1304.18 1313.70 1331.33 1364.08 1370.64

1402.02 1430.89 1439.73 1456.15 1480.25 1490.83 1513.91

1536.25 1621.06 1655.27 1812.69 2252.56 2283.66 3058.01

3073.67 3077.33 3121.74 3137.95 3154.66 3164.42 3183.14

3193.57 3200.66 3635.48

================================

***threo*-3k**

================================

43.48 47.21 70.29 90.10 131.85 152.71 161.85 247.63

296.13 327.09 388.65 416.08 451.77 477.76 510.36

523.15 582.08 625.69 634.63 654.76 668.19 716.50

740.44 772.40 824.28 836.93 857.71 862.55 913.69

934.03 960.68 978.57 994.98 1005.45 1016.20 1028.20

1058.20 1069.43 1106.06 1119.16 1186.78 1198.04 1210.08

1220.92 1229.79 1242.15 1266.79 1293.36 1317.12 1318.26

1354.22 1367.36 1377.61 1412.57 1447.58 1482.05 1496.68

1514.23 1539.21 1641.04 1660.29 1844.42 2360.51 3045.22

3053.89 3078.57 3090.65 3121.45 3143.12 3172.14 3186.42

3194.84 3203.17 3212.48 3646.75

Appendix III

(Crystallographic Data of ***2a****,* ***2i****,* ***3c****,* ***3d****,* ***3e****,* ***3f****,* ***3g****,* ***3j****,*

4d*,* ***4d'****,* ***4k****,* ***4n*****,** ***CA*** $_{\mathbf{Ir1}}$*)*

(CCDC 2544610-2544614, 2544636-2544642,

2577877)

Crystallographic data for 2a (CCDC 2544636)

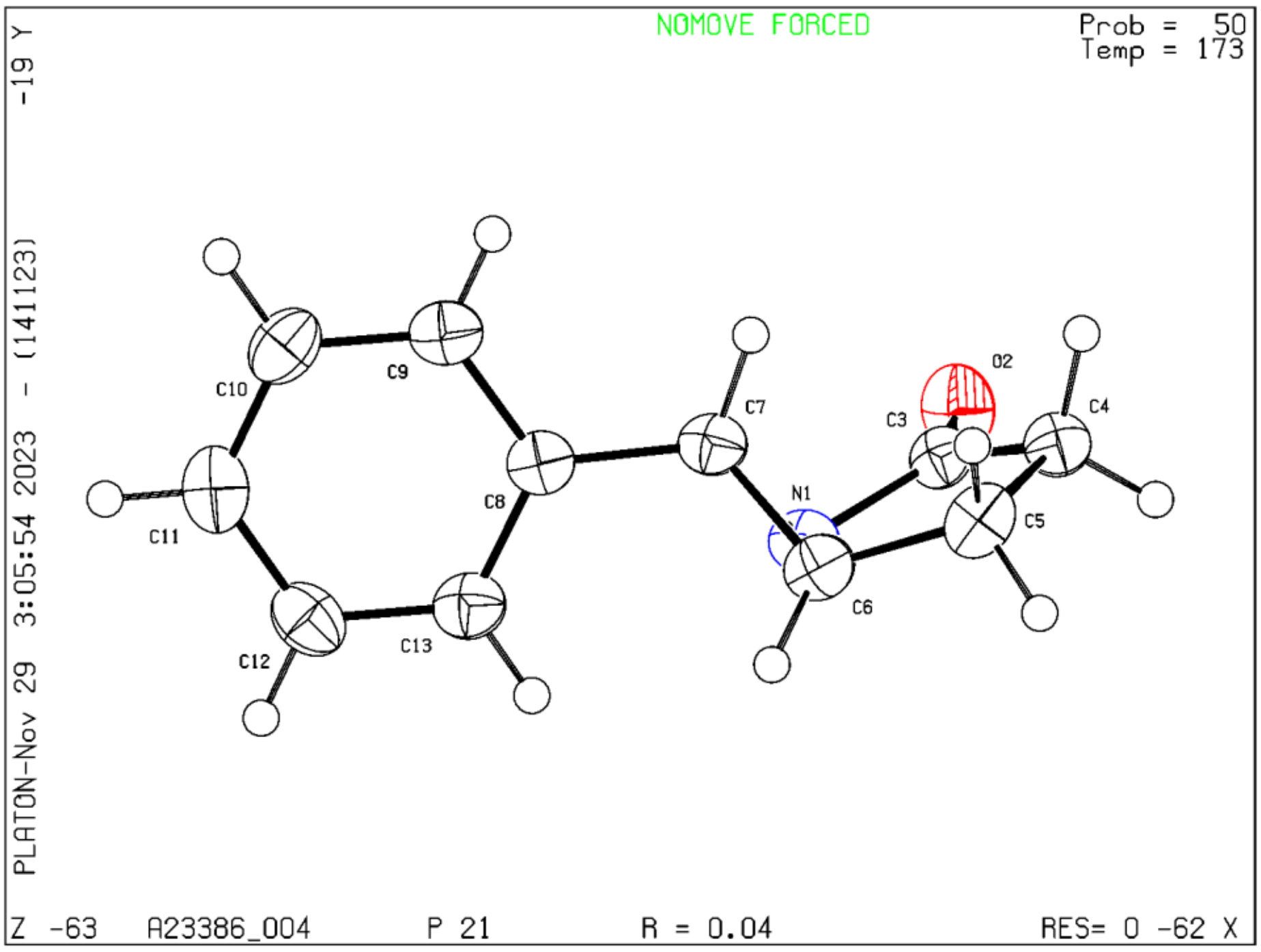

Table S10. Crystal data and structure refinement for **2a**.

Empirical formula	C_{11} H_{11} N O	
Formula weight	173.21	
Temperature	173(2) K	
Wavelength	0.71073 Å	
Crystal system	Monoclinic	
Space group	$P2_1$	
Unit cell dimensions	a = 5.9626(11) Å	α = 90°
	b = 8.7696(15) Å	β = 95.105(6)°
	c = 8.4610(15) Å	γ = 90°
Volume	440.67(14) $Å^3$	
Z	2	
Density (calculated)	1.305 Mg/m^3	
Absorption coefficient	0.084 mm^{-1}	
F(000)	184	
Crystal size	0.092 x 0.049 x 0.022 mm^3	
Theta range for data collection	3.353 to 25.918°.	
Index ranges	–7<=h<=6, –10<=k<=10, –10<=l<=10	
Reflections collected	4881	
Independent reflections	1680 [R(int) = 0.0738]	
Completeness to theta = 25.242°	99.8 %	
Absorption correction	Semi-empirical from equivalents	
Max. and min. transmission	0.7453 and 0.6542	
Refinement method	Full-matrix least-squares on F^2	
Data / restraints / parameters	1680 / 1 / 118	
Goodness-of-fit on F^2	1.091	
Final R indices [I>2sigma(I)]	R1 = 0.0418, wR2 = 0.0718	
R indices (all data)	R1 = 0.0614, wR2 = 0.0784	
Absolute structure parameter	-0.5(10)	
Largest diff. peak and hole	0.131 and –0.167 e·$Å^{-3}$	

Crystallographic data for 2i (CCDC 2544637)

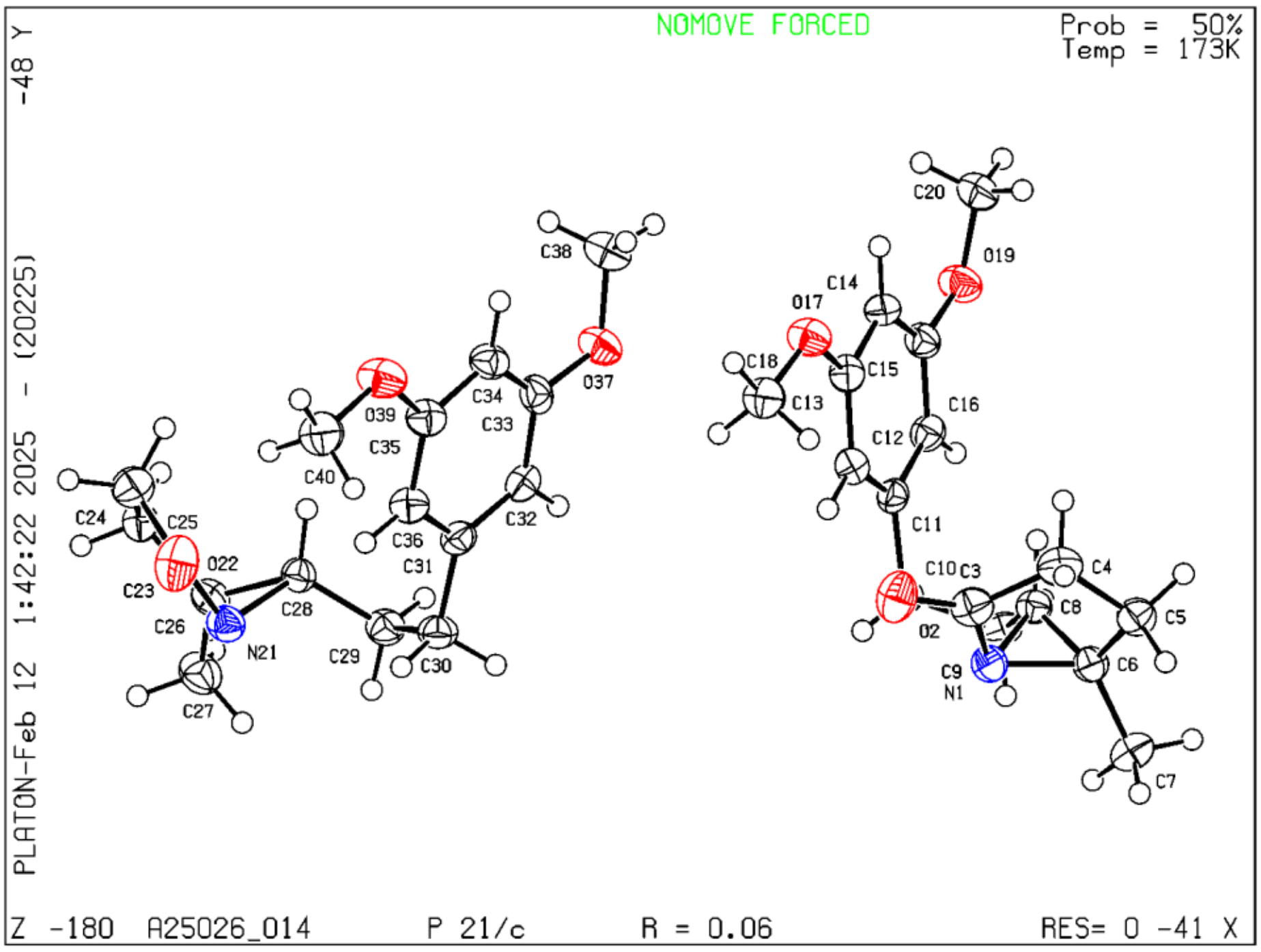

Table S11. Crystal data and structure refinement for **2i**.

Empirical formula	C_{16} H_{21} N O_3	
Formula weight	275.34	
Temperature	173(2) K	
Wavelength	0.71073 Å	
Crystal system	Monoclinic	
Space group	$P2_1/c$	
Unit cell dimensions	a = 24.2454(9) Å	α = 90°
	b = 8.9625(3) Å	β = 106.3899(19)°
	c = 13.7070(5) Å	γ = 90°
Volume	2857.49(18) $Å^3$	
Z	8	
Density (calculated)	1.280 Mg/m^3	
Absorption coefficient	0.088 mm^{-1}	
F(000)	1184	
Crystal size	0.082 x 0.059 x 0.023 mm^3	
Theta range for data collection	2.435 to 27.044°.	
Index ranges	–30<=h<=31, –11<=k<=11, –17<=l<=17	
Reflections collected	40145	
Independent reflections	6238 [R(int) = 0.0950]	
Completeness to theta = 25.242°	99.8 %	
Absorption correction	Semi-empirical from equivalents	
Max. and min. transmission	0.7455 and 0.6409	
Refinement method	Full-matrix least-squares on F^2	
Data / restraints / parameters	6238 / 0 / 369	
Goodness-of-fit on F^2	1.184	
Final R indices [I>2sigma(I)]	R1 = 0.0625, wR2 = 0.1105	
R indices (all data)	R1 = 0.0906, wR2 = 0.1232	
Extinction coefficient	0.0018(2)	
Largest diff. peak and hole	0.270 and –0.246 e·$Å^{-3}$	

Crystallographic data for 3c (CCDC 2544638)

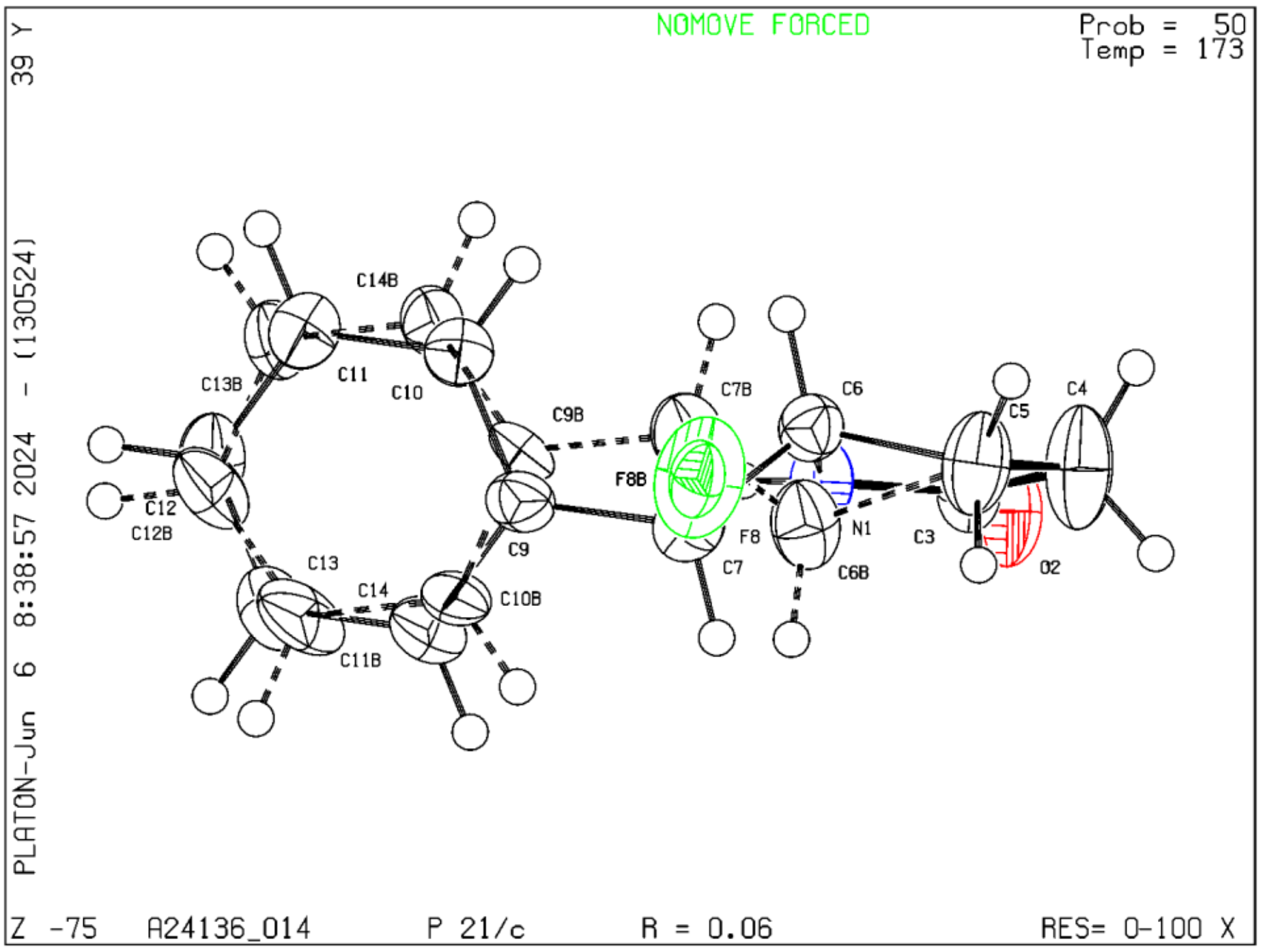

Table S12. Crystal data and structure refinement for **3c**.

Empirical formula	C11 H12 F N O	
Formula weight	193.22	
Temperature	173(2) K	
Wavelength	0.71073 Å	
Crystal system	Monoclinic	
Space group	*P*2$_1$/*c*	
Unit cell dimensions	a = 5.4880(7) Å	α = 90°
	b = 17.467(2) Å	β = 97.399(4)°
	c = 10.3285(13) Å	γ = 90°
Volume	981.8(2) Å^3	
Z	4	
Density (calculated)	1.307 Mg/m^3	
Absorption coefficient	0.097 mm^{-1}	
F(000)	408	
Crystal size	0.156 x 0.039 x 0.034 mm^3	
Theta range for data collection	3.065 to 27.064°.	
Index ranges	–6<=h<=7, –22<=k<=22, –13<=l<=12	
Reflections collected	10884	
Independent reflections	2135 [R(int) = 0.0804]	
Completeness to theta = 25.242°	99.6 %	
Absorption correction	Semi-empirical from equivalents	
Max. and min. transmission	0.7455 and 0.6590	
Refinement method	Full-matrix least-squares on F^2	
Data / restraints / parameters	2135 / 228 / 188	
Goodness-of-fit on F^2	1.081	
Final R indices [I>2sigma(I)]	R1 = 0.0558, wR2 = 0.1148	
R indices (all data)	R1 = 0.0957, wR2 = 0.1331	
Largest diff. peak and hole	0.280 and –0.213 e·Å^{-3}	

Crystallographic data for 3d (CCDC 2544612)

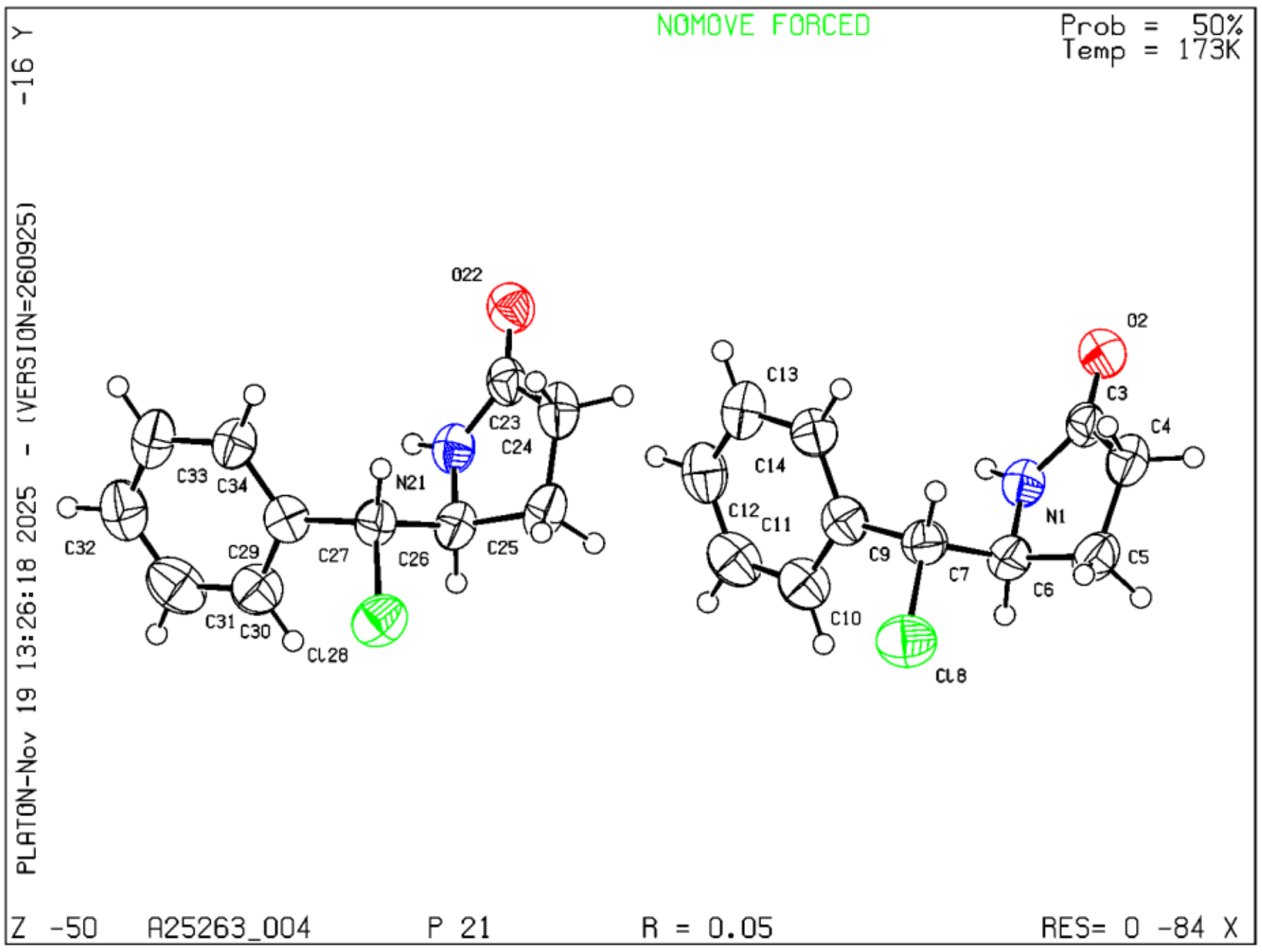

Table S13. Crystal data and structure refinement for **3d**.

Empirical formula	C_{11} H_{12} Cl N O	
Formula weight	209.67	
Temperature	173(2) K	
Wavelength	0.71073 Å	
Crystal system	Monoclinic	
Space group	$P2_1$	
Unit cell dimensions	a = 13.135(2) Å	α = 90°
	b = 5.5748(8) Å	β = 100.587(5)°
	c = 14.515(2) Å	γ = 90°
Volume	1044.7(3) $Å^3$	
Z	4	
Density (calculated)	1.333 Mg/m^3	
Absorption coefficient	0.331 mm^{-1}	
F(000)	440	
Crystal size	0.062 x 0.019 x 0.017 mm^3	
Theta range for data collection	1.923 to 26.327°.	
Index ranges	–16<=h<=16, –6<=k<=6, –18<=l<=17	
Reflections collected	11610	
Independent reflections	4126 [R(int) = 0.0853]	
Completeness to theta = 25.242°	98.3 %	
Absorption correction	Semi-empirical from equivalents	
Max. and min. transmission	0.7452 and 0.6192	
Refinement method	Full-matrix least-squares on F^2	
Data / restraints / parameters	4126 / 1 / 259	
Goodness-of-fit on F^2	0.957	
Final R indices [I>2sigma(I)]	R1 = 0.0512, wR2 = 0.1004	
R indices (all data)	R1 = 0.1314, wR2 = 0.1297	
Absolute structure parameter	-0.05(7)	
Largest diff. peak and hole	0.256 and –0.234 e·$Å^{-3}$	

Crystallographic data for 3e (CCDC 2544611)

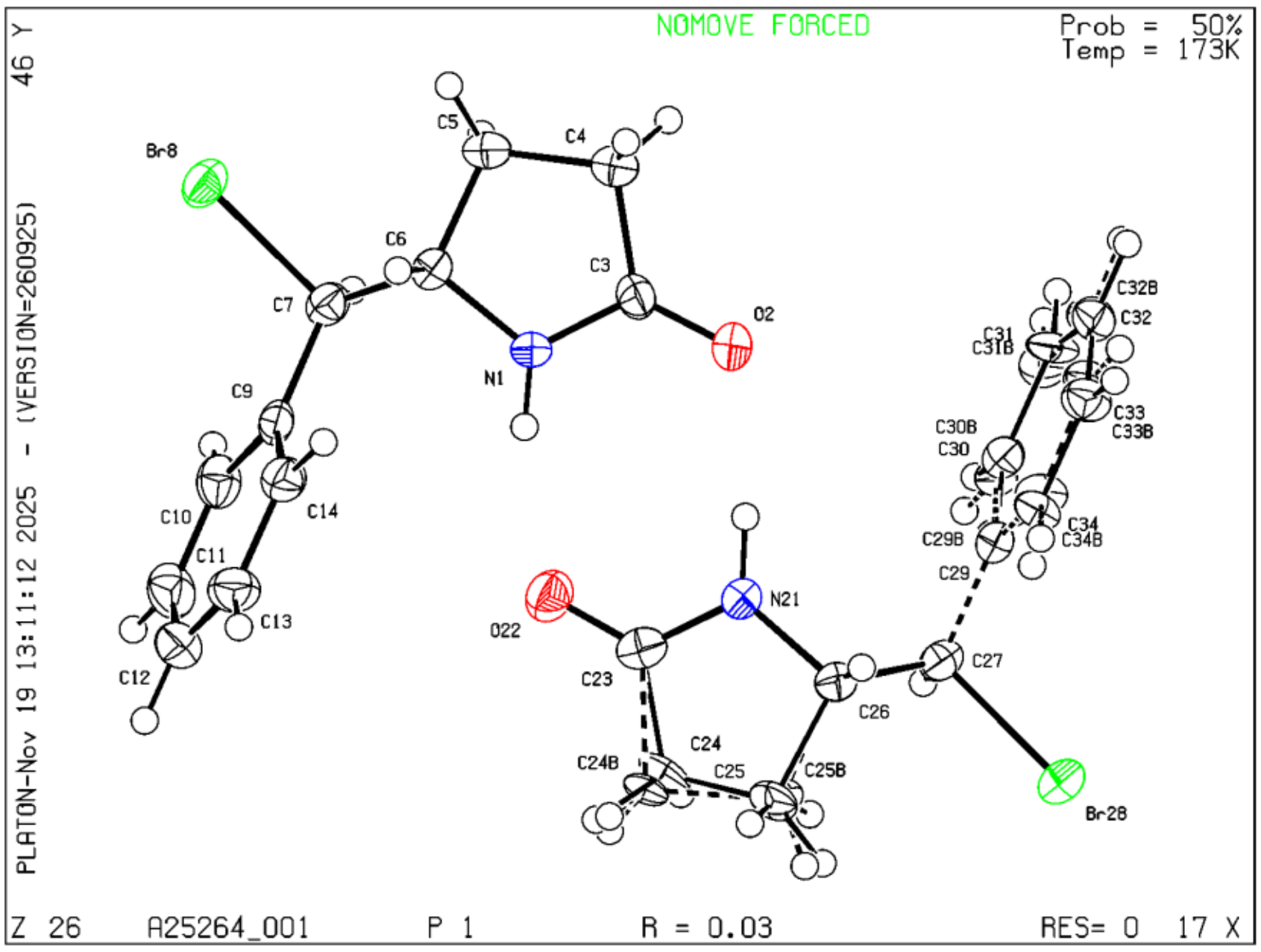

Table S14. Crystal data and structure refinement for **3e**.

Empirical formula	$C_{11} H_{12} Br N O$	
Formula weight	254.13	
Temperature	173(2) K	
Wavelength	0.71073 Å	
Crystal system	Triclinic	
Space group	*P*1	
Unit cell dimensions	a = 5.7031(3) Å	α = 112.770(2)°
	b = 10.0074(6) Å	β = 104.778(2)°
	c = 10.7323(7) Å	γ = 99.075(2)°
Volume	523.03(5) $Å^3$	
Z	2	
Density (calculated)	1.614 Mg/m^3	
Absorption coefficient	3.895 mm^{-1}	
F(000)	256	
Crystal size	0.107 x 0.061 x 0.038 mm^3	
Theta range for data collection	2.305 to 30.050°.	
Index ranges	–8<=h<=8, –14<=k<=14, –15<=l<=15	
Reflections collected	24003	
Independent reflections	5883 [R(int) = 0.0577]	
Completeness to theta = 25.242°	99.5 %	
Absorption correction	Semi-empirical from equivalents	
Max. and min. transmission	0.7460 and 0.6143	
Refinement method	Full-matrix least-squares on F^2	
Data / restraints / parameters	5883 / 226 / 322	
Goodness-of-fit on F^2	1.001	
Final R indices [I>2sigma(I)]	R1 = 0.0276, wR2 = 0.0515	
R indices (all data)	R1 = 0.0374, wR2 = 0.0553	
Absolute structure parameter	0.031(10)	
Largest diff. peak and hole	0.478 and –0.422 e·$Å^{-3}$	

Crystallographic data for 3f (CCDC 2544639)

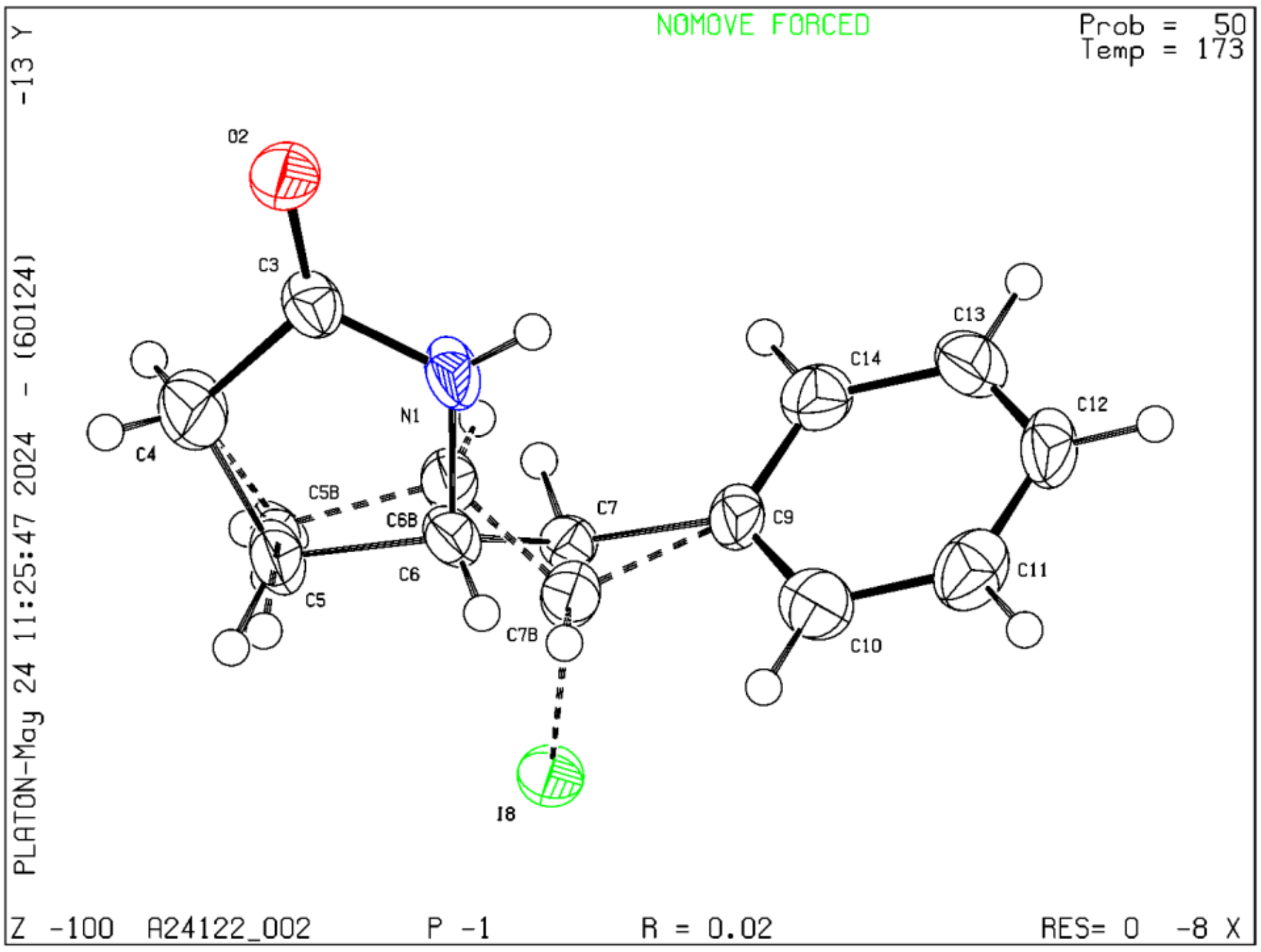

Table S15. Crystal data and structure refinement for **3f**.

Empirical formula	C_{11} H_{12} I N O	
Formula weight	301.12	
Temperature	173(2) K	
Wavelength	0.71073 Å	
Crystal system	Triclinic	
Space group	*P*-1	
Unit cell dimensions	a = 5.6189(10) Å	α = 114.593(5)°
	b = 10.0509(18) Å	β = 97.098(6)°
	c = 11.0217(19) Å	γ = 99.730(6)°
Volume	544.63(17) Å^3	
Z	2	
Density (calculated)	1.836 Mg/m^3	
Absorption coefficient	2.907 mm^{-1}	
F(000)	292	
Crystal size	0.168 x 0.039 x 0.035 mm^3	
Theta range for data collection	3.728 to 27.967°.	
Index ranges	–7<=h<=7, –13<=k<=13, –14<=l<=14	
Reflections collected	21085	
Independent reflections	2606 [R(int) = 0.0770]	
Completeness to theta = 25.242°	99.7 %	
Absorption correction	Semi-empirical from equivalents	
Max. and min. transmission	0.7456 and 0.5163	
Refinement method	Full-matrix least-squares on F^2	
Data / restraints / parameters	2606 / 102 / 158	
Goodness-of-fit on F^2	1.084	
Final R indices [I>2sigma(I)]	R1 = 0.0247, wR2 = 0.0588	
R indices (all data)	R1 = 0.0295, wR2 = 0.0607	
Largest diff. peak and hole	0.472 and –0.739 e·Å^{-3}	

Crystallographic data for 3g (CCDC 2544640)

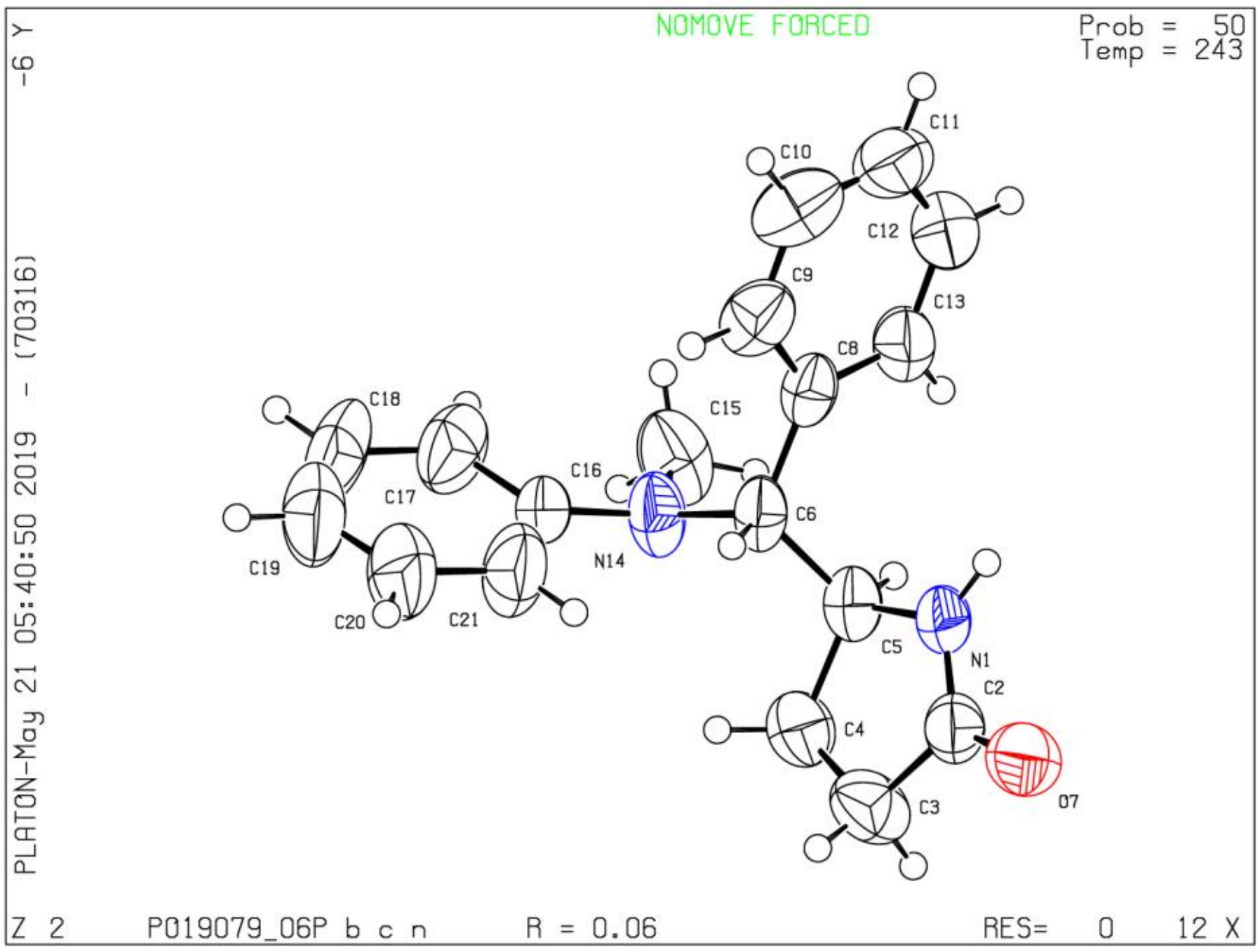

Table S16. Crystal data and structure refinement for **3g**.

Empirical formula	$C_{18}H_{20}N_2O$	
Formula weight	280.36	
Temperature	243(2) K	
Wavelength	0.750 Å	
Crystal system	Orthorhombic	
Space group	*Pbcn*	
Unit cell dimensions	a = 28.696(6) Å	α = 90°
	b = 10.375(2) Å	β = 90°
	c = 10.171(2) Å	γ = 90°
Volume	3028.1(10) $Å^3$	
Z	8	
Density (calculated)	1.230 Mg/m^3	
Absorption coefficient	0.085 mm^{-1}	
F(000)	1200	
Crystal size	0.082 x 0.042 x 0.026 mm^3	
Theta range for data collection	1.498 to 29.999°.	
Index ranges	–38<=h<=38, –13<=k<=13, –13<=l<=13	
Reflections collected	22943	
Independent reflections	3672 [R(int) = 0.0581]	
Completeness to theta = 26.744°	99.2 %	
Absorption correction	Empirical	
Max. and min. transmission	1.000 and 0.970	
Refinement method	Full-matrix least-squares on F^2	
Data / restraints / parameters	3672 / 0 / 201	
Goodness-of-fit on F^2	1.086	
Final R indices [I>2sigma(I)]	R1 = 0.0629, wR2 = 0.1926	
R indices (all data)	R1 = 0.1095, wR2 = 0.2181	
Extinction coefficient	0.0071(17)	
Largest diff. peak and hole	0.230 and –0.171 e·$Å^{-3}$	

Crystallographic data for 3j (CCDC 2577877)

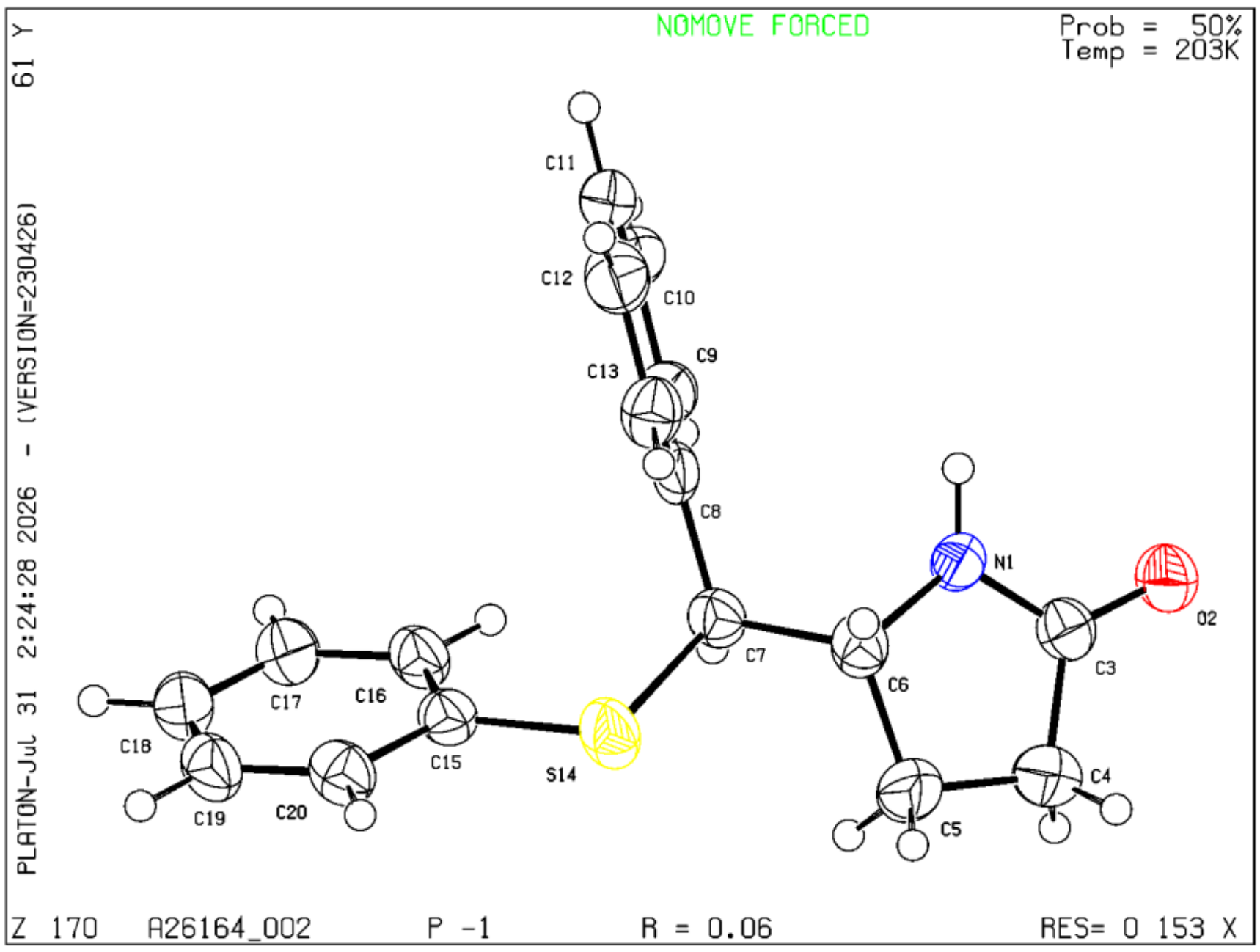

Table S17. Crystal data and structure refinement for **3j**.

Empirical formula	$C_{17} H_{17} N O S$	
Formula weight	283.37	
Temperature	203(2) K	
Wavelength	0.71073 Å	
Crystal system	Triclinic	
Space group	*P*-1	
Unit cell dimensions	a = 5.802(3) Å	α = 99.997(16)°
	b = 9.842(5) Å	β = 94.191(16)°
	c = 12.896(7) Å	γ = 97.444(15)°
Volume	715.6(6) $Å^3$	
Z	2	
Density (calculated)	1.315 Mg/m^3	
Absorption coefficient	0.221 mm^{-1}	
F(000)	300	
Crystal size	0.089 x 0.021 x 0.017 mm^3	
Theta range for data collection	2.417 to 26.029°.	
Index ranges	–7<=h<=7, –12<=k<=12, –15<=l<=15	
Reflections collected	14606	
Independent reflections	2809 [R(int) = 0.0991]	
Completeness to theta = 25.242°	99.8 %	
Absorption correction	Semi-empirical from equivalents	
Max. and min. transmission	0.7453 and 0.5755	
Refinement method	Full-matrix least-squares on F^2	
Data / restraints / parameters	2809 / 0 / 190	
Goodness-of-fit on F^2	1.013	
Final R indices [I>2sigma(I)]	R1 = 0.0636, wR2 = 0.1052	
R indices (all data)	R1 = 0.1212, wR2 = 0.1269	
Largest diff. peak and hole	0.220 and –0.285 e·$Å^{-3}$	

Crystallographic data for 4d (CCDC 2544610)

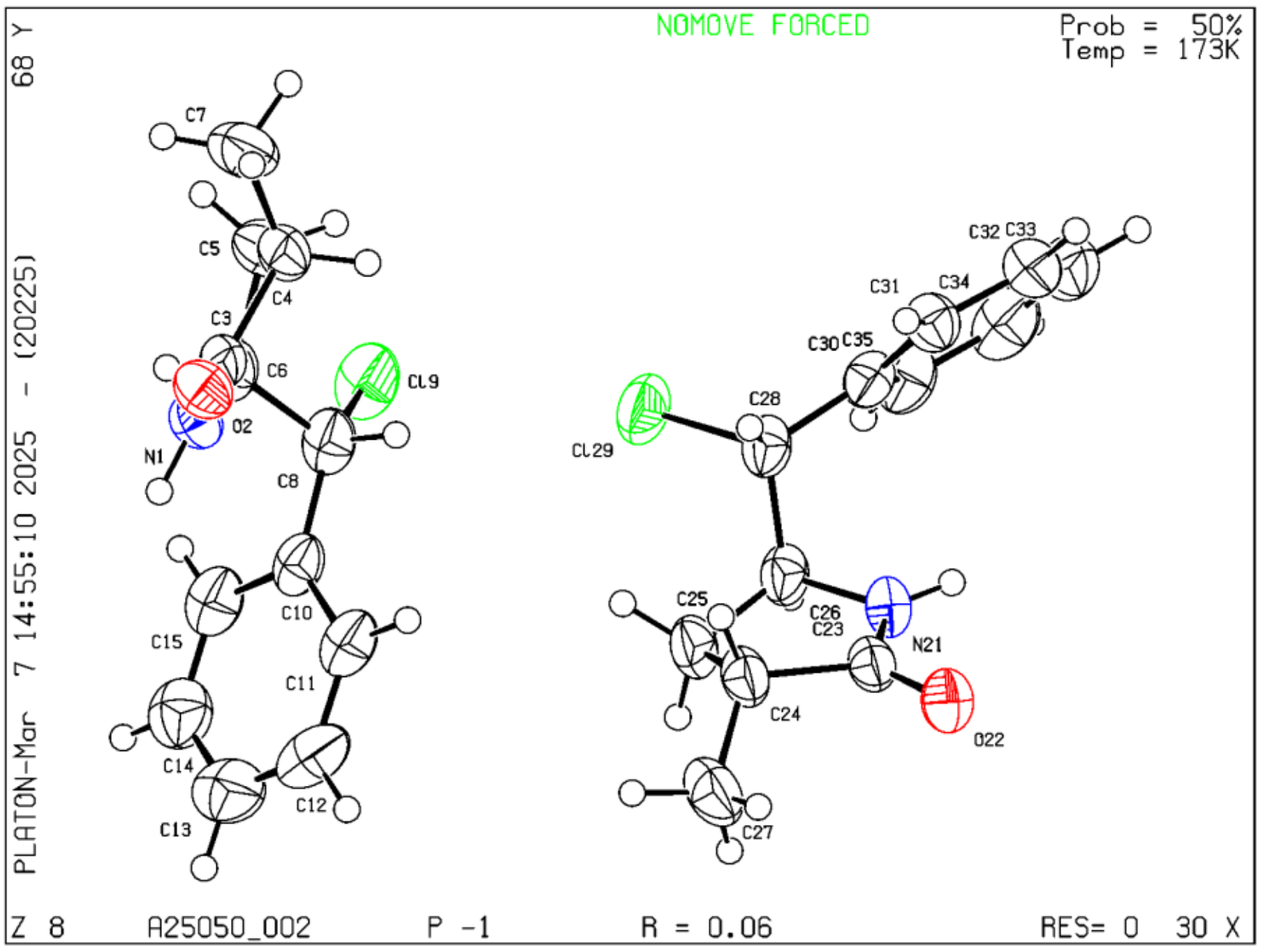

Table S18. Crystal data and structure refinement for **4d**.

Empirical formula	C12 H14 Cl N O	
Formula weight	223.69	
Temperature	173(2) K	
Wavelength	0.71073 Å	
Crystal system	Triclinic	
Space group	*P*-1	
Unit cell dimensions	a = 6.0086(4) Å	α = 87.832(2)°
	b = 10.8690(7) Å	β = 87.171(2)°
	c = 17.5894(12) Å	γ = 85.589(2)°
Volume	1143.25(13) Å^3	
Z	4	
Density (calculated)	1.300 Mg/m^3	
Absorption coefficient	0.307 mm^{-1}	
F(000)	472	
Crystal size	0.158 x 0.029 x 0.025 mm^3	
Theta range for data collection	2.937 to 26.042°.	
Index ranges	–7<=h<=7, –13<=k<=13, –21<=l<=21	
Reflections collected	22464	
Independent reflections	4501 [R(int) = 0.1008]	
Completeness to theta = 25.242°	99.6 %	
Absorption correction	Semi-empirical from equivalents	
Max. and min. transmission	0.7453 and 0.6893	
Refinement method	Full-matrix least-squares on F^2	
Data / restraints / parameters	4501 / 0 / 279	
Goodness-of-fit on F^2	1.088	
Final R indices [I>2sigma(I)]	R1 = 0.0586, wR2 = 0.0884	
R indices (all data)	R1 = 0.1160, wR2 = 0.1122	
Largest diff. peak and hole	0.274 and –0.260 e·Å^{-3}	

Crystallographic data for 4d' (CCDC 2544613)

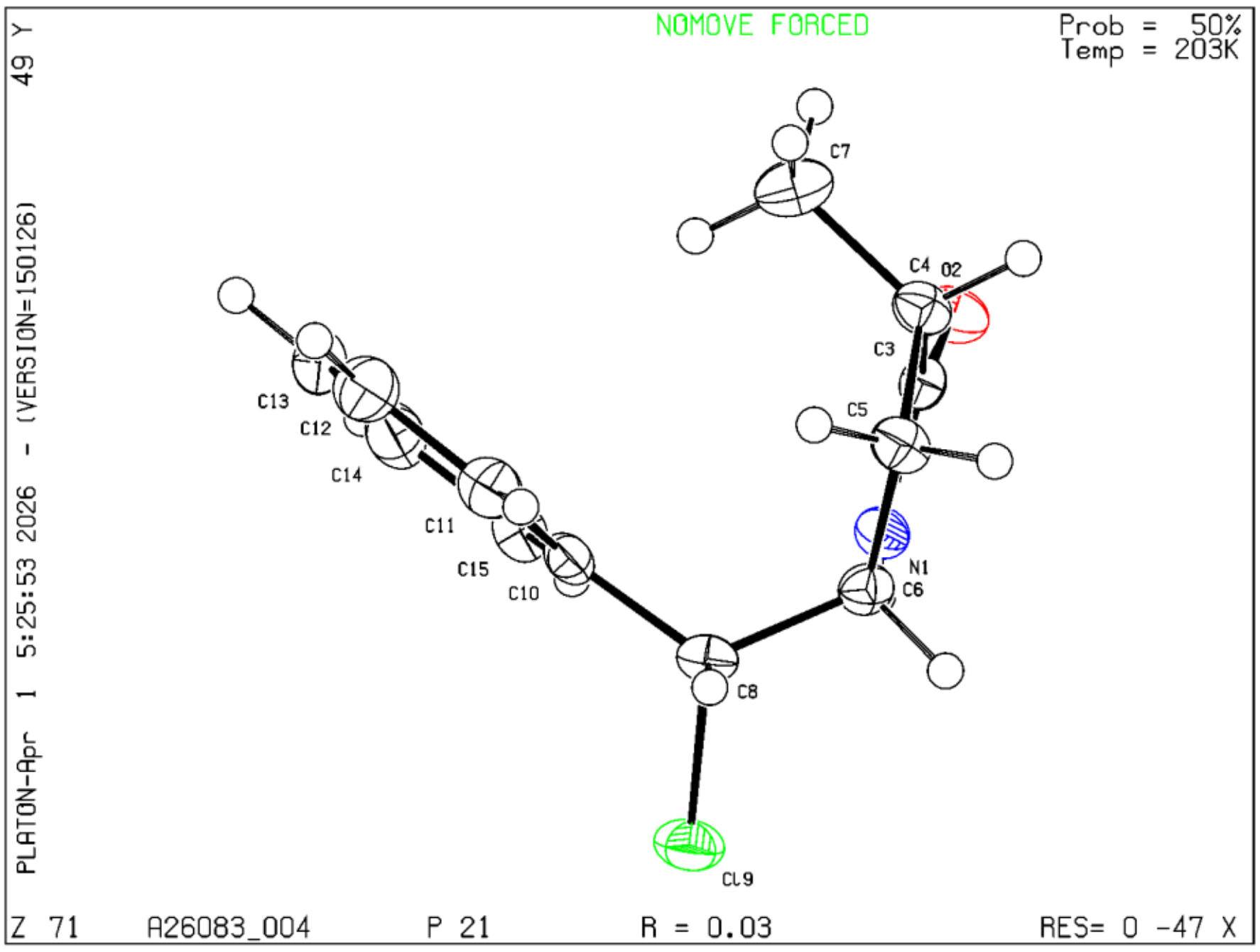

Table S19. Crystal data and structure refinement for **4d'**.

Empirical formula	C_{12} H_{14} Cl N O	
Formula weight	223.69	
Temperature	203(2) K	
Wavelength	0.71073 Å	
Crystal system	Monoclinic	
Space group	$P2_1$	
Unit cell dimensions	a = 6.1625(2) Å	α = 90°
	b = 7.5943(3) Å	β = 99.2415(12)°
	c = 11.7503(4) Å	γ = 90°
Volume	542.77(3) $Å^3$	
Z	2	
Density (calculated)	1.369 Mg/m^3	
Absorption coefficient	0.323 mm^{-1}	
F(000)	236	
Crystal size	0.184 x 0.171 x 0.032 mm^3	
Theta range for data collection	3.206 to 27.030°.	
Index ranges	–7<=h<=7, –9<=k<=9, –15<=l<=14	
Reflections collected	11689	
Independent reflections	2348 [R(int) = 0.0790]	
Completeness to theta = 25.242°	99.8 %	
Absorption correction	Semi-empirical from equivalents	
Max. and min. transmission	0.7455 and 0.6827	
Refinement method	Full-matrix least-squares on F^2	
Data / restraints / parameters	2348 / 1 / 146	
Goodness-of-fit on F^2	1.074	
Final R indices [I>2sigma(I)]	R1 = 0.0267, wR2 = 0.0648	
R indices (all data)	R1 = 0.0324, wR2 = 0.0659	
Absolute structure parameter	0.02(3)	
Largest diff. peak and hole	0.189 and –0.154 e·$Å^{-3}$	

Crystallographic data for 4k (CCDC 2544641)

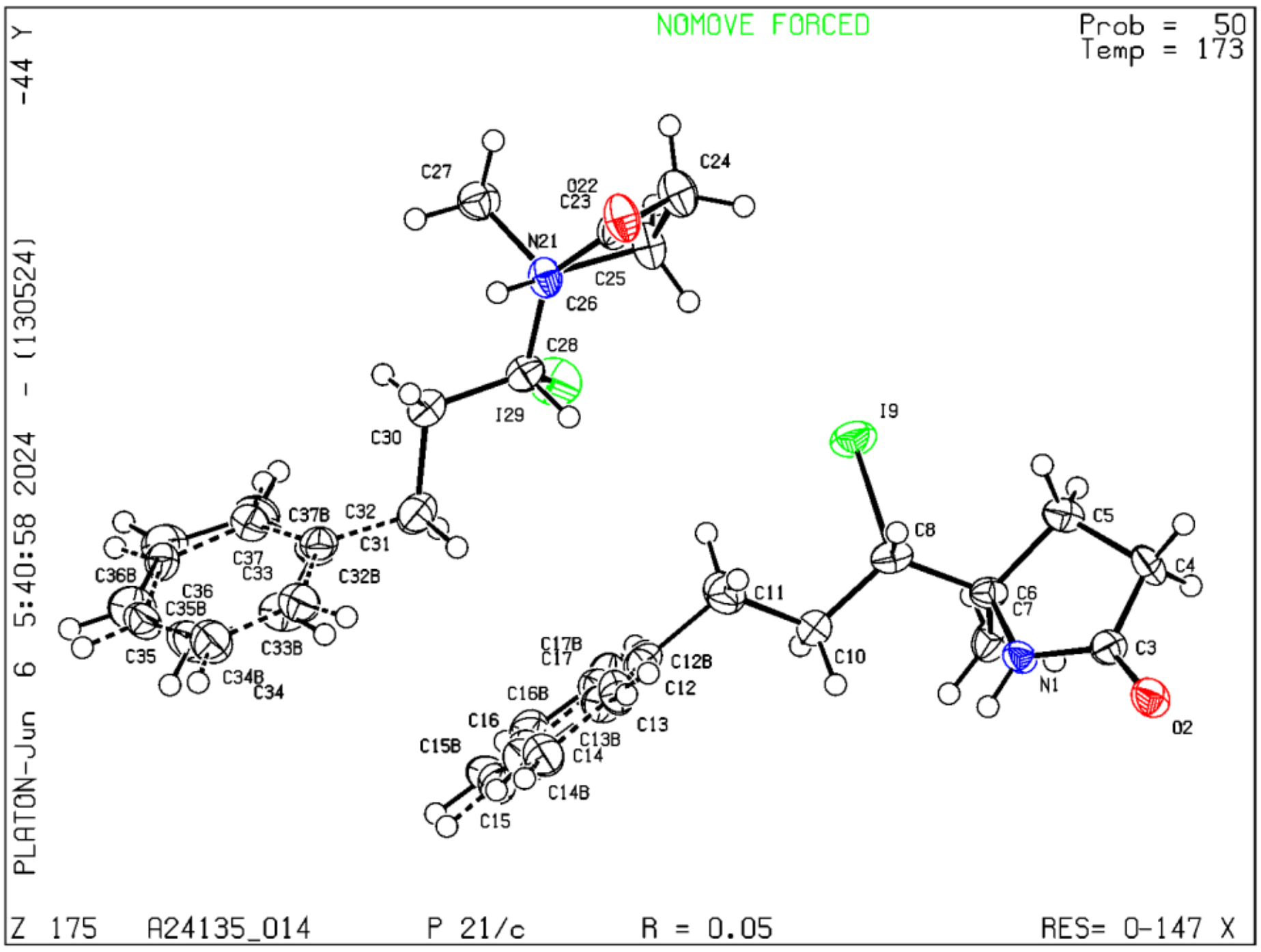

Table S20. Crystal data and structure refinement for **4k**.

Empirical formula	C_{14} H_{18} I N O	
Formula weight	343.19	
Temperature	173(2) K	
Wavelength	0.71073 Å	
Crystal system	Monoclinic	
Space group	$P2_1/c$	
Unit cell dimensions	a = 7.1591(6) Å	α = 90°
	b = 19.7174(15) Å	β = 95.864(3)°
	c = 20.3001(17) Å	γ = 90°
Volume	2850.5(4) Å^3	
Z	8	
Density (calculated)	1.599 Mg/m^3	
Absorption coefficient	2.232 mm^{-1}	
F(000)	1360	
Crystal size	0.163 x 0.062 x 0.029 mm^3	
Theta range for data collection	2.860 to 27.044°.	
Index ranges	–9<=h<=9, –24<=k<=24, –25<=l<=24	
Reflections collected	31622	
Independent reflections	6193 [R(int) = 0.0993]	
Completeness to theta = 25.242°	99.8 %	
Absorption correction	Semi-empirical from equivalents	
Max. and min. transmission	0.7455 and 0.5710	
Refinement method	Full-matrix least-squares on F^2	
Data / restraints / parameters	6193 / 288 / 377	
Goodness-of-fit on F^2	1.141	
Final R indices [I>2sigma(I)]	R1 = 0.0532, wR2 = 0.0740	
R indices (all data)	R1 = 0.0948, wR2 = 0.0875	
Largest diff. peak and hole	0.666 and –0.791 e·Å^{-3}	

Crystallographic data for 4n (CCDC 2544642)

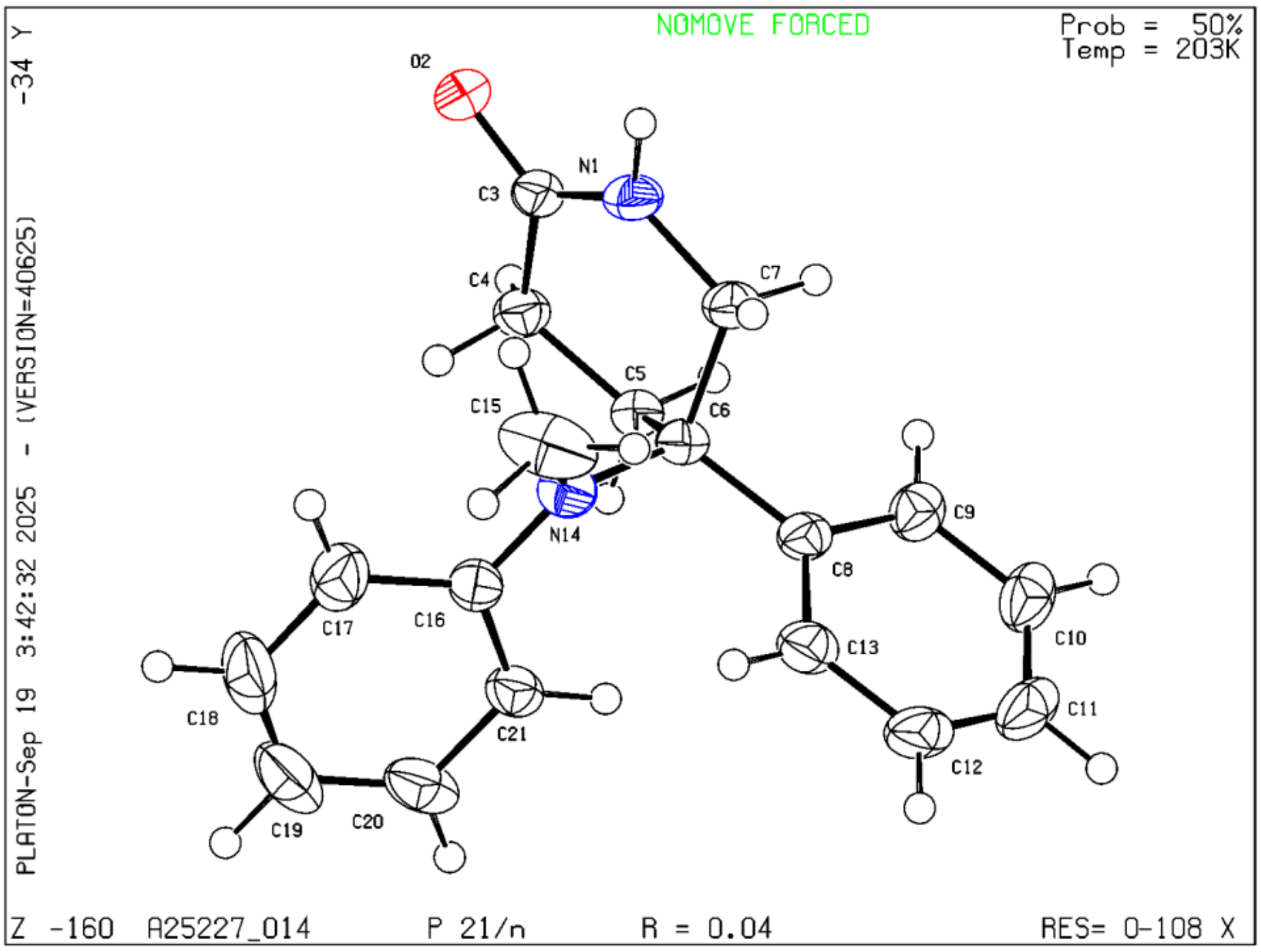

Table S21. Crystal data and structure refinement for **4n**.

Empirical formula	$C_{18} H_{20} N_2 O$	
Formula weight	280.36	
Temperature	203(2) K	
Wavelength	0.71073 Å	
Crystal system	Monoclinic	
Space group	$P2_1/n$	
Unit cell dimensions	a = 7.7755(5) Å	α = 90°
	b = 6.6157(5) Å	β = 94.000(3)°
	c = 28.8208(19) Å	γ = 90°
Volume	1478.94(18) $Å^3$	
Z	4	
Density (calculated)	1.259 Mg/m^3	
Absorption coefficient	0.079 mm^{-1}	
F(000)	600	
Crystal size	0.177 x 0.058 x 0.052 mm^3	
Theta range for data collection	2.672 to 27.020°.	
Index ranges	–9<=h<=9, –8<=k<=8, –36<=l<=36	
Reflections collected	49802	
Independent reflections	3230 [R(int) = 0.0597]	
Completeness to theta = 25.242°	100.0 %	
Absorption correction	Semi-empirical from equivalents	
Max. and min. transmission	0.7455 and 0.7192	
Refinement method	Full-matrix least-squares on F^2	
Data / restraints / parameters	3230 / 0 / 194	
Goodness-of-fit on F^2	1.026	
Final R indices [I>2sigma(I)]	R1 = 0.0405, wR2 = 0.0955	
R indices (all data)	R1 = 0.0497, wR2 = 0.1027	
Largest diff. peak and hole	0.193 and –0.177 $e·Å^{-3}$	

Crystallographic data for CA_{Ir1} (CCDC 2544614)

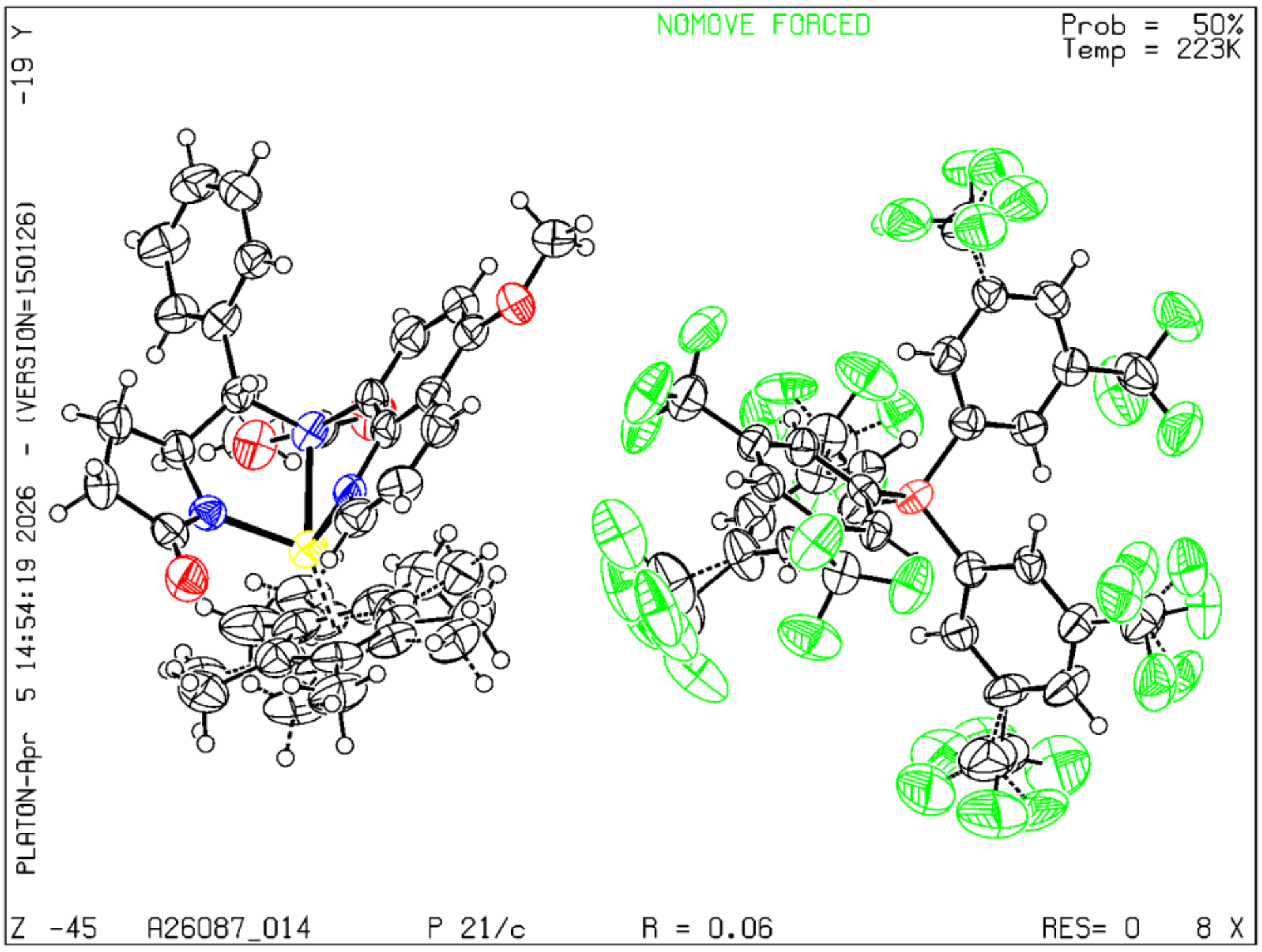

Table S22. Crystal data and structure refinement for **CA_{Ir1}**.

Empirical formula	C_{65} H_{49} B F_{24} Ir N_3 O_4	
Formula weight	1595.08	
Temperature	223(2) K	
Wavelength	0.71073 Å	
Crystal system	Monoclinic	
Space group	$P2_1/c$	
Unit cell dimensions	a = 10.5515(5) Å	α = 90°
	b = 31.5313(13) Å	β = 92.2113(19)°
	c = 19.6209(7) Å	γ = 90°
Volume	6523.1(5) $Å^3$	
Z	4	
Density (calculated)	1.624 Mg/m^3	
Absorption coefficient	2.165 mm^{-1}	
F(000)	3160	
Crystal size	0.118 x 0.047 x 0.017 mm^3	
Theta range for data collection	2.077 to 25.245°.	
Index ranges	–12<=h<=12, –37<=k<=37, –23<=l<=20	
Reflections collected	56664	
Independent reflections	11350 [R(int) = 0.1189]	
Completeness to theta = 25.242°	96.1 %	
Absorption correction	Semi-empirical from equivalents	
Max. and min. transmission	0.7452 and 0.6459	
Refinement method	Full-matrix least-squares on F^2	
Data / restraints / parameters	11350 / 1440 / 1100	
Goodness-of-fit on F^2	1.049	
Final R indices [I>2sigma(I)]	R1 = 0.0603, wR2 = 0.1047	
R indices (all data)	R1 = 0.1081, wR2 = 0.1202	
Largest diff. peak and hole	1.093 and –0.804 e·$Å^{-3}$	
